# Proceedings of Reanimation 2023, the French Intensive Care Society International Congress

**DOI:** 10.1186/s13613-023-01131-y

**Published:** 2023-06-14

## Oral Communications

## CO-01 PRESS-KT-KID: Prevalence of Severe Sepsis due to catheter related infection in children

### Manon Passard^1^, Aurélie Portefaix^2^, Yves Gillet^3^, Olivier Dauwalder^4^, Carine Halfon Domenech^5^, Etienne Javouhey^1^

#### ^1^Réanimation pédiatrique-HFME-HCL, Bron, France; ^2^CIC-HCL, Bron, France; ^3^Urgences pédiatriques-HFME-HCL, Bron, France; ^4^Laboratoire maladies infectieuses-HCL, Lyon, France; ^5^Hématologie pédiatrique-IHOPe-HCL, Lyon, France

##### **Correspondence:** Manon Passard (manon.passard@chu-lyon.fr)

*Annals of Intensive Care *2013, **13(Suppl 1):**CO-01

**Rationale:** Central lines (CL) are frequent in pediatrics but implicate some severe complications. CL infection is the main cause of nosocomial infection in pediatrics. Prevalence of CL infection varies a lot depending on the populations studied and there are no recent data regarding the prevalence of severe sepsis due to CL.

**Objective**: To estimate the prevalence and identify factors associated with occurrence of severe sepsis due to CL associated bloodstream infection (CLABSI) in pediatrics.

**Patients and methods/materials and methods:** We conducted a 4-year multicentric retrospective cohort study including all children with confirmed CLABSI from birth to 18 years old, hospitalized in all pediatric units in two university hospitals. Neonatalogy units were excluded. Patients were screened from bacteriological datas. Epidemiology and bacteriology of CLABSI were described. To find potential factors associated with severe sepsis due to CLABSI we performed univariate and multivariate analysis.

**Results:** Over 4476 suspected CL infections, we included 372 CLABSI. The prevalence of severe sepsis due to CLABSI was 17.2% (15.25–19.16%) among confirmed CLABSI and 1.43% (0.02–2.84%) among suspected CL infections. 12 children died because of severe sepsis due to CLABSI. In univariate analysis, factors associated with the occurrence of severe sepsis due to CLABSI were antibiotic therapy within 3 weeks before, known bacterial colonization, CL daily use and hospitalization in intensive care unit when the CLABSI occurred. Tachycardia and hypotension at the initial presentation, augmentation of lactate and procalcitonin at the initial biological sample were associated with the occurrence of severe sepsis due to CLABSI. In multivariate analysis, daily use of CL, know bacterial colonization, tachycardia and hypotension were associated with the occurrence of severe sepsis due to CLABSI. We couldn’t evaluate the dosage of lactate and procalcitonin due to a lack of power. There was an augmentation of gram-negative bacillus proportion among the severe sepsis due to CLABSI.

**Conclusion:** Severe sepsis prevalence in children with documented CLABSI is close to 20%. We identified factors associated with the occurrence of severe sepsis complicating CL infection. Those factors should be tested in a prospective multicenter study, to improve our ability to identify patients at risk of severe sepsis and to prevent the evolution to septic shock. This retrospective study is the first step to a prospective multicenter study aiming to optimize the initial treatment and identify children at risk to develop severe sepsis due to CLABSI, in order to improve sepsis bundles application.

**Reference 1**

Niedner MF, Huskins WC, Colantuoni E, Muschelli J, Harris JM, Rice TB, et al. Epidemiology of Central Line-Associated Bloodstream Infections in the Pediatric Intensive Care Unit. Infect Control Hosp Epidemiol. déc 2011;32(12):1200?8.

**Reference 2**

Ziegler MJ, Pellegrini DC, Safdar N. Attributable mortality of central line associated bloodstream infection: systematic review and meta-analysis. Infection. févr 2015;43(1):29?36.

**Compliance with ethics regulations:** Yes in clinical research.

**Table 1 (abstract CO-01):**
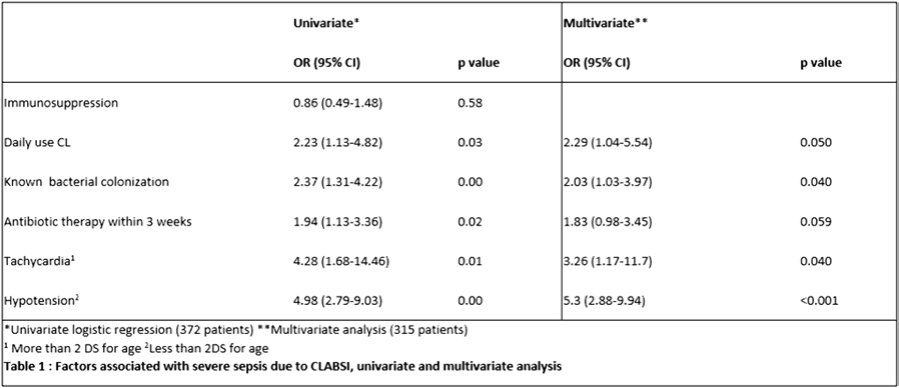
Factors associated with severe sepsis due to CLABSI, univariate and multivariate analysis

## CO-02 Fluid Resuscitation for Suspected Sepsis in Pediatric Emergency Departments (FRESSPED): a prospective multicenter study

### Julian San Geroteo^1^, Stéphane Dauger^1^

#### ^1^Hôpital Robert-Debré, AP-HP, Paris, France

##### **Correspondence:** Julian San Geroteo (julian.sangeroteo@gmail.com)

*Annals of Intensive Care *2013, **13(Suppl 1):**CO-02

**Rationale:** Pediatric sepsis is the leading cause of mortality in children under five years of age. The latest international guidelines of the Surviving Sepsis Campaign 2020 (SSC-2020) have revised the management of paediatric sepsis, particularly concerning fluid resuscitation. Although these guidelines are important, no study has evaluated their application in pediatric emergency departments. The aim of the study was to assess physician adherence to the SSC-2020 fluid resuscitation guidelines in infants and children with suspected sepsis in Pediatric Emergency Departments (PED).

**Patients and methods/materials and methods: **Fluid REsuscitation for Suspected Sepsis in Pediatric Emergency Departments (FRESSPED) was a prospective multicenter observational study conducted in 21 French hospitals over 5 sequential weeks, between November 2021 and March 2022. Children with suspected sepsis and who received anti-infectious treatment within 72 h were included. Severe sepsis was confirmed using the 2005 consensus criteria in patients admitted to PICU according to a review of each medical file by pediatric intensivist. Primary outcome was SSC-2020 fluid resuscitation guidelines adherence (low 0–24%, moderate 25–74% or high 75–100%) according to: volume of 10–20 ml/kg for each bolus; exclusive administration of balanced crystalloids at 1 h and 24 h of management; and initiation of fluid resuscitation within one hour of sepsis recognition. An electronic survey was sent to all the PEDs’physicians working to report their adherence to SSC-2020.

**Results: **A total of 63 patients were included, 10 of whom had confirmed sepsis. Median age [IQR] was 7.9 months [2.8–35.9] and a comorbidity was present in 19 children (30.1%). Primary site of infection was respiratory in half of the cases (50.8%) and unknown in 8 (12.7%) subjects. Microbiological documentation was found in 47 (74.6%) cases. Compared to the SSC-2020 guidelines: 43 (68.3%) patients received boluses of 10 to 20 ml/kg; exclusive administration of balanced crystalloids was found in 35 (55.6%) and 34 (54.8%) children at one hour and twenty-four hours of management respectively; and fluid resuscitation was initiated within one hour of sepsis recognition in 42 (76.4%) cases. Main barriers reported by physicians to such adherence were: difficult intravenous access (43.2%); lack of team training (28.8%); workload constraints (28.0%); absence or out-of-date protocols (24.0%).

**Conclusion: **FRESSPED study found high adherence for fluid resuscitation initiation and moderate adherence for bolus volume and fluid choice. Implementation of up-to-date standardized management protocols and teaching programs could improve adherence and outcomes of suspected sepsis children.

**Compliance with ethics regulations:** Yes in clinical research.

## CO-03 Epidemiology of Children admitted to Pediatric Intensive Care Unit for Sickle Cell Disease associated Complication

### Michael Levy^1^, Jérôme Naudin^1^, Guillaume Geslain^1^, Géraldine Poncelet^1^, Fleur Le Bourgeois^1^, Anna Deho^1^, Maryline Chomton^1^, Arielle Maroni^1^, Stéphane Dauger^1^, Sébastien Julliand^1^, Julie Sommet^1^

#### ^1^Hôpital Universitaire Robert-Debré, Paris, France

##### **Correspondence:** Michael Levy (michael.levy@aphp.fr)

*Annals of Intensive Care *2013, **13(Suppl 1):**CO-03

**Rationale:** Sickle cell disease (SCD) is one of the most frequent inherited diseases in France and in the world. Over the last decades, in high-income countries, an important decrease in mortality have been observed due to the improved of care. However, children with SCD can become critically ill and require admission in Pediatric Intensive Care Units (PICU). Only a few studies with small samples have focused on children with SCD admitted to PICU and robust data regarding the epidemiology of these patients are missing. The aim of this study was to describe the epidemiology of children admitted to PICU for acute complications of SCD and their outcomes.

**Patients and methods/materials and methods:** We conducted a single center retrospective study in a tertiary center PICU. Consecutive children with SCD admitted to PICU between January 1st, 2009 and December 31, 2019 were included. Patients with sickle cell trait (heterozygous for the causative gene) or that have been admitted following bone-marrow transplant were not included.

**Results:** 582 children with SCD were admitted to PICU during the study period. The median age was 9.2 [5.5–13.4] years and 60% were boys. 538 (93%) were of SS genotype and the baseline hemoglobin was 8.4 g/dl. 42% had a history of PICU admission and 51% of acute chest syndrome (ACS). Patients were mainly admitted from pediatric ward (n = 440, 76%) and emergency units (n = 114, 19.5%). ACS was the main reason of admission (54%) followed by vaso-occlusive crisis (17%) and stroke (17%). 492 (85%) were treated with antibiotics, 293 (50%) patients required non-invasive ventilation and 47 (8%) invasive ventilation. Cardiovascular failure was rare (2%). 223 (38%) patients required blood transfusion and 155 (27%) exchange transfusion. The median duration of PICU stay was 3 [1–4] days. The mortality rate at PICU discharge was 1% (n = 7) and 1% (n = 8) after 6 months.

**Conclusion:** Children with SCD hospitalized in PICU were mainly admitted for acute chest syndrome and half of patients required non-invasive ventilation. The median length of stay was short and the mortality low.

**Compliance with ethics regulations:** Yes in clinical research.

## CO-04 Pharmacist optimization of the therapeutic management of patients on ECMO in the pediatric intensive care unit

### Omar Hanafia^1^, Héloïse Capelle^2^, Julie Leonelli^1^, Pierre Bertault-Peres^1^, Stéphane Honore^3^, Fabrice Michel^1^

#### ^1^Hôpitaux Universitaires de Marseille/AP-HM, Marseille, France; ^2^CH Aubagne, Abagne, France; ^3^Aix Marseille Université/Faculté de Pharmacie, Marseille, France

##### **Correspondence:** Omar Hanafia (omarhanafia@yahoo.fr)

*Annals of Intensive Care *2013, **13(Suppl 1):**CO-04

**Rationale:** Extracorporeal Membrane Oxygenation (ECMO) is a rescue technique that allows the replacement of circulatory and/or respiratory functions. The pharmacokinetic modifications generated by this circulatory assistance require a dosage adaptation of certain drugs. The objective of the study was to compare the drug prescription of patients under ECMO and to propose appropriate dosages adjustments.

**Patients and methods/materials and methods:** Our 6-months prospective observational monocentric study focuses on critically ill patients assisted by ECMO. Clinico-biological data were collected from the computerized patient record and by the daily presence of the pharmacist in the department. We noted the type and indication of ECMO, complications and adequacy of dosages compared to the literature for relevance.

**Results:** Fourteen patients with ECMO were included: mean age 18 months [0 to 168 months], sex ratio = 1. Renal function was impaired in 8 patients (57%). The average duration of ECMO was 15 days [3–24 days]. 6 patients were weaned, 4 of whom were still hospitalized on the ward (43%) and 8 patients died (57%). 13 patients (93%) were on veno-arterial ECMO, following acute respiratory distress syndrome (8 cases or 61%), refractory cardiac arrest (3 cases 23%), cardiogenic shock (8%) or septic shock (8%). 1 patient (7%) was on veno-venous ECMO following acute respiratory distress syndrome (ARDS). Elevent patients (79%) developed complications related to ECMO (9 hemorrhages, 8 hemolysis, 6 oxygenation difficulties, 5 PAO, 4 stroke). Concerning the drug management of these patients, we counted 16 overdosages and 2 underdosages not justified according to the literature. There was also no dosage that would explain these discrepancies, i.e. 18 nonconformities out of 73 lines analyzed concerning Vancomycin, Gentamicin, Fluconazole, Caspofungin, Voriconazole, Ganciclovir, Heparin, Morphine, Sufentanil, Midazolam, Cisatracurium, Hydrocortisone Hemisuccinate, Methadone.

**Discussion:** The populations studied in the literature remain different from ours, making it difficult to discuss our clinical results.

**Conclusion:** However, following the non-conformities of dosage noted, the pharmacist proposed a table of dosage adaptation under ECMO synthesizing the literature for the studied molecules which is systematically accompanied by instructions to make a therapeutic drug monitoring.

**Compliance with ethics regulations:** N/A.

## CO-05 Paediatric traumatic out-of-hospital cardiac arrest is associated with lower survival rates compared to those of medical aetiology: Results from the French national registry

### Marguerite Lockhart-Bouron^1^, Valentine Canon^2^, Stéphane Leteurtre^1^, Hervé Hubert^2^, Morgan Recher^1^

#### ^1^Centre hospitalier universitaire de Lille, Lille, France; ^2^Université de Lille, Lille, France

##### **Correspondence:** Morgan Recher (morgan.recher@chu-lille.fr)

*Annals of Intensive Care *2013, **13(Suppl 1):**CO-05

**Rationale:** Trauma is an important cause of paediatric out-of-hospital cardiac arrest with a high mortality rate. The first aim of this study was to compare the survival rate at day 30 and at hospital discharge following paediatric traumatic and medical out-of-hospital cardiac arrest. The second aim was to compare the rates of return of spontaneous circulation and survival rates at hospital admission (day 0).

**Patients and methods/materials and methods:** This multicentre comparative post-hoc study was conducted between July 2011 and February 2022 based on the French National Cardiac Arrest Registry data. All patients aged < 18 years with out-of-hospital cardiac arrest were included in the study. Patients with traumatic aetiology were matched with those with medical aetiology using propensity score matching.

**Results:** There were 398 traumatic and 1,061 medical out-of-hospital cardiac arrests. Matching yielded 227 pairs. In non-adjusted comparisons, days 0 and 30 survival rates were lower in the traumatic aetiology group than in the medical aetiology group (19.1% vs. 24.0%, odds ratio (OR) 0.75, 95% confidence interval (CI) 0.56–0.99, and 2.0% vs. 4.5%, OR 0.43, 95% CI 0.20–0.92, respectively). In adjusted comparisons, day 30 survival rate was lower in the traumatic aetiology group than in the medical aetiology group (2.2% vs. 6.2%, OR 0.36, 95% CI 0.13–0.99).

**Conclusion:** In paediatric out-of-hospital cardiac arrest patients, the adjusted survival rates at day 30 for those with a traumatic aetiology were lower than those with a medical aetiology. Prioritising trauma-specific interventions that address possible reversible causes of cardiac arrest may lead to improved survival in this population.

**Compliance with ethics regulations:** Yes in clinical research.

**Figure 1 (abstract CO-05):**
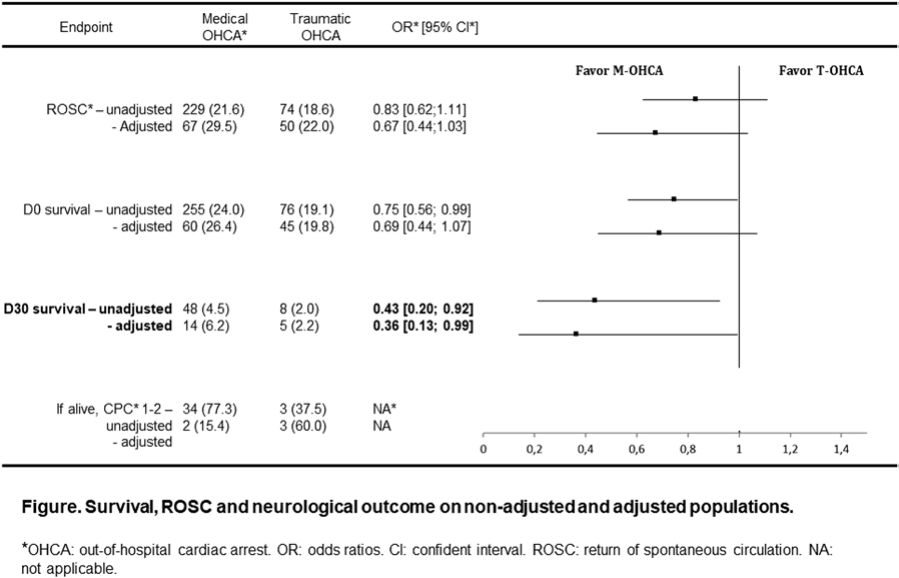
Survival, ROSC and neurological outcome on non-adjusted and adjusted populations

## CO-06 Incidence of refeeding syndrome in pediatric intensive care

### Kaoutar El Fakhr^1^, Wissal Aissaoui^1^, Samira Kalouch^1^, Abdelaziz Chlilek^1^

#### ^1^CHU Ibn Rochd, Casablanca, Maroc

##### **Correspondence:** Kaoutar El Fakhr (kaoutarelfakhr2013@gmail.com)

*Annals of Intensive Care *2013, **13(Suppl 1):**CO-06

**Rationale:** Refeeding syndrome (RFS) describes a potentially fatal physiological response to the increase in energy from any source including: intravenous, oral food, enteral nutrition or parenteral nutrition following a period of starvation or severely reduced energy intake, reflecting a change from catabolism to anabolism following the release of insulin. However, data on its incidence is lacking, and the heterogeneity of diagnostic criteria and frequent electrolyte disorders in this population make its diagnosis complex. In 2020, the American Society for Parenteral and Enteral Nutrition (ASPEN) developed consensus recommendations for identifying patients at risk and with refeeding syndrome. The aim of this study was to determine the incidence of refeeding syndrome in critically ill children on nutritional support.

**Patients and methods/materials and methods:** A prospective cohort spread over three years from January 2020-January 2023, conducted in a tertiary pediatric intensive care unit was undertaken, and additional data were retrospectively collected.

**Results:** A total of 2058 children were included in the study, with 350 children (17%) classified as undernourished, who were at risk of refeeding syndrome (142F, 158 M). Over a period of 3 years, 50 children of these were identified as having refeeding syndrome, giving a cumulative incidence of 14.28%; IC95% [0.106–0.179], aged 12 days -17 years (median 24SD + -62.7 months). Patients with refeeding syndrome have a higher risk of mortality (odds ratio [OR], 6.75; CI, 3.18–14.3; P = 0.001). Children who were fed late > 5 days are more likely to have refeeding syndrome (odds ratio [OR], 5.33; CI, 3.13–9.8; P = 0.001), children who received early feeding < 5 days are less likely to have refeeding syndrome (odds ratio [OR], 0.18; CI, 0.11–0.32; P = 0.001).

**Conclusion:** Refeeding syndrome is a serious, life-threatening metabolic complication that can lead to increased morbidity and mortality.

**Compliance with ethics regulations:** Yes in clinical research.

## CO-07 Impact of systemic causes of secondary brain injury factors on the short-term prognosis of critically ill patients with sepsis-associated encephalopathy

### Michael Thy^1,2,3^, Romain Sonneville^1,2,4^, Stéphane Ruckly^4^, Claire Dupuis^5^, Julien Poujade^1^, Lila Bouadma^1,2,4^, Etienne De Montmollin^1,2,4^, Jean-François Timsit^1,2,4^

#### ^1^Department of medical and infectious intensive care unit, Bichat-Claude Bernard Hospital-AP-HP Nord, Paris, France; ^2^Université Paris Cité, Paris, France; ^3^EA 7323-Pharmacology and Therapeutic Evaluation in Children and Pregnant Women, Paris, France; ^4^INSERM, UMR 1137, IAME, Paris, France; ^5^Service de Médecine Intensive et Réanimation, CHU de Clermont-Ferrand, Clermont-Ferrand, France

##### **Correspondence:** Michael Thy (michael245thy@gmail.com)

*Annals of Intensive Care *2013, **13(Suppl 1):**CO-07

**Rationale:** Sepsis is complicated in about 50% of cases by an acute encephalopathy ranging from delirium to coma (sepsis-associated encephalopathy, SAE), which is associated with poor outcome. Systemic causes of secondary brain injury (SSBI), that are frequently observed in ICU patients, may worsen outcomes of SAE. We aimed to investigate the association between the presence of at least one SSBI within the first two days of intensive care unit (ICU) admission with outcomes of SAE patients.

**Patients and methods/materials and methods:** We performed a retrospective analysis using data from the French OUTCOMEREA multicenter prospective database. We included consecutive SAE patients (defined by a score on the Glasgow coma scale ≤ 13 and severe sepsis or septic shock (SEPSIS 2.0 definition) criteria) requiring invasive mechanical ventilation at ICU admission. We excluded patients with infectious and non-infectious acute cerebral pathologies. For each patient, we analyzed the SSBI events (abnormal glycemia, hypotension, temperature abnormalities, anemia, dysnatremia, oxygenation abnormalities, dyscapnia) present within the first 2 days of hospitalization in ICU and the impact of their control at day 3 on day-14 mortality using a Cox-model with adjusted hazard ratio (aHR) and 95% confidence interval [95% CI].

**Results:** From 1997 to 2020, 995 patients were included in the study. Compared to decedents at D14, alive patients had significantly less SSBI events, including less hypoglycemia (< 3 mmol/L), less hypothermia (< 36 °C), less hypoxemia (PaO2 < 60 mmHg), less hypotension (DAP < 50 mmHg) and anemia (hematocrit < 21%) within the 2 first days hospitalization in ICU. After adjusting for confounders (SOFA score, type of admission, comorbid conditions), the control of the following SSBI at day 3 was significantly associated with lower day-14 mortality for blood pressure (diastolic pressure > 50 mmHg: aHR = 1.997 [1.42–2.8], p < 0.001), oxygenation (PaO2 between 60 and 200 mHg: aHR = 2.076 [1.36–3.16], p < 0.001), glycemia (glycemia between 3 and 11 mM/l: aHR = 1.604 [1.21–2.12], p < 0.001), temperature (36 °C-38.3 °C: aHR = 1.55 [1.14–2.11], p = 0.006).

**Conclusion:** We demonstrate that the control of SSBI (particularly oxygenation, arterial pressure, glycemia, temperature) improves prognosis of patients with SAE. SSBI monitoring and control should be systematically done in all septic patients with SAE.

**Compliance with ethics regulations:** Yes in clinical research.

**Table 1 (abstract CO-07):**
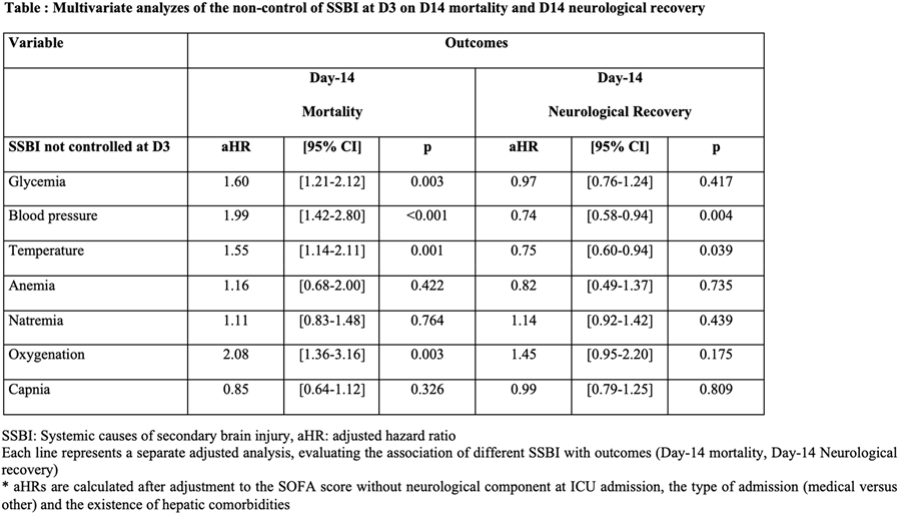
Multivariate analyzes of the non-control of SSBI at D3 on D14 mortality and D14 neurological recovery

## CO-08 Sepsis at time of venoarterial extracorporeal membrane oxygenation initiation is associated with early neurobiological and neurophysiological alterations

### Chloé Tridon^1^, Delphine Bachelet^2^, Majda El Baied^2^, Philippine Eloy^2^, Sofia Ortuno^2^, Marylou Para^2^, Paul-Henri Wicky^2^, Geoffroy Vellieux^2^, Etienne De Montmollin^2^, Lila Bouadma^2^, Hana Manceau^3^, Jean-François Timsit^2^, Katell Peoc'h^3^, Romain Sonneville^2^

#### 1Hôpital Mignot, Le Chesnay, France; 2Hôpital Bichat, Paris, France; 3Hôpital Beaujon, Clichy, France

##### **Correspondence:** Chloé Tridon (chloe.tridon12@gmail.com)

*Annals of Intensive Care *2013, **13(Suppl 1):**CO-08

**Rationale:** Neurologic outcomes of patients under venoarterial ECMO (VA-ECMO) may be worsened by secondary insults of systemic origin. We aimed to assess whether sepsis, commonly observed during ECMO support, is associated with brain injury and outcomes in these patients.

**Patients and methods/materials and methods:** We conducted a single-center cohort "exposed-non-exposed" type study (2013–2020) in a university hospital in consecutive adult patients treated by VA-ECMO. Patients with "sepsis" at the time of VA-ECMO cannulation were compared with "non-sepsis" patients. Main outcome measures included clinical status, NSE and S100β blood concentrations, and EEG parameters measured within 72 h of VA-ECMO cannulation. We investigated the association between sepsis and poor functional outcome at 90 days (modified Rankin scale (mRS) score ≥ 4) in adjusted (logistic regression, inverse probability of treatment weighting (IPTW)) analyses. Subgroup analyses were conducted in patients with or without pre-ECMO cardiac arrest.

**Results:** A total of 196 patients was included (“sepsis”, n = 128; “non-sepsis”, n = 68), of whom 87 (44.4%) presented cardiac arrest before VA-ECMO cannulation. Compared to the “non-sepsis” group, “sepsis” patients presented a significant increase in the S100β protein concentrations at day 1 (0.94 μg/L versus 0.52 μg/L, p = 0.03), and more frequent EEG alterations (i.e., severe slowing, discontinuous background, and a lower prevalence of sleep patterns). A poor functional outcome was observed in 99/128 (77.3%) patients of the "sepsis" group and 46/68 (67.6%) of the "non-sepsis" group (adjusted logistic regression OR 1.21, 95%CI 0.58–2.47; IPTW OR 1.24, 95%CI 0.79–1.95). Sensitivity analyses suggested that sepsis was independently associated with poorer functional outcomes in the “pre-ECMO cardiac arrest” subgroup (adjusted logistic regression OR 3.44, 95%CI 1.06–11.40; IPTW OR 3.52, 95%CI 1.68 -7.73), whereas no such association was observed in patients without pre-ECMO cardiac arrest (adjusted logistic regression OR 0.69, 95%CI 0.27 -1.69; IPTW OR 0.76, 95%CI 0.42–1.35).

**Conclusion:** Sepsis at the time of VA-ECMO initiation is associated with early biochemical and electrophysiological alterations. We observed a detrimental role of sepsis on neurologic outcomes in the subgroup of patients who had experienced pre-ECMO cardiac arrest, but not in other patients.

**Compliance with ethics regulations:** Yes in clinical research.

## CO-09 Effect of adding erythromycin on mortality and sepsis biomarkers in ICU septic patients: randomized trial

### Ahlem Trifi^1^, Badis Tlili^1^, Hounaida Galai^1^, Salma Ghalloussi^1^, Salma Kamoun^1^, Emir Bedhiafi ^1^, Ahmed Albattrawi^1^, Eya Seghir^1^, Lynda Messaoud^1^, Asma Mehdi^1^, Sami Abdellatif^1^, Salah Ben Lakhal^1^

#### ^1^Medical ICU, Hôpital la Rabta, Tunis, Tunisie

##### **Correspondence:** Ahlem Trifi (trifiahlem2@gmail.com)

*Annals of Intensive Care *2013, **13(Suppl 1):**CO-09

**Rationale:** In sepsis, the imbalance of the immune-inflammatory response is a source of organ dysfunction. Immunomodulatory therapies have been tried in order to moderate this imbalance and improve the outcome of critically septic patients. We sought to determine whether the adjunction of erythromycin (a macrolide antibiotic known to have immunomodulatory effects) in septic patients has a regulatory effect on the infection biomarkers and decreases mortality.

**Patients and methods/materials and methods:** We performed a single-blind randomized trial comparing erythromycin 1 gx3/d intravenously for 5 days versus placebo for 5 days in adult patients admitted to intensive care with. The exclusion criteria was death within 5 days of the protocol. Inflammatory markers (whole blood count, CRP and procalcitonin) were assayed on inclusion (D0) and on D6 (end of prescription). The primary outcome was 28-day mortality and the secondary outcomes were changes in infection biomarkers between D0-D6, vasopressor requirements, ventilation use and duration, and length of stay. [ClinicalTrials.gov ID: NCT04665089].

**Results:** One hundrer and nine patients were enrolled, of whom 80 were included (erythromycin group, n = 40 and placebo group, n = 40). The male sex was predominant in the first group (31 (77.5%) vs 20 (50%), p = 0.01). The other baseline characteristics were similar (age = 63 [51–78] vs 60 [50–84], p = 0.78; SOFA = 9 vs 7.5, p = 0.19; diabetes: 30% vs 50%, p = 0.11 and cardiovascular diseases: 32.5% vs 60%, p = 0.37) in erythromycin versus placebo groups respectively. There was no difference on the time of occurrence of sepsis (6.5 [5–10] d vs 5.5 [2–16] d, p = 0.16) nor on the nosocomial nature (47,5% vs 58%, p = 0.37). No favorable effect of adding erythromycin in septic patients was found compared to placebo on mortality (45% vs 37.5%, p = 0.63) nor on the other parameters. Concerning the biomarkers, they were similar at baseline. At the end of the protocol, no difference was showed. In the intragroup analysis, both procalcitonin and CRP decreased significantly from day 0 to day 6 in the erythromycin arm (from 2.47 to 1.14 ug/l, p < 10–3 and from 172 to 109 mg/l, p = 0.001 respectively). In the placebo arm, only procalcitonin decreased (attached table). No conductive disorders or QT segment abnormalities were described in the erythromycin group.

**Conclusion:** the adjunction of erythromycin did not reduce mortality but it conducted to a better control of the inflammatory response, in particular the reduction of procalcitonin and CRP.

**Compliance with ethics regulations:** Yes in clinical research.

**Table 1 (abstract CO-09):**
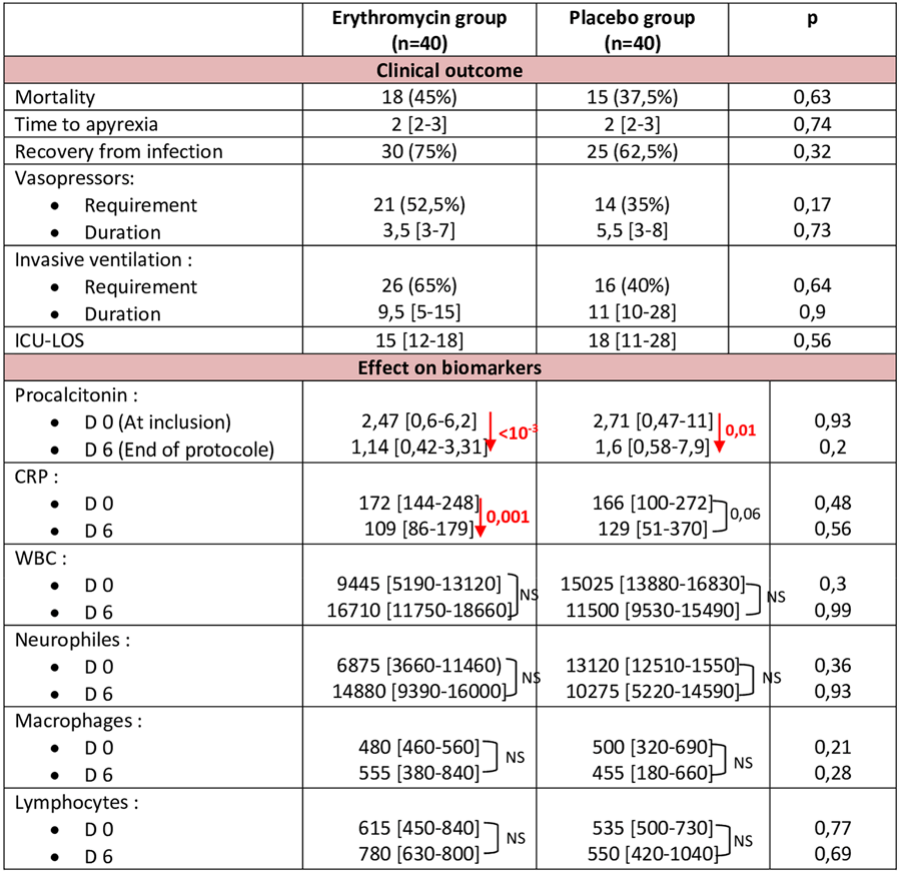
Assessment of adding erythromycin on the clinical outcome and on biomarkers

## CO-10 Interest of early mobilization by cycloergometry in patients with septic shock

### Gaëtan Béduneau^1^, Valentin Harter^8^, Emilie Occhiali^1^, Antoine Dewitte^2^, Thierry Boulain^3^, Richard Galliot^4^, Olivier Guisset^5^, Julien Charpentier^6^, Bertrand Rozec^7^, Baptiste Michaux^1^

#### ^1^CHU Charles Nicolle, Rouen, France; ^2^CHU Groupe Hospitalier Sud Haut Lévêque, Bordeaux, France; ^3^CHR, Orléans, France; ^4^Hôpital Foch, Suresnes, France ; ^5^CHU Saint André, Bordeaux, France; ^6^Hôpital Cochin, Service MIR, Groupe Hospitalier Paris Centre, Université Paris Cité, Paris, France; ^7^Hôpital Nord Laennec, Nantes, France; ^8^Centre François Baclesse, Caen, France

##### **Correspondence:** Baptiste Michaux (baptiste.michaux@chu-rouen.fr)

**Rationale:** Acquired weakness in intensive care unit (ICU), particularly favored by multi-organ failure caused by septic shock and prolonged bed rest, is responsible for an increase in duration of weaning from mechanical ventilation, duration of hospitalization and morbi-mortality. In addition to the usual physiotherapy, cycloergometry sessions are proposed. A recent meta-analysis however did not show superiority of these sessions (1). The main objective of this study was to determine impact on ICU length of stay in patients with septic shock of early mobilization by cycloergometer in association with standard physiotherapy.

**Patients and methods/materials and methods:** This is a prospective, randomized, open multicenter study, which included patients with a septic shock, invasive ventilation and sedation, hemodynamically stabilized. At inclusion, patients were randomized in intervention group (cycloergometer (20 min/day) added to standard physiotherapy) or in control group (standard physiotherapy alone). In both groups, physiotherapy started as soon as the patients were included and was adapted to the patient's condition. Patients were randomized a second time at the first lifting of sedation. This double randomization constituted a dataset for the endpoints analysis from hemodynamic stability to the first lifting of sedations (phase I) and another dataset for unbiased endpoints analysis from lifting of sedations to the discharge from ICU (phase II).

**Results:** We included 119 patients in eight ICU, with median SAPS II and SOFA (ICU admission) of respectively 60 [95%RR 23.9–93.0] and 9 [95%RR 2.00–15.00]. Characteristics at inclusion were similar. We followed 109 patients until the cessation of sedation (Phase I). Then, we re-randomized 107 patients until ICU discharge (Phase II). There was no difference regarding length of stay in ICU at each phase between the two groups (Figure 1A, B). The phase I median duration is 4 days for both groups. For phase II, the median is 9 days [6–15] days for cycloergometer group and 12 [6–28] days for control group. Patients with standard physiotherapy during the two phases spent 20 days in ICU [11–66] versus 13 days [8–33] for patients with cycloergonometry in phase I and/or phase II (p = 0.052) (Figure 1C). Similarly, the median duration of invasive mechanical ventilation was significantly higher for patients without cycloergometry (14 vs 9 days, p = 0,04). Another abstract proposes the results concerning the safety and feasibility.

**Conclusion:** Although we did not show significant difference between groups, a trend emerges concerning the increase in the length of stay for patients who do not benefit from a cycloergometry in either of the two phases.

**Reference 1**

Takaoka A, Utgikar R, Rochwerg B, Cook DJ, Kho ME. The Efficacy and Safety of In-Intensive Care Unit Leg-Cycle Ergometry in Critically Ill Adults. A Systematic Review and Meta-analysis. Ann Am Thorac Soc. oct 2020;17(10):1289?307.

**Compliance with ethics regulations:** Yes in clinical research.

**Figure 1 (abstract CO-10):**
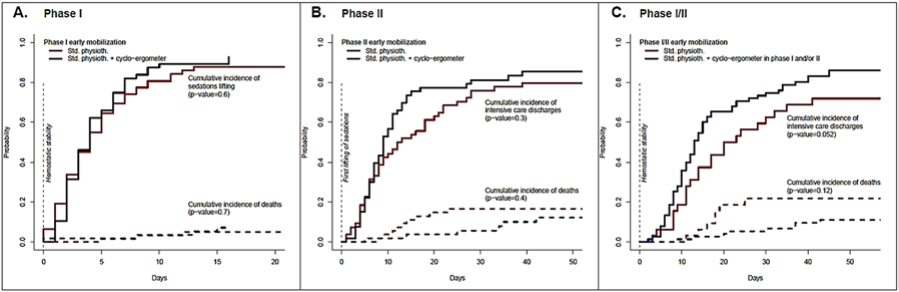
Phase I-II durations by arms

## CO-11 Characteristics and outcomes of patients admitted in ICUs for sepsis transmitted by cats or dogs: A Multicenter, Retrospective Study (PETSEPSIS)

### Juliette Quintin^1^, Aurélie Le Thuaut^1^, Fabienne Plouvier^2^, Julien Maizel^3^, Michel Sirodot^4^, Nicholas Sedillot^5^, Cécile Aubron^6^, Anthony Le Meur^7^, Bertrand Souweine^8^, Jean-Pierre Quenot^9^, Jean-Claude Lacherade^10^, Julie Noublanche^11^, Mickael Landais^12^, Didier Thevenin^13^, Saad Nseir^14^, Thomas Lafon^15^, Thierry Van Der Linden^16^, Pierre Bouju^17^, Laurent Argaud^18^, Louis Chauvelot^19^, Marlène Dubois^20^, Romaric Larcher^21^, Pierre-Yves Egreteau^22^, Jean Dellamonica^23^, Saber Barbar^24^, Mathieu Serie^25^, Thierry Boulain^26^, Frederic Jacobs^27^, Nicolas De Prost^28^, Jérôme Devaquet^29^, Nicholas Heming^30^, Marc Pineton De Chambrun^31^, Philippe Michel^32^, Michel Djibre^33^, Alexis Ferre^34^, Walter Picard^35^, Kieran Pinceaux^36^, Fabienne Tamion^37^, François Legay^38^, Maud Jonas^39^, Jessy Cattelan^40^, Stéphane Ehrmann^41^, Julien Huntzinger^42^, Christophe Cracco^43^, Olivier Michel^44^, Frederique Schortgen^45^, Philippe Letocart^46^, Jean Reignier^1^

#### ^1^CHU Nantes, Nantes, France; ^2^CH Agen-Nerac, Agen, France; ^3^CHU Amiens, Amiens, France; ^4^CH Annecy Genevois, Annecy, France; ^5^CH Bourg-En-Bresse, Bourg-En-Bresse, France; ^6^CHU Brest, Brest, France; ^7^CH Cholet, Cholet, France; ^8^CHU Clermont-Ferrand, Clermont-Ferrand, France; ^9^CHU Dijon, Dijon, France; ^10^CHD Vendée, La-Roche-Sur-Yon, France; ^11^CH La Rochelle, La Rochelle, France; ^12^CH Le Mans, Le Mans, France; ^13^CH Lens, Lens, France; ^14^CHRU Lille, Lille, France; ^15^CHU Limoges, Limoges, France; ^16^Groupement des Hôpitaux de l'Institut Catholique de Lille, Lommes, France; ^17^Groupe Hospitalier Bretagne Sud, Lorient, France ; ^18^Hôpital Edouard Herriot-Hospices Civils de Lyon, Lyon, France; ^19^Hôpital de la Croix-Rousse-Hospices Civils de Lyon, Lyon, France; ^20^CH Montauban, Montauban, France; ^21^CHU Montpellier, Montpellier, France; ^22^CH Morlaix, Morlaix, France; ^23^CHU Nice, Nice, France; ^24^CHU Nîmes, Nîmes, France ; ^25^Centre Hospitalier Territorial Gaston-Bourret, Nouméa, France; ^26^Centre hospitalier Régional Orléans, Orléans, France; ^27^AP-HP Hôpital Antoine-Béclère, Clamart, France ; ^28^CHU Créteil-Hôpital Henri Mondor, Créteil, France ; ^29^Hôpital Foch, Suresnes, France ; ^30^AP-HP Hôpital Raymond-Poincaré, Garches, France ; ^31^AP-HP Hôpitaux Universitaires Pitié Salpêtrière-Charles Foix, Paris, France ; ^32^Hôpital NOVO (Nord-Ouest Val-d'Oise), Pontoise, France ; ^33^AP-HP Hôpital Tenon, Paris, France ; ^34^Centre Hospitalier de Versailles-Hôpital André Mignot, Le Chesnay-Rocquencourt, France ; ^35^Centre Hospitalier François Mitterrand de Pau, Pau, France ; ^36^CHU Rennes, Rennes, France ; ^37^CHU Rouen, Rouen, France ; ^38^CH Saint-Brieuc, Saint-Brieuc, France ; ^39^CH Saint-Nazaire, Saint-Nazaire, France ; ^40^CHRU Strasbourg, Strasbourg, France ; ^41^CHRU Tours, Tours, France ; ^42^Centre Hospitalier Bretagne Atlantique, Vannes, France ; ^43^CH Angoulême, Angoulême, France ; ^44^Centre Hospitalier Jacques Cœur, Bourges, France ; ^45^Centre Hospitalier Intercommunal de Créteil, Créteil, France ; ^46^CH Rodez, Rodez, France

##### **Correspondence:** Juliette Quintin (quintin.juliette@gmail.com)

*Annals of Intensive Care *2013, **13(Suppl 1):**CO-011

**Rationale:** Living with dogs or cats is common. Adverse events in relationship with pets include bites, infections and are increasing. Bacteria most frequently involved are *Pasteurella*, *Bartonella*, *Tularemia*, or *Capnocytophaga*. The goal of the PETSEPSIS study was to report characteristics, clinical symptoms and outcomes of patients requiring intensive care unit (ICU) admission for infection transmitted by cats or dogs.

**Patients and methods/materials and methods:** PETSEPSIS was a retrospective multicenter study conducted in 46 ICUs in France. Consecutive adults admitted to the ICU from January 2009 to December 2019 for sepsis after bites by cats or dogs, or due to *Pasteurella* *spp*, *Bartonella* *spp*, *Tularemia* *spp*, or *Capnocytophaga spp*, were included. Data analysis was descriptive, and univariate and multivariate analysis was performed to identify risks factors of mortality.

**Results:** 174 patients were included; median age was 64.5 years [50.3–73.7] and SAPSII score was 41.5 [28–57.5]. 51.2% of the patients were mechanically ventilated, 53.49% needed cardiovascular drugs, 18.97% renal replacement therapy and 3.6% had limbs amputation. Duration of mechanical ventilation was 7 [4–13] days. The length of stay in ICU was 7 days [4–16]. Hospital mortality was 24.1%. Clinical presentation or severity of sepsis did not vary with bacteria, except for infections due to *Pasteurella spp,* for which patients seem to be older and to have more comorbidities. Contacts with cats were more often reported in *Pasteurella* infections whereas contacts with dogs were more often reported in *Capnocytophaga* and *Bartonella* infections. Risks factors of death were older age, worse SAPSII score, bacteriemia, chronic alcohol abuse and chronic hepatic dysfunction, need for endotracheal ventilation, vasoactive drugs and renal replacement therapy. Initial antimicrobial therapy had no impact on outcome.

**Conclusion:** This is the first and largest multicenterobservational study on severe sepsis transmitted by cats or dogs. Mortality was associated with preexisting disease and severity of organ failure, and not with bacteria identified.

**Compliance with ethics regulations:** Yes in clinical research.

## CO-12 Corticosteroids and mortality in septic shock complicated by the acute respiratory distresss syndrome

### Nicholas Heming^1^, Alain Renault^1^, Emmanuelle Kuperminc^1^, Miguel Carlos^1^, Virginie Maxime^1^, Rania Bounab^1^, Pierre Moine^1^, Djillali Annane^1^

#### ^1^Hôpital Raymond Poincaré, Garches, France

##### **Correspondence:** Nicholas Heming (nicholas.heming@aphp.fr)

*Annals of Intensive Care *2013, **13(Suppl 1):**CO-012

**Rationale:** The role of corticosteroids in the management of non COVID-19 related acute respiratory distress syndrome (ARDS) remains controversial.

**Patients and methods/materials and methods:** The Activated Protein C and Corticosteroids for Human Septic Shock (APROCCHSS) trial was a placebo, controlled trial of the efficacy and safety of the corticosteroids hydrocortisone and fludrocortisone, in adults with septic shock. Patients with ARDS were identified a priori as a subgroup of interest. The primary outcome was 90-day mortality. Key secondary outcomes included mortality at intensive care unit (ICU) discharge, at hospital discharge, at day 28 and day 180 as well as mechanical ventilation free-days within 28 days.

**Results:** Among 1241 APROCCHSS participants, 648 met the criteria for ARDS at the time of inclusion. In ARDS patients, the mean ± SD time between ICU admission and randomization was 2 ± 4 days. There were 155/320 (48.4%) deaths at day 90 in the corticosteroid arm and 186/328 (56.7%) in the placebo arm. The risk ratio (RR) for death at day 90 was 0.85 (95%CI: 0.74 to 0.99; P = 0.04) and 0.91 (95% CI: 0.74 to 1.12; P = 0.36) in patients with and without ARDS, respectively (Breslow-Day homogeneity test P = 0.45). In the ARDS subset, the RR of dying was 0.83 (95%CI: 0.69 to 0.99; P = 0.04) at 28 day, 0.82 (95%CI: 0.70 to 0.97; P = 0.02) at ICU discharge, 0.84 (95%CI: 0.72 to 0.98; P = 0.03) at hospital discharge, and 0.87 (95%CI: 0.76 to 1.00; P = 0.05) at day 180. In the ARDS subset, there was no significant difference regarding ventilator free days 8 ± 10 vs. 7 ± 9 in the corticosteroid vs placebo arm (P = 0.24).

**Conclusion:** The effects of hydrocortisone plus fludrocortisone on 90-day mortality are consistent in septic shock with and without early ARDS.

**Reference 1**

Annane D et al. NEJM 2018.

**Compliance with ethics regulations:** Yes in clinical research.

## CO-13 Impact of patient’s position on esophageal and transpulmonary pressures

### Gaëlle Fouqué^1^, Julien Hagry^1^, Christopher Lai^1^, Rui Shi^1^, Daniela Rosalba^1^, Xavier Monnet^1^, Tai Pham^1^

#### ^1^Hôpital de Bicêtre, APHP, Paris, France

##### **Correspondence:** Tai Pham (tai.pham@aphp.fr)

*Annals of Intensive Care *2013, **13(Suppl 1):**CO-13

**Rationale:** Intubated patients with the acute respiratory distress syndrome (ARDS) usually receive protective ventilation limiting plateau pressure below 30 cmH2O and, if possible, a driving pressure under 15 cmH2O. However, airway pressures might not reflect the actual pressure applied to the lung. Transpulmonary pressure is the difference between airway pressure and pleural pressure, the latter is estimated by the esophageal pressure (1). This technique and measurements had been validated, initially, in healthy patients in half-sitting position, and then confirmed in different positions (2). Few studies have evaluated the impact of patient positioning on pleural pressure and transpulmonary pressure in intubated patients with ARDS. We aimed to assess the changes in esophageal and transpulmonary pressures at different positions (supine at 0° and semi-recumbent at 45°) in intubated patients with ARDS.

**Patients and methods/materials and methods:** Single-center, prospective study performed in a medical ICU of a tertiary care center. All patients diagnosed with ARDS in the study period and monitored with an esophageal catheter were included. We collected end-expiratory and end-inspiratory esophageal pressures, airway pressures and intra-abdominal pressure at the two positions. Inspiratory transpulmonary pressure was calculated using the elastance ratio method. Positive end expiratory pressure (PEEP) was not modified and remained at the level set by the clinician in charge of the patient. Comparisons of values at 0° and 45° were performed using paired Wilcoxon tests.

**Results:** We included 36 patients admitted from January to August 2022. Their median (IQR) age, SOFA score and SAPS II were 63 years (58–72), 9 (5–12) and 52 (33–69) respectively and 77% were men. Twenty-nine of them (80%) were intubated for pneumonia including seven positive to SARS-CoV2 (19%). Their median PaO2/FiO2 on the day of intubation was 96 (72–111) mmHg and 33 (92%) had moderate or severe ARDS. At the time of measurement, set and total PEEP were 12 [10;14] and 13 [11;15] cmH2O respectively. Comparisons of the airway pressures, esophageal pressures and transpulmonary pressures are shown in Table 1. Intra-abdominal pressure also significantly increased from 10 [7;13] to 20 [16;25] cmH2O when changing position from 0° to 45° (p = 0.001).

**Conclusion:** Keeping PEEP stable and changing the patient position from supine at 0° to semi-recumbent at 45° significantly increases all airway pressures and decreases the esophageal pressure. The relative changes led to a non-significant increase in transpulmonary plateau pressure but a significant increase all transpulmonary pressures (end-inspiratory; end-expiratory) and driving pressures.

**Reference 1**

Coudroy R, Chen L, Pham T, Piraino T, Telias I, Brochard L. Acute Respiratory Distress Syndrome: Respiratory Monitoring and Pulmonary Physiology. Semin Respir Crit Care Med. 2019;40(01):066?80.

**Reference 2**

Milic-Emili J, Mead J, Turner JM. Topography of esophageal pressure as a function of posture in man. J Appl Physiol. 1964;19(2):212?6.

**Compliance with ethics regulations:** Yes in clinical research.

**Table 1 (abstract CO-13):**
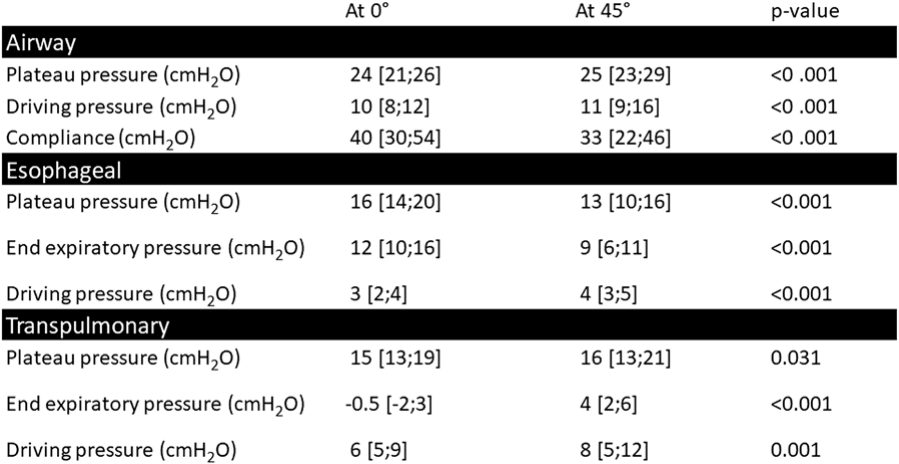
Comparison of pressures at supine at 0° and semi-recumbent 45°

## CO-14 Longitudinal description of complete airway closure in acute respiratory distress syndrome

### Damien Barrau^1^, Yoann Zerbib^1^, Clément Brault^1^, Sami Hraiech^2^, Jack Richecoeur^3^, Michel Slama^1^, Julien Maizel^1^

#### ^1^Chu Amiens, Amiens, France; ^2^Assistance Publique Hôpitaux de Marseille, Marseille, France; ^3^Centre hospitalier de Beauvais, Beauvais, France

##### **Correspondence:** Damien Barrau (djrbarrau@gmail.com)

*Annals of Intensive Care *2013, **13(Suppl 1):**CO-014

**Rationale:** Complete airway closure (CAC) is a frequent phenomenon in ventilated acute respiratory distress syndrome (ARDS) patients. Setting external positive end-expiratory pressure (PEEP) regarding the presence of a CAC and the level of airway opening pressure (AOP) is still debated. The objective of this study was to assess the prevalence of CAC in ventilated-ARDS patients and its evolution over time.

**Patients and methods/materials and methods:** We conducted a bicentric study including all adult patients with moderate or severe ARDS between August 2021 and August 2022. We detected Complete airway closure and measured airway opening pressure using low-flow pressure–volume curve (from PEEP 0 cmH2O) every day, in the absence of patient’s inspiratory effort.

**Results:** Here are preliminary data of 25 patients, corresponding to 89 PV curves. The prevalence of CAC with AOP > 5 cmH2O was 32% (8/25 patients) and 2 patients (6%) developed once during the follow up. The median AOP was 5.3 cmH2O on day 1, 6.2 cmH2O on day 2, and 4.7 cmH2O on day 3. The average weight gain during the follow-up was significantly higher in patients with CAC compared to those without CAC (8.0 [0.5–16.5] kg vs. 1.5 [0.0–9.0] kg, p = 0.019). CAC with an AOP > 5 cmH2O was more common in patients with focal than in non-focal ARDS (50% vs. 6%, p = 0.025).

**Conclusion:** The CAC is a frequent and dynamic phenomenon in ARDS patients. The occurrence of CAC seems to be influenced by hydrolytic balance and chest CT pattern. Repeated measurements seem to be a requisite for personalized lung protective ventilation.

**Compliance with ethics regulations:** Yes in clinical research.

**Figure (abstract CO-14):**
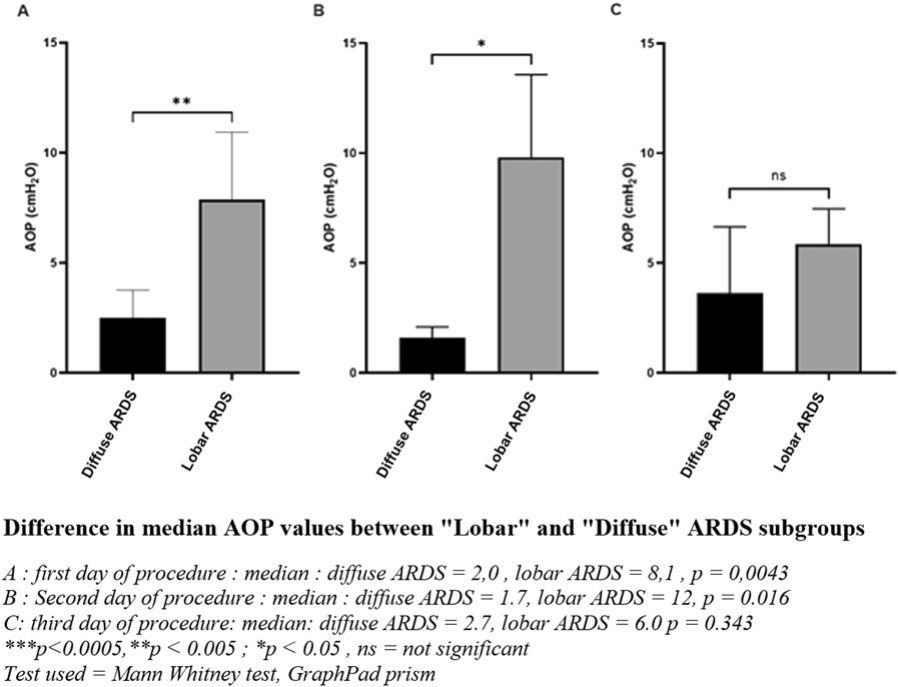
CAC with an AOP > 5 cmH2O was more common in patients with focal than in non-focal ARDS (50% vs. 6%, p = 0.025)

## CO-15 Prone positioning effects on oxygenation and respiratory mechanics according to the R/I ratio in patients with acute respiratory distress syndrome

### Cassilia Dei^1^, Laurent Bitker^2^, Driss Laghlam^3^, Nicolas Bele^4^, Alexandre Robert^5^, Raphaël Devanlay^5^, Giovanni Bousquet^6^, Antoine Goury^7^, Hervé Hyvernat^1^, Clément Saccheri^1^, Jean Dellamonica^1^, Lucas Morand^1^, Mathieu Jozwiak^1^

#### ^1^CHU de Nice, Nice, France; ^2^Hôpital de la Croix Rousse, Hospices Civils de Lyon, Lyon, France; ^3^Assistance Publique-Hôpitaux de Paris, site COCHIN, Paris, France; ^4^Centre hospitalier de la Dracénie, Draguignan, France; ^5^CH de Cannes Simone Veil, Cannes, France; ^6^Hôpitaux Universitaire de Marseille Nord-APHM, Marseille, France; ^7^Hôpital Maison Blanche-CHU de Reims, Reims, France

##### **Correspondence:** Cassilia Dei (cassilia.dei@live.fr)

*Annals of Intensive Care *2013, **13(Suppl 1):**CO-015

**Rationale:** A non-invasive method called "Recruitment to inflation ratio" (R/I ratio), has been proposed to assess the potential of recruitment in patients with acute respiratory distress syndrome (ARDS) and then to titrate and personalize the level of positive end-expiratory pressure (PEEP) to ensure lung recruitment while limiting lung hyperinflation. Prone positioning (PP) is recommended in most severe patients with ARDS. The effects of PP on oxygenation and respiratory mechanics according to the R/I ratio remain to be determined.

**Patients and methods/materials and methods:** This multicenter and prospective study was conducted in 7 French intensive care units and included patients with ARDS requiring prone positioning. The R/I ratio as well as oxygenation parameters and respiratory mechanics were recorded just before and during the 16-h PP session and after supine repositioning. The respiratory rate, the PEEP level and the inspired oxygen fraction (FiO2) remained unchanged during the study period. The median R/I ratio before PP in our cohort was used to classify patients as high and low recruiters.

**Results:** Overall, 41 patients were included: 24 (58%) were men, the median age was 63 (57–70) years old and 32 (78%) had ARDS related to SARS-CoV-2 pneumoniae. The median R/I was 0.50 (0.30–0.84): R/I ratio was 0.24 (0.06–0.35) in low recruiters and 0.83 (0.58–1.00) in high recruiters (p < 0.001). Baseline characteristics, partial arterial pressure of oxygen over FiO2 (PaO2/FiO2) ratio, partial arterial pressure of carbon dioxide (PaCO2) and respiratory mechanics did not significantly differ between high and low recruiters. In high recruiters, PP induced a significant decrease in R/I ratio (p < 0.0001) and increase in PaO2/FiO2 ratio p < 0.0001), while the driving pressure, the compliance of the respiratory system and PaCO2 were unchanged. The PP-induced decrease in R/I ratio persisted after supine repositioning, while the PP-induced increase in PaO2/FiO2 diminished as early as 2 h after supine repositioning but the PaO2/FiO2 ratio did not differ between 2 and 4 h after supine repositioning. In low recruiters, PP induced a significant increase in PaO2/FiO2 ratio (p < 0.0001), while the R/I ratio, the driving pressure, the compliance of the respiratory system and PaCO2 were unchanged. As in high recruiters, the PP-induced increase in PaO2/FiO2 diminished as early as 2 h after supine repositioning but the PaO2/FiO2 ratio did not differ between 2 and 4 h after supine repositioning.

**Conclusion:** The effects of PP on oxygenation and respiratory mechanics appear to differ in high and low recruiters. Inclusions are in progress.

**Compliance with ethics regulations:** Yes in clinical research.

## CO-16 Effects of prone position on lung mechanics in Acute Respiratory Distress Syndrome differ according to the recruitment-to-inflation ratio in supine position. Prospective observational EVALPRO Study

### Christopher Lai^1^, Rui Shi^1^, Ludwig Jelinski^1^, Florian Lardet^1^, Marta Fasan^1^, Hugo Belotti^1^, Nicolas Biard^1^, Laurent Guérin^1^, Nicolas Fage^1^, Quentin Fosse^1^, Thibaut Gobé^1^, Arthur Pavot^1^, Guillaume Roger^1^, Alex Yhuel^1^, Tai Pham^1^, Jean-Louis Teboul^1^, Xavier Monnet^1^

#### ^1^Hôpital Bicêtre, Le Kremlin-Bicêtre, France

##### **Correspondence:** Christopher Lai (christopher.lai@aphp.fr)

*Annals of Intensive Care *2013, **13(Suppl 1):**CO-016

**Rationale:** One of the beneficial respiratory effects of prone position in patients with acute respiratory distress syndrome related to Coronavirus disease 2019 (C-ARDS) could be secondary to improvement in lung mechanics. However, these improvements are inconstant. The objective of the study was to investigate whether an improvement of the compliance of the respiratory system (Crs) and reduction of the driving pressure at the end of a prone position session could be predicted by the potential of lung recruitment at baseline in supine position.

**Patients and methods/materials and methods:** In this prospective, observational, monocentric study in patients who underwent prone position for C-ARDS, from January to May 2021, respiratory variables were assessed just before prone positioning and at the end of the session. Respiratory variables included mechanical ventilation settings and variables of respiratory mechanics, including the recruitment-to-inflation ratio (R/I), an estimate of the potential of lung recruitment compared to lung overinflation.

**Results:** In 41 patients, 156 prone position sessions lasting 18.9 ± 2.7 h, were evaluated. Mortality at day 60 was 49%. Neuromuscular blockade agents were used in 91(58%) sessions. At baseline, tidal volume was 6.1 ± 0.5 mL/kg of predicted body weight, PEEP was 14 ± 3 cmH_2_O and plateau pressure was 29 ± 4 cmH_2_O. The median of the R/I ratio was 0.53 (0.31–0.79), separating low- and high-recruiters. The airway opening pressure was present in 76 (49%) patients and was 6 (5–9) cmH2O in these patients. At the end of the prone position session PaO_2_/FiO_2_ increased from 111 ± 31 mmHg to 165 ± 67 mmHg (p < 0.001), with an increase > 20 mmHg in 96 (62%) sessions. The driving pressure decreased from 14.3 ± 4.4 to 13.7 ± 4.7 cmH_2_O (p = 0.045) and Crs increased from 32 ± 11 to 34 ± 11 (p = 0.037) ml/cmH2O. Whereas the PaO_2_/FiO_2_ improved to the same extent in both low- and high-recruiters, driving pressure and Crs improved only in high-recruiters (from 14 ± 4 to 12 ± 4, p = 0.027, and from 34 ± 11 to 38 ± 13, p = 0.014, respectively).

**Conclusion:** Whereas oxygenation improves to the same extent in low- and high-recruiters with prone position in patients with C-ARDS, driving pressure and Crs improve only in high-recruiter patients. The benefits of prone position could be thus even greater in these patients.

**Compliance with ethics regulations:** Yes in clinical research.

## CO-17 Physiological Effects of Volume Assist Control, Pressure Regulated Volume Control and Airway Pressure Release Ventilation at post-acute phase of acute respiratory distress syndrome

### Mathilde Taillantou-Candau^1^, Alice Vuillermoz^1^, Mathilde Lefranc^1,2^, Elise Yvin^1^, Arnaud Lesimple^1,2^, Antonin Courtais^1^, Dara Chean^1^, Bertrand Pavlovsky^1^, Alain Mercat^1^, Jean-Christophe Richard^1,2^, François Beloncle^1^

#### ^1^Vent’Lab, Medical Intensive Care Unit, University Hospital of Angers, University of Angers, Angers, France; ^2^Med2Lab, Antony, France

##### **Correspondence:** Mathilde Taillantou-Candau (m.taillantou@gmail.com)

*Annals of Intensive Care *2013, **13(Suppl 1):**CO-017

**Rationale:** The optimal ventilation mode after the early phase of acute respiratory distress syndrome (ARDS) when spontaneous ventilation (SV) is resumed is still debated. Volume Assist Control (VAC) is the most frequently used mode, but Pressure regulated volume control (PRVC) has been proposed to limit dyspnea and asynchrony and Airway Pressure Release Ventilation (APRV), a non-synchronized mode, has been suggested to decrease the risk of ventilator induced lung injury (VILI) and improve compliance, gas exchange and respiratory variability. We hypothesized that APRV may promote SV and respiratory variability without altering gas exchange, dyspnea and respiratory effort.

**Patients and methods/materials and methods:** In this prospective, cross-over study, APRV, PRVC and VAC were randomly applied as soon as SV reached 20 to 30% of minute ventilation. For each mode, the ventilator was set to obtain similar positive end-expiratory pressure, tidal volume and respiratory rate. Airway and esophageal pressure and volume distribution in electrical impedance tomography (EIT) were continuously recorded. Gas exchange, hemodynamics, dyspnea (IC-RDOS score), P0.1 and pressure–time product (PTP) were assessed at the end of application of each mode. Tidal volume and PTP variability were assessed by the respective coefficients of variation (standard deviation divided by the mean value).

**Results:** Twenty ARDS patients were included and data were analyzed in 19 patients (one patient discontinued the study because of clinical deterioration). Main ARDS cause was pneumonia in 17 patients (89%). The duration of ventilatory support was 12 (5–19) days before inclusion. Minute ventilation did not differ between the three modes. Sedative drugs were unchanged during the study and RASS (Richmond agitation sedation scale) was similar among the three modes. Gas exchange, hemodynamics, IC-RDOS score, ventilation distribution, P0.1 and PTP did not significantly differ among the three modes. Tidal volume variability was significantly higher in APRV than in VAC and PRVC, PTP variability was significantly higher in APRV than in VAC but did not differ between APRV and PRVC.

**Conclusion:** When SV is resumed after the early phase of ARDS, gas exchange, hemodynamics, dyspnea and respiratory effort did not differ among APRV, VAC and PRVC. Respiratory variability was higher in APRV than in VAC but did not differ between APRV and PRVC.

**Compliance with ethics regulations:** Yes in clinical research.

## CO-18 Acute respiratory distress syndrome in patients with hematological malignancies: a nationwide cohort study

### Pierre-Nicolas Bris^1^, Sami Hraiech^1^, Vanessa Pauly^3^, Veronica Orleans^3^, Laurent Boyer^2^

#### ^1^CHU Nord-Assistance publique des Hôpitaux de Marseille, Marseille, France; ^2^Faculté de médecine Timone, Marseille, France; ^3^CHU Conception-Assistance publique des Hôpitaux de Marseille, Marseille, France

##### **Correspondence:** Pierre-Nicolas Bris (pierrenicolasbris@free.fr)

*Annals of Intensive Care *2013, **13(Suppl 1):**CO-018

**Rationale:** Acute respiratory distress syndrome (ARDS) occurring in patients with hematological malignancies (HM) is a life-threatening condition with specific features. Mortality rate remains high but improvement has been described over the past several years. We aimed to describe the characteristics and outcomes of ARDS in HM patients admitted during one year in French ICUs (Intensive Care Units).

**Patients and methods/materials and methods:** Data for this nationwide cohort study were collected from the French national hospital database (Programme de Médicalisation des Systèmes d’Information (PMSI)). All patients (18 years or older) admitted to French ICUs in 2017 and with an ICD-10 diagnosis of ARDS were included. Diagnosis of HM or solid cancer was detected using an algorithm provided by the Institut National du Cancer (INCa). Three groups were determined and compared according to the presence of an HM, a solid cancer or absence of cancer. Primary endpoint was ARDS mortality (in ICU or at day 90). Secondary endpoints were ICU characteristics (duration of mechanical ventilation, prone positioning, the use of high flow nasal cannula or vasopressors, the use of renal replacement therapy), ARDS characteristics (associated infections) and mortality predictors.

**Results:** A total of 12 846 ARDS patients were included. Among them, 990 had HM and 2744 had a solid cancer. The main malignancies were non-Hodgkin lymphoma (NHL) (28.5%), acute myeloid leukemia (AML) (20.4%) and multiple myeloma (19.7%). Day-90 mortality in patients with HM was higher than patients with no cancer (64.4% vs 46.6% p = 0.0001) but there was no significant difference when comparing to patients with solid cancer (64.4% vs 61.4%, p = 0.091). Intubation rate was lower in patients with HM in comparison with both groups (87.7% vs 90.4% p = 0.017 for patients with solid cancer and 87.7% vs 91.3% p = 0.0002 with no cancer) and SAPSII at admission was higher in this population (49 vs 42.3 p < 0.0001 in solid cancer patients and 49 vs 42.7 p < 0.0001 in no cancer patients). Independent predictors of mortality for patients with HM were a diagnosis of NHL or AML, age, a high SAPS II score, a renal replacement therapy and a septic shock. Bacterial pneumonia and extrapulmonary sepsis causing ARDS were protective.

**Conclusion:** Mortality remains high in patients with HM admitted in ICU with ARDS but patients’ and ARDS characteristics evolved in comparison of older studies.

**Compliance with ethics regulations:** Yes in clinical research.

## CO-19 Relationship between Arterial Oxygen and Mortality Across Critically Ill Patients with Hematologic Malignancies: result from an international collaborative network

### Guillaume Dumas^1,4^, Virginie Lemiale^2^, Idunn Morris^4,8^, Alexandre Demoule^3^, Geeta Mehta^4^, Sean Bagshaw^7^, Tamishta Hensman ^8^, Achille Kouatchet ^6^, Bruno Ferreyro^4^, Djamel Mokart^5^, Elie Azoulay^2^, Laveena Munshi^4^

#### ^1^CHU Grenoble, La Tronche, France; ^2^Hôpital Saint-Louis, APHP, Paris, France; ^3^Hôpital la Pitié Salpêtrière, Paris, France; ^4^Mount Sinaï Hospital, Toronto, Canada; ^5^IPC, Marseille, France; ^6^CHU Angers, Angers, France; ^7^Université d'Alberta, Edmonton, CANADA; ^8^ANZICS, Sidney, Australia

##### **Correspondence:** Guillaume Dumas (GDumasgalant@chu-grenoble.fr)

*Annals of Intensive Care *2013, **13(Suppl 1):**CO-019

**Rationale:** Patients with hematological malignancies are at high risk for life-threatening complications. In this context, most of the studies have focused on the initial oxygenation strategy but little attention has been paid to the impact of hyperoxemia and excess oxygen use in this population. We sought to investigate the impact of partial pressure of arterial oxygen (PaO2) and administered oxygen (FiO2) on day-28 mortality in critically ill patients with hematologic malignancies.

**Patients and methods/materials and methods:** Clinical and oxygenation data from three international cohorts (Europe, Canada, Oceania) of critically-ill patients with hematologic malignancies who required respiratory support (noninvasive ventilation, high-flow nasal cannula, or invasive mechanical ventilation) were obtained. We descriptively evaluated the incidence of normoxemia (paO2 between 60-120 mmHg), hypoxemia (paO2 ≤ 60) and hyperoxemia (paO2 ≥ 120). The association between PaO2 on day 1 and 28 days-mortality was first modeled using a restricted cubic spline. We used mixed-effect Cox models to estimate the effect of hyperoxemia and excess oxygen use (defined as a FiO2 ≥ 0.6 with a PaO2 > 100 mmHg on day 1 of ICU admission) on day-28 mortality.

**Results:** 11,249 patients were included in the analysis. On day 1, 6911 patients (61.4%) had normoxemia, 1454 (12.9%) hypoxemia, and 2884 patients (25.6%) hyperoxemia. The crude day-28 mortality rate was 40.6%. Excess oxygen use was noticed in 2,201 patients (20 After adjustment, there was a significant association between PaO2 and day-28 mortality which demonstrated a U-shape relationship (p < 0.001). We found an independent association between day-1 excess oxygen use and subsequent day-28 mortality (aHR: 1.11[1.03–1.20], p-value = 0.006). On a propensity-matched analysis (n = 4,402 patients), excess oxygen use remained associated with day-28 mortality (HR:1.27[1.16–1.39], p-value < 0.001). In subgroup analysis, higher PaO2 values were associated with poor outcomes in patients admitted with neurological disorders or cardiac arrest contrary to those with sepsis.

**Conclusion:** In critically-ill patients with hematological malignancies, excess oxygen use, and high PaO2 exposure on day 1 were associated with increased mortality. These results suggest a new way to potentially improve cancer patients’ prognosis and should be confirmed in the future.

**Compliance with ethics regulations:** Yes in clinical research.

## CO-20 Prognosis of critically ill allogeneic hematopoietic stem-cell transplantation recipients: data from 1164 patients included in 14 French intensive care units

### Antoine Lafarge^1^, Thibault Dupont^1^, Adrien Joseph^1^, Naike Bigé^2^, Jean-Herlé Raphalen^3^, Julien Mayaux^4^, Djamel Mokart^5^, Anne Renault^6^, Anne-Sophie Moreau^7^, Emmanuel Canet^8^, Laura Platon^9^, Nahema Issa^10^, Muriel Picard^11^, Florent Wallet^12^, Olfa Mghirbi^1^, Frédéric Pène^13^, Elie Azoulay^1^

#### ^1^Hôpital Saint Louis, Paris, France; ^2^Hôpital Saint Antoine, Paris, France; ^3^Hôpital Necker, Paris, France; ^4^Hôpital Pitié Salpêtrière, Paris, France; ^5^Institut Paoli-Calmettes, Marseille, France; ^6^CHU Brest, Brest, France; ^7^CHU Lille, Lille, France; ^8^CHU Nantes, Nantes, France; ^9^CHU Montpellier, Montpellier, France; ^10^CHU Bordeaux, Bordeaux, France; ^11^CHU Toulouse, Toulouse, France; ^12^CHU Lyon, Lyon, France; ^13^Hôpital Cochin, Paris, France

##### **Correspondence:** Antoine Lafarge (antoine.lafarge@aphp.fr)

*Annals of Intensive Care *2013, **13(Suppl 1):**CO-20

**Rationale:** Despite notable changes in the practice of allogeneic hematopoietic stem cell transplantation (Allo-HSCT) in the past years, intensive care unit (ICU) admission is still associated with poor outcome in recipients. Since multicenter longitudinal data on this topic remain scarce, we analyzed reasons for ICU admission, critical care management as well as short- and long-term outcome of critically-ill Allo-HSCT recipients.

**Patients and methods/materials and methods:** The medical records of Allo-HSCT recipients admitted to 14 French ICU during a 6-year period from 1 January 2015 to 31 December 2020 were retrospectively analyzed. Acute graft-versus-host disease (aGVHD) trajectory prior to ICU admission was specifically assessed.

**Results:** Data from 1164 Allo-HSCT recipients admitted to the ICU during the study period were analyzed. Median age at ICU admission was 56.4 [42.7–63.6] years, 61.4% were male. Median time from Allo-HSCT to ICU admission was 84 [15–369] days and 463 (39.8%) patients were transferred to the ICU during the engraftment period. Respiratory dysfunction (461 patients, 39.6%) and shock (228 patients, 19.6%) were the main reasons for ICU admission. Seventy-nine (6.8%) patients were admitted for acute kidney injury. Median SOFA at ICU admission was 6.0 [4.0–8.0]. Six hundred and eight (52.2%) patients displayed ≥ 1 infection(s) during the ICU stay, including opportunistic infections in 228 (37.5%) patients. In the whole cohort, 221 (19.0%) patients underwent renal replacement therapy, 478 (41.1%) received vasopressors and 440 (37.8%) required mechanical ventilation. Eight hundred and sixty-two (74.1%) patients could be discharged alive from the ICU and 590 (57.4%) from the hospital. Day-90, 1-year and 3-year survival rates were 54.1%, 41.3% and 33.8% respectively (Figure 1). Age (hazard ratio (HR) 1.02 [1.01–1.03]; P < 0.001), glucocorticoid-refractory GVHD (HR 1.46 [1.22–1.75]; P < 0.001), male sex (HR 1.50 [1.14–1.98]; P < 0.001), glucocorticoids posology (HR 1.61 [1.18–2.18]; P < 0.001), vasopressors (HR 1.70 [1.27–2.29]; P < 0.001), mechanical ventilation (HR 2.67 [1.95–3.64]; P < 0.001) and renal replacement therapy (HR 3.66 [2.48–5.42]; P < 0.001) were independent predictive factors of day-90 mortality.

**Conclusion:** In Allo-HSCT recipients, ICU admission remains associated with a dismal prognosis. Beyond host factors, glucocorticoid-refractory aGVHD and organ dysfunctions requiring live sustaining therapies are the major determinants of death. Further analyses are warranted to stratify Allo-HSCT recipients based on a high risk of mortality and identify patients for whom critical care management may be aggressive but nonbeneficial.

**Compliance with ethics regulations:** Yes in clinical research.

**Figure (abstract CO-20):**
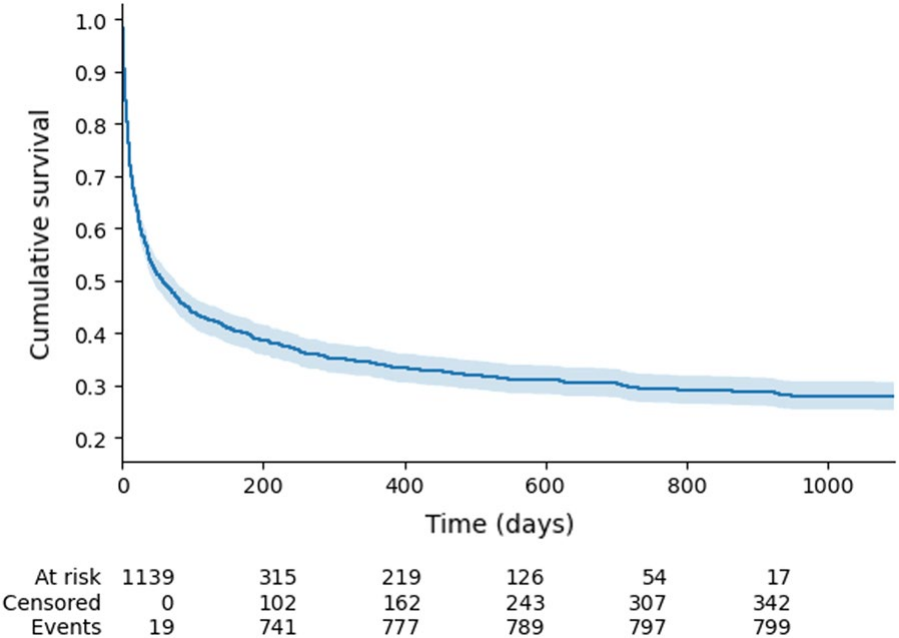
Whole cohort survival analysis

## CO-21 Cardiogenic shock in patients with malignancies: a multicenter retrospective study: the CARTOON Study

### Mickael Lescroart^1^, Helene Kemp^1^, Olivier Imauven^2^, Jean Herlé Raphalen^2^, Laurent Argaud^3^, Hamid Merdji^4^, Guillaume Morel^5^, Emmanuel Canet^6^, Julien Schmidt^7^, Guillaume Lacave^8^, Francois Bagate^11^, Guillaume Louis^9^, Bruno Mourvillier^12^, Fabienne Tamion^14^, Maxens Decavele^13^, Florent Wallet^15^, Hadrien Winiszewski^16^, Nahema Issa^17^, Anne-Sophie Moreau^18^, Natacha Maquigneau^19^, Audrey De Jong^20^, Alexandre Lautrette^21^, Elia Azoulay^1^, Guillaume Dumas^1^, Lara Zafrani^1^

#### ^1^Hôpital Saint Louis, Paris, France; ^2^Adult Intensive Care Unit, Necker-Enfants-Malades University Hospital, Assistance Publique-Hôpitaux de Paris (AP-HP), Paris, France; ^3^Hospices Civils de Lyon, Hôpital Edouard Herriot, Service de Médecine Intensive-Réanimation, 5, Place d'Arsonval, Lyon, France; ^4^Hôpitaux Universitaires de Strasbourg, Service de Médecine Intensive Réanimation, Nouvel Hôpital Civil, 1, place de l'Hôpital, Strasbourg, France; ^5^Service d'hématologie et d'oncologie, Hôpitaux universitaires de Strasbourg, Strasbourg, France; ^6^Medecine Intensive Réanimation, University Hospital Center, Nantes, France; ^7^Unité de médecine intensive et réanimation, Assistance Publique-Hôpitaux de Paris, Avicenne Hospital, Groupe Hospitalier Paris Seine Saint-Denis, Bobigny, France; ^8^Medico-Surgical Intensive Care Department, Centre Hospitalier de Versailles, Site André Mignot, Le Chesnay, Versailles, France; ^9^Médecine Intensive et réanimation, Centre hospitalier Régional Metz-Thionville, Mercy Hospital, 1 allée du château, Metz, France; ^10^Service de Réanimation Médicale, Normandie Univ, UNIROUEN, U1096, CHU de Rouen, Rouen, France; ^11^Service de Médecine Intensive Réanimation, AP-HP, CHU Henri Mondor, DHU A-TVB, 51, avenue du Mal de Lattre de Tassigny, Créteil, France; ^12^Centre Hospitalo-Universitaire de Reims (CHU), Hôpital Robert-Debré, Service de Réanimation médicale, Reims, France; ^13^APHP, Groupe Hospitalier Universitaire APHP-Sorbonne Université, site Pitié-Salpêtrière, Service Médecine Intensive et Réanimation (Département R3S), Paris, France; ^14^Service de Réanimation Médicale, Normandie Univ, UNIROUEN, U1096, CHU de Rouen, Rouen, France; ^15^Médecine Intensive et Réanimation, Hôpital Lyon Sud, Pierre-Bénite, Lyon, France; ^16^Médecine Intensive et Réanimation, Centre Hospitalier Universitaire de Besançon, Besançon, France; ^17^Réanimation médicale, groupe hospitalier Saint-André, 1, rue Jean-Burguet, 33075 Bordeaux cedex, France; Médecine interne et maladies infectieuses, groupe hospitalier Saint-André, 1, rue Jean-Burguet, Bordeaux, France; ^18^CHU de Lille, hôpital Salengro, service de médecine intensive réanimation, rue Emile-Laine, Lille, France; ^19^Service Medico-Chirurgical, Unité de soins intensifs, Centre Hospitalier de La Roche-sur-Yon, La Roche Sur Yon, France; ^20^Médecine intensive et réanimation, Département d’Anesthésie et soins critiques Hôpital Saint-Éloi, CHU de Montpellier, INSERM U1046, 80, Avenue Augustin Fliche, Montpellier, France; ^21^Medical Intensive Care Unit, Gabriel-Montpied University Hospital, Clermont-Ferrand, France

##### **Correspondence:** Mickael Lescroart (dr.lescroart@gmail.com)

*Annals of Intensive Care *2013, **13(Suppl 1):**CO-21

**Rationale:** Despite significant advancements in oncology/hematology over the past decades, cancer patients (CP) continue to face a significant cardiovascular (CV) burden due to both their malignancy and the toxicity of anti-cancer therapy. No data is currently available regarding the characteristics and outcomes of cancer patients (CP) admitted in intensive care unit (ICU) for cardiogenic shock (CS). We aimed to provide a contemporary cohort of cardiogenic shock in CP and determine risk factors associated with ICU mortality.

**Patients and methods/materials and methods:** 214 oncological patients with CS from 21 French ICUs were included between 2010 and 2022. Clinical data were extracted from medical records. The primary outcome was overall ICU survival. Secondary outcomes were the need for renal replacement therapy (RRT), mechanical ventilation, and ICU length of stay. Risk factors for ICU mortality were determined through logistic regression.

**Results:** Hemopathy (68.7%) was the most represented type of malignancy. CV risk factors were prevalent as one third of patients had a history of smoking or hypertension. The overall ICU mortality was 50% with a 37-days median survival. One hundred sixty-eight patients (78.5%) required vasopressor therapy, 140 (65.7%) mechanical ventilation and 52 (24.9%) renal replacement therapy. Independent risk factors for ICU mortality were: mechanical ventilation (OR 2.55 [1.16–5.72], p = 0.02), vasopressor therapy (OR 3.36 [1.15–11.31], p = 0.03), home medication with renin–angiotensin–aldosterone system inhibitors (OR 2.84 [1.06–8.17], p = 0.04), and the inotropic drug (OR for dobutamine, versus epinephrine: 0.27 [0.08–0.78], p = 0.02). Thirteen patients (6.0%) required VA-ECMO with similar survival rates (n = 7/13, p = 0.95). Among patients alive at discharge, the median survival was 418 days.

**Conclusion:** The prognosis of cancer patients admitted in ICU for cardiogenic shock remains poor with a high mortality rate and frequent need for invasive organ support. The population that could benefit from intensive care has yet to be determined.

**Compliance with ethics regulations:** Yes in clinical research.

## CO-22 Do renin-angiotensin system blockers impact on the prognosis of critically ill cancer patients?

### Driss Laghlam^1^, Anis Chaba^1^, Tarneaud Matthias^1^, Julien Charpentier^1^, Jean-Paul Mira^1^, Frédéric Pène^1^, Clara Vigneron^1^

#### ^1^Hôpital Cochin, Paris, France

##### **Correspondence:** Clara Vigneron (claravigneron@hotmail.fr)

*Annals of Intensive Care *2013, **13(Suppl 1):**CO-22

**Rationale:** Increasing evidence argue for promotion of tumorigenesis through activation of renin-angiotensin system pathway. Accordingly, a benefit of renin-angiotensin system blockers (RABs) treatments has been suggested in patients with solid cancers in terms of survival. Our study aims to evaluate in-ICU survival and one-year survival in cancer patients admitted to the ICU with respect to the use of RABs.

**Patients and methods/materials and methods:** We conducted a retrospective observational single-center study in a 24-bed medical ICU. Consecutive adult patients (age ≥ 18 years) with a diagnosis of solid tumour (previously known or diagnosed during the ICU stay) requiring unplanned ICU admission were included.

**Results:** From 2007 to 2020, 1845 patients with solid malignancies were admitted (median age 67 years [59–75], males 61,7%). The most frequent primary tumor sites were the gastrointestinal tract (26.8%), the lung (24.7%), the urological tract (20.1%), and gynecologic and breast cancers (13.9%). RABs were used in 414 patients, distributed into 220 (53.1%) with angiotensin-receptor blockers (ARBs) and 194 (46.9%) with angiotensin-converting enzyme inhibitors (ACEi). After adjustment in a multivariate model, ARBs use (OR = 0.62 (0.40–0.92), p = 0.03) and ACEi use (OR = 0.52, 95%CI (0.32–0.82), p = 0.006) were both associated with improved in-ICU survival (Figure 1). Treatment with ARBs was independently associated with decreased one-year mortality (OR = 0.6 95% CI (0.4–0.9), p = 0.02), whereas treatment with ACEi was not (Figure 1).

**Conclusion:** The use of ARBs, but not of ACEi, is associated with improved one-year survival in patients with solid malignancies admitted to the ICU.

**Compliance with ethics regulations:** Yes in clinical research.Whole cohort survival analysis

**Figure 1 (abstract CO-22):**
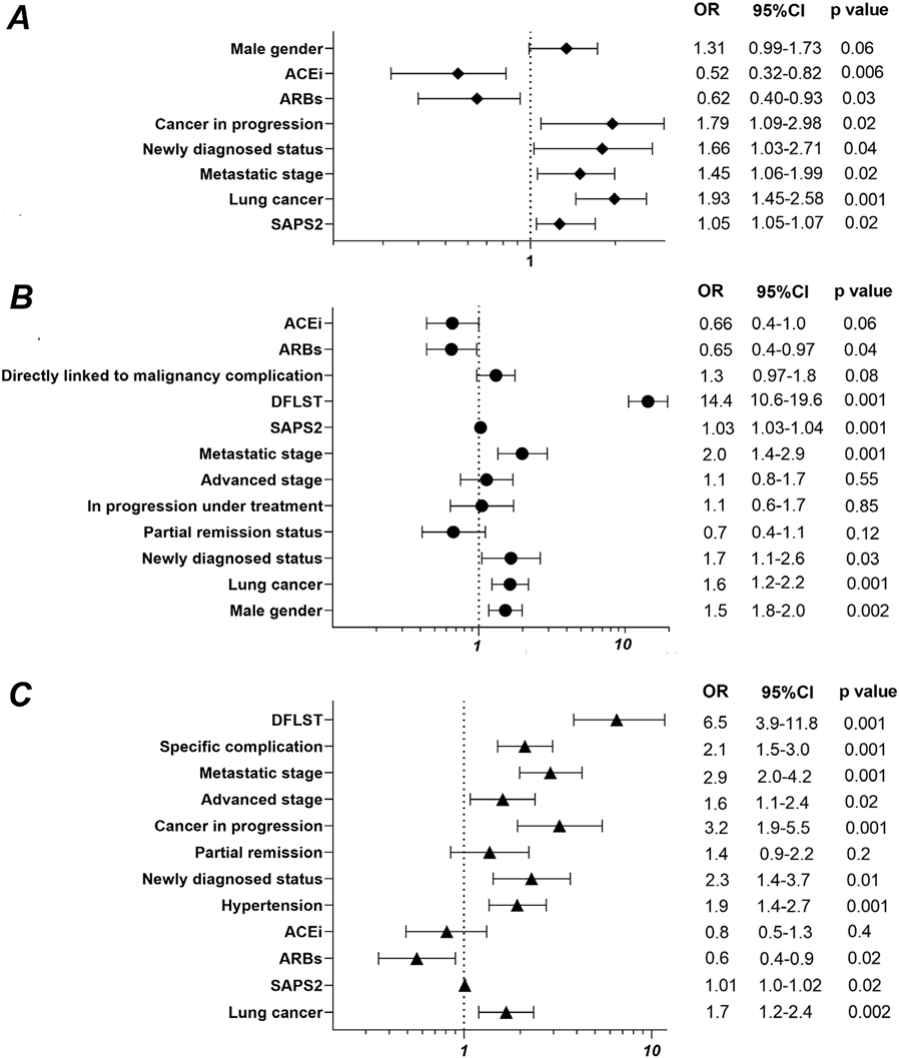
Independent determinants of in-ICU, in-hospital and one-year mortality Panel A. Independent risk factors for in-ICU mortality. Panel B. Independent risk factors for in-hospital mortality. Panel C. Independent risk factors for one-year mortality

## CO-23 ICU-acquired major bleeding events in critically ill patients with haematological malignancies

### Clément Devautour^1^, Clara Vigneron^1^, Julien Charpentier^1^, Sandrine Valade^2^, Matthieu Jamme^3^, Frédéric Pene^1^

#### ^1^Hôpital Cochin, APHP, Paris, France; ^2^Hôpital Saint Louis, APHP, Paris, France; ^3^Hôpital Privé de l’ouest Parisien, Ramsay Générale de Santé, Paris, France

##### **Correspondence:** Clément Devautour (cdevautour@gmail.com)

*Annals of Intensive Care *2013, **13(Suppl 1):**CO-23

**Rationale:** Bleeding events are common complications in critically ill patients with haematological malignancies. We aimed to describe acquired major bleeding events during their ICU stay.

**Patients and methods/materials and methods:** We conducted an observational monocenter retrospective study including all adult patients with a history of haematological malignancy requiring unplanned ICU admission over a 12-year period (2007–2018). Non-inclusion criteria were the following: admission to secure a procedure, planned admissions following elective surgery and patients with haematological malignancies cured for more than 5 years. Endpoints were major bleeding events (WHO classification grade 3–4) acquired during their ICU stay.

**Results:** A total of 1012 patients (age = 65 [55–76] years) were included. The main haematological malignancies were non-Hodgkin lymphoma (n = 339, 33.5%), acute myeloid leukemia (n = 183, 18.1%), and multiple myeloma (n = 171, 16.9%). Most patients were recently diagnosed (n = 339, 33.5%) or in progression under treatment (n = 262, 25.9%). Five hundred ninety-six (58.9%) patients were under treatment. The leading causes of admission were circulatory (n = 347, 34.2%) and acute respiratory failures (n = 287, 28.4%). Among them, 109 (10.8%) presented at least one episode of acquired major bleeding event in the ICU, mainly gastrointestinal (n = 45, 41.3%), airway (n = 13, 11.9%) and vascular catheter (n = 13, 11.9%) bleeding events. Compared to patients without haemorrhagic event, they were more likely to have been admitted to the ICU for acute kidney injury (56.9% vs. 38.1% vs p < 0.001) and haemorrhagic event (28.4% vs 2.5%, p < 0.001). Biological exams performed at ICU admission showed multiple alterations in coagulation measurements including decreased platelet count (54 vs 80 G/L, p = 0.002), prolonged prothrombin time (62.0 vs 72.5%, p < 0.001) and increased activated partial thromboplastin ratio (1.25 vs 1.18, p = 0.002), acute kidney dysfunction as assessed by increased urea (10.6 vs 8.7 mmol/L, p = 0.011) and creatinine levels (129.0 vs 102.5 µmol/L, p = 0.007), lower albumin level (23.0 vs 28.0 g/L, p = 0.026) and increased bilirubin level (17.0 vs 12.0 µmol/L, p < 0.001). They required more frequently invasive mechanical ventilation (85.3% vs 43.4%, p < 0.001), inotropes/vasopressors (67.9% vs 32.6%, p < 0.001) or renal replacement therapy (46.8% vs 21.5%, p < 0.001) during their ICU stay. Accordingly, they exhibited poorer prognosis: ICU, hospital and one-year mortality rates were 55.0% vs 18.0% (p < 0.001), 59.6% vs 23.7% (p < 0.001) and 80.4% vs 54.7% (p < 0.001), respectively.

**Conclusion:** In patients with haematological malignancies, ICU-acquired major bleeding events are associated with acute kidney injury and recent history of bleeding event. Our study revealed biological disturbances associated with those events. Patients developing major bleeding events exhibit a dismal short and long-term prognosis.

**Compliance with ethics regulations:** Yes in clinical research.

## CO-24 Electroencephalography for prognostication of outcome in adults with severe herpes simplex encephalitis

### Lina Jeantin^1^, Claire Dupuis^2^, Geoffroy Vellieux^3^, Pierre Jaquet^4^, Etienne De Montmollin^5^, Jean-François Timsit^5^, Romain Sonneville^5^

#### ^1^GHU Psychiatrie et neurosciences, Paris, France; ^2^Clermont-Ferrand University Hospital, Clermont-Ferrand, France; ^3^Pitié-Salpêtrière University Hospital, Paris, France ; ^4^Delafontaine Hospital, Saint Denis, France; ^5^Bichat-Claude Bernard University Hospital, Paris, France

##### **Correspondence:** Lina Jeantin (lina.jeantin@aphp.fr)

*Annals of Intensive Care *2013, **13(Suppl 1):**CO-24

**Rationale:** Electroencephalography (EEG) is recommended for the practical approach to the diagnosis and prognosis of encephalitis. We aimed to investigate the prognostic value of standard EEG (_std_EEG) in adult patients with severe herpes simplex encephalitis (HSE).

**Patients and methods/materials and methods:** We performed a retrospective analysis of consecutive ICU patients with severe HSE in 38 French centers between 2006 and 2016. Patients with at least one _std_EEG study performed at ICU admission were included. _std_EEG findings were reviewed independently by two investigators. The association between _std_EEG findings (i.e., background activity, lateralized periodic discharges, seizures/status epilepticus, and reactivity to painful/auditory stimuli) and poor functional outcome, defined by a score on the modified Rankin Scale (mRS) of 3 to 6 (moderate to severe disability or death) at 90 days, were investigated.

**Results:** We included 214 patients with at least one available _std_EEG study. The first stdEEG was performed after a median time of one (IQR 0 to 2) day from ICU admission. At the time of recording, 138 (64.5%) patients were under invasive mechanical ventilation. Lateralized periodic discharges were recorded in 91 (42.5%) patients, seizures in 21 (9.8%) and status epilepticus in 16 (7.5%). In the whole population, reactivity to auditory/noxious stimuli was tested in 140/214 (65.4%) patients and was absent in 71/140 (33.2%) cases. In mechanically ventilated patients, _std_EEG reactivity was tested in 91/138 (65.9%) subjects, and was absent in 53/91 (58.2%) cases. Absence of reactivity was the only independent _std_EEG finding associated with poor functional outcome in the whole population (OR 2.80, 95%CI 1.19 to 6.58) and in the subgroup of mechanically ventilated patients (OR 4.99, 95%CI 1.6 to 15.59). adjusted analyses for common clinical predictors of outcome and sedation at time of _std_EEG revealed similar findings in the whole population (OR 2.03, 95% CI 1.18 to 3.49) and in mechanically ventilated patients (OR 2.62, 95%CI 1.25 to 5.50).

**Discussion:** The lack of EEG reactivity to external stimuli in critically ill patients with HSE is associated with poor outcome, independently from the presence of sedation and common clinical prognostic factors in this population (including age, coma, and fever at admission). To date, little data is available about the prognostic value of _std_EEG and our study assembled one of the largest cohorts of patients with HSE requiring ICU admission to assess the value of _std_EEG markers for functional outcome.

**Conclusion:** Absence of _std_EEG reactivity to auditory/noxious stimuli is an independent marker of poor functional outcome in severe herpes simplex encephalitis.

**Reference 1**

ENCEPHALITICA study group, Jaquet P, de Montmollin E, Dupuis C, Sazio C, Conrad M, et al. Functional outcomes in adult patients with herpes simplex encephalitis admitted to the ICU: a multicenter cohort study. Intensive Care Med. 2019.

**Reference 2**

Azabou E, Navarro V, Kubis N, Gavaret M, Heming N, Cariou A, et al. Value and mechanisms of EEG reactivity in the prognosis of patients with impaired consciousness: a systematic review. Crit Care. 2018.

**Compliance with ethics regulations:** Yes in clinical research.

**Table 1 (abstract CO-24):**
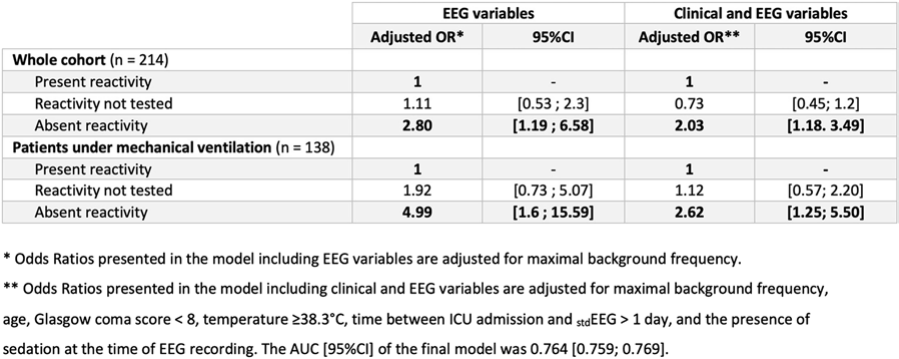
Association of stdEEG reactivity with poor functional outcome, multivariable analyses

## CO-25 Neurological complications in adult patients with pneumococcal meningitis in the intensive care unit: a french retrospective multicenter cohort study

### Camille Legouy^1^, Renaud Cornic ^2^, Keyvan Razazi ^9^, Damien Contou ^4^, Stéphane Legriel ^5^, Eve Garrigues ^8^, Pauline Buiche ^7^, Maxence Decavele ^6^, Sarah Benghanem ^11^, Thomas Rambaud ^10^, Marina Esposito-Farèse^2^, Jean-François Timsit^2^, Camille Couffignal^2^, Romain Sonneville^2^

#### ^1^GHU Paris Psychiatrie & Neurosciences, Paris, France; ^2^Hôpital Bichat, Paris, France; ^3^Hôpital Delafontaine, Saint-Denis, France; ^4^Hôpital d'Argenteuil, Argenteuil, France; ^5^Hôpital de Versailles, Versailles, France; ^6^Hôpital Pitié-Salpêtrière, Paris, France; ^7^Hôpital Saint-Antoine, Paris, France; ^8^Hôpital Ambroise Paré, Boulogne-Billancourt, France; ^9^Hôpital Mondor, Créteil, France; ^10^Hôpital Avicenne, Bobigny, France; ^11^Hôpital Cochin, Paris, France

##### **Correspondence:** Camille Legouy (c.legouy@ghu-paris.fr)

*Annals of Intensive Care *2013, **13(Suppl 1):**CO-25

**Rationale:** Pneumococcal meningitis is life-threatening infection with high morbidity and mortality rates, likely resulting from the occurrence of neurological complications, such as brain oedema, ischemic stroke, and intracranial hemorrhage. To investigate the association of neurological complications diagnosed on admission neuroimaging with functional outcomes of adults with severe pneumococcal meningitis, requiring care in the intensive care unit (ICU).

**Patients and methods/materials and methods:** We performed a retrospective multicenter study in 11 centers in France, between February 2005 and September 2021. We included consecutive adults with pneumococcal meningitis who required at least 48 h of ICU stay and who underwent neuroimaging assessment. Neurological complications were defined as 1) ischemic stroke, 2) intracranial hemorrhage (i.e. intracerebral hematoma, subdural hematoma, or subarachnoid hemorrhage), 3) abscess/empyema, 4) ventriculitis, 5) thrombophlebitis, 6) hydrocephalus, and 7) cerebral oedema on neuroimaging at admission. Exposure: Neuroimaging at admission. Main outcome and measures: AUnfavorable neurological outcome at 90 days after ICU admission, defined by the modified Rankin Scale (mRS) > 2, indicating moderate-to-severe disability or death.

**Results:** Among the 237 patients included, 103 (44%, 95%CI 37–50%) had an unfavorable outcome at 90 days, including 71 (30%, 95%CI 25–37%) deaths. Neurological complications at admission occurred in 68 of the 237 patients (31%) and in 70 patients (64%) during ICU stay with a majority of ischemic stroke. Using a multivariate logistic model at ICU admission, we identified four predictive markers independently associated with unfavorable outcome: chronic alcohol consumption (adjusted Odds Ratio (aOR 2.37, 95%CI 1.09–5.26), focal neurological sign(s) (aOR 2.11 95%CI 1.06–4.24), neurological complications on ICU admission (aOR 3.65, 95%CI 1.83–7.47) and cerebrospinal fluid (CSF) leukocyte count < 1000 cell/microL, (aOR 4.17, 95%CI 2.22–8.34). Competing risk analysis with death as competing risk and showed that chronic alcohol consumption was the only significant marker for the risk of persistent disability (mRS > 2) at 90 days (Cause specific Hazard Ratio 4.26, 95%CI 1.83–9.91).

**Conclusion:** In adult patients with severe pneumococcal meningitis, the identification of neurological complications on admission neuroimaging is an independent predictor of unfavorable neurological outcome. Chronic alcohol consumption is the only predictive risk of persistent disability at three months in survivors.

**Compliance with ethics regulations:** Yes in clinical research.

**Table 1 (abstract CO-25):**
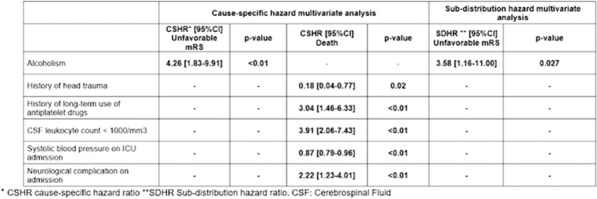
Competing risk analyses-adjusted Hazard Ratios and their 95%CIs from the Cause-Specific Hazard model and the Fine-Gray Subdistribution Hazard models

## CO-26 Prognostic value of EEG in severe stroke patients requiring mechanical ventilation: a pre-planned ancillary study of the SPICE prospective multicenter study

### Sarah Benghanem^1,2,3^, Etienne Gayat^4^, Nathalie Kubis^5^, Ambre Loiodice^6^, Martine Gavaret^7^, Tarek Sharshar^8^, Arnaud Foucrier^9^, Charles Gregoire^10^, David Cortier^11^, Samy Figueiredo^12^, Viviane Bouilleret^13^, Pierre Jacquet^14^, François Bagate^15^, Jean Pascal Lefaucheur^16^, Bertrand Guidet^17^, Emmanuelle Appartis^18^, Bruno Megarbane^19^, Lionel Naccache^20^, Alain Cariou^1,2^, Isabelle Crassard^21^, France Woimant^21^, Mickael Mazighi^22^, Romain Sonneville^23^

#### ^1^APHP.Centre, Hôpital Cochin, Paris, France; ^2^University Paris Cité, Medical School, Paris, France; ^3^INSERM UMR 1266, FHU NeuroVasc, Institut de Psychiatrie et Neurosciences de Paris-IPNP, Paris, France; ^4^APHP.Nord, Department of Anesthesiology and Critical Care, DMU Parabol, Paris, FRANCE; ^5^APHP.Nord, Neurophysiology department, Paris, France; ^6^Department of Biostatistics, ICUREsearch, Paris, France; ^7^Neurophysiology and Epileptology department, GHU Psychiatry & Neurosciences, Sainte Anne, Paris, France; ^8^Department of Neuroanesthesiology and Intensive Care, Saint Anne Hospital, Paris, France; ^9^APHP, Department of Anesthesiology and Critical Care, Beaujon University Hospital, Clichy, France; ^10^Department of Intensive Care, Rothschild Hospital Foundation, Paris, France; ^11^Department of Intensive Care, Foch Hospital, Suresnes, France; ^12^APHP, Department of Anesthesiology and Critical Care, Bicêtre University Hospitals, Kremlin Bicêtre, France; ^13^Neurophysiology and Epileptology department, Bicêtre University Hospitals, Le Kremlin Bicêtre, France; ^14^Department of Intensive Care Medicine, Delafontaine Hospital, Saint Denis, France; ^15^APHP, Department of Intensive Care Medicine, Henri Mondor University Hospital, Créteil, France; ^16^Neurophysiology department, Henri Mondor University Hospital, Créteil, France; ^17^APHP, Department of Intensive Care Medicine, Saint Antoine University Hospital, Paris, France; ^18^Neurophysiology department, Saint Antoine University Hospital, Paris, France; ^19^APHP, Department of Medical and Toxicological Critical Care, Lariboisière Hospital, Paris, France; ^20^Neurophysiology department, Pitié-Salpêtrière university hospital, Paris, France; ^21^Agence régionale de Santé Ile-de-France, Paris, France; ^22^APHP Nord, Department of Neurology, Lariboisière University Hospital, Paris, France; ^23^APHP Nord, Department of Intensive Care Medicine, Bichat-Claude Bernard University Hospital, Paris, France

##### **Correspondence:** Sarah Benghanem (sarah.benghanem@aphp.fr)

*Annals of Intensive Care *2013, **13(Suppl 1):**CO-26

**Rationale:** We aimed to investigate the prevalence of early EEG abnormalities in adult stroke patients requiring mechanical ventilation and their predictive value for functional outcome.

**Patients and methods/materials and methods:** We conducted a pre-planned ancillary study of the prospective multicenter SPICE cohort study (ClinicalTrials.gov Identifier: NCT03335995, study protocol PMID: 32026446). We included patients from the SPICE cohort with at least one EEG study performed during intensive care unit (ICU) stay. The primary endpoint was poor functional outcome at one year, defined by a score of 4–6 (severe disability or death) on the modified Rankin scale (mRS).

**Results:** Among the 364 patients prospectively enrolled in SPICE, 153 patients (49 ischemic strokes, 52 intracranial hemorrhages, and 52 subarachnoid hemorrhages) had at least one EEG performed, after a median time of 4 (IQR, 2–7)) days. Diffuse slowing (70% vs 63%, p = 0.37), focal slowing (38% vs 32%, p = 0.15), periodic discharge (2.3% vs 3.7%, p = 0.9), and electrographic seizure (4.5% vs 3.7%, p = 0.4) rates were comparable between poor outcome and good outcome patients. After adjustment for clinical confounders (i.e. age, stroke subtype, non-neurological organ failure, Glasgow coma score), an unreactive EEG background to noise (OR 4.8, 95%CI (1.84–12.53)) or pain (OR 4.76, 95%CI (1.89–11.96)) remained independently associated with poor functional outcome. Conversely, a benign EEG (defined as continuous and reactive background, without seizure, periodic discharges, and burst suppression) was protective (OR 0.29, 95%CI (0.12–0.73)).

**Conclusion:** Unreactive and reactive EEG predicts respectively poor and good one-year functional outcomes in mechanically ventilated stroke patients.

**Compliance with ethics regulations:** Yes in clinical research.

**Figure 1 (abstract CO-26):**
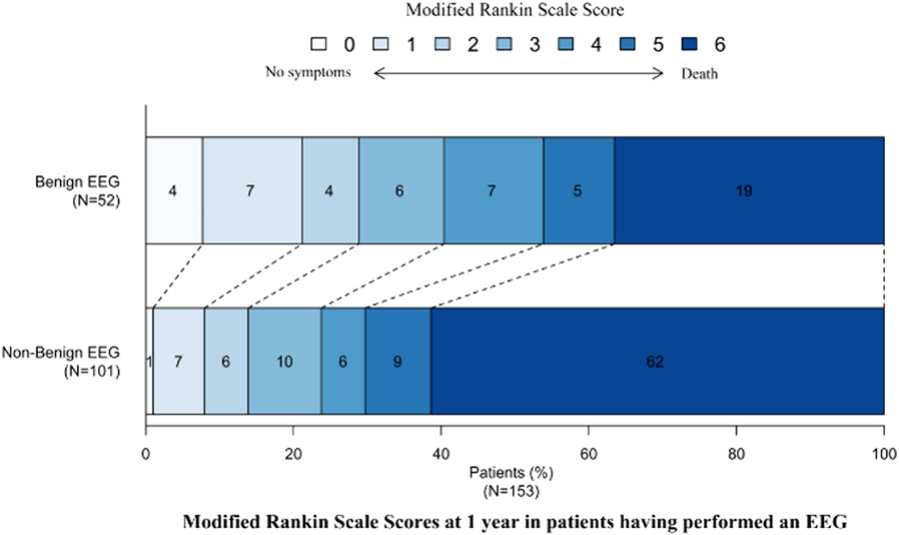
Modified Rankin Scale Scores at 1 year in patients having performed an EEG

## CO-27 Aberrant brain–heart coupling is associated with the severity and prognosis of hypoxic-ischemic brain injury after cardiac arrest

### Bertrand Hermann^1^, Diego Candia-Rivera^2^, Tarek Sharshar^3^, Martine Gavaret^3^, Jean-Luc Diehl^1^, Alain Cariou^4^, Sarah Benghanem^4^

#### ^1^HEGP, AP-HP. Centre-Université Paris Cité, Paris, France; ^2^Institut du Cerveau et de la Moelle épinière-ICM, INSERM U1127, CNRS UMR 7225, F-75013, Paris, France, Paris, France; ^3^GHU Paris Psychiatrie Neurosciences, Paris, France, Paris, France; ^4^Cochin, AP-HP Centre-Université de Paris, Paris, FRANCE

##### **Correspondence:** Bertrand Hermann (bertrand.hermann@aphp.fr)

*Annals of Intensive Care *2013, **13(Suppl 1):**CO-27

**Rationale:** Approximately 50% of post-cardiac arrest survivors remain comatose after 72 h, a substantial proportion of which will have a poor neurological outcome, predominantly due to irreversible hypoxic-ischemic brain injury. Recent findings in healthy subjects and patients suggested that autonomic nervous system activity measured by brain–heart interactions could be reliable markers of consciousness and cognitive processing. Thus, we hypothesized that brain–heart interactions are associated with the severity of hypoxic-ischemic brain injury and the prognosis of these patients.

**Patients and methods/materials and methods:** In post-cardiac arrest patients still comatose 48 h after sedation weaning, brain–heart interaction markers were computed on 5 min of continuous EEG/ECG recording using a synthetic data generation model, gathering bidirectional interactions between EEG frequency bands (delta, theta and alpha) and heart-rate variability frequency bands (low and high frequency—LF and HF). The strength and complexity of the interactions were quantified using medians and refined composite multiscale entropy (RCMSE). Primary outcome was the severity of brain injury, assessed by: (i) standardized qualitative EEG classification, (ii) somatosensory evoked potentials (N20), and (iii) neuron-specific enolase levels. Secondary outcome was the 3-month neurological status, assessed by the Cerebral Performance Category score [good (1–2) vs. poor outcome (3–4-5)].

**Results:** Between January 2007 and July 2021, 181 patients [116 males (64%), median age 61 years, age range 49–72 years] were admitted to ICU for a resuscitated cardiac arrest (76% out-of-hospital, 69% non-shockable rhythm). Poor neurological outcome was observed in 134 patients (74%). Qualitative EEG patterns suggesting high severity were associated with a decreased sympatho-vagal balance. Severity of EEG changes were proportional to higher absolute values of brain-to-heart coupling strength (p < 2 × 10–3 for all brain-to-heart frequencies) and lower values of complexity (all p-values < 0.05 except for alpha-to-low frequency). Brain-to-heart coupling strength was significantly higher in patients with bilateral absent N20 and correlated with neuron-specific enolase levels at day 3. This aberrant brain-to-heart coupling (increased strength, decreased complexity) was also associated with 3-month poor neurological outcome (Table).

**Conclusion:** Our results suggest that autonomic dysfunctions may well represent hypoxic-ischemic brain injury post-cardiac arrest pathophysiology. These results open avenues for integrative monitoring of autonomic functioning in critical care patients with potential prognostic applications.

**Compliance with ethics regulations:** Yes in clinical research.

**Table 1 (abstract CO-27):**
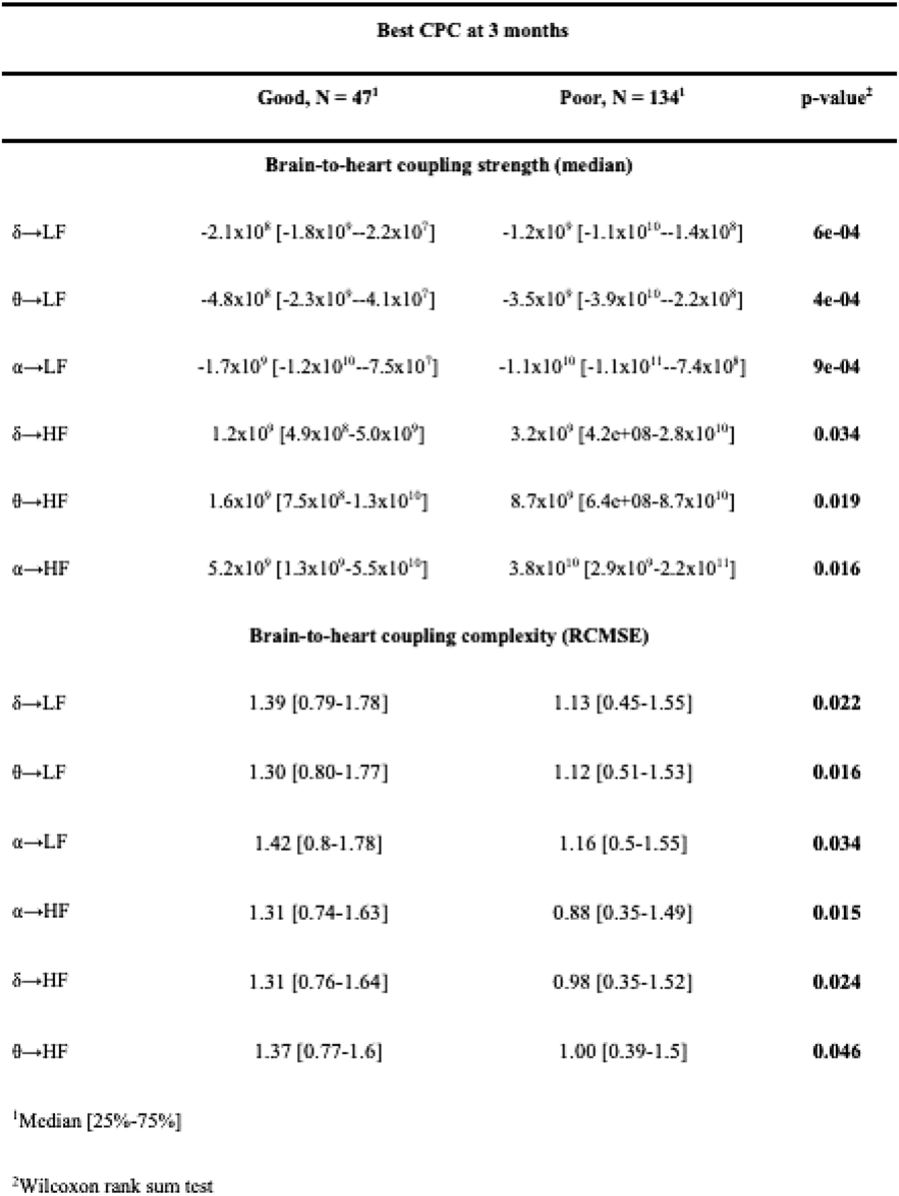
Brain-to-heart coupling strength and complexity according to patients’ outcomes

## CO-28 Distinct profiles of microglial activation are associated with neurological outcome in anoxic and traumatic coma

### Benjamine Sarton^1,2^, Clovis Tauber^1,3^, Patrice Peran^2^, Adrian David^1^, Stein Silva^1,2^

#### ^1^CHU Toulouse, Toulouse, France; ^2^INSERM—UMR 1214, Toulouse, France; ^3^Université de Tours, Tours, France

##### **Correspondence:** Benjamine Sarton (benjamine.sarton@hotmail.fr)

*Annals of Intensive Care *2013, **13(Suppl 1):**CO-28

**Rationale:** Coma resulting from traumatic brain injury (TBI) and anoxia, remains a major issue for prognostication and care. Accumulative evidence suggests that coma is due to a disconnection within a “consciousness network”: fronto-parietal cortex and subcortical structures (mesocircuit)(1). Inflammation is potentially one of the leading causes of this connectome disruption. PET imaging allows in-vivo brain inflammation insight, triggering TSPO receptor expressed by activated microglia (MA). We aimed to study acute neuroinflammation in coma and its relationship with neurological outcome. We hypothesised that (i) brain injuries triggers MA within brain structures responsible for conscious processing (ii) with distinct MA pattern for anoxic and traumatic mechanism (iii) there is a significant relationship between in-vivo MA features and patients 3 month’s neurological outcome.

**Patients and methods/materials and methods:** We conducted a cross sectional study of coma patients compared to controls. We included comatose adult patient, GCS < 9 with motor score < 6 induced by traumatic or anoxic brain injury (< 30 days), free from sedations. Patients and controls were excluded in case of neurological illness, anti-inflammatory medication, low affinity binding phenotype for TSPO receptor. Control group was gender and age matched. The protocol included a neurological examination, T1-MRI and TSPO PET with 18F-DPA-714 radioligand. Brain 18F-DPA-714 binding was quantified with a supervised clustering algorithm (2). Neurological outcome was assessed with Coma recovery scale (CRS-r) at 3 months. The ethic committee approved the study.

**Results:** Seventeen coma (6 anoxic and 13 TBI) patients and 20 controls were included (2019–2022). Significant increased binding potential (BP) of 18F-DPA-714 was observed in coma patient compared to control, with different intensity and spatial distribution between anoxic and traumatic groups (Figure 1A). Anoxic patients have showed significant increased BP in the thalamus, pallidum, putamen, posterior cingular cortex, medial prefrontal cortex (mPFC), precuneus; and in mPFC for trauma patients. A significant higher 18F-DPA-714 binding was observed in coma patients with unfavorable outcome (Figure 1B) notably in the pallidum (p = 0,001), putamen (p = 0,02) and PCC (p = 0,004).

**Conclusion:** For the first time we have in vivo identified significant neuroinflammation in acute coma. MA is identified into networks implicated in conscious processing. Interestingly, MA profile was different between anoxic and traumatic coma. MA intensity and its spatial distribution were associated with patient’s further neurological outcome. Those results provide new insights into the characterization of neuroinflammation after severe brain injury and hold promise for personalized treatment.

**Reference 1**

Giacino JT, Fins JJ, Laureys S, Schiff ND. Disorders of consciousness after acquired brain injury: the state of the science. Nat Rev Neurol. févr 2014;10(2):99?114.

**Reference 2**

2. Schubert J, Tonietto M, Turkheimer F, Zanotti-Fregonara P, Veronese M. Supervised clustering for TSPO PET imaging. Eur J Nucl Med Mol Imaging. déc 2021;49(1):257?68.

**Compliance with ethics regulations:** Yes in clinical research.

**Figure 1 (abstract CO-28):**
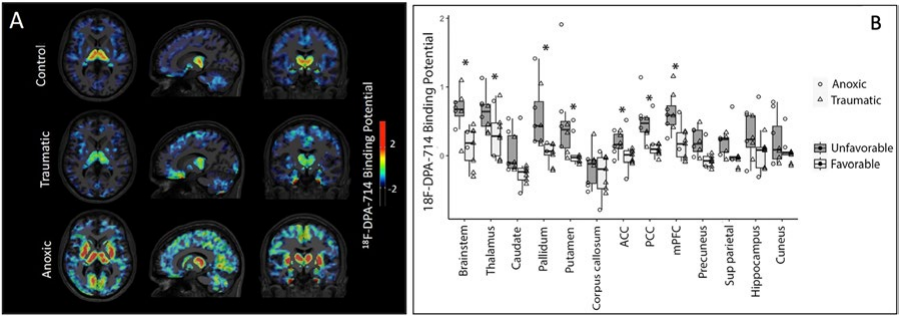
A In vivo microglial activation profiles (18F-DPA 714 BP) in control, traumatic and anoxic groups. 1.B. Microglial activation in vivo assessment in cortical and subcortical ROI and coma patient’s neurological outcome (favorable/unfavorable)

## CO-29 P3 event-related potential impairments in critically ill patients with delirium using a multidimensional cognitive electrophysiological battery

### William Buffieres^1,3^, Fabrice Ferre^1,2,3^, Lizette Heine^2^, Béatrice Riu^1^, Benjamine Sarton^1,3^, Fabien Perrin^2^, Stein Silva^1,3^

#### ^1^CHU Toulouse Purpan, Toulouse, France; ^2^Centre de Recherche en Neurosciences de Lyon, Lyon, France; ^3^Unité ToNIC INSERM UMR 1214, Toulouse, France

##### **Correspondence:** William Buffieres (buffieres.w@chu-toulouse.fr)

*Annals of Intensive Care *2013, **13(Suppl 1):**CO-29

**Rationale** Delirium is a common problem in critically ill patients but has only recently been recognized as associated with important clinical outcomes such as long-term cognitive impairment. Currently, there is a major knowledge gap related to the covert yet potentially incapacitating higher-order cognitive impairment underpinning this disorder of consciousness.

**Patients and methods/materials and methods:** The aim of the current study was to analyze the electrophysiological signatures of delirium by using a specifically designed multidimensional auditory event-related potentials (ERPs) battery to probe hierarchical cognitive processes, including automatic and voluntary attention, self-related processing and detection of arithmetic incongruences. Clinical variables and electrophysiological data were prospectively collected in controls subjects (n = 14) and in critically ill patients with (n = 18) and without (n = 20) delirium.

**Results:** Overall, we have specifically identified in patients with delirium, both a preservation of low-level central auditory processing—notably the detection of local regularity violation (MMN and P3a or ‘local effect’)—and a coherent ensemble of covert higher-order cognitive dysfunctions encompassing the processing of global regularity violation (P3b or ‘global effect’), self-relevant stimulus (P3 to subject's own name), and simple arithmetic facts violation (P3 to incorrect results) (spatio-temporal clustering, p-cluster ≤ 0.05). In this setting, estimation of the cortical generators of the P3 response to target stimuli by source modeling identified the predominant role of the frontal lobe in control subjects, whose activation was modulated downward by delirium.

**Conclusion:** Multidomain cognitive processes involving P3 ERP responses to auditory protocols are impaired in patients with delirium and could be due to frontal brain dysfunction. We suggest that our results shed new light on the neuropsychological disorders associated with ICU-related delirium and may constitute a valuable method for patient’s bedside diagnosis and monitoring in this clinically challenging setting.

**Compliance with ethics regulations:** Yes in clinical research.

## CO-30 Accuracy of tidal volume delivery by pediatric intensive care ventilators: a bench model study

### Meryl Vedrenne-Cloquet^1,4^, Samuel Tuffet^2,3,5^, Bruno Louis^3,5^, Sonia Khirani^1,4,6^, Charlotte Collignon^1^, Sylvain Renolleau^1,4^, Brigitte Fauroux^1,4^, Guillaume Carteaux^2,3,5^

#### ^1^Centre hospitalo-universitaire Necker-Enfants Malades, Paris, France; ^2^Centre hospitalo-universitaire Henri Mondor, Créteil, France; ^3^INSERM, CNRS ERL 7000, IMRB, Créteil, France; ^4^Université Paris Cité, Paris, France; ^5^Université Paris-Est Créteil, Créteil, France; ^6^ASV Santé, Genevilliers, France

##### **Correspondence:** Meryl Vedrenne-Cloquet (meryl.vedrenne@aphp.fr)

*Annals of Intensive Care *2013, **13(Suppl 1):**CO-30

**Rationale:** Accurate control of low tidal volumes (Vt) is crucial during pediatric protective ventilation. Vt delivery can be influenced by gas compression, humidity, temperature, and ventilatory circuit compliance, thus might be altered by the humidification system. The impact of the humidification system on the accuracy of Vt delivery during pediatric volume-controlled ventilation is unknown. The primary objective of this study was to assess the accuracy of Vt delivery by pediatric intensive care ventilators according to the humidification system. Secondary objectives were to assess: i) the accuracy of Vt delivery according to the presence or absence of an integrated Y-piece pneumotachograph; ii) the ability of ventilators to deliver and maintain the preset positive end-expiratory pressure (PEEP).

**Patients and methods/materials and methods:** Six modern intensive care ventilators equipped with a pediatric mode were tested on the ASL5000 test lung. Ventilators were tested in volume-controlled mode under 4 simulated pediatric bench models (full-term neonate (NN), infant (I), pre-school kid (PS), school kid (S)) with a heated humidifier (HH) or a heat-moisturer exchanger (HME), under various loading conditions (healthy, obstructive, restrictive), and with two levels of PEEP. Three ventilators equipped with Y-piece pneumotachograph were tested with or without the integrated pneumotachograph in the NN and I models. “Accurate Vt” delivery was defined as the volume error (percentage of the preset Vt under body temperature and pressure and saturated water vapour (BTPS) conditions) ≤ 10% of absolute preset value.

**Results:** Vt accuracy varied significantly across ventilators. The use of a HH or HME was associated with a significant difference in volume error for almost all the ventilators (p < 0.05). Ventilation with HH tended to deliver a lower-than-set Vt, the opposite was observed with HME, especially in the NN and I models. The use of an integrated pneumotachograph was associated with lower volume error in only one ventilator (p < 0.001). All the tested ventilators were able to maintain adequate PEEP levels.

**Conclusion:** The accuracy of Vt delivery is highly variable across pediatric intensive care ventilators during volume-controlled ventilation, and is altered by the humidification system, especially in the youngest patients.

**Reference 1**

Lyazidi A, Thille AW, Carteaux G, et al.: Bench test evaluation of volume delivered by modern ICU ventilators during volume-controlled ventilation. Intensive Care Med 2010; 36:2074–2080.

**Reference 2**

Lellouche F, Taillé S, Maggiore SM, et al.: Influence of ambient and ventilator output temperatures on performance of heated-wire humidifiers. Am J Respir Crit Care Med 2004; 170:1073–1079.

**Compliance with ethics regulations:** N/A.

**Figure 1 (abstract CO-30):**
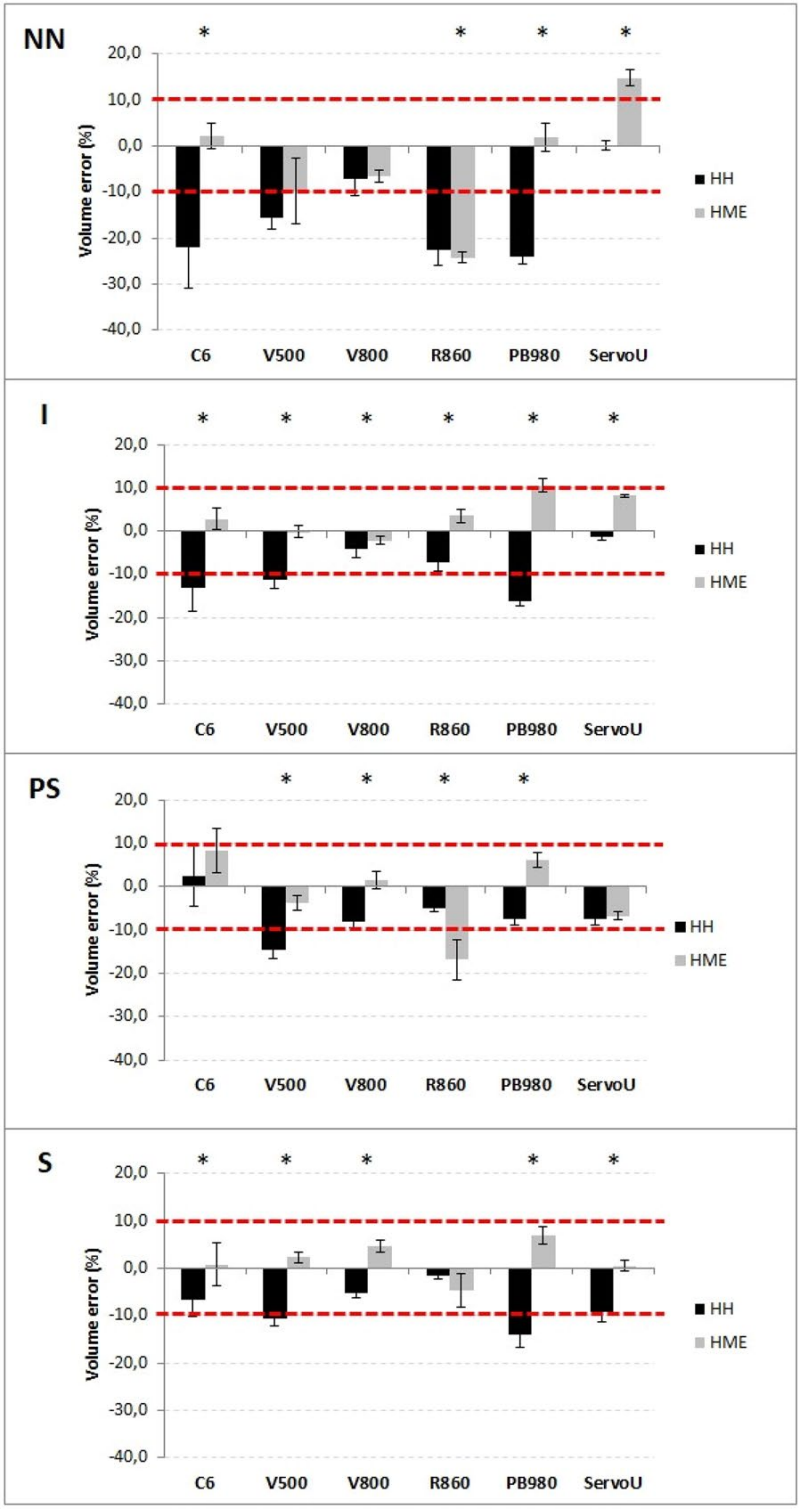
Comparison of the volume error in the tested ventilators according to the humidification system in the four pediatric bench models, each model being simulated with three respiratory mechanics (healthy, obstructive and restrictive). Data are presented as m

## CO-31 Electrical impedance tomography and clinical impact in a pediatric intensive care unit

### Sirine Boussena^1^, Robin Pouyau^2^, Fleur Cour Andlauer ^2^, Etienne Javouhey^2^, Florent Baudin^2^

#### ^1^Centre hospitalier universitaire vétérinaire-VetAgro Sup, APCSe., Marcy L'étoile, France; ^2^Hospices Civils de Lyon, Pediatric intensive care unit, Bron, France

##### **Correspondence:** Sirine Boussena (boussenasirine@gmail.com)

*Annals of Intensive Care *2013, **13(Suppl 1):**CO-31

**Rationale:** Electrical impedance tomography (EIT) is a non-invasive real-time bedside lung ventilation monitoring. The aim of the study was to describe the changes in PEEP (positive end expiratory pressure) values induced by an EIT-guided PEEP trial.

**Patients and methods/materials and methods:** We conducted a prospective study (Peluche 1) with IRB approval n°2022-A00505-38. All patient admitted in the PICU and monitored with EIT (Pulmovista 500, Draeger, France) were included after informed consent. Demographic and clinical data, indications and recordings from the EIT were collected and analyzed.

**Results:** Between June and December 2020, ten patients, median [IQR] age 4.2 years [1.7–6.9], were included in the study and nine had at least one EIT-guided PEEP trial. Among the 16 EIT-guided PEEP trials performed, ten led to a change of the level of PEEP: seven increases with a median change of 3 cmH20 [2–5] and 3 decreases (median [IQR] − 2 cmH2O [− 2; − 4]). For the 7 patients with a diagnosis of ARDS, the PEEP value set after the EIT-guided PEEP trial was (median [IQR]) 3 cmH20 [0–6] upper than the lowest value proposed by the PEEP/FIO2 table.

**Conclusion:** In pediatric intensive care unit, EIT-guided PEEP trial is feasible and resulted in change in PEEP for 2 third of patients.

**Compliance with ethics regulations:** Yes in clinical research.

## CO-32 ROX index at H0 and H1 to predict HFNC outcome in respiratory failure due to severe bronchiolitis

### Christophe Milesi^1^, Julien Baleine^1^, Juliette Apert^1^, Guillaume Mortamet^3^, Robin Pouyau^2^, Gilles Cambonie^1^

#### ^1^CHU Arnaud de Villeneuve, Montpellier, France; ^2^CHU Lyon, Lyon, France; ^3^CHU Grenoble, Grenoble, France

##### **Correspondence:** Christophe Milesi (c-milesi@chu-montpellier.fr)

*Annals of Intensive Care *2013, **13(Suppl 1):**CO-32

**Rationale:** The moderate forms of acute viral bronchiolitis (AVB) benefit from ventilatory support by High-Flow Nasal Cannul (HFNC) or Continuous Positive Airway Pressure (CPAP). HFNC can be used in the paediatric wards unlike CPAP, which requires ICU monitoring. Early identification of this group is an essential organisational element. The ROX index [pulsea oximetry/FiO2/respiratory rate] is a relevant tool in predicting the HFNC failure in hypoxic adult population. Our objective was to validate the ROX index before (H0) and 1 h (H1) after HFNC initiation to determine the failure in children with AVB.

**Patients and methods/materials and methods:** This is an ancillary study of a RCT performed in 16 pediatric intensive care units (TRAMONTANE 2), focusing on patients younger than 6 months with moderate to severe bronchiolitis ()defined by mWCAS score > 3)Data were collected at H0 and H1 (ROX index, heart and respiratory ratemWCAS score, EDIN scale).

**Results:** From November 2016 to March 2017, 286 infants were included. Failure occurred early, within the first 6 h for half of children. The ROXindex at H0 and H1 does not predict failure. H0: Area Under the Curve 0.56 (0.48; 0.63); H1: AUC 0.60 (0.53; 0.67). mWCAS was higher at H1 in the failure group (3.3 (± 1.0) vs 4.0 (± 1.5); p < 0.01). Comfort under HFNC improved more significantly in the successful group (Delta EDIN: − 2.18 (± 2.38) vs − 1.54 (± 2.34): p = 0.02).

**Conclusion:** ROX index cannot predict HFNC failure in patients with moderate bronchiolitis. **Compliance with ethics regulations:** Yes in clinical research.

**Table 1 (abstract CO-32):**
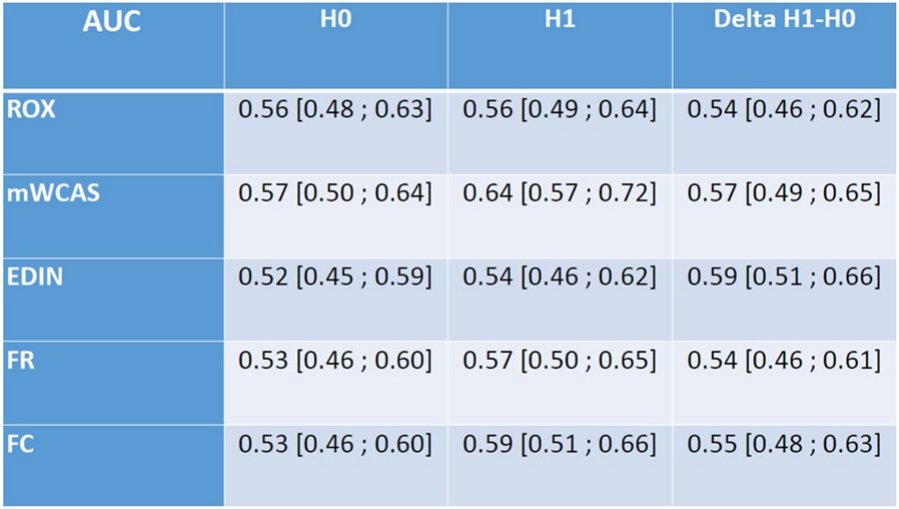
ROC and Area Under the Curve patient with HFNC failure

## CO-33 Parental experience of end-of-life signs in pediatric intensive care unit

### Coralie Nicolle^3^, Fleur Le Bourgeois^2^, Charlotte Pierron^1^

#### ^1^Centre hospitalier du Luxembourg, Luxembourg, Luxembourg; ^2^Hôpital Robert Debré, Paris, France; ^3^Université Paris Est-Créteil, Centre d’étude des discours, images, textes, écrits, communication, Créteil, France

##### **Correspondence:** Charlotte Pierron (pierron.charlotte@chl.lu)

*Annals of Intensive Care *2013, **13(Suppl 1):**CO-33

**Rationale:** Few studies reported the end-of-life (EOL) signs in pediatric intensive care unit (PICU), while these signs can be impressive or distressful for parents (1). The aim of this study was to assess the impact of EOL signs on parents' experience of their child's death.

**Patients and methods/materials and methods:** To evaluate the emotional state of parents confronted to EOL signs and their wishes on information they would like to receive, a psychologist interviewed parents, whose child had died in PICU during the previous months. Parents were informed of the study before their child's death and gave their consent to be contacted afterword. The interview was conducted face-to-face or by telephone and was semi-directive to avoid suggested answers. The interviews were transcribed and analyzed by a sociologist according to a qualitative method.

**Results:** We interviewed 11 parents, 2 to 27 months after the death of their child. As parents thought about their child's end of life, the EOL signs were not in the foreground. They talked about it only as the psychologist mentioned it. Changes on physical appearance, e.g. oedemas, pallor…, were the EOL signs the most often mentioned by parents. Those signs were connected to a symbolism for the parents, even when they received information from the healthcare professionals about these signs. They were associated with the disembodiment of their child, the child became a stranger to them, they no longer recognized him. Furthermore, signs related to respiratory movements evoked a battle between the child and death, as if the child was fighting to survive. About the information they would like to receive on EOL signs, they suggested that it should be given while the signs were occurring and not before, to prevent parents from actively searching for it. And above all, that this information should not be provided in writing, as it would seem violent for them. Our study has some limitations, the interviews did not occur at the same time for all parents, making it challenging to compare them and most of the parents had lost their child during the first week of life.

**Conclusion:** Parents who lost their child in PICU did not spontaneously mention EOL perceptible signs. The most common signs they cited were signs that modified appearance, which are the most common in critically ill children (2). The most appropriate information about these signs, according to the parents, should be given at the time of their occurrence, orally.

**Reference 1**

1. Perkin RM: The agony of agonal respiration: is the last gasp necessary? J Med Ethics 2002; 28:164–169

**Reference 2**

2. Pierron C, Levy M, Mattioni V, et al.: Perceptible Signs of End of Life in Pediatric Intensive Care Patients. J Palliat Med 2022; 25:1829–1834

**Compliance with ethics regulations:** Yes in clinical research.

**Table 1 (abstract CO-33):**
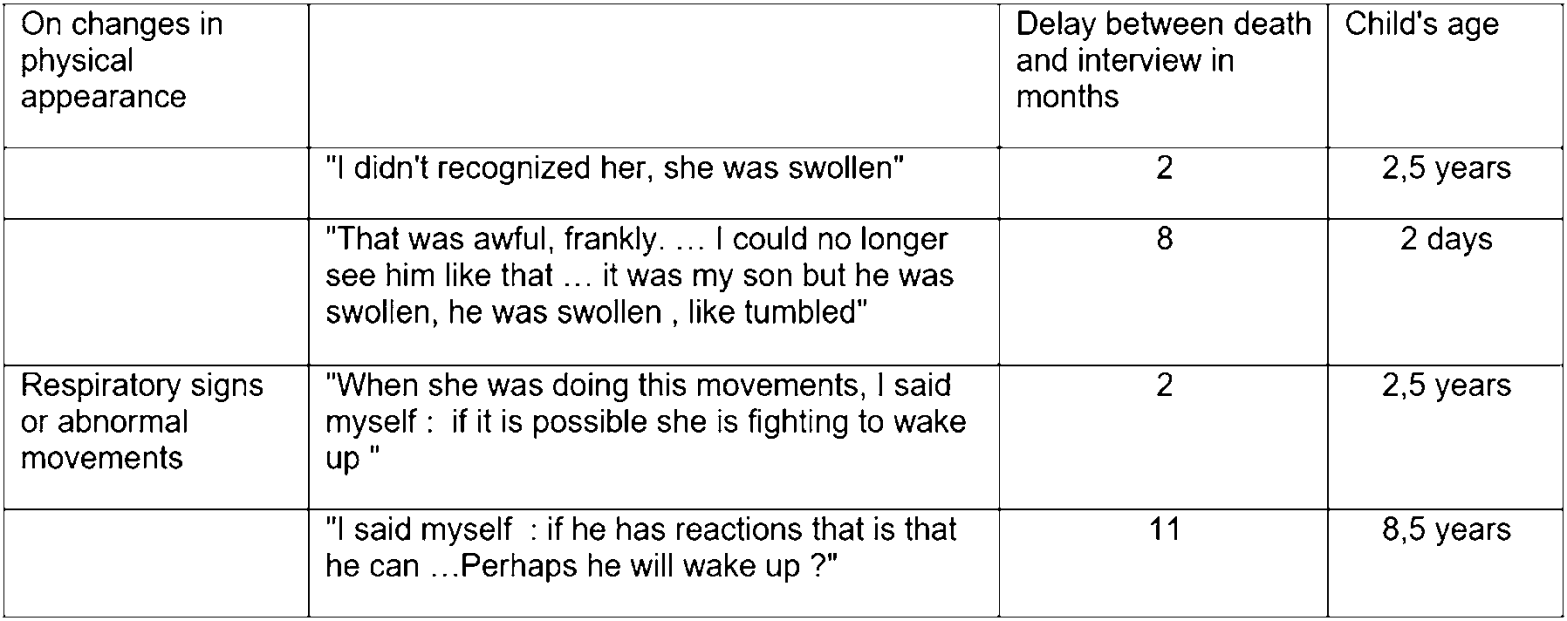
Quotes from parents' interviews

## CO-34 Influence of Parental Proximity on Reasonable Treatment Decisions in Pediatric Intensive Care

### Fleur Le Bourgeois^1^, Michael Levy^1^, Arielle Maroni^1^, Violaine Mattioni^1^, Jérôme Naudin^1^, Géraldine Poncelet^1^, Alain Lefevre Utile^2^

#### ^1^Hôpital Robert Debré, Paris, France; ^2^Hôpital Jean Verdier, Bondy, France

##### **Correspondence:** Fleur Le Bourgeois (fleurlebourgeois@gmail.com)

*Annals of Intensive Care *2013, **13(Suppl 1):**CO-34

**Rationale:** In France, decision to withdraw or limit life-sustaining treatment (WLST) in pediatrics is a medical decision discussed collectively, it is based mainly on medical criteria. However, subjective factors can influence this decision. To what extent do pediatric intensive care unit (PICU) healthcare professionals' (HCP) perceptions of the possibility of future parental presence and involvement influence the decision that is considered most appropriate?

**Patients and methods/materials and methods:** In a PICU, HCP were asked to fill out an anonymous electronic survey. It presented a vignette, where a decision of WLST is discussed. It was a 2-month-old patient with multi-organ disease, whose neurological, digestive, and respiratory prognosis were uncertain. The patient was dependent on mechanical ventilation and parenteral nutrition. HCP were asked to choose between continuation of artificial organ support in long-term care institution (LTCI), palliative support care until death or no decision. Five vignettes were presented: unknown parental location, parents living near the institution, parents living abroad, a single mother living 1 h from the institution and the child was an orphan. Qualitative data were described as numbers and percentages.

**Results:** Fifty-eight HCP completed the questionnaire, HCP’s decisions changed dramatically according to scenario (Figure 1). When the parental background was unknown, the decision was balanced between all alternatives. When the parents were living nearby the LTCI, 64% of HCP decided continuation of treatments, conversely, if the parents were living far away or completely absent, a WLST was decided by 64 and 79% of HCP respectively.

**Discussion:** Although the decision of WLST mainly relies on medical factors, it considers non-measurable as the parents ‘ability to easily visit and accompany their multi-disabled child in a LTCI. The quality of the bond between parents and their child is difficult to predict. However, the possible absence or the difficulty in developing one, due to distance from the LTCI, or even the absence of a parent, seems to be a pejorative factor in HCP’s projection, of the child’s future situation. In this decision-making process, HCPs’ perceptions of the possibility of future parental involvement seem to be oversimplified and translated into a stereotypical assumption: parents will take better care of their child because they live near the LTCI, and conversely. To limit these biases, multidisciplinary collegial meetings should allow reasoned discussion before a decision of WLST.

**Conclusion:** This study highlights the need to be aware of these biases and their impact, and to know how to identify them.

**Compliance with ethics regulations:** Yes in clinical research.

**Figure 1 (abstract CO-34):**
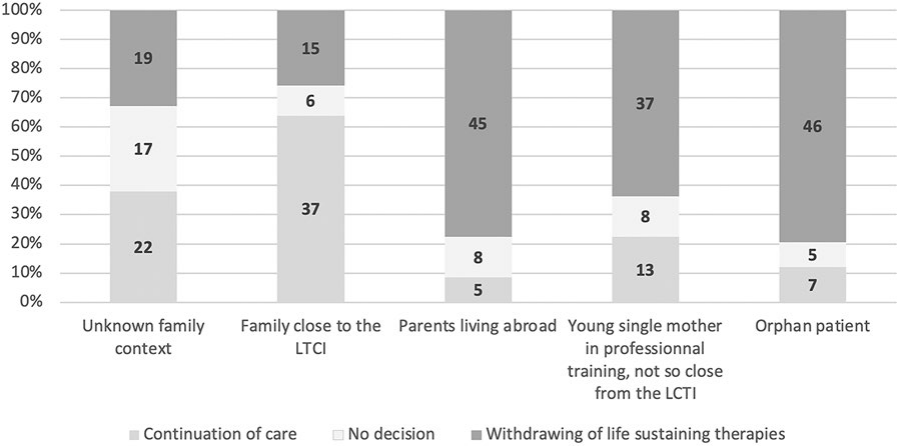
Decisions of the respondents about appropriate therapy according to parental possibility, or not, to easily visit and accompany their multi-disabled child in the LCTI

## CO-35 Feasibility of performing PICU follow-up in a Pediatric Tertiary Care Institution

### Simon Macdonald^1^, Geneviève Du Pont-Thibodeau^1^, Lara-Kim Huynh^2^, Miriam Beauchamp^2^, Karen Harrington^1^, Nadia Roumeliotis^1^, Jacques Lacroix^1^, Catherine Farrell^1^, Camille Jutras^1^, Imen Hamdi^2^, Vincent Lague^2^, Laurence Ducharme-Crevier^1^

#### ^1^CHU Sainte-Justine, Montréal, Canada; ^2^Centre de Recherche, CHU Sainte-Justine, Montréal, Canada

##### **Correspondence:** Simon Macdonald (simon.macdonald@umontreal.ca)

*Annals of Intensive Care *2013, **13(Suppl 1):**CO-35

**Rationale:** Organizing follow-up of Pediatric Intensive Care Unit (PICU) survivors is challenging. Many children have complex medical conditions that require multiple appointments. Scheduling and transportation constraints are common barriers to follow-up. Participation rates to PICU follow-up have been very variable with some studies reporting rates as low as 30%. We evaluated the participation rate of families to a PICU follow-up clinic offering flexible schedules, dedicated research personnel and the option to choose in-person versus telemedicine follow-ups.

**Patients and methods/materials and methods:** This is a retrospective descriptive study of the feasibility of a post-PICU clinic in a pediatric tertiary care university hospital in the year 2021 (post-pandemic). Inclusion criteria to the clinic were: age < 18 years at PICU admission, PICU hospitalization > 96 h, AND invasive ventilation > 48 h OR non-invasive ventilation > 96 h. Follow-up is planned 2 months post-PICU discharge with a dedicated PICU provider. To optimize patient participation, the following strategies were put into place: (1) patient enrolment obtained prior to PICU discharge, (2) information on post-PICU clinic and post-PICU complications provided prior to discharge with an informative pamphlet, (3) dedicated research personnel responsible to track participants and organize follow-ups, (4) flexible days and schedule options offered for accommodation, including rescheduling, (5) six PICU providers available at various time periods, (6) option to choose between in person vs. telemedicine appointments, (7) visits planned at same time as other hospital appointments when possible, (8) research personnel meeting with families at hospital entrance to guide to appointment, (9) phone call reminders done 1 week prior to appointment. A subgroup of children followed at the clinic underwent 15–60 min neurocognitive evaluations. They were performed either in-person or virtually.

**Results:** From 01/2021 to 12/2021, 103/123 (83.8%) patients were enrolled. Most common reasons for non-enrollment were: (1) difficulties to reach parents, (2) no interest in additional follow-up, (3) distance from hospital, (4) language barrier. Of those enrolled, 82/103 (79.6%) patients were evaluated at 2-months follow-up. 23.2% of appointments were performed virtually. 13.6% of appointments needed to be rescheduled to accommodate parents. Of 23 children that were offered neurocognitive evaluations, 30.4% were performed in-person and 69.6% were performed virtually and all evaluations were completed.

**Conclusion:** Performing PICU follow-up clinic is feasible with a strong participation rate when strategies including a dedicated team, scheduling flexibility and virtual options are set into place.

**Reference 1**

Maddux A.B., Pinto N., Fink E.L., Hartman M.E., Nett S., Biagas K., Killien E.Y., Dervan L.A., Christie L.M., Luckett P.M., et al. Postdischarge Outcome Domains in Pediatric Critical Care and the Instruments Used to Evaluate Them: A Scoping Review. Crit.

**Compliance with ethics regulations:** Yes in clinical research.

## CO-36 Prospective comparison of prognostic scores for prediction of outcome after out-of-hospital cardiac arrest: results of the AfterROSC1 multicentric study

### Jean Baptiste Lascarrou^1,2^, Wulfran Bougouin^2,3^, Jonathan Chelly^4^, Jeremy Bourenne^5^, Cedric Daubin^6^, Olivier Lesieur^7^, Pierre Asfar^8^, Gwenhael Colin^9^, Marine Paul^10^, Nicolas Chudeau^11^, Gregoire Muller^12^, Guillaume Geri^13^, Sophie Jacquier^14^, Nicolas Pichon^15^, Bruno Levy^16^, Bertrand Sauneuf^17^, Kada Klouche^18^, Martin Cour^19^, Caroline Sejourne^20^, Fabio Taccone^21^, Jean-Herle Raphalen^22^, Arnaud Galbois^23^, Cedric Bruel^24^, Nicolas Mongardon^25^, Nadia Aissaoui^2,29^, Nicolas Deye^27^, Julien Maizel^28^, Florence Dumas^2,29^, Stephane Legriel^10^, Alain Cariou^2,29^

#### ^1^CHU Nantes, Nantes, France; ^2^PARCC, Paris, France; ^3^Hôpital Jacques Cartier, Massy, France; ^4^CH Toulon, Toulon, France; ^5^APHM, Marseille, France; ^6^CHU Caen, Caen, France; ^7^CH La Rochelle, La Rochelle, France; ^8^CHU Angers, Angers, France; ^9^CHD Vendée, La Roche Sur Yon, France; ^10^CH Versailles, Le Chesnay, France; ^11^CH Le mans, Le Mans, France; ^12^CHR Orléans, Orléans, France; ^13^CHU Ambroise Pare, Boulogne-Billancourt, France; ^14^CHRU Tours, Tours, France; ^15^CH Bourges, Bourges, France; ^16^CHU Nancy, Nancy, France; ^17^CH Cherbourg, Cherbourg En Cotentin, France; ^18^CHU Montpellier, Montpellier, France; ^19^HCL, Lyon, France; ^20^CH Béthune, Béthune, France; ^21^ERASME, Bruxelles, France; ^22^APHP, Necker, Paris, France; ^23^Hôpital Privé Claude Galien, Quincy Sous Sénart, France; ^24^Groupe Hospitalier Paris Saint Joseph, Paris, France; ^25^APHP, Mondor, Créteil, France; ^26^APHP, HEGP, Paris, France; ^27^APHP, Lariboisière, Paris, France; ^28^CHU Amiens, Amiens, France; ^29^APHP, Cochin, Paris, France

##### **Correspondence:** Jean Baptiste Lascarrou (jeanbaptiste.lascarrou@chu-nantes.fr)

*Annals of Intensive Care *2013, **13(Suppl 1):**CO-36

**Rationale:** Out-of-hospital cardiac arrest (OHCA) is a heterogeneous entity with multiple origins and various prognosis. An early and reliable assessment of the prognosis is useful for various reasons, in particular to adapt the therapeutic strategy and to inform relatives. Our aim was to evaluate a large panel of dedicated scores that predict outcome after OHCA in a prospective multicentric study.

**Patients and methods/materials and methods:** We prospectively collected data from 24 French ICUs between August 2020 and June 2022. All cases of non-traumatic OHCA (both cardiac and non-cardiac cause) patients with stable return of spontaneous circulation (ROSC) and comatose at ICU admission (defined by Glasgow coma score ≤ 8) were included. Primary outcome was the modified Rankin scale at day 90 after cardiac arrest assessed by phone interview. A large panel of developed scores (CAHP; OHCA; CREST; C-Graph; TTM; CAST; NULL-Please; MIRACLE2) were included in the analysis. Their accuracies in predicting poor outcome at 3 months after OHCA were determined using the area under the receiving operating characteristic curve (AUROC) and calibration belt with Utstein style criteria as a reference.

**Results:** During the study period (22 months), 907 patients were screened and 658 were included in the study. Patients were mainly male (72%), 61 ± 15 years old and mostly collapsed from supposed cardiac cause (64%). Mortality rate at day 90 was 63% and an unfavorable neurological outcome was observed in 66%. Performance (AUROC) of Utstein criteria for poor outcome prediction was 0.79 [0.76–0.83] whereas AUROC from others score included varied from 0.79 [0.75–0.83] to 0.88 [0.86–0.91]. Considering their respective AUROC, the 3 most performant scores were the TTM (0.88 [0.86–0.91]), the CAHP (0.87 [0.84–0.90] and the mCAHP (0.86 [0.83–0.89]). At the opposite, the 3 less performant scores were the C-GRAPH (0.76 [0.71–0.80]), the CREST (0.79 [0.75–0.83]) and the NULL-PLEASE (0.81 [0.77–0.84]). For each score, the proportion of patients for whom individual value cannot be determinable varied from 1.4% to 17.4%.

**Conclusion:** In patients admitted to intensive care after a cardiac arrest, most of the scores available for the evaluation of the subsequent prognosis are more efficient than the usual Utstein criteria. Some of these scores revealed superior performance to others, even if the observed differences are modest. If a choice must be made among these different scores, the immediate availability of all the variables in the environment should guide this choice.

**Compliance with ethics regulations:** Yes in clinical research.

## CO-37 Diastolic arterial pressure normalized to the vasopressor-dose in patients with septic shock as a marker of vasoplegia: a post-hoc analysis of the ANDROMEDA-SHOCK study

### Antoine Goury^1^, Zoubir Djerada^2^ , Glenn Hernandez^3^, Romain Griffon^1 ^^2^, Bruno Mourvillier^1^, Jean-Louis Teboul^4 ^^1^, Olfa Hamzaoui^1^ 

#### ^1^Service de Médecine Intensive-Réanimation Polyvalente, Hôpital Robert Debré, Centre hospitalo-universitaire de Reims, Reims, France; ^2^Department of Pharmacology, Reims University Hospitals, Reims, France; ^3^Department of intensive care, Pontifical Catholic University of Chile, Santiago, CHILI; ^4^Service de médecine intensive-réanimation, Hôpital de Bicêtre, AP-HP Université Paris-Saclay, INSERM-UMR_S999, Le Kremlin-Bicêtre, France

##### **Correspondence:** Romain Griffon (rgriffon@chu-reims.fr)

*Annals of Intensive Care *2013, **13(Suppl 1):**CO-37

**Rationale:** Diastolic arterial pressure (DAP) is mainly determined by vascular tone and less by heart rate (HR) and arterial stiffness. Consequently, in septic shock patients, vasoplegia may be diagnosed simply by a low value of DAP. However, the doses of vasopressors (VP) should be taken into account when correlating values of DAP to the degree of vasoplegia. Due to the lack of clinical data, DAP tends to be neglected and underused by the clinicians. We hypothesized that if the DAP/VP dose ratio is a better indicator of the degree of vasoplegia than DAP alone, it would be more associated with mortality than DAP alone.

**Patients and methods/materials and methods:** This is a post-hoc analysis of ANDROMEDA-SHOCK trial, a multicenter randomized controlled trial conducted between March 2017 to April 2018 in 28 hospitals in 5 countries. All patients included were admitted in intensive care unit (ICU) for septic shock. Systolic arterial pressure (SAP) and DAP were directly measured using an arterial catheter, which also displayed the mean arterial pressure (MAP) by calculating it from the SAP and DAP values. We calculated the DAP/VP dose ratio at inclusion time and compared it between survivors and non-survivors. We also calculated the SAP/VP dose ratio. A ROC curve was performed to define the statistical performance of the DAP/VP dose ratio and compared it to ROC curves constructed for DAP/HR and DAP alone.

**Results:** A total of 424 patients were included in the analysis. Median age was 66 [52–76;IQR]. Median SOFA score and SAPSII at baseline were 10 [7–12;IQR] and 21 [17–28;IQR], respectively. Median ICU length of stay was 6 [3–12;IQR]. In-hospital mortality rate was 43%. There was a significant difference between survivors and non survivors for DAP/VP dose (426 ± 24 in the survivors group and 283 ± 22 in the non-survivors group; p = 0.001), but not for the SAP/VP dose (800 ± 44 in the survivors group and 542 ± 44 in the non-survivors group; p = 0.055) (Figure 1). The AUC of DAP/VP ratio was 0.661 (Se = 54, Sp = 73; p = 0.001) for predicting in-hospital mortality and was significantly higher than the AUC of DAP/HR (p = 0.0002) and DAP alone (p = 0.0002) (Figure 1).

**Conclusion:** The DAP/VP dose ratio calculated at the early phase of septic shock is probably a better indicator of the degree of vasoplegia than DAP alone as it is better associated with poor outcome than DAP alone.

**Compliance with ethics regulations:** Yes in clinical research.

**Figure 1 (abstract CO-37):**
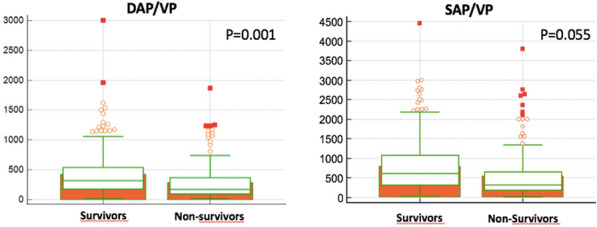
Difference between survivors and non survivors according to DAP/VP dose and SAP/VP dose ratio for the in-hospital mortality. DAP: diastolic arterial pressure. VP: vasopressors dose. SAP: systolic arterial pressure. There was a significant difference between survivors and non suvivors for DAP/VP dose (426 ± 24 in the survivor group and 283 ± 22 in the non-survivour group; p= 0.001), but not for the SAP/VP dose (800 ± 44) in the survivor group and 542 ± 44 in the non-survivour group; p= 0.005)

## CO-38 Severe cardiogenic acute pulmonary oedema and ventilation strategies. A French survey involving 1048 physicians

### Grégoire Muller^1,2^, Clément Delmas^5^, Saïd Laribi^3^, Étienne Puymirat^4^, Tahar Chouihed^6^, Nicolas Danchin^4^, Denis Angoulvant^2,3^, Nadia Aissaoui^7^

#### ^1^CHR Orléans, Orléans, France; ^2^Université de Tours, Tours, France; ^3^CHRU Tours, Tours, France; ^4^Hôpital Européen Georges Pompidou, Paris, France; ^5^CHU de Toulouse, Toulouse, France; ^6^CHU de Nancy, Nancy, France; ^7^CHU Cochin, Paris, France

##### **Correspondence:** Grégoire Muller (muller.gregoire.chro@protonmail.com)

*Annals of Intensive Care *2013, * 13(1): *CO-38

**Rationale:** To determine whether current management of severe acute pulmonary oedema (APE) is in line with international guidelines and differs according to medical specialty and recognized experts in the field, we designed a survey withfour case vignettes presenting different phenotypes of APE [1].

**Patients and methods/materials and methods:** Four clinical cases vignettes were designed by a scientific committee representing the scientific societies of emergency medicine, cardiology, and intensive care. In addition, 20 experts were enrolled according to their publications and involvement in the scientific societies. Target participants and designated experts were French physicians in cardiology, intensive care medicine and emergency medicine. From 06/2022 to 09/2022, the vignettes were submitted to the physicians and experts using an open online survey.

**Results:** Among the 1048 respondents 60% emergency physicians, 22% intensivists and 18% cardiologists), 781 completed the 4 cases whereas the 20 experts completed the whole survey.

- SpO_2_ < 90% threshold for O_2_ administration (ESC Guidelines I-C recommendation [1]): only 22% of physicians acted according to guidelines (SpO_2_ < 90%), 47% administered O_2_ for SpO_2_ < 95%, and 31% gave O_2_ in case of respiratory distress. The results were homogeneous according to physician specialty. Among experts, 53% followed the guidelines.
- Non-invasive ventilation (NIV) for respiratory distress (Respiratory rate > 25/mn, SpO_2_ < 90%) (IIa-B recommendation): 54% used NIV because of respiratory rate > 30/mn, 83% because of respiratory distress signs, 43% according to oxygen flow rate, and 42% in case of past COPD history. Except for past COPD history (cardiologists 32%, intensivists 52%, emergency physicians 41%), results were homogeneous according to physician specialty. Among the experts, the respective figures were 93%, 87%, 67%, 47%. BiPAP was chosen in 66% (respectively 55%, 84%, 61%, 73%, in cardiologists, intensivists, emergency physicians, experts), whereas CPAP in 34%.
- Intubation for progressive respiratory failure persisting despite O_2_ or NIV (I-C recommendation): in case of pre-hospital NSTEMI worsening despite NIV and medications, only 55% used mechanical ventilation (respectively 65%, 73%, 43%, 85% in cardiologists, intensivists, emergency physicians, experts). In case of STEMI worsening while waiting for coronary angiography, 44% used mechanical ventilation (respectively 45%, 70%, 33%, 70% in cardiologists, intensivists, emergency physician, experts).

**Conclusion:** Ventilation strategies regarding classical clinical cases of severe APE remain highly variable, and variations exist among medical specialties. The low level of evidence of the ESC Guidelines may explain these heterogeneities [2]. Further research is needed to fill the current gap in evidence.

**Reference 1**

McDonagh TA, Metra M, Adamo M, Gardner RS, Baumbach A, Böhm M, et al. 2021 ESC Guidelines for the diagnosis and treatment of acute and chronic heart failure. Eur Heart J. 2021;42:3599–726.

**Reference 2**

Aissaoui N, Hamzaoui O, Price S. Ten questions ICU specialists should address when managing cardiogenic acute pulmonary oedema. Intensive Care Med. 2022;48:482–5.

**Compliance with ethics regulations:** Yes in clinical research.

**Table 1 (abstract CO-47):**
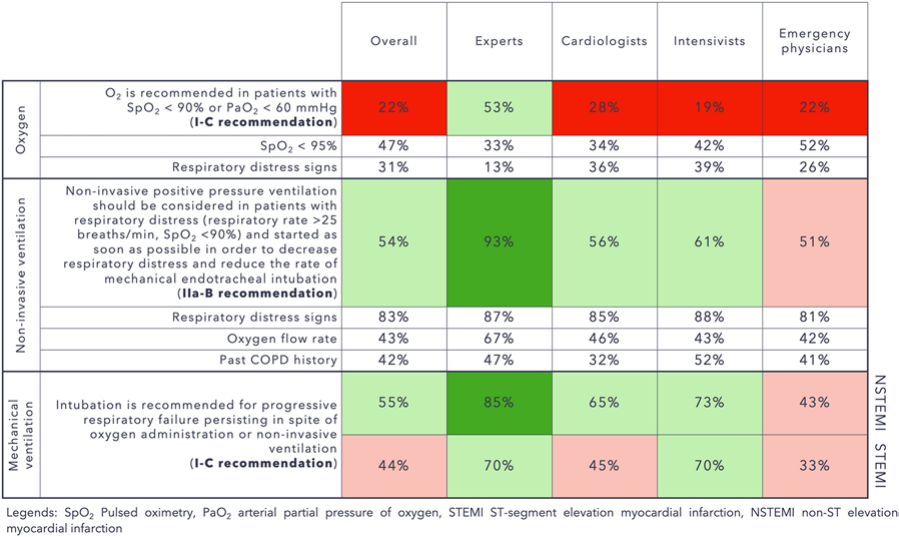
Quotes from parents' interviews

## CO-39 Outcomes and Clinical Impact of Early Endomyocardial Biopsy in Patients with Fulminant Myocarditis

### Florent Huang^1,2^, Enrico Ammirati^24^, Maharajah Ponnaiah ^2^, Santiago Montero^2,38^, Victor Raimbault^2^, Darryl Abrams^35^, Guillaume Lebreton^2^, Vincent Pellegrino^29^, Joshua Ihle^29^, Maurizio Bottiroli^24^, Romain Persichini^11^, Marisa Isabel Barrionuevo-Sánchez^18^, Albert Ariza Solé^18^, Pauline Ng Yeung^34^, Simon Sin Wai Ching^34^, Raj Ayer^30^, Hergen Buscher^30^, Clément Delmas^7^, Slimane Belaid^7^, Rita Ferreira^26^, Roberto Roncon-Albuquerque Jr^26^, Teresa Lopez-Sobrino^12^, Jeroen Jh Bunge^22^, Christoph Fisser^27^, Guillaume Franchineau^4^, Jamie Mccanny^25^, Shinichiro Ohshimo^33^, Alessandro Sionis^13^, Francisco José Hernández-Pérez^15^, Eduardo Barge-Caballero^20^, Martin Balik^28^, Henrique Muglia^23^, Sunghoon Park^32^, Dirk W Donker^21^, Beatriz Porral^17^, Nadia Aïssaoui^36^, Armand Mekontso Dessap^3^, Virginia Burgos^16^, Matthieu Lesouhaitier^6^, Justin Fried^35^, Jae-Seung Jung^31^, Sandra Rosillo^14^, Vincent Scherrer^5^, Saad Nseir^9^, Hadrien Winiszewski^10^, Pablo Jorge^19^, Antoine Kimmoun^8^, Rodrigo Diaz^37^, Alain Combes^2^, Matthieu Schmidt^2^

#### ^1^Hôpital Foch, Suresnes, France; ^2^CHU Pitié Salpêtrière, Paris, France; ^3^CHU Henri Mondor, Créteil, France; ^4^CHU Bichat, Paris, France; ^5^CHU Rouen, Rouen, FRANCE; ^6^CHU Pontchaillou, Rennes, France; ^7^CHU Rangueil, Toulouse, France; ^8^CHU Nancy, Nancy, France; ^9^CHU Lille, Lille, France; ^10^CHU Besançon, Besançon, France; ^11^CHU Félix-Guyon, Saint-Denis, La Réunion, France; ^12^Hospital Clínic de Barcelona, Barcelone, Espagne; ^13^Hospital de la Santa Creu i Sant Pau, Barcelone, Espagne; ^14^Hospital Universitario La Paz, Madrid, Espagne; ^15^Hospital Puerta de Hierro, Madrid, Espagne; ^16^Hospital Marqués de Valdecilla, Santander, ESPAGNE; ^17^Hospital Alvaro Cunqueiro, Vigo, Espagne; ^18^Hospital Universitario de Bellvitge, Barcelone, Espagne; ^19^Tenerife Hospital, Tenerife, ESPAGNE; ^20^CHUAC, La Corogne, Espagne; ^21^UMC Utrecht, Utrecht, PAYS-BAS; ^22^Erasmus Hospital, Rotterdam, PAYS-BAS; ^23^Hôpital Erasme, Bruxelles, BELGIQUE; ^24^Niguarda Hospital, Milan, Italie; ^25^Guy's and St Thomas Trust hospital, Londres, ROYAUME-UNI; ^26^Hospital S.João, Porto, Portugal; ^27^University Medical Centre Regensburg, Regensburg, Allemagne; ^28^General University Hospital, Prague, Republique Tcheque; ^29^Alfred Hospital, Melbourne, Australie; ^30^St Vincent hospital, Sydney, Australie; ^31^Korea University Medical Center, Seoul, Republique de Coree; ^32^Hallym University Sacred Heart Hospital, Anyang, Republique de Coree; ^33^Hiroshima Hospital, Hiroshima, Japan; ^34^Queen Mary Hospital, Hong Kong, CHINE; ^35^New York Presbitarian Hospital, New York City, ETATS-UNIS; ^36^Milton S. Hershey Medical Center, Hershey, ETATS-UNIS; ^37^Clínica Las Condes, Las Condes, Chili; ^38^Hospital Germans Trias i Pujol, Barcelona, Espagne

##### **Correspondence:** Florent Huang (florenth27@hotmail.com)

*Annals of Intensive Care *2013, * 13(1): *CO-39

**Rationale:** While endomyocardial biopsy (EMB) is recommended in adult patients with fulminant myocarditis (FM), the clinical impact of its timing is still unclear.

**Patients and methods/materials and methods:** Data were collected from 419 adult patients with clinically suspected FM admitted to intensive care units (ICUs) across 36 tertiary centers in 15 countries worldwide. Histology or cardiac magnetic resonance imaging confirmed the diagnosis in 306 (73%) cases. The primary outcome of survival free of heart transplantation (HTx) or left ventricular assist device (LVAD) at one year was specifically compared between patients with early EMB (within two days after ICU admission, n = 103) and delayed EMB (n = 80). A propensity score-weighted analysis was done to control for confounders.

**Results:** Median age on admission was 40 [29–52] years and 322 (77%) patients received temporary mechanical circulatory support. 273 (65%) patients survived without HTx/LVAD. The primary outcome was significantly different between patients with early and delayed EMB (70% vs. 49%, p = 0.004). After propensity score weighting, the early EMB group still significantly differed from the delayed EMB group in terms of survival free of HTx/LVAD (63% vs. 40%, p = 0.021). Moreover, early EMB was independently associated with a lower rate of death or HTx/LVAD at one year (odds ratio of 0.44; 95% confidence of interval: 0.22–0.86; p = 0.016).

**Conclusion:** EMB should be broadly and promptly used in patients admitted to ICU for clinically suspected FM.

**Compliance with ethics regulations:** Yes in clinical research.

## CO-40 Quantitative and qualitative assessment of fluid balance in septic shock

### Lisa Raia^1^, Julien Charpentier^1^, Sarah Benghanem^1^, Alain Cariou^1^, Jean-Paul Mira^1^, Nadia Aissaoui^1^, Frédéric Pene^1^

#### ^1^Hôpital Cochin, Paris, France

##### **Correspondence:** Lisa Raia (lr.lisa.raia@gmail.com)

*Annals of Intensive Care *2013, * 13(1): *CO-40

**Rationale:** Intravenous fluid therapy is a daily concern in the management of ICU patients, especially in patients with acute circulatory failure. Several studies reported the consistent association between positive fluid balance and outcome. However, the assessment of intravenous fluids is most often restricted to fluids given for resuscitation fluids, whereas the contribution of alternative fluid infusions to fluid balance has been poorly estimated. We herein aimed at quantifying the whole determinants and trends in fluid balance in septic shock, as well as the impact on outcome.

**Patients and methods/materials and methods:** We conducted a retrospective study in a medical ICU over a 13-year period (2008–2020). Adult patients with septic shock fulfilling the Sepsis-3 definition within 48 h of ICU admission were included. Three periods of admission (2008–2011, 2012–2015 and 2016–2020) were predefined. The components of fluid balance were extracted from the patient data management system from admission to day-7. We also assessed the impact of fluid balance on durations under organ supports.

**Results:** Among 1756 patients admitted to the ICU for septic shock, 954 patients were included. Pneumonia was the first source of infection (42.3%), and 793 (83.2%) patients required invasive mechanical ventilation. ICU and hospital mortality rates were 38.9% and 46.6% respectively. From their admission to day7, patients received a median of 1.8 [1.0–3.3] L of intravenous fluids per day. Fluid intake was 5.3 [3.6–7.5] L at day-1, and then rapidly decreased from day-2 and thereafter. Resuscitation fluids accounted for an important proportion (45.2%) of intravenous intake on day-1 only. From day-2, maintenance and vehicle fluids were mostly responsible for daily fluid intake, and therefore for fluid balance. There was a significant inverse relationship between the cumulative fluid balance within the first three days and the number of days alive without organ support (invasive mechanical ventilation and vasopressors). Patients admitted after 2011 received significantly less fluid between day-2 and day-6, associated with increased use in albumin during the initial resuscitation phase.

**Conclusion:** Fluid balance in septic shock is mainly determined by maintenance and vehicle fluids from day-2, whereas the overall contribution of resuscitation fluids is limited. These data suggest that targeted interventions based on non-resuscitation fluids are likely to result in major impact on fluid balance.

**Compliance with ethics regulations:** Yes in clinical research.

**Figure 1 (abstract CO-40):**
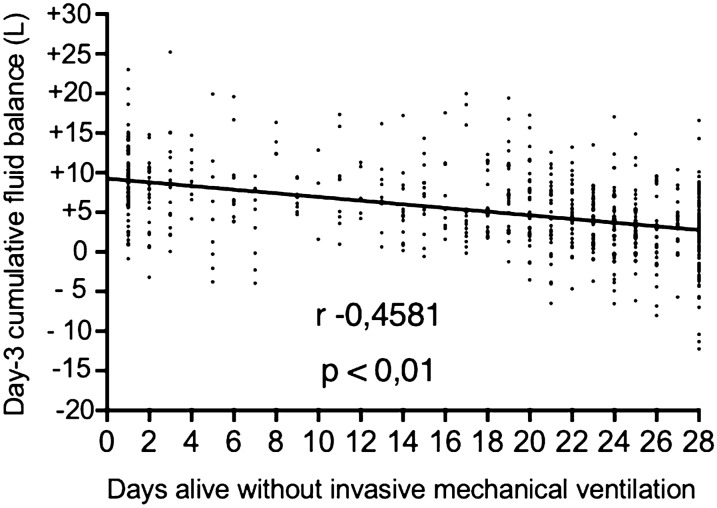
Correlation between day-3 cumulative fluid balance (in liters) and days alive without invasive mechanical ventilation

## CO-41 RV injury in COVID-19 ARDS patients characterized by critical care echocardiography: definitions, evolution and association with ICU mortality. A post-hoc analysis of longitudinal data from the ECHO-COVID study

### Stephen Huang^2^, Antoine Vieillard-Baron^3^, Bruno Evrard^1^, Gwenaël Prat^5^, Michelle S Chew^6^, Martin Balik^7^, Fernando Clau-Terré^8^, Daniel De Backer^9^, Armand Mekontso-Dessap^10^, Sam Orde^2^, Andrea Morelli^11^, Filippo Sanfilippo^12^, Cyril Charron^3,4^, Philippe Vignon^1^

#### ^1^Réanimation polyvalente, Inserm CIC 1435 et UMR 1092, CHU Dupuytren, Limoges, France; ^2^Intensive Care Medicine, Nepean Hospital, NBMLHD, The University of Sydney, Sydney, Australie; ^3^Service de Médecine Intensive Réanimation, Assistance Publique-Hôpitaux de Paris, University Hospital Ambroise Paré, Boulogne-Billancourt, France; ^4^INSERM UMR 1018, Clinical Epidemiology Team, CESP, Université de Paris Saclay, Villejuif, France; ^5^Service de Médecine Intensive Réanimation, CHU Cavale Blanche, Brest, France; ^6^Dept of Anaesthesiology and Intensive Care, Biomedical and Clinical Sciences, Linköping University, Linköping, SUEDE; ^7^Department of Anesthesiology and Intensive Care, General University Hospital and 1st Medical Faculty, Charles University, Prague, Republique Tcheque; ^8^Department of Anaesthesiology and Critical Care Medicine, Vall d'Hebron University Hospital, Barcelona, Espagne; ^9^CHIREC Hospitals, Université Libre de Bruxelles, Bruxelles, Belgique; ^10^Service de Médecine Intensive Réanimation, Hôpitaux universitaires Henri Mondor, Assistance Publique-Hôpitaux de Paris, Groupe de recherche clinique CARMAS, Inserm U955, Université Paris-Est Créteil, Créteil, France; ^11^Department Clinical Internal, Anesthesiological and Cardiovascular Sciences, University of Rome, "La Sapienza", Policlinico Umberto Primo, Viale del Policlinico, Rome, ITALIE; ^12^Department of Anesthesia and Intensive Care, Policlinico-Vittorio Emanuele University Hospital, Catania, Italia

##### **Correspondence:** Philippe Vignon (philippe.vignon@unilim.fr)

*Annals of Intensive Care *2013, * 13(1): *CO-41

**Rationale:** To evaluate the temporal course of right ventricular (RV) injury during the ICU stay of patients with COVID-19 acute respiratory distress syndrome (ARDS) and its association with mortality, according to currently proposed definitions.

**Patients and methods/materials and methods:** This is a post-hoc analysis of longitudinal data collected in the multicenter ECHO-COVID observational study in patients who underwent at least two echocardiography assessments during ICU stay. Screened RV injuries were acute cor pulmonale (ACP: RV cavity dilatation and paradoxical septal motion), RV failure (RVF: RV cavity dilatation and systemic venous congestion), and RV dysfunction (tricuspid annular plane systolic excursion ≤ 16 mm).

**Results:** Of 281 patients who underwent 948 echocardiography studies during ICU stay, 189 (67%) were diagnosed with at least one type of RV injury during one or several examinations: ACP (37.4%), RVF (54.7%) and/or RV dysfunction (29.0%). Keeping the other RV injuries constant, patients with all examinations displaying ACP had shortened survival time by 0.479 times [0.284–0.803] (P = 0.005) when compared to patients with all examinations depicting no ACP. RVF showed a trend towards shortened survival time (0.642 [0.405–1.018], P = 0.059), whereas RV dysfunction had no significant effect (0.0834 [0.521–1.336], P = 0.451).

**Conclusion:** RV injury is prevalent in patients ventilated for COVID-19 ARDS. Unlike RV dysfunction, the development of ACP during ICU stay was associated with an increased risk of death. RVF showed a trend towards reduced survival time but was not significantly associated with ICU mortality. Determining consensus definitions of different types of RV injury is warranted since their impact on outcome appears variable.

**Compliance with ethics regulations:** N/A.

## CO-42 Prediction of esophagogastroduodenoscopy therapeutic usefulness for in-ICU suspected upper gastrointestinal bleeding

### Victor Penaud^1^, Tomas Urbina^1^, Vincent Bonny^1^, Paul Gabarre^1^, Louai Missri^1^, Jean-Luc Baudel^1^, Nicolas Carbonell^1^, Sayma Chaibi^1^, Aurelia Retbi^1^, Bertrand Guidet^1^, Eric Maury^1^, Hafid Ait-Oufella^1^, Jeremie Joffre^1^

#### ^1^Hôpital Saint-Antoine APHP, Paris, France

##### **Correspondence:** Jeremie Joffre (jeremie.joffre@aphp.fr)

*Annals of Intensive Care *2013, * 13(1): *CO-42

**Rationale:** Suspected upper-gastrointestinal bleeding (SUGIB) is a common issue for critically ill patients during ICU stay. In the absence of specific guidelines regarding the indication and timing of oesogastroduodenoscopy (EGD), there is substantial variability in EGD practice policy between ICUs, depending partly on EGD accessibility. This study aimed to investigate factors associated with the need for per-EGD hemostatic therapy and to develop a point-based score predicting the therapeutic value of emergency bedside EGD in ICU patients presenting a SUGIB.

**Patients and methods/materials and methods:** We conducted a retrospective cohort study in our university hospital ICU, a high-volume and reference center for acute GI hemorrhage in Paris (France), with 24/7 access to EGD. Two hundred fifty-five patients, not primarily admitted for GI bleeding, who underwent a bedside EGD for SUGIB during their ICU stay were analyzed. Clinicals, biologicals, EGD-related data, and outcomes were collected. We followed the TRIPOD Checklist to develop a prediction model relying on a simple point-based score.

**Results:** Amongst the 255 patients who underwent EGD for SUGIB during their ICU stay, the preeminent EGD indication was anemia (79%), melena (19%), shock (14%), and hematemesis (13%). EGD was normal in 24.3%, and the primary lesions reported were ulcers (23.1%), esophagitis (18.8%), and gastritis (12.5%). Only 12.9% of patients underwent hemostatic endotherapy during EGD. Based on predictive factors of the need for hemostasis during EGD, we developed the SUspected GIB in Icu score (SUGIBI): Male gender, smoking, and history of cirrhosis were attributed one point. RRT, shock and/or hematemesis indicating EGD were attributed two points. No external bleeding was associated with minus one point. A SUGIBI score < 4 has a negative predictive value of 97% (0.94 to 0.99) for hemostatic endotherapy and a positive predictive value of 53% (0.39 to 0.66) if ≥ 4 (AUC of the model: 0.82; 0.73–0.9 (P < 0.0001)).

**Conclusion:** Although bedside emergency EGD for SUGIB has an indisputable diagnostic value in critically ill patients, hemostatic therapy is performed in a minority of patients. We propose the SUGIBI score based on simple criteria to help the clinician stratify the probability of a therapeutic EGD. In case of a SUGIBI score < 4, we advocate that EGD might safely be postponed or reconsidered, especially if low accessibility or frail patients are at risk of EGD complications. Conversely, if the SUGIBI score > 4 EGD must be performed without delay, given the high probability of hemostatic endotherapy. This score requires prospective external validation.

**Compliance with ethics regulations:** Yes in clinical research.

## CO-43 Alcoholic hepatitis in intensive care units: clinical and biological spectrum and mortality risk factors: a multicenter study

### Maxime Gasperment^1^, Guillaume Dumas^2^, Côme Gerard^2^, Mialy Randrianarisoa^3^, Luc Haudebourg^4^, Sacha Sarfati^5^, Léa Duhaut^1^, Charlène Le Moal^6^, Gabriel Pedra^7^, Thibault Vieille^8^, Arnaud Galbois^9^, Hafid Ait-Oufella^1^

#### ^1^CHU Saint-Antoine, Paris, France; ^2^CHU de Grenoble-Alpes, Grenoble, France; ^3^CHU Hautepierre, Strasbourg, France; ^4^CHU Pitié-Salpêtrière, Paris, France; ^5^CHU Rouen-Normandie, Rouen, France; ^6^CH du Mans, Le Mans, France; ^7^CH Delafontaine, Saint-Denis, France; ^8^CHU Besançon, Besançon, France; ^9^CH Claude Galien, Quincy-Sous-Sénart, France

##### **Correspondence:** Maxime Gasperment (maxime.gasperment@gmail.com)

*Annals of Intensive Care *2013, * 13(1): *CO-43

**Rationale:** Alcoholic hepatitis (AH) is a clinical syndrome responsible for high morbidity and mortality, but little is known about the management of AH patients admitted in Intensive Care Units (ICUs). Our study aimed to describe the clinical and biological characteristics of these patients and to analyze mortality risk factors.

**Patients and methods/materials and methods:** We conducted a retrospective study of all AH patients hospitalized in 9 French ICUs (Jan 2006–Dec 2017). AH was defined histologically or by an association of clinical and biological criteria according to current guidelines. Data collected included usual clinical and biological features, overall and disease severity, organ failures and patient management.

**Results:** 187 patients (median age: 53 [43;60]; male: 69%) were included. Median Child–Pugh score at admission was 12 [11;13]. Patients were admitted for impaired consciousness (71%), sepsis (64%), shock (44%), respiratory failure (37%) or hemorrhage (8%). At admission, median SOFA, SAPS II and MELD scores were 10 [7;13], 52 [39;74] and 31 [26;40] respectively. During ICU stay, main liver-related complications were encephalopathy (76%), ascites (67%) and hepato-renal syndrome (60%). 62% of patients received vasopressors, 63% invasive mechanical ventilation and 36% renal replacement therapy. 66% received steroids, and liver transplantation was performed in 16 patients (8.5%). ICU death was 37%, in-hospital death 53%. In univariate analysis, ICU mortality was associated with unemployment (p = 0.034), and admission respiratory failure (p < 0.001), shock (p < 0.001), sepsis (p < 0.001), acute kidney injury (p < 0.001), lower Glasgow score (p = 0.006) and higher SOFA (p < 0.001), SAPS II (p < 0.001) and MELD scores (p < 0.001). Need for supportive therapy (vasopressors (p < 0.001), ventilation (p < 0.001), renal (p < 0.001)) was associated with worse outcomes, as well as hepato-renal syndrome (p < 0.001) and encephalopathy (p = 0.002). In multivariate analysis, ICU mortality was associated with admission SOFA score (HR 1.08 [1.02;1.14]), arterial lactate (HR 1.08 [1.03;1.13]) and MELD score (HR 1.09 [1.04;1.14]), while employment was a protective factor (HR 0.49 [0.28;0.86]). Overall, use of steroids did not affect ICU mortality (HR 0.96 [0.48;1.91]); however, significant differences were found between steroids responders and non-responders as assessed by Lille score at day 7 (Lille score > 0.45: OR 6.67 [2.44;20.15], p < 0.001).

**Conclusion:** Alcoholic hepatitis is a severe condition leading to high mortality in ICU patients. Severity of organ failure at admission are mortality risk factors, as well as hepato-renal syndrome during ICU stay. Outcome was significantly worse in non-responders to steroids therapy.

**Compliance with ethics regulations:** Yes in clinical research.

## CO-44 Acute liver failure due to Herpes simplex virus: a retrospective, multicentre, observational study—ALIVE-HSV study

### Thomas Frapard^1^, Giuliana Amaddeo^1^, Maxens Decavele^4^, Paer-Selim Abback^3^, Charlotte Bouzbib^4^, Claire Vanlemmens^5^, Romy Younan^6^, Emmanuel Canet^7^, Anne Sophie Moreau^8^, Benjamin Zuber^9^, Djamel Mokart^11^, Sylvie Radenne^12^, Olivier Roux^13^, Agnes Bonadona^14^, Audrey De Jong^10^, Jerome Dumortier^2^, Nicolas De Prost^1^

#### ^1^Hôpital Henri Mondor APHP, Créteil, France; ^2^Hôpital Edouard Herriot HCL, Lyon, France; ^3^CHRU Tours, Tours, France; ^4^Hôpital Pitié Salpêtrière APHP, Paris, France ; ^5^CHU Besançon, Besançon, France; ^6^Hôpital Saint Louis APHP, Paris, France; ^7^CHU Nantes, Nantes, France ; ^8^CHU Lille, Lille, France ; ^9^Hôpital Foch, Suresnes, France ; ^10^CHU Montpellier, Montpellier, France ; ^11^Institut Paoli-Calmettes, Marseille, France ; ^12^Hôpital de la Croix Rousse HCL, Lyon, France ; ^13^Hôpital Beaujon APHP, Clichy, France ; ^14^CHU Grenoble Alpes, Grenoble, France

##### **Correspondence:** Thomas Frapard (thomas.frapard@aphp.fr)

*Annals of Intensive Care *2013, ** 13(1)***: *CO-44

**Rationale:** Acute liver failure is a rare but life-threatening critical illness. Among its causes, one of the least reported is infection by Herpes Simplex Virus (HSV). This aetiology is evoked in case of febrile hepatic insufficiency with cutaneous signs, in particular in immunocompromised patients. Previous small case series have reported marked transaminase elevation and high mortality. The aim of the current study is to describe the clinico-biological characteristics and prognosis of patients admitted to an intensive care unit for acute hepatitis and proven HSV infection.

**Patients and methods/materials and methods:** This is a retrospective multicenter study conducted in 14 intensive care units in France. All patients hospitalized in participating intensive care units between 2006 and 2022 with a compatible history and a proven HSV infection (PCR blood or tissue) were included.

**Results:** A total of 29 patients were included. The diagnosis was confirmed by histology for 14 (48.3%) patients. There were 3 (10.3%) pregnant patients and 21 (72.4%) immunocompromised patients. Upon ICU admission 27 (93.1%) patients were febrile with a median temperature of 38.7 °C [38.4–39.4], a skin rash was noted in 11 (37.9%) patients, the median SOFA score was 15 [11–17], the median platelet counts was 50 G/L [18–82], the median prothrombin rate (PT) was 35% [28–50], and the median ASAT level was 2497 IU/L [765.75–6304]. The median time interval between the onset of symptoms and the initiation of acyclovir was 8.5 days [5–11]. During ICU stay, 25 (86.2%) patients developed hepatic encephalopathy, 3 (10.3%) developed HSV encephalitis, 4 (13.8%) myocarditis, 10 (34.5%) haemorrhagic shock, 12 (41.4%) disseminated intravascular coagulation (DIC) and 16 (55.2%) a hemophagocytic syndrome. 21 (72.4%) of the patients died in intensive care unit. A liver transplantation was attempted in 4 (13.8%) patients, but the procedure was unsuccessful in all cases (100% mortality). Factors associated with mortality in univariable logistic regression were SAPS II and SOFA scores, temperature, confusion, creatinine at ICU admission, and hepatic encephalopathy, the occurrence of DIC and the nadir of PT during ICU stay.

**Conclusion:** Herpetic hepatitis in the ICU is an extremely severe condition associated with a very high mortality. Despite previously known risk factors (immunosuppression and pregnancy) and a clinico-biological picture associating a febrile hepatitis with a predominance of ASAT, skin rash and cytopenia, the diagnosis remains difficult with a median delay of 8 days until the introduction of aciclovir. The benefit/risk ratio of early initiation of aciclovir in acute febrile hepatitis seems very favourable.

**Reference 1**

Little L, Rule J, Peng L, Gottfried M, Lee WM, Acute Liver Failure Study Group. Herpes Simplex Virus-Associated Acute Liver Failure Often Goes Unrecognized. Hepatology. févr 2019;69(2):917?9.

**Reference 2**

Ichai P, Roque Afonso AM, Sebagh M, Gonzalez ME, Codés L, Azoulay D, et al. Herpes simplex virus-associated acute liver failure: a difficult diagnosis with a poor prognosis. Liver Transpl. déc 2005;11(12):1550?5.

**Compliance with ethics regulations:** Yes in clinical research.

## CO-45 Prognosis Of Liver Abscess in the Intensive caRe unit (POLAIR): a multicentre observational study

### Marie Le Goff^1^, Danielle Reuter^2^, Elsa Moncomble^3^, Megan Fraisse^4^, Elise Yvin^5^, Etienne De Montmollin^6^, Julien Schmidt^7^, Nahema Issa^8^, Laure Calvet^9^, Eric Mariotte^1^

#### ^1^Hôpital Saint-Louis, APHP, Paris, France; ^2^Centre Hospitalier Sud Francilien, Corbeil-Essonnes, France; ^3^Hôpital Henri-Mondor, APHP, Créteil, France; ^4^Hôpital Ambroise-Paré, APHP, Boulogne-Billancourt, France; ^5^CHU Angers, Angers, France; ^6^Hôpital Bichat, APHP, Paris, France; ^7^Hôpital Avicenne, APHP, Bobigny, France; ^8^Hôpital Saint-André, CHU de Bordeaux, Bordeaux, France; ^9^CHU Clermont-Ferrand, Clermont-Ferrand, France

##### **Correspondence:** Marie Le Goff (legoffmarie1@gmail.com)

*Annals of Intensive Care *2013, * 13(1): *CO-45

**Rationale:** Liver abscess (LA) is a rare and potentially life-threatening disease. There is no available data on the epidemiology and prognosis of LA patients requiring intensive care unit (ICU) admission in France.

**Patients and methods/materials and methods:** We conducted a retrospective multicenter study including adults admitted in 27 French intensive care units with a diagnosis of LA from 2010 to 2020. Data are presented as number (%) or median (interquartile range). Risk factors for ICU mortality were identified using univariate analysis.

**Results:** 400 patients met inclusion criteria. In this preliminary report we present the data of 102 patients, included in 9 centers. Patients’ age was 66 years (55–73) and 73% were men. The main causes of admission were sepsis (90%) and respiratory failure (9%). At ICU admission, IGS2 score was 39 (29–53) and SOFA score was 5 [2–9]. The putative origin of LA was biliary (25%), portal (13%), due to pre-existing lesions (16%), arterial (7%) or cryptogenetic (30%). Fifty-eight percent of patients had a solitary LA, the rest having 2 (2–2.25) lesions. LA involved the right part of the liver in 51% of cases, the left in 32% or both in 17%. Larger abscess size was 60 mm [32–90]. Eighty-seven percent of LA were microbiologically documented, with the isolation of gram-negative bacilli (68%), gram-positive cocci (34%) and/or other germs (19%) (details in Figure 1). Multiple pathogens identification occurred in 33 (37%) of patients. Fine needle aspiration of LA was performed in 69 (68%) patients and drain insertion in 47 (46%). Duration of antibiotherapy was 28 days [18–42]. During ICU stay, 24 (24%) patients developed septic shock, 20 (20%) renal failure and 16 (16%) respiratory failure. In-ICU mortality was 11,7%. Factors associated with mortality were organ dysfunctions, illustrated by SOFA score (OR1.184 [1.065–1.316] IC95%, p = 0.0018) and lack of microbial documentation (OR5.347 [1.27–22.489] IC95%, p = 0.022). Abscess evacuation by puncture was associated with better short-term survival (OR4.375 [1.18–16.216] IC95%, p = 0.0272) with no additional benefit of drainage (OR0.2887 [0.034–2.745] IC95%, p = 0.2887).

**Conclusion:** In this multicentric study we described the characteristics and short-term outcome of severe LA in France. Based on our preliminary results, we will analyse the full cohort of 400 patients looking for parameters associated with improved short- and long-term survival.

**Reference 1**

1. Chen W, Chen C-H, Chiu K-L, et al. (2008) Clinical outcome and prognostic factors of patients with pyogenic liver abscess requiring intensive care: Critical Care Medicine 36:1184–1188.

**Reference 2**

2. Kaplan G, Gregson D, Laupland K (2004) Population-based study of the epidemiology of and the risk factors for pyogenic liver abscess. Clinical Gastroenterology and Hepatology 2:1032–1038

**Compliance with ethics regulations:** Yes in clinical research.

**Figure 1 (abstract CO-45):**
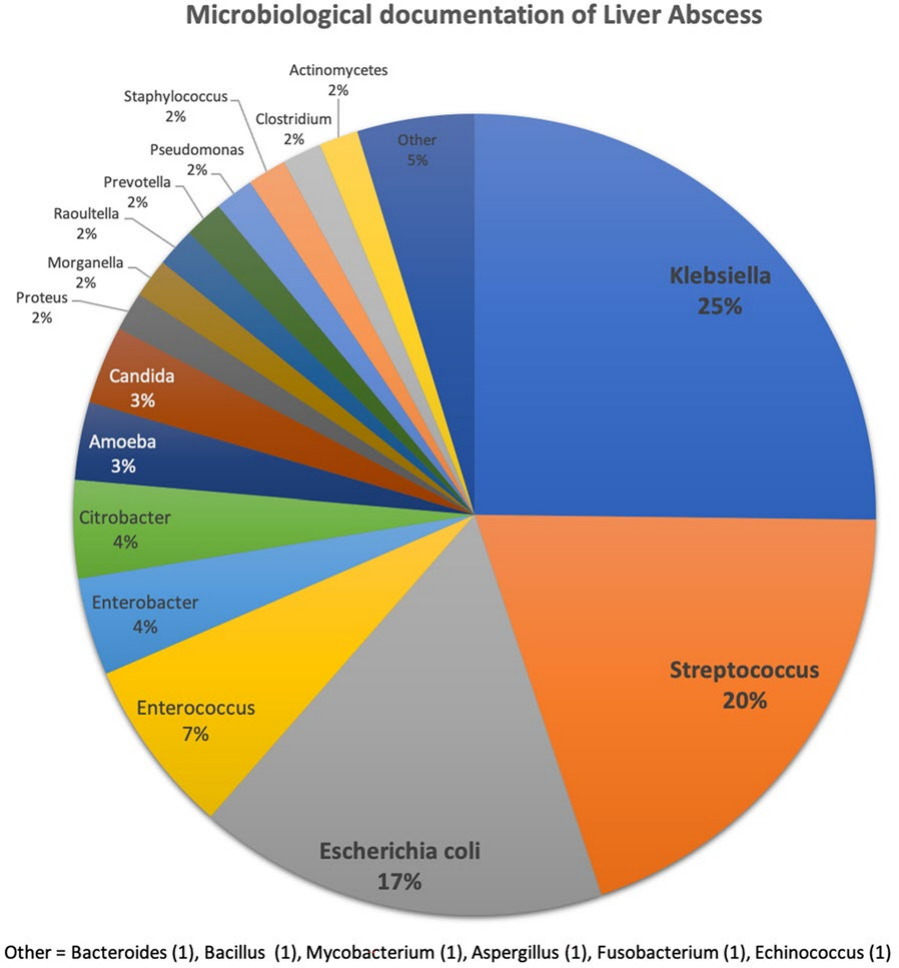
Microbiological documentation of Liver Abscess

## CO-46 Factors associated with mortality among patients with severe acute pancreatitis requiring laparostomy for abdominal compartment syndrome

### Thibault Vieille^1^, Melissa Crotet^1^, Hadrien Winiszewski^1^, Gilles Capellier^1^, Gael Piton^1^

#### ^1^CHU Besançon, Besançon, France

##### **Correspondence:** Thibault Vieille (thibault.vieille91@gmail.com)

*Annals of Intensive Care *2013, * 13(1): *CO-46

**Rationale:** Abdominal compartment syndrome (ACS) may occur during severe acute pancreatitis (SAP) and is associated with a poor outcome. Guidelines provide to perform invasive decompression in patients with a sustained intra-abdominal pressure > 25 mmHg with new onset organ failure refractory to medical therapy. Nevertheless, current criteria triggering surgical decompression are probably too selective, leading to delayed treatment. The primary objective of this retrospective study was to determine predictors associated with 28-day mortality in patients requiring laparostomy for ACS during SAP. Secondary objectives were to evaluate the benefits of surgical decompression on organ failure improvement, and the prevalence of acute mesenteric ischemia.

**Patients and methods/materials and methods:** Monocenter, retrospective, observational study including all adult patients admitted to the ICU for SAP and requiring surgical decompression for abdominal compartment syndrome between January 2006 and March 2022. Comparison of the patients according to the vital status at 28-day.

**Results:** 24 patients with SAP and laparostomy for ACS were included. 28-day mortality rate was 38%. Decompressive laparostomy did not improve organ failure (SOFA score, norepinephrine dose, arterial pH, urine output and Pa02/Fi02 ratio), despite a significant decrease of intra-abdominal pressure. At the surgical decompression time, arterial pH, SOFA score, norepinephrine dose, and urinary output, were significantly associated with 28 day-mortality. We identified 4 criteria associated with a poor evolution when present in the 24 h before laparostomy: arterial pH < 7.25, arterial lactate level > 5 mmol/L, urine output (/24 h) < 0.5 L/day, and norepinephrine > 0.5mcg/kg/min. These criteria were suggestive of the development of non-occlusive mesenteric ischemia (NOMI). Indeed, 2/3 of the non-survivors developed acute mesenteric ischemia.

**Conclusion:** We suggest that during severe acute pancreatitis, practitioners should focus not only on the level of intra-abdominal pressure to consider decompressive laparostomy but should also consider the possibility of NOMI. Indeed, NOMI may develop early during SAP, before the threshold of 25 mmHg of intra-abdominal pressure has been reached. Suspicion of NOMI during severe AP should lead to early decompressive laparotomy, even if the criteria of ACS are not reached, in order to improve overall survival.

**Compliance with ethics regulations:** Yes in clinical research.

**Figure 1 (abstract CO-46):**
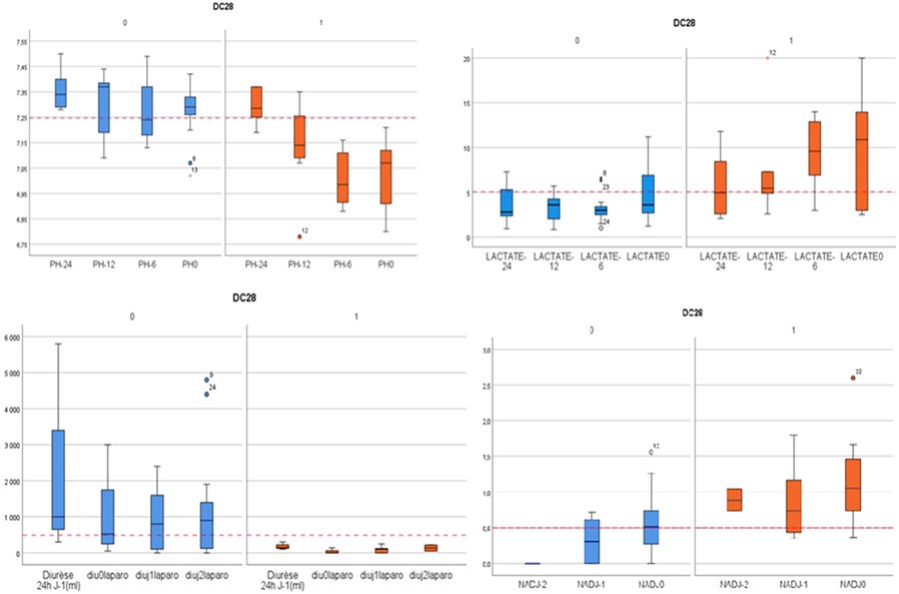
Evolution of pH, arterial lactate, norepinephrine, urinary output during the previous 24 h of decompressive laparostomy according 28-day mortality. “Rule of 5”

## CO-47 Nutritional status and dietetic rehabilitation in weaning unit after prolonged stay in ICU

### Jean-Baptiste Peretout^1^, Maxime Cadoret^1^, Camille Lezzier^1^, Cécile Seron^1^, Gérald Choukroun^1^

#### ^1^Hôpital Forcilles-Fondation Cognacq-Jay, Ferolles-Attilly, France

##### **Correspondence:** Jean-Baptiste Peretout (jperetout@cognacq-jay.fr)

*Annals of Intensive Care *2013, * 13(1): *CO-47

**Rationale:** A recent systematic review revealed the strikingly high prevalence of malnutrition in intensive care unit (ICU) patients (ranged from 38 to 78%)^1^. Enteral feeding through a nasogastric tube is the most common form of nutrition in ICU. Prolonged immobilization and neuromyopathy also lead to malnutrition and sarcopenia^2^. Reinforced multidisciplinary management in the weaning unit makes it possible to monitor and adapt their nutritional needs and facilitate their reautonomization. The objective of this study is to determine the proportion of patients suffering from malnutrition, their clinico-biological characteristics and the effect of rehabilitation on their renutrition capacities.

**Patients and methods/materials and methods:** This is a prospective, descriptive cohort study of patients hospitalized in respiratory weaning unit (WU) after a prolonged ICU stay over the year 2022. All patients hospitalized in the WU were included. Demographic characteristics and nutritional characteristics were collected. All inpatients with complete data were analyzed. All patients experienced active dietetic and speech therapy management.

**Results:** In 2022, 103 patients were admitted in our unit and a total of 97 patients were analyzed. All patients hospitalized in WU were tracheostomized. The main diagnosis at admission in ICU was acute respiratory failure in 58,7%. Characteristics at admission to WU are presented in Table I. 60% of the patients presented malnutrition: moderate 20% and severe 40%. All biological criteria for malnutrition were abnormal: hypoalbumenia, high CRP and low 25(OH)D. At admission, almost all patients had exclusive enteral feeding (92.8%): 74.2% by nasogastric tube and 18,6% by gastrostomy tube. At discharge from WU, 85.6% of patients had resumed oral feeding compared to admission 7.2% (p < 0,001) and only 25.8% of patients still had enteral feeding compared to admission 92.8% (p < 0,001).

**Conclusion:** After a stay in WU, a vast majority of hospitalized patients were able to resume exclusive oral feeding despite a significant proportion of patients with malnutrition. The dietetic management of these patients remains essential and is fully integrated in the multidisciplinary management of patients in WU.

**Reference 1**

1. Lew CCH, Yandell R, Fraser RJL, Chua AP, Chong MFF, Miller M. Association between malnutrition and clinical outcomes in the intensive care unit: a systematic review. JPEN J Parenter Enteral Nutr. 2017;41(5):744–58. https://doi.org/10.1177/0148607115625638

**Reference 2**

2. Hill A, Elke G, Weimann A. Nutrition in the Intensive Care Unit-A Narrative Review. Nutrients. 2021 Aug 19;13(8):2851. https://doi.org/10.3390/nu13082851

**Compliance with ethics regulations:** Yes in clinical research.

**Table 1 (abstract CO-47):**
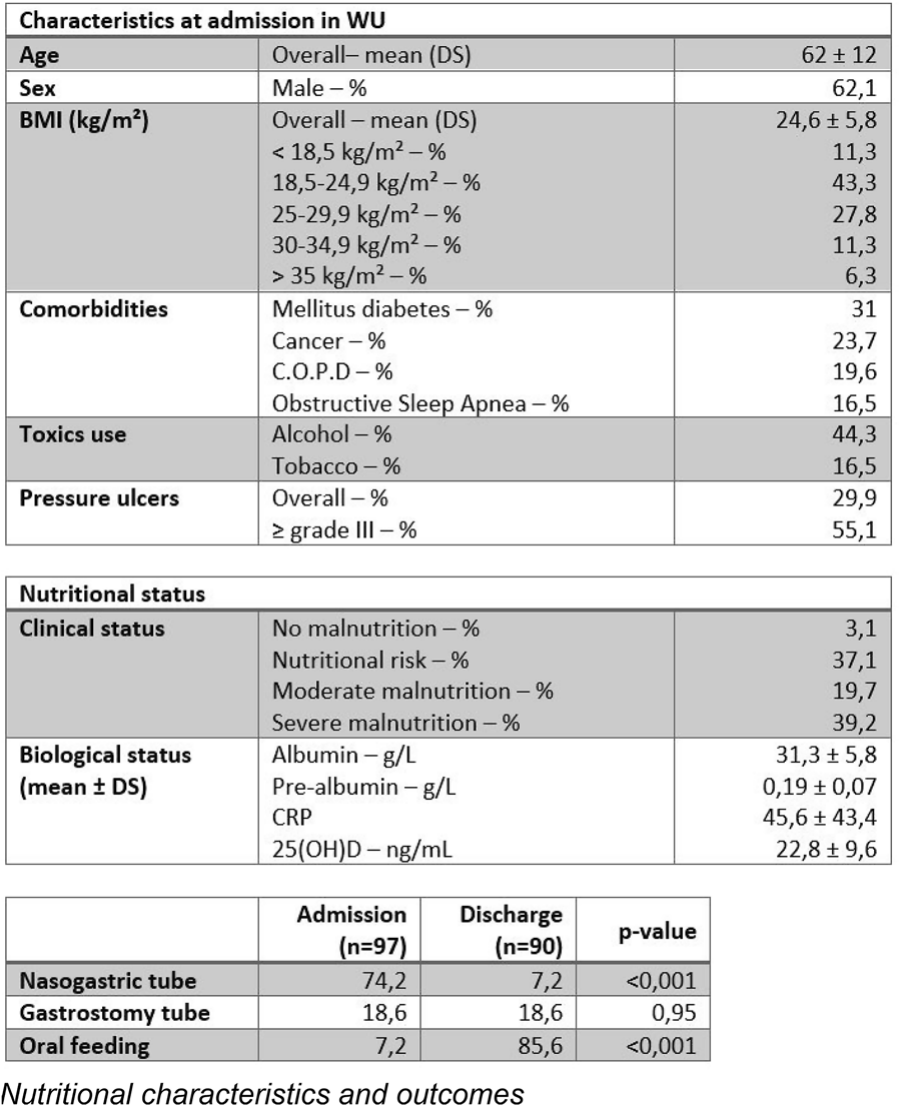
Nutritional characteristics and outcomes

## CO-48 Electrical impedance tomography-derived “optimal” PEEP and recruitment-to-inflation ratio to assess lung recruitability in patients with ARDS on venovenous ECMO

### Alexandre Coppens^1^, Sarah Aissi James^1^, Charles-Edouard Luyt^1^, Guillaume Hekimian^1^, Juliette Chommeloux^1^, Marc Pineton De Chambrun^1^, Alain Combes^1^, Guillaume Franchineau^2^, Matthieu Schmidt^1^

#### ^1^Groupe Hospitalier Pitié–Salpêtrière, Assistance Publique-Hôpitaux de Paris, Paris, France; ^2^Centre Hospitalier Intercommunal de Poissy Saint Germain en Laye, Poissy, France

##### **Correspondence:** Alexandre Coppens (alex95630@yahoo.fr)

*Annals of Intensive Care *2013, * 13(1): *CO-48

**Rationale:** Electrical impedance tomography (EIT) allows for determining “optimal” positive end-expiratory pressure (PEEP) at the bedside by identifying the PEEP level with minimal lung overdistension and collapse [1]. On the other hand, the recruitment-to-inflation ratio (R/I) has been developed to evaluate the potential for lung recruitment in patients with ARDS but has never been studied in patients on ECMO, frequently ventilated with very low tidal volume. The aim of this study was to assess the accuracy of the R/I and to compare its performance with EIT-based optimal PEEP in ECMO-treated patients ventilated with low tidal volume.

**Patients and methods/materials and methods:** Intubated patients with severe ARDS on venovenous ECMO with a tidal volume > 2 ml/kg were included for 9 months. First, a low-flow insufflation was performed to detect airway opening closure. EIT-derived optimal PEEP was determined during a decremental PEEP trial (20 to 6 cmH2O). Similarly, R/I was calculated between a PEEP of 15 to 5 cmH_2_O or the airway opening pressure. The whole cohort was randomly split into two groups. In the first one (regression cohort), the correlation between EIT “optimal” PEEP and R/I was assessed using a mixed model for repeated measures whereas the agreement between the two methods was evaluated by Cohen's kappa method in the second group (validation cohort). If possible, similar measurements were repeated on Day 7 of ECMO.

**Results:** A total of 54 measurements were obtained in 31 patients (median age 47 years, tidal volume 4.7 [range: 3.0–5.8] mL/kg) after a median of 3 days on ECMO. An airway opening pressure of 13.5 [10–14.25] cmH_2_O was found in 6 patients. In the regression cohort (22 patients and 39 measurements), EIT-derived “optimal” PEEP was correlated with R/I with a correlation coefficient of 0.038 (95% IC, 0.021 to 0.055), p < 0.001 (Figure 1). Based on the regression equation, a R/I of 0.47 corresponds to an optimal PEEP of 11 cmH2O. In the validation cohort (8 patients with 13 measurements), R/I showed moderate agreement with EIT “optimal” PEEP (K = 0.70) to classify recruiter and non-recruiter.

**Conclusion:** In patients on ECMO ventilated with very low tidal volume, R/I was correlated with “optimal” PEEP determined by EIT. However, beyond identifying the potential for lung recruitment in that population, R/I value had a moderate agreement with EIT optimal PEEP.

**Reference 1**

Franchineau, G., Bréchot, N., Lebreton, G., Hekimian, G., Nieszkowska, A., Trouillet, J.-L., Leprince, P., Chastre, J., Luyt, C.-E., Combes, A., Schmidt, M., 2017. Bedside Contribution of Electrical Impedance Tomography to Setting Positive End-Expiratory.

**Compliance with ethics regulations:** Yes in clinical research.

**Figure 1 (abstract CO-48):**
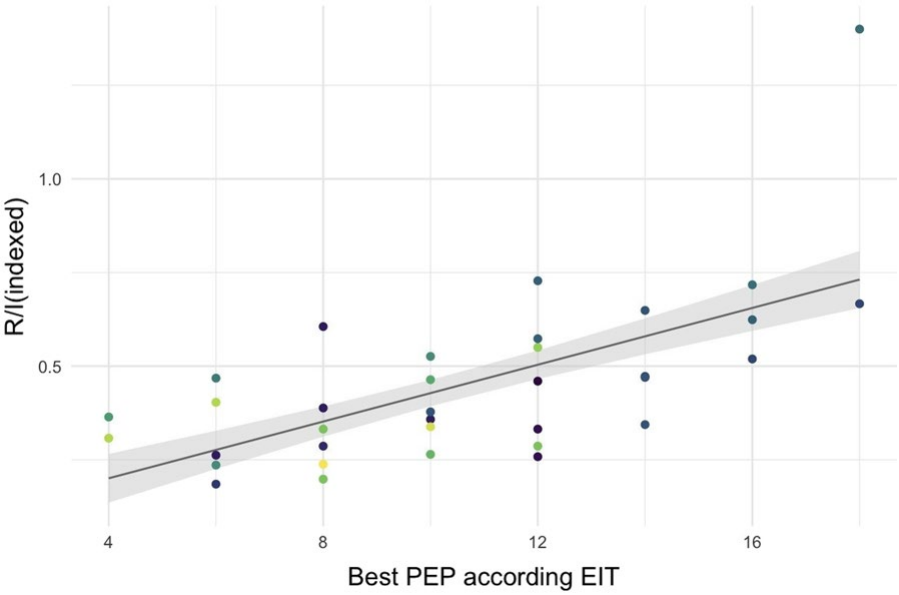
Linear regression between EIT-derived “optimal” PEEP and R/i (from PEEP of 15 to 5 cmH2O). Linear mixed model for repeated measurements. Each patient is represented by a different color. 22 patients and 39 measurements are represented

## CO-49 Impact of dyspnea and diaphragm function in patients presenting with de novo acute hypoxemic respiratory failure

### Sébastien Clerc^1^, Riccardo La Roca^2^, Mélodie Parfait^1^, Vincent Jousselin^1^, Antonin Sieye^1^, Maxens Decavèle ^1^, Julien Mayaux^1^, Alexandre Demoule^1^, Savino Spadaro^2^, Martin Dres^1^

#### ^1^AP-HP. Sorbonne Université, Hôpital Pitié-Salpêtrière, Service de Médecine Intensive-Réanimation (Département "R3S"), Paris, France; ^2^Department of Translational medicine, Intensive Care Unit, University of Ferrara, Sant’Anna Hospital, Ferrara, Italie

##### **Correspondence:** Sebastien Clerc (seb.clerc23@gmail.com)

*Annals of Intensive Care *2013, * 13(1): *CO-49

**Rationale:** The effects of spontaneous breathing efforts during de novo acute respiratory failure (ARF) are debated. While avoiding invasive mechanical ventilation is an important goal, excessive inspiratory efforts and dyspnea may be harmful and associated with worst prognosis, i.e. intubation. This bicentric study sought to investigate dyspnea and diaphragm activity of patients presenting with de novo ARF according to the risk of intubation.

**Patients and methods/materials and methods:** Patients aged of more than 18 years old, hospitalized in the intensive care unit for de novo ARF (respiratory rate > 25/min and hypoxemia defined by PaO2/FiO2 ratio < 300mHg with face mask oxygen ≥ 10L/min O2 or high flow nasal oxygen with FiO2 ≥ 50%) were included. Dyspnea was evaluated with a visual analogic scale (VAS) at inclusion and at H2 and H4. Right ultrasound evaluation of diaphragmatic activity (excursion and thickening fraction) was also performed at inclusion and at H2 and H4. Primary outcome was the intubation rate censored at 7 days after enrolment.

**Results:** Forty-nine patients were included over an 18 months period. Patients were mostly men (69%) with a median age of 66 years. Main causes of ARF were COVID-19 (49%) and post-operative ARF (32%). Thirty patients (61%) were treated with high flow nasal oxygen and in total 13 (27%) were intubated. Median (25–75 IQR) dyspnea was 21 mm (10–40) at inclusion. Dyspnea VAS was 33 mm vs 15 mm (p = 0.11) at inclusion, 41 mm vs 21 mm (p = 0.10) at H2 and 31 mm vs 16 mm at H4 (p = 0.07), in intubated and non-intubated patients respectively. The thickening fraction at inclusion was significantly lower in intubated patients (11% vs 30%; p = 0.003) and H4 (8% vs 24%; p = 0.027). The difference was not significative at H2. The diaphragm excursion was 14 mm vs 13 mm (p = 0.69) at inclusion, 15 mm vs 13 mm (p = 0.94) and 12 mm vs 13 mm (p = 0.65), in intubated and non-intubated patients respectively.

**Conclusion:** These preliminary findings show that dyspnea and lower diaphragm thickening fraction might be associated with the risk of intubation in de novo ARF.

**Compliance with ethics regulations:** Yes in clinical research.

## CO-50 Diaphragm neurostimulation-assisted ventilation in ARDS: a proof-of-concept human study

### Mélodie Parfait^1,2^, Elisabeth Rohrs^3^, Julien Mayaux^1,2^, Thomas Similowski^1,2^, Alexandre Demoule^1,2^, Martin Dres^1,2^

#### ^1^Sorbonne Université, INSERM, UMRS1158 Neurophysiologie respiratoire expérimentale et clinique, Paris, France; ^2^AP-HP. Sorbonne Université, Hôpital Pitié-Salpêtrière, Service de Médecine Intensive-Réanimation (Département "R3S"), Paris, France; ^3^RCH Advancing Innovation in Medicine Institute, New Westminster, Canada

##### **Correspondence:** Mélodie Parfait (melodie.parfait@gmail.com)

*Annals of Intensive Care *2013, * 13(1): *CO-50

**Rationale:** While being life-saving, positive-pressure ventilation (PPV) is associated with harmful effects that carry morbidity and mortality in patients with acute respiratory distress syndrome (ARDS). Reducing the amount of pressure delivered by the ventilator inside the thorax may reduce the development of ventilator-induced lung injury and drop in cardiac output. Diaphragm neurostimulation-assisted ventilation (DiSAV) could overcome these issues.

**Patients and methods/materials and methods:** Patients with moderate ARDS were included after 48 h of invasive mechanical ventilation and were equipped with a left subclavian dedicated catheter which was used to deliver phrenic nerve stimulation. Diaphragm neurostimulation was delivered continually, in synchrony with volume-controlled mode ventilation (DiSAV) during two 60-min sessions, interspersed with two 60-min washout sessions (PPV, no stimulation). Lung mechanics were continuously monitored with esophageal and gastric pressures. Regional lung ventilation was continuously monitored with electrical impedance tomography. Cardiac index was continuously monitored with a transpulmonary thermodilution system. Plateau pressure, driving pressure, transdiaphragmatic pressure, dorsal/ventral ventilation surface ratio and cardiac index were compared between DiSAV and PPV.

**Results:** Thirteen patients were included. Phrenic stimulation was associated with an increase in transdiaphragmatic pressure to 10 cmH2O [8–11] while it was 2 cmH2O [1–4] during PPV (p = 0.003). During stimulation sessions driving pressure was 11 cmH2O while it was 14 cmH2O without stimulation (p < 0.001). Lung compliance improved with a median gain of 35% (p < 0.001) during stimulation sessions. There was also an improvement in ventilation distribution to dorsal areas during DiSAV. Finally, the cardiac index went from 2.7 L/min/m2 [2.3–3.5] without stimulation to 3.3 L/min/m2 [2.5–3.9] during stimulation sessions (p = 0.14).

**Conclusion:** This pilot study shows that transvenous phrenic stimulation during ARDS 1) is feasible, 2) is associated with an improvement in ventilatory mechanics (increase in compliance) and more homogeneous regional ventilation distribution, suggesting a more protective mechanical ventilation.

**Compliance with ethics regulations:** Yes in clinical research.

## CO-51 Attributable mortality and cause of death in hyperinflammatory and hypoinflammatory phenotypes of ARDS and Sepsis

### Bruno Evrard^1^, Pratik Sinha^2,3^, Kevin Delucchi^4^, Carolyn M. Hendrickson^5^, Kirsten N. Kangelaris^6^, Kathleen D. Liu^7,8^, Nelson Wu^7^, Antonio Gomez^5^, Michael A. Matthay^1,8,9^, Lorraine B. Ware^10,11^, Carolyn S. Calfee^1,8,9^

#### ^1^Division of Pulmonary, Critical Care, Allergy and Sleep Medicine, Department of Medicine, University of California San Francisco, San Francisco, Ca, ETATS-UNIS; ^2^Division of Clinical and Translational Research, Washington University School of Medicine, Saint-Louis, Mo, ETATS-UNIS; ^3^Department of Anesthesia, Division of Critical Care, Washington University, Saint Louis, Mo, ETATS-UNIS; ^4^Department of Psychiatry and Behavioral Sciences, University of California San Francisco, San Francisco, Ca, ETATS-UNIS; ^5^Division of Allergy, Pulmonary, and Critical Care Medicine, Department of Medicine, Zuckerberg San Francisco General Hospital and Trauma Center, San Francisco, Ca, ETATS-UNIS; ^6^Division of Hospital Medicine, Department of Medicine, University of California San Francisco, San Francisco, Ca, ETATS-UNIS; ^7^Division of Nephrology, Department of Medicine, University of California San Francisco, San Francisco, Ca, ETATS-UNIS; ^8^Department of Anesthesia, University of California San Francisco, San Francisco, Ca, ETATS-UNIS; ^9^Cardiovascular Research Institute, University of California San Francisco, San Francisco, Ca, ETATS-UNIS; ^10^Division of Allergy, Pulmonary, and Critical Care Medicine, Department of Medicine, Vanderbilt University Medical Center, Nashville, Tn, ETATS-UNIS; ^11^Department of Pathology, Microbiology and Immunology, Vanderbilt University Medical Center, Nashville, Tn, ETATS-UNIS

##### **Correspondence:** Bruno Evrard (bruno.evrard@chu-limoges.fr)

*Annals of Intensive Care *2013, * 13(1): *CO-51

**Rationale:** Previous studies of patients with Acute Respiratory Distress Syndrome (ARDS) and sepsis have identified two phenotypes: the more prevalent but less well understood hypoinflammatory phenotype, and the hyperinflammatory phenotype which is characterized by elevated levels of inflammatory biomarkers. We aimed (i) to mathematically estimate the attributable mortality from ARDS in hypo-inflammatory and hyper-inflammatory sepsis; and (ii) to determine via chart review the primary cause of death within each phenotype.

**Patients and methods/materials and methods:** We studied 1816 septic patients from two prospectively enrolled critically ill adult cohorts in United States of America (EARLI and VALID studies) who were previously assigned to the hyperinflammatory or hypoinflammatory phenotype using Latent Class Analysis (1). Population attributable fraction (AF_ARDS_) of mortality from hypoinflammatory and hyperinflammatory ARDS were mathematically estimated adjusting for illness severity using APACHE II stratification (2), with the comparator groups being hypoinflammatory and hyperinflammatory sepsis, respectively. Organ dysfunction, severe comorbidities, and withdrawal of life support were collected from the medical record when available on patients who died from the EARLI cohort (n = 129/180) by personnel blinded to ARDS status and phenotype, using a standardized instrument (Stapleton RD, CHEST 2005). The primary cause of death was then defined as the organ system that most directly contributed to death or withdrawal of life support.

**Results:** Among 1253 (69%) hypoinflammatory patients, 443 (35%) developed ARDS, while among the 563 (31%) hyperinflammatory patients, 258 (46%) developed ARDS. In the hypoinflammatory phenotype, the AF_ARDS_ was 19% (95%CI: 10–27%). In the hyperinflammatory group, the AF_ARDS_ was 13% (95%CI: 5–21%). In the 129 patients who died with medical records available for review, 34% died without any severe underlying comorbidities in the hyperinflammatory phenotype, versus 20% in the hypoinflammatory phenotype (p = 0.01) (Figure). Cause of death as determined by review of the medical records differed between the two phenotypes (p < 0.001). Respiratory failure (including failure to wean from mechanical ventilation) was the most common cause of death in hypoinflammatory ARDS (63%), whereas circulatory failure was the most common cause of death (65%) in hyperinflammatory ARDS (Figure). These proportions were similar in patients with hypoinflammatory and hyperinflammatory sepsis.

**Conclusion:** Attributable mortality of ARDS remains higher in the hypoinflammatory phenotype of ARDS, with a high proportion of patients dying primarily from respiratory failure. Conversely, attributable mortality of ARDS was lower in the hyperinflammatory phenotype, with more patients dying from circulatory failure. Further studies of the hypoinflammatory phenotype are needed to determine the factors associated with mortality.

**Reference 1**

Sinha P, He J, Delucchi K, Zhuo H, Abbott J, Jones C, et al. Latent Class Analysis-Derived Hypoinflammatory and Hyperinflammatory Phenotypes Are Generalisable to Sepsis Patients Requiring Intensive Care. B95 ARDS: WHAT’S THE LATEST AND GREATEST? American.

**Reference 2**

Auriemma CL, Zhuo H, Delucchi K, Deiss T, Liu T, Jauregui A, et al. Acute respiratory distress syndrome-attributable mortality in critically ill patients with sepsis. Intensive Care Med 2020;46:1222–1231.

**Compliance with ethics regulations:** Yes in clinical research.

**Figure 1 (abstract CO-51):**
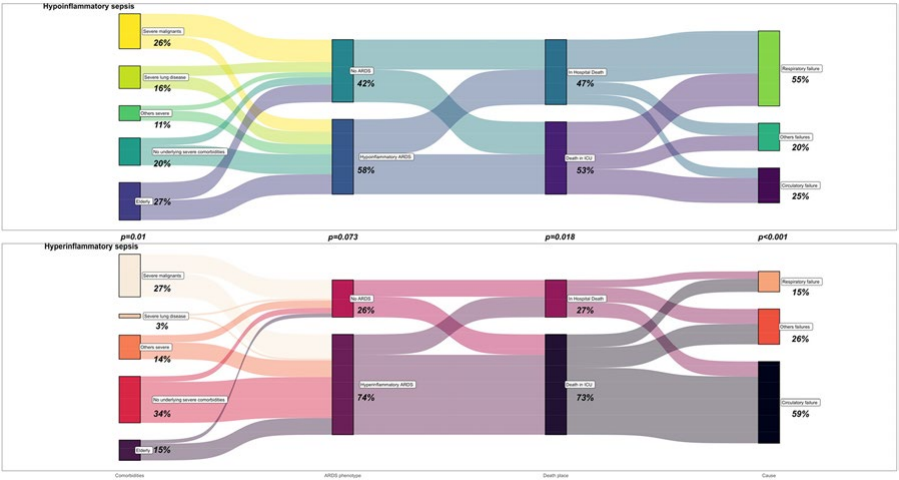
Sankey charts showing the relation between the severe comorbidities, ARDS phenotype, and the final cause of death for the subset of patients who died with hypo or hyperinflammatory sepsis. P values for comparison between phenotypes resulted from Chi2

## CO-52 Interobserver agreement between chest X-ray and computed tomography scan for acute respiratory distress syndrome diagnosis

### Maïa Joos^1^, Guillaume Herpe^2,3^, Jean-Pierre Frat^1,4^, François Arrivé^1^, Anne Veinstein^1^, Delphine Châtellier^1^, Florence Boissier^1,4^, Sylvain Le Pape^1^, Chloé Villaret^2^, Arnaud W. Thille^1,4^, Rémi Coudroy^1,4^

#### ^1^Service de Médecine Intensive Réanimation, Poitiers, France; ^2^Service de Radiologie, Poitiers, France; ^3^UMR 7348, DACTIM-MIS, Université de Poitiers, Poitiers, France; ^4^INSERM CIC 1402, IS-ALIVE research group, Université de Poitiers, Poitiers, France

##### **Correspondence:** Maïa Joos (mjoos@hotmail.fr)

*Annals of Intensive Care *2013, * 13(1): *CO-52

**Rationale:** According to the Berlin definition of acute respiratory distress syndrome (ARDS), bilateral infiltrates can be diagnosed using chest X-ray or computed tomography (CT)-scan. However, the interindividual agreement for the diagnostic of lung infiltrates on chest X-ray is poor and may explain the low rate of ARDS recognition by clinicians. Indeed, as compared to CT-scan, the accuracy of chest X-ray interpreted by a radiologist to identify ARDS was limited. However, in daily practice, chest X-rays are interpreted at the bedside by intensivists, not radiologists. We hypothesized that the agreement between chest X-ray interpreted by an intensivist and CT-scan interpreted by a radiologist, as reference standard for ARDS diagnosis, was poor.

**Patients and methods/materials and methods:** From January 2015 to December 2021, we included all CT-scan performed in patients invasively ventilated with PaO_2_/FiO_2_ ratio ≤ 300 mmHg with positive end-expiratory pressure ≥ 5 cmH_2_O on the day of the CT-scan and a chest X-ray performed within the 24 h around the CT-scan. Patients whose hypoxemia was related to cardiogenic pulmonary edema were excluded. CT-scans and chest X-rays were interpreted semi-quantitatively by a senior radiologist and by a senior intensivist, respectively. The primary outcome was the agreement between chest X-ray and CT-scan for ARDS diagnosis using Cohen’s kappa. Secondary outcomes were the sensitivity of chest X-ray for ARDS diagnosis in the overall population, and in the subgroup of focal ARDS, defined as infiltrates in the lower lobes on CT-scan.

**Results:** Among the 1015 CT-scan performed during the study period, 332 were included in the analysis. Mean age of patients was 62, 74% were males, and 29% were immunocompromised. In-ICU mortality was 41%. CT-scans were performed 3 [1–11] days from intubation. The day of the CT-scan, mean PaO_2_/FiO_2_ ratio was 165 mmHg. The prevalence of ARDS on CT-scan was 82%. Consolidations were mostly found in inferior lobes whereas ground glass was found evenly in all lobes. The agreement between chest X-ray and CT-scan for ARDS diagnosis was moderate (Cohen’s kappa 0.41 [95% confidence interval 0.30–0.51]). Sensitivity of chest X-ray for ARDS diagnosis in the overall population was 77% (95%CI 71–82) and 54% (95%CI 40–67) in the subgroup of focal ARDS.

**Conclusion:** Chest X-ray interpreted by an intensivist and CT-scan interpreted by a radiologist had moderate agreement for ARDS diagnosis. The performance of chest X-ray was particularly poor in focal ARDS. Systematic CT-scan assessment in hypoxemic patients may improve the likelihood of ARDS diagnosis.

**Compliance with ethics regulations:** Yes in clinical research.

## CO-53 Paradoxical response to continuous anterior chest compression may unveil occult overdistension during protective ventilation

### Elsa Moncomble^1^, Samuel Tuffet^1^, Mohamed Boujelben^1^, Anne Fleur Haudebourg^1^, Pascale Labedade^1^, Keyvan Razazi^1^, Nicolas De Prost^1^, Armand Mekontso Dessap^1^, Guillaume Carteaux^1^

#### ^1^Hôpital Henri Mondor, Créteil, France

##### **Correspondence:** Elsa Moncomble (elsa.moncomble@aphp.fr)

*Annals of Intensive Care *2013, * 13(1): *CO-53

**Rationale:** Recent reports in patients with prolonged acute respiratory distress syndrome (ARDS) have described a paradoxical decrease in plateau pressure (Pplat) and increase in respiratory system compliance (Crs) in response to continuous anterior chest compression (CACC). This effect could be explained by the decrease in overdistension due to the decrease in end-expiratory lung volume. We hypothesized that in the early phase of ARDS, a paradoxical response to CACC may unveil overdistension during protective ventilation.

**Patients and methods/materials and methods:** Patients with moderate-to-severe ARDS were enrolled in a single-center study. The tidal volume was set to 6 mL/kg of predicted body weight. Positive end-expiratory pressure (PEEP) was initially set so that the Pplat reached 28 to 30 cmH20 (High PEEP). A CACC of 80 cmH_2_0 was obtained by compression of a 1 L saline bag opposite the sternum. Respiratory mechanics was assessed at High PEEP, before and during CACC. Subsequently, a decremental PEEP trial from PEEP 20 to 4 cmH20 (with steps of 2 cmH_2_0) was performed while recording electrical impedance tomography (Enlight 800, Timpel), which allowed estimation of the percentage of overdistended lung at each PEEP level. PEEP was then set to the highest level that minimized overdistension, defined as the first level with < 1% of overdistended lung (Low PEEP). Finally, respiratory mechanics was assessed at Low PEEP, before and during CACC.

**Results:** 20 patients were included, 16 males and four females, aged 63 [42–65] years, intubated for 1 [0–2] days at the time of enrollment. The etiology of ARDS was pneumonia in 15 (75%) patients. At High PEEP (16 cmH_2_O [16–18]), PaO_2_/FiO_2_ was 123 [93–172] mmHg and Crs was 38 [33–44] ml.cmH_2_O^−1^. When CACC was applied, Pplat increased in only two (10%) patients (delta Pplat: + 1.6 and + 2 cmH_2_0). These two patients had a percentage of overdistended lung < 1%. A paradoxical response was observed in the remaining 18 (90%) patients (delta Pplat:—1 cmH_2_0 [-2–1]). These patients had a percentage of overdistended lung of 25% [15–34], significantly higher than their counterpart (p = 0.01). At Low PEEP (8 cmH_2_O [6–10]), when CACC was applied, Pplat did not decrease in 19 (95%) patients (delta Pplat: + 1.8 cmH_2_0 [1.6–2]) and decreased in only one (Figure 1).

**Conclusion:** The paradoxical response to CACC may unveil “occult” overdistension in moderate-to-severe ARDS during protective ventilation. Whether the response to CACC could help to customize ventilator settings deserves further investigation.

**Compliance with ethics regulations:** Yes in clinical research.

**Figure 1 (abstract CO-53):**
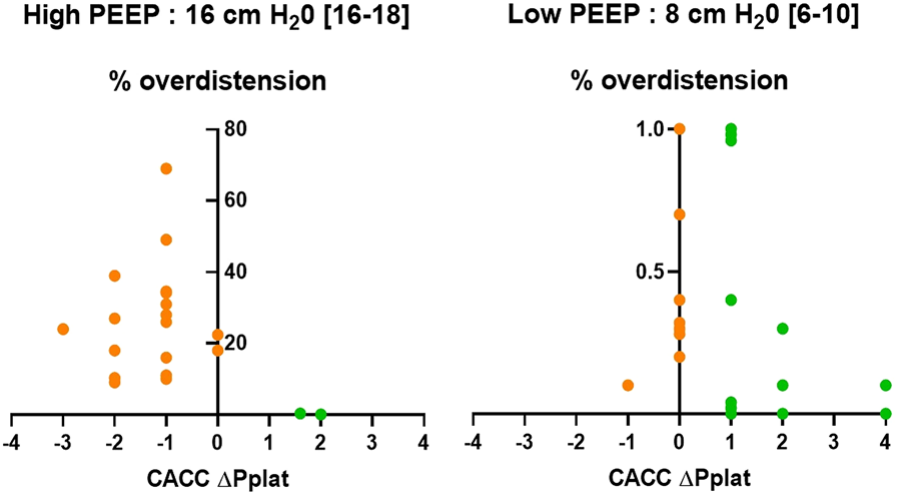
Percentage of lung overdistension (y-axis) by change in Pplat (x-axis) at high and low PEEP. Note that scale of the y-axis is different between the two panels

## CO-54 Impact of implementing a checklist specific to antibiotic prescription in intensive care unit

### Marion Giry^1^, Déborah Boyer^1^, Kévin Alexandre^1^, Christian Caillard^2^, Dorothée Carpentier^1^, Zoé Demailly^1^, Diane Ducloux^3^, Grégoire Jolly^1^, Christophe Girault^1^, Maximilien Grall^1^, Jonathan Nicolas^1^, Benjamin Popoff^1^, Sacha Sarfati^4^, Amandine Verbeke^1^, Fabienne Tamion^1^, Gaëtan Beduneau^1^

#### ^1^CHU de Rouen, Rouen, France; ^2^CHI d'Elbeuf, Elbeuf, France; ^3^GH du Havre, Le Havre, France; ^4^Institut Pasteur, Paris, France

##### **Correspondence:** Marion Giry (marion.giry7@gmail.com)

*Annals of Intensive Care *2013, * 13(1): *CO-54

**Rationale:** The increase in antimicrobial resistance (AMR) is a major public health issue. Antibiotic stewardship programs (ASP) are being developed to curb this phenomenon. Implementing ASP in the intensive care unit (ICU) is an acute topic given that AMR is common and antibiotic consumption is the highest in these departments. Different resources are available for ASP (prospective audit and feedback, referees, antibiotic time-out, etc.). Among them, checklists developed in different fields are interesting tools that can be used in the ICU. This study aimed to assess the impact of a checklist for prescribing antibiotics in the ICU.

Patients and methods/materials and methods: We conducted a monocenter before-after study in the medical ICU of a French tertiary hospital. We analyzed patients admitted to the ICU who received non-prophylactic antibiotic therapy for more than 48 h over periods of nine months before and after the implementation of the checklist (from June 2020 to February 2021 and from June 2021 to February 2022, respectively). The checklist consisted of a paper sheet compiling all data related to antibiotic prescriptions daily filled by physicians or residents. We evaluated the rate of appropriate antibiotic therapy.

Results: Among the 385 patients included (239 for the baseline period and 146 for the intervention period), there was no statistically significant difference in the percentage of appropriate antibiotic therapy between the two periods (23% versus 22%, p = 0.9). There was a significant increase in the rate of appropriate dosing (81% vs. 91%, p = 0.006), in the rate of appropriate antibiotic therapy duration (59% vs. 69%, p = 0.037), and in the performance of therapeutic drug monitoring by increasing the performance of antibiotic blood measures (31% vs. 49%, p < 0.001). These improvements were achieved without any negative impact on mortality (19% vs. 27%, p = 0.062) or length of stay in the ICU (24 days vs. 21 days, p = 0.063). Also, a decrease in the ICU antibiotic costs (€113,667 for the baseline period versus €102,266 for the intervention period) and a decrease in antibiotic use in ICU were observed after the checklist implementation (1,350 versus 1,271 Daily Defines Dose/1,000 patients days).

Conclusion: The introduction of a specific checklist for antibiotics in the ICU was associated with an improvement in quality markers for antibiotic prescription without impacting the safety of patients hospitalized in the ICU. To our knowledge, this was the first time a checklist specific to antibiotic prescription has been evaluated in an adult ICU and its encouraging results show the importance of developing such ASP.

**Compliance with ethics regulations:** Yes in clinical research.

## CO-55 Towards Optimization of Ceftazidime Dosing in Intensive Care Unit Obese Patients: the end of the “one-size-fits-all” approach?

### Patricia Correia^1^, Manon Launay^1^, Rémi Balluet^1^, Laurent Gergelé^2^, Vincent Gauthier^3^, Morel Jérôme^1^, Pascal Beuret^4^, Christophe Mariat^1^, Guillaume Thiery^1^, Sophie Perinel-Ragey^1^

#### ^1^CHU de Saint Etienne, Saint Etienne, France; ^2^Hôpital privé de la Loire, Saint Etienne, France; ^3^Clinique Mutualiste, Saint Etienne, France; ^4^CHR de Roanne, Roanne, France

##### **Correspondence:** Manon Launay (manon.launay@chu-st-etienne.fr)

*Annals of Intensive Care *2013, * 13(1): *CO-55

**Rationale:** Ceftazidime (CAZ) is commonly used as a pivotal antibiotic against *Pseudomonas aeruginosa* in critically ill patients. ICU patients have severely altered and variable antibiotic pharmacokinetics, resulting in lower antimicrobial concentrations and potentially poor outcomes. Several factors, including obesity and renal function, may influence pharmacokinetics. Thus, the objective of the study was to evaluate the impact of obesity and renal function on CAZ plasma concentrations and dosing regimens in ICU patients.

**Patients and methods/materials and methods:** All consecutive adult patients from 6 ICUs, treated with continuous CAZ infusion and under Therapeutic Drug Monitoring evaluation were included. Obesity was defined as body mass index ≥ 30 kg/m^2^. Glomerular filtration rate (GFR) was estimated by Chronic Kidney Disease Epidemiology Collaboration formula. CAZ recommended levels for plasma concentrations were between 35 and 80 mg/L.

**Results:** A total of 111 patients (45 obese), weighted 90 (± 24.4) kg, were included. Mean GFR was 82 mL/min/1.73m2 (± 40.3). Recommended CAZ plasma concentrations were achieved only for 49.6% of patients, with a median dosing regimen of 6 g/d. Obese patients had lower CAZ plasma concentrations compared to non-obese patients (37.8 vs 56.3 mg/L; p = 0.0042*) despite similar dosing regimens (5.83 g/d vs 5.52 g/d, p = 0.2529). Almost all Augmented Renal Clearance patients were underdosed despite CAZ dosing of 6.6 g/d (± 0.8).

**Conclusion:** ICU obese patients required significantly greater CAZ amount to achieve the target range. A ailored dosing regimen may be considered based on weight and GFR. Future prospective studies should be performed to confirm this individualized dosing approach.

**Compliance with ethics regulations:** Yes in clinical research.

**Figure 1 (abstract CO-55):**
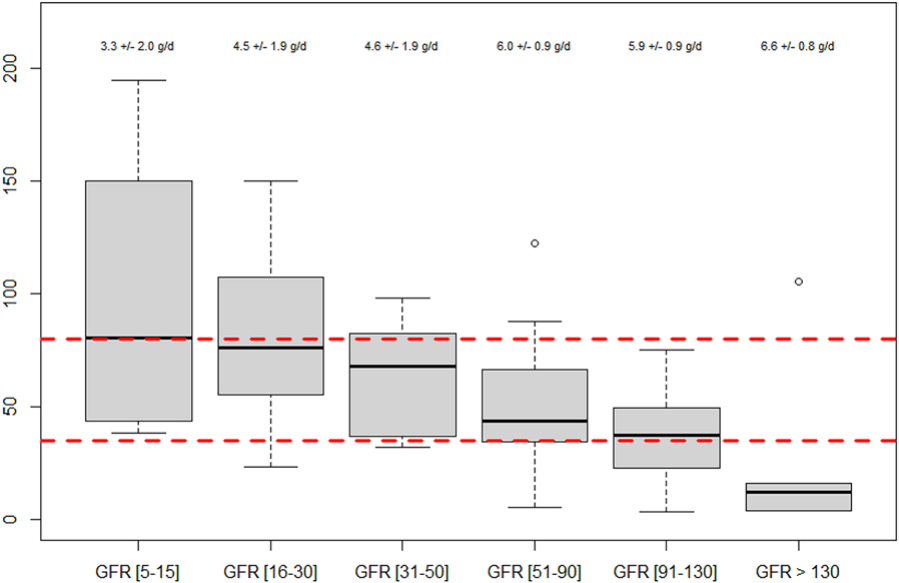
Box plot showing the difference in mean ceftazidime (CAZ) concentrations according to CKD-EPI classes. Target CAZ concentration are shown within the red dashed lines. The mean ± standard deviation of the daily amounts of CAZ are shown above the boxplot

## CO-56 Aggressive versus restrictive initiation of antimicrobial treatment in critically ill patients with suspected ventilator acquired pneumonia without severity: a before/after observational study

### Maëlle Martin^1^, Solène Forveille^1^, Aurélie Le Thuaut^1^, Jean-Baptiste Lascarrou^1^, Amélie Seguin^1^, Emmanuel Canet^1^, Jérémie Lemarie^1^, Maïté Lacou Agbakou^1^, Luc Desmedt^1^, Gauthier Blonz^1^, Olivier Zambon^1^, Jean Reignier^1^

#### ^1^CHU de Nantes, Nantes, France

##### **Correspondence:** Maëlle Martin (maellemart1@gmail.com)

*Annals of Intensive Care *2013, * 13(1): *CO-56

**Rationale:** Ventilator-associated pneumonia (VAP) is the leading nosocomial infection in intensive care and is associated with prolonged mechanical ventilation (MV) and greater antibiotic use. When VAP is suspected, starting antibiotic therapy (AT) immediately after pulmonary sampling may expose uninfected patients to unnecessary treatment, whereas waiting for bacteriological confirmation may delay AT in infected patients. In absence of robust data, VAP suspicion management remains at the physicians’ discretion, balancing diagnostic probability, patient risks, and risk of selecting resistant bacteria. The aim of this study is to compare an immediate AT (aggressive strategy) to a delayed AT (restrictive strategy) in patients with suspected non-severe VAP, in terms of antibiotic sparing and morbi-mortality.

**Patients and methods/materials and methods:** We conducted a 2-year retro-prospective before/after monocentric study, comparing an aggressive to a restrictive strategy of VAP suspicion management. All patients under MV longer than 48 h, pulmonary sampled for a first VAP suspicion, were included, excepted in case of clinical severity (Onset/worsening shock or hypoxemia with PaO2/FiO2 < 150 or neutropenia < 1G/L) or ongoing AT predicted ≥ 4 weeks. In the aggressive period (2019), AT was started immediately after distal lung sampling was performed to assess VAP. In the restrictive period (2022), no AT was given before cultures results were available. If cultures were positive, probabilistic AT was begun with subsequent adaptation to antibiotic susceptibility for a total of 7 days. We compared the number of days alive without AT censored at day 28, ventilator-free days (VFD), and Day-28 mortality.

**Results:** Respectively 44 and 43 patients were included in the aggressive and restrictive phases. Compared to an aggressive strategy, a restrictive strategy was not associated with a reduction in AT consumption (12.8 ± 9.1 days alive without AT over 28 days vs. 13.4 ± 10.2 days, p = 0.58), including no reduction in broad-spectrum AT (17.9 ± 11.2 days vs. 17.6 ± 12.3 days, p = 0.66). There was no significant difference in MV duration (13.1 ± 11.1 VFD in the restrictive group vs 12.8 ± 11.2, p = 0.92) nor in Day-28 mortality (HR = 0.96 IC95% [0.42–2.23] p = 0.93). Treatment was significantly more appropriate in the restrictive than aggressive strategy (38/43 [88.4%] vs 30/44 [68.2%] patients, p = 0.02).

**Conclusion:** In the setting of suspected VAP without severity, delaying AT to a confirmed VAP, compared to an immediate AT, was not associated with morbidity or mortality. Nonetheless, it was not associated with antibiotic sparing (including broad spectrum), even if treatment was significantly more appropriate.

**Compliance with ethics regulations:** Yes in clinical research.

**Figure 1 (abstract CO-56):**
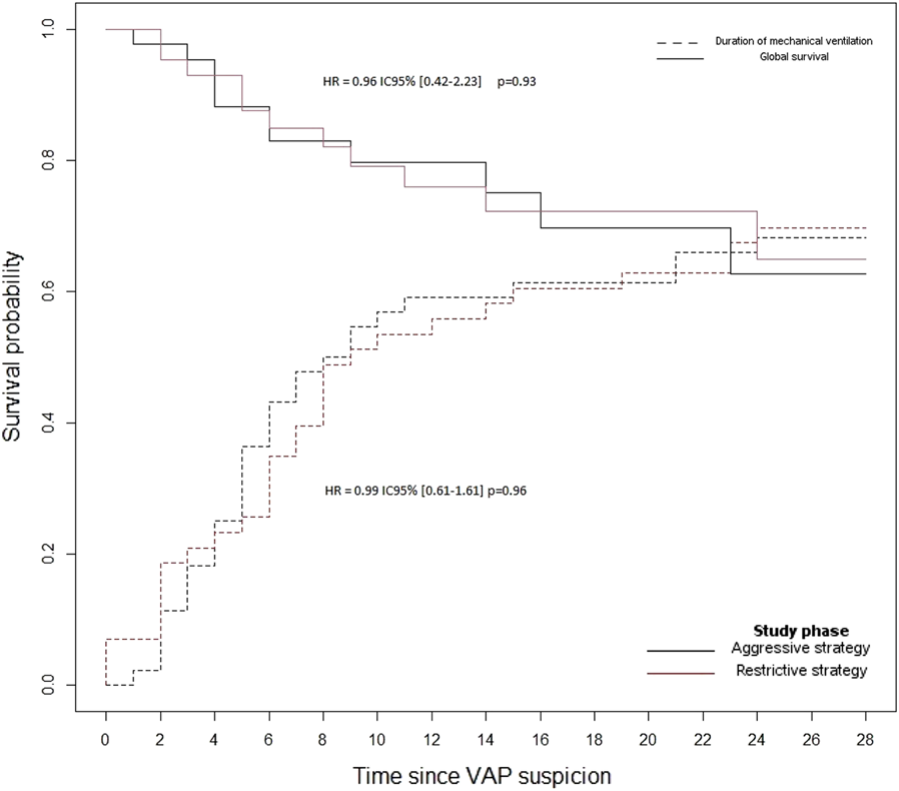
Survival analysis: Duration of mechanical ventilation and Global survival

## CO-57 Impact of early empirical antibiotic therapy in the management of severe COVID-19 pneumonia

### Thomas Douillard^1^, Bruno Legendre^2^, Camille Ohler-Leonetti^3^, Matthieu Raymond^4^, Pierre Asfar^3^, Florian Reizine^5^, Gwenhaël Colin^6^, Johan Auchabie^7^, Julien Lorber^8^, Baptiste Hourmant^9^, Agathe Delbove^10^, Aurélien Frérou^11^, Jean Morin^4^, Pierre-Yves Egreteau^12^, Pierre Kergoat^13^, Béatrice La Combe^14^, Philippe Seguin^5^, Jean Reignier^4^, Emmanuel Canet^4^, Jean-Baptiste Lascarrou^4^, Cédric Darreau^1^

#### ^1^Centre Hospitalier du Mans, Le Mans, France; ^2^Centre Hospitalier Universitaire de Caen, Caen, France; ^3^Centre Hospitalier Universitaire d’Angers, Angers, France; ^4^Centre Hospitalier Universitaire de Nantes, Nantes, France; ^5^Centre Hospitalier Universitaire de Rennes, Rennes, France; ^6^Centre Hospitalier Départemental de Vendée, La Roche Sur Yon, France; ^7^Centre Hospitalier de Cholet, Cholet, France; ^8^Centre Hospitalier de Saint-Nazaire, Saint Nazaire, France; ^9^Centre Hospitalier Universitaire de Brest, Brest, France; ^10^Centre Hospitalier Bretagne Atlantique, Vannes, France; ^11^Centre Hospitalier de Saint-Malo, Saint-Malo, France; ^12^Centre Hospitalier de Morlaix, Morlaix, France ; ^13^Centre Hospitalier de Cornouaille, Quimper, France; ^14^Centre Hospitalier Bretagne Sud, Lorient, France

##### **Correspondence:** Thomas Douillard (douillardt@gmail.com)

*Annals of Intensive Care *2013, * 13(1): *CO-57

**Rationale:** Most patients admitted to the ICU for severe COVID-19 pneumonia received early empirical antibiotic therapy (1). This is explained by the severity at presentation with profound hypoxemia and a high rate of bacterial co-infection in other viral infections, like influenza pneumonia. In fact, the rate of bacterial co-infection seems to be very low, under 10% of cases (2). We sought to evaluate the impact of early empiric antibiotic therapy for patients admitted to the ICU with severe SARS-CoV-2 pneumonia.

**Patients and methods/materials and methods:** Our study is an ancillary analysis of the retrospective cohort “COCOV IRL”, conducted in 13 ICUs in France to assess the impact of corticosteroids. All adult patients admitted between February 1 and December 31, 2021, for severe COVID-19 were included. Two groups were formed: patients who received empiric antibiotic therapy between admission and day 2, and those who did not. The primary outcome was death on day 28. Secondary outcomes included intubation or death on day 28 and the number of ventilator-free days on day 28. Univariate and multivariate analyses were conducted including age, comorbidities, severity at presentation, corticosteroids administration, ventilatory support, and center effect as co-factors.

**Results:** One thousand and fifty-six patients were included, 852 (80%) received antibiotic therapy and 204 (20%) did not. Patients who received antibiotic therapy were more severe with higher IGS2 scores at admission (35 ± 13.2 vs 30 ± 12.7, p < 0,001). In a multivariate analysis, factors associated with death at day 28 were older age and higher IGS2 scores. There was no association between early antibiotic therapy and mortality on day 28 (HR = 0.78 [0.41; 1.47] p = 0.4). After adjustment on confounding factors, the absence of early empiric antibiotic therapy was associated with an increase of ventilator-free days at day 28 (beta = 2.8 [0.7; 4.9], p = 0.009), and a risk reduction of intubation or death at day 28 (HR 0.62 [0.45; 0.84], p = 0.003).

**Conclusion:** In our study, early empiric antibiotic therapy in severe SARS-CoV-2 pneumonia was not associated with day 28 mortality. Nevertheless, the absence of early empiric antibiotic therapy was associated with more ventilator-free days and less intubation or death at day 28. Our results do not establish a causal link but open a discussion about the real impact of early empiric antibiotic therapy in severe SARS-CoV-2 pneumonia.

**Reference 1**

1.Langford BJ, So M, Raybardhan S, Leung V, Soucy J-PR, Westwood D, et al. Antibiotic prescribing in patients with COVID-19: rapid review and meta-analysis. Clin Microbiol Infect. 2021;27:520–31.

**Reference 2**

2. Rouzé A, Martin-Loeches I, Povoa P, Metzelard M, Du Cheyron D, Lambiotte F, et al. Early Bacterial Identification among Intubated Patients with COVID-19 or Influenza Pneumonia: A European Multicenter Comparative Clinical Trial. Am J Respir Crit Care Me.

**Compliance with ethics regulations:** Yes in clinical research.

## CO-58 Multiplex PCR assay to detect nasopharyngeal viruses in immunocompromised patients with acute respiratory failure

### Alexis Maillard^1^, Jérôme Le Goff^1^, Mariame Barry^1^, Virginie Lemiale^1^, Séverine Mercier-Delarue^1^, Alexandre Demoule^2^, Linda Feghoul^1^, Samir Jaber ^3^, Kada Klouche^4^, Achille Kouatchet^5^, Laurent Argaud^6^, Francois Barbier^7^, Naike Bigé^8^, Anne-Sophie Moreau^9^, Emmanuel Canet^10^, Frédéric Pène^11^, Maud Salmona^1^, Djamel Mokart^12^, Elie Azoulay^1^

#### ^1^Hôpital Saint-Louis, Assistance Publique des Hôpitaux de Paris, Paris, France; ^2^Hôpital Pitié-Salpêtrière, Assistance Publique des Hôpitaux de Paris, Paris, France; ^3^Centre Hospitalier Universitaire de Montpellier, Hôpital Saint-Eloi, Montpellier, France; ^4^Hôpital Lapeyronie, Montpellier, France; ^5^CHU d’Angers, Angers, France; ^6^Hospices Civils de Lyon, Hôpital Edouard Herriot Lyon, Lyon, France; ^7^La Source Hospital, Centre Hospitalier Régional d'Orléans, Orléans, France; ^8^Hôpital Saint-Antoine, Assistance Publique des Hôpitaux de Paris, Paris, France; ^9^Hôpital Roger Salengro, Centre Hospitalier Universitaire de Lille, Lille, France; ^10^Centre Hospitalier Universitaire Hôtel-Dieu, Nantes, France; ^11^Hôpital Cochin, Assistance Publique des Hôpitaux de Paris, Paris, France; ^12^institut Paoli Calmette, Marseille, France

##### **Correspondence:** Alexis Maillard (alexis.maillard@aphp.fr)

*Annals of Intensive Care *2013, * 13(1): *CO-58

**Rationale:** In immunocompromised patients with acute respiratory failure (ARF), the clinical significance of respiratory virus detection in the nasopharynx remains uncertain. We aimed to evaluate the association between viral detection in nasopharyngeal swabs and the causes and outcomes of ARF.

**Patients and methods/materials and methods:** Preplanned post hoc analysis of a randomized controlled trial that enrolled immunocompromised patients admitted to 32 intensive care units for ARF. Nasal swabs sampled at inclusion were assessed for 23 respiratory pathogens using a multiplex PCR assay. The causes of ARF were established by managing physicians and reviewed by three expert investigators blinded to the multiplex PCR assay results. Associations between virus detection in nasal swabs, causes of ARF, and a composite outcome of Day-28 mortality and/or invasive mechanical ventilation (IMV) were assessed.

**Results:** Among the 512 sampled patients, 44.9% had hematological malignancies, 35.6% had solid tumors, 11.4% were solid organ transplant (SOT) recipients and 7.3% were allogeneic hematopoietic stem cell transplant (HSCT) recipients. The multiplex PCR assay was positive in 105 patients (20.5%), and a virus was detected in 104, including 36.6% rhinovirus/enteroviruses, 10.7% coronaviruses, and 53.8% flu-like viruses (influenza virus, parainfluenza virus, respiratory syncytial virus, or human metapneumovirus). The etiology of ARF varied significantly according to the results of the multiplex PCR assay, especially the proportion of viral pneumonia:50.0% with flu-like viruses, 18.2% with other viruses, and 3.6% when no virus was detected (p < 0.001). There was no difference in the composite outcome of Day-28 mortality and/or IMV according to assay positivity (53.8% vs. 54.7%, p = 0.882). In a pre-established subgroup analysis, flu-like virus detection was associated with a higher rate of Day-28 mortality and or IMV among allogeneic HSCT recipients.

**Conclusion:** In immunocompromised patients with ARF, the results of nasopharyngeal multiplex PCR assays are not associated with the need for IMV or mortality. A final diagnosis of viral pneumonia is retained in one-third of patients with a positive assay and in half of the patients carrying a flu-like virus.

**Compliance with ethics regulations:** Yes in clinical research.

## CO-59 Secondary outcomes of the CONFIDENT randomized trial of convalescent plasma therapy with high titers of neutralizing antibodies (NAb) in COVID-ARDS patients

### Benoît Misset^1^, Michael Piagnerelli^13^, Eric Hoste^4^, Nadia Dardenne^1^, David Grimaldi^20^, Isabelle Michaux^8^, Elisabeth De Waele^2^, Alexander Dumoulin^3^, Philippe Jorens^12^, Emmanuel Van Der Hauwaert^17^, Frédéric Vallot^16^, Stoffel Lamotte^14^, Walter Swinnen^19^, Nicolas De Schryver^11^, Vincent Fraipont^5^, Nathalie De Mey^9^, Nicolas Dauby^10^, Nathalie Layios^1^, Jean-Baptiste Mesland^6^, Geert Meyfroidt^7^, Michel Moutschen^1^, Veerle Compernolle^18^, André Gothot^1^, Daniel Desmecht^1^, Maria Isabel Taveira Da Silva Pereira^1^, Mutien Garigliany^1^, Tomé Najdovski^15^, Axelle Bertrand^1^, Anne-Françoise Donneau^1^, Pierre-François Laterre^6^

#### ^1^ULg, Liège, Belgique; ^2^UZB, Bruxelles, Belgique; ^3^AZ Delta, Roeselare, Belgique; ^4^UGent, Gent, Belgique; ^5^CHR, Liège, Belgique; ^6^UCL, Bruxelles, Belgique; ^7^UZL, Leuven, Belgique; ^8^UCL, Namur, Belgique; ^9^OLVZ, Aalst, Belgique; ^10^CHU St Pierre, Bruxelles, Belgique; ^11^CSPO, Ottignies, Belgique; ^12^UZA, Antwerpen, Belgique; ^13^CHU Marie-Curie, Charleroi, Belgique; ^14^AZ Groninge, Kortrijk, Belgique; ^15^Croix-Rouge, Suarlé, Belgique; ^16^CHWAPI, Tournai, Belgique; ^17^Imelda, Bonheiden, Belgique; ^18^Rode Kruis, Mechelen, Belgique; ^19^AZ Sint Blasius, Dendermonde, Belgique; ^20^ULB Erasme, Bruxelles, Belgique

##### **Correspondence:** Benoît Misset (benoit.misset@chuliege.be)

*Annals of Intensive Care *2013, * 13(1): *CO-59

**Rationale:** Passive immunization with plasma collected from convalescent patients has been used to treat COVID-19. The CONFIDENT open-label randomized trial found that mortality on day 28 was lower in COVID-ARDS patients treated with convalescent plasma (CP) than in patients treated with standard of care (SOC) (SRLF 2022 and (1,2)). The protocol planned secondary outcomes including a long-term assessment. The objective is to present secondary endpoints of the CONFIDENT trial.

**Patients and methods/materials and methods:** 475 patients with COVID-19 ARDS mechanically ventilated for less than 5 days (stratified at 48 h) were randomly assigned in a 1:1 ratio to receive either convalescent plasma with a neutralizing antibodies titer at least 1/320 or standard of care in the trial between October 2020 and March 2022. The primary outcome—day-28 mortality—was 35.4% in the CP group and 44.9% in the SOC group (p = 0.03), and 32.7% and 46.8% in the stratum included < 48 h (p = 0.008). Secondary endpoints included the antibody response to COVID-19, ICU-acquired bacteremia and pneumonia, and day-365 mortality and quality of life. Funded by KCE # COV201004.

**Results:** Total IgG titers (BAU/mL) increased from 262 [31–881] on inclusion to 1180 [621–1,720] on day 7 (p < 0.0001), 1,470 [872–2,260] on day 28, and 1,260 [797–1,960] on day 90 (p > 0.05 between CP and SOC and between days 7, 28 and 90). NAb titers and total IgG titers to SARS-CoV-2 in the patients were associated (p < 0.0001, linear mixed model). In the CP and SOC groups: bacteremia occurred in 85 (35.9%) and 102 (42.9%) (Δ [CI95] = − 7.0 [− 15.95 to 1.84]), VAP in 182 (76.8%) and 188 (79.0%) (Δ [CI95] = − 2.2 [− 9.54 to 5.05]) patients. Bacteremia per 1,000 ICU-days were 22 and 23 and occurred at a median [IQR] of 53 [10– > 100] days for the CP group and 22 [9– > 100] days for the SOC group. VAP per 1,000 MV days were 73 and 75 (p > 0.05) and occurred at median [IQR] of 7 [2–18] days for the CP group and 5 [2–13] days for the SOC group. Mortality on day 90 was 102 (44.0%) in the CP group and 121 (50.8%) in the SOC group. Day-365 mortality was 107/237 (45.1%) in the CP group and 123/238 (51.6%) in the SOC group.

**Conclusion:** In COVID-ARDS patients, anti-SARS-Cov-2 antibodies increased in the first week following inclusion and secondary infections were very high. The beneficial effect of convalescent plasma on day-28 mortality was not associated with a difference in these prespecified secondary outcomes.

**Reference 1**

Misset, BMC Pulm Med. 2020 Dec 7;20(1):317.

**Reference 2**

Misset, Intensive Care Medicine Experimental 2022, 10(2):000713.

**Compliance with ethics regulations:** Yes in clinical research.

## CO-60 Epidemiology of acute intoxications among detainees in the intensive care unit

### Hassen Ben Ghezala^1^, Boudour Ben Dhia^1^, Amira Ben Jazia^1^, Nozha Brahmi.^1^

#### ^1^Centre Mahmoud Yaacoub d'assistance médicale urgente de Tunis, Tunis, Tunisie

##### **Correspondence:** Hassen Ben Ghezala (hassen.ghezala@gmail.com)

*Annals of Intensive Care *2013, * 13(1): *CO-60

**Rationale:** Intoxication among detained patients for the purpose of autolysis is still a taboo subject. The aim of the study is to assess the epidemiological indicators of acute intoxications, the factors that affect the clinical course and the outcome in ICU.

**Patients and methods/materials and methods:** It is a retrospective, descriptive and cross-sectional study, including all critically ill detainees patients admitted in the medical and toxicology intensive care unit of a reference poison center, from 1st January 2020 to 31st January 2023. We collected demographic data of the detained population, exposures, suspected intoxications, clinical and paraclinical manifestations, and treatments.

**Results:** Eighty-one acute poisoning was included in the study: sixty patients (96% male); twenty-one recurrent episodes [2,5]. Most patients (51%) were unemployed, 10% were a day labourer. The mean age was 34 years [24,69]. 75% were single. Diabetes mellitus, chronic heart disease and chronic obstructive pulmonary disease were the most common co-morbidities (40.7%, 17.3%, 11.1%). Psychiatric disorders were recorded in 35% patients. 40% had a personal history of suicide attempts ranging from 2 to 5 attempts,75% of which required admission in ICU. 80% were belonging to low socioeconomic class. Median SAPS II and APACHEII scores at admission were respectively 8[6,50], 2[0,22]. Suicidal attempt leading to acute poisoning was present in 65%22% have intoxication for a secondary interest, 10% were body packer and withdrawal syndrome was recorded in 2.5% of cases. The mean delay of presentation to the emergency department was 15 h. For forty-five patients poisoning was due to oral antidiabetics (50% metformin and 22% sulfonamide), 22% due to conversion enzyme inhibitor, 13% due to beta-blockers, 6% to paracetamol. The predominant clinical manifestations were gastrointestinal manifestations (79%), hemodynamic failure (13%), neurologic failure (13%), and only one patient had respiratory failure. 53% had metabolic disorders: metabolic acidosis (79,4%), hyperlactatemia (64%), renal failure (26.5%), rhabdomyolysis (12%). Most patients were treated with supportive measures, one patient was given gastrointestinal decontamination and specific antidotes were used in 20% of patients. Four patients (5%) required invasive mechanical ventilation. Hemodialysis was used for five patients. The median length of hospital stay was 2 days [2,31], and the mortality rate was about 1%.

**Conclusion:** Acute poisoning is an alarming health problem in the prison. It is important to think about how to limit its impact.

**Compliance with ethics regulations:** Yes in clinical research.

## CO-61 Proposal of a new severity score in toxicology: the "scoretox"

### Hela Maamouri^1,2^, Yosra Ghali ^2^, Nozha Brahmi^2^

#### ^1^CH Rambouillet, Rambouillet, France; ^2^Centre Mahmoud Yaacoub d'Assistance Médicale Urgente, Tunis, Tunisie

##### **Correspondence:** Hela Maamouri (helamaamouri@yahoo.fr)

*Annals of Intensive Care *2013, * 13(1): *CO-61

**Rationale:** Various scores in intensive care are calculated to estimate the severity of the patients, or to help with diagnostic approach. Until now, there is no specific score for intoxicated patients that can be easily calculated and validated to evaluate the severity of the patients. The aim of this work was to validate a pre-established score; the "scoretox" for patients admitted following acute intoxication by cardiotoxic product and to study its performance.

**Patients and methods/materials and methods:** It is an observational, longitudinal, retrospective, monocentric study including all acute intoxications with cardiotropic agents admitted to the medical intensive care unit Mahmoud Yaacoub Center from January 2018 to October 2021. The primary endpoint was severity. The score is calculated at admission and based on sixteen items including patientage, history, clinical and paraclinical features, and therapeutic behaviors at admission.

**Results:** We included 2495 patients including 604 patients admitted for poisoning by cardiotropic agents. The median age of the patients was 25 years [18–37] with a female predominance and a gender ratio M/F at 0,5. The IGSII, APACHE II and scoretox scores were calculated after patient admission with a respective median of 14 [8–28], 5 [2–10] and 2 [1–4]. Sinus bradycardia was found in 21% of patients (n = 106). Eleven patients (1.8%) showed a membrane stabilizing effect on the electrocardiogram. Seventy-seven patients (12%) were in cardiogenic shock. The performance study had shown that scoretox performed better in patients admitted for cardiotoxic poisoning by comparing it with IGSII and APACHE II. It also had the highest likehood ratio and the highest specificity. There was a linear and positive correlation between scortox and these scores (p < 10^4^) with a stronger relationship between scortox and APACHEII (r = 0.457). In addition, in the study of calibration, the scoretox was not well calibrated.

**Conclusion:** The new severity prediction score for patients admitted for cardiotropic intoxication was outperformed by the IGSII, APACHEII and PSS scores.This study suggests that the "scoretox" can be useful to assess the severity of patients admitted to intensive care for poisoning with cardiotoxic drugs. An external validation on a multicenter study is however necessary.

**Compliance with ethics regulations:** N/A.

## CO-62 Long-term exposure to Sargassum-seaweed pollution in the French Caribbean Islands: clinical characteristics, consequences, and outcome

### Dabor Resiere^1^, Jonathan Florentin^1^, Rishika Banydeen^1^, Hatem Kallel^1^, Mehdaoui Hossein^1^, Remi Neviere^1^

#### ^1^CHU de Martinique, Fort-de-France, Martinique, France

##### **Correspondence:** Dabor Resiere (dabor.resiere@chu-martinique.fr)

*Annals of Intensive Care *2013, * 13(1): *CO-62

**Rationale:** To evaluate the clinical characteristics and consequences of long-term exposure to noxious gas emissions from decomposing Sargassum in the local population of Martinique.

**Patients and methods/materials and methods:** We conducted a prospective follow-up of a patient cohort admitted to the emergency department (University Hospital of Martinique) due to exposure to sargassum from March 2018 to December 2022. We assessed the patient's exposure to Sargassum and air pollutants based on recordings of coastline sensors measuring H2S and NH3 levels and data from the Regional Air Quality Observatory. Demographics and clinical data (including cardiovascular, neurological, and respiratory events) were collected.

**Results:** In the six-year study period, 560 patients were included. Patients mainly arrived with referral letters from their general practitioner (80%) and presented headaches (76%), gastrointestinal disturbances (79%), dizziness (54%), skin lesions (30%), cough (44%), and conjunctivitis (33%). Not all patients were clinically symptomatic. Initial lung function tests were regular (50%), and twelve patients were admitted to the intensive care unit.

**Conclusion:** Our study indicates that the magnitude of health effects following long-term exposure to Sargassum may be larger than previously recognized. Efforts to limit long-term exposure are mandatory.

**Compliance with ethics regulations:** N/A.

## CO-63 Datura "devil's herb" rare intoxication: A 15-year retrospective study

### Salma Ghalloussi^1^, Amira Ben Jazia^1^, Hassen Ben Ghezala^1^, Boudour Ben Dhia^1^, Mariem Cheikhrouhou^1^, Ons Ellouze^1^, Nozha Brahmi^1^

#### ^1^CAMU, Tunis, Tunisie

##### **Correspondence:** Salma Ghalloussi (salmaghalloussi93@gmail.com)

*Annals of Intensive Care *2013, * 13(1): *CO-63

**Rationale:** Datura stramonium is a ubiquitous solanaceous plant found in urban and rural areas. It has become popular for its hallucinogenic effect and is consumed in various forms. All parts of the plant are toxic because they contain substances (alkaloids) that oppose the action of a neurotransmitter, acetylcholine. These compounds are atropine, scopolamine, hyoscyamine.

**Patients and methods/materials and methods:** We report a series of cases of acute intoxication with Datura hospitalized in the department of intensive care and toxicology of Mahmoud Yaacoub center of Tunis (CAMU) from October 2008 to February 2022.

**Results:** Sixteen patients were eligible, among them nine were male (56%). The median age was 39 years with extremes of 17 and 79 years. The main season of intoxication was spring (37%). The median time to onset of symptoms was 1 h [1,8] and the median time to emergency room visit was 6 h [2,24]. Only one patient was followed in psychiatry for a bipolar disorder and four patients were addicted to drugs (25%). Intoxication was voluntary in six patients and accidental in six others. Clinical signs were mydriasis with photophobia and blurred vision (93%), mucocutaneous dryness (43%), intense thirst (40%), fever (10%), dysphagia (25%), nausea (35%) and difficulty in speaking (13%). The presence of a bladder globe was noted in five patients (31%). The neuropsychological signs were almost constant including agitation (88%), hallucinations (88%), confusion (81%) reminiscent of alcoholic drunkenness, somnolence (63%), coma in three patients (19%) and seizures in two patients who required mechanical ventilation. Cardiovascular signs were palpitations (49%), sinus tachycardia (41%), bradycardia (13%), and hypertension (39%). Acute renal failure was present in 12% of cases. No digestive decontamination was performed in this series. Symptomatic treatment was effective in most cases. All intubated patients were extubated and evolved favorably.

**Conclusion:** The diagnosis of Datura intoxication is clinical. Its low cost and easy access are the factors of an increase in its consumption as a psychoactive substance. The prognosis is related to clinical disorders: hospitalization is always necessary.

**Compliance with ethics regulations:** Yes in clinical research.

## CO-64 White henbane «Jusquiame» poisoning: a ten-year retrospective study

### Boudour Ben Dhia^1^, Amira Benjazia^1^, Hassen Benghazala^1^, Salma Ghalloussi^1^, Salma Esseghaeir^1^, Mariem Hakmouni^1^, Ikram Ben Braeik^1^, Ons Ellouze^1^, Nozha Brahmi^1^

#### ^1^CAMU, Tunis, Tunisie

##### **Correspondence:** Boudour Ben Dhia (bidourabdbg2014@gmail.com)

*Annals of Intensive Care *2013, * 13(1): *CO-64

**Rationale:** White henbane « jusquiame» is a very rare plant, of the family Solanaceae, its use is reserved for the medical profession since it is a powerful narcotic, used as antispasmodic, sedative and analgesic or even anesthetic. The alkaloids contained in this plant have toxic power both dermal and ingested form. We reported this series of cases of jusquiame poisoning to emphasize on clinical signs and a potentially fatal prognosis.

**Patients and methods/materials and methods:** It is a retrospective study conducted in our intensive care unit during a period of ten-year (2012–2022). Were included all patients admitted for henbane intoxication.

**Results:** Seven patients were included in our study, with gender-ratio (F/M) = 1.3 (ages 24–83 years). Intoxications were consequent to accidental ingestion. One patient used this herb from self-medication and one for recreative purpose. All the cases have been severely intoxicated and they were presented with an anticholinergic toxidrome two to six hours after ingestion. Neurological manifestations of intoxication were mydriasis and exaggeration ofosteo-tendon reflexes for all cases. Four patients had loss of consciousness with CGS (from 14 to 8), 67% with confusion, agitated delirium in two cases and one patient presented seizures. The conscious patients (33%) had described excruciating headaches. All patients had mild tachycardia with a mean of 90 ± DS 20. One patient had respiratory failure. The other symptoms were mucocutaneous dryness (83%), flushed skin (32%), urinary retention (50%) and nausea, vomiting (50%). One patient had acute renal failure. Management was symptomatic for all patients, one case required mechanical ventilation and sedation by benzodiazepine. The median length of stay was 1 day [1–9]. There were no death.

**Conclusion:** Health care providers and physicians, in particular emergency physicians and clinical toxicologists, should be aware of the nature, medical uses, clinical characteristics, diagnosis and management of White henbane intoxication.

**Compliance with ethics regulations:** Yes in clinical research.

## CO-65 Metformin associated lactic acidosis: mechanism of toxicity and extracorporeal treatment

### Hela Maamouri^1,2^, Yosra Hamrouni ^2^, Nozha Brahmi^2^

#### ^1^CH Rambouillet, Rambouillet, France; ^2^Centre d'Assistance médicale Urgente, Tunis, Tunisie

##### **Correspondence:** Hela Maamouri (helamaamouri@yahoo.fr)

*Annals of Intensive Care *2013, * 13(1): *CO-65

**Rationale:** Metformin is a biguanide oral antidiabetic agent used as a first line treatment of type II diabetes. Metformin toxicity is rare but may lead to life threatening lactic acidosis with high mortality rate. Hemodialysis is the only method allowing removing of metformin and lactate clearance. The aim of this study is to determin the best moment of hemodialysis initiation and its predictive factors.

**Patients and methods/materials and methods:** It is a retrospective monocentric study on a series of clinical cases with metformin poisoning admitted to intensive care unit of CMYAMU conducted from January 2011 to February 2021. We analyzed anamnestic, clinical and gasometrical data (kinetics of pH, HCO3 and lactate at H8 and H12 of admission) with SPSS version 24.0

**Results:** We included 200 patients admitted for metformin poisoning. Most of cases (99,5%) had voluntary intake metformin. Median ingested dose was 14,45 g [8.5–22.5]. Co-ingestions were found in 38.5% of cases. Median age was 25 years [20–33] with female predominance. Psychiatric history was frequently identified (27,5%, n = 55). The mean delay between ingestion and admission was about 3 h. Most of patients consulted for digestive symptoms. Eight patients needed vasoactive amines treatment and four of them required mechanical ventilation. At admission, average of pH was at 7.36, bicarbonates at 19.83 and lactate at 4.45 mmol/l. Hemodialysis was required for thirty-two patients (16%) and was based on kinetics of pH, bicarbonates and serum lactates at H8 and H12 from admission. Median duration of dialysis was about 4 h [3–6]. Independent predictive hemodialysis factors identified by multivariate analysis were serum lactate 11 mmol/l [95% CI 6.03–715], ingested dose superior to 35.843 g [95% CI 3.1–50.1] and anion gap superior to 27.5 [95% CI 4.3–284].

**Conclusion:** Metformin poisoning prognosis depends on patient comorbidities, circumstances that leaded to toxicity and moment of extracorporeal treatment. Kinetics of pH, bicarbonates and serum lactates may help clinicans to indicate the best moment of hemodialysis. Four hours of conventional hemodialysis may be effective for patients without comorbidities.

**Compliance with ethics regulations:** N/A.

## CO-66 Effect of post extubation High-Flow Nasal Oxygen vs Non-invasive Ventilation on reintubation among burn patients at high risk of extubation failure

### Hajer Fakhfakh^1^, Hana Fredj^1^, Sarra Zarrouk^1^, Emna Hammas^1^, Bahija Gasri^1^, Imen Jami^1^, Manel Ben Saad^1^, Amel Mokline^1^, Amen Allah Messadi^1^

#### ^1^Centre de Traumatologie et des Grands brûlés, Ben Arous, Tunisie

##### **Correspondence:** Hana Fredj (fredjhana@yahoo.fr)

*Annals of Intensive Care *2013, **13(Suppl 1)**:CO-66

**Rationale:** Extubation failure is a complication that increases morbidity and mortality. Non invasive ventilation (NIV) and High flow nasal cannula (HFNC) are two commonly used methods to provide respiratory support after. However, the optimal approach for post-extubation support in severe burn patients remains controversial. The aim of this study was to assess the effect of postextubation High-Flow Nasal Oxygen vs Noninvasive Ventilation on reintubation among burn patients at high risk of extubation failure.

**Patients and methods/materials and methods:** Retrospective case–control study was conducted in intensive burn care in Tunisia over a period of two years (2021–2022). Two groups of patients were assigned: (G1: HFNC), and (G2: NIV). The primary outcome was the proportion of patients reintubated at day 3; secondary outcomes were length of stay and ICU mortality.

**Results:** During this period, 235 were undergoing mechanical ventilation. Sixty-two were extubated. Thirty-four patients were included and divided in 2 groups: G1 (HFNC; n = 17), and G2 (: NIV; n = 17). The majority of patients were intubated for facial burns (N = 21). The two groups were comparable in terms of age, TBSA and severity of burns. Our study shows that there was no significant difference between the use of HFNC and NIV on the prevention of reintubation. The number of reintubated patients was comparable in two groups (3/17 versus 4/17, p: ns). Reintubation was within less than 72 h in all cases. There was no difference between the two groups in terms of interface tolerance (Table1). The length of stay and the mortality were comparable in the two groups (Table1).

**Conclusion:** In mechanically ventilated burns at high risk of extubation failure, HFNC therapy was comparable to NIV for preventing reintubation.

**Compliance with ethics regulations:** Yes in clinical research.

**Table 1 (abstract CO-66):**
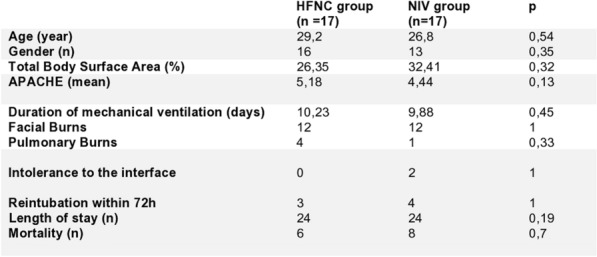
Comparative study of two groups

## CO-67 Assessment of sound generated by cough during weaning of mechanical ventilation to predict extubation failure: Sono-Wean study

### Vincent Bonny^1^, Jeremie Joffre^1^, Paul Gabarre^1^, Tomas Urbina^1^, Louai Missri^1^, Maxime Gasperment^1^, Bertrand Guidet^1^, Eric Maury^1^, Hafid Ait-Oufella^1^

#### ^1^Hôpital Saint-Antoine, Paris, France

##### **Correspondence:** Vincent Bonny (vincent.bonny@aphp.fr)

*Annals of Intensive Care *2013, **13(Suppl 1)**:CO-67

**Rationale:** The main risk of extubation is a relapse of respiratory distress with the need for reintubation. This is associated with increased morbidity and mortality. The mechanisms implicated in weaning failure are not fully understood and determining when a patient can be separated from the ventilator remains challenging.

**Patients and methods/materials and methods:** In patients who presented a successful spontaneous breathing trial on a T piece, we performed a quantitative measure of sound (dB) induced by three coughing efforts. This measure was done by using the sonometer Pulsar 14, two centimeters away from the distal extremity of the endotracheal tube.

**Results:** 106 patients were analyzed. Extubation failure occurred in 15 patients. The median [interquartile range] age was 65 [51–75], 36% of patients were women, 43% required intubation for acute respiratory failure, 25% for coma, 17% for shock, 6% for cardiac arrest and 9% for other reasons. The median duration of mechanical ventilation at enrollment was 4 [3–7] days. The sonoscore was significantly higher in patients who succeeded extubation, the median score being 222 [197–233] against 174 [156–191] dB, p < 0.001 in patients presenting extubation failure. In patients who succeeded extubation, the sound intensity produced by the last cough was also significantly higher than the one produced by the first, whereas there was no difference in sound intensity in patients with extubation failure. The area under the Receiver Operating Characteristic curve of the sonoscore to predict extubation failure was 0.911 IC 95% [0.829–0.993], p < 0.001. With a cut-off value of sonoscore at 180 dB the relative risk to present extubation failure was 16.38 [5.2–51], p < 0.001, and with a cut-off value at 200 dB the relative risk was at 5.3 [3.2–8.6], p < 0.001.

**Conclusion:** In weaning patients, undergoing mechanical ventilation for at least 24 h in the intensive care unit, the intensity of the sound induced by coughing measured with a sonometer seems to predict extubation failure.

**Compliance with ethics regulations:** Yes in clinical research.

**Figure 1 (abstract CO-67):**
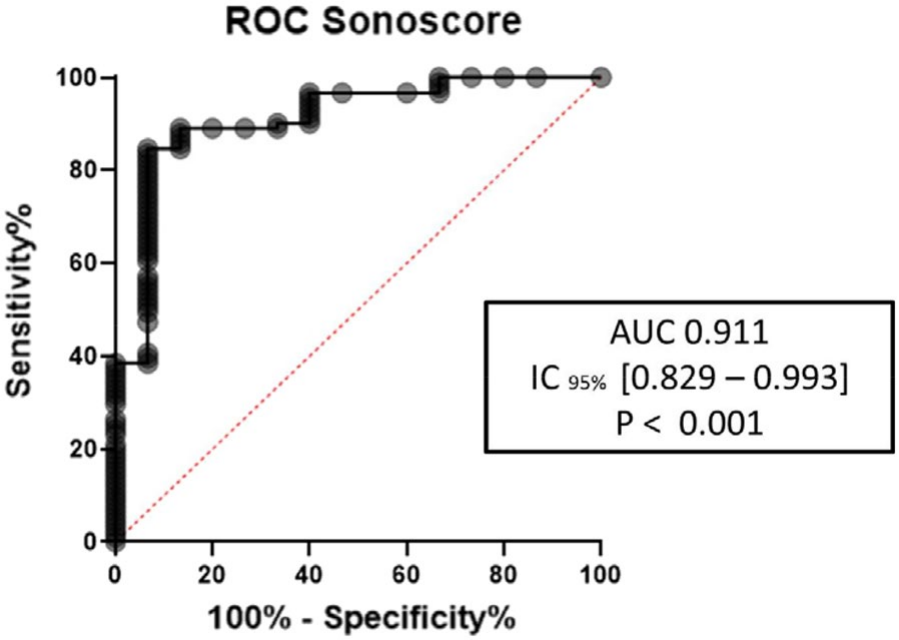
Performance of the Sonoscore during spontaneous breathing trial to predict extubation failure

## CO-68 Patient outcomes in a weaning unit after prolonged stay in intensive care: a review of 2022

### Jean-Baptiste Peretout^1^, Esther Mbakulu^1^, Karim Nssair^1^, Razach Abdallah^1^, Raphael Lepecq^1^, Gérald Choukroun^1^

#### ^1^Hôpital Forcilles-Fondation Cognacq-Jay, Ferolles-Attilly, France

##### **Correspondence:** Jean-Baptiste Peretout (jperetout@cognacq-jay.fr)

*Annals of Intensive Care *2013, **13(Suppl 1)**:CO-68

**Rationale:** Weaning units have shown their value in the management of tracheostomized patients after prolonged intensive care^1^. The global and multidisciplinary approach contributes to accelerate and improve weaning^2^. The objective of this study is to describe the results of weaning and decannulation in these patients.

**Patients and methods/materials and methods:** This is a retrospective, descriptive study in a respiratory weaning unit over the year 2022. All patients hospitalized in the weaning unit were included. Demographic characteristics and weaning unit outcomes were collected. All inpatients with complete data were analyzed. The analysis was completed by a subgroup analysis of patients on 24-h spontaneous ventilation at admission and patients still with mechanical ventilation (MV) at admission (intermittent or continuous).

**Results:** In 2022, 103 tracheostomized patients were admitted. A total of 97 patients were analyzed with male 62%, age 62 ± 12 years and length of stay 58 ± 27 days in intensive care unit. The main diagnosis at admission in ICU was acute respiratory failure. The goal of admission on weaning unit was: ventilatory and tracheostomy weaning in 77% and tracheostomy weaning in 23%. On admission, patients were on 24-h spontaneous ventilation in 24% (n = 23) and on mechanical ventilation in 76% (n = 74): 80% intermittent MV (n = 59); 20% continuous MV (n = 15). In overall patients, 24-h spontaneous ventilation was achieved in 10.2 ± 13.9 days, decannulation in 19 ± 16.3 days and the length of stay was 35.8 ± 27 days. The overall decannulation success rate was 86.6%. Subgroup analyses are presented in Table I.

**Conclusion:** Patients with prolonged intensive care and complex medical needs treated in a specialized weaning unit had high weaning and decannulation rates. This was achieved with a multidisciplinary team approach to continued intensive care and simultaneous rehabilitation with physiotherapists, speech therapists, nutritionists… Cares in a weaning unit remains relevant despite a prolonged stay in ICU.

**Reference 1**

1. Boles JM, Bion J, Connors A, Herridge M, Marsh B, Melot C, Pearl R, Silverman H, Stanchina M, Vieillard-Baron A, Welte T. Weaning from mechanical ventilation. Eur Respir J. 2007 May;29(5):1033–56.

**Reference 2**

2. Nelson JE, Cox CE, Hope AA, Carson SS. Chronic critical illness. Am J Respir Crit Care Med. 2010 Aug 15;182(4):446–54.

**Compliance with ethics regulations:** Yes in clinical research.

**Table I (abstract CO-68):**
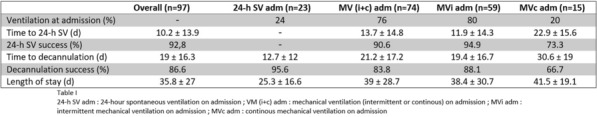
Clinical outcomes

## CO-69 A machine learning approach to predict weaning outcome among ventilated patients in Intensive Care unit

### Romain Lombardi^1^, Mathieu Jozwiak^1^, Jean Dellamonica^1^, Claude Pasquier^2^

#### ^1^CHU De Nice, Nice, France; ^2^Laboratoire I3S, Biot, France

##### **Correspondence:** Romain Lombardi (lombardi.romain@gmail.com)

*Annals of Intensive Care *2013, **13(Suppl 1)**:CO-69

**Rationale:** Machine learning has been illustrated in various medical fields, such as fluid management in sepsis, prediction of renal failure and others. The weaning period is the key to the management of a patient on mechanical ventilation. Weaning can take up to half the time spent in intensive care. Up to 20% of patients do not pass their first withdrawal test. However, mortality can reach 38% in patients with the most difficult weaning. [1] Only a small number of studies have looked at the application of machine learning to the weaning process. Being able to predict the success of spontaneous breathing test and extubation is essential to improve morbi-mortality and reduce length of stay. We propose to develop a predictive algorithm for the success of a weaning test and identify the different factors that determine this success.

**Patients and methods/materials and methods:** It is a critical care, single-centre and retrospective study. Most of the variables are taken from the literature and extracted from the computerized patient record. We designed several machine learning algorithms: Logistic Regression, Random Forest Classifier, Support Vector Classifier (SVC), K-Nearest Neighbors algorithm (KNN), XGBoost, and Light Gradient Boosting Method (LGBM). In order to maximize the accuracy of the prediction and to reduce the risk of overfitting, we computed different methods: multiple imputation with K-Nearest Neighbors for missing data, the SMOTE method (Synthetic Minority Oversampling technique) and K-fold cross validation. The results are expressed in terms of AUC, and the factors’ importance is determined by the SHAP (Shapley added explanations) method.

**Results:** Our cohort included 80 patients (60 successful, 20 unsuccessful). The best algorithm was LGBM with AUC 0.873 (F1score: 0.87, AUC-pr: 0.95, accuracy: 0.813), Figure 1. The other algorithms had a worse predictive performance (Random Forest: 0.727, Logistic Regression: 0.8, XGBoost: 0.836). Weight gain since admission, presence of VAP, protidemia and fibrinogenemia at the time of testing, and duration of invasive ventilation were considered to have the greatest impact on the model.

**Discussion:** Despite the small number of patients, all the techniques in place allow good prediction and reproducibility. However, the cohort will continue to grow until the congress (to a minimum of 250 patients). The main determinants affecting the success of the test are similar to those found in other studies in this field and are factors that can be directly influenced.

**Conclusion:** Machine learning will be useful in predicting weaning success and will be a real help in daily clinical practice.

**Reference 1**

Béduneau, G., 2017. Epidemiology of Weaning Outcome according to a New Definition. The WIND Study. Am J Respir Crit Care Med 195, 772–783. https://doi.org/10.1164/rccm.201602-0320OC

**Compliance with ethics regulations:** Yes in clinical research.

**Figure 1 (abstract CO-69):**
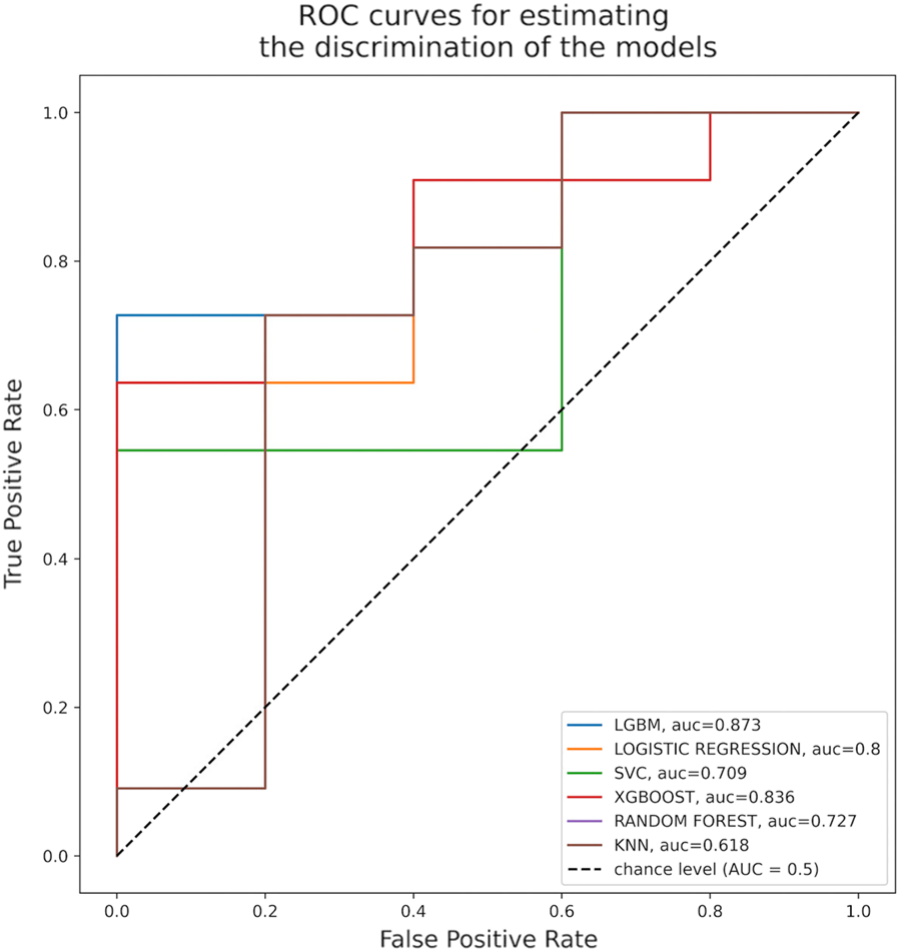
ROC curves demonstrating the performances of the machine learning algorithms for predicting successful weaning

## CO-70 Evaluation of physiological parameters on spontaneous breathing trials with T-piece and weaning outcomes in mechanically ventilated patients

### Kahaia De Longeaux^1^, Erwan L'her^1^

#### ^1^CHU de Brest, Brest, France

##### **Correspondence:** Kahaia De Longeaux (delongeaux.kahaia@gmail.com)

*Annals of Intensive Care *2013, **13(Suppl 1)**:CO-70

**Rationale:** Despite a successful spontaneous breathing trial (SBT), until 30% of the patients will need a new intubation in the 72 h following extubation, defining weaning failure. SBT failure and weaning failure are associated with poor outcomes. The aim of this study was to evaluate tidal volume, EWS.O2 score (including respiratory rate, pulsed saturation of oxygen (SpO2), inspired fraction of oxygen and heart rate) and heart rate variability (HRV) in patients during weaning process.

**Patients and methods/materials and methods:** We conducted a prospective cohort study in the medical critical care department of a teaching hospital. Patients ventilated for more than 24 h and meeting predefined criteria for weaning were included. SBTs were performed with a T-piece during 30 min. Tidal volume, EWS.O2 score and HRV parameters were collected, continuously and non-invasively, before, during the test and after extubation. Tidal volume was measured using a non-contact time-of-flight camera. EWS.O2 was recorded automatically and continuously with a closed-loop device used for oxygen flow titration according to SpO2 target set by the clinician.

**Results:** 52 spontaneous breathing trials were analyzed. We observed an increase of the EWS.O2 score and no increase of tidal volume in patients failing SBT. In patients extubated after a successful test but needing reintubation in the 72 h, an increase in EWS.O2 during and after the test was observed, no increase in tidal volume was observed and multiple scale entropy (an HRV nonlinear parameter) increased in the first ten minutes of the test. A > 7,8 ml/kg and a > 450 mL cutoffs for tidal volume at 5 min from the start of the SBT have a 100% sensitivity to predict weaning success (AUC = 0,824, p < 0,001 and AUC = 0,789, p = 0,002 respectively).

**Conclusion:** Non-invasive and continuous evaluation, easy to acquire, of physiological parameters during T-piece SBTs could help us better and early identify patients at risk of weaning failure. We aim to develop an artificial intelligence dynamic model to predict weaning outcome using those parameters.

**Compliance with ethics regulations:** Yes in clinical research.

## CO-71 Cortical respiratory drive predicts weaning failure

### Christophe Rault^1,2,3^, Quentin Heraud^3^, Stéphanie Ragot^2,3^, Jean-Pierre Frat^1,2,3^, Rémi Coudroy^1,2,3^, Rene Robert^1,2,3^, Arnaud Wilfrid Thille^1,2,3^, Xavier Drouot^1,2,3^

#### ^1^CHU Poitiers, Poitiers, France; ^2^Université de Poitiers, Poitiers, France; ^3^CIC 1402, Poitiers, France

##### **Correspondence:** Christophe Rault (christophe.rault@chu-poitiers.fr)

*Annals of Intensive Care *2013, **13(Suppl 1)**:CO-71

**Rationale:** Introduction: In intensive care unit, prediction of spontaneous breathing trial outcome and weaning from assisted ventilation remains difficult (1). Since the inspiratory cortical command is strongly involved during respiratory efforts (2), the amplitude of this inspiratory premotor cortical command could be associated with the success or failure of weaning from the ventilator.

**Patients and methods/materials and methods:** Material and methods: In 68 intubated and ventilated patients in intensive care unit, the amplitude of inspiratory premotor potentials was measured by quantified electroencephalography. The evolution (ΔIPP) of this amplitude between breathing under mechanical ventilation and spontaneous breathing through a T-piece was calculated. ΔIPP was compared between patients who failed and those who passed the spontaneous breathing test.

**Results:** Results: ΔIPP was significantly different between the failure and success groups (p < 0.0001). ΔIPP increased (i.e. inspiratory premotor potentials amplitude increased in spontaneous breathing compared to mechanical ventilation) in the failure group and decreased in the success group. An increase in ΔIPP above the threshold of 0.425 µV was associated with failure of the T-piece test with a sensitivity of 100% and a specificity of 87% (Figure).

**Conclusion:** Conclusion: Premotor inspiratory potentials amplitude is a reliable reflection of the difficulties experienced by intubated patients when stopping the ventilator and could be useful in clinical practice if the measurement method was simplified.

**Reference 1**

Thille AW, Richard JC, Brochard L. The decision to extubate in the intensive care unit. Am J Respir Crit Care Med. 2013;187(12):1294–302.

**Reference 2**

Rault C, Sangare A, Diaz V, Ragot S, Frat JP, Raux M, et al. Impact of Sleep Deprivation on Respiratory Motor Output and Endurance. A Physiological Study. Am J Respir Crit Care Med. 2020;201(8):976–83.

**Compliance with ethics regulations:** Yes in clinical research.

**Figure 1 (abstract CO-71):**
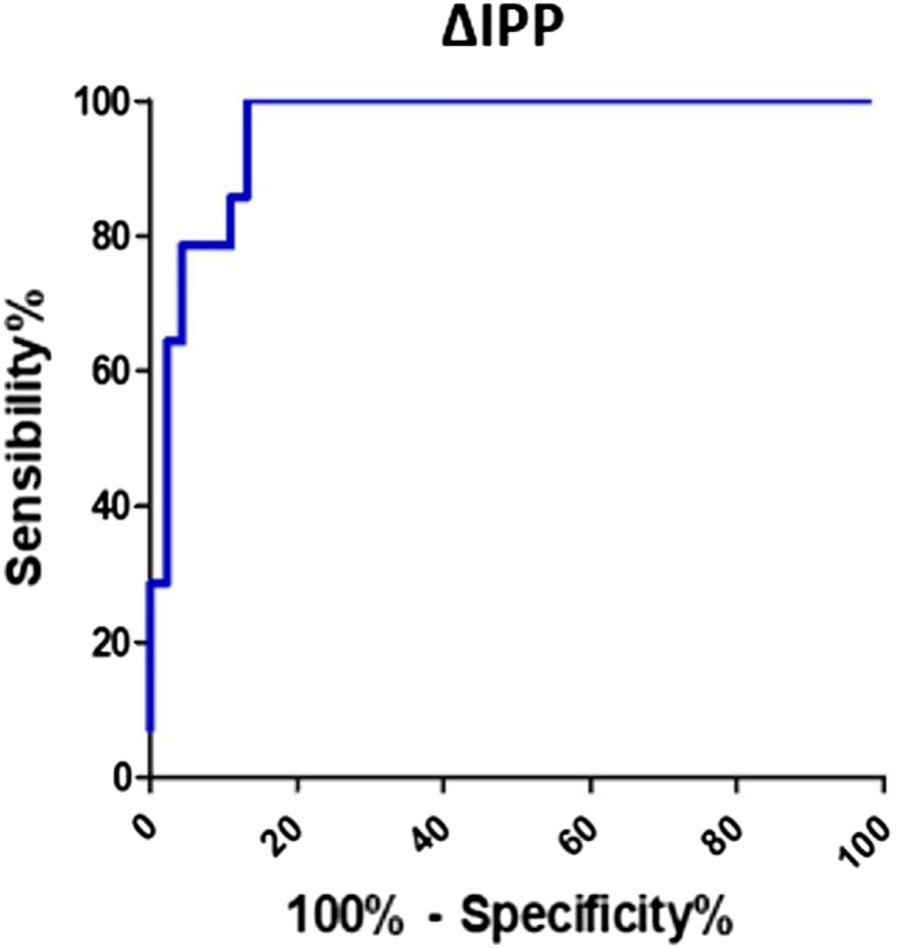
ROC curve for determining an inspiratory premotor potential amplitude threshold predicting the outcome of spontaneous breathing trial. The threshold of 0.425 µV in DeltaIPP gave a sensitivity of 100%, a specificity of 87%

## CO-72 Community-acquired Legionella Pneumonia in Intensive Care Unit: clinical characteristics and prognostic factor—Primary results of a multicenter retrospective study

### Anaïs Dartevel^1^, Louis-Marie Galerneau^1^, Vincent Peigne^5^, Nicholas Sedillot^6^, Stepan Erhmann^7^, Kada Klouche^2^, Alexandre Lautrette^8^, Julien Poissy^9^, Guillaume Thiery^4^, Bertrand Sauneuf^10^, Jean-Philippe Rigaud^11^, Michel Ramakers^12^, Carole Schwebel^1^, Nicolas Terzi^3^

#### ^1^CHU Grenoble, Grenoble, France; ^2^CHU Montpellier, Montpellier, France; ^3^CHU Rennes, Rennes, France; ^4^CHU Saint-Etienne, Saint-Etienne, France; ^5^Centre Hospitalier Chambéry, Chambéry, France; ^6^Centre Hospitalier Bourg en Bresse, Bourg En Bresse, France; ^7^CHU Tours, Tours, France; ^8^CHU Clermont-Ferrand, Clermont-Ferrand, France; ^9^CHU Lille, Lille, France; ^10^Centre Hospitalier Cotentin, Cotentin, France; ^11^Centre Hospitalier Dieppe, Dieppe, France; ^12^Centre Hospitalier Saint-Lô, Saint-Lô, France

##### **Correspondence:** Anaïs Dartevel (adartevel@chu-grenoble.fr)

*Annals of Intensive Care *2013, **13(Suppl 1)**:CO-72

**Rationale:** Legionella Pneumophilia is a cause of pneumonia in ICU (Intensive Care Unit). We aimed for the first end-point to describe the epidemiology and outcomes of Legionella pneumonia in French ICU. Secondary, we aimed to explore potentials death risk factors.

**Patients and methods/materials and methods:** We performed a multi-center retrospective study in 11 different French medical or medico-chirurgical ICUs between January 2014 and December 2019.

**Results:** Legionella pneumonia was diagnosed in 157 patients in the study period. Invasive ventilation was necessary for 93 patients (58.6%). Among these, 73 presented the criteria of acute respiratory distress syndrome (ARDS). Most of these patients were treated with bi-therapy antibiotics (124, patients; 79%). The most frequent bi-therapy was fluoroquinolone and macrolide (116 patients). The median duration of ICU stay was 11 days (Q1–Q3 = 5–19). Among the 157 patients, 19 patients did not survive at 28 days. In multivariate analyses, only age (HR, 1.07; 95% CI, 1.01–1.13) SOFA score (HR, 1.30; 95% CI, 1.04–1.63) and septic shock at baseline (HR, 4.37; 95% CI, 1.05–18.32) were significantly associated with mortality.

**Conclusion:** Mortality at 28 days in ICU of legionella was 12.1%. The septic shock in Legionella Pneumophilia in ICU was independently associated with mortality.

**Compliance with ethics regulations:** Yes in clinical research.

**Figure 1 (abstract CO-72):**
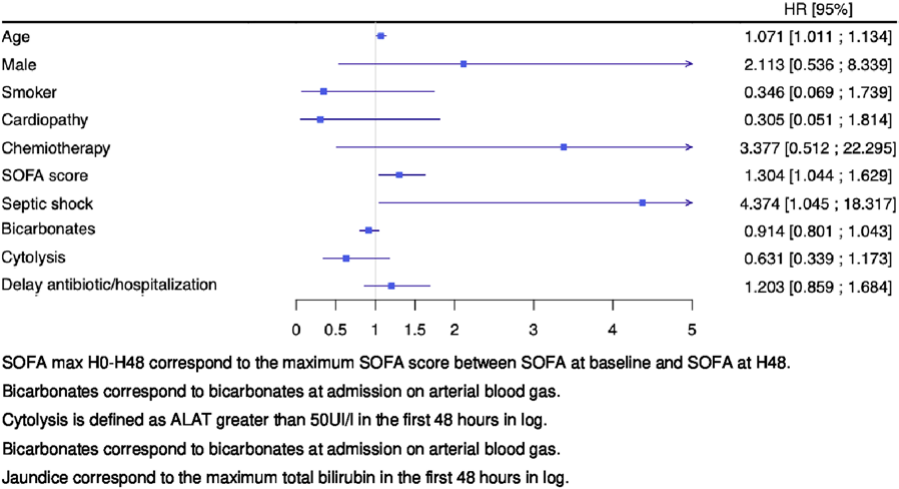
Forrest plot of multivariate analysis of survival at 28 days by Cox regression and stratified by center

## CO-73 Hydrocortisone and fludrocortisone for community acquired pneumonia

### Nicholas Heming^1^, Alain Renault^1^, Emmanuelle Kuperminc^1^, Miguel Carlos^1^, Virginie Maxime^1^, Rania Bounab^1^, Pierre Moine^1^, Djillali Annane^1^

#### ^1^Hôpital Raymond Poincaré, Garches, France

##### **Correspondence:** Nicholas Heming (nicholas.heming@aphp.fr)

*Annals of Intensive Care *2013, **13(Suppl 1)**:CO-73

**Rationale:** The role of corticosteroids in the management of non COVID-19 community acquire pneumonia (CAP) remains controversial.

**Patients and methods/materials and methods:** The Activated Protein C and Corticosteroids for Human Septic Shock (APROCCHSS) trial was a placebo, controlled trial of the efficacy and safety of the corticosteroids hydrocortisone and fludrocortisone, in adults with septic shock. Patients with CAP were identified a priori as a subgroup of interest. The primary outcome was 90-day mortality.

**Results:** In patients with CAP, there were 109/283 (38.5%) deaths at 90-day in the hydrocortisone plus fludrocortisone arm and 143/279 (51.3%) in the placebo arm (RR [95% CI]: 0.75 [0.62; 0.91] P = 0.002). In patients without CAP, there were 148/319 (46.4%) deaths at 90-day in the hydrocortisone and fludrocortisone arm and 157/329 (47.7%) in the placebo arm (RR [95% CI]: 0.97 [0.83; 1.14] P = 0.73). There was a significant statistical heterogeneity in corticosteroids effect on 90-day mortality across subgroups with or without CAP (Breslow-Day homogeneity test P = 0.045).

**Conclusion:** Hydrocortisone plus fludrocortisone was associated with reduced 90-day mortality in community acquired pneumonia associated with septic shock.

**Reference 1**

Annane D et al. NEJM 2018.

**Compliance with ethics regulations:** Yes in clinical research.

## CO-74 Relationship between micro aspiration and ventilator-associated events in mechanically ventilated ICU patients: a post hoc analysis of the BESTCUFF trial

### Guillaume Millot^1^, Hélène Behal^1^, Emmanuelle Jaillette^1^, Julien Labreuche^1^, Christophe Girault^2^, Guillaume Brunin^3^, Isabelle Alves^4^, Franck Minacori^5^, Hugues Georges^6^, Patrick Herbecq^7^, Farid Zerimech^1^, Saad Nseir^1^

#### ^1^Centre Hospitalier Universitaire de Lille, Lille, France; ^2^Centre Hospitalier Universitaire de Rouen, Rouen, France; ^3^Centre Hospitalier de Boulogne-sur-Mer, Boulogne-Sur-Mer, France; ^4^Centre Hospitalier de Valenciennes, Valenciennes, France; ^5^Hôpital Saint-Philibert, Lomme, France; ^6^Centre Hospitalier de Tourcoing, Tourcoing, France; ^7^Centre Hospitalier de Roubaix, Roubaix, France

##### **Correspondence:** Guillaume Millot (millot.gui@gmail.com)

*Annals of Intensive Care *2013, **13(Suppl 1)**:CO-74

**Rationale:** The relationship between ventilator-associated events (VAE) and micro aspiration in intubated patients has not been studied to this day. The objective was to study the relationship between abundant micro aspiration of oropharyngeal secretions or gastric content and the incidence of VAE.

**Patients and methods/materials and methods:** This was a post hoc analysis of the BESTCUFF study, which was a multicenter, cluster randomized, cross-over, controlled, open-label trial conducted in French intensive care units (ICU) from June 2014 to October 2015 to study the impact of tracheal tube cuff shape on micro aspiration (1). In adult patients ventilated for over 48 h, tracheal aspirates were sampled repeatedly for 48 h following enrollment, with measurement of pepsin and amylase in said samples. VAE were identified using NHSN VAE criteria (2) based on PEEP or FiO2 variations compared to stable parameters in previous days. The primary outcome was the impact of overall abundant micro aspiration on the incidence of VAE using a survival analysis, adjusted for potential confounding factors.

**Results:** Among 326 patients included in the BESTCUFF study, at least one measurement of either pepsin or amylase was available for 261 patients, of which 31 (11.9%) developed VAE, with an overall mean age of 62.7 ± 14.7, a majority of male patients (164, 62.8%), a median SAPS II score of 50 [40–61], a median SOFA score of 8 [5–11]. Patients were mainly admitted for acute respiratory failure (117, 44.8%). There was no statistically significant impact of overall abundant micro aspiration on the incidence of VAE at day 28 (adjusted hazard ratio (HR): 1.55 [0.46–5.17], p = 0.48) nor of gastric abundant micro aspiration (adjusted HR: 1.24 [0.61–2.53], p = 0.55) or oropharyngeal abundant micro aspiration (adjusted HR: 1.07 [0.47–2.42], p = 0.88). Similarly, we could not find an association between overall abundant micro aspiration and day-28 mortality (adjusted HR: 0.79 [0.35–1.73], p = 0.55), 28-day ICU length-of-say (adjusted HR: 1.21 [0.74–1.98], p = 0.44) and day-28 duration of mechanical ventilation (adjusted HR: 1.12 [0.75–1.66]).

**Conclusion:** In this post hoc analysis, there was no significant association between abundant micro aspiration and the incidence of VAE, nor on secondary outcomes including mortality, ICU length-of-stay and duration of mechanical ventilation. Given the small amount of patients who presented with VAE in this study, the analysis might have been underpowered. A dedicated study enrolling more patients could be required to rule out a relationship between those events.

**Reference 1**

Jaillette, E., Girault, C., Brunin, G. et al. Impact of tapered-cuff tracheal tube on microaspiration of gastric contents in intubated critically ill patients: a multicenter cluster-randomized cross-over controlled trial. Intensive Care Med 43, 1562–1571.

**Reference 2**

Magill, S., Klompas, M., Balk, R. et al. Developing a New, National Approach to Surveillance for Ventilator-Associated Events. Am J Crit Care 22 (6), 469–473 (2013). https://doi.org/10.4037/ajcc2013893

**Compliance with ethics regulations:** Yes in clinical research.

## CO-75 Diagnostic value of PCR for Pneumocystis jirovecii pneumonia in non-HIV immunocompromised patients in critical care

### Maxime Vaconsin^1^, Michael Thy^1^, Julien Dessajan^1^, Paul-Henri Wicky^2^, Etienne De Montmollin^1^, Romain Sonneville^1^, Lila Bouadma^1^, Christine Bonnal^1^, Jean-François Timsit^1^

#### ^1^Service de Médecine Intensive Réanimation, Hôpital Bichat, Paris, France; ^2^Institut mutualiste Montsouris, Paris, France

##### **Correspondence:** Maxime Vaconsin (maximevaconsin@hotmail.fr)

*Annals of Intensive Care *2013, **13(Suppl 1)**:CO-75

**Rationale:** Pneumocystis jiroveccii is a ubiquitous fungus responsible for Pneumocystis pneumonia (PCP) in immunocompromised patients. As the number of non-HIV immunocompromised patients escalates in intensive care units, predominantly admitted for acute respiratory failure, identifying fungal infection is crucial. According to a recent systematic review, no data was published on the diagnostic value of PCR in the specific subgroup of HIV-negative immunocompromised patients in critical care. The diagnosis of PCP is challenged by cysts scarcely recovered from respiratory samples in this population, and few DNA copies often found in healthy subjects. Also, the added value of ß-D-glucan (BDG) assays on blood samples in this population is imperfectly known.

Our aim is to describe the non-HIV critical care population with a positive Pneumocystis PCR, and to evaluate its relevance to diagnose PCP.

**Patients and methods/materials and methods:** Data were collected retrospectively in one ICU specialized in infectious diseases between 2013 to 2022. Non-HIV patients with a positive Pneumocystis PCR were divided into three groups at the time of care, confirmed PCP, uncertain PCP and ruled-out PCP, according to factors like the presence of cysts, radiographic findings, or the likelihood of other diagnoses. We compared the PCR cycles and BDG assays of patients in each of these groups.

**Results:** Forty-four non-HIV patients had a positive Pneumocystis PCR, 18 were categorized as having a confirmed PCP, 10 as uncertain PCP, and 16 as ruled-out PCP. The main characteristics of the patients can be found in the attached table. Immunosuppression was mostly secondary to autoimmune conditions (45%) and solid organ transplantation (20%). No clinical or biological characteristics were significantly different between groups, except cycle threshold (CT) and BDG values. PCR < = 29 cycles was the best diagnostic threshold, with an area under ROC curve of 0,845 (95% IC = [0,674–0,948], p < 0.001), a sensitivity of 78% (95% IC = [52–94]), and a specificity of 79% (95% IC = [49–95]). BDG > 80 pg/ml increased the diagnostic value in this population. Cysts were only found in respiratory samples of 7 patients. From the 16 patients in the ruled-out PCP group, 11 received no treatment and did not later develop PCP.

**Conclusion:** Diagnosing PCP in non-HIV patients can be challenging. Pneumocystis PCR can be positive in immunocompromised patients who do not have PCP. Using a threshold of 29 cycles and combining it with a BDG > 80 pg/ml increases its diagnostic value.

**Reference 1**

Senécal et al., Non-invasive diagnosis of Pneumocystis jirovecii pneumonia: a systematic rev.

**Reference 2**

Giacobbe et al., Performance of Existing Definitions and Tests for the Diagnosis of Invasive Fungal Dise.

**Compliance with ethics regulations:** Yes in clinical research.

**Figure 1 (abstract CO-75):**
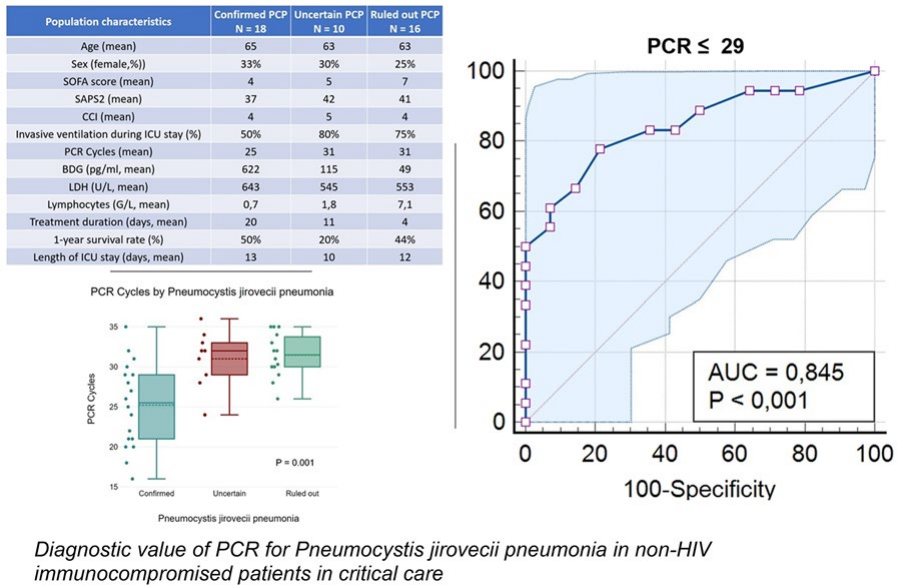
Diagnostic value of PCR for Pneumocystis jirovecii pneumonia in non-HIV immunocompromised patients in critical care

## CO-76 De-escalation of β-lactams in wild-type AmpC-producing Enterobacterales ventilator associated pneumonia in critically ill patients: a prospective multicenter study

### Matthieu Petit^1^, Frank Bidar^2^, Quentin Fosse^3^, Lucie Lefevre^4^, Marine Paul^5^, Tomas Urbina^6^, Paul Masi^7^, Florent Bavozet^8^, Jérémie Lemarié^9^, Etienne De Montmollin^10^, Chloé Andriamifidy-Berti^11^, Julien Dessajan^12^, Benjamin Zuber^13^, Lara Zafrani^14^, Edwige Peju^15^, Paris Meng^16^, Liliane Charrier^17^, Loïc Le Guennec^18^, Marie Simon^20^, Luc Haudebourg^19^, Guillaume Geri^21^

#### ^1^Service de Médecine Intensive Réanimation, Hôpital Ambroise Paré, APHP, Boulogne-Billancourt, France; ^2^Département d'Anesthésie-Réanimation, Service de Réanimation Médico-Chirurgicale, Hôpital Edouard Herriot, Hospices Civils de Lyon, Lyon, France; ^3^Service de Médecine Intensive Réanimation, Hôpital Bicêtre, APHP, Le Kremlin-Bicêtre, France; ^4^Service de Médecine Intensive Réanimation, Institut de Cardiologie, Hôpital Pitié Salpêtrière, APHP, Paris, France; ^5^Service de Réanimation, Centre Hospitalier de Versailles, Hôpital André Mignot, Le Chesnay-Rocquencourt, France; ^6^Service de Médecine Intensive Réanimation, Hôpital Saint-Antoine, APHP, Paris, France; ^7^Service de Médecine Intensive Réanimation, Hôpital Henri Mondor, APHP, Créteil, France; ^8^Service de Médecine Intensive Réanimation, Centre hospitalier Victor Jousselin, Dreux, France; ^9^Service de Médecine Intensive Réanimation, Centre Hospitalier Universitaire de Nantes, Nantes, France; ^10^Service de Médecine Intensive Réanimation, Hôpital Bichat, APHP, Paris, France; ^11^Service de Médecine Intensive Réanimation, Centre hospitalier intercommunal de Poissy-Saint-Germain-en-Laye, Poissy, France; ^12^Service de Médecine Intensive Réanimation, Hôpital Tenon, APHP, Paris, France; ^13^Service de Réanimation polyvalente, Hôpital Foch, Suresnes, France; ^14^Service de Médecine Intensive Réanimation, Hôpital Saint-Louis, APHP, Paris, France; ^15^Service de Médecine Intensive Réanimation, Hôpital Cochin, APHP, Paris, France; ^16^Service de Réanimation, Centre Hospitalier Intercommunal Robert Ballanger, Aulnay-Sous-Bois, France; ^17^Service de Médecine Intensive Réanimation, Centre Hospitalier Public du Cotentin, Cherbourg-En-Cotentin, France; ^18^Service de Médecine Intensive Réanimation Neurologique, Hôpital Pitié Salpêtrière, APHP, Paris, France; ^19^Service de Médecine Intensive Réanimation R3S, Hôpital Pitié Salpêtrière, APHP, Paris, France; ^20^Service de Médecine Intensive Réanimation, Hôpital Edouard Herriot, Hospices Civils de Lyon, Lyon, France; ^21^Service de Réanimation Médico-Chirurgicale, Clinique Ambroise Paré, Neuilly-Sur-Seine, France

##### **Correspondence:** Matthieu Petit (matthieu.petit@aphp.fr)

*Annals of Intensive Care *2013, **13(Suppl 1)**:CO-76

**Rationale:** Ventilator associated pneumonia (VAP) due to wild-type AmpC-producing Enterobacterales (wtAE) is frequent in critically ill patients. Despite a low level of evidence, definitive treatment with cefepime is recommended to treat wtAE VAP over the use of third generation cephalosporin (3GC) or piperacillin, because the latter may fail or induce selection of AmpC-overproducing mutants. Our aim was to assess the risk of treatment failure and relapse of VAP of a strategy of β-lactams de-escalation in these patients.

**Patients and methods/materials and methods:** Our prospective, observational study included consecutive wtAE VAP patients in 20 French intensive care units between February 2021 and June 2022. The primary outcome was treatment failure, defined as an inadequate response to antimicrobial therapy leading to death or to the necessity to switch to a broader-spectrum antibiotic at day-7. Relapse of infection was collected as a secondary outcome and defined as a new AE pneumoniae within the first 28 days after inclusion. De-escalation of β-lactams was defined as definitive treatment by piperacillin or 3GCs. Characteristics were compared across de-escalation and full-treatment groups. Factors associated with the primary outcome were evaluated using a multivariable mixed logistic regression was performed to take into account the center effect.

**Results:** Two-hundred and eighty patients were included. Patients were mostly ventilated for respiratory failure (66%), and 54% had COVID-19 pneumonia. At VAP diagnosis, the median SOFA was 7 [5;10] and 41 (15%) patients had ECMO. Enterobacter cloacae was the most prevalent specie (30%), followed by Klebsiella aerogenes (25%) and Serratia marcescens (22%). De-escalation was performed in 87 (31%) patients while 193 (69%) received a full-treatment with broad spectrum antibiotics. Treatment failure was similar in the de-escalation and full-treatment group (26 vs. 34%, p = 0.32). At day-28, relapse was as frequently observed in the de-escalation group as in the full-treatment group (31 vs. 24%). In multivariable analysis, de-escalation to 3GCs or piperacillin was not associated with treatment failure or relapse. There was no difference in vital status at day-28, mechanical ventilation duration or ICU length of stay between the two groups. ESBL acquisition or Clostridium difficile infection during hospitalization were also similar.

**Conclusion:** In patients presenting wtAE VAP, treatment failure and risk of relapse at day 28 were not higher when a de-escalation of β-lactams was used. A randomized controlled trial could provide more data in support of such a strategy.

**Compliance with ethics regulations:** Yes in clinical research.

## CO-77 Performance and impact on antimicrobial therapy of rapid multiplex PCR in ventilated hospital-acquired pneumonia in ICU patients colonized with extended-spectrum beta-lactamase-producing Enterobacteriaceae: a prospective monocentric study.

### Pierre Bay^1^, Vincent Fihman^1^, Paul-Louis Woerther^1^, Ségolène Gendreau^1^, Romain Arrestier^1^, Pascale Labedade^1^, Elsa Moncomble^1^, Antoine Gaillet^1^, Guillaume Carteaux^1^, Nicolas De Prost^1^, Armand Dessap^1^, Keyvan Razazi^1^

#### ^1^Service de Médecine Intensive Réanimation, CHU Henri Mondor, Créteil, France

##### **Correspondence:** Pierre Bay (pierre.bay@aphp.fr)

*Annals of Intensive Care *2013, **13(Suppl 1)**:CO-77

**Rationale:** Antibiotherapy stewardship for suspected ventilator-associated pneumonia (VAP)/ventilated hospital-acquired pneumonia (vHAP) in ESBL-E carriers is challenging. BioFire® FilmArray® Pneumonia plus Panel (mPCR) allows the detection of 18 bacteria, 9 viruses, and 7 antibiotic resistance genes, including blaCTX-M, the most common ESBL gene.

**Patients and methods/materials and methods:** This monocentric, prospective study was conducted from March 2020 to August 2022. First, we evaluated the performance of mPCR on ESBL-E related pneumonia detection through blaCTX-M on respiratory samples performed for suspicion of ventilated hospital-acquired pneumonia. Over the study period, 228 mPCR were performed for a suspected VAP/hVAP, 41 in patients with ESBL-E rectal carriage and 187 in non-carriers. Second, we assessed the impact on antibiotherapy stewardship of mPCR, in 95 ESBL-E carriers (mPCR performed n = 22 or non-performed n = 73) with confirmed ventilated hospital-acquired pneumonia (confirmed from quantitative cultures of lower respiratory tract secretions sampled).

**Results:** mPCR performance: Among ESBL-E carriers, mPCR was positive for 24 patients (59%) including 15 (62%) with blaCTX-M detected. Among carriers, 9 (22%) developed an ESBL-E related pneumonia. The negative predictive value of mPCR to identify ESBL-E related pneumonia was 100% in carriers. In patients without ESBL-E carriage, sensitivity and positive predictive value of mPCR to identify ESBL-E related pneumonia were respectively 100% (95% CI 2–100) and 33% (95% CI 11–66). Antibiotic assessment: ESBL-E carriers developed 95 VAP/vHAP, 47 (49%) episodes were related to ESBL-E, and 24 (25%) to carbapenem-resistant bacteria. A mPCR-based approach was independently associated with an adequate empiric antibiotic therapy in the multivariable logistic regression (adjusted odds ratio (aOR) 7.8, 95% confidence interval (95% CI) 2–30.9, p = 0.003) and the propensity-weighted (aOR 10.1, 95% CI 2.7–37.9, p = 0.001) models.

**Conclusion:** mPCR showedgood microbiological performances with a negative predictive value of 100% to rule out the diagnosis of ESBL-E related VAP/vHAP. A mPCR-based antibiotherapy stewardship was associated with increased adequate empiric antibiotic therapy. mPCR on respiratory sample seems to be a promising tool in ESBL-E carriers with suspected VAP/vHAP.

**Compliance with ethics regulations:** Yes in clinical research.

**Figure 1 (abstract CO-77):**
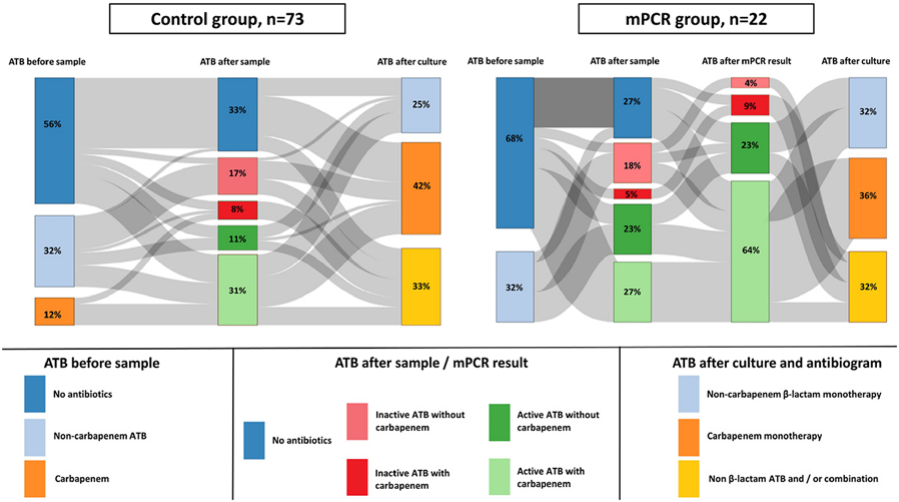
Sankey of diagram of antibiotic stewardship according to the use of mPCR in ESBL-E carriers with definite VAP/vHAP

## Flash communications

## FC-001 Hemodynamic and respiratory effects of alveolar recruitment

### Chihebeddine Romdhani^1^, Aïcha Rebai^1^, Imen Chaieb^2^, Walid Samoud^1^, Khadija Bahrini^1^, Hédi Gharsallah^1^, Mustapha Ferjani^1^

#### ^1^Hôpital Militaire Principal d'Instruction de Tunis, Tunis, Tunisie; ^2^Hôpital Militaire, Gabès, Tunisie

##### **Correspondence:** Aïcha Rebai (aicha.rebai@fmt.utm.tn)

*Annals of Intensive Care *2013, **13(Suppl 1)**:FC-001

**Rationale:** The primary cause of gas exchange abnormalities during anesthesia is pulmonary atelectasis. The aim of our study is to assess the potential effects of the recruitment maneuver on blood pressure and respiratory parameters. The primary end point was the change in lung compliance after alveolar recruitment.

**Patients and methods/materials and methods:** This is a prospective observational study conducted in the operating room of our Military Hospital. Patients from ASA1 to ASA 3 proposed exclusively for elective major abdominal surgery were included in the current study. The alveolar recruitment maneuver was performed to characterize a continuous positive airway pressure of 30 cm H_2_O for 30 s. Then, respiratory and hemodynamic parameters before, during, and after alveolar recruitment were measured.

**Results:** Thirty-six patients underwent 79 alveolar recruitment maneuvers. No significant difference was detected in heart rate during and after alveolar recruitment (p = 0.63). In addition, we found a significant decrease in the minimum systolic blood pressure values during alveolar recruitment as compared to the values measured before and after alveolar recruitment (100 mmHg [83; 113],104 mmHg [94; 121], and 102 mmHg [90; 113] respectively. Diastolic blood pressure did not drop during recruitment (p = 0.16). However, we detected a significant decrease in mean arterial pressure during alveolar recruitment as compared to values detected before and after the alveolar recruitment maneuver (68 mmHg [58; 80], 77 mmHg [67; 86], and 76 mmHg [66; 84] respectively). The pulse pressure variation values were significantly higher during alveolar recruitment (p = 0.0001). Evenly, lung and tele-expiratory CO 2 pressure compliance were significantly increased after the alveolar recruitment maneuver.

**Conclusion:** The alveolar recruitment maneuver highly improves lung compliance. This ventilation strategy was relatively well tolerated on the hemodynamic level. However, further studies are necessary to specify the appropriate protocols for this approach.

**Compliance with ethics regulations:** Yes in clinical research.

**Figure 1 (abstract FC-001):**
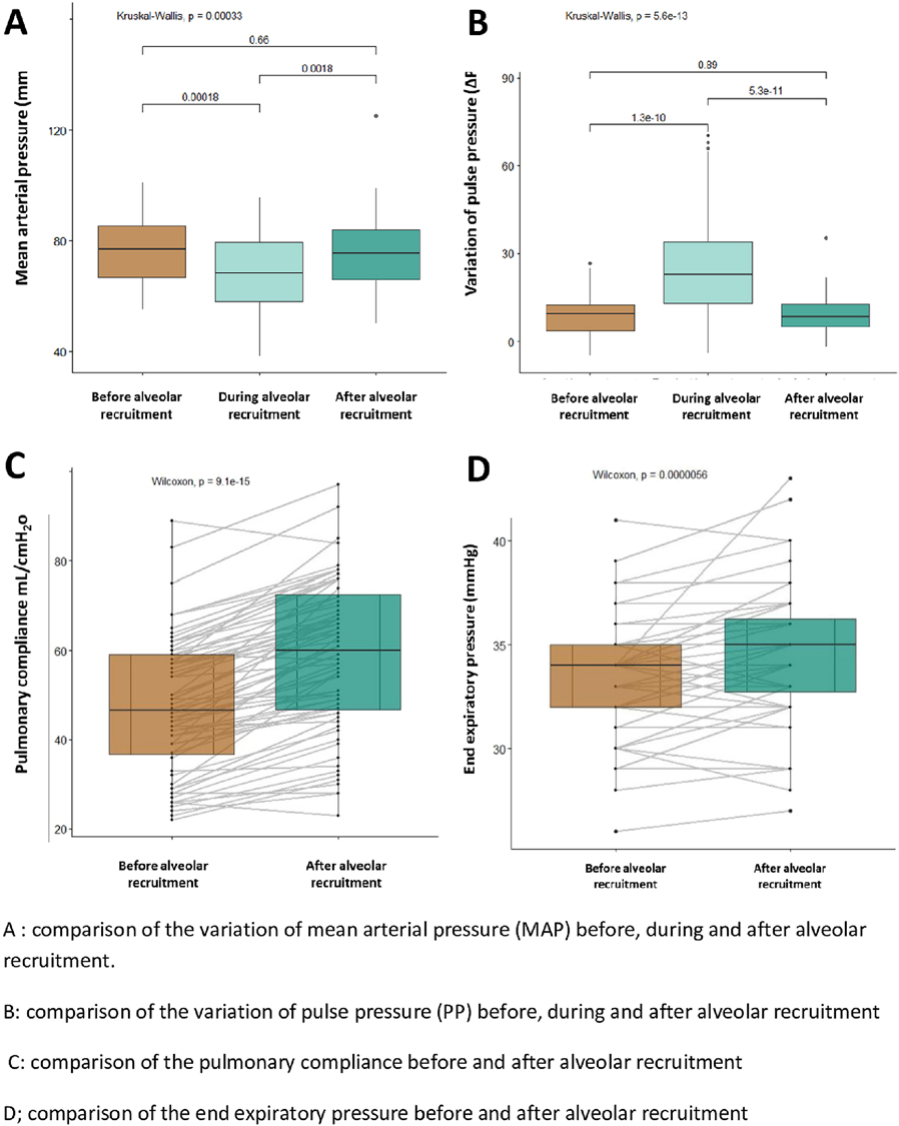
Hemodynamic and respiratory effects of alveolar recruitment

## FC-002 Radial strain accurately depicts paradoxical septal motion in ventilated patients with acute respiratory distress syndrome using a machine learning approach

### Bruno Evrard^1,2^, Jean-Baptiste Woillard^2,3,4,5^, Annick Legras^6^, Mysilias Bouaoud^6^, Maeva Gourraud^6^, Antoine Humeau^3^, Marine Goudelin^1,2^, Philippe Vignon^1,2,4,7^

#### ^1^Réanimation polyvalente, CHU Dupuytren, Limoges, France; ^2^Inserm CIC 1435, CHU Dupuytren, Limoges, France; ^3^Pharmacologie et Transplantation, Inserm U1248, Université de Limoges, Limoges, France; ^4^Faculté de Médecine, Université de Limoges, Limoges, France; ^5^Service de Pharmacologie, Toxicologie et Pharmacovigilance, CHU Dupuytren, Limoges, France; ^6^Médecine Intensive Réanimation, CHRU Bretonneau, Tours, France; ^7^Inserm UMR1092, CHU Dupuytren, Limoges, France

##### **Correspondence:** Bruno Evrard (bruno.evrard@chu-limoges.fr)

*Annals of Intensive Care *2013, **13(Suppl 1)**:FC-002

**Rationale:** Accurate diagnosis of acute cor pulmonale is key since it is prognostic in acute respiratory distress syndrome (ARDS). Identification of paradoxical septal motion is highly subjective. LV radial strain has not yet been used to help identification of paradoxical septal motion. Machine learning approaches provide individual prediction which can be reused for further assessment. We aimed to evaluate the accuracy of left ventricular (LV) radial strain using a supervised machine learning approach for the diagnosis of paradoxical septal motion, using conventional two-dimensional assessment as reference.

**Patients and methods/materials and methods:** In this retrospective study, a multicenter prospective cohort of ventilated patients with moderate-to-severe acute respiratory distress syndrome (ARDS) related to COVID-19 (n = 327) was used as the train dataset (75% of the cohort) and as test dataset (25% of the cohort). A second multicenter cohort of patients with moderate-to-severe ARDS was used as external validation dataset (n = 85). A supervised machine learning approach was compared with conventional visual identification of paradoxical septal motion in the two-dimensional short-axis view used as reference by two independents abstractors. The same digital loop used for conventional identification were used for LV short-axis strain assessment. Data were extracted and converted to perform data engineering using previous described method (Evrard B preprint 2023). Four algorithms (Extreme gradient boosting (XGBoost), Generalized Linear Model-Lasso (GLM-Lasso), Support Vector Machines (SVM) and random forest (RF)) were used and benchmarked to develop a diagnostic algorithm using 20 variables featured from LV radial strain curves assessment which were previously described for paradoxical septal motion diagnosis (ref).

**Results:** In the train set, the accuracy to identify paradoxical septal motion obtained by tenfold cross validation reached 0.73, 0.77, 0.78 and 0.75 for XG Boost, GLM-Lasso, SVM and RF, respectively. In the test set, similar performances were obtained (Figure 1A) with the best performances exhibited for the SVM and GLM-Lasso algorithm (area under the receiver operating characteristic curve [AUC], 0.84 and 0.90, respectively). Finally, all the models accurately classified the patients in the external validation (Figure 1B) with the best performances obtained by SVM algorithm (AUC, 0.85).

**Conclusion:** LV radial strain promises to accurately diagnose paradoxical septal motion with a machine learning approach.

**Compliance with ethics regulations:** Yes in clinical research.

**Figure 1 (abstract FC-002):**
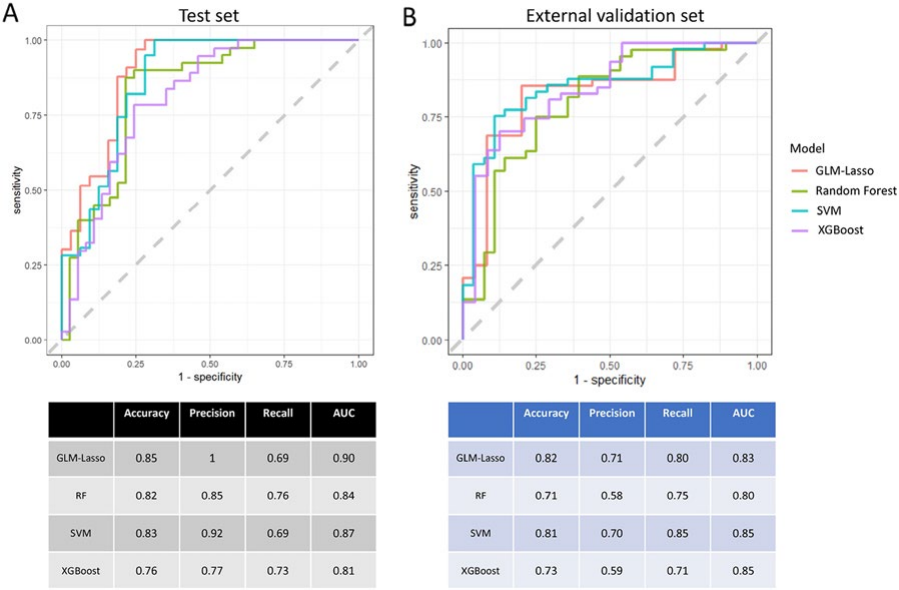
Receiving operator curve (ROC) with accuracy, precision and recall in the test set (A) and in the validation set (B) of the four Machine learning algorithms

## FC-003 Left ventricular radial strain depicts and quantitatively assesses the severity of paradoxical septal motion in ventilated patients with the acute respiratory distress syndrome

### Bruno Evrard^1,2^, Jean-Baptiste Woillard^2,3,4,5^, Annick Legras^6^, Misylias Bouaoud^6^, Maeva Gourraud^6^, Antoine Humeau^3^, Marine Goudelin^1,2^, Philippe Vignon^1,2,4,7^

#### ^1^Réanimation polyvalente, CHU Dupuytren, Limoges, France; ^2^Inserm CIC 1435, CHU Dupuytren, Limoges, France; ^3^Pharmacologie et Transplantation, Inserm U1248, Université de Limoges, Limoges, France; ^4^Faculté de Médecine, Université de Limoges, Limoges, France; ^5^Service de Pharmacologie, Toxicologie et Pharmacovigilance, Université de Limoges, Limoges, France; ^6^Médecine Intensive Réanimation, CHRU Bretonneau, Tours, France; ^7^Inserm UMR 1092, CHU Dupuytren, Limoges, France

##### **Correspondence:** Bruno Evrard (bruno.evrard@chu-limoges.fr)

*Annals of Intensive Care *2013, **13(Suppl 1)**:FC-003

**Rationale:** Accurate diagnosis of acute cor pulmonale (ACP) is key since it is prognostic in patients ventilated for an acute respiratory distress syndrome (ARDS). ACP is characterized by a paradoxical septal motion (PSM) which identification using two-dimensional echocardiography is highly subjective. Left ventricular (LV) radial strain has not yet been used to help the identification and assess the severity of PSM. We sought to describe LV radial strain changes related to PSM in patients at risk of sustaining ACP according to its severity.

**Patients and methods/materials and methods:** This prospective bicentric study included patients ventilated for an ARDS related to COVID-19 who were assessed using transesophageal echocardiography between March 2020 and June 2021. Two-dimensional transgastric short-axis view at mid-papillary level was used to grade septal motion: normal (grade 0), transient end-systolic septal flattening (grade 1), prolonged end-systolic septal flattening or reversed septal curvature (grade 2). LV radial strain analysis was performed off-line on the same digital loops and six LV segments were distinguished: mid-infero-septal and mid antero-septal, their opposite segments (mid-infero-lateral and mid-antero-lateral, respectively), and the remaining two segments (mid-anterior and mid-inferior). After having confirmed visually that LV radial strain curves were altered in certain segments when a PSM was present (Figure), we performed feature engineering. We calculated the “time-to-peak” defined as the time lag required to reach the maximal value of strain, which was normalized by the length of cardiac cycle and the “partial area under segmental strain curves” which was calculated as the area under each LV segmental strain curve between 33 and 66% of the cardiac cycle length (time period selected on graphical examination of the strain curves where most alterations of strain pattern occurred) to standardize the measurement.

**Results:** Overall, 318 echocardiography examinations performed in 184 patients were analyzed. Two-dimensional assessment identified a grade 1 and a grade 2 PSM at end-systole in 106 (33%) and 43 (14%) examinations, respectively. When compared with mid-anterior or mid-inferior segments, the time-to-peak of mid-septal and mid-lateral segments occurred significantly later in systole and this delay gradually increased with the grade of PSM (Figure). Similarly, the area under the strain curve of mid-septal and mid-lateral segments increased significantly with the grade of PSM, compared with mid-anterior or mid-inferior segments (Figure).

**Conclusion:** LV radial strain can objectively depict PSM and quantitatively assess its severity.

**Compliance with ethics regulations:** Yes in clinical research.

**Figure 1 (abstract FC-003):**
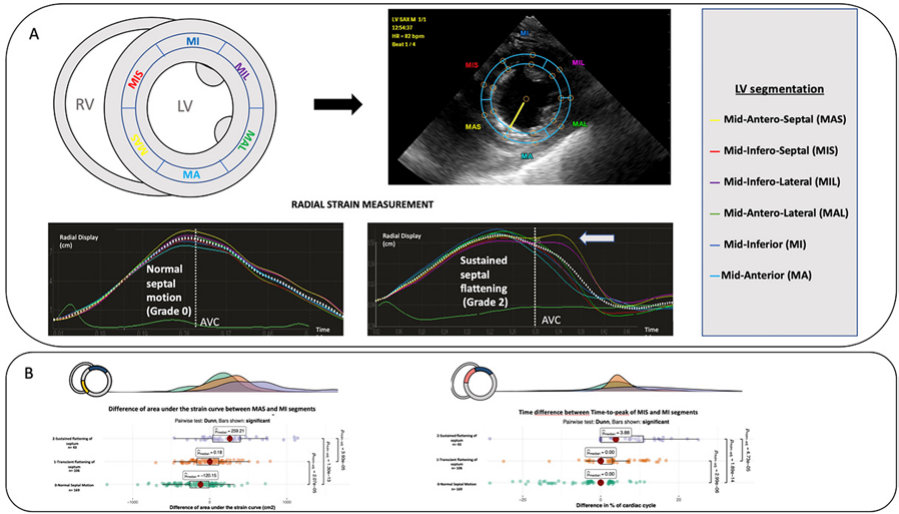
A. Schematic representation of LV segmentation for strain analysis with illustrative examples of a normal septal pattern of contraction (grade 0) and of sustained septal flattening (grade 2). B. Boxplots with density plots of main results

## FC-004 Effects of positive end-expiratory pressure on coronary flow in patients with acute respiratory distress syndrome

### Yoann Zerbib^1^, Rosalie Schoux^1^, Jack Richecoeur^1^, Clément Brault^1^, Thomas Bradier^1^, Julien Maizel^1^, Michel Slama^1^

#### ^1^CHU Amiens, Amiens, France

##### **Correspondence:** Rosalie Schoux (rschoux@gmail.com)

*Annals of Intensive Care *2013, **13(Suppl 1)**:FC-004

**Rationale:** During Acute respiratory distress syndrome (ARDS), high positive end expiratory pressure (PEEP) may have multiple and complex hemodynamic deleterious effects. To aim of this study was to evaluate the effect of high PEEP on coronary blood flow (CBF) measured at pressure 5 and 15 cmH2O.

**Patients and methods/materials and methods:** Thirty-two patients have been included. A multicenter prospective observational study of ARDS patients under mechanical ventilation underwent a transthoracic (TTE) and transesophageal echocardiography (TEE).

**Results:** Compared to PEEP 5 cmH2O, respiratory system compliance (Crs) (32[25–45]vs38 [27–57] mL/cm H2O; p < 0.0001), mean arterial pressure (MAP) (77[67–84] vs 83 [73–90] mmHg; p < 0.0001) and cardiac output (CO) (4.6 [3.8–5.8] vs 5.1 [3.8–5.8] l/mn, p = 0.0009) were lower at 15cmH2O. Left ventricular (LV) diastolic function assessed using early mitral annulus velocity (6 [5–8] vs 8 [7–11] cm/s, p = 0.004) and right ventricular (RV) systolic function assessed using systolic tricuspid annulus velocity (11[9–14] vs 13[11–15] cm/s, p = 0.02) were impaired at PEEP 15 cmH2O. Assessed using Doppler velocity time integral, diastolic and systolic left CBF (16.2[11.9–22] vs 18.1[13.1–23.2] cm/s, p = 0.03) (5.1[3.4–7.7] vs 6.3[4.4–8.7] cm/s, p = 0.006) and systolic right CBF (7.5[5.3–10.6] vs 9.6[8–14.6] cm/s, p = 0.02) were lower at PEEP 15cmH2O compared to PEEP 5cmH2O. Impairment of left CBF was associated with Crs and CO impairement.

**Conclusion:** Left diastolic and systolic and right systolic CBF were lower at PEEP 15cmH2O in comparison with PEEP 5 cmH2O and associated with decreased Crs, CO, MAP, and impaired LV diastolic function and RV systolic function.

**Compliance with ethics regulations:** Yes in clinical research.

## FC-005 Hemodynamic improvement after maximal recruitment in patients with moderate to severe ARDS ventilated with high PEEP

### Alexis Lambour^1^, Yoann Zerbib^1^, Clément Brault^1^, Julien Maizel^1^, Michel Slama^1^

#### ^1^CHU Amiens Picardie, Amiens, France

##### **Correspondence:** Alexis Lambour (alexis.lambour@hotmail.fr)

*Annals of Intensive Care *2013, **13(Suppl 1)**:FC-005

**Rationale:** Mechanical ventilation with high PEEP in ARDS patients could induce cardiac dysfunction mainly due to the cardiac effect of lung overdistension. We hypothesized that lung recruitment maneuver (LRM) by opening and preventing lung over distension may improve the hemodynamic tolerance of high PEEP. Then, we decided to apply the same high PEEP to ARDS patients before and after recruitment maneuver to assess hemodynamics.

**Patients and methods/materials and methods:** Thirty-two patients with moderate to severe ARDS hospitalized in our intensive care unit. Respiratory and hemodynamic evaluation by transthoracic echocardiography were done at the same incremental (before LRM) and decremental (after LRM) PEEP = 25cmH_2_O and driving pressure = 15cmH_2_O. Patients were separated into two groups based on median compliance gain (“responders” and “non-responders”).

**Results:** Left ventricular (LV) ejection fraction (EF) (58% [51.3–71] vs 52.6% [44.4–67.3]; p = 0.04), mitral annulus plane systolic excursion (1.29 [1.13–1.37] vs 1.01 [0.85–1.13]; p = 0.007) and the product of heart rate and aortic velocity time integral (a surrogate of cardiac output) (1600 [1310–1789] vs 1370 [1215–1689]; p = 0.02) were improved after LRM. After LRM, we observed a decrease in right ventricular (RV) to the LV ratio (RV/LV ratio 0.88 [0.79–1] vs 1.01 [0.93–1.1]; p = 0.01) and an improvement of RV systolic function (tricuspid annulus plane systolic excursion 1.93 [1.65-0.2.19] vs. 1.6 [1.24–1.8]; p = 0.0002). The subgroup analysis revealed an improvement in LVEF and a decreased RV/LV in the "responders" group, and not in the "non-responders" group.

**Conclusion:** LRM induced an improvement of the cardiac function in patients ventilated with high PEEP particularly in the responders group of patients.

**Compliance with ethics regulations:** Yes in clinical research.

## FC-006 The haemodynamic effects of PEEP depend on alveolar recruitability in patients with acute respiratory distress syndrome

### Julien Hagry^1^, Simone Cappio Borlino^1^, Christopher Lai^1^, Gaëlle Fouqué^1^, Marta Fasan^1^, Eduardo Rocca^1^, Rui Shi^1^, Tài Pham^1^, Jean-Louis Teboul^1^, Xavier Monnet^1^

#### ^1^Université Paris Saclay, AP-HP, Service de médecine intensive-réanimation, Hôpital Kremlin Bicêtre, Le Kremlin-Bicêtre, France

##### **Correspondence:** Simone Cappio Borlino (simone.cappio@unimi.it)

*Annals of Intensive Care *2013, **13(Suppl 1)**:FC-006

**Rationale:** During acute respiratory distress syndrome (ARDS), positive end-expiratory pressure (PEEP) might increase pulmonary vascular resistance (PVR). In theory, according to the U-shape of the relationship between PVR and lung volume, which nadir corresponds to the functional residual capacity, PEEP should increase PVR in case of alveolar overdistention and lower PVR if alveolar recruitment predominates. This has never been proven in patients with ARDS. We evaluated PVR changes during PEEP modifications depending on the potential of lung recruitability, estimated through the recruitment-to-inflation (R/I) ratio.

**Patients and methods/materials and methods:** In intubated ARDS patients, monitored by a pulmonary artery catheter, an esophageal balloon and transthoracic echocardiography, PEEP was lowered by 10 cmH_2_O from baseline. At the two levels of PEEP, hemodynamic, echocardiographic and respiratory variables were measured and preload dependance was evaluated through the passive leg raising test. An R/I ≥ 0.5 defined high recruitment potential (HRP) and R/I < 0.5 defined low recruitment potential (LRP).

**Results:** Forty measurements were performed in 14 patients (2 [1; 3] measures per patient): mean age was 65 ± 11 years, 12 (86%) were male and median (IQR) baseline PaO_2_/FiO_2_ was 110 (74; 124) mmHg. The R/I ratio was ≥ 0.5 (0.70 [0.59; 0.85]) in 23 (57%) measurements. PEEP was lowered from 15 (12; 15) to 5 (2; 5) cmH_2_O. By lowering PEEP, the PVR decreased in case of LRP (-25 [-30; -14]%) while they remained unvaried in case of HRP (0 [-11; 13]%, p < 0.0001) (figure). The gradient between mean pulmonary arterial pressure and pulmonary arterial occlusion pressure decreased in case of LRP (-8 [-18; -4]%) but increased in case of HRP (9 [0; 17]%, p = 0.0001). A similar effect was observed for right to left ventricular end-diastolic area ratio (-16 [-25; -14]% vs. 8 [-13; 20]%, respectively, p = 0.0008). The magnitude of PVR change induced by PEEP lowering was also correlated with R/I ratio (r = 0.52 [0.25; 0.71], p = 0.0006). In case of LRP, the variation in cardiac index induced by PEEP reduction was not different in preload dependent and preload non-dependent cases (23 [5; 32]% vs. -1 [-7; 26]%, respectively, p = 0.1).

**Conclusion:** Effects of PEEP on PVR in mechanically ventilated patients with ARDS might depend on alveolar recruitment. A significant risk of PVR increase exists when PEEP leads to pulmonary overdistention. This suggests that the potential of lung recruitment should be considered during PEEP titration, in order to better individualize respiratory support. The study is ongoing.

**Reference 1**

Chen L, Del Sorbo L, Grieco DL, et al. Potential for Lung Recruitment Estimated by the Recruitment-to-Inflation Ratio in Acute Respiratory Distress Syndrome. A Clinical Trial. Am J Respir Crit Care Med. 2020;201(2):178–187. https://doi.org/10.1164/rccm.201902-0334OC

**Compliance with ethics regulations:** Yes in clinical research.

**Figure 1 (abstract FC-006):**
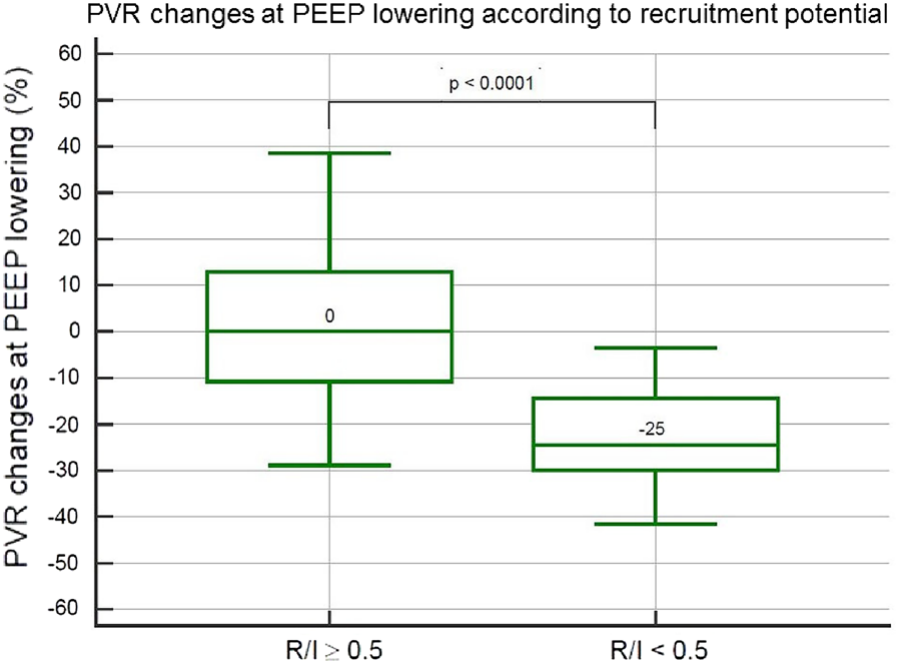
PVR changes at PEEP lowering according to recruitment potential

## FC-007 Effects of fluid loading and extravascular lung water on the total fluid content measured by bioimpedance

### Daniela Rosalba^1,2^, Rui Shi^2^, Chiara Bruscagnin^2^, Christopher Lai^2^, Gaëlle Fouqué^2^, Julien Hagry^2^, Jean-Louis Teboul^2^, Xavier Monnet^2^

#### ^1^Università del Piemonte Orientale "Amedeo Avogadro", Novara, Italie ; ^2^Université Paris-Saclay, AP-HP, Service de médecine intensive-réanimation, Hôpital de Bicêtre, Paris, France

##### **Correspondence:** Daniela Rosalba (danielarosalba245@gmail.com).

*Annals of Intensive Care *2013, **13(Suppl 1)**:FC-007

**Rationale:** The Starling system is a non-invasive hemodynamic monitoring device that measures cardiac output through bioreactance. Through bioimpedance, it also measures the mean transthoracic electric impedance (Z0), from which the thoracic fluid content (TFC) can be derived, which should reflect extravascular lung water, fluid in the large thoracic vessels and the cardiac cavities. We compared TFC to the extravascular lung water indexed for ideal body weight (EVLWI) and the global end-diastolic volume index for body surface (GEDVI) estimated by transpulmonary thermodilution (TPTD).

**Patients and methods/materials and methods:** A group of patients with shock and another one with acute respiratory distress syndrome (ARDS) were included. All the patients were equipped with calibrated TPTD device and the Starling system. Variables measured by TPTD and by the Starling system were collected at the same time before and after volume expansion (500 mL saline) for patients with shock, and once a day during the course of the disease for ARDS patients.

**Results:** In the 42 patients with shock receiving a fluid bolus, TPTD-derived cardiac output increased by 0.44 [− 0.16 to 1.06] (22.5%) L/min/m2 and bioreactance-derived cardiac output by 0.22 [− 0.72 to 1.16] (11.8%) L/min/m2. GEDVI increased by 43.8 [− 11.9–99.5] (7.6%) ml/m2 and TFC by 2.4 [− 0.3 to 5.1] (3%). EWLVI decreased by 0.02 [− 1.83 to 1.79] (0.25%) ml/kg. There was no correlation between the fluid-induced changes in GEDVI or in EWLVI on the one side and in TFC on the other (p = 0.95 and p = 0.58). In the 23 patients with ARDS, 124 measurements were performed. Between two successive measurements, EVLWI changed by − 0.1 (6.3%) ml/kg and the TFC by − 0.4 (4.6%). There was no correlation between the changes in EVLWI and in TFC between successive timepoints (p = 0.42). Considering all measurement together (n = 166), the was no correlation between the absolute values of GEDVI and TFC (p = 0.16), EVLWI and TFC (p = 0.06) and the sum of GEDVI and EVLWI on the one side and TFC on the other (p = 0.19). Still considering all measurements, there was no correlation between the bias between the sum of GEDVI and EVLWI vs. TFC on the one side and the bias between TPTD- and bioreactance-derived cardiac output on the other (p = 0.29).

**Conclusion:** In patients with shock or ARDS, there is no significant relationship between TFC measured by bioimpedance and GEDVI or EVLWI measured by TPTD, neither in absolute values, nor in relative changes.

**Compliance with ethics regulations:** Yes in clinical research.

## FC-008 Impact of Fluid Balance assessment on the occurrence of Primary graft Dysfunction in lung transplant recipients

### Denis Bontemps^1^, Benjamin Coiffard^1^, Christophe Guervilly^1^, Sami Hraiech^1^, Florence Daviet^1^

#### ^1^Assistance publique hôpitaux de Marseille/Hôpital Nord, Marseille, France

##### **Correspondence:** Florence Daviet (florence.daviet@ap-hm.fr)

*Annals of Intensive Care *2013, **13(Suppl 1)**:FC-008

**Rationale:** Lung transplant is associated with poorer survival than that observed in other solid organ transplants. Primary graft dysfunction (PGD) and infections are the leading causes of death during the first month of transplantation. The main objective was to assess whether the Fluid balance of the first 24 h after lung transplantation had an impact on the occurrence of PGD at 72 h.

**Patients and methods/materials and methods:** This is an ambispective observational study including all adult patients who received a lung transplant in our hospital from November 1^st^ 2020. The primary endpoint was the occurrence of stage 3 PGD 72 h after lung transplantation, defined by the presence of pulmonary edema on the chest X-ray, associated with a PaO2/FiO2 ratio < 2001. The fluid balance was calculated by making the difference between fluid intake and fluid loss from the operating room to 24 h after clamp release from the second lung (corresponding to Day1) then every day for 72 h.

**Results:** We present the preliminary results on the first 30 patients included, transplanted between 1/11/2020 and 31/10/2021. They had a median age of 55 years (45–63), 53% were men. Half of the patients presented with PGD whatever the stage during the first 72 h, including 11 patients (37%) with stage 3 PGD. The median Fluid balance on Day 1 was 1278 ml (259–3087) and 8341 ml (5218–11,861) at 72 h. In univariate analysis, the total Fluid balance at Day 1 (3024 ml (675–6880) versus 678 ml (− 200 to 2323)), as well as the total Fluid balance at 72 h (11,945 ml (5853–14,595) versus 7596 ml (4516–10,415)) were significantly higher in patients with stage 3 PGD (p = 0.006 and 0.03 respectively) (Figure 1). The other main factors associated with the occurrence of stage 3 PGD were: the recipient's body mass index (p = 0.005), the total lung capacity on the recipient's pre-transplant Exploration Respiratory Tests (p = 0.008) and the worst Donor PaO2/FiO2 (p = 0.04). There was a positive correlation between PGD stage and total Fluid balance at Day 1 (r = 0.43, p = 0.019).

**Conclusion:** These preliminary results support a correlation between the occurrence of stage 3 PGD and fluid balance in the first 24 h after lung transplantation. They suggest that optimized management of fluid balance in the first hours after transplantation could improve patient prognosis.

**Reference 1**

Snell, G. I. et al. Report of the ISHLT Working Group on Primary Lung Graft Dysfunction, part I: Definition and grading—A 2016 Consensus Group statement of the International Society for Heart and Lung Transplantation. J. Heart Lung Transplant. 36, 1097–11.

**Compliance with ethics regulations:** Yes in clinical research.

**Figure 1 (abstract FC-008):**
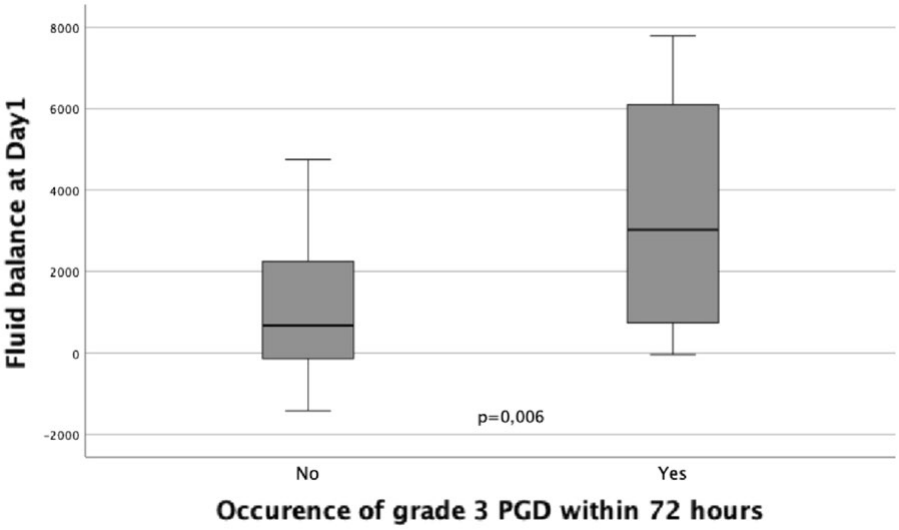
Differences in Median Fluid balance at Day 1 between patients presenting or not PGD stage 3 during the first 72 h

## FC-009 Acute phenobarbital poisoning in a Tunisian intensive care unit: a retrospective study

### Iyed Maatouk^1^, Amira Ben Jaziy^1^, Hassen Ben Ghzela^1^, Soumaya Saad^1^, Salma Sghaier^1^, Nozha Brahmi^1^

#### ^1^Mahmoud Yaacoub Emergency Medical Center of Tunis, Tunis, Tunisie

*Annals of Intensive Care *2013, **13(Suppl 1)**:FC-009

##### **Correspondence:** Iyed Matouk (maatouk.yed@gmail.com)

**Rationale:** Phenobarbital (PB) is one of the oldest anti-epileptic drug. It is effective in all types of epilepsy except typical absences. However, its pharmacokinetic profile and its numerous adverse effects limit its use as a first-line drug in the long-term treatment of epilepsy. Barbiturates, particularly phenobarbital, the main anti-seizure drug, are an important cause of intoxication and mortality. The objective of this study was to describe the clinical, para-clinical, therapeutic and outcome characteristics of phenobarbital intoxications.

**Patients and methods/materials and methods:** We conducted a retrospective study including all cases of phenobarbital poisoning confirmed by serum assay and admitted in our ICU from 2004 to 2021.

**Results:** One hundred thirty-seven patients were included for voluntary poisoning with suicidal attempts. They were female in 66.4%. The mean age was 29.02 ± 12.36 years. A history of psychiatric disorders were found in 21.9%, and 27.6% had previous suicide attempts. The mean estimated ingested dose was 1250 mg (Interquartile range: 912.5–2000). The mean Apache II score was 4 (Interquartile range: 6–19). The mean SAPSII was 11 (Interquartile range 2–7). Neurological, cardiovascular and respiratory distress were present respectively in 59.3%, 12%, 11% of the patients respectively. The most frequent neurological signs were drowsiness (76.3%) followed by coma (12%), agitation (9%) and confusion (3%). Hypotension was observed in 7% and acute respiratory failure due to aspiration pneumonia in 11%. The serum level (phenobarbitolaemia) on admission was 56.95 ± 19.43 mg. Mechanical ventilation was required in (37.2%), fluid expansion (12%), activated charcoal (17.5%), alkaline diuresis (8%), gastric lavage (7.3%). All patients recovered without any sequelae.

**Conclusion:** Phenobarbital poisoning can lead to serious neurological, cardiovascular and respiratory distress. Coma can last for some days. Physicians should be aware of the severity of this drug poisoning which has a good prognostic if symptomatic treatment is started in time.

**Compliance with ethics regulations:** Yes in clinical research.

## FC-010 Epidemiological, clinical and evolutionary profile of patients with acute amitriptyline intoxication

### Soumaya Saad^1^, Amira Ben Jazia^1^, Hassen Ben Ghezala^1^, Iyed Maatouk^1^, Mariem Cheikhrouhou^1^, Salma Essghaier^1^, Salma Ghalloussi^1^, Nozha Brahmi^1^

#### ^1^Centre d'Assistance Médicale Urgente de Tunis (CAMU), Tunis, Tunisie

##### **Correspondence:** Soumaya Saad (saadsoumaya1994@gmail.com)

*Annals of Intensive Care *2013, **13(Suppl 1)**:FC-010

**Rationale:** Amitriptyline is one of the major tricyclic antidepressants that is used widely and is still one of the common causes of fatal drug poisoning. It manifests iespecially with electrocardiographic (ECG) abnormalities, arrhythmias, and hypotension. Predictive factors for the development of life-threatening complications are limited and not well known. The objective of our study is to describe clinical and prognosis findings in patients presenting with acute amitriptyline intoxication.

**Patients and methods/materials and methods:** This is an observational, retrospective single-center study, over a period of 6 years (2017 to 2022). Inclusion of patients over the age of 14 years admitted to ICU for acute amitriptyline intoxication. Demographics, clinical, laboratory, electrocardiographic (ECG) findings and patient’s progress were evaluated retrospectively. Predictive parameters for the development of serious complications were studied.

**Results:** 91 patients were included. Mean age was 32 ± 13 years. Sixty-eight patients were female. Ingested amitriptyline median dose was 400 [240, 500] mg. 56 patients were followed for psychiatric disorders (psychosis (50%), depression (28.5%), bipolar disorder (14.3%), and personality disorder (7.1%). The main clinical neurological symptoms on admission were drowsiness in 84.6% of cases followed by coma in 56 patients (61.5%). Acute respiratory failure was reported in 3 patients. Hemodynamically, tachycardia was reported in 54(59.3%) patients, hypotension in 9(9.9%) patients and shock in 3 patients. Electrocardiographic abnormalities reported in our study series were: widening of the QT space in 6 patients, widening of the QRS complex in 4 cases, ventricular tachycardia in 4 patients and asystole in 2 patients. Therapeutic management was characterized by oxygen in 57(62.6%), volume expansion in 7 patients, catecholamines in 5 patients, bowel accelerator in 21 patients, anticonvulsant therapy in 8 patients. Invasive mechanical ventilation was used in 61.5% of patients. The median hospital stay at ICU was 2 days and only one patient died.

**Conclusion:** Amitriptyline intoxication in our series was associated with a good prognosis but still remains serious due to its cardiac toxicity. Early and rapid management is necessary to anticipate and control potential complications.

**Compliance with ethics regulations:** Yes in clinical research.

## FC-011 Acute Olanzapine intoxication in a Toxicology Intensive Care Unit: a retrospective study

### Hassen Ben Ghezala^1^, Iyed Maatouk^1^, Amira Ben Jazia^1^, Nozha Brahmi^1^

#### ^1^Centre Mahmoud Yaacoub d'assistance médicale urgente de Tunis, Tunis, Tunisie

##### **Correspondence:** Hassen Ben Ghezala (hassen.ghezala@gmail.com)

*Annals of Intensive Care *2013, **13(Suppl 1)**:FC-011

**Rationale:** An overdose of Olanzapine, an atypical antipsychotic, is increasingly used in intentional drug poisoning. It can lead to serious or fatal poisoning. Objective: The objective of this study was to describe the clinical, para-clinical, therapeutic and outcome characteristics of Olanzapine intoxications observed at the Reference Tunisian poison center.

**Patients and methods/materials and methods:** We conducted a retrospective study including all patients admitted for Olanzapine poisoning in our Intensive Care Unit of Urgent Medical Assistance Center Mahmoud Yaacoub (Tunisia) from January 2011 to June 2022.

**Results:** In total, 80 patients were included. In our study, 63,8% patients were female and 36.3% patients were male. The mean age was 34.1 ± 13.06 years. Mental health problems were found in 87.5% of patients. The most frequent was schizophrenia (51.9%). All cases of Olanzapine intoxication were related to suicide attempts. The mean estimated ingested dose of Olanzapine was 166.47 mg ± 112.9. Poison Severity Score was minor in one patient, moderate in 40 patients, and severe in 39 patients. In our series, no death was reported. The intoxication was concomitant to benzodiazepine in 50% of cases. In our study, neurological symptoms were the most common adverse effect. Coma was found in 49% with Glasgow Coma Score (GCS) less than 8 in 33% and drowsiness in 45%. Cardiovascular symptoms were tachycardia (25%), hypotension (3.8%) and shock (1.3%). The membrane stabilizing effect was not noted in any of the patients. Respiratory symptoms were ARF (1.3%) whose etiology was aspiration pneumonia. The most frequent biological abnormality was hypokalemia (35.4%). At the electrocardiogram, the rhythm was regular and sinus (67.5%) except a tachycardia in 24.7% and bradycardia in 3.8%. The screening of olanzapine was performed in the urine (26.3%), the gastric fluid (17.5%) and in blood (1.3%). The treatment was mainly symptomatic: mechanical ventilation (41.3%), gastric lavage (1.3%), vascular expansion (2.5%) and Correction of hypokalemia (35.4%). The different complications noted were: decreased level of consciousness (76.8%), agitation (20.3%), aspiration pneumonia (11,4%), hypotension (2.5%) and bradycardia (1.3%). In our study, no death was reported and all cases recovered without any consequences.

**Conclusion:** In our study, Olanzapine Poisoning was associated with considerable toxicity and a significant number of complications. Therefore, physicians should be aware of the necessity of a careful clinical monitoring in severe cases.

**Compliance with ethics regulations:** Yes in clinical research.

## FC-012 Acute Quetiapine poisoning in the intensive care unit: a retrospective study

### Hassen Ben Ghezala^1^, Iyed Maatouk^1^, Amira Ben Jazia^1^, Nozha Brahmi^1^

#### ^1^Centre Mahmoud Yaacoub d'assistance médicale urgente de Tunis, Tunis, Tunisie

##### **Correspondence:** Hassen Ben Ghezala (hassen.ghezala@gmail.com)

*Annals of Intensive Care *2013, **13(Suppl 1)**:FC-012

**Rationale:** An overdose of Quetiapine can lead to serious or fatal complications. There are few data on quetiapine poisoning in the literature. Objective: The objective of this study was to describe the clinical, para-clinical, therapeutic and outcome characteristics of quetiapine intoxications observed at the Tunisian reference poison center.

**Patients and methods/materials and methods:** We conducted a retrospective case series monocentric study including all quetiapine poisoning admitted the Toxicology and medical Intensive Care Unit from March 2018 to July 2022.

**Results:** Fifteen patients were enrolled. Thirteen (13) patients were female and 2 patients were male. The mean age was 38 ± 14 years. Twelve patients were known to have bipolar disorder. All cases of quetiapine intoxication were related to suicide attempts. The mean estimated ingested dose of Quetiapine was 883 mg ± 665.2. Poison Severity Score was minor in 4 patients, moderate in 5 patients and severe in 6 patients. In our series, no death was reported. The intoxication was concomitant to benzodiazepine consumption in 8 cases. The most frequent neurological signs were coma (6/15) with Glasgow Coma Score (GCS) less than 8 (6/15) and drowsiness (4/15). Cardiovascular symptoms observed were hypotension (4/15), shock (1/15), tachycardia (1/15) and a long QT (2/15). We did not observe any membrane stabilizing effect in our patients. Respiratory symptoms observed were acute respiratory failure (7/15) with mainly aspiration pneumonia in 5 cases. The most frequent laboratory disorder was hypokalemia (8/15). At the electrocardiogram, we noticed regular sinus rhythm (12/15) except one case with tachycardia (1/15) and two others with a long QT (2/15). The qualitative serum screening of Quetiapine was performed only in one patient. The treatment was mainly symptomatic: mechanical ventilation (5/15) gastric lavage (2/15), fluid expansion (2/15), transit accelerator (2/15) and Correction of hypokalemia (8/15). The different complications seen were: hypotension in 4 cases with one case of shock, aspiration pneumonia in 5 cases, hypokalemia in 8 cases, decreased level of consciousness in 6 cases, delirium, agitation and irritability in one case. In our study, no death was reported and all cases recovered without any sequelae.

**Conclusion:** In our study, quetiapine overdoses were associated with considerable neurological and cardiovascular toxicity. Emergency department physicians should be aware of the increasing number and the severity of this emerging drug poisoning by quetiapine.

**Compliance with ethics regulations:** Yes in clinical research.

## FC-013 Acute Risperidone Intoxication: Retrospective Study 2012–2022

### Hassen Ben Ghezala^1^, Boudour Ben Dhia^1^, Amira Ben Jazia^1^, Nozha Brahmi^1^

#### ^1^Centre Mahmoud Yaacoub d'assistance médicale urgente de Tunis., Tunis, Tunisie

##### **Correspondence:** Hassen Ben Ghezala (hassen.ghezala@gmail.com)

*Annals of Intensive Care *2013, **13(Suppl 1)**:FC-013

**Rationale:** Risperidone intoxication is uncommon and known to have a good prognosis with a low mortality rate. Very few reports on this intoxication exist.

**Patients and methods/materials and methods:** Retrospective observational study that included all patients admitted to the medical and toxicology intensive care unit of a reference poison center for management of Risperidone intoxication. The study period was 10 years (from 2012 to 2022).

**Results:** Forty-four patients were included during the study period, a female predominance was noted with a gender ratio (1.93), the mean age was 33 years ± 14. Psychiatric history was reported in 86% of the patients (22.7% depressed; 18.2% bipolar disorder; 18.2% schizophrenic and 40.9% had unspecified psychiatric history). In 9% of the cases the intoxication was related to a psychiatric decompensation and a therapeutic overdose and in 91% of cases was for suicidal purposes. Multi-drug intoxication was reported in the majority of cases: 62% associated with benzodiazepines, 20% associated with another neuroleptic and in 10% antidepressant. The supposed dose ingested was 40 mg [8,120] with an average consultation time of 3 h [1,6]. The neurological symptoms reported were: 50% somnolence, 25% extrapyramidal syndrome, 15.9% agitation, 4.1% seizures. A decreased level of consciousness (average Glasgow score 11 ± 4) was reported with a delay after intoxication of 5 ± 2 h and required mechanical ventilation in 36%. Pupils were reflexive in 45.5%, mydriatic in 11.4% and miosis in 43.2%. No hyperthermia was reported. Cardiovascular manifestations reported: tachycardia 75%, collapsus 16% (43% requiring catecholamines and 47% responding to fluid expansion), rarely an ECG change was reported: long QT 6.8%, right bundle branch block 4.5%. The most frequent metabolic disorders were metabolic acidosis (29.5%), rhabdomyolysis (9.1%) and acute renal failure (4.5%), of which only one patient required hemodialysis. Activated charcoal was used in two patients. 42% of the patients presented pneumonia: 18.8% ventilator-associated pneumonia; 22.7% aspiration pneumonia. The median duration of mechanical ventilation was 2 days [1; 24]. Among intubated patients, 32% presented an agitated awakening and 93.8% were subsequently extubated. The median length of stay in intensive care was 2 days [2,32]. The reported death rate was 2.3%.

**Conclusion:** This study shows that acute risperidone intoxication can cause severe neurological and cardiovascular manifestations. Treatment is mainly symptomatic with no specific antidote.

**Compliance with ethics regulations:** Yes in clinical research.

## FC-014 Minoxidil poisoning in critical care

### Maroua Jemii^1^, Hassen Ben Ghezala^1^, Amira Ben Jazia^1^, Nozha Brahmi^1^

#### ^1^Centre Mahmoud Yaacoub d'assistance médicale urgente de Tunis, Tunis, Tunisie

##### **Correspondence:** Hassen Ben Ghezala (hassen.ghezala@gmail.com)

*Annals of Intensive Care *2013, **13(Suppl 1)**:FC-014

**Rationale:** Minoxidil was initially an antihypertensive drug, which had restricted therapeutic indications. It is currently used in topical form for the treatment of hair loss.

**Patients and methods/materials and methods:** Monocentric retrospective observational study which included all patients admitted for minoxidil intoxication at the medical and toxicological intensive care unit of the Tunis reference poison center over a period of 13 years (2009–2022). Inclusion criteria were mainly: ingestion of minoxidil associated with clinical and biological signs of intoxication.

**Results:** During the study period, 16 patients were admitted for the management of minoxidil intoxication and included in the study. The mean age was 43 ± 21 years with a female predominance of 81.3%. Three patients (18.8%) had a history of hypertension and one (6.3%) patient had a psychiatric history. Thirteen patients (81.3%) mistook the treatment for a cough syrup and ingested it accidentally, while 3 patients (18.8%) took the treatment voluntarily for suicide attempt. The average quantity ingested was 16 ± 14 cc with an average time to the emergency room of 10 ± 9 h. At presentation, all patients were conscious, among which 6 (37.5%) presented with hypotension, 1 of which had peripheral signs of shock. Other signs of intoxication were dizziness with nausea in 7 patients (43.8%), abdominal pain and vomiting. Blurred vision was noted in only one patient and palpitations in half of the patients. During hospitalization, 10 patients (62.5%) presented shock that lasted on average 2.5 ± 1.4 with a maximum vasoactive drug dose of 5 mg/h. On ECG, 10 patients (62.5%) had repolarization disorders, and troponins were elevated in 4 patients (25%). The major complications in ICU were mainly cardiogenic pulmonary edema in 3 patients (18.75%) and infectious pneumonia in 1 patient. The average length of stay was 3 ± 1 days.

**Conclusion:** Although minoxidil intoxication is rare, it can be serious and life-threatening. In fact and as demonstrated by this work, several hemodynamic and cardiovascular complications can occur, Treatment is mainly symptomatic.

**Compliance with ethics regulations:** Yes in clinical research.

## FC-015 Acute Colchicine intoxication: A 16-year retrospective study

### Amira Ben Jazia^1^, Salma Ghalloussi^1^, Hassen Ben Ghezala^1^, Boudour Ben Dhia^1^, Mariem Cheikhrouhou^1^, Ons Ellouze^1^, Nozha Brahmi^1^

#### ^1^CAMU, Tunis, Tunisie

##### **Correspondence:** Salma Ghalloussi (salmaghalloussi93@gmail.com)

*Annals of Intensive Care *2013, **13(Suppl 1)**:FC-015

**Rationale:** Colchicine is a drug with a narrow therapeutic margin. The intoxication is rare but can be serious, even fatal, due to multivisceral failure.

**Patients and methods/materials and methods:** Retrospective observational study from 2007 to 2022 including all patients admitted for colchicine intoxication in the department of intensive care and toxicology of CAMU. Were included poisoned patients with supposedly toxic ingested dose ≥ 0.15 mg/kg. We collected demographic, clinical, biological and prognostic data.

**Results:** During the study period, 25 patients were admitted for acute colchicine voluntary intoxication with a female predominance (88%) and an average age of 27 ± 11 years. Co-morbidities were Gout disease (8%), Behcet's disease (8%), pericarditis (4%), lupus in (4%), rheumatic disease (4%), hypertension and dyslipidemia (4%). No psychiatric history was found. The intoxication was poly-drug in 28% of cases. The digestive signs were predominant with vomiting (84%), nausea (82%), abdominal pain (72%), profuse diarrhoea (64%) and digestive haemorrhage (8%). Cardiovascular signs were also frequent including tachycardia (52%), hypotension (12%), QT and ST segment prolongation (8%) with repolarization disorders (8%). Fever was present in 16% of cases. Neurological signs were rare as confusion (8%) or delirium (4%). Hydroelectrolytic disorders were frequent (48%) dominated by hypokalemia (44%), thrombocytopenia (16%), cytolysis (8%), cholestasis (4%) and lactic acidosis (24%). The poisoning was complicated by hepatocellular insufficiency (12%), rhabdomyolysis (12%), acute renal failure (8%) and pancytopenia in two patients. Excessive alopecia occurred in one patient. Mechanical ventilation was necessary in three patients (12%) with fatal outcome for the three intubated patients. Prognostic factors were an assumed ingested dose of 0.3 mg/kg [0.1, 1.5], a mean delay to initial consultation (7 h [1,47]), the decrease in prothrombin level (12%), the occurrence of cardiogenic shock (20%), an acute respiratory distress syndrome (8%), a sepsis (8%) or a multivisceral failure (16%).

**Conclusion:** Colchicine intoxication is potentially lethal and there is no specific antidote to date. It is recommended to observe the patient for at least 24 h. Close monitoring should be performed and supportive treatment should be initiated early.

**Compliance with ethics regulations:** Yes in clinical research.

## FC-016 Theophylline intoxications: clinical presentation management and prognosis

### Amira Benjazia^1^, Boudour Ben Dhia^1^, Salma Ghalloussi^1^, Hassen Ben Ghezala^1^, Mariem Cheikhrouho ^1^, Salma Esseghaeir^1^, Ons Ellouze^1^, Ikram Ben Braeik^1^, Nozha Brahmi^1^

#### ^1^CAMU, Tunis, Tunisie

*Annals of Intensive Care *2013, **13(Suppl 1)**:FC-016

##### **Correspondence:** Boudour Ben Dhia (bidourabdbg2014@gmail.com)

**Rationale:** Theophylline belongs to the xanthine family and is used for its bronchodilator effect. However, because of its very narrow therapeutic margin, it is less and less prescribed. Theophylline intoxications, whether accidental or voluntary, are rare but can be serious in some cases due to life-threatening cardiac and neurological complications.

**Patients and methods/materials and methods:** It was a retrospective study between 2017 and 31st January 2023. We followed all patients who had been referred to our ICU for theophylline intoxication. Information collected included age, sex, amount of drug supposedly ingested and any initial clinical manifestations of toxicity. A blood sample was also collected for determination of plasma theophylline concentration. A plasma theophylline concentration ≥ 15 mg/L was considered a reference for the diagnosis of poisoning.

**Results:** Forty-five patients were included. Their median age was 20 [14;84] years; they were predominantly female 35 (79,5%). The co-morbidities were in 27% chronic obstructive pulmonary disease (asthmatic n = 7; 58% and chronic obstructive pulmonary disease n = 5; 42%). A history of psychiatry depressive disorder was noted in 12%. The mean delay in presenting to the emergency department was of 6 h [1;25]. The median theophylline supposed ingested dose was 3000 mg [300; 12000]. It was associated with other substances in 23 cases. Intoxication was voluntary in 91% and the overdose was reported in 9%. Clinical features were digestive disorders in 36 cases (80%), tachycardia in 28 patients (62.2%), tremors in 12 patients (27%), agitation for two patients (4%), seizures in one case and mean of CGS was 15 [14–15]. Six patients had a hemodynamic distress requiring the use of cathecolamines. Metabolic acidosis was present in 36 patients (80%) with a mean bicarbonate level of 18.8 ± 3.66 mmol/L. Lactate measurement was performed in 16 patients with a mean of 4.69 ± 2.02 mmol/L. Hypokalemia was found on admission in 27 patients (60%), with a medium level of 3.18 ± 0.53 mmol/l. Theophylline measurement was performed at admission with a median of 40[20;86] mg/l. The use of beta blockers as antidote was required in 15 cases (33%). The average length of stay in hospital was 2 days [1,6]. The evolution was favorable in all cases.

**Conclusion:** Doctors should be aware of theophylline toxicity. Theophylline poisoning can be fatal for serum level above 100 mg/l. Recognition of clinical signs of poisoning and rapid management are the only guarantees of the prognosis.

**Compliance with ethics regulations:** Yes in clinical research.

## FC-017 Relevance of the REM Score in predicting mortality in patients admitted to the life-saving emergency room

### Manel Kallel^1^, Houyem Zouari^1^, Salma Kammoun^1^, Khedija Zaouche^1^

#### ^1^Emergency departement of Mahmoud ElMatri hospital, Ariana, Tunisie

##### **Correspondence:** Manel Kallel (manel.kallel@fmt.utm.tn)

*Annals of Intensive Care *2013, **13(Suppl 1)**:FC-017

**Rationale:** Early assessment of the prognosis of patients admitted to emergency departments (EDs) is a major objective of the emergency physician. Management and referral depend on this assessment. Numerous severity scores based on the patient's clinical and biological parameters have been developed and validated. The Rapid Emergency Medicine Score (REMS) is one of these scores and has been studied in trauma emergencies and in septic patients. The main objective of this study is to evaluate the prognostic value of REMS in the prediction of intra-hospital mortality at 24 h in patients admitted to the emergency room.

**Patients and methods/materials and methods:** This is a prospective, observational, single-centre study conducted in an emergency department of a regional hospital over a six-month period. We included all patients managed in the life-saving emergency room and calculated their REMS.

**Results:** During the study period, we included 367 patients. The mean age of the patients was 55 with a sex ratio of 1.75. The in-hospital mortality rate was estimated to be 5.7%. The REMS score was statistically higher in the group of deceased patients (p = 0.02). The mean REMS in survivors was 4.75versus 9.43 (p = 0.02); patients deceased with a REMS inferior to 6 were six and those with a REMS superior to 6 were fifteen (p < 0.001). A REMS superior to 6 was also significantly associated with admission to intensive care (p = 0.031). In univariate study, it was predictive of mortality (OR = 5.98, 95% CI [2.25—15.84], p = 0.001). The characteristics of the ROC curve of the REMS score for predicting mortality were therefore: AUC 0.79, Sensitivity 71.43%, Specificity 70%, positive predictive value 12.82%, negative predictive value 97.6%.

**Conclusion:** The REMS is a good predictive tool for in-hospital mortality at the 24th hour of admission of patients in the emergency department. It is simple and easy to calculate.

**Compliance with ethics regulations:** Yes in clinical research.

## FC-018 Profile of patients admitted to the vital reception emergency room for a cardiovascular pathology

### Manel Kallel^1^, Sana Sallemi^1^, Salma Kammoun^1^, Amal Oussaifi^1^, Khedija Zaouche^1^

#### ^1^Emergency departement Hospital of Mahmoud El Matri, Ariana, Tunisie

##### **Correspondence:** Manel Kallel (manel.kallel@fmt.utm.tn)

*Annals of Intensive Care *2013, **13(Suppl 1)**:FC-018

**Rationale:** Cardiovascular pathology is one of the most frequent causes of admission of patients to the emergency room. Classically associated with the male gender and advanced age, its prevalence in women and young adults is nevertheless clearly increasing. The aim of our work was to study the epidemiological, clinical and evolutionary characteristics of cardiovascular pathologies requiring admission to life-saving emergency room.

**Patients and methods/materials and methods:** Retrospective, descriptive, comparative and monocentric study conducted over a period of four months in a multipurpose emergency unit of a regional hospital. We included patients aged 18 years and older with a cardiovascular pathology requiring a temporary hospitalization in the emergency room. We excluded pregnant women and any post-traumatic context.

**Results:** During the study period, 397 patients required admission to the life-saving emergency room, and cardiovascular pathology was identified in 93 patients (23.4%). Among them, there were 63 men (67.7%) and 30 women (32.3%). The average age of the women was 68 ± 15 years, while that of the men was 59 ± 16 years (p = 0.03). The diagnosis was acute coronary syndrome (ACS) in 43% of the cases (of which 20% were STEMI), acute heart failure (AHF) in 24.7%, a rhythm disorder in 17.2%, aorto-vascular pathology in 8.7%, pericarditis and cardiorespiratory arrest in 3.2%. In the ACS subgroup, the average age of onset was 57 ± 15 years (p = 0.03). Pain was particularly severe (p = 0.046). Blood glucose was initially higher at 2 ± 1.4 g/l (p = 0.048). However, the heart rate was significantly lower at 89 ± 17batt/mn (p = 0.05). The platelet count was significantly higher at 267,840 ± 67,000 el/mm3. In the CIA subgroup, the mean age was 74 ± 9 years (10–3), the MEWS score was significantly elevated (10–3) as was the SAP (10–3). In the rhythm disorder subgroup, the mean age was 60 ± 15 years (p = 0.7). There was no significant association with NEWS score, MEWS or pain intensity. Systolic blood pressure was significantly lower at 100 mmHg (10–3) with no particular elevation in lactate levels (p = 0.8). Five patients died, including two in the ACS subgroup.

**Discussion:** Cardiovascular pathologies require scores to assess the severity and choose the orientation of patients admitted to the emergency room.

**Conclusion:** Cardiovascular pathology concerned elderly men and young women. The ACS subgroup was distinguished by high adrenergic stimulation, which was higher than in the rhythm disorder subgroup.

**Compliance with ethics regulations:** Yes in clinical research.

## FC-019 Acute heart failure in the emergency departement: prognosis value of D-dimeres

### Badra Bahri^1^, Hanene Sakhri^1^, Ines Sedghiani^1^, Hamdi Doghri^1^, Borsali Falfoul^1^

#### ^1^Hôpital Habib Thameur, Tunis, Tunisie

##### **Correspondence:** Badra Bahri (bahribadra@gmail.com)

*Annals of Intensive Care *2013, **13(Suppl 1)**:FC-019

**Rationale:** Acute heart failure (AHF) is a frequent cause of admission in the emergency departement (ED). The prognosis evaluation is an essential step for the emergency physician. D- dimers are frequently collected in the ED given the importance of venous thromboembolic disease among the causes of decompensation of AHF. The purpose of our study was to identify the prognostic value of D-dimers in patients admitted to the ED for AHF.

**Patients and methods/materials and methods:** Monocentric observational study. Prospective collection of clinical, biological and evolution demographic data from computerized medical records. The diagnosis of AHF was retained on clinical, electrocardiographic, radiological, biological and gazometric data. The dosage of D-dimers was performed on admission to the ED. The primary endpoint was intra-hospital mortality.

**Results:** One hundred and twenty patients were included. The mean age was 72 ± 11.4 years, the sex ratio was 0.96. The comorbidities were high blood pressure (78.3%), diabetes (49.2%), coronary artery disease (45.8%), heart failure (53.3%). 70% of patients consulted for orthopnea. The average length of stay was 6.36 ± 5.03 days. The use of continuous positive airway pressure (CPAP) support was indicated in 36% of patients. The use of ventilatory support with inspiratory support was necessary in 37% of patients. The rate of admission to intensive care was 11.8%. The average D-dimer value was 1966 ± 2627ug/L. Intra-hospital mortality was 14.2%. Comparing the two groups of patients, the deceased (G1) versus the survivors (G2), the mean value of the D-dimers was (ug/L): G1: 4262 ± 5370 vs G2: 1550 ± 1390; p = 0.000. In multivariate analysis, a D-dimer value greater than 913 ug/L was an independent factor of intra-hospital mortality (AUC = 0.810; Se = 84%; Sp 60%; OR 3; 95% CI: 0.64–14.6; p = 0.017).

**Conclusion:** A Ddimer value greater than 913ug/L is an independent factor associated with in-hospital mortality in patients admitted to the emergency department for acute heart failure.

**Compliance with ethics regulations:** N/A.

## FC-020 Preventable and Potentially Preventable Mortality in Victims of Terrorism

### Chihebeddine Romdhani^1^, Aïcha Rebai^1^, Imene Chaieb^2^, Maha Shimi^1^, Walid Samoud^1^, Hazem Fourati^1^, Mustapha Ferjani^1^

#### ^1^Hôpital Militaire Principal d'Instruction de Tunis, Tunis, Tunisie; ^2^Hôpital Militaire, Gabès, Tunisie

##### **Correspondence:** Aïcha Rebai (aicha.rebai@fmt.utm.tn)

*Annals of Intensive Care *2013, **13(Suppl 1)**:FC-020

**Rationale:** Terrorism is an international scourge that is the source of non-negligible mortality among victims. However, some deaths can be avoided through pre-hospital care. This study aimed to classify the deaths among victims of terrorism.

**Patients and methods/materials and methods:** We conducted a retrospective study from 2012 to 2017. We included victims of terrorist acts in our country who underwent autopsies at our Military Institution. A group of experts separately classified the deceased patients based on autopsy reports and images of injuries using the following classification: A preventable death, when a lifesaving act could be performed at the scene of the event by the victim, a person present next to the victim, or a first-line medical or paramedical professional. A potentially preventable death, when a potential lifesaving act is possible but unavailable due to lack of means or skills and can only be performed by a highly qualified person in a well-developed hospital setting. An unavoidable death: when the injury is beyond all therapeutic resources and cannot be treated no matter the means and skills available. Death was defined as preventable, potentially preventable, or unavoidable if at least two experts established this consideration.

**Results:** Our study included forty-six victims who died during a terrorist event, of which 93.4% died at the scene of the event and 6.6% in-hospital. A group of experts classified the patients using the following classification: preventable death, potentially preventable death, and unavoidable death. The three experts agreed on 82.6% of the deceased victims. The experts considered ten patients as having had preventable and potentially preventable death. The others were considered to have had an unavoidable death. We analyzed the degree of agreement among the experts' opinions. The intraclass coefficient was 0.9, indicating excellent agreement. The Kendal and Fleiss Kappa coefficients favored a strong agreement between the experts. The Kendal coefficients between the experts were all greater than 0.7, in favor of a strong agreement among the experts. The Kappa coefficient was greater than or equal to 0.66, indicating a strong to excellent agreement between the experts. All victims whose death was preventable and potentially preventable died from shock and hemorrhage.

**Conclusion:** All preventable deaths were secondary to a state of shock and hemorrhage. Classifying the deaths of victims of terrorism allows for future strategies to be put in place for both in-hospital and pre-hospital care to reduce preventable and potentially preventable deaths.

**Compliance with ethics regulations:** Yes in clinical research.

**Table 1 (abstract FC-020):**
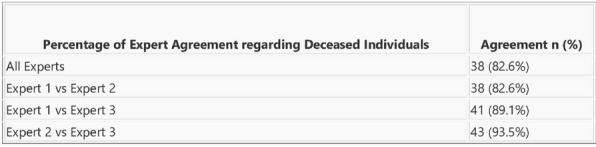
Expert Agreement on Preventable and Potentially Preventable or Non-Preventable Mortality

## FC-021 Thoracic polytrauma

### Rim Karray ^1^, Alaeddine Zouari^1^, Rezk Ghorbel ^1^, Hela Fourati^1^, Noureddine Rekik^1^

#### ^1^Hôpital Habib Bourguiba Sfax, Sfax, Tunisie

*Annals of Intensive Care *2013, **13(Suppl 1)**:FC-021

##### **Correspondence:** Alaeddine Zouari (zouari.aladin@gmail.com)

**Rationale:** Chest trauma is the third leading cause of trauma mortality. They are rarely isolated and are most often associated with other life-threatening lesions. The aim of our study is to report the experience of our department concerning chest trauma.

**Patients and methods/materials and methods:** This is a prospective study including patients who are admitted for chest trauma, over a period of one year, from 1^st^ August, 2020 to 31^st^ July, 2021 at the emergency department in the CHU Habib Bourguiba in Sfax.

**Results:** The total number of polytrauma patients admitted to our department during this period was 470 patients. Patients with chest trauma counted 203 patients. The average age was 37.6 years with extremes ranging from 1 to 80 years. They were 172 men and 31 women with a sex ratio of 5/1. It was a blunt trauma in 199 cases. On clinical examination, the respiratory rate was 18.4 bpm with extremes ranging from 12 to 33 bpm. Pulmonary auscultation was abnormal in 80 cases. The average oxygen saturation was 96.16% with extremes ranging from 66 to 99%. The average shock index was 0.85. The average Glasgow score was 13.2. On clinical examination of the chest, forty-nine patients presented pain and forty-seven patients presented chest contusion and bruises. Sixteen patients had rib fractures. Subcutaneous emphysema was identified in twenty cases. A chest X-ray was performed in one hundred and seven patients and was abnormal in ninety-five cases. An E-FAST ultrasound was performed in sixty-nine cases and showed the presence of pneumothorax in seventeen cases and hemothorax in fifteen cases. A Bodyscan was performed. It showed that the most frequent lesions in decreasing order were a rib fracture in 129 cases, a pulmonary contusion in 126 cases, a pneumothorax in 126 cases, an hemothorax in fifty-six cases. A flail chest was noted in eleven patients. Thirteen patients had a sternum fracture. A scapula fracture was noted in thirty-eight patients. Twenty-five patients had a clavicle fracture. Dorsal spinal trauma was associated in twenty-nine cases. Fifty drains were inserted. Fifty-three patients were intubated and ventilated. The evolution was marked by the death of nine patients, the transfer to another department of 127 patients and sixty-seven patients were discharged.

**Conclusion:** High-energy accident should always lead to the examination of the chest, even if the initial presentation is reassuring. Chest trauma should be considered as an evolving lesion and should therefore benefit from a reevaluation.

**Compliance with ethics regulations:** Yes in clinical research.

## FC-022 Sedation in the emergency surgical setting

### Karima Naanani^1^, Loubna Benaadi^1^, Ayman Eloudghiri ^1^, Soufiane Saadaoui^1^, Rachid Alharrar^1^

#### ^1^Réanimation P33, CHU Ibnrochd, Casablanca, MAROC

##### **Correspondence:** Karima Naanani (naananik@gmail.com)

*Annals of Intensive Care *2013, **13(Suppl 1)**:FC-022

**Rationale: **The organization of the practice of sedation analgesia imposes a strict rigor, to reach an optimal and rationalized level of sedation. This study allowed us to review the practice of sedation analgesia in the emergency surgical unit, to evaluate the complications of prolonged sedation and in particular its impact on the duration of mechanical ventilation (MV) and on the length of stay.

**Patients and methods/materials and methods: **Our prospective observational study lasted 17 months and included 230 patients sedated in the emergency surgical unit of the CHU Ibn Rochd.

**Results: **Our study included 230 sedated patients, 79% were male, the mean age was 46.2 ‡ 19.3 years. Post-traumatic consciousness disorders represented the first indication for admission to the intensive care unit (43.5%). The mean duration of sedation was 3.6 days [1–36 days], the median duration of VM and the median length of stay were 5 and 6.5 days, respectively. Mortality was more common in the sedated group superior to 48H. The introduction of sedation analgesia protocol and a better understanding of the pharmacology of sedation means, as well as the application of optimal and rationalized sedation significantly reduced the duration of MV, the length of stay, the total duration of sedation, the duration of intubation, the risks of tracheotomy and ventilator-associated pneumonia (all P value less than 0.001). This also led to better management of the weaning syndrome, delayed recovery, and the occurrence of critical illness neuromyopathy.

**Conclusion: **Sedation-analgesia, although frequently used, involves certain risks. Its practice has been evolving for several years towards a rationalization of its administration according to the needs of the patient. Reducing the duration of sedation could considerably reduce the duration of MV, and consequently the length of stay.

**Compliance with ethics regulations:** Yes in clinical research.

## FC-023 Association between antibiotic therapy and prehospital hemodynamic optimization with 30-day mortality in patients with septic shock

### Romain Jouffroy^1^, Florian Negrello^2^, Basile Gilbert^3^, Stéphane Travers^4^, Emmanuel Bloch-Laine^5^, Patrick Ecollan^6^, Vincent Bounes^3^, Benoit Vivien^7^, Papa Gueye^2^

#### ^1^APHP-CHRU Ambroise Paré, Boulogne-Billancourt, France; ^2^SAMU 972 Hôpital Universitaire de Martinique, Fort-de-France, Martinique, France; ^3^CHRU Toulouse SAMU 31, Toulouse, France; ^4^Paris Fire Brigade, Paris, France; ^5^APHP-CHRU Cochin Hôtel Dieu, Paris, France; ^6^APHP-CHRU La Pitié Salpêtrière, Paris, France; ^7^APHP-CHRU Necker enfants malades SAMU 75, Paris, France

##### **Correspondence:** Romain Jouffroy (romain.jouffroy@gmail.com)

*Annals of Intensive Care *2013, **13(Suppl 1)**:FC-023

**Rationale: **International recommendations for the management of sepsis and septic shock recommend early recognition, diagnosis and treatment to reduce associated mortality [1,2]. More than a single treatment, a set of care including antibiotic therapy (ABT) and hemodynamic optimization has demonstrated its effectiveness in a hospital setting. This study aims to study the association between 30-day mortality in patients with septic shock and the administration of ABT combined with hemodynamic optimization defined by a volume expansion of at least 10 ml kg^−1^ h^−1^.

**Patients and methods/materials and methods: **From May 2016 to March 2021, patients with septic shock requiring prehospital intervention by an Out-of-Hospital Mobile Emergency Unit (SMUR) were retrospectively analyzed. To assess the association with 30-day mortality, the Inverse Probability Treatment Weighting (IPTW) propensity method was applied.

**Results: **Among the 529 patients included, 354 (67%) were analyzed. Suspected pulmonary, digestive, and urinary infections were the cause of septic shock in 49%, 25%, and 13% of cases, respectively. The overall 30-day mortality was 32%. Seventy-one patients (20%) received prehospital ABT (C3G or Tazocillin) and volume expansion of at least 10 ml.kg-1.h-1. Binomial log regression weighted by the IPTW found a significant association between 30-day mortality and prehospital administration of antibiotic therapy combined with prehospital hemodynamic optimization by optimal vascular replacement (RR of 0.56 [0.33—0.89], p = 0.02 and adjusted RR 0.52 [0.27–0.93], p = 0.03).

**Conclusion: **The administration of antibiotic therapy combined with prehospital hemodynamic optimization is associated with a reduction in 30-day mortality in patients with septic shock treated by a SMUR team.

**Reference 1**

Singer M. JAMA. 2016.

**Reference 2**

Chen AX. N Engl J Med. 2019.

**Compliance with ethics regulations:** Yes in clinical research.

## FC-024 Prognosis value of procalcitonine in patients admitted to the emergency departement for acute dyspnea

### Badra Bahri^1^, Yosra Jridi^1^, Ines Sedghiani^1^, Hamdi Doghri^1^, Nebiha Falfoul^1^

#### ^1^Hôpital Habib Thameur, Tunis, Tunisie

##### **Correspondence:** Badra Bahri (bahribadra@gmail.com)

*Annals of Intensive Care *2013, **13(Suppl 1)**:FC-024

**Rationale: **Acute dyspnea is a frequent complaint in the emergency depatement (ED). The use of biological markers is a tool that can help the emergency physician for an appropriate orientation of patients according to severity. The prognostic value of procalcitonine (PCT) has been described in many pathologies. The purpose of our study was to assess the prognostic value of PCT in patients admitted to the ED for acute dyspnea.

**Patients and methods/materials and methods: **It was a single-center study between January and March 2022. We collected prospective collection of demographics, clinical and biological data from a computerized medical record. We included all patients over the age of 18 admitted to ED for non-traumatic acute dyspnea. A PCT assay was performed on admission. The primary endpoint was intra-hospital mortality. The secondary endpoint was the use of mechanical ventilation.

**Results: ** 211 patients were included. The average age was 72 ± 13.46 years. The sex ratio 1.04. The comorbidities were (%): high blood pressure (n = 115, 54%), diabetes (n = 83, 39%), coronary artery disease (n = 47, 22.2%), obstructive pulmonary disease (COPD) (n = 48, 22.7%). The main causes of dyspnea were n (%): pneumonia (n = 46, 21.8%), heart failure (n = 49, 23%), pulmonary embolism (n = 11, 5.2%), covid (n = 75, 35.5%), exacerbation of COPD (n = 11, 5,2%). In-hospital mortality was 23%. The mechanical ventilation rate was 8%. The average PCT value was 5.5 ng/L. We compared the PCT value of the group of deceased patients (G1) versus G2 survivors: G1: 12.96 ng/L; G2: 3.35 ng/l; p = 0.001. PCT AUC for predicting mortality was 0.734. In multivariate analysis, a PCT value greater than 0.60 ng/l was an independent factor of mortality (OR 3, 74; 95% CI: 0.96–14.8; p = 0.000); with a specificity of 82%. By comparing the PCT value in patients who required mechanical ventilation (GA) vs those not ventilated (GB): GA 22.4 ng/L; GB 4.11 ng/l; p = 0.000. The AUC for the prediction of mechanical ventilation was 0.600. A PCT greater than 0.8 ng/l was an independent factor for the use of mechanical ventilation (OR 1.12; 95% CI 1.06–1.19, p = 0.004).

**Conclusion :**A PCT greater than 0.6 ng/L is an independent factor of intra-hospital mortality and a value greater than 0.8 ng/L is a factor associated with mechanical ventilation in patients admitted to the ED for acute dyspnea.

**Compliance with ethics regulations:** N/A.

## FC-025 Chronic Critical Illness in burn patients: incidence and risk factors

### Mariem Cheikhrouhou^1^, Hana Fredj^1^, Sarra Ben Zarrouk^1^, Imen Jami^1^, Bahija Gasri^1^, Manel Ben Saad ^1^, Amel Mokline^1^, Amen Allah Messadi^1^

#### ^1^Centre de traumatologie et des grands brûlés, Ariana, Tunisie

##### **Correspondence:** Mariem Cheikhrouhou (cheikhrouhou.mariem@yahoo.com)

*Annals of Intensive Care *2013, **13(Suppl 1)**:FC-025

**Rationale: **Chronic critical illness (CCI) characterizes patients admitted to the intensive care unit who, after an acute illness, remain dependent on care. Its incidence varies from 7 to 33% depending on the studied population. The aim of our study was to determine the incidence of CCI in burn patients and its risk factors.

**Patients and methods/materials and methods**: Retrospective observational case–control study, conducted over a period of 1 year (2021) in a severely burned patients’ intensive care unit of Tunisia. The RTI defined CCI by a length of stay more than eight days and one of the following criteria: sepsis, mechanical ventilation (MV) of more than 96 h, severe injury, head trauma or stroke and tracheostomy (1). All patients aged over 16 years, burned and having been hospitalized for more than eight days were included.

**Results: **Out of 463 hospitalized patients, 103 patients were included. The average age was 43 ± 18 years, the sex ratio was 1.5, the median total burned skin area (TBSA) was 30%. The history was dominated by diabetes (n = 22; 21,4%) and psychiatric disorders (n = 12; 11,7%). The population was subdivided into two groups: 85 patients in the CCI group and eight patients in the non-CCI group. The incidence of CCI was 27%. The eligible criteria for CCI were mainly sepsis (n = 83; 97,6%) and MV (n = 57; 67%). Risk factors for CCI in multivariate analysis were: APACHE II, SOFA, UBS, TBSA, development of acute renal failure and neuropsychiatric disorders, certain hematologic disorders (anemia, thrombocytopenia, lymphopenia) and metabolic disorders (hypernatremia, hypophosphatemia and metabolic acidosis). A TBSA greater than or equal to 21.5% (AUC = 0,759; Se = 68,2% and Sp = 72,2%) and a UBS score greater than or equal to 22 (AUC = 0,768; Se = 76,5% and Sp = 72,2%) predicted CCI. The median of hospital stay was 12 days [8 to 128 days], longer in the CCI group (p = 0.02). The mortality rate of the CCI group was 55.3%.

**Conclusion**: Our study showed that CCI is frequent in burn patients with an incidence of 27% and it is associated with a high mortality rate.

**Reference 1**

1. Kahn JM, Le T, Angus DC, Cox CE, Hough CL, White DB, et al. The Epidemiology of Chronic Critical Illness in the United States. Crit Care Med. févr 2015;43(2):282?7.

**Compliance with ethics regulations:** Yes in clinical research.

**Table 1 (abstract FC-025):**
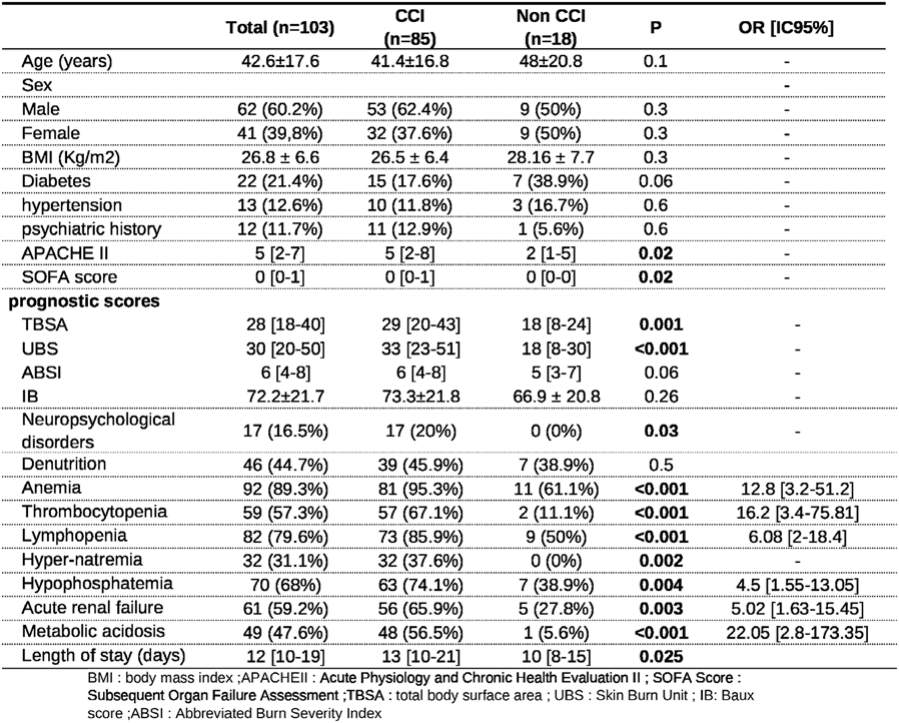
Multivariate analysis: CCI vs Non-CCI group

## FC-026 One-year prognosis of the elderly with at least one organ failure treated in the ICU

### Rémy Marnai^1^, Mickaël Landais^1^, Lev Volkov^1^, Jean-Christophe Callahan^1^, Sébastien Gibot^2^, Christophe Guitton^1^

#### ^1^CH Le Mans, Le Mans, France; ^2^CHRU Nancy-Hôpital Central, Nancy, France

##### **Correspondence:** Rémy Marnai (rmarnai@ch-lemans.fr)

*Annals of Intensive Care *2013, **13(Suppl 1)**:FC-026

**Rationale: **The admission and level of care of the very old (≥ 80 years) in the ICU, require lengthy upstream discussions and should be focused on the expected benefit to the patient. The main objective of this work was to study the one-year prognosis of patients aged 80 years and over, admitted to the ICU with at least one organ failure treated.

**Patients and Methods/Materials and Methods: **From January 2015 to December 2019, we conducted an observational, retrospective, single-center study in the ICU of the Le Mans Hospital. All patients aged 80 years and older admitted during the inclusion period were eligible. Exclusion criteria were: the absence of organ failure during the stay, an absence of specific ICU therapy implemented during the stay (exclusive invasive or non-invasive mechanical ventilation, vasopressor support, high flow oxygen therapy, renal replacement therapy), an admission for scheduled surgery or for a complication related to scheduled surgery, the need for transfer to an expert center (neurosurgery, cardiac surgery) or an admission in anticipation of an organ donation.

**Results: **From January 2015 to December 2019, among 569 patients aged 80 years and older, admitted to the ICU of our hospital, 393 were included. The mean age was 83.71 ± 3.07 years; the mean SAPS-2 score was 55.66 ± 19.99; 351 patients (90.93%) lived at home; 223 patients (62.29%) had a Clinical Frailty Score (CFS) ≤ 4; 47 patients (13.62%) had a home care nurse; the mean Charlson score was 5.70 ± 1.67 with a median of 5 (IQR 4–7). Overall, one-year mortality rate was 57.25%. The ICU mortality and hospital mortality rates were 41.73% and 49.56% respectively. In multivariate analysis, SAPS-2 score (HR 1.039 [1.028–1.050], p < 0.0001), age (HR 1.077 [1.024–1.133], p = 0.004) and passage of a home care nurse (HR 1.766 [1.089–2.866], p = 0.021) were significantly associated with an increase of one-year mortality rate. All patients were treated for at least one organ failure, of which 181 (46.06%) received vasopressor support, 275 (69.98%) received invasive mechanical ventilation (19.59%) and 77 patients (19.59%) received exclusive non-invasive mechanical ventilation.

**Conclusion: **In this observational study conducted in a medical-surgical intensive care unit between 2015 and 2019, the one-year mortality rate was 57.25% in patients aged 80 years and over with at least one organ failure. This mortality rate was significantly correlated with age, high severity score and loss of autonomy characterized by a home care nurse.

**Reference 1**

Guidet B,et al. The contribution of frailty, cognition, activity of daily life and comorbidities on outcome in acutely admitted patients over 80 years in European ICUs: the VIP2 study. Intensive Care Med.2020.

**Reference 2**

Demiselle J et al. Determinants of hospital and one-year mortality among older patients admitted to intensive care units: results from the multicentric SENIOREA cohort. Annals of Intensive Care. 2021.

**Compliance with ethics regulations:** Yes in clinical research.

## FC-028 Psychic trauma and resilience process of patients after severe critical illness requiring mechanical ventilation: the Resirea study

### Alice Mathieu^1^, Jean Reignier ^1^, Amélie Le Gouge^2^, Gaetan Plantefeve^3^, Jean Pierre Quenot^4^, Virginie Maxime^6^, Laurent Argaud^5^, Pierre Asfar^7^, Julio Badie^12^, Nicolae Vlad Botoc^10^, Hoang Nam Bui^8^, Delphine Chatellier^9^, Louis Chauvelot^5^, Michael Darmon^13^, Agathe Delbove^14^, Jérôme Devaquet^15^, Louis Marie Dumont^16^, Olivier Gontier^17^, Samuel Groyer^18^, Laurent Guérin^19^, Yannick Hourmant^1^, Samir Jaber^20^, Fabien Lambiotte^21^, Christophe Leroy^22^, Benjamin Madeux^23^, Julien Maizel^24^, Olivier Martinet^25^, Frédéric Martino^26^, Emmanuelle Mercier^27^, Jean Paul Mira^28^, Mai-Anh Nay^29^, Saad Nseir^30^, Gael Piton^31^, Anne Renault^32^, Jean Philippe Rigaud^33^, Francis Schneider^34^, David Schnell^35^, Michel Sirodot^36^, Bertrand Souweine^37^, Fabienne Tamion^38^, Didier Thévenin^39^, Guillaume Thiery^40^, Nathalie Thieulot-Rolin^41^, Jean François Timsit^42^, François Tinturier^24^, Patrice Tirot^43^, Isabelle Vinatier^44^, Christophe Vinsonneau^45^, Alexandra Laurent^4^

#### ^1^CHU Nantes, Nantes, France; ^2^CHU de Tours, Tours, France; ^3^Centre Hospitalier d'Argenteuil, Argenteuil, France; ^4^CHU de Dijon, Dijon, France; ^5^CHU de Lyon, Lyon, France; ^6^Hôpital Raymond Poincaré, Paris, France; ^7^CHU Angers, Angers, France; ^8^CHU Bordeaux, Bordeaux, France; ^9^CHU Poitiers, Poitiers, France; ^10^CH Saint Malo, Saint Malo, France; ^11^CH Montauban, Montauban, France; ^12^Hôpital de Belfort, Belfort, France; ^13^CHU Saint Louis, Paris, France; ^14^CH Bretagne Atlantique, Vannes, France; ^15^Hôpital Foch, Suresnes, France; ^16^Hôpital Louis-Mourier, Colombes, France; ^17^CH de Chartres, Chartres, France; ^18^CH de Montauban, Montauban, France; ^19^CHU Bicêtre, Paris, France; ^20^CHU Montpellier, Montpellier, France; ^21^CH Valenciennes, Valenciennes, France; ^22^CH Emile Roux, Le Puy En Velay, France; ^23^CH de Bigorre, Tarbes, France; ^24^CHU Amiens Picardie, Amiens, France; ^25^CHU La Réunion, Saint Denis, France; ^26^CHU Pointe à Pitre, Pointe À Pitre, France; ^27^CHU Bretonneau, Tours, France; ^28^Hôpital Cochin, Paris, France; ^29^CHR Orléans, Orléans, France; ^30^CHU Lille, Lille, France; ^31^CHU Besançon, Besançon, France; ^32^CHU La Cavale Blanche, Brest, France; ^33^CH Dieppe, Dieppe, France; ^34^CHU Strasbourg, Strasbourg, France; ^35^CH Angoulême, Angoulême, France; ^36^CH Annecy Genevois, Pringy, France; ^37^CHU Gabriel Montpied, Clermont Ferrand, France; ^38^CHU Rouen, Rouen, France; ^39^CH Lens, Lens, France; ^40^CH Saint Etienne, Saint Priest En Jarez, France; ^41^CH Marc Jacquet, Melun, France; ^42^CHU Bichat, Paris, France; ^43^CH Le Mans, Le Mans, France; ^44^CHD La Roche sur Yon, La Roche Sur Yon, France; ^45^CH Béthune, Béthune, France

##### **Correspondence:** Alice Mathieu (alicewanda@wanadoo.fr)

*Annals of Intensive Care *2013, **13(Suppl 1)**:FC-028

**Rationale:** Patients admitted to intensive care unit (ICU) for severe critical illness experience multi-organ failure with high risk of death. This condition can lead to a potentially long-lasting post-traumatic stress disorder (PTSD). The RésiRéa study aimed to identify patients with PTSD after ICU stay, to assess resilience skills and to have a better understanding in the involvement of some factors in the resilience process such as social support, illness perception or quality of life.

**Patients and methods/materials and methods:** RésiRéa was a prospective and longitudinal study involving patients admitted in 42 ICUs in France. RESIREA was a parallel study of the NUTRIREA-3 trial, which included patients treated with mechanical ventilation and vasoactive amine for shock. Three months and one year after ICU admission, patients were asked to complete five scales assessing PTSD (IES-R), resilience (CDRISC), quality of life (SF-36), illness perception (BIPQ) and social support (MSPSS). Resilience was defined by a score at CDRISC ≥ 80 and a low IESR score (absence of post-traumatic stress).

**Results:** 380 patients were included: 125 women (32.9%), 61 ± 13 years old, SAPS-2 56 ± 18, SOFA 10 ± 3. 22.1% [17.9; 26.9] had PTSD at three months and 17.3% [12.6; 22.9] at one year. The rate of patients with CDRISC score ≥ 80 was 18.0% [14.0; 22.6] at three months and 24.3% [18.8; 30.5] at one year. The rate of resilient patients was 12.6% [9.5; 16.4]. In comparison to non-resilient patients, resilient patients had a higher score of social support at three months (MSPSS 78.9 ± 9.3 vs. 70.9 ± 14.3, p < 0.001), a better perception of illness (BIPQ 27.7 ± 8.8 vs 38.2 ± 12.6, p < 0.001) and a better quality of life (PCS 44.1 ± 10.6 vs 38.9 ± 10.8, p = 0.009; MCS 55.3 ± 8.1 vs 47.6 ± 12.4, p < 0.001). These differences still remained one year after inclusion in the study.

**Conclusion:** Resilience seems to be an important indicator to consider when focusing on how to better support patients after an ICU stay and to reduce the risks of PTSD or increase quality of life. Resilience in ICU patients seems to be influenced by social support and illness perception.

**Compliance with ethics regulations:** Yes in clinical research.

## FC-029 Association between physical strength and quality of life three months after ICU discharge

### Maite Agbakou^1^, Jeremie Lemarie^1^, Amélie Seguin^1^, Maëlle Martin^1^, Olivier Zambon^1^, Gauthier Blonz^1^, Luc Desmedt^1^, Robin Souron^2^, Camille Juhel^1^, Jean Reignier^1^, Emmanuel Canet^1^, Jean Baptiste Lascarrou^1^

#### ^1^Centre Hospitalier Universitaire de Nantes, Nantes, France; ^2^Nantes Université, Nantes, France

##### **Correspondence:** Maite Lacou Agbakou (maite.agbakou@gmail.com)

*Annals of Intensive Care *2013, **13(Suppl 1)**:FC-029

**Rationale:** Critical illness survivors are likely to suffer from Intensive Care Unit (ICU) acquired weakness as part of the Post Intensive Care Syndrome (PICS), and poor quality of life (QOL). Handgrip strength (HGS) measured by dynamometer is an easy and reproducible test which reflects global muscle status. We aim to assess the association between HGS at three months after ICU discharge and QOL.

**Patients and methods/materials and methods:** This monocentric retrospective descriptive study was set in a French University Hospital medical ICU. Our team holds post ICU medical visits (PIMV) three months after ICU discharge for each patient who suffered from a cardiac arrest or underwent mechanical ventilation for at least 48 h. During this PMIV, a multimodal evaluation is performed, including a HGS measure with a handheld Jamar hydraulic dynamometer, as well as a QOL questionnaire, the Short Form 36 Health Survey (SF36). We retrospectively collected data from our PIMV register.

**Results:** 152 patients were seen in PIMV between 8th of August 2020 and 16th of September 2022. There were 108 (71%) men, with a median age of 58 15 years old. Main reason of the PIMV was MV longer than 48 h (n = 131) followed by cardiac arrest (n = 21; most of them received MV > 48 h). The HGS was performed for 121 patients with a median value index of 28.8 kg [22–37.8]. We split our population into two subgroups: strong (HGS ≥ 30 kg) and weak patients (HGS < 30 kg). Differences between strong and weak patients appear in Table 1.

**Conclusion:** Muscular strength assessed by HGS at three months after ICU discharge is associated with lower cognition but increased physical component of SF36. This result emphasizes the need for interventions promoting physical recovery after ICU discharge in order to improve patients centered outcome.

**Compliance with ethics regulations:** Yes in clinical research.

**Table 1 (abstract FC-029):**
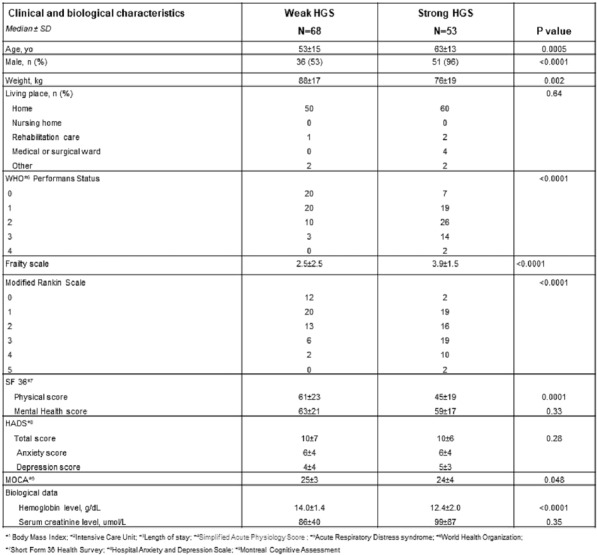
Clinical and biological characteristics of weak and strong patients at 3 months after ICU discharge

## FC-030 Post-ICU consultation: a time to discuss advance directives? The CoPRADA study

### Estelle Burban^1^, Anne Renault^1^

#### ^1^CHU de Brest, Brest, France

##### **Correspondence:** Estelle Burban (stl.burban@gmail.com)

*Annals of Intensive Care *2013, **13(Suppl 1)**:FC-030

**Rationale:** After a stay in intensive care unit (ICU), patients can experience physical, psychological and cognitive impairments. To assess this post intensive care syndrom (PICS), post-ICU consultations have been developed. Since 5-year mortality is higher in ICU survivors, it might be interesting to address those patients' views relating to illness and end of life through a discussion on advance directives (AD), during post-ICU consultation. The aim of this study is to assess the possibility of discussing AD during post-ICU consultation.

**Patients and methods/materials and methods:** This is a single-center prospective, observational study conducted in a French medical ICU. All patients with at least 48 h of mechanical ventilation and/or a 5-day stay in ICU, and then summoned to post-ICU consultation, were included. The primary endpoint was the number of patients willing to discuss AD after an ICU stay. Secondary endpoints were the number of patients knowing about AD before their ICU stay, the factors influencing their writing and the evaluation of the privileged interlocutor to discuss and then complete AD.

**Results:** A total of 47 patients were included: 72% had never heard of AD and among those knowing about AD, only three had written theirs. For 29 patients (66%), post-ICU consultation appears to be an appropriate time for the discussion of AD. Twenty-one patients (47.7%) relied on a written form to inform them about AD; 15 (34%) on a specialist and seven (15,9%) on their general practitioner. They planned to write AD with their loved one (40.9%) or their attending physician (38.6%) before a specialist, including an ICU practitioner (29.5%). At the end of the consultation, 12 patients (27%) were considering writing AD, 18 patients (40.9%) had no intention to write theirs; the 14 others could not position themselves. Univariate or multivariate analysis did not make it possible to define factors promoting the will to write AD (Figure 1).

**Conclusion:** As 66% of patients thought the discussion of AD conceivable, the implementation of this discussion should be considered during the post-ICU consultation. However, few patients seem to contemplate their writing following this appointment. Today, more than encouraging the writing of AD, the reflection should evolve towards the development of "Advance Care Planning": a possibility for the patient to reflect gradually on his therapeutic project and end of life, with a reflection which could be initiated in the post-ICU consultation.

**Reference 1**

Rigaud, J.-P., GELINOTTE, S., BEUZELIN, M., MARCHALOT, A., ERALDI, J.-P., BOUGEROL, F., ECARNOT, F., QUENOT, J.-P., & DECLERCQ, P.-L. (2022). Post-ICU consultation: an indispensable tool in 2022. Médecine Intensive Réanimation, 31(Hors-série1), 79–86.

**Reference 2**

Lone NI, Walsh TS. Impact of intensive care unit organ failures on mortality during the five years after a critical illness. Am J Respir Crit Care Med. 2012 Oct 1;186(7):640–7. https://doi.org/10.1164/rccm.201201-0059OC. Epub 2012 Jul 26. PMID: 22837381.

**Compliance with ethics regulations:** Yes in clinical research.

**Figure 1 (abstract FC-030):**
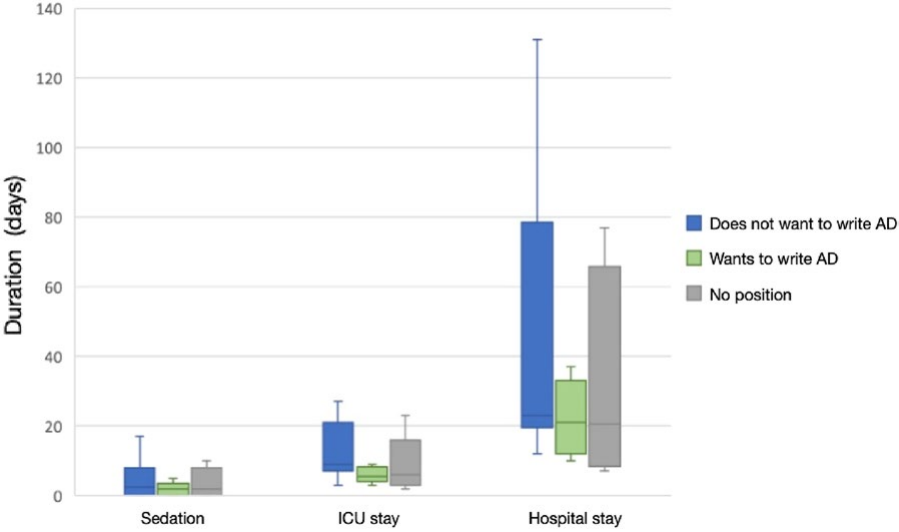
Factors promoting the will to write AD

## FC-031 Impact of acute kidney injury occurrence in critical care on long-term prognosis in patients with hematological malignancies

### Nacim Benchabane^1^, Laura Platon^1^, Caroline Mollevi^1^, Fanchon Herman^1^, Eddine Bendiab^1^, Mickael Francois^1^, Delphine Daubin^1^, Noémie Besnard^1^, Vincent Brunot^1^, Romaric Larcher^1^, Jean-Jacques Tudesq^1^, Patrice Ceballos^1^, Charles Herbaux^1^, Kada Klouche^1^

#### ^1^CHU Lapeyronie, Montpellier, France

##### **Correspondence:** Nacim Benchabane (nacim.benchabane@gmail.com)

*Annals of Intensive Care *2013, **13(Suppl 1)**:FC-031

**Rationale:** Acute kidney injury (AKI) is associated with high morbidity and mortality and 75% of patients with hematological malignancies (HM) are at higher risk. We aimed to evaluate the impact on long-term prognosis of the occurrence of AKI in patients with HM who survived their critical care admission.

**Patients and methods:** This is a single-center retrospective cohort study that included patients with HM, admitted to the Intensive Care Unit (ICU) between January 1, 2012 and December 31, 2020. Two groups were distinguished according to the occurrence of AKI. AKI was defined according to the Kidney Disease: Improving Global Outcomes (KDIGO) criterias. The primary outcome was 3-year mortality.

**Results:** A cohort of 251 patients was analyzed: 64.5% were males and the median age was 63 [52; 69] years old. The median Sequential Organ Failure Assessment was 5 [4; 8]. AKI occurred in 58.6% of the patients, including 36.7% KDIGO stage 1, 31.3% KDIGO stage 2 and 32% KDIGO stage 3. Among these, 25.6% required renal replacement therapy (RRT). Three-year mortality was 57.7% in patients without AKI and 66.7% in patients with AKI (p = 0.27). There was a non-significant trend towards excessive 3-year mortality in patients who required RRT compared to those without AKI or KDIGO stage 1 (78.9% vs. 58.9%, p = 0.07). Recovery of previous renal function was 86.8% without AKI and 69.9% with AKI (p < 0.01). In patients developing AKI, 59.9% were able to continue their hematological course versus 73% without AKI (p = 0.03).

**Conclusion:** AKI affects more than half of the patients with HM admitted to the ICU. No significant impact on mortality was observed until three years after ICU discharge. Most of the patients recovered their previous renal function. The occurrence of AKI during the ICU stay did not significantly impact the long course of the hematological disease.

**Compliance with ethics regulations:** Yes in clinical research.

**Table 1 (abstract FC-031):**
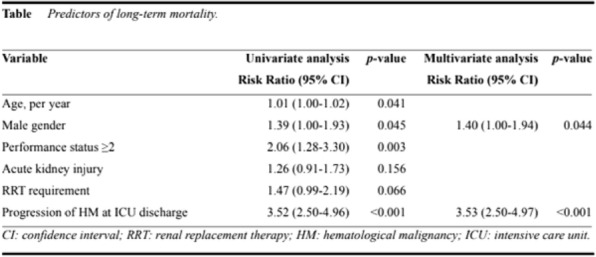
Predictors of long-term mortality

## FC-032 Importance of clinical and biological clusters among ICU-patients with acute myeloid leukaemia requiring chemotherapy

### Guillaume Rigault^1,2^, Louis Marie Galerneau^1^, Florian Sigaud^1^, Martin Carre^3^, Lucile Bussot^2,3^, Carole Schwebel^1,2^, Guillaume Dumas^1,2^

#### ^1^Médecine Intensive-Réanimation—CHU Grenoble Alpes, La Tronche, France; ^2^Université Grenoble-Alpes, Grenoble, France; ^3^Hématologie—CHU Grenoble Alpes, La Tronche, France

##### **Correspondence:** Guillaume Rigault (grigault@chu-grenoble.fr)

*Annals of Intensive Care *2013, **13(Suppl 1)**:FC-032

**Rationale:** Patients with Acute myeloid leukemia (AML) are at high risk of life-threatening complications, leading to intensive care unit (ICU) admission. In this context, mortality remains high, and identifying patients who will benefit the most from early ICU admission is of major concern. In this study, we sought to identify different clusters of patients using their clinical and biological characteristics and investigate their association with outcome.

**Patients and methods/materials and methods:** We designed an observational, retrospective, monocentric study including consecutive patients with an AML diagnosis admitted in ICU between 2010 and 2022 in a tertiary teaching Hospital. We used a hierarchical clustering method including clinical and biological characteristics available at admission to identify patient sub-phenotypes. The associations between clusters and both 28-day mortality and the need for life-sustaining therapies during ICU stay were evaluated.

**Results:** Overall, 73 patients with a diagnosis of AML (age 58.2 [47.25—65.82] years, 47.9% male) were included. Two different clusters were identified. Patients in Cluster 1 (N = 37) referred from another hospital, were significantly younger with less comorbidities than patients in Cluster 2 (N = 36) exhibiting higher proportion of hyperleukocytosis. Patients in cluster 2 presented more hypotension, acute renal failure, or coma and demonstrated higher risk for oxygen therapy requirement. Specific management of AML did not differ across clusters, respected to leukocytapheresis use (73 vs 63.9%, respectively for Cluster 1 and 2, p 0 0.457) or hydroxycarbamide administration before intravenous chemotherapy initiation (29.7 vs 33.3, p 0 0.804) and the median time to intravenous chemotherapy initiation (6.4 [-1.1—19.2] vs 7.6 [3.4—15.2] hours, p 0.548) was similar. During ICU stay, patients in Cluster 1 were less treated by vasopressor or renal replacement therapy, without any differences about the use of invasive mechanical ventilation. Twenty-eight days mortality was 13.5% and 47.2% for Cluster 1 and 2 respectively (p = 0.002). This difference is still significant after adjustment on SAPSII and the use of invasive mechanical ventilation (HR4.3 [95%IC, 1.5 to 12]).

**Conclusion:** Among an ICU population with a diagnosis of AML, two different clusters with different ICU trajectories and outcomes have been identified. Such variability could help to improve the management of AML patients and guide intensivist decisions most suitable for each patient at the bedside.

**Compliance with ethics regulations:** Yes in clinical research.

## FC-033 Severe infections requiring intensive care unit admission in patients with lymphoproliferative diseases receiving ibrutinib: data from 92 critically-ill patients included in ten French centers

### Louise Baucher ^1^, Virginie Lemiale^1^, Adrien Joseph^1^, Florent Wallet^2^, Marc Pineton De Chambrun^3,4^, Alexis Ferré^5^, Romain Lombardi^6^, Laura Platon^7^, Adrien Contejean^8^, Charline Fuseau^9^, Laure Calvet^10^, Frédéric Pène^11^, Achille Kouatchet^12^, Djamel Mokart^13^, Elie Azoulay^1^, Antoine Lafarge^1^

#### ^1^Médecine Intensive Réanimation, Hôpital Saint Louis, Paris, France; ^2^Médecine Intensive Réanimation, Hospices civils de Lyon, Lyon, France; ^3^Sorbonne Université, Assistance Publique-Hôpitaux de Paris (APHP), Hôpital de la Pitié-Salpêtrière, Service de Médecine Intensive-Réanimation, Paris, France; ^4^Sorbonne Université, INSERM, UMRS_1166-ICAN, Institut de Cardiométabolisme et Nutrition (ICAN), Paris, France; ^5^Réanimation médico-chirurgicale, Centre Hospitalier de Versailles, Le Chesnay, France; ^6^Médecine Intensive Réanimation, Hôpital Pasteur, Nice, France; ^7^Médecine Intensive Réanimation, Hôpital Lapeyronie, Montpellier, France; ^8^Equipe mobile d’infectiologie, Hôpital Cochin, Paris, France; ^9^Hématologie, Institut de Cancérologie (ICANS), Strasbourg, France; ^10^Médecine Intensive Réanimation, Hôpital Gabriel Montpier, Clermont-Ferrand, France; ^11^Médecine Intensive Réanimation, Hôpital Cochin, Paris, France; ^12^Médecine Intensive Réanimation, Hôpital d'Angers, Angers, France; ^13^Anesthésie Réanimation, Institut Paoli Calmette, Marseille, France

##### **Correspondence:** Louise Baucher (louise.baucher@hotmail.fr)

**Rationale:** In the last decade, ibrutinib has become the standard of care in the treatment of several lymphoproliferative diseases. Beyond Bruton tyrosine kinase inhibition, ibrutinib shows broad immunomodulatory effects that may promote the occurrence of infectious complications, including opportunistic infections. The infectious burden has been shown to vary by disease status, neutropenia and prior therapy but data focusing on severe infections requiring intensive care unit (ICU) admission remain scarce.

**Patients and methods/materials and methods:** The medical records of hematological patients receiving ibrutinib admitted to 10 ICU departments in France from January 1st, 2015 to December 31st, 2020 were retrospectively analyzed. Severe infections in patients receiving ibrutinib were analyzed and compared to severe infections in patients receiving conventional chemotherapies from an historic cohort (TRIAL-OH^1^) matched on age and underlying malignancy.

**Results:** Among 92 critically-ill patients receiving ibrutinib, 69 (75%) patients were admitted to the ICU for severe infections. The most common underlying malignancy was chronic lymphocytic leukemia (59%). Forty-seven (68%) patients received ibrutinib as monotherapy. The median interval from ibrutinib initiation to ICU admission was 6.6 [3–18] months. Severe infections were mostly bacterial infections (71%), while viral and fungal infections accounted for 17% and 12% respectively. Opportunistic infections included invasive aspergillosis in six (9%) patients and Pneumocystis pneumonia in two (3%) patients. Thirty-nine (57%) patients received vasopressors and 28 (41%) required mechanical ventilation. Twenty (29%) patients died in the ICU and day-90 mortality reached 55%. In comparison with decedents, survivors displayed more severe organ dysfunctions at ICU admission (SOFA 7 [5–11] vs. 4 [3–7]; P = 0.004) but there was no difference regarding age, underlying malignancy, prior therapy and infection type. Sixty-three patients receiving ibrutinib were matched with 63 patients receiving conventional chemotherapy. Age, sex ratio and underlying malignancies did not differ between the two groups. Among these patients, chronic lymphocytic leukemia was the most common underlying disease (65% and 64% respectively). Despite a higher median number of prior treatment regimens (3 vs. 1; P < 0.001) and a higher rate of fungal [13% vs. 8%] and viral [19% vs. 5%] infections; [P = 0.023], patients receiving ibrutinib displayed similar outcomes in comparison with patients receiving conventional chemotherapies (ICU mortality: 27% vs. 38% [P = 0.254]; day-90 mortality: 52% vs. 48% [P = 0.785]).

**Conclusion:** In hematological patients receiving ibrutinib, severe infections requiring ICU admission are associated with a dismal prognosis, mostly impacted by initial organ dysfunctions. Due to the high proportion of fungal infections, opportunistic agents should be routinely screened by ICU clinicians in this immunocompromised population.

**Reference 1**

Azoulay E, Mokart D, Pène F, Lambert J, Kouatchet A, Mayaux J, Vincent F, Nyunga M, Bruneel F, Laisne LM, Rabbat A, Lebert C, Perez P, Chaize M, Renault A, Meert AP, Benoit D, Hamidfar R, Jourdain M, Darmon M, Schlemmer B, Chevret S, Lemiale V. Outcomes o.

**Compliance with ethics regulations:** Yes in clinical research.

## FC-034 High prevalence of pre-existing sarcopenia in critically ill patients with hematologic malignancies admitted to the intensive care unit for sepsis or septic shock

### Antoine Herault^1^, Emilie Leveque^2^, Simon Draye-Carbonnier^2^, Alexandra Zduniak^2^, Pierre Decazes^2^, Romain Modzelewski^2^, Julie Libraire^2^, Najate Achamrah^1^, Anne-Lise Menard^2^, Pascal Lenain^2^, Nathalie Contentin^2^, Maximilien Grall^1^, Stéphane Lepretre^2^, Emilie Lemasle^2^, Helene Lanic^2^, Mustafa Alani^2^, Aspasia Stamatoullas-Bastard^2^, Herve Tilly^2^, Fabrice Jardin^2^, Fabienne Tamion^1^, Vincent Camus^2^

#### ^1^CHU Rouen, Rouen, France; ^2^Centre Henri Becquerel, Rouen, France

##### **Correspondence:** Antoine Herault (antoine-herault@hotmail.fr)

*Annals of Intensive Care *2013, **13(Suppl 1)**:FC-034

**Rationale:** Severe infections requiring admission to the intensive care unit (ICU) often lead to rapid loss of skeletal muscle mass. These changes in body composition can be measured by computed tomography (CT) images. The objective of this study was to evaluate the prognostic impact of pre-existing sarcopenia measured by CT in patients with hematologic malignancies (HM) admitted to the ICU for sepsis or septic shock.

**Patients and methods/materials and methods:** We performed a retrospective study of 186 patients with HM who had a first hospitalization in ICU between 2013 and 2020 at the Rouen University Hospital for sepsis or septic shock. We used CT images collected as part of routine care in the 2 months preceding ICU admission to assess baseline body composition by manual segmentation of slices on the third lumbar vertebra (L3) and twelfth thoracic vertebra (T12).

**Results:** The mean age of patients was 54.1 (15.5) years. The sex ratio (M/F) was 1.90. Patients displayed severity criteria at admission: median SAPS II [q1; q3] of 52 [40;66] and a median SOFA score of 8 [5;12]. We classified as sarcopenic 64.9% of patients on L3 slices and 83% on T12 slices on baseline CT images. There was a good correlation between skeletal muscle measurements on L3 and T12 sections (r = 0.77). The raw ICU mortality rate was 45.7%. The overall survival rate at 1 month from ICU admission in patients with pre-existing sarcopenia versus patients without pre-existing sarcopenia was 47.9% (95% IC [37.6; 61.0]) versus 55.0% (95% IC [41.6; 72.8]), p = 0.99) for measurements performed in L3; and 48.4% (95% IC [40.4; 58.0]) versus 66.7% (95% IC [51.1; 87.0], p = 0.062) for measurements performed in T12, respectively.

**Discussion:** Mortality rate in our cohort of HM patients with severe infection was particularly high. The majority of patients in the cohort had pre-existing sarcopenia before ICU admission. Patients with T12-assessed pre-existing sarcopenia had a lower overall survival. Pre-existing sarcopenia might be influenced by the underlying HM. We observed hydro sodic inflation responsible for tissue edema that occasionally hampered the manual segmentation and body composition assessment on L3 images.

**Conclusion:** Pre-existing sarcopenia may be regarded as a risk factor for severe infection in HM patients and contributed to the high mortality observed in this ICU population.

**Compliance with ethics regulations:** Yes in clinical research.

## FC-035 The clinical picture of ICU patients diagnosed with both hemophagocytic lymphohistiocytosis and systemic lupus erythematosus

### Julien Schmidt^1^, Guillaume Millot^2^, Julien Dessajan^3^, Decavele Maxens^4^, Martino Frédéric^5^, Stéphane Legriel^6^, Florent Wallet^7^, Clara Vigneron^8^, Julien Moury^9^, Damien Roux^10^, Yves Cohen^1^

#### ^1^CHU Avicenne, Bobigny, France; ^2^CHRU Lille, Lille, France; ^3^Hôpital Bichat, Paris, France; ^4^Hôpital Pitié Salpêtrière, Paris, France; ^5^CHU de Pointe-à-Pitre, Pointe-à-Pitre, France; ^6^CHSF, Corbeille-Essonnes, France; ^7^CHU Lyon, Lyon, France; ^8^Hôpital Cochin, Paris, France; ^9^Hôpital Saint Luc, Namur, Belgique; ^10^Hôpital Louis Mourier, Colombes, France

##### **Correspondence:** Julien Schmidt (julien.schmidt@aphp.fr)

*Annals of Intensive Care *2013, **13(Suppl 1)**:FC-035

**Rationale:** Hemophagocytic syndrome (HS), a rare and life-threatening disease, encompasses a wide spectrum of heterogeneous conditions, all resulting in severe systemic inflammation. Systemic lupus erythematosus (SLE) flare-up can mimic reactive HS, both entities sharing common features can frequently lead to ICU admission.

**Patients and Methods:** This study aims to focus on SLE patients with reactive HS admitted to ICU, regarding clinical, laboratory and pathological features as well as treatment strategies and prognosis. It is a retrospective multicenter study including SLE patients with reactive HS in 9 French and 1 Belgian ICUs over 12 years.

**Results:** Twenty-one patients were included in the study. Main reason for ICU transfer was hemodynamic failure in 52.4% of patients, acute respiratory failure in 19%, altered mental state in 14.3%, acute liver failure in 4.8% and monitoring in 9.6%. Median HS score was 201 and was > 168 in 100% of patients. Besides SLE, another associated condition was found in 12 (57.1%) patients. 95.2% of patients were treated with corticosteroids. Etoposide was used in 47.6% of patients, cyclophosphamide in 33.3%, MMF in 9.5%, plasma exchange therapy in 14.3%, tacrolimus, rituximab and polyvalent immunoglobulins in 4.8% each. Catecholamine infusion was used in 52.4% of patients; 52.4% of patients underwent invasive mechanical ventilation; 19% of patients required renal replacement therapy. Overall ICU mortality was 9.6% and overall 28-days mortality was 14.3%.

**Discussion:** Reactive HS is a rare complication of SLE, inaugural in more than half of patients; distinction between SLE flare-up, sepsis and non SLE-related reactive HS is tricky, as most clinical and biological symptoms are non-specific. Yet, given the significant need for organ support therapy among young patients without comorbidities, early recognition is crucial. To that extent, multi-organ failure in young patients with suggestive clinical and biological signs should promptly lead to a biological and immunological workup to assess for both SLE and reactive HS; ferritin assessment may be especially useful in this setting when cytopenia tends to be less frequent. Severe bleeding events seem to be frequent and should be considered before performing non-urgent invasive procedures. Front-line therapy needs to be clarified, are both SLE and reactive HS with severe presentation require distinct kinds of immunosuppressive therapy; randomized controlled trials and prospective data are most needed.

**Conclusion:** Reactive HS is a rare complication of SLE. Yet, given the significant need for organ support therapy among young patients without comorbidities, early recognition is crucial. Front-line therapy needs to be clarified.

**Compliance with ethics regulations:** Yes in clinical research.

## FC-036 Antiphospholipid status in critically-ill COVID-19 patients: profile and correlation with thromboembolic complications

### Aïcha Rebai^1^, Wafa Anene^1^, Zied Hajjej^1^, Khadija Bahrini^1^, Yasmine Boukhalfa^1^, Iheb Labbene^1^, Mustapha Ferjani^1^

#### ^1^Hôpital Militaire Principal d'Instruction de Tunis, Tunis, Tunisie

##### **Correspondence:** Aïcha Rebai (aicha.rebai@fmt.utm.tn)

*Annals of Intensive Care *2013, **13(Suppl 1)**:FC-036

**Rationale:** Since the beginning COVID-19 pandemic, an increased risk of venous or arterial thrombotic events has been observed in infected patients [1]. Many studies have described the presence of anti-phospholipid antibodies (aPL). Aim of the study: This study aimed to determine the prevalence and the kinetics of APL in COVID-19 patients and their correlation to thromboembolic disease.

**Patients and methods/materials and methods:** In a prospective study, 43 critically-ill COVID-19 admitted to the intensive care unit of the Military Hospital of Tunis have been consecutively enrolled from October 01, 2020 to April 30, 2021. Anti-cardiolipin antibodies (aCL), antiphosphatidylserine antibodies (aPS), D-Dimers, fibrinogen and C-reactive protein (CRP) have been measured with enzyme-linked immunosorbent assay and the SOFA score, the Disseminated Intravascular Coagulation score (DIC) and the Sepsis Induced Coagulopathy score (SIC) were estimated on day 1, 3, 5, 8 and 10 for each patient. At a final point, patients were compared based on the occurrence of a thromboembolic event. Data were analyzed with SPSS version 25.0.

**Results:** 58% (25/43) of the study population were positive for APL distributed as follow: 23 had positive aCL, 12 positive aPS and 10 were positive for aCL and aPS. When comparing patients based on the presence of APL, there was no significant difference between the demographic criteria between the two groups. The length of stay in intensive care was 19 ± 9 days and the mortality rate was 79%. The severity scores on admission, at D3, D5, D8 and D10 were comparable between the two groups. Thirteen patients presented a thromboembolic event, ie 30.23% of the population, with no significant difference between the two populations.

**Discussion:** The incidence of APL and their correlation to thromboembolic events in COVID19 patients varies from one population to another; while our results are consistent with some previous studies, others have reported a cumulative incidence of thrombotic complications in patients with a severe form of COVID-19 admitted to intensive care.

**Conclusion:** In our study, there was no correlation between APL status and the occurrence of thromboembolic events. To be confirmed, it would be essential to carry out a multicenter study with a larger workforce, by measuring the different subtypes of APL and correlating them with the occurrence of thrombosis, but also with the evolution of the pro-coagulant inflammatory status of COVID19 patients.

**Reference 1**

Bilaloglu S, Aphinyanaphongs Y, Jones S, Iturrate E, Hochman J, Berger JS. Thrombosis in Hospitalized Patients With COVID-19 in a New York City Health System. JAMA. 2020;324(8):799–801.

**Compliance with ethics regulations:** Yes in clinical research.

**Table 1 (abstract FC-036):**
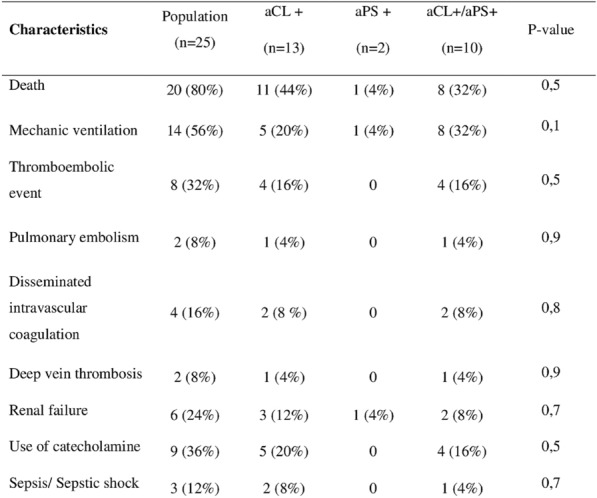
Correlation between APL status and thromboembolic event’s occurrence

## FC-037 Reversible microvascular hyporeactivity in patients with immune-mediated thrombocytopenic thrombotic purpura

### Jeremie Joffre^1^, Lisa Raia^1^, Tomas Urbina^1^, Vincent Bonny^1^, Paul Gabarre^2^, Louai Missri^1^, Jean-Luc Baudel^1^, Paul Coppo^1^, Bertrand Guidet^1^, Eric Maury^1^, Hafid Ait-Oufella^1^

#### ^1^Hôpital Saint-Antoine APHP, Paris, France; ^2^hôpital Tenon—APHP, Paris, France

##### **Correspondence:** Jeremie Joffre (jeremie.joffre@aphp.fr)

*Annals of Intensive Care *2013, **13(Suppl 1)**:FC-037

**Rationale:** Immune-mediated thrombotic thrombocytopenic purpura (iTTP) is a rare disease characterized by arteriolar and capillary microthrombosis precipitating organ failure. However, the contribution of endothelial dysfunction on impaired microvascular blood flow in iTTP patients has been poorly explored. This pilot observational study aimed to explore endothelial-mediated vasoreactivity in iTTP patients at admission and its changes after plasma exchange therapy (PE).

**Patients and methods/materials and methods:** We conducted a prospective observational study in patients (> 18-yr old) admitted in ICU for iTTP. Using laser Doppler flowmetry and iontophoretic acetylcholine (Ach) in the forearm, we recorded the skin microvascular blood flow and the endothelium-mediated vasoreactivity at admission and after PE. Demographics, biological, clinical courses, and outcomes were also collected. As a control group, we used a previously published cohort of young diabetic patients after correction of ketoacidosis.

**Results:** Eighteen confirmed iTTP patients and 34 controls were included in the study, mainly female (72%) aged 43 ± 16-year-old. At admission, 55% had neurological abnormalities, 50% cardiac issues and 27.8% an acute kidney injury. Median platelet count was 19 G/mL [10–37]. Baseline microvascular blood flow was decreased in iTTP patients when compared to controls (5.97 ± 4.5 vs. 10.1 ± 6.3 PU, P = 0.03), associated with markedly impaired endothelial-mediated skin microvascular reactivity (AUC: 9627 ± 8122 vs. 16,475 ± 11,738, P = 0.03). Microvascular reactivity improved after the first PE session (AUC: 9627 ± 8122 vs 16,558 ± 10,699, P = 0.007, respectively baseline and post PE1) and much more after the second session (26,431 ± 23,181, P = 0.04 post PE1 vs post PE2). Hemolysis biomarkers (LDH and bilirubin) negatively correlates with microvascular flow and vasoreactivity.

**Conclusion:** We highlighted a reversible marked endothelium-mediated microvascular hyporeactivity in iTTP patients that could participate in organ injury pathophysiology.

**Compliance with ethics regulations:** Yes in clinical research.

## FC-038 Evaluation of the management of oncological patients under immune checkpoint inhibitors presenting at the emergency department

### Francesco Pini^1^, Bogdan Grigoriu^1^, Ameye Lieveke ^1^, Anne-Pascale Meert^1^

#### ^1^Institut Jules Bordet, Bruxelles, Belgique

##### **Correspondence:** Francesco Pini (francesco.pini@hotmail.com)

*Annals of Intensive Care *2013, **13(Suppl 1)**:FC-038

**Rationale:** With the rising use of immune checkpoint inhibitors (ICIs) in cancer treatment, emergency physicians are increasingly confronted with their adverse effects. These immune related adverse events (irAEs) can affect multiple organs and present in a variety of ways, making their diagnosis complex. This study aims to evaluate the acute management of patients undergoing an ICI treatment and presenting to the emergency department (ED).

**Patients and Methods:** A retrospective analysis of emergency room visits of patients on ICI was performed in an oncology referral center. The study was spread over a period of four years, from 15/12/2016 to 06/12/2020. The primary objective was to evaluate the chief complaint for consultation at the ED. The secondary objectives were to characterize irAEs, to describe the therapeutic management, as well as to evaluate survival 7-day and 30-day post-consultation.

**Results:** 227 patients on ICI presented to the ED, with a total of 452 visits. 55 (12.2%) of the visits resulted in a diagnosis of irAE, from which 44 (80%) were suspected by emergency physicians. The most common irAEs were colitis and diarrhea (25.5%), followed by pneumonia (23.6%), hepatitis (9.1%), and dermatitis (9.1%). Two chief complaints were associated with an irAE: gastro-intestinal complaints (p < 0.006) and skin rashes (p < 0.02). Another association was found between an irAE and three different factors: a cancer status in remission (OR = 5.43, 95% CI 2.75–10.7), a combination of two ICIs (OR = 4.41, 95% 2.08–9.37) and any medical history of irAE (OR = 2.34, 95% 1.26–4.3).

**Conclusion:** Diagnosing an irAE can be particularly complicated in the emergency department. Two chief complaints should raise the suspicion of physicians of facing an adverse event: gastro-intestinal and dermatological ones. It is also essential to look for predictive factors of developing an adverse effect: any history of irAE, a concomitant use of two ICI or patients with a cancer status in remission.

**Compliance with ethics regulations:** Yes in clinical research.

## FC-039 Impact of pre-existing comorbidities on critically ill patients with COVID-19

### Dhouha Ben Braiek^1^, Hend Zorgati^1^, Yosri Ben Ali^1^, Sourour Bel Haj Youssef^1^, Rihab Boubtane^1^, Nadine Boukadida^1^, Rahma Ben Jazia^2^, Amani Kacem^2^, Jihene Ayachi^1^

#### ^1^Medical Intensive Care Unit, Ibn El Jazzar University Hospital, Kairouan, Tunisie; ^2^Pulmonology Department, Ibn El Jazzar University Hospital, Kairouan, Tunisie

##### **Correspondence:** Jihene Ayachi (ayachijihen@gmail.com)

*Annals of Intensive Care *2013, **13(Suppl 1)**:FC-039

**Rationale:** Morbidity and mortality associated with COVID-19 infection has prompted us to understand potential risk factors that can predict patient outcomes. Little is known about most common underlying diseases in COVID-19 patients and were they really associated with poor outcomes. Aim: To describe the prevalence of underlying diseases in critically ill COVID-19 patients and determine underlying diseases predictors of poor outcomes of COVID-19 infection.

**Patients and methods/materials and methods:** A retrospective analytical cohort study was performed from September 2020 to December 2021 in a 9-bed medical ICU of a university hospital including patients admitted for hypoxemic acute respiratory failure secondary to COVID-19 pneumonia. Characteristics, underling diseases, severity scores, management and outcomes are recorded. Univariate and multivariate analysis were used to identify underlying diseases independently associated with poor outcomes. The primary outcomes were adverse events and mortality.

**Results:** During the study period, 172 patients were included. Mean age was 51.5 ± 14.4 years with male predominance 104 (60.5%). Median SAPSII and Charlson scores were respectively 24 [18–32] and 1 [0–2]. Eighty (46.5%) patients had underlying conditions. The most common comorbidities were obesity 60 (34.9%), diabetes 48 (28.5%) and hypertension 30 (17.4%). Twelve (7%) patients had underlying lung diseases and 5 (2.9%) patients had immunodeficiency. The median PaO_2_/FiO_2_ ratio was 92 [69–136]. Invasive mechanical ventilation was performed in 69 (40.1%) cases. The most frequent adverse events were: shock 68 (39.5%), nosocomial infection 58 (33.7%), acute renal failure 51 (29.7%) and acute metabolic complications of diabetes 31 (18%). Median ICU length of stay was 9 [6–14] days and death rate was at 40.7%. On univariate analysis, two underlying diseases were associated with poor outcomes. Mortality is associated with hypertension (p = 0.005) and diabetes (p = 0.037) and occurrence of adverse events is also associated with hypertension (p = 0.007) and diabetes (p = 0.004). On multivariate analysis, hypertension is an independent predictive factor of mortality (OR, 3; 95%CI, [1.36–6.98]; p = 0.007) and diabetes is independently associated with the occurrence of adverse events (HR, 2.64; 95%CI, [1.34–5.21]; p = 0.005) in critically ill COVID-19 patients.

**Conclusion:** This study showed that patients with pre-existing hypertension and diabetes should take all necessary precautions to avoid getting infected with COVID-19, as they seem to be more vulnerable to developing severe outcomes as occurrence of adverse events and mortality.

**Compliance with ethics regulations:** Yes in clinical research.

## FC-040 COVID19 in critically ill patients Epidemiological, clinical characteristics and outcomes

### Yahyaoui Hajer^1^, Doghri Hamdi Hemdene ^1^, Borsali Falfoul Nebiha^1^

#### ^1^Hôpital Habib Thameur, Tunis, Tunisie

##### **Correspondence:** Yahyaoui Hajer (hajer.yahyaoui94@hotmail.com)

*Annals of Intensive Care *2013, **13(Suppl 1)**:FC-040

**Rationale:** Emerging SARS COV2 has been challenging intensive care units all over the world, not only by the increasing number of admissions, but also by the management of severe forms that require special knowledge of this disease’s characteristics. Our objective is to study epidemiological and clinical features and outcomes of COVID 19 critically ill patients and to identify mortality associated factors.

**Patients and methods/materials and methods:** Descriptive, retrospective and monocentric study including all patients admitted in an intensive care units (ICU) department between 01/09/2020 and 30/06/2022. All patient’s data including demographics, clinical, bacteriological, therapeutical and outcomes were collected.

**Results:** During the period of the study, 450 patients infected by the SARS COV2 were admitted to our unit. Sex ratio was 1.14 and mean age was 63 ± 15. 46.8% and 42.6% of patients had respectively arterial hypertension and diabetes mellitus and 51% had obesity. Mean SAPSII, APACHEII and SOFA score were respectively 30 ± 39, 30 ± 39 and 8,6 ± 5. Diagnosis of COVID 19 infection was made by RT-PCR in 85 cases (19%), by an antigenic test in 250 cases (55.5%), and by a chest CT scan in 115 cases (25.5%). The percentage of lung parenchyma affected in the chest CT scan was less than 25% in 26 patients (6%), 25 to 50% in 118 patients (26.5%), 50 to 75% in 153 patients (34.5) and above 75% in 147 cases (37%). It revealed a pulmonary embolism (PE) in 44 cases. 19.5% of our patients needed invasive mechanical ventilation (IV) (n = 88). Among ICU complications, at least one nosocomial infection occurred in 95 patients, and 77 patients had septic shock. Mean duration of stay was 10.7 days ± 6 and mortality were 35.5%. Several factors associated significantly to mortality were identified: Delay of admission > 12 days (p = 0.049, OR:1.81; CI: 1.O1-321), sever ARDS at admission (p = 0.010, OR:2,74; CI: 1.2–5,7), IV (p < 10^–3^, OR:75,7; CI: 14–407), nosocomial infection (p < 10^–3^, OR: 6,3; 95% CI: 2,5–15,7), sever affection at CT scan ( p = 0,003, OR:18.1; 95% CI: 1.01–12) and APACHE II score > 6 (p = < 10–3, OR: 8,4; 95% CI: 4,6–19,4).

**Conclusion:** Patients admitted in ICU for a SARS COV2 infection are mostly patients with at least one comorbidity. Sever forms predispose to ICU complications and are associated to a higher mortality.

**Compliance with ethics regulations:** Yes in clinical research.

## FC-041 Role of Chest CT in the diagnosis and prognostication of COVID 19 in intensive care units

### Doghri Hamdi Hemdene ^1^, Yahyaoui Hajer^1^, Borsali Falfoul Nebiha^1^

#### ^1^Hôpital Habib Thameur, Tunis, Tunisie

##### **Correspondence:** Yahyaoui Hajer (hajer.yahyaoui94@hotmail.com)

*Annals of Intensive Care *2013, **13(Suppl 1)**:FC-041

**Rationale:** In the COVID 19 pandemic, and since the RT-PCR tests are less available and take more time, it was primordial to find an efficient and rapid way to make the diagnosis of SARS COV2 infection. The chest computed tomography (CT) scan revealing a typical aspect of the COVID 19 had a crucial contribution in this pandemic. Our objective is to describe the role of the chest CT in diagnosis and prognosis in COVID 19 patients admitted in the intensive care unit (ICU).

**Patients and methods/materials and methods:** Descriptive and prospective study including patients admitted in an ICU department between 01/09/2020 and 30/06/2022. All patients data including demographics, clinical, biological, radiographic and outcomes were taken from patient’s medical files.

**Results:** In the period of the study, a total of 450 patients infected by the SARS COV2 were admitted in our department. Sex ratio was 1.14. Mean age, SAPSII, APACHE II and SOFA score were respectively 63 years 30 ± 39, 30 ± 39 and 8.6 ± 5. At admission, 444 patients had a chest CT scan. The diagnosis of COVD 19 pneumonia was made with the contribution of the chest CT in 115 cases. Ground-glass opacities and crazy paving were found respectively in 82% and 70% of cases. Radiological signs of associated bacterial infection were found in 49 cases (11%). Quantification of lung lesions was moderate, important, severe, and critical in 26 (6%), 118 (26.5%), 153 (34.5%), and 147 (33%) patients respectively. Other findings were reported including consolidation (409%). The chest CT revealed 47 cases (10.4%) of pulmonary at admission. Sever ARDS at admission and consultation delay > 12 days were identified as an independent risk factor predictive of a severe to critical pulmonary damage (superior to 50%) on the chest CT with respective (OR: 2.4; 95% CI: 1.5–4.1; p < 10–^3^) and (OR: 7.7; 95% CI: 3.2–18.2; p < 10^–3^). Mortality was 35.5%. Sever to critical pulmonary damage (superior to 50%) on the imaging was associated to a higher mortality (p < 10^–3^).

**Conclusion:** The chest CT was identified as a swift, accessible, and reliable diagnostic modality that served not only as analternative to the RT-PCR but also helped in assessing prognosis by quantifying lung lesions and revealing complications in critical ill patients.

**Compliance with ethics regulations:** Yes in clinical research.

## FC-042 Genetically determined thymic function affects strength and duration of immune response in COVID patients with pneumonia

### Amira Marouf^1^, Hélène Roux^2^, Jacques Dutrieux^2^, Bénédicte Charmeteau-De Muylder^2^, Suzanne Figueiredo-Morgado^2^, Pelagia Cuvelier^1^, Cécile Naudin^1^, Fatma Bouaziz^1^, Anne Couëdel-Courteille^2^, Stefano Marullo^2^, Rémi Cheynier^2^, Guillaume Geri^1^, Pierre Squara^1^

#### ^1^Groupe Hospitalier privé Ambroise Paré-Hartmann, Neuilly Sur Seine, France; ^2^Université Paris Cité, CNRS UMR8104, INSERM U1016, Institut Cochin, Paris, France

##### **Correspondence:** Amira Marouf (amira.marouf.pro@gmail.com)

*Annals of Intensive Care *2013, **13(Suppl 1)**:FC-042

**Rationale:** Thymic activation improves the outcome of COVID-19 patients with severe pneumonia.^1^ The rs2204985 genetic polymorphism within the TCRA-TCRD locus, which affects thymic output in healthy individuals, was found here to influence SARS-CoV-2-specific immunity and disease severity in COVID-19 patients with severe pneumonia.^2^

**Patients and methods/materials and methods:** Forty consecutive patients hospitalized in intensive care unit for severe COVID-19 with pulmonary involvement, who gave written consent for TCRA-TCRD locus DNA sequencing, were included. Thoracic CT-scans were performed for the initial evaluation of pulmonary status. All images were independently reviewed and classified by two radiologists. DNA sequencing of the rs2204985-containing region in Covid-19 patients was performed and correlated with the extent of lung involvement, the level of thymic function and the strength of immune response in those patients. Thymic function was estimated through the quantification of T cell receptor excision circles (TRECs) and peripheral T cells phenotyping. Patient’s SARS-CoV-2 specific immune response was assessed by quantification of anti-SARS-CoV-2 T-cell responses and plasma containing SARS-CoV-2 neutralizing antibodies.

**Results:** The GG genotype at the rs2204985 locus was associated with less severe lung involvement: only 54.5% of GG patients showed a CT-scan score of 3 or higher, compared to 83.3% and 100% in GA and AA patients, respectively (logistic regression model of AA + GA to GG, odds ratio = 0.16, 95% confidence interval: [0.03–0.8], p = 0.032; Figure 1A). Despite a comparable age distribution according to the genotype, the GG patients displayed a higher median sj/βTREC ratio compared to the combined AA + GA group (p = 0.042; Figure 1B) suggesting an increased thymic production of new lymphocytes. The GG genotype, independently of age and sex, was also associated with higher counts of blood naïve T lymphocytes including recent thymic emigrants, higher frequency of neutralizing antibodies during acute infection and with stronger anti-SARS-CoV-2 helper and cytotoxic T-cell responses six month after recovery.

**Conclusion:** We report the first evidence that rs2204985 polymorphism can impact the clinical and immunological responses to severe viral infections, by affecting both the baseline production of T-cells by the thymus and its enhancement during the acute phase of the infection. Patients with the GG genotype, who display a greater basal thymic activity and a larger naïve T-cell pool, were able to mount stronger and more efficient T-cell responses against SARS-CoV-2. The analysis of the rs2204985 sequence variants might be included among the predictive tests in critically ill COVID-19 patients and more generally in severe infections and other clinical situations where stimulated thymopoïesis has mechanistic and/or prognostic importance.

**Reference 1**

Cuvelier, P. et al. Protective reactive thymus hyperplasia in COVID-19 acute respiratory distress syndrome. Crit Care 25, 4 (2021).

**Reference 2**

Clave, E. et al. Human thymopoiesis is influenced by a common genetic variant within the TCRA-TCRD locus. Science Translational Medicine 10, eaao2966 (2018).

**Compliance with ethics regulations:** Yes in clinical research.

**Figure 1 (abstract FC-042):**
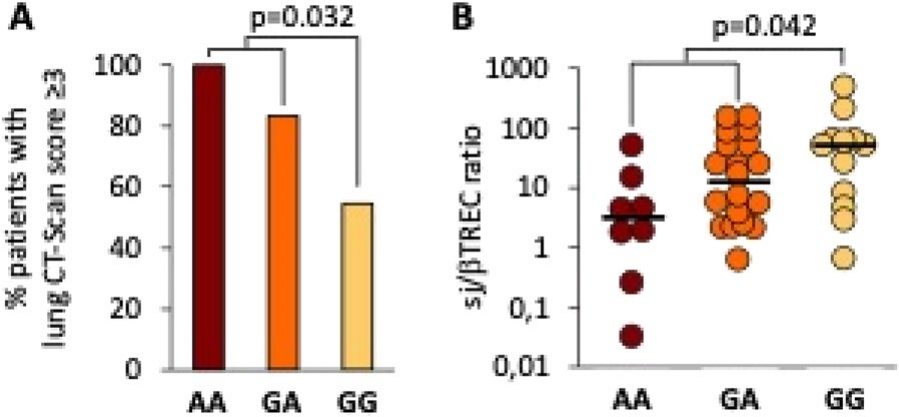
Patients with GG genotype demonstrate less severe disease and higher thymic output. COVID-19 patients were classified according to their genotype at the rs2204985 (n = 8 AA, n = 20 GA and n = 12 GG) locus and plotted against disease severity (A) and thymic function (sj/bTREC ratio; B)

## FC-043 Solid Organ Transplant Recipients in the ICU: etiologies and 6 month outcome in a retrospective monocenter study (2014–2022)

### Laurent Camous^1^

#### ^1^CHU de Guadeloupe, Les Abymes, France

##### **Correspondence:** Laurent Camous (laurent.camous@chu-guadeloupe.fr)

*Annals of Intensive Care *2013, **13(Suppl 1)**:FC-043

**Rationale:** Describe epidemiology, outcome of and death risk factors of Solid Organ Transplant (SOT) patients admitted in the intensive care unit (ICU) of the Guadeloupe University Hospital. Six months graft function and post ICU immunologic and infectious events were also recorded.

**Patients and methods/materials and methods:** All SOT patients admitted in the ICU between January 2014 to December 2022 were included in the study. Three groups were defined according to time from organ transplantation (< 1 year, 1–5 years and > 5 years). Using univariate and multi-variate analysis, we assessed risk factors associated with death.

**Results:** Among the 199 patients admitted to the ICU on the study period, 57 (29%) were assigned to the early group (< 1 year), 80 (40%) to the middle group (1–5 year), and 62 (31%) to the late group (> 5 years from transplant). Overall median age and SAPS II were 60 years and 42 respectively. In the early group, life threatening events leading to ICU admission were mostly linked to transplantation procedure related complications (including immunosuppressors toxicity) differing statistically from the two other groups. In the middle and late group, infectious diseases were the leading cause for ICU admission (65% and 56% respectively). Severe cardiovascular events were mostly seen in the early and middle group. Overall mortality rate at 3 months after ICU admission was 30%. Risk factors for death in multivariate analysis were shock, mechanical ventilation during ICU course and SARS Cov2 infection as cause for ICU admission. Original nephropathy and previous immunosuppression before transplantation were not associated with a worse outcome. Immunosuppressors discontinuation during ICU course was not associated with graft rejection at 6 months.

**Conclusion:** ICU admission in solid organ transplant recipients is frequent, with several etiologies. Pattern of ICU admission is different, according to time to transplantation time. In the early period (< 1 year), transplantation procedures related etiologies are predominant and after 1-year, infectious events are predominant. In multivariate analysis, mechanical ventilation, shock and SARS COV2 infection were independently predictive of death. The burden of SARS COV 2 infection in SOT patients was major as the pandemic period spanned less than 1/3 of the study period.

**Compliance with ethics regulations:** Yes in clinical research.

## FC-044 Hemorrhagic complications secondary to anticoagulation treatment among COVID-19 ARDS patients

### Iyed Maatouk^1^, Oussema Saadaoui^1^, Khouloud Hafi^1^, Safa Fathallah^1^, Zeineb Hammouda^1^, Abir Chihaoui^1^, Rania Lahouimel^1^, Wiem Nouira^1^, Manel Lahmar^1^, Saoussen Benabdallah^1^, Fahmi Dachraoui^1^, Fekri Abroug^1^, Lamia Ouanes Besbes^1^

#### ^1^University hospital Fattouma Bourguiba of Monastir, Monastir, Tunisie

##### **Correspondence:** Iyed Matouk (maatouk.yed@gmail.com)

*Annals of Intensive Care *2013, **13(Suppl 1)**:FC-044

**Rationale:** Because of the frequent association between COVID-19 infection and thromboembolic events in patients hospitalized in the ICU, anticoagulation was often prescribed. Nevertheless, anticoagulation treatment can lead to serious complications such as bleeding. We aimed to describe the characteristics of patients hospitalized with COVID-19 pneumonia who developed a hemorrhagic complication.

**Patients and methods/materials and methods:** We conducted a retrospective study between 2020 and 2022 in the Intensive Care Unit (ICU) of a Tunisian University Hospital. We included COVID-19 patients aged more than 18 years admitted to ICU for severe ARDS due to SARS-COV2.

**Results:** During the study period, 433 patients were admitted to our ICU for COVID-19 ARDS. The majority were male (66.7%). Mean age was 59.6 ± 13 years. Obesity was found among 38.3% of patients. Median SAPSII was 29 (Interquartile range (IQR): 23.7- 35). Median APACHE was 6 (IQR: 9–12). The mean SOFA score was 3 (IIQ: 3–4). Median length of stay was 10 (IQR: 6–14) days. Invasive Mechanical ventilation was required in 43.3% of patients. In total, 20 patients presented hemorrhagic complications secondary to anticoagulation (5%). Among them, 60% were male. Their mean age was 65.6 ± 11 years. Regarding COVID-19 disease, 60% of the patients had moderate to severe lung injury. All patients received anticoagulation, of which 90% was preventive. Low molecular weight heparin was prescribed in almost all patients (80%). Out of the 20 cases, 9 patients had a digestive hemorrhage (45%), one patient had a muscle hematoma (5%) and one patient had a retroperitoneal hematoma (5%). Other patients had minor complications (epistaxis, gingivorrhage or ecchymosis). Only one patient presented a hemorrhagic shock and 8 patients presented a deglobulation without hemodynamic repercussions. No patient had interventional treatment, and 40% required transfusion. Eighty percent of patients died secondary to septic shock or refractory hypoxemia.

**Conclusion:** The prevalence of hemorrhagic complications among COVID-19 patients was low in our study. They are dominated by severe digestive hemorrhage and epistaxis. Other studies should be conducted to assess the risk–benefit ratio of this treatment among COVID-19 patients known by their state of hypercoaguability.

**Compliance with ethics regulations:** Yes in clinical research.

## FC-045 Balance between neutrophil extracellular trap formation and DNase activity in COVID-19 patients

### Renaud Prével^3,4^, Geoffrey Garcia^6^, Sylvie Labrouche-Colomer^6^, Alexandre Duvignaud^1,5^, Etienne Clequin^2,3^, Charles Dussiau^6,7^, David-Alexandre Tregouet^1^, Denis Malvy^1,5^, Julien Goret^2^, Maria Mamani-Matsuda^2^, Antoine Dewitte^2,3^, Chloé James^6,7^

#### ^1^INSERM UMR 1219, Bordeaux Population Health Center, Bordeaux University, Bordeaux, France; ^2^ImmunoConcEpT CNRS UMR 5164, ERL Inserm U1303, Bordeaux University, Bordeaux, France; ^3^Medical intensive care unit, Bordeaux University Hospital, Bordeaux, France; ^4^Centre de Recherche Cardio-Thoracique de Bordeaux, Inserm UMR 1045, Bordeaux University, Bordeaux, France; ^5^Departement of Infectious and tropical Diseases, Bordeaux University Hospital, Bordeaux, France; ^6^Inserm, Umr1034, Biology of Cardiovascular disease, Bordeaux University, Bordeaux, France; ^7^Laboratory of Hematology, Bordeaux University Hospital, Pessac, France.

##### **Correspondence:** Geoffrey Garcia (geoffrey.garcia@inserm.fr).

*Annals of Intensive Care *2013, **13(Suppl 1)**:FC-045

**Rationale:** Immunothrombosis is increased in severe COVID-19, and potentially involved in its pathogenesis but the mechanisms explaining this deregulation are currently not understood. We hypothesized that decreased DNase activity was associated with increased NETosis and clinical worsening. We aimed to compare the balance between NETs and DNase activity according to COVID-19 severity and investigate the mechanisms responsible for the imbalance between NETosis and DNase activity.

**Patients and methods/materials and methods:** We studied 93 non-severe (outpatients), 27 severe (hospitalized) and 37 critical COVID-19 patients from COLCOV19-BX and COVERAGE studies. We quantified NET markers (MPO-DNA, H3Cit, H3cit-DNA), total DNase activity and the levels of DNase 1 and DNase1L3 proteins in plasmas from the 157 patients. In the 54 patients that were hospitalized, we sequenced DNase1 and DNase1L3 genes and used flow cytometry to quantify the numbers of plasmacytoid dendritic cells (pDC) and dendritic cells (DC) among peripheral blood mononuclear cells. Lastly, we analyzed scRNAseq public data from COVID-19 patients.

**Results:** Plasma NET markers were higher in the most severe patients while DNase activity was lower. Critical patients showed decreased level of DNase 1 & 1L3 proteins compared to severe patients. We did not find any recurrent mutations in DNase1 and DNase1L3 that was associated with decreased amount of DNase1 or DNase1L3. We observed a quantitative defect of DC and pDCs in critical patients. Besides, analysis of public scRNaseq data revealed an inverse correlation between DNase1L3 RNA expression in pDC and disease severity.

**Conclusion:** Altogether, our study shows that severe and critical COVID-19 is associated with an imbalance between NETs and DNase activity, with decreased amount of DNase 1 and DNase1L3. We speculate that the defect in DNase1L3 may be due to a defect in DCs and pDCs. Early identification of patients with NETosis imbalance could allow administration of targeted therapies, such as DNase, to prevent clinical worsening.

**Compliance with ethics regulations:** Yes in clinical research.

## FC-046 Relationship between vaccination status, clinical manifestations and outcome in critical COVID-19 patients: A retrospective study

### Asma Mehdi^1^, Salma Ghalloussi^1^, Hounaida Galai^1^, Ahlem Trifi^1^, Asma Ouhibi^1^, Eya Seghir^1^, Sami Abdellatif^1^

#### ^1^Hôpital La Rabta, Tunis, Tunisie

##### **Correspondence:** Salma Ghalloussi (salmaghalloussi93@gmail.com)

*Annals of Intensive Care *2013, **13(Suppl 1)**:FC-046

**Rationale:** The resurgence of COVID-19 cases since June 2021, referred to as the fourth COVID-19 wave, has led to the approval and administration of repeated vaccine doses. Our study aims to investigate the association between vaccination status and clinical presentation, biological features, amount of oxygen support and outcome among patients admitted in ICU for critical SARS-COV-2 infection.

**Patients and methods/materials and methods:** A retrospective, observational study conducted from January 2021 to 30 August 2022 carried out in the medical ICU of the University Hospital Center of la Rabta-Tunisia. Were included all adult patients admitted for critical SARS-CoV-2 infection after generalization of vaccination. Patients were divided into four groups according to their vaccination status: Unvaccinated, receiving one dose, two doses and three doses or more. Data related to clinical and biological features, oxygen support needed and outcome were collected and compared according to vaccination status.

**Results:** 237 patients were included. Between groups, Age > 60 years (p = 0.007) and co- morbidities including hypertension (p = 0.001), immunodeficient status (p = 0.037), chronic heart failure (p = 0.034), chronic respiratory failure (p = 0.011) and chronic kidney disease (p = 0.013) were significantly more reported among vaccinated patients with 3 doses or more. PaO_2_/FiO_2_ ratio was significantly correlated to the dose received (p < 10^–3^). Severe to critical CT scan lesions (p = 0.002), Severe ARDS (p < 10^–3^), mechanical ventilation (p = 0.003) and mortality (p = 0.04) were lower among vaccinated patients with 3 doses or more.

**Conclusion:** Vaccination was found to be a protective factor, since vaccinated patients presented moderate symptoms, less biological disorders, CT scan extent, critical forms and need of mechanical ventilation. This protective effect was, for some parameters, more found among patients vaccinated with 3 doses or more.

**Compliance with ethics regulations:** Yes in clinical research.

**Table 1 (abstract FC-046):**
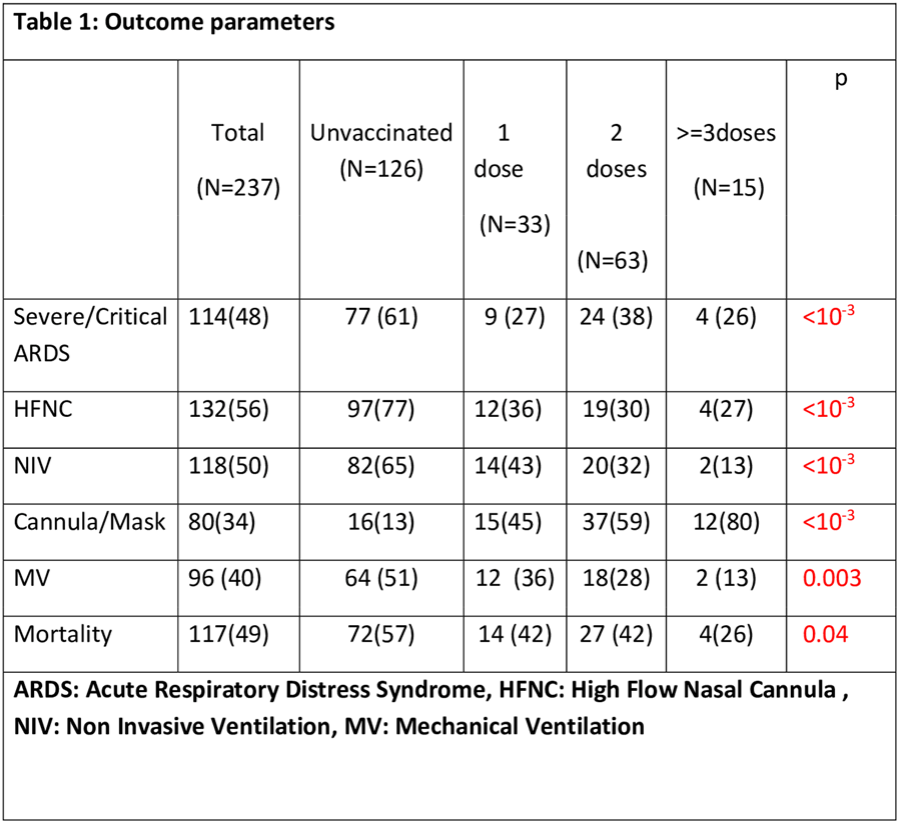
Outcomes parameters

## FC-047 Microbiological profiles of severe pneumopathies during the COVID-19 pandemic: interest of multiplex PCR screening

### Hedia Ben Ahmed ^1^, Manel Lahmar^1^, Hamza Ben Hassine ^1^, Abir Chihaoui^1^, Maha Hamdi^1^, Wiem Nouira ^1^, Zeineb Hammouda^1^, Saousen Ben Abdallaha^1^, Selma Mhalla^1^, Fahmi Dchraoui^1^, Fekri Abroug^1^, Lamia Ouanes Besbes^1^

#### ^1^Hôpital Fattouma Bourguiba, Monastir, Tunisie

##### **Correspondence:** Manel Lahmar (firassmal4@gmail.com)

*Annals of Intensive Care *2013, **13(Suppl 1)**:FC-047

**Rationale:** The clinical presentation during respiratory infection with SARS-CoV-2 is similar to the infection with other respiratory pathogens; however, the management and prognosis are different. The search for these agents has been reduced during the covid pandemic but does not prevent their co-existence with SARS-CoV-2. The objectives of this study were to identify the bacteria and viruses responsible for respiratory infections using the multiplex PCR diagnostic test and characterize clinical profiles during the COVID-19 pandemic.

**Patients and methods/materials and methods:** An observational study was performed between January 2020 and September 2022, in the Intensive Care Unit (Monastir, Tunisia), including patients admitted with hypoxemic respiratory failure. Respiratory samples were analyzed using a qualitative multiplex PCR technique (RespiFinder). Diagnosis of Covid-19 infection was based on COVID PCR test or chest CT scan.

**Results:** During the study period, 352 COVID-19 patients with hypoxemic respiratory failure were admitted to our ICU. PCR multiplex was made in 60 patients given the unavailability of this technique during the COVID-19 pandemic. The median age of these patients was 50 years [IQR: 41–70] and the median SAPS II score was 31 [IQR: 20–39]. The main clinical signs presented by the patients were dyspnea and cough. The multiplex PCR was positive in 45% of cases. When the PCR panel was performed, 86% of patients were receiving antibiotic treatment. All germs identified were viruses and no bacterial infection was detected in our study: 14 cases of Influenza A (H1N1), 3 cases of VRS B, 3 cases of coronavirus 229E, 1 case of coronavirus HKU1, 3 cases of coinfection Rhinovirus/Enterovirus, 1 case of coinfection coronavirus NL63/HKU1, 1 case of coinfection coronavirus 229E/VRS B and 1 case of coinfection Influenza B/Adenovirus. The diagnosis of SARS-Cov2 infection was observed in 28.3% of cases, 23.5% of them were coinfections (4 cases). During hospitalization, 69% of these patients required invasive mechanical ventilation.

**Conclusion:** Pathogenic microorganisms with respiratory tropism still exist during.

The COVID-19 pandemic and may be responsible for severe respiratory infections. Their research must be performed simultaneously with the SARS-CoV-2 diagnostic test to adapt the therapeutic management of patients.

**Compliance with ethics regulations:** Yes in clinical research.

## FC-048 Oropharyngeal bacterial microbiota analysis at hospital admission: a comparison of COVID-19 and cerebral vascular disease patients

### Delphine Bernard^1^, Bernard Taminiaux^2^, Mayssam Medlej^1^, Michel Moutschen^1^, Benoit Misset^1^, Georges Daube^2^, Anne-Françoise Rousseau^1^

#### ^1^University Hospital of Liège, Liège, Belgique; ^2^Faculty of Veterinary Medicine, University of Liege, Liège, Belgique

##### **Correspondence:** Anne-Françoise Rousseau (afrousseau@chuliege.be)

*Annals of Intensive Care *2013, **13(Suppl 1)**:FC-048

**Rationale:** Alterations in oropharyngeal (OP) microbiota have been associated with viral infections, with potential bi-directional interactions. In published studies, the OP of COVID-19 patients was mostly compared to healthy patients, with inconsistent observations. The aim of this monocenter prospective study was to compare the OP bacterial microbiota profile in patients admitted to the hospital for COVID-19 and for stroke.

**Patients and methods/materials and methods:** Patients admitted in our tertiary hospital either for COVID-19 pneumonia diagnosed with a positive polymerase chain reaction (PCR) for SARS-CoV-2 in nasal swab or for an ischemic or hemorrhagic stroke (with negative SARS-CoV-2 PCR) were enrolled respectively during the second and third waves of the pandemic, and between September and December 2021. COVID-19 patients were admitted to general wards or directly to the intensive care unit (ICU). Exclusion criteria were antibiotic exposure during the past 15 days, coagulopathy, endotracheal intubation and pregnancy. OP swabs were taken from the hard palate within the first 24 h after admission and used for a 16S rRNA gene-targeted metagenomic analysis. Demographic data, medical history, and in-hospital deaths were recorded.

**Results:** A total of 135 swabs were taken, and 83 were analyzed: 55 in the COVID group (65 [64–69] years, 22 women), including 16 patients in an ICU subgroup, and 28 in the stroke group (72 [64–80] years, 11 women). The multivariate analysis showed that the reason for admission significantly influenced the beta diversity (p < 0.001). The beta diversity in the two subgroups of COVID patients was similar. The beta diversity was not influenced by age, smoking habits, or proton pump inhibitor treatment and was not associated with in-hospital mortality. Alpha diversity in COVID patients (population richness and population diversity) and the stroke group was not significantly different. At the genus level, 38 genera were identified with significant relative abundance differences between COVID and stroke groups. Among them, 34 taxa, including the genus *Veillonella* and family *Prevotellaceae* were significantly increased in COVID patients, while 4 taxa, including the genus *Streptococcus* were significantly depleted. The microbiota was dominated by taxa belonging to 4 phyla: Firmicutes (*Streptococcus, Veillonella, Gemella*), Bacteroidota (*Prevotella*, *Prophyromonas*), Fusobacteriota (*Fusobacterium, Leptotrichia*) and Proteobacteria (*Neisseria*).

**Conclusion:** In this study, a significant difference in OP bacterial microbiota was observed in COVID-19 patients, including more pro-inflammatory genera, compared to other newly admitted patients for a stroke. It can be assumed that this dysbiosis occurred earlier during viral infection. Moreover, this dysbiosis was not associated with respiratory failure severity.

**Compliance with ethics regulations:** Yes in clinical research.

## FC-049 Lung microbiota compositions differ between influenza, COVID-19 and bacteria-related acute respiratory distress syndromes

### Sébastien Imbert^1^, Raphaël Enaud^1^, Mathilde Revers^1^, Arthur Orieux^1^, Florian Lussac-Sorton^1^, Adrian Camino^1^, Alexandre Massri^2^, Laurent Villeneuve^2^, Cédric Carrié^1^, Laurent Petit^1^, Alexandre Boyer^1^, Patrick Berger^1^, Didier Gruson^1^, Laurence Delhaes^1^, Renaud Prével^1^

#### ^1^CHU Bordeaux, Bordeaux, France; ^2^CH François Mitterrand, Pau, France

##### **Correspondence:** Renaud Prével (renaud.prevel@hotmail.fr)

*Annals of Intensive Care *2013, **13(Suppl 1)**:FC-049

**Rationale:** Acute respiratory distress syndrome (ARDS) is still responsible for about 400,000 deaths per year worldwide, requiring new areas of research to improve patients’ care. The lung microbiota is involved in numerous chronic inflammatory pulmonary diseases and recent data suggest that it could play a role in ARDS as well. Nevertheless, the only improvements made despite five decades of intensive research consist in limiting the adverse effects of supportive care. Failure in the development of new therapeutics may be partly explained by the fact that ARDS is a highly heterogeneous syndrome involving different types of aetiologies and extremely clinically and biologically diverse patients. The aim of this study is to compare lung microbiota (both bacterial and fungal kingdoms) composition between specific ARDS subphenotypes, i.e., pulmonary ARDS due to influenza, SARS-CoV-2, or bacterial infection.

**Patients and methods/materials and methods:** Consecutive ARDS patients according to Berlin’s classification requiring invasive mechanical ventilation were screened in 2 ICUs. Influenza and SARS-CoV-2 infections were confirmed by PCR, and bacterial infection when > 10^5^ CFU/mL bacteria were isolated on endotracheal aspirate. Endotracheal aspirate was collected at admission. After DNA extraction, V3-V4 and ITS2 regions were amplified by PCR. Deep sequencing was performed on MiSeq sequencer (Illumina®); data were analysed using DADA2 pipeline.

**Results:** Twenty-four COVID-19-, 18 consecutive influenza-, and 11 bacteria-related ARDS patients were included. Bacteria-related ARDS patients had lower lung bacteriobiota α-diversity compared with COVID-19-related ARDS patients (p = 0.42 and p = 0.029 for Shannon and Simpson indices, respectively). Influenza-related ARDS patients had higher lung mycobiota α-diversity compared with COVID-19-related ARDS ones (p = 0.01 and 0.05 for Shannon and Simpson indices, respectively). Lung bacteriobiota composition was dissimilar between COVID-19-related and both influenza- and bacteria-related ARDS patients (β-diversity, Permanova p = 0.05 for each). Lung mycobiota composition was dissimilar between influenza-related and both COVID-19- and bacteria-related ARDS patients (β-diversity, Permanova p = 0.01 and p = 0.04, respectively).

**Conclusion:** Compositions of both lung bacteriobiota and lung mycobiota are different between influenza-, COVID-19- and bacteria-related ARDS. Future studies investigating the role of lung microbiota in ARDS pathophysiology should take aetiology into account.

**Compliance with ethics regulations:** Yes in clinical research.

## FC-050 Influence of selective digestive decontamination on survival and prevention of ICU acquired infections in Sars-Cov-2 infected patients with mechanical ventilation for acute respiratory failure

### Michael Ejzenberg^1^, Paul-Henri Wicky^1^, Juliette Patrier^1^, Etienne De Montmollin^1^, Romain Sonneville ^1^, Lila Bouadma^1^, Jean-François Timsit^1^

#### ^1^CHU Bichat, Paris, France

##### **Correspondence:** Michael Ejzenberg (ejzenberg.michael@gmail.com)

*Annals of Intensive Care *2013, **13(Suppl 1)**:FC-050

**Rationale:** During the first months of the COVID pandemic, we observed a high rate of ventilator-associated pneumonia (VAP) and nosocomial bacteremia. In accordance with formal expert recommendations on VAP, we decided to implement selective digestive decontamination (SDD) for all intubated patients with SARS-CoV-2 infection. We evaluated the effectiveness of this measure on day-60 mortality, rates of acquired bloodstream infection (BSI) and VAP.

**Patients and methods/materials and methods:** We performed a retrospective, monocentric cohort study based on the analysis of data collected in the OUTCOMEREA database. In addition to standard hygiene and nosocomial infection prevention measures, the SDD regimen consisted in enteral administration 4 times daily of colimycin 135 mg, gentamicin 100 mg, and amphotericin B 500 mg, without intravenous antibiotic prophylaxis. ICU mortality, rates of bacteremia and VAP were assessed over 2 periods, before and during SDD administration. Multivariate analyses used Cox models for survival and the Fine and Gray model for the risk of nosocomial infection.

**Results:** 104 patients were included from may 2020 to may 2021: 59 patients in the cohort without SDD, and 45 patients in the SDD cohort (Table). Nearly ¾ of the patients in the 2 groups were treated with IV antibiotic therapy within 48 h of intubation. After adjustment on case-mix and confounders, Day-60 mortality was not different between periods (adj HR:0.61 95% CI 0.29–1.27), p = 0.19). BSI rates were similar between groups (Table), without significant difference after controlling for confounders (sHR 1.29, [0.65—2.52], p = 0.47). VAP rates were also similar after adjustment (sHR 1.74, [0.94—3.2]) and the VAP incidence densities were also similar (11.69/1000 vs 11.68/1000 ventilator-days, without and with SDD respectively). Except for 2 bezoars, SDD was not associated with adverse events.

**Conclusion:** In a before-after study, the use of systematic SDD in mechanically ventilated patients with SARS-CoV-2 pneumonia was not associated with a significant reduction in the rate of VAP and ICU-BSI and had no beneficial effect on day-60 survival.

**Compliance with ethics regulations:** Yes in clinical research.

**Table 1 (abstract FC-050):**
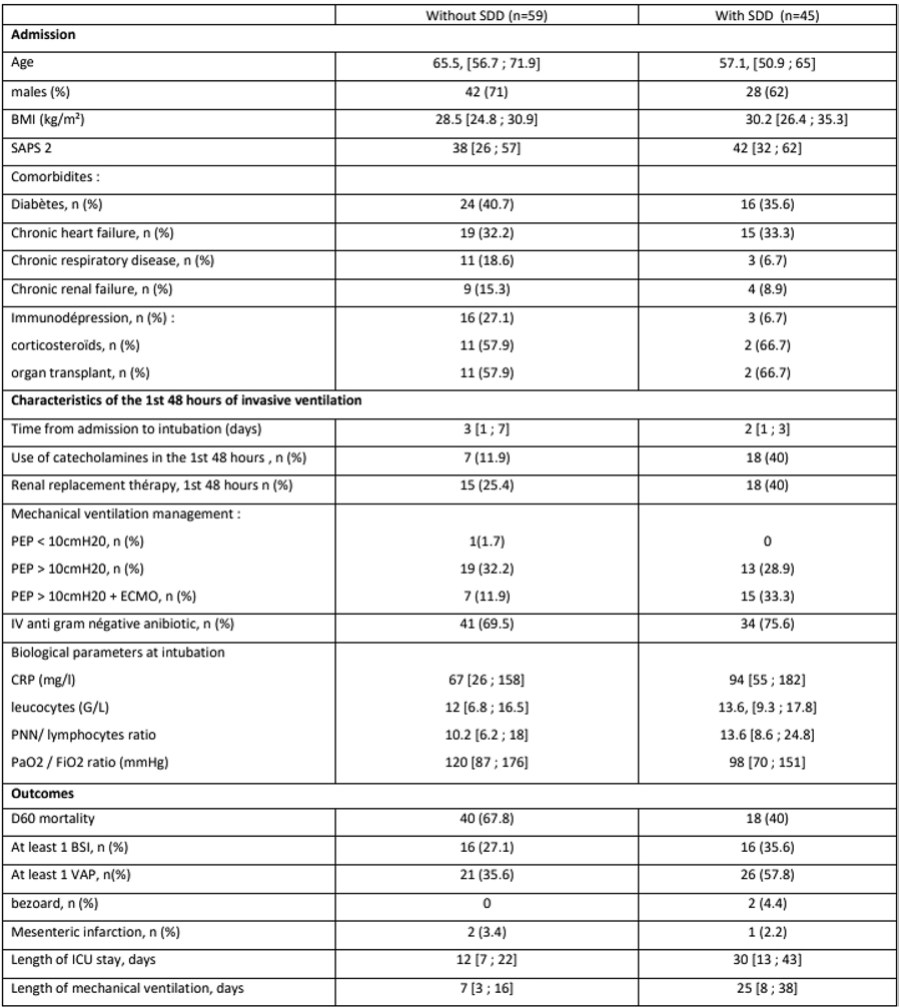
Patients characteristics at admission, intubation and outcomes

## FC-051 Relationship between SARS-CoV-2 infection and bacteremia involving multidrug-resistant bacteria in intensive care unit

### Antoine Piantoni^1^, Marion Houard^1^, Gaetan Piga^1^, Ghadi Zebian^1^, Sarah Ruffier Des Aimes^1^, Berenice Holik^1^, Saadalla Nseir^1^

#### ^1^CHU Lille, Critical Care center, Lille, France

##### **Correspondence:** Antoine Piantoni (antoine.piantoni@hotmail.fr)

*Annals of Intensive Care *2013, **13(Suppl 1)**:FC-051

**Rationale:** Bloodstream infection (BSI) is a common ICU-acquired infection. A growing proportion of them is caused by multidrug-resistant (MDR) bacteria. Recently, the SARS-CoV-2 pandemic led to an increased rate of critical illness and seems to be associated with a high rate of secondary infections, such as pneumonia and BSI. However, there is a lack of data on the influence of SARS-CoV-2 infections on the incidence of BSI-related MDR. In this study, our aim was to evaluate the influence of SARS-CoV-2 infection on MDR BSI occurrence. The secondary objectives were to study the impact of COVID on outcomes, including mortality and length of ICU stay, in MDR BSI patients.

**Patients and methods/materials and methods:** This retrospective study was conducted in a single mixed intensive care unit (ICU) from January 1 to December 31, 2020. All adult patients admitted to our ICU for more than 48 h were included. Collected data included COVID-19 status, MDR BSI, demographic characteristics, main admission diagnosis, comorbidities, prior antibiotic exposure, invasive life support procedures, and immunomodulators. Univariate and multivariate analyses were conducted with a Cox regression analysis. Exposure to COVID-19 was considered as a time-dependent covariate in the Cox model.

**Results:** Among the 1327 patients included in the analysis, 502 had COVID-19. MDR BSI occurred in 36 (7%) COVID-19 patients and in 14 (1.6%) non-COVID-19 patients. SARS-CoV-2 infection was associated with a significantly higher risk of MDR BSI starting on the 15^th^ day of ICU hospitalization in univariate (HR 3.74 (1.43–9.79)) and multivariate analysis (HR 4.09 (1.50–11.1)), adjusting for age, SAPS II, immunosuppression, prior hospitalization, prior antibiotic treatment, MDR colonization, and dialysis. No significant impact of COVID-19 was found on mortality (HR 2.11 (1.13–3.93) vs 1.36 (0.50–3.70), interaction p-value 0.55) or length of ICU stay (HR 1.06 (0.73–1.52) vs 0.76 (0.43–1.32), interaction p-value 0.31) in COVID-19 and non-COVID-19 patients, respectively.

**Conclusion:** In this study, COVID-19 was independently associated with a higher risk of MDR BSI starting on the 15th day of ICU hospitalization.

**Compliance with ethics regulations:** Yes in clinical research.

## FC-052 Impact of COVID-19 on ICU-acquired multidrug-resistant bacteria: a multicenter before-after study

### Louis Kreitmann^1^, Jermoumi Sonia^1^, Julien Labreuche^2^, Nseir Saad^1,3^

#### ^1^Médecine Intensive Réanimation, CHU de Lille, Lille, France; ^2^CHU Lille, Department of Biostatistics, Lille, France; ^3^Inserm U1285, Université de Lille, CNRS, UMR 8576-UGSF, Lille, France

##### **Correspondence:** Louis Kreitmann (louis.kreitmann@gmail.com)

*Annals of Intensive Care *2013, **13(Suppl 1)**:FC-052

**Rationale:** Patients presenting the most severe form of severe acute respiratory syndrome coronavirus 2 (SARS-CoV-2) disease 2019 (COVID-19) pneumonia have a prolonged intensive care unit (ICU) stay and are exposed to broad-spectrum antibiotics, but the impact of COVID-19 on antimicrobial resistance is unknown. The main objective of this study was to investigate the association of COVID-19 with the cumulative incidence of ICU-acquired colonization and infection related to multidrug-resistant (MDR) bacteria (ICU-MDR-col and ICU-MDR-inf, respectively).

**Patients and methods/materials and methods:** Observational prospective before-after study in 7 ICUs in France. All consecutive patients with an ICU stay longer than 48 h and a confirmed SARS-CoV-2 infection were included prospectively and followed for 28 days. Patients underwent systematic screening for colonization with MDR bacteria upon admission and every week subsequently. COVID-19 patients were compared to a recent prospective cohort of control patients from the same ICUs [1].

**Results:** During the first and second waves of the pandemic, 367 COVID-19 patients were included and compared to 680 controls. After adjustment for prespecified baseline confounders, the cumulative incidence of a composite outcome including ICU-MDR-col and/or ICU-MDR-inf was not significantly different between groups (adjusted sub-hazard ratio [sHR] 1.39, 95% confidence interval [CI] 0.91–2.09). When considering both outcomes separately, COVID-19 patients had a higher incidence of ICU-MDR-inf than controls (adjusted sHR 2.50, 95%CI 1.90–3.28), but the incidence of ICU-MDR-col was not significantly different between groups (adjusted sHR 1.27, 95%CI 0.85–1.88).

**Conclusion:** COVID-19 patients had an increased incidence of ICU-MDR-inf compared to controls, but the difference was not significant when considering a composite outcome including ICU-MDR-col and/or ICU-MDR-inf. If confirmed, our findings could have important practical implications regarding the choice of empiric antibacterial regimens in COVID-19 patients.

**Reference 1**

1. Kreitmann L, Vasseur M, Jermoumi S, Perche J, Richard J-C, Wallet F, et al. Relationship between immunosuppression and intensive care unit-acquired colonization and infection related to multidrug-resistant bacteria: a prospective multicenter cohort stu.

**Compliance with ethics regulations:** Yes in clinical research.

## FC-053 Recurrences of ventilator-associated pneumonia in COVID-19 acute respiratory distress syndrome requiring veno-venous extracorporeal membrane oxygenation

### Elena Collado Lledó^1^, Charles-Edouard Luyt^1^, Alain Combes^1^, Matthieu Schmidt^1^, Juliette Chommeloux^1^, Marc Pineton De Chambrun^1^, Guillaume Hekimian^1^, Charles Juvin^1^, Lucie Le Fèvre^1^

#### ^1^Assistance Publique-Hôpitaux de Paris (APHP), Groupe Hospitalier Pitié-Salpêtrière, Paris, France

##### **Correspondence:** Lucie Le Fevre (lucie.lefevre@aphp.fr)

*Annals of Intensive Care *2013, **13(Suppl 1)**:FC-053

**Rationale:** As compared to other etiologies of acute respiratory distress syndrome (ARDS), COVID-19 is associated with a higher incidence of ventilator-associated pneumonia (VAP) and a higher incidence of VAP recurrences. We aimed to describe successive VAP episodes and factors associated with VAP recurrence in patients with COVID-19 ARDS requiring veno-venous extracorporeal membrane oxygenation (VV-ECMO) support.

**Patients and methods/materials and methods:** Consecutive adult patients with COVID-19 ARDS requiring VV-ECMO support admitted to our ICU between March 2020 and January 2022 were included. Microbiological data and antimicrobial regimen were collected for each VAP episode. All VAP episodes, when clinically suspected, were bacteriologically confirmed (bronchoalveolar lavage fluid yielding one or more pathogens growing ≥ 10^4^ UFC/ml). Each VAP recurrence was categorized as persistent infection (same pathogen growing at a significant concentration while receiving antimicrobial treatment for a previous VAP), relapse (same pathogen growing at a significant concentration and occurring at least 48 h after treatment cessation of a previous VAP), or superinfection (new pathogen growing at a significant concentration).

**Results:** Of the 252 patients included during the study period, 226 (89.7%) developed a first VAP (VAP1), of which 182 (80.5%) had at least one VAP recurrence. Enterobacteriaceae were the most frequent pathogens identified at VAP1 (50.4%), followed by non-fermenting gram-negative bacilli (NF-GNB) (36.3%). At first recurrence, Enterobacteriaceae and NF-GNB were identified in similar numbers of patients (60.4% and 64.8%, respectively). Sixty-three percent of first recurrences were relapses (30.2%) or persistent infections (33%), demonstrating the difficulty of durably eradicating pathogens from the lungs. Of the 226 patients who experienced one VAP episode, 168 (74%) had an antimicrobial course of less than 7 days for the first VAP (median duration 6 days, IQR 5–7) and 58 (26%) had a prolonged course of 8 days or more (median duration 9 days, IQR 8–11). In a survival model comparing short and long treatment groups of VAP1 considering extubation and death as competing events, we found no significant difference in terms of VAP recurrence rate.

**Conclusion:** In COVID-19 ARDS requiring VV-ECMO support, rates of VAP relapses and persistent infections were extremely high, with Enterobacteriaceae and NF-GNB being predominant causative microorganisms. Antimicrobial treatment of a first VAP episode for 8 days or more did not significantly reduce VAP recurrence rate.

**Compliance with ethics regulations:** Yes in clinical research.

**Table 1 (abstract FC-053):**
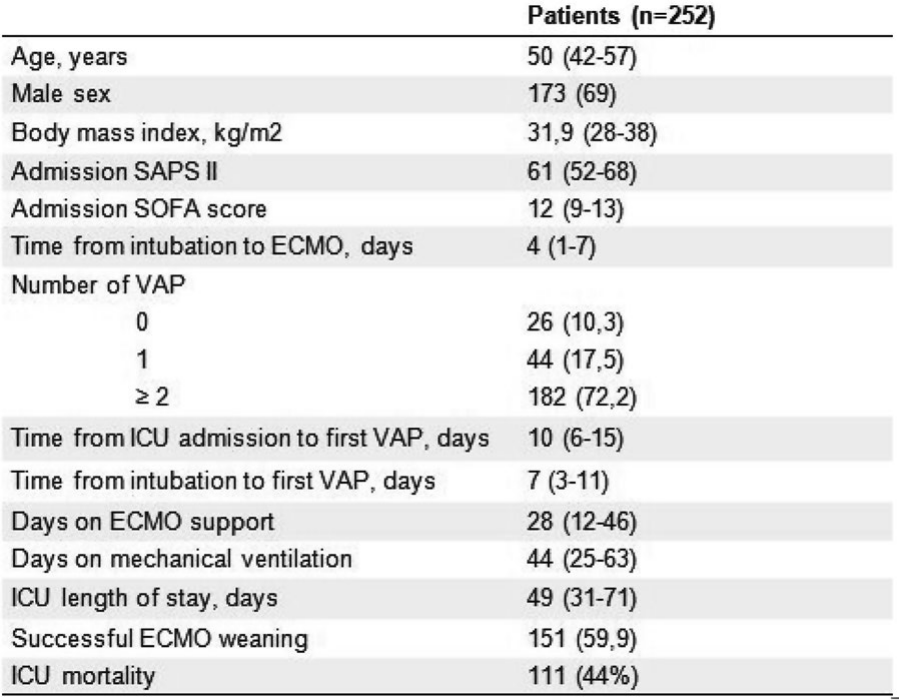
Population characteristics (data represented as median (interquartile range) or n (%))

## FC-054 Empiric antibiotic therapy in severe SARS-CoV-2 infection

### Yahyaoui Hajer^1^, Doghri Hamdi Hemdene ^1^, Borsali Falfoul Nebiha^1^

#### ^1^Hôpital Habib Thameur, Tunis, Tunisie

##### **Correspondence:** Yahyaoui Hajer (hajer.yahyaoui94@hotmail.com)

*Annals of Intensive Care *2013, **13(Suppl 1)**:FC-054

**Rationale:** The SARS-CoV-2 pandemic was characterized by increased antibiotic prescribing. This was initially justified by bacterial co-infection and superinfection risk and the antiviral and immunomodulator effects of macrolide antibiotics. Rapidly, this prescription became controversial due to a higher risk of nosocomial infection and multidrug-resistant bacterial selection. Our objective was to study empiric antibiotic therapy in COVID-19 critically ill patients, and to investigate its supporting arguments and evolving features.

**Patients and methods/materials and methods:** Descriptive, retrospective, and monocentric study including all patients admitted to an intensive care unit (ICU) between September 1, 2020, and June 30, 2022. All patient’s data including demographics, clinical, biological, radiological, bacteriological, therapeutic data and outcomes were taken from patient’s medical records.

**Results:** 450 patients infected with SARS-CoV-2 were included. The sex ratio was 1.14 and the mean age was 63 ± 15 years. Mean SAPSII, APACHEII and SOFA score were respectively 30 ± 39, 30 ± 39 and 8.6 ± 5. 83 patients had a severe form of acute respiratory distress syndrome (ARDS) on admission. The mean lymphocytes count, D-dimer, lactates, CRP and procalcitonin levels were 1295 ± 1721 cells/mm^3^, 3049 ± 10,090 ng/µL, 4 ± 14 mg/L, 137 ± 96 mg/L and 2.8 ± 10 ng/µL, respectively. 333 patients received empiric antibiotics in combination with parenteral corticosteroid therapy. Monotherapy was used in 6 cases (2%), dual therapy in 241 cases (73%) and tritherapy in 83 cases (25%). Betalactams were the most prescribed antibiotic (269 patients (81.5%)). Patients receiving antibiotics had a higher CRP ((151 vs 99), p = 0.046), procalcitonin ((3.6 vs 0.3), p < 10^–3^) and a lower count of lymphocytes ((1222 vs 1498) and p = 0.053)). Global mortality was 49% (n = 33). Empiric antibiotic prescription was justified by high procalcitonin levels in 58 cases (17.5%), by parenchymal opacities on chest CT scan in 18 cases (5.5%) and systematically in 254 cases (77%). No microbiologic evidence of a community bacterial superinfection was found. Empiric antibiotic therapy on admission was associated with nosocomial infection (p = 0.07), invasive mechanical ventilation (P = 0.001) and mortality (p < 10^–3^).

**Conclusion:** The COVID-19 pandemic is associated with an over-consumption of antibiotics, especially in the ICU. This systematic prescription is not justified and is associated with nosocomial infections and mortality.

**Compliance with ethics regulations:** Yes in clinical research.

## FC-055 Clinical description and outcome of overall Varicella zoster virus-related organ dysfunctions admitted in intensive care units: the VAZOREA cohort study

### Jolan Malherbe^1,2^, Pierre Godard^15^, Jean-Claude Lacherade^25^, Valentin Coirier^16^, Laurent Argaud^3^, Hervé Hyvernat^17^, Francis Schneider^4^, Julien Charpentier^5^, Florent Wallet^6,7^, Juliette Pocquet^26^, Gaëtan Plantefeve^27^, Jean-Pierre Quenot^20^, Pierre Bay^8,9^, Agathe Delbove^13^, Hugues Georges^22^, David Schnell^14^, Charlène Le Moal^18^, Matthieu Stanowski^28^, Corentin Muris^24^, Maud Jonas^10^, Bertrand Sauneuf^23^, Olivier Lesieur^11^, Amaury Lhermitte^21^, Laure Calvet^12^, Inès Gueguen^19^, Damien Du Cheyron^1,2^

#### ^1^CHU de Caen Normandie, Caen, France; ^2^Normandie Univ, UNICAEN, Caen, France; ^3^Service de Médecine Intensive-Réanimation, Hospices civils de Lyon, Hôpital Edouard Herriot, Université de Lyon, Université Claude Bernard Lyon 1, Faculté de Médecine Lyon-Est, Lyon, France; ^4^Médecine intensive Réanimation, Hôpital de Hautepierre, Hôpitaux Universitaires de Strasbourg et Unistra, Strasbourg, France; ^5^Service de Médecine Intensive-Réanimation, Hôpital Cochin, Assistance Publique-Hôpitaux de Paris, Centre-Université Paris Cité, Paris, France; ^6^Médecine intensive reanimation, CHU Lyon Sud, Pierre Benite, Lyon, France; ^7^RESHAPE Research on healthcare performance, U1290, Université Claude Bernard Lyon 1, Lyon, France; ^8^AP-HP Assistance Publique Hôpitaux de Paris, Hôpitaux universitaires Henri Mondor, DMU Médecine, Service de Médecine Intensive Réanimation, Créteil, France; ^9^UPEC Université Paris-Est Créteil, INSERM, Unité U955, Equipe 18, Créteil, France; ^10^Service Médecine Intensive Réanimation/USC, Centre hospitalier de Saint-Nazaire, Saint-Nazaire, France; ^11^Réanimation polyvalente, Centre Hospitalier Saint-Louis, La Rochelle, France; ^12^Service de Médecine Intensive et Réanimation, CHU de Clermont-Ferrand, Clermont-Ferrand, France; ^13^Service de réanimation polyvalente, CHBA Vannes, Vannes, France; ^14^Réanimation polyvalente et USC, CH Angoulême, Angoulême, France; ^15^Service de Médecine Intensive-Réanimation, CHU Bordeaux site Pellegrin, Bordeaux, France; ^16^Service de Médecine Intensive Réanimation, CHU de Rennes, Rennes, France; ^17^Service de Médecine Intensive Réanimation, CHU de Nice, 151 route Saint Antoine de Ginestière, 06200 Nice, Université Côte d’Azur (UCA), Nice, France; ^18^Service Réanimation/USC, Centre Hospitalier du Mans, Le Mans, France; ^19^Service de réanimation médicale, CHRU de Lille, Lille, France; ^20^Department of Intensive Care, Burgundy University Hospital, Dijon, France; ^21^Hôpital Universitaire Félix Guyon, Réanimation polyvalente, Allée des Topazes, 97400 Saint-Denis, La Réunion, Saint-Denis, France; ^22^Service de réanimation polyvalente, Centre hospitalier de Tourcoing, Tourcoing, France; ^23^Service de Réanimation polyvalente, Centre Hospitalier Public du Cotentin, Cherbourg En Cotentin, France; ^24^Université de Poitiers, CHU de Poitiers, Médecine intensive Réanimation, 2 rue de la miletrie, Poitiers, France; ^25^Médecine intensive-Réanimation, CH La Roche sur Yon, La Roche Sur Yon, France; ^26^Médecine intensive-Réanimation, CHR Orléans, Orléans, France; ^27^Service de Réanimation, CH Argenteuil, Argenteuil, France; ^28^Médecine intensive-Réanimation, CHRU de Nancy, Nancy, France

##### **Correspondence:** Jolan Malherbe (malherbe-j@chu-caen.fr)

*Annals of Intensive Care *2013, **13(Suppl 1)**:FC-055

**Rationale:** Varicella-zoster virus (VZV) is a ubiquitous herpesvirus known to cause infections in humans. The incidence of Herpes zoster is increasing, probably due to the aging population and the increasing number of immunocompromised patients. Therefore, an increase in life-threatening organ damage related to VZV can be expected. VZV is recognized as the second most common agent of encephalitis in France (the first one in immunocompromised patients) and is a well-known cause of viral pneumonia, but there are no data on VZV-related severe organ involvement. The main objective of this study was to describe the clinical features and outcome of all life-threatening VZV manifestations requiring admission to an intensive care unit (ICU).

**Patients and methods/materials and methods:** This retrospective cohort study was conducted in 25 French ICUs and included adult patients with life-threatening VZV-related events requiring ICU admission or occurring in ICU between 2010 and 2019.

**Results:** One-hundred and sixteen patients were included with a median age of 66 years and a median SOFA score of 6. Forty-three were primo-infected (37.1%), and 58 (50%) were immunocompromised. Encephalitis was the most prominent organ involvement (64 patients, 55.2%), followed by pneumonia (51, 44%) and hepatitis (11, 9.5%). Fifty-two patients (44.8%) received norepinephrine, and respiratory support was used in 96 patients (83.5%) including invasive mechanical ventilation in 70 of them (72.9%). Thirty-one patients (27%) received renal replacement therapy. In-hospital mortality was 36.2% and was significantly associated with 3 independent risk factors: immunosuppression, VZV reactivation and alcohol abuse in multivariate logistic regression. Hierarchical clustering on principal components revealed 4 phenotypically distinct clusters of patients (Figure): VZV-related pneumonia, mild-encephalitis, severe encephalitis predominantly due to VZV reactivation, and diffuse VZV reactivation occurring in profoundly immunocompromised patients. Phenotypes differed by medical history, clinical manifestations, and prognosis, with in-hospital mortality ranging from 5.6% to 70.4% (p < 0.001).

**Conclusion:** Overall, severe VZV manifestations still result in high mortality in the ICU. In-hospital mortality appears to be driven by immunosuppression status rather than by the predominance of organ involvement. Deciphering the different phenotypes may help clinicians assess prognosis.

**Compliance with ethics regulations:** Yes in clinical research.

**Figure 1 (abstract FC-055):**
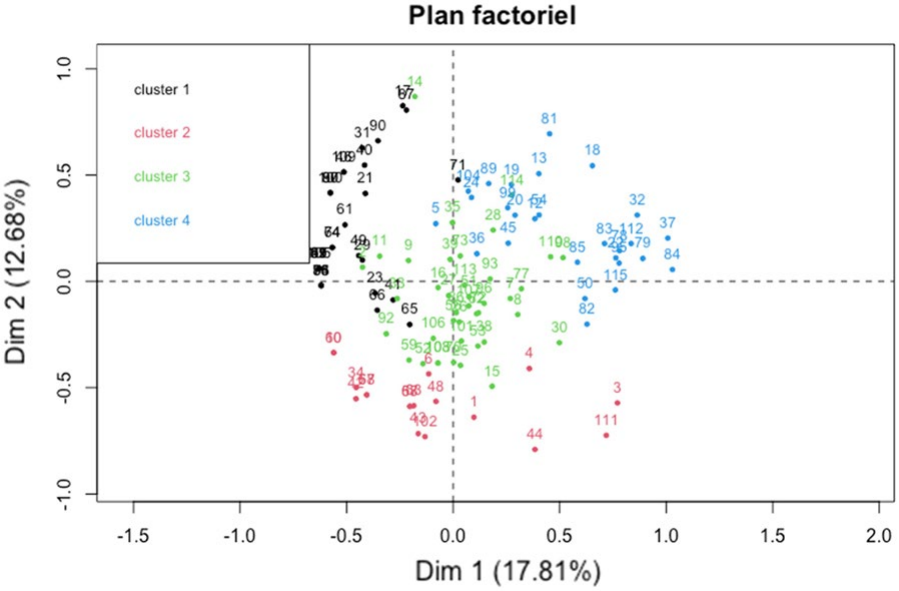
Factorial plan of hierarchical classification on principal components. These first two dimensions summarize 30.5% of the data variability

## FC-056 Long-term immunosuppressive treatment is not associated with increased mortality in patients admitted to the intensive care unit ICU for septic shock: the PACIFIC study

### Julien Vaidie^1^, Edwige Peju^4^, Louise-Marie Jandeaux^5^, Mathieu Lesouhaitier^6^, Jean-Claude Lacherade^7^, Antoine Guillon^8^, Xavier Wittebole^9^, Pierre Asfar^10^, Bruno Evrard^1,2^, Thomas Daix^1,2,3^, Philippe Vignon^1,2,3^, Bruno François^1,2,3^

#### ^1^Réanimation polyvalente, CHU Dupuytren, Limoges, France; ^2^Inserm CIC 1435, CHU Dupuytren, Limoges, France; ^3^Inserm UMR 1092, CHU Dupuytren, Limoges, France; ^4^Service de Médecine Intensive & Réanimation, Assistance Publique-Hôpitaux de Paris, Hôpital Cochin, Paris, France; ^5^Médecine intensive et Réanimation, Nouvel hôpital civil, CHRU de Strasbourg, Strasbourg, France; ^6^Réanimation médicale, CHU de Rennes, Rennes, France; ^7^Médecine Intensive-Réanimation, CHD Vendée, La Roche-Sur-Yon, France; ^8^Médecine Intensive-Réanimation, CHRU Bretonneau, Tours, France; ^9^Service des soins intensifs, Cliniques universitaire Saint-Luc, Bruxelles, France; ^10^Médecine intensive-réanimation et médecine hyperbare, CHU Angers, Angers, France

##### **Correspondence:** Julien Vaidie (julien.vaidie@chu-limoges.fr)

*Annals of Intensive Care *2013, **13(Suppl 1)**:FC-056

**Rationale:** With the exception of a few retrospective studies mainly including patients under chemotherapy, information regarding the impact of immunosuppressive therapy on the prognosis of patients admitted to the intensive care unit (ICU) for septic shock is scarce. Accordingly, the PACIFIC study aimed to assess if immunosuppressive therapy was associated with an increased mortality in patients admitted to the ICU for septic shock.

**Patients and methods/materials and methods:** This was a multicenter retrospective exposure/no exposure study. To better identify the effect of immunosuppressive treatment on outcome, we used an original selection approach to reduce limitations of a retrospective design. Patients in the “exposed” group were selected from the screen failure logs of seven recent randomised controlled trials (RCT) since they had not been enrolled because of their immunosuppressive treatment (e.g.: steroids, calcineurin, mTOR or TNF inhibitors, immunosuppressive monoclonal antibodies…). The “unexposed” patients were those included in the placebo arm of the same RCTs. We used a ratio of three "unexposed" patients to one "exposed" patient to increase power. Eight high enroller centers in septic shock RCTs participated in the study. A multivariate logistic regression model was used to estimate the risk of death.

**Results:** Among the 433 patients enrolled, 103 were included in the “exposed” group and 330 in the “unexposed” group. Reasons for immunosuppressive therapy included organ transplantation (n = 45 [44%]) or systemic disease (n = 58 [56%]). Baseline characteristics were similar between the two groups, with a mean SAPS 2 of 56 in the "exposed" group and 60 in the "unexposed" group (p = 0.14). ICU mortality rate was 24% in the “exposed” group and 25% in the “unexposed” group (p = 0.9). In univariate and multivariate analysis, immunosuppressive therapy was not associated with a higher ICU mortality (OR: 0.95; [95%CI: 0.56–1.58]: p = 0.86 and 1.21 [95%CI: 0.69–2.08]: p = 0.5, respectively) or 3-month mortality (OR: 1.13; [95%CI: 0.69–1.82]: p = 0.27 and OR: 1.37 [95%CI: 0.78–2.38]: p = 0.27, respectively). ICU length of stay was similar in the “exposed” and “non-exposed” groups (8 ± 4 vs. 9 ± 5 days: p = 0.2), as well as the duration of ventilation (4 ± 2 vs. 4 ± 2 days: p = 0.7) and the number of vasopressor-free days within 30 days (27 ± 2 days vs 26 ± 3 days: p = 0.11).

**Conclusion:** In this multicenter retrospective study, long-term immunosuppressive therapy excluding chemotherapy was not associated with a significantly higher ICU and 3-month mortality rate in patients admitted to the ICU for septic shock.

**Compliance with ethics regulations:** N/A.

## FC-057 Clinical features and outcomes of Human Herpesvirus-6 DNAemia in critically ill patients: a retrospective multicenter analysis

### Margot Combet^1,2^, Agnès Gautheret-Dejean^3,4,5^, Charles-Edouard Luyt^6,7^, Nicolas Weiss^8,9^, Julien Mayaux^1^, Marie Lecronier^1^, Sophie Demeret^8,9^, Julie Delemazure^1^, Elise Morawiec^1^, Nicolas Gauthier^10^, Valérie Pourcher^11,12^, Alexandre Demoule^1,2^, Maxens Decavèle^1,2^

#### ^1^APHP Sorbonne Université, site Pitié-Salpêtrière, Service de Médecine Intensive-Réanimation (département R3S), Paris, France; ^2^Sorbonne Université, INSERM, UMRS1158 Neurophysiologie Respiratoire Expérimentale et Clinique, Paris, France; ^3^Université Paris cité, INSERM UMR-S 1139, 3PHM, Paris, France; ^4^AP-HP, Hôpitaux Universitaires Pitié Salpêtrière-Charles Foix, Service de Virologie, Paris, France; ^5^Université Paris cité, Faculté de Pharmacie, Laboratoire de Microbiologie, Paris, France; ^6^Service de Médecine Intensive Réanimation, Institut de Cardiologie, Groupe Hospitalier Pitié–Salpêtrière, APHP, Paris, France; ^7^Sorbonne Université, INSERM UMRS_1166-iCAN, Institute of Cardiometabolism and Nutrition, Paris, France; ^8^Unité de Médecine Intensive Réanimation Neurologique, Département de Neurologie, DMU Neurosciences et Institut de Neurosciences Translationnelles, IHU-A-ICM, Hôpital de la Pitié-Salpêtrière, AP-HP. Sorbonne Université, Paris, France; ^9^Sorbonne Université, Brain Liver Pitié-Salpêtrière (BLIPS) Study Group, INSERM UMR_S 938, Centre de recherche Saint-Antoine, Maladies métaboliques, biliaires et fibro-inflammatoire du foie, Institute of Cardiometabolism and Nutrition (ICAN), Paris, France; ^10^AP-HP, Groupe Hospitalier Pitié-Salpêtrière Charles Foix, Service d’hématologie clinique, Paris, France; ^11^APHP Sorbonne Université, site Pitié-Salpêtrière, Service des maladies infectieuses et tropicales, Paris, France; ^12^Institut Pierre Louis d’Epidémiologie et de Santé Publique (iPLESP), UMR_S 1136, Paris, France

##### **Correspondence:** Margot Combet (margotcombet@gmail.com)

*Annals of Intensive Care *2013, **13(Suppl 1)**:FC-057

**Rationale:** Human Herpesvirus 6 (HHV-6) DNAemia is not rare in intensive care unit (ICU) patients, especially in the most severely ill and in immunocompromised patients. However, evidence for a causal association of HHV-6 with diseases and link with mortality is limited. We sought 1) to measure the incidence of HHV-6 related disease in ICU patients with HHV-6 DNAemia and 2) to explore its association with ICU mortality.

**Patients and methods/materials and methods:** Retrospective multicenter cohort study in three ICUs from January 2009 to January 2022. All consecutive patients with at least one positive viral load measured by real-time PCR (expressed as log10 HHV-6 DNA copies/106 cells [log/106 cells]) in whole blood samples, performed during the ICU stay, were included. Patients with positive HHV-6 viral load were classified as reactivation (DNAemia without HHV-6 related organ failure) or HHV6 related disease (DNAemia with HHV-6 related organ failure) based on guidelines and expert adjudication committee. Patients with inherited chromosomally integrated HHV-6 were excluded. Logistic regression multivariate analysis was performed on ICU mortality.

**Results:** One hundred and sixty-eight patients were included (100 (60%) males, 46 [37 − 65] median [interquartile interval] years old, simplified acute physiology score 2 45 [32–68]). Invasive mechanical ventilation and extracorporeal lung circulation were initiated in 119 (71%) and 61 (36%) patients, respectively. Among these 168 patients, 140 (83%) patients were classified as HHV-6 reactivation and 28 (17%) as HHV-6 related disease. The two main HHV-6 diseases were encephalitis (n = 14/28, 50%) and pneumonitis (n = 12/28, 43%). Patients with HHV-6 related disease were more frequently immunocompromised than patients with HHV-6 reactivation (93% vs. 46%, p < 0.001), whereas veno-arterial extracorporeal membrane oxygenation (VA-ECMO) was more frequently observed in patients with HHV-6 reactivation (32% vs. 0%, p < 0.001). Maximum viral load was higher in patients with HHV-6 related disease than their counterpart (4.63 [3.30, 5.55] vs. 1.86 [1.55, 2.38] log/106 cells, p < 0.001). Among the 28 patients with HHV-6 related disease, only 12 (43%) received anti-HHV-6 therapies during the ICU stay. ICU mortality was 32% (n = 53). Using multivariate analysis, HHV-6 related disease (OR 3.1 95% confidence interval (CI) 1.10–8.80, p 0.03), being immunocompromised (OR 35 [5.80–218], p < 0.001) and VA-ECMO (OR 13.35 95%CI 2.30–76.8, p < 0.001) were independently associated with ICU mortality.

**Conclusion:** Organ disease could be attributable in up to 20% of ICU patients with HHV-6 DNAemia. Such HHV-6 related disease is an independent predictor of ICU mortality.

**Compliance with ethics regulations:** Yes in clinical research.

## FC-058 Heterogeneity of acquired immunodeficiency in the ICU

### Morgane Snacken^1^, David Grimaldi^1^, Daniele Casula^1^

#### ^1^Hôpital Erasme, Bruxelles, Belgique

##### **Correspondence:** Morgane Snacken (snackenmorgane@gmail.com)

*Annals of Intensive Care *2013, **13(Suppl 1)**:FC-058

**Rationale:** Post-aggressive acquired immunosuppression after septic or sterile insults is characterized among other immunological change: i) by a monocytic functional deactivation highlighted by a decrease of HLA-DR expression and ii) by the overexpression of PD1 and PDL-1 in CD4 + cells and monocytes. These markers have been shown to be associated with the development of nosocomial infections and are thus attractive therapeutic target of post-aggressive acquired immunosuppression. In this setting, the question whether these markers are concomitantly impaired has been poorly studied. In particular, the ability of HLA-DR expression to predict overexpression of PD1/PDL1 is unclear. A clear association would indicate that testing for HLA-DR expression would be sufficient both for prognostic tool (risks of nosocomial infections, mortality) and to decide a treatment even targeting PD-1/PDL-1. The aim of this work was to study the association between HLA-DR expression at the monocyte membrane and PD1-PDL 1 expression at the lymphocyte and monocyte membrane.

**Patients and methods/materials and methods:** This observational, prospective, monocentric study controlled by healthy volunteers was performed in the medico-surgical intensive care unit. HLA DR, PD1, PDL1 to J2-3 and J5-7, among other immunological markers were analyzed by FACS.

**Results:** We recruited 21 controls to monitor and calibrate the cytometry technique and define the patient analysis strategy. We also included 63 patients admitted to our ICU between November 2019 and September 2020 and a total of 47 were analyzed. Main patients characteristics are reported (table 1). We did not find any correlation between PD1/PDL1 and HLADR expression (y = 0,856x + 16,812. R^2^ = 0,0621, y = 0,1897x + 19,015. R^2^ = 0,0355.) We compared septic versus non septic profiles. Septic patients had lower HLADR and CD86 (monocyte inactivation profile) but no difference in PD1/PDL1 was found. Finally, we compared PD1 in low HLA DR (< 30%) patients with normal HLA DR (Chi2 test. P = 0,754) with no significant difference.

**Conclusion:** The study of HLA DR alone is not sufficient to characterize the immunological profile of severely assaulted patients. There is an interest in studying PD1/PDL 1 markers to better select candidates to PD-1/PDL-1 inhibition therapies.

**Reference 1**

Guignant and al. Critical Care 2011, 15:R99.

**Reference 2**

Morris and al. British Journal of Anaesthesia 111 (5): 778–87 (2013).

**Compliance with ethics regulations:** Yes in clinical research.

**Table 1 (abstract FC-058):**
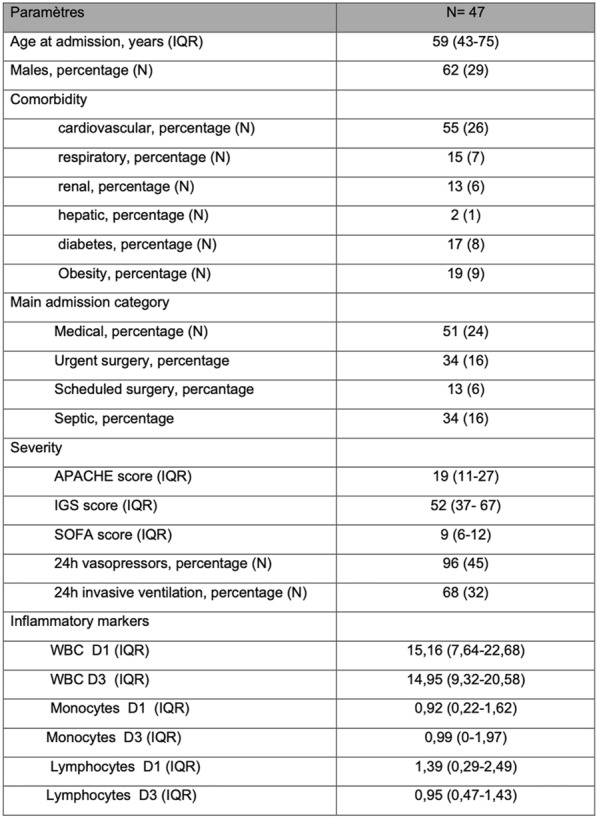
Clinical characteristics of the patients

## FC-059 Very severe imported malaria in adults treated by intravenous artesunate: PALUSTAR Study

### Chloé Tridon^1^, Cédric Laouenan^3^, Carine Roy^3^, Eric Kendjo^2^, Alexia Le Corre^4^, Paul Chabert^5^, Philippe Corne^6^, Clément Dubost^7^, Maité Agbakou^8^, Morgan Caplan^9^, Jean-François Timsit^3^, Hervé Hyvernat^10^, Marc Thellier^2^, Fabrice Bruneel^1^

#### ^1^Hôpital Mignot, Le Chesnay, France; ^2^Pitié Salpêtrière, Paris, France; ^3^Hôpital Bichat, Paris, France; ^4^Hôpital Pontchaillou, Rennes, France; ^5^Hôpital de la Croix-Rousse, Lyon, France; ^6^CHU de Montpellier, Montpellier, France; ^7^Hôpital Bégin, Saint-Mandé, France; 8CHU de Nantes, Nantes, France; ^9^CHU de Lille, Lille, France; 10Hôpital Pasteur, Nice, France

##### **Correspondence:** Chloé Tridon (chloe.tridon12@gmail.com)

*Annals of Intensive Care *2013, **13(Suppl 1)**:FC-059

**Rationale:** In endemic areas, intravenous (IV) artesunate is now firmly established as the treatment of choice for severe malaria. However, during severe imported malaria treated in France with IV artesunate, little is known about the management in the intensive care unit (ICU) of the most severe forms. As a first step of PALUSTAR Study, we sought to describe the characteristics, management and outcome of patients with a very severe malaria treated with IV artesunate over the period 2011–2019.

**Patients and methods/materials and methods:** Multicenter observational retrospective study in adults admitted for very severe malaria treated by IV artesunate. Data were collected from medical charts using standardized case-report forms, in 58 French ICU in 2011–2019. In a patient with a Plasmodium falciparum asexual parasitaemia, very severe malaria was defined by at least one criterion, during the first 72 h of ICU stay, among the following criteria: coma with Glasgow Coma Scale score < 11 and/or repeated convulsions, shock, respiratory distress, acidosis, hyperlactatemia > 5 mmol/L, and/or hospital death.

**Results:** Over the 2011–2019 period, 259 patients were included. Most were male (62.9%), and median age was 52 (IQR, 38—62). On the first day (D0) of ICU admission, 97 (37.5%) patients had impaired consciousness, 118 (45.6%) were in respiratory distress, 136 (52.5%) had shock, 100 (38.6%) had acidosis, and 98 (37.8%) had hyperlactatemia. Median SAPS II and SOFA score was 41 (IQR, 30—58) and 10 (IQR, 7—14), respectively. During the ICU stay, mechanical ventilation was required in 84 (32.4%) patients, 137 (53.1%) received vasopressive drugs and 77 (29.8%) required renal replacement therapy. Post Artesunate Delayed Hemolysis (PADH) was reported in 47 (18.1%) patients. D28 mortality was 11.2%. By multivariate analysis, three variables at ICU admission were independently associated with death at D28: SOFA score, arterial lactatemia, Glasgow Coma Scale score.

**Conclusion:** In a large cohort of patients hospitalized in the ICU with very severe imported malaria and treated by IV artesunate, the D28 mortality was 11.2%. As a second step of PALUSTAR Study, we plan to compare these patients (treated by IV artesunate) with those of an historical cohort (treated with IV quinine).

**Compliance with ethics regulations:** Yes in clinical research.

## FC-060 Severe Leptospirosis in ICU in the French West Indies: Statistical clustering and prognosis factors- a retrospective monocenter study (2014–2022)

### Laurent Camous^1^, Jean-David Pommier^1^, Benoit Tressieres^1^, Frederic Martino^1^, Marc Valette^1^, Sébastien Breurec^1^

#### ^1^CHU de Guadeloupe, Les Abymes, France

##### **Correspondence:** Laurent Camous (laurent.camous@chu-guadeloupe.fr)

*Annals of Intensive Care *2013, **13(Suppl 1)**:FC-060

**Rationale**: Study characterics and outcome of patients with biologically confirmed Leptospirosis admitted in the intensive care unit (ICU) of the Guadeloupe Teaching Hospital.Identify patients’ clusters by multiCorrespondence analysis.

**Patients and methods/materials and methods**: All patients admitted in the ICU for biologically Leptospirosis between January 2014 to December 2022 were included in the study. Multiple Correspondence statistical analysis (MCA)was realized on the overall population. Using univariate and bi-variate analysis, we assessed risk factors at ICU admission and during ICU course associated with death.

**Results:** On the study period, 130 patients admitted in the ICU had confirmed leptospirosis. Median age and SAPS II were 56 years and 48 respectively. During ICU course, acute respiratory failure was present in 34% of the patients and 26% were mechanically ventilated. Patients had shock in 52% (n = 67) and 21% (n = 27) had neurological involvement. Myocardial involvement was present in 41% (n = 53) of the patients. Based on clinical and biological parameters at ICU admission, MCA identified three patient clusters: “mild leptospirosis” (n = 62), “neurological leptospirosis” (n = 27) and “multiple organ failure (MOF)” (n = 41) with different ICU courses and outcomes. Overall mortality rate at day 28 was 13%. Risk factors associated with death in uni/bivariate analysis were shock, acute respiratory failure, myocardial damage, neurological involvement at ICU admission and need for dialysis during ICU course.

**Conclusion:** Leptospirosis is an endemic tropical area zoonosis, responsible for severe manifestations with frequent organ failures. Identification in endemic areas of patients with potential life-threatening worsening is essential. In our study, one of the determinent at ICU admission for evolution to a severe form was cardiac involvement which helped to identify two different sub-populations with different outcomes. A third cluster, corresponding to neurological forms seems to represent a different, late form of the disease.

**Compliance with ethics regulations:** Yes in clinical research.

**Figure 1 (abstract FC-060):**
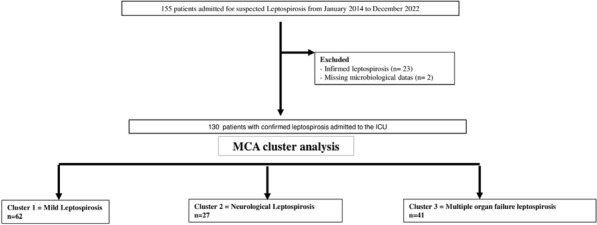
Chart flow of biologically proven leptospirosis patients admitted in the ICU in University Hospital of Guadeloupe on the study period (2014–2022)

## FC-061 Severe community-acquired Acinetobacter baumannii pneumonia in La Reunion: a retrospective case–control study

### Giacomo Rotini^1^, Axel De Mangou^1^, Agathe Combe^1^, Mathilde Nativel^1^, Guillaume Miltgen^1^, Julien Jabot^1^, Nicolas Allou^1^, Charles Vidal^1^

#### ^1^Centre hospitalier universitaire Felix Guyon, Saint-Denis, France

##### **Correspondence:** Giacomo Rotini (giacomo.rotini17@gmail.com)

*Annals of Intensive Care *2013, **13(Suppl 1)**:FC-061

**Rationale:** *Acinetobacter baumannii* (Ab) is a well-known nosocomial pathogen that has emerged in the last decades as a cause of community-acquired pneumoniae (CAP) in tropical and subtropical regions. Few global epidemiological studies on CAP-Ab have been published to date and, to our knowledge, no data are available on this pathology in France. The objective of this work is to perform an epidemiological analysis of CAP caused by Ab in Reunion Island and compare them with severe CAP due to other pathogens.

**Patients and methods/materials and methods:** Case–control study listing severe CAP-Ab cases hospitalized in one of the two intensive care unit (ICU) of Réunion’ University Hospital, during the period 2014–2022; and compare them with an historical cohort (PAC_RUN), obtained by retrospective chart (2016–2021) of severe community-acquired pneumonia, in which PAC-Ab were excluded (n = 4). CAP-Ab was confirmed by microbiological respiratory samples and/or blood cultures realized at the admission in ICUs. All patients who presented with with nosocomial infection were excluded (n = 50).

**Results:** During the study period, eight CAP-Ab cases were identified, giving an incidence of 0.1 case for 100 000 people/year. By comparing with CAP no Ab (n = 761), patients had more alcohol disorders (p = 0.005) and a lower BMI (p = 0.007). Six CAP-Ab occurred during wet season (p = 0.06). Mortality was higher (62,5% vs 24,3%, p = 0.02) and time to death was shorter (median 2 days vs 7, p = 0.009) in CAP-Ab group. Bacteremic pneumonia was strongly associated with CAP-Ab (p = 0.004). Significant differences were found for need for renal replacement therapy (p < 0.001), catecholamines (p = 0.01) and mechanical ventilation (p = 0.03). Ab strains isolated had low resistance pattern, all strains were found sensitive to ceftazidime, cefepime, piperacillin-tazobactam, ciprofloxacine, gentamicin, and imipenem. All patients with CAP-Ab received first line inappropriate antibiotic therapy.

**Conclusion:** Severe CAP-Ab have a low incidence but a fulminant course with a high mortality. Some demographics and clinical features, such as a fulminant pneumonia during the rainy season among an alcoholic patient, should suggest this pathogen prompting an antibiotic therapy targeting *A. baumannii*.

**Reference 1**

Dexter C, Murray GL, Paulsen IT, Peleg AY. Community-acquired Acinetobacter baumannii?: clinical characteristics, epidemiology and pathogenesis. Expert Rev Anti Infect Ther. 4 mai 2015;13(5):567?73.

**Reference 2**

Davis JS, McMillan M, Swaminathan A, Kelly JA, Piera KE, Baird RW, et al. A 16-Year Prospective Study of Community-Onset Bacteremic Acinetobacter Pneumonia. Chest. oct 2014;146(4):1038?45.

**Compliance with ethics regulations:** Yes in clinical research.

**Table 1 (abstract FC-061):**
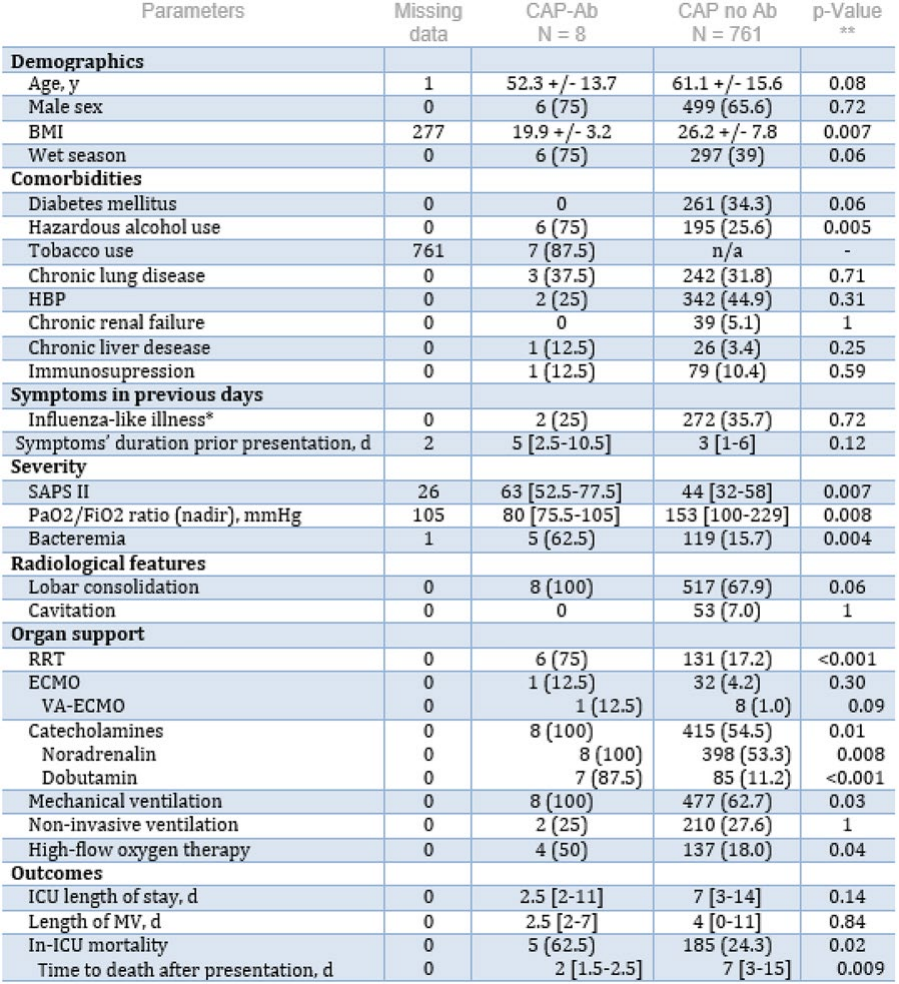
Comparison of CAP-Ab and CAP no Ab

## FC-062 Clinical spectrum and outcome of adult patients with emphysematous pyelonephritis requiring ICU: a French multicenter retrospective cohort study

### Keyvan Razazi^1^, Pierre-Louis Blot^1^, Amélie Renou^2^, Yannis Lombardi ^3^, Laurent Camous^4^, Elsa Moncomble^1^, Guillaume Carteaux^1^, Nicolas De Prost^1^, Armand Mekontso Dessap ^1^, Study Group Pyelemphy^1^

#### ^1^Médecine intensive et réanimation, AP-HP (Assistance Publique-Hôpitaux de Paris), Hôpitaux universitaires Henri Mondor, Créteil, France; ^2^Intensive Care Unit, Reunion University Hospital, Saint-Denis, France, Saint-Denis, France; ^3^Service de néphrologie, AP-HP (Assistance Publique-Hôpitaux de Paris) Tenon,, Paris, France; ^4^Service de réanimation Centre Hospitalier Universitaire de Guadeloupe, Les Abymes, France

##### **Correspondence:** Keyvan Razazi (keyvan.razazi@aphp.fr)

*Annals of Intensive Care *2013, **13(Suppl 1)**:FC-062

**Rationale:** Emphysematous pyelonephritis (EP) is a rare and severe necrotizing infection of the urinary tract, with gas accumulation, probably produced through a glucose fermentation process within the collecting system, the renal parenchyma, and/or the perirenal tissue. Data on emphysematous pyelonephritis in ICU are scarce. We aimed at reporting the clinical features and outcomes of adult patients admitted in the intensive care unit (ICU) for an infectious EP, as well as the predictive factors for nephrectomy and mortality.

**Patients and methods/materials and methods:** A 21-year national multicenter retrospective cohort study in 48 ICUs in France from 2001 to 2021, including adult patients admitted for an infectious EP. Primary outcome variables included ICU mortality and nephrectomy during the follow-up period (time between ICU admission and amputation, death or end of follow-up).

**Results:** A total of 115 patients were included in the study. We herein present the preliminary results of 107 patients. Patients had a median age of 62 years [54; 72], with a female predominance (64%), and 74% of them were either overweight or obese. Diabetes mellitus was present in 62% of cases. The median SAPS2 score at ICU admission was 50 [37; 66] and the median SOFA was 8 [5; 12]. Most patients fulfilled septic shock criteria (68%). A urinary tract obstruction was found in 50 patients (48%). Regarding surgical procedures, urine drainage or percutaneous drainage was performed in 60 patients (56%) and nephrectomy was performed in 23 patients (21%). Enterobacteriaceae represented 93% of microbiological documentation, E. coli being the most frequently identified species (57%). Mortality at 90 days was 21%. Factors associated with day 90 mortality were SOFA, and SAPS2 scores, and presence of a septic shock.

**Conclusion:** Emphysematous pyelonephritis carries a high mortality and morbidity. Need for nephrectomy was not infrequent.

**Compliance with ethics regulations:** Yes in clinical research.

## FC-063 Neurophysiological characteristics of mismatch negativity and others auditory evoked potentials responses to looming and receding deviants sounds for future assessment of disorder of consciousness

### Sarah Benghanem^1,3,4^, Estelle Pruvost Robieux^2,3,4^, Coralie Joucla^5^, Alain Cariou^1,4^, Jean Julien Aucouturier^5^, Martine Gavaret^2,3,4^

#### ^1^APHP.Centre, Hôpital Cochin, Paris, France; ^2^Neurophysiology and Epileptology department, GHU Psychiatry & Neurosciences, Sainte Anne, Paris, France; ^3^INSERM UMR 1266, FHU NeuroVasc, Institut de Psychiatrie et Neurosciences de Paris-IPNP, Paris, France; ^4^University Paris Cité, Medical School, Paris, France; ^5^Université de Franche-Comté, SUPMICROTECH, CNRS, institut FEMTO-ST, Besançon, France

##### **Correspondence:** Sarah Benghanem (sarah.benghanem@aphp.fr)

*Annals of Intensive Care *2013, **13(Suppl 1)**:FC-063

**Rationale:** Mismatch negativity (MMN) is elicited during an oddball paradigm, using frequent sounds (“standards”) mixed with rare sounds (“deviants”). MMN response is mainly used for awakening prediction of disorder of consciousness(1). The use of emotional sounds, as looming sound intensity, might improve the MMN prognostic performance although they are usually not used for MMN. We hypothesize that the acoustics characteristics of deviant auditory stimulus could modify the neurophysiological characteristics of auditory evoked potentials.

**Patients and methods/materials and methods:** We assess 18 healthy volunteers with 64-channels EEG and compared MMN responses with three modalities of deviant (looming, receding and flat) during an oddball paradigm (80% standard, 6.7% of each deviant). Deviant sounds duration was 600 ms with a gradual linear increased (looming) or decreased (receding) intensity of 15db. Deviant flat duration was 600 ms without intensity variation, standard sounds were shorter (300 ms, no intensity variation).

**Results:** Compared to receding sounds, looming deviants elicited a later (206 vs 175 ms, p = 0.02) and a wider (area under curve AUC 0.116 vs 0.053, p = 0.002) MMN. Looming, receding and flat deviants also elicited a later potential at 600 ms, with a higher peak amplitude (-1.96 µV vs -1.43 µV, p = 0.01) and a wider AUC (0.26 vs 0.18, p = 0.02) for looming than for flat sounds. Our interpretation is that the first component purely reflects the physical characteristics of the stimuli, while the second component reflects a higher-level, cognitive emphasis of looming sounds.

**Conclusion:** Looming and receding “3D” sounds elicit different MMN responses compared to flat. Looming sounds may act as a warning clue (2) and may constitute a trigger to elicit higher level of consciousness processing.

**Reference 1**

André-Obadia N, Zyss J, Gavaret M, Lefaucheur JP, Azabou E, Boulogne S, et al. Recommendations for the use of electroencephalography and evoked potentials in comatose patients. Neurophysiol Clin. juin 2018;48(3):143?69.

**Reference 2**

Bach DR, Furl N, Barnes G, Dolan RJ. Sustained Magnetic Responses in Temporal Cortex Reflect Instantaneous Significance of Approaching and Receding Sounds. Snyder J, éditeur. PLOS ONE. 30 juill 2015;10(7):e0134060.

**Compliance with ethics regulations:** Yes in clinical research.

**Figure 1 (abstract FC-063):**
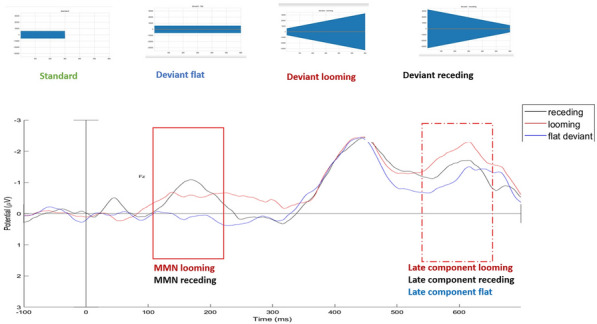
Evoked potentials (EPs) with different waves between deviants and standard tones, elicit mismatch negativity and late auditory EPs

## FC-064 PRES in ICU: retrospective observational study of cases of patients with posterior reversible encephalopathy syndrom hospitalized in 6 Intensive Care units in a French region

### Juliette Pocquet^1^, Stephan Ehrmann^1^, François Barbier^2^, Liliana Dominte^1^, Julie Badin^3^, Florent Bavozet^4^, Walid Nicola^6^, Pierre Kalfon^5^, Grégoire Muller^2^, Charlotte Salmon Gandonniere^1^

#### ^1^CHRU de Tours, Tours, France; ^2^CHR d'Orleans, Orléans, France; ^3^CH de Blois, Blois, France; ^4^CH de Dreux, Dreux, France; ^5^CH de Chartres, Chartres, France; ^6^CH de Montargis, Montargis, France

##### **Correspondence:** Juliette Pocquet (juliette@pocquet.fr)

*Annals of Intensive Care *2013, **13(Suppl 1)**:FC-064

**Rationale:** Posterior reversible encephalopathy syndrome (PRES) is a clinico-radiological entity gathering unspecific symptoms (headache, confusion, visual disturbances, seizures or coma), classically associated with a sudden rise in blood pressure, related to white matter edema. Brain MRI is the gold standard exam, with bilateral and symmetrical T2 and FLAIR hypersignals and T1 hypo and/or isosignals, predominant in the parieto- occipital cortico-subcortical regions. These symptoms and radiological abnormalities are most often reversible. PRES is a rare syndrome, but with technical advances in neuroradiology, more and more cases are being diagnosed. More than 40% of PRES require hospitalization in intensive care unit (ICU). Very few studies focus on ICU patients in the literature (clinical cases or cases series). The largest retrospective cohort was conducted by S. Legriel and included 70 patients (1). Our goal was to characterize the population and the prognostic factors.

**Patients and methods/materials and methods:** We conducted an observational, retrospective and multicentric study. From January 1, 2009 to December 31, 2020, we selected all the medical records of adult patients hospitalized in 6 ICUs in a French region with PRES via the CIM 10 coding and the brain MRI reports. 50 patients were included in our study.

**Results:** Most of our patients were women (66%), with a median age of 52 years-old. PRES was mainly attributed to drugs (44%), hypertension (28%), and pre-eclampsia (14%); 24% did not have arterial hypertension; a sepsis was associated in 46% of our cases. More than half of the patients required mechanical ventilation, 38% had acute kidney injury; 80% had neurological symptoms on admission, and 70% consciousness disorders. Thirty-two percent had sequelae at day 90 (seizures, persistent deficit, cognitive alteration), 28% had an unfavorable outcome (Glasgow Outcome Scale < 5) at day 180 and 10% died (table 1). The risk factors associated with an unfavorable outcome were extra neurologic organ failure at the time of admission (OR 6,66 [1,7–27]) and the occurrence of acute renal failure requiring dialysis (OR 24 [2,5–229]). These two factors were also risk factors for the persistence of sequelae (OR 4.58 [1.25–16.8] and 6.4 [1.07–38], respectively). Pre-eclampsia seemed to be a protective factor for good outcome, but the difference was not significant.

**Conclusion:** Our epidemiological data are similar to those described in the literature. The term "reversible" seems inappropriate since we found a significant rate of neurologic sequelae and unfavorable outcomes. Our results plead for a specific follow-up in PRES patients.

**Reference 1**

Legriel S, Schraub O, Azoulay E, Hantson P, Magalhaes E, Coquet I, Bretonniere C, Gilhodes O, Anguel N, Megarbane B, Benayoun L, Schnell D, Plantefeve G, Charpentier J, Argaud L, Mourvillier B, Galbois A, Chalumeau-Lemoine L, Rivoal M, Durand F, Geffroy A.

**Compliance with ethics regulations:** Yes in clinical research.

**Table 1 (abstract FC-064):**
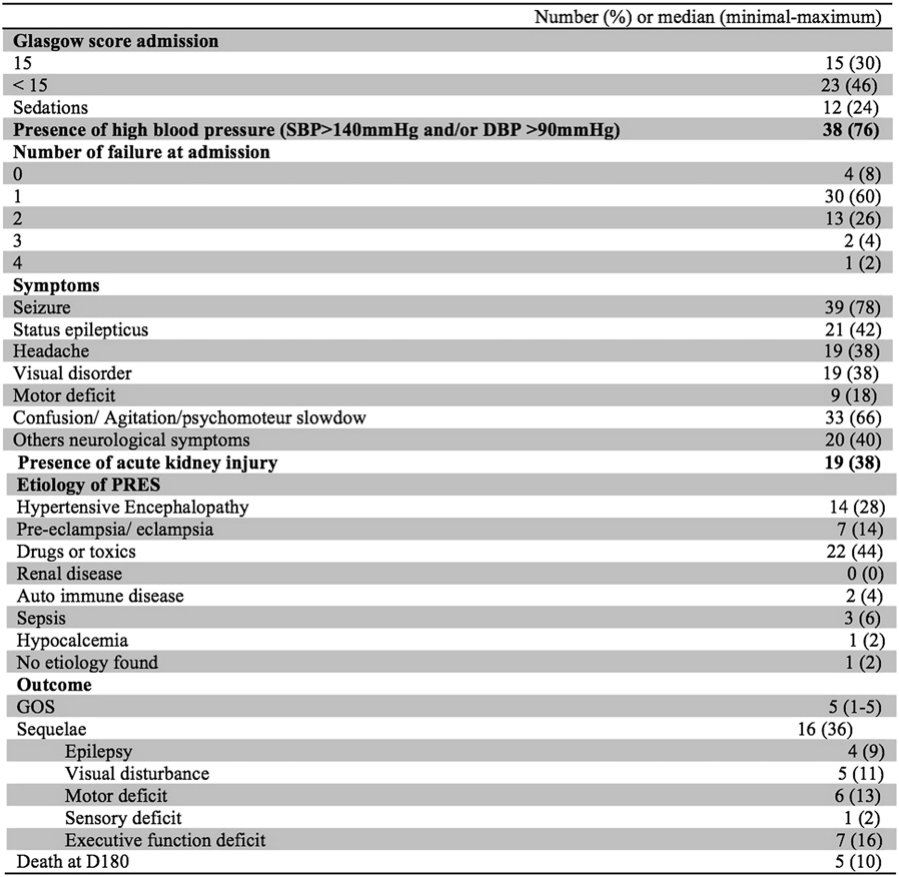
Characteristics of patients (n = 50)

## FC-065 Electroencephalogram in intensive care, analysis of the retrospective cohort RéVE (RéaÉValuationEeg)

### Adam Celier^1^, Clémence Marois^1^, Alexandre Demoule^1^, Romain Sonneville^2^, Lambrecq Virginie^1^, Benjamin Rohaut.^1^

#### ^1^Pitié-Salpétrière, Paris, France; ^2^Bichat Claude-Bernard, Paris, France

##### **Correspondence:** Adam Celier (adam.celier@aphp.fr)

*Annals of Intensive Care *2013, **13(Suppl 1)**:FC-065

**Rationale:** While electroencephalogram (EEG) plays an important role in the management of many resuscitation conditions, few studies have evaluated its use and impact on patient management in intensive care units (ICU). The prevalence of pathologies requiring EEG in a general ICU and most frequently identified EEG abnormalities is poorly documented.

**Patients and methods/materials and methods:** This project is a retrospective study of the RéVE (RéaÉValuationEeg) cohort. This cohort includes all patients admitted to an ICU and who had a bedside EEG recording between 1 January 2017 and 1 April 2020. For each patient, demographic, clinical, paraclinical and evolution data were collected. For each report, many elements concerning the EEG background activity and EEG abnormalities were collected, as well as the main diagnosis.

**Results:** Over the period, 305 patients had an EEG recording in this ICU. Based on the number of hospitalizations in the department in 2018, 11.5% of patients admitted to the ICU have an EEG during their stay. The reasons for admission to the ICU resulting in the performance of an EEG during the patient's stay were mainly represented by three reasons: disease of consciousness (DOC) (25%), status epilepticus (20%) and cardiac arrest (18%). Mean age was 57 years old. History of epilepsy was known for 20% of the patients. EEGs were mostly abnormal, with disparities between the 3 main groups. A majority (44%) of EEG findings were of the slowing-sedation type, followed by metabolic encephalopathy (16%), post-anoxic encephalopathy (14%). Epilepsy was reported in only 7% of EEGs. EEG trace was continuous for 87% of the patients, but only for 43% of the cardiac arrest patients. Slow triphasic waves were seen in 15% of the DOC patients, epileptic abnormalities in 18% of the epilepsy group. Only 14 seizures (5%) were recorded. Two-thirds of the EEGs were considered to have had an impact on patient management.

**Discussion:** The analysis of all EEG reports allowed us to have an overview of the EEG findings in the ICU. This work sheds light on the impact of EEG in ICU and provides a better understanding of intensivists expectations with regard to this examination.

**Conclusion:** EEG is now an important part of the management of many intensive care patients. In this single-centre observational study, the three main indications of an EEG in the ICU were disorder of consciousness, status epilepticus and cardiac arrest.

**Compliance with ethics regulations:** Yes in clinical research.

## FC-066 Cyclic recurrence of seizures during super-refractory status epilepticus

### Maeva Le Goïc^1^, Mario Chavez^1^, Sophie Demeret^1^, Meriem Bouguerra^1^, Baptiste Criniere^1^, Vincent Navarro^1^, Virginie Lambrecq^1^

#### ^1^CHU La Pitié Salpêtrière, Paris, France

##### **Correspondence:** Maeva Le Goic (maevalgo@gmail.com)

*Annals of Intensive Care *2013, **13(Suppl 1)**:FC-066

**Rationale:** Status epilepticus (SE) is an acute brain condition of extreme severity which is associated with significant morbimortality. Its severity depends on many factors including age, aetiology, and duration of SE. Some patients may reach a prolonged super-refractory SE, requiring the maintenance of a prolonged induced coma and continuous EEG monitoring to early diagnose and treat non-convulsive SE. The aim of this study was to develop a descriptive and comprehensive approach of a specific electric pattern of seizures that has a periodic/cyclic mode of recurrence.

**Patients and methods/materials and methods:** In a retrospective single-centre study, we screened 138 patients admitted with SE in a neurological ICU, between 2016 and 2022. A subgroup of SE patients with cyclic electrographic seizures i.e. displaying recurrent seizures at regular intervals was compared to SE patients without cyclic seizures (controls) on several pre-set clinical, biological and electrophysiological variables, along with their on-going medication (sedatives, antiepileptic drugs and concomitant treatments that may lower the seizures threshold). Their clinical outcomes (rate of mortality, functional status, and sequel of epilepsy at ICU discharge and after six months) were also compared. A computerized seizure detection method was used in complement to the standard visual EEG analysis and allowed characterization of seizures features as well as their dynamics of occurrence during the whole continuous EEG monitoring period (42 days on average).

**Results:** Our results show that patients with cyclic seizures had a more important seizures burden, were younger, stayed longer in ICU and all reached a prolonged super refractory status epilepticus condition in comparison to the control population. Moreover, they displayed a poorer clinical outcome at discharge, with a higher rate of sequelar refractory epilepsy. In majority, cyclic seizures had no clinical correlates, a focal ictal onset and a shorter duration and, strikingly often organized into clusters. When comparing the first and terminal intra-cluster duration of seizure, this latter was longer which may imply a better capacity to activate inhibitory mechanisms and terminate a cluster. In addition to these endogenous mechanisms, our assumption that multiple associated factors contribute to their cessation and/or generation was confirmed as metabolic disturbances such as renal failure, hypomagnesemia, higher vigilance, clinical severity and a more aggressive treatment were associated with a higher risk of cyclic seizures.

**Conclusion:** This study may help provide insight into the underlying pathophysiology of cyclic seizures and may help to improve the management of super-refractory SE patients.

**Compliance with ethics regulations:** Yes in clinical research.

**Figure 1 (abstract FC-066):**
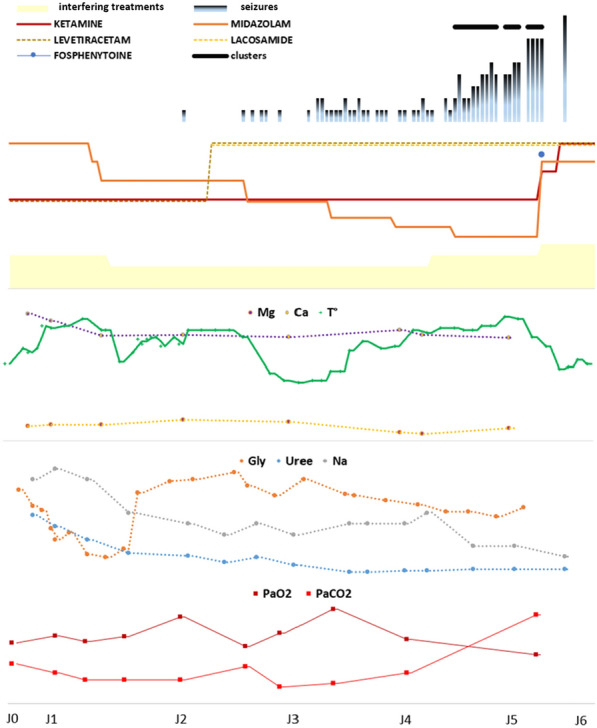
Associated factors for cyclic seizures occurrence

## FC-067 Phenytoin versus valproate acid in the management of status epilepticus

### Ines Sdiri^1^, Hamdi Hamdene Doghri^1^, Wafa Homrani^1^, Ines Sedghiani^1^, Imen Klai^1^, Imen Zaghdoudi^1^, Nebiha Falfoul Borsali^1^

#### ^1^Hôpital habib thameur, Tunis, Tunisie

##### **Correspondence:** Ines Sdiri (sdiri.ynes@gmail.com)

*Annals of Intensive Care *2013, **13(Suppl 1)**:FC-067

**Rationale:** Phenytoin and valproate are commonly used antiepileptic drugs. It's generally believed that phenytoin is more effective for partial onset seizures and valproate is more effective in generalized onset seizures. Although there'is no evidence between this two antiepileptic molecules in the management of status epilepticus. We aim to compare phenytoin and valproate when used as antiepileptic drug in the management of status epilepticus in the prognostic.

**Patients and methods/materials and methods:** We conducted a retrospective study in an intensive care unit including critically ill patients with status epilepticus between January 2016 and December 2022. We divided into 2 groups: those who received valproate acid (G1) and those who received phenytoin (G2). Groups were matched on age, gender, SAPSII and APACHE scores, severity of distress on admission and etiologies for status epilepticus.

**Results:** Eighty patients with status epilepticus were enrolled in our institution. The median age was 53 [16–90] years and sex-ratio was 4,71. 37,5% had epilepsy, 27,2% had diabetes and 25% had HTA. Only 4,3% had renal failure. The mean APACHEII and SAPS II scores were respectively 18 ± 2 and 37 ± 4. Only 8,5% of patients had an electroencephalogram which demonstrated a comiality in third cases. Generalized tonic–clonic movement was the most frequent type of seizure (87,2%). The differents etiologies of status epilepticus were: cerebrovascular accidents (hemorrhagic stroke in 21,7% and ischemic stroke in 10,9%); switching off the epileptic drug (19,6%); central nervous system infection (meningitis 10,9%); cerebral thrombophlebitis (6,5%); drug toxicity (6,8%) and metabolic abnormalities (2,2%). The management of secondary neurological injuries was achieved in 93,6% of cases. Invasive mechanical ventilation with neurosedation were needed for 76,6% of patients and 38,3% of patients had shock. The complications of status epilepticus were observed in 13,2% cases: hemiplegia (6,6%), frontal lobe syndrome (1,7%), vegetative state (6,6%) and facial palsy (1,7%). ICU mortality was 23,5%. ICU length of stay was 13 ± 22 days [1–132]. There were 37 patients in G1 versus 43 in G2. There was no difference in the etiologies (p = 0,329) or the type of seizure (p = 0,11) between the two groups. A repeat crisis was observed in 10,6% of cases without difference between the two groups (p = 0,6). We noticed a higher incidence of the need of mechanical ventilation, nosocomial infection, need of vaso-active drugs and mortality in G1 without signification (p:0,373, p = 0,56, p = 0,34 and p = 0,34 respectively).

**Conclusion:** We did not find any evidence that a significant difference exists between phenytoin and valproate for the outcomes. However, a propective study is needed to identify bias and to confirm these results.

**Compliance with ethics regulations:** Yes in clinical research.

## FC-068 Myasthenic crises in the intensive care unit: Epidemiological, therapeutic and evolutionary characteristics

### Rabeb Hammami^1^, Mahmoud Marzouk^1^, Rym Karaborni^1^, Sabeur Thamlaoui^1^, Nader Baffoun^1^, Chokri Kaddour^1^

#### ^1^Institut National De Neurologie, Tunis, Tunisie

##### **Correspondence:** Rabeb Hammami (hammamirabeb2@gmail.com)

*Annals of Intensive Care *2013, **13(Suppl 1)**:FC-068

**Rationale:** Myasthenia gravis is a disease of the neuromuscular junction that progresses in bouts called myasthenic attacks. The latter leads to acute respiratory failure which may require respiratory assistance and a prolonged stay in the intensive care unit. The aim of this study was to describe the epidemiological, therapeutic, and evolutionary characteristics of myasthenic attacks managed in an intensive care unit and to study the factors associated with mortality.

**Patients and methods/materials and methods:** This was a retrospective, descriptive study conducted in our intensive care unit over a 10-year period between 2012 and 2021. Patients admitted to the intensive care unit for the myasthenic crisis, aged over 18 years, and who required mechanical ventilation were included. Patients followed for congenital myasthenia were not included. The epidemiological (age, gender, and history), therapeutic (therapeutic adjustment; plasmapheresis and immunoglobulins), and evolutionary (infectious and thromboembolic complications) characteristics of the patients retained for the study were described. Factors associated with mortality were also studied.

**Results:** Fifty-seven patients were selected for the study. The average age was 41.21 ± 13.57 years. The sex ratio was 0.23: 11 men (19.29%) and 46 women (80.70%). The average age of discovery of myasthenia was 38.44 ± 13.50 years, and that of the first myasthenia crisis at 40 years ± 13.78. Diabetes dominated the medical history (10.5%). The majority of myasthenic attacks were triggered by an infectious episode (47.36%) or a therapeutic deviation (31.57%). Regarding the specific treatment, 17 attacks were treated with intravenous immunoglobulin, and 17 attacks were treated with plasmapheresis. 23 attacks did not require specific treatment because of the effectiveness of the therapeutic adjustment or because of Clinical instability contraindicating immunoglobulins and plasmapheresis. The mean duration of mechanical ventilation was 15 ± 15.94 days, and the mean length of stay was 20.42 days ± 22.49. The mortality rate was 17.54%. The most frequent direct cause of death was a septic shock in 80% of cases, followed by cardiogenic shock. The factors associated with mortality—shown in table 1—in univariate analysis were a high APACHE III score on admission and the presence of a thymoma. The use of plasmapheresis was significantly associated with a higher survival rate.

**Conclusion:** The advent of resuscitation has markedly changed the prognosis of myasthenia gravis, with respiratory support and the development of specific immunomodulatory therapy having considerably reduced the mortality rate.

**Compliance with ethics regulations:** Yes in clinical research.

**Table 1 (abstract FC-068):**
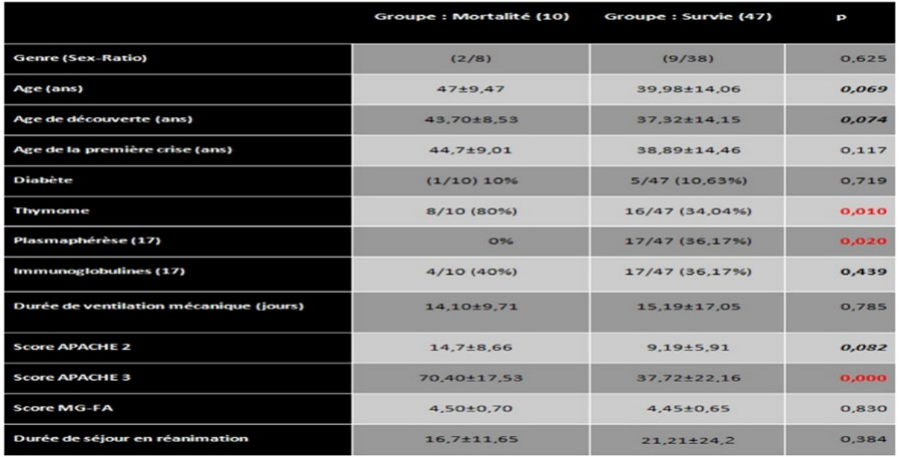
Factors associated with mortality

## FC-069 Follow-up at 1 year of the quality of life of patients with Guillain-Barré syndrome

### Badreddine Errouane^1^, Abdallah Mechebbek^1^, Belarbi Khemliche^1^, Ourida Drouiz^1^

#### ^1^Etablissement Hospitalier Universitaire d'Oran, Oran, Algerie

##### **Correspondence:** Badreddine Errouane (badreddine468@gmail.com)

*Annals of Intensive Care *2013, **13(Suppl 1)**:FC-069

**Rationale:** Guillain-Barré syndrome (GBS) is the most common cause of acute extensive paralysis. 30% of patients will require mechanical ventilation and 10% of them will have very debilitating motor sequelae and motor disabilities that can affect their quality of life. Quality of life is a multidimensional concept defined by an individual's perception of their position in life, in the context of the culture and value system in which they live, in relation to their goals, expectations, standards and concerns.

**Patients and methods/materials and methods:** Our study is prospective observational, carried out in a medical intensive care unit. Included were patients over 16 years of age with acute polyradiculoneuritis, GBS type. On their discharge, the patients were either reviewed as part of the post-resuscitation consultation at 03 months, 06 months and one year. Quality of life was measured by a standardized test (WHO in 1995), namely the “Medical Outcome Study Short Form-36 Health Survey” (MOS SF-36). Our results will be compared to the reference values in terms of quality of life of the national study "TAHINA": Transition and Health Impact In North Africa.

**Results:** 32 patients were collected with an average age of 40.75 years (16–72 years) and a sex ratio of 1.67. The average Hughes handicap score was 4.03. The extension phase lasted an average of 8.06 days and the plateau phase 10.90 days. 14 patients required mechanical ventilation, the average duration of which was 16.69 days. Plasma exchanges were performed in all patients. The average time to resume walking with assistance was 44.19 days and walking without assistance was 89.62 days. For quality of life, the results were compared to the national reference, the “TAHINA” study (11). At 03 months and 06 months, the evaluation scales are all lowered significantly compared to the national reference, both in physical and psychological dimensions. At 01 year, the calculated means are not all significantly different from the national reference means.

**Conclusion:** GBS is a serious pathology that sometimes leads to significant and lasting motor disability (up to 01 year). This motor handicap with its psychic repercussions will alter the quality of life of these patients. The study shows that the Values of the quality of life (MOS-SF36), tend to improve gradually and the values become comparable to the general population only after 01 year of evolution. Overall, our results are comparable to data from the international literature.

**Reference 1**

Forsberg, A., et al., Disability and health-related quality of life in Guillain-Barré syndrome during the first two years after onset: a prospective study. Clin Rehabil, 2005. 19(8): p. 900–9.

**Reference 2**

Institut national de santé publique (INSP)—Programme INCOMED. (2007). « Etude TAHINA—La transition épidémiologique et son impact sur la santé dans les pays nord africains». Alger.

**Compliance with ethics regulations:** Yes in clinical research.

## FC-070 Trends in the use of noninvasive respiratory support use over consecutive COVID-19 waves: toward a protective non-invasive management

### Amir Bedhiafi^1,2^, Radhouane Toumi^1,2^, Hajer Zouari^1^, Emna Ennouri^1,2^, Khaoula Meddeb^1,2^, Imen Ben Saida^1,2^, Mohamed Boussarsar^1,2^

#### ^1^Medical Intensive Care Unit, Farhat Hached University Hospital, Sousse, TUNISIE; ^2^Research Laboratory N° LR12SP09. Heart Failure. Farhat Hached University Hospital, University of Sousse, Sousse, Tunisie

##### **Correspondence:** Amir Bedhiafi (bedhiafi.emir@gmail.com)

*Annals of Intensive Care *2013, **13(Suppl 1)**:FC-070

**Rationale:** Non-invasive respiratory support (NIRS) has always been controversial in the management of acute hypoxemic respiratory failure. During the COVID-19 outbreak, the use of NIRS evolved progressively in line with the disease spread and the pathophysiological processes understanding. This study aims to describe trends in the use of NIRS over the consecutive COVID-19 waves.

**Patients and methods/materials and methods:** This is a single-center retrospective observational study conducted in the medical ICU of a Tunisian University Hospital over two years from March 2020 to March 2022 covering four consecutive waves of the disease spread. COVID-19 waves were defined as first, from March 2020 to April 2020, second, from August 2020 to May 2021, third, from June 2021 to September 2021 and fourth, from December 2021 to March 2022. All consecutive patients admitted for COVID-19-related acute respiratory failure (ARF) and requiring NIRS were included. Baseline demographics and clinical data were collected. Univariate descriptive analysis was performed.

**Results:** During the study period, 407 patients were included out of 512 ICU-admission for COVID-19-related ARF. During the first-wave, eleven critical patients were admitted, among them only two received Non-invasive positive pressure ventilation (NIPPV). In the second-wave, 149 patients were included, median age 66 [60–74], PaO2/FiO2 112 [79–166] mmHg, and SAPS II 31[26–37]. NIPPVuse was predominant (66%), either through NIV alone (44%) or alternating with High-Flow Nasal Cannula (HFNC) (22%). HFNC was used alone in 34% of cases and awake proning was performed in more than half of patients (62%). 61% required intubation within 2 [1–4] days with an overall mortality of 55.7%. Over the third-wave, 203 patients were included, median age 59 [48–70], PaO2/FiO2 107 [77–164] mmHg, and SAPS II 27[22–32]. Respiratory management strategies have evolved towards the predominantly combined use of HFNC (74%) and awake proning (86%). NIPPV was used in 20% of cases as rescue therapy through an attempt of alveolar recruitment before escalation to invasive mechanical ventilation. The intubation rate was lower at 41.5% (p = 0.001) with a shorter delay of 1 [1–2] days (p = 0.07). The mortality rate was also lowered to 32.3% (p = 0.001). About the fourth-wave, 57 patients were included. Respiratory management was quite similar to the third wave. HFNC was the first-line treatment in 63% of patients. Awake proning was performed in 63%. NIPPV attempt was required in 20% of cases.

**Conclusion:** NIRS use in the management of COVID-19-related ARF has evolved gradually as the disease has progressed. Combined HFNC with awake proning as first-line treatment might be associated with better outcomes.

**Compliance with ethics regulations:** Yes in clinical research.

**Figure 1 (abstract FC-070):**
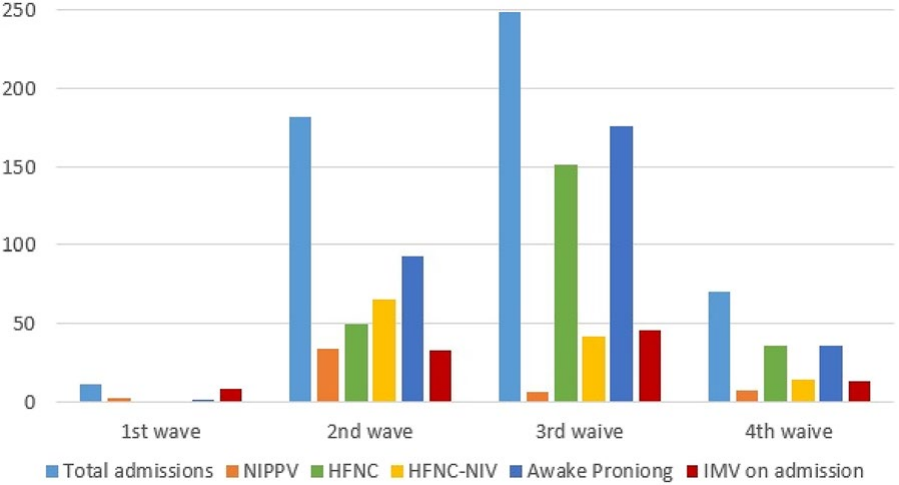
Evolving use of non-invasive respiratory support aver consecutive waves

## FC-071 Awake prone positioning in patients with Covid-19: a retrospective analytic study about 1069 patients

### Inass Arhoun El Haddad^1^, Amine Elmouhib^1^, Younes Oujidi^1^, Ilyass Laaribi^1^, Houssam Bkiyar^1^, Brahim Housni^1^

#### ^1^CHU Mohammed VI OUJDA, Oujda, MAROC

##### **Correspondence:** Inass Arhoun El Haddad (iarhounelhaddad@gmail.com)

*Annals of Intensive Care *2013, **13(Suppl 1)**:FC-071

**Rationale:** If early and prolonged prone positioning reduces mortality in patients on invasive ventilation with acute respiratory distress syndrome, its role in conscious patients remains a subject of debate and research. The objective of our study was to evaluate the effectiveness of awake prone positioning in preventing intubation or death in spontaneously breathing patients with COVID-19 associated acute respiratory failure. The primary endpoint was death within 28 days of hospitalization, while the secondary endpoints were: intubation rate, length of intensive care stay, time between hospitalization and death, between hospitalization and intubation and the factors influencing the survival.

**Patients and methods/materials and methods:** This is a retrospective, monocentric, descriptive and analytical study carried out over a period of 22 months from March 2020 to December 2021 and involving 1069 patients hospitalized in the intensive care unit of our hospital for the management of COVID-19 associated acute respiratory failure, of which, 681 patients have been put on prone position ( PP (prone position) group), and 388 have received standard care (SP (supine position) group). We defined the treatment failure as the proportion of patients who died within 28 days as a major endpoint. To assess the effectiveness of our procedure, we have conducted a univariate analysis by the kaplan meier methode for the survival rate, and a multivariate analysis by cox regression.

**Results:** Mortality in our total sample was 26.28% (15.12% in the PP group and 46.13% in the SP group) The intubation rate on day 28 was 33.20% for the general population, (25.58% in the PP group and 46.16% in the SP group). The major factors influencing survival werethe age, the degry of pulmonary involvement on CT scan and the lactate rate.

**Conclusion:** Awake prone position is significantly associated with a reduction in 28-day mortality. Its use in acute respiratory distress syndrome with and without the context of Covid-19 deserves to be studied in the context of randomized controlled trials.

**Compliance with ethics regulations:** Yes in clinical research.

**Figure 1 (abstract FC-071):**
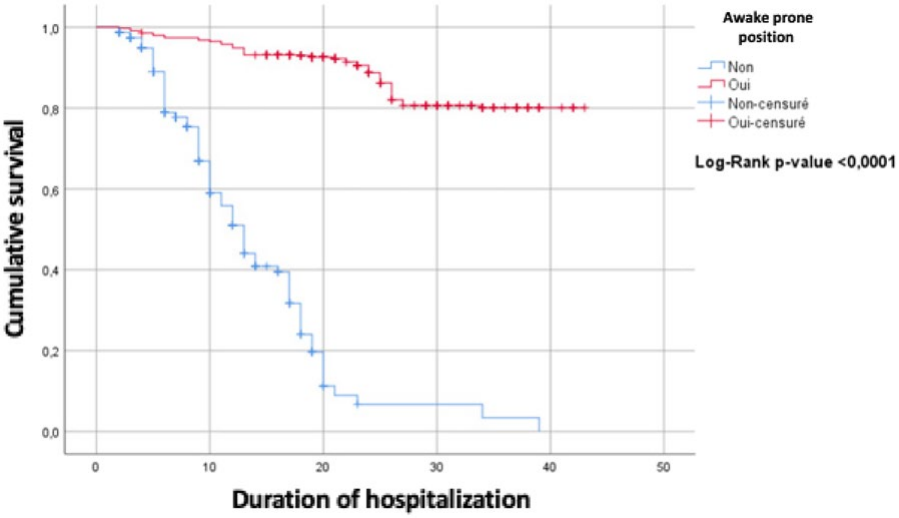
Kaplan meier survival rate

## FC-072 Covid-19 acute respiratory distress syndrome: clinical data, outcomes and ventilatory strategies

### Ines Sedghiani^1^, Malek Cheroufa^1^, Walaa Brahmi^1^, Hamdi Doghri^1^, Imen Zaghdoudi^1^, Nebiha Borsali Falfoul^1^

#### ^1^Hôpital Habib Thameur de Tunis, Tunis, Tunisie

##### **Correspondence:** Ines Sedghiani (sedghiani.ines@gmail.com)

*Annals of Intensive Care *2013, **13(Suppl 1)**:FC-072

**Rationale:** Covid-19 can be complicated by pneumonia of varying severity up to acute respiratory distress syndrome (C-ARDS). The study aim was to describe the clinical, paraclinical and evolutionary data of C-ARDS according to the severity.

**Patients and methods/materials and methods:** We conducted a retrospective study between March 2020 and February 2021 including all patients hospitalized for C-ARDS (according to the Berlin criteria with a positive virological test). We included patients hospitalized via the hospital emergency department for C-ARDS. Patients who died within 48 h of admission and those admitted from another hospital were not included in the study. Patients without C-ARDS and those with ARDS of other etiologies were excluded. We compared the initial clinical and paraclinical data and patients outcomes according to the severity of the ARDS.

**Results:** We enrolled 215 patients with a mean age of 66 ± 12 years and a sex ratio of 1.38. The main comorbidities were hypertension (55.8%) and diabetes (45.1%). The median consultation time was seven days and was shorter in mild C-ARDS (5 days). Oxygen saturation was lower in severe and moderate C-ARDS (80 and 84% respectively) than in mild C-ARDS (88%). Lung injury extension was < 10% in four cases (1.9%), 10 to 25% in 29 cases (13.5%), 25 to 50% in 67 cases (31.2%), 50 to 75% in 83 cases (38.6%) and > 75% in 32 cases (14.9%). C-ARDS was mild in 121 cases (56.3%), moderate in 84 cases (39.1%) and severe in 10 cases (4.7%). Demographic characteristics, comorbidities, initial biological and CT data were comparable between the different severity stages of C-ARDS. Non-invasive ventilation was the most used oxygenation modality; indicated in 204 cases (94.9%) alternating with High-Flow Nasal Cannula (HFNC) oxygen therapy in 30 cases. It was more indicated in mild (98.3%) and moderate (91.7%) ARDS than in severe ARDS (80%). Invasive ventilation was indicated in 28.8% of cases (n = 62) and 80% of severe ARDS; after a median delay of five days [2–10]. Ventilation was performed in the prone position in 81 patients (37.7%), 76 of whom received spontaneous ventilation. ICU length of stay was comparable between the three stages of C-ARDS. The mortality rate was 34.9% (n = 75); higher in severe C-ARDS.

**Conclusion:** Patients with C-ARDS have a mean age of 66 years. Oxygen saturation and lung injury extension define the C-ARDS severity. The most frequent modes needed in C-ARDS are non-invasive ventilation. The use of invasive ventilation and mortality are high in severe forms.

**Compliance with ethics regulations:** Yes in clinical research.

## FC-073 Airway pressure and ventilatory efficiency trends and association to mortality in invasively ventilated COVID-19-related ARDS patients. A retrospective observational cohort study

### Radhouane Toumi^1,2^, Khaoula Meddeb^1,2^, Amir Bedhiafi^1^, Nabil Bouguezzi^1^, Rym Chelbi^1^, Emna Ennouri^1,2^, Imen Ben Saida^1,2^, Mohamed Boussarsar^1,2^

#### ^1^Medical Intensive Care Unit, Farhat Hached University Hospital, Sousse, TUNISIE; ^2^Research Laboratory No. LR12SP09. Heart Failure. Farhat Hached University Hospital, Sousse, Tunisie

##### **Correspondence:** Radhouane Toumi (Radhouane.toumi@gmail.com)

*Annals of Intensive Care *2013, **13(Suppl 1)**:FC-073

**Rationale:** Most severe cases of COVID-19-related ARDS (CARDS) often require invasive mechanical ventilation. CARDS has been described as presenting a dissociation between hypoxemia’s severity and relatively good respiratory mechanics while a minority of severe patients develop decreased pulmonary compliance. The aim of this study was to describe airway pressures and ventilatory efficiency trends in CARDS patients and their association to mortality.

**Patients and methods/materials and methods:** This is a retrospective observational monocentric cohort study carried out in the medical ICU of a University hospital in a low-middle income country, between March 3rd 2020 and December 31st 2021. All consecutive patients with confirmed SARS-CoV-2 infection admitted to the ICU and requiring invasive mechanical ventilation (IMV) were included. Demographic, clinical, physiological and ventilator data were collected. Univariate regression models were used to identify risk factors of mortality.

**Results:** 465 patients were admitted to the ICU for a confirmed COVID-19 infection, 247(53.1%) required IMV. Median [IQR] age was 67 [59–74] years. At admission, 15 (6.1%) patients were already on IMV; SAPS II was 31 [27–37] and P/F ratio was 96 [76.3–139.8] mmHg. At days 1 and 5 of IMV, respectively, plateau pressure was 27[24–28] and 28[26–30] cmH2O; driving pressures, 16[14–19] and 18[15–20] cmH2O; modified oxygenation index, 29.8 ± 13.2 and 24 ± 13.7 cmH2O; mechanical power, 36.0 [29.3–45.2] and 41.6[31.5–49.6] J/min; P/F ratio, 126.3 [90.3–170.8] and 142 [99.3–199.3] mmHg and ventilatory ratio, 2.36 ± 0.89 and 2.55 ± 1.01. At day 1 of IMV, higher plateau and driving pressures, mechanical power and ventilatory ratio were significantly associated to mortality. These were also significantly associated to mortality at day 5 of IMV along with higher modified oxygenation index and lower P/F ratio.

**Conclusion:** In CARDS, it is important to rely on trends of indexes of poor respiratory mechanics properties (plateau pressure, driving pressure, modified oxygenation index and mechanical power) and indexes of poor ventilatory efficiency (oxygen exchange, P/F and carbon-dioxide clearance, ventilatory ratio).

**Compliance with ethics regulations:** Yes in clinical research.

**Figure 1 (abstract FC-073):**
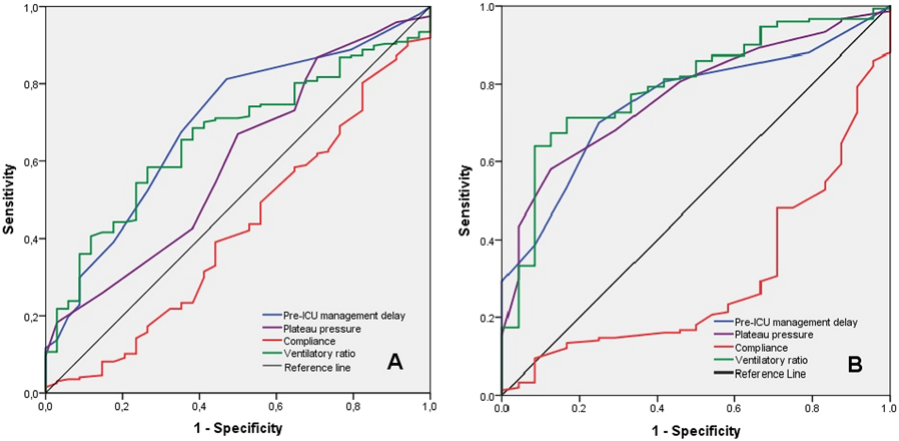
ROC curves at day1 (A) and day5 (B) of airway pressure and ventilatory efficiency in IMV COVID-19 ARDS

## FC-074 Epidemiological, clinical course and outcome of critically ill patients with Covid19 in our hospital: a retrospective cohort study

### Younes Oujidi^1^, Maroua Talhaoui^1^, Inass Arhoun El Haddad^1^, Amine Elmouhib^1^, Ilyass Laaribi^1^, Houssam Bkiyar^1^, Brahim Housni^1^

#### ^1^CHU Mohammed VI OUJDA, Oujda, Maroc

##### **Correspondence:** Inass Arhoun El Haddad (iarhounelhaddad@gmail.com)

*Annals of Intensive Care *2013, **13(Suppl 1)**:FC-074

**Rationale:** COVID-19 first appeared in China in Wuhan, Hubei province, and then rapidly spread worldwide due to the highly contagious nature of this virus. On March 2, 2020, we experienced the first confirmed case of covid-19, and since then, the number of cases has continued to increase exponentially. The management of a case of COVID-19 differs according to the protocol of each country. In this study we were interested in the protocol of our intensive care unit.

**Patients and methods/materials and methods:** This was a single-center descriptive retrospective study of the records of 1381 patients admitted to intensive care at the Oujda University Hospital in Morocco. This work was carried out in the anesthesia and resuscitation department of our hospital. It runs from March 22, 2020, to September 30, 2022, including all patients with confirmed Covid-19 admitted to the intensive care unit during the study period, and excluding the patients who died within 48 h of admission.

**Results:** The median period of observation following hospital admission was 8.52 days for survivors and 10.89 days for non-survivors. The median age of patients was 63.48 years. Men were more represented than women with a percentage of 33% against 31.5%. Two hundred and three (14.6%) patients had obesity (defined as body-mass index [BMI] ≥ 30 kg/m2). Patients presented to hospital a median of 6 days (2–7) after symptom onset. The most common presenting symptoms were shortness of breath, fever, cough, myalgia, and diarrhea. Lymphocytopenia was common. Concentrations of IL-6, high-sensitivity C-reactive protein, ferritin, D-dimer, high-sensitivity troponin, and procalcitonin were elevated in most patients. Invasive mechanical ventilation (IMV) during hospitalization had been necessary in 306 cases (27.22%). Six hundred and ninety-three (61.65%) patients were discharged alive versus 431 patients (38.34%) were died. Analyzing the survival curve as a function of Kaplan–Meier days of hospitalization, we note that survival was increasingly nil in patients who exceed hospitalization days and this constitutes a mortality factor (figure). The mortality rate was higher in patients with a degree of lung parenchymal involvement between [75%-100%] on the chest CT scan on admission compared to patients with a lower degree of parenchymal involvement [50–75%] (p-value = 0.015).

**Conclusion:** Our study shows that age, sex, obesity, as well as certain comorbidities, and biological assessments on admission of COVID-19 patients are among the criteria that make COVID-19 disease serious as well as increase the mortality rate.

**Compliance with ethics regulations:** Yes in clinical research.

## FC-075 Predictive factors of venous thromboembolic events in COVID-19 patients

### Molka Ketata^1^, Ines Sedghiani^1^, Hager Touj^1^, Hamdi Doghri^1^, Amenne Alouini^1^, Yosra Ghali^1^, Imen Zaghdoudi^1^, Nebiha Borsali- Falfoul^1^

#### ^1^Hôpital Habib Thameur de Tunis, Tunis, Tunisie

##### **Correspondence:** Ines Sedghiani (sedghiani.ines@gmail.com)

*Annals of Intensive Care *2013, **13(Suppl 1)**:FC-075

**Rationale:** Since the beginning of the COVID-19, several studies showed a high incidence of venous thromboembolic events (VTE). They are frequently asymptomatic and they have adverse impact on patient prognosis. Our study objective was to identify predictive factors of VTE in COVID-19 patients.

**Patients and methods/materials and methods:** We conducted a retrospective study over a period of one year from March 01, 2020 to February 28, 2021. We included patients hospitalized for COVID-19 confirmed by virological and/or CT scan data. All patients had chest CT scan at admission. We have analyzed risk factors for VTE diagnosed at the first radiological imaging.

**Results:** Three hundred and twenty-three patients were included. They had a mean age of 65 ± 13 years and a sex ratio of 1.31. The common comorbidities were hypertension (52%) and diabetes (44%). Respiratory symptoms were the principal reason for consultation (99.1%). The initial chest CT scan exam revealed an extent of parenchyma lung ≥ 50% in 45.5% of patients. Forty-six patients had a confirmed diagnosis of pulmonary embolism with an incidence of 14.2%. Age over 65 years (p = 0.003; OR = 2; 95% CI [2–10]), female sex (p = 0.03), hypertension (p = 0.01; OR = 2.1), chronic heart failure (p = 0.01; OR = 2.17) and long-term therapeutic anticoagulation (p = 0.05; OR = 1.9) were identified as the risk factors of VTE among COVID-19 patients. On admission, clinical respiratory deterioration signs (p = 0.05) and an oxygen saturation lower than 86% (p = 0.01) were more common in patients with VTE. A Wells score above 2 and a SOFA score above 7 were associated with a higher risk of VTE (p < 10^-3). Patients developing VTE had higher white blood cell count (10.3 vs 8.6 × 10^9/L), platelets count (267 vs 227*10^9/L) and LDH levels (492 vs 360 UI/L) than non-VTE patients. D-dimer > 1600 µg/L was predictive of a higher risk of VTE. Patients with pulmonary embolism had longer hospital stay (11 vs 8 days) and higher in-hospital mortality (49% vs 27.5%).

**Conclusion:** The VTE incidence is high in COVID-19 patients. An age over than 65 years, comorbidities and a D-dimer value greater than 1600 µg/L were significantly associated with a higher risk of VTE among COVID-19 patients.

**Compliance with ethics regulations:** Yes in clinical research.

## FC-076 Is age a good factor to deny intubation in COVID-19 patients?

### Nabil Bouguezzi^1,2^, Imen Ben Saida^1,2^, Radhouane Toumi^1,2^, Rihab Rajah^1^, Rym Chelbi^1,2^, Azer Yaacoub^1,2^, Imen Belhouchet^1,2^, Nour Belaaj^1^, Khaoula Meddeb^1,2^, Mohamed Boussarsar^1,2^

#### ^1^University of Sousse, Faculty of Medicine of Sousse, Sousse, TUNISIE; ^2^Farhat Hached University Hospital, Medical Intensive Care Unit, Research Laboratory “Heart Failure”, LR12SP09, 4000, Sousse, Tunisia., Sousse, Tunisie

##### **Correspondence:** Nabil Bouguezzi (dr_nabil@live.fr)

*Annals of Intensive Care *2013, **13(Suppl 1)**:FC-076

**Rationale:** The increasing age in the industrialized countries places significant demands on intensive care unit (ICU) resources and triggers debates about end-of-life care for the elderly. Actual data are leading intensivists to wonder whether breathing tubes should be used as often as they are. We aimed to investigate the patterns of 28-day mortality, by age group, among mechanically ventilated critically ill COVID-19 patients.

**Patients and methods/materials and methods:** It is a retrospective observational study conducted from March 2020 to December 2021, in a Medical ICU. Information regarding demographic, clinical characteristics and outcomes of Critically Ill COVID-19 patients was obtained from medical records. The population was divided into 3 groups according to age; < 60 years old (G1), 60 to 80 years old (G2), and > = 80 years old (G3). We evaluated mortality rates between age groups. Kaplan Meier curves were used to describe 28-day mortality.

**Results:** Among the465 COVID-19 patients admitted in the study period, we analyzed 244 patients who had undergone IMV. They were mostly male patients. Two hunded and twelve patients (85.8%) had at least one comorbidity. The mean age was 65.3 ± 11.9. Patients’ presentation on admission was severe, all patients presented with a severe to critical COVID-19, of which 160 (64.8%) had a severe ARDS; median P/F ratio at admission was at 96 [76.3–139.8] mmHg. The mortality rate was high at 84.4%. We had 62, 163 and 19 patients in G1, G2 and G3, respectively. By age group, the mortality rates were 69.4%, 89% and 100% in patients in G1, G2 and G3, respectively. There was no significant difference in length of stay between different groups (9 [5–14], 9 [6–15] and 10 [8–14] days in G1, G2 and G3, respectively). Kaplan- Meier analyses of survival of patients under IMV are displayed in Figure 1.

**Conclusion:** Patients aged 80 and over seem to be a reasonable situation to discuss for ICU admission denial or at least for intubation denial.

**Compliance with ethics regulations:** Yes in clinical research.

**Figure 1 (abstract FC-076):**
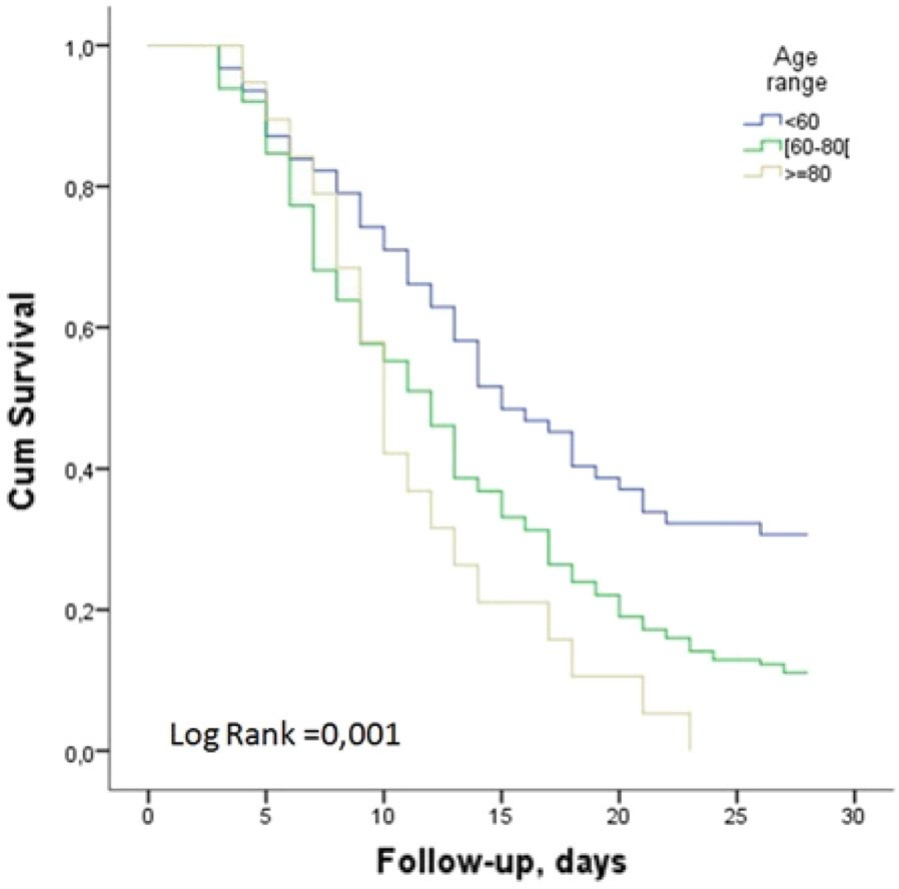
Kaplan–Meier survival curves (28-day mortality) according to age

## FC-077 Prognosis of late intubation for COVID-19 acute hypoxic respiratory failure (AHRF), a retrospective multicentric observational study from the Outcomerea Network

### Mathilde Phillips-Houlbracq^1^, Claire Dupuis^2,12^, Karl Bitar^1^, Siami Shidasp^4^, Yves Cohen^5^, Virginie Laurent^6^, Bruno Mourvillier^7^, Jean Reignier^8^, Dany Goldgran?toledano ^9^, Carole Schwebel ^10^, Stéphane Ruckly ^11^, Etienne De Montmollin^3,12^, Niccolò Buetti^11,12^, Charles Cerf^1^, Jean- François Timsit^3,12^, Mathilde Neuville^1^

#### ^1^Hôpital Foch, Suresnes, France; ^2^Medical Intensive Care Unit, Gabriel Montpied University Hospital, Clermont-Ferrand, France, Clermont-Ferrand, France; ^3^Medical and Infectious Diseases Intensive Care Unit, Bichat-Claude Bernard Hospital, Paris, France; ^4^General Intensive Care Unit, Sud Essonne Hospital, Etampes, France; ^5^Intensive Care Unit, University Hospital Avicenne, AP-HP, Bobigny, France; ^6^Polyvalent Intensive Care Unit, André Mignot Hospital, Le Chesnay, France; ^7^Medical Intensive Care Unit, University Hospital of Reims, Reims, France; ^8^Medical Intensive Care Unit, University Hospital of Nantes, Nantes, France; ^9^Medical and Surgical Intensive Care, Montfermeil Hospital, Montfermeil, France; ^10^Medical Intensive Care Unit, University Hospital Grenoble?Alpes, Grenoble, France; ^11^Infection Control Program and WHO Collaborating Centre on Patient Safety, Faculty of Medicine, University of Geneva Hospitals, Geneve, Suisse; ^12^Université de Paris, UMR 1137, IAME, APHP, Paris, France

##### **Correspondence:** Mathilde Phillips-Houlbracq (mathilde.phillips@gmail.com)

*Annals of Intensive Care *2013, **13(Suppl 1)**:FC-077

**Rationale:** Among severe Sars-CoV2 pneumoniae, 20–40% are admitted in Intensive Care Unit for Acute Hypoxemic Respiratory Failure (AHRF) to receive non-invasive oxygen-support (NI-OS) and/or mechanical ventilation. The timing of intubation for COVID-AHRF remained controversial. The aim of this study was to compare day-60 mortality between patients intubated 5 days after the hospitalization in ICU and those with similar criteria of AHRF after day 5, maintained under NI-OS.

**Patients and methods/materials and methods:** This observational retrospective study was performed with data from the French prospective multicenter (n = 11 ICUs) OutcomeReaTM database from February, 1^st^ 2020 to May, 30th 2022. Patients with HFAR were included if they had an ICU stay of more than 5 days without intubation (under NIOS: CPAP, HFNC, NIV). The 60-day mortality rate of intubated patients after day 5 were compared to non-intubated patients (under NIOS) who harbored the same gravity of AHRF (FiO2 of 80% and having a PaO2 of less than 80 mmHg) at the same length of stay.

**Results:** From February 2020 to August 2022, 1206 patients were admitted for COVID-19 pneumonia in the 11 French ICUs of OutcomeRea TM database. 387 patients were still in ICU and non-mechanically ventilated at day 5. Patients were mainly men (70%), median age of 66.4 years and at least one comorbidity in 70% cases. 73,6% (n = 285) received steroids during hospitalization. 25.8% were finally intubated during stay of intensive care and the mortality rate was 26.8% at day 60 among the 387 patients. Patients intubated after day 10 (n = 28) had a poor prognosis compared to those still under NIOS after day 10 (n = 10) with 60-day mortalityrespectively 89.3% and 60% (p = 0.04), HR 2.43 (0.96–6.11) after adjustment for BMI and age.

**Conclusion:** These data suggest a poorer prognosis for patients intubated after 10 days under NIOS for COVID AHRF. In this context, invasive ventilation should be avoided.

**Compliance with ethics regulations:** Yes in clinical research.

## FC-078 Impact of treatment with high-flow nasal oxygen therapy during the first three waves of Covid-19 pandemic: a French monocentric retrospective study

### Tifany Vatignez^1^, Benjamin Popoff^1^, Grégoire Jolly^1^, Zoé Demailly^1^, Christian Caillard^1^, Diane Ducloux^1^, Déborah Boyer^1^, Dorothée Carpentier^1^, Gaëtan Beduneau^1^, Steven Grangé^1^, Maximilien Grall^1^, Fabienne Tamion^1^, Christophe Girault^1^

#### ^1^CHU Charles Nicolle, Rouen, France

##### **Correspondence:** Tifany Vatignez (tifany.vatignez@hotmail.fr)

*Annals of Intensive Care *2013, **13(Suppl 1)**:FC-078

**Rationale:** The COVID-19 pandemic provoked a surge of intensive care unit (ICU) patients with profound hypoxemia. Due to the specificities of this pneumonia and risk of viral aerosolization, the different oxygenation techniques have been debated. We used, however, high flow nasal oxygen therapy (HFNO) from the start of the pandemic in our ICU. Only few studies have evaluated the impact of the oxygenation strategy on the outcome of COVID-19 ICU patients during the different waves of the pandemic.

**Patients and methods/materials and methods:** We conducted a single center retrospective study to assess the impact of HFNO use in COVID-19 patients with severe hypoxemic acute respiratory failure (ARF) during the first 3 waves of the pandemic. All COVID-19 ARF patients admitted in our ICU between March 2020 and May 2021 were evaluated for their clinical characteristics, respiratory management and outcome. We also assessed the prognosis of patients according to the HFNO duration before intubation (HFNO late failure: > 48 h and FiO2 ≥ 80% and flow rate ≥ 60 L/min).

**Results:** 198 patients were included during the 3 first waves: 56, 85 and 57 patients, respectively. Despite similar clinical characteristics on admission, the use of HFNO increased significantly between the 3 waves (62.5%, 96.5%, 98.2% respectively; p < 0.001) with a trend toward for a higher HFNO success rate (46.4%, 65.9% and 63.2%; p = 0.057). When HFNO failed, intubation occurred later during the 3rd wave compared to the ICU admission (p < 0.001), duration of HFNO before intubation (1.8, 3.0 and 5.2 days, respectively; p = 0.015) and with more severe underlying hypoxemia (PaO2/FiO2 ratio; p = 0.012). The length of stay and overall ICU mortality were similar between the 3 waves. Finally, all waves combined, the intubation time (early n = 19, intermediate n = 24, late n = 31) had no deleterious effect on patients’ outcomes (duration of ventilation, length of stay and ICU mortality).

**Conclusion:** Our study confirms that HFNO is a useful oxygenation strategy for the management of severe COVID-19 ARF patients. HFNO can avoid intubation in satisfactory respiratory conditions without compromising outcomes, including in patients with prolonged HFNO and late failure.

**Compliance with ethics regulations:** Yes in clinical research.

**Figure 1 (abstract FC-078):**
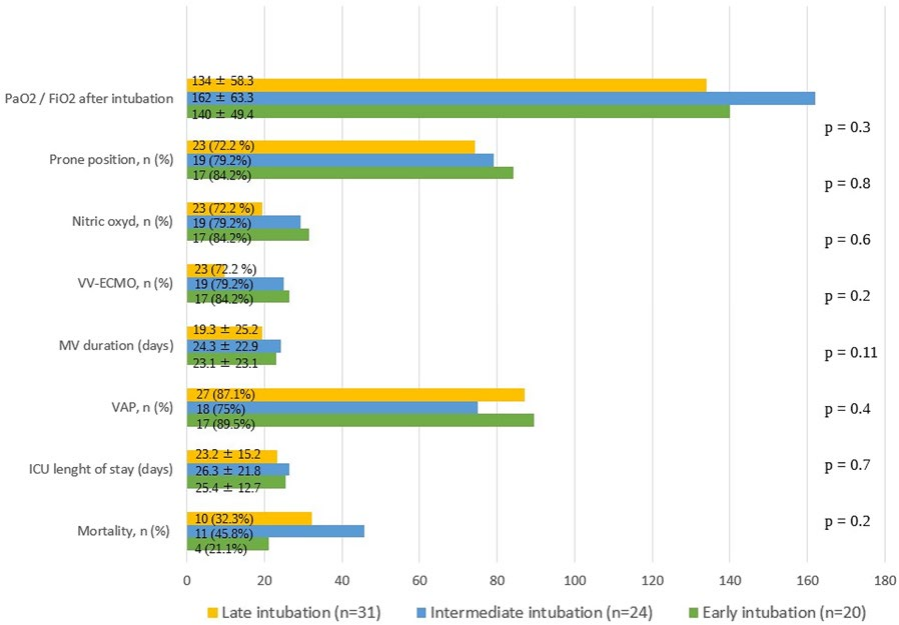
Impact of delay intubation on patients outcomes

## FC-079 Predicting NIV failure in hypoxemic COVID19 patients: the utility of the HACOR score and the updated HACOR score

### Yosri Ben Ali^1^, Oussama Saadaoui^1^, Hend Zorgati^1^, Dhouha Ben Braiek^1^, Sourour Bel Haj Youssef^1^, Rihab Boubtane^1^, Rahma Ben Jazia^2^, Amani Kacem ^2^, Jihene Ayachi^1^

#### ^1^Medical Intensive Care Unit, Ibn El Jazzar University Hospital, Kairouan, Tunisie; ^2^Pulmonology Department, Ibn El Jazzar University Hospital, Kairouan, Tunisie

##### **Correspondence:** Jihene Ayachi (ayachijihen@gmail.com)

*Annals of Intensive Care *2013, **13(Suppl 1)**:FC-079

**Rationale:** Non-invasive ventilation (NIV) has been largely applied to treat COVID-19 patients with moderate to severe acute respiratory failure (ARF). HACOR score has been used to predict non-invasive ventilation (NIV) failure. Updated HACOR score was proposed to evaluate NIV outcomes. This study aimed to analyze the ability of the HACOR and updated-HACOR score to predict NIV failure of critically ill patients admitted for COVID-19 pneumonia.

**Patients and methods/materials and methods:** A retrospective observational study including patients receiving NIV with confirmed COVID19, admitted in a 9-bed medical intensive care unit from September 2020 to December 2021. Scores were calculated one hour after NIV initiation. HACOR score associates heart rate, acidosis, consciousness, oxygenation and respiratory rate. Updated HACOR score associates HACOR score, SOFA score, pneumonia, cardiogenic pulmonary oedema, ARDS, immunosuppression and septic shock. Patients with updated-HACOR scores were classified into ≤ 7, 7.5–10.5, 11–14, and > 14. A receiver operating characteristic (ROC) curve was used to evaluate discrimination of HACOR and Updated-HACOR score for NIV failure.

**Results:** During the study period, 47 patients required NIV (first line and after high flow nasal cannula failure). Main characteristics were mean age 57.1 ± 12.3 years, men predominance (53.2%), SAPS II 27.8 ± 8.9, P/F ratio 109 ± 54 mmHg. The most common comorbidities were obesity (53.2%), diabetes (34%) and hypertension (31.9%). Mean Pressure support was 16 ± 2 cmH2O, mean PEEP 5.8 ± 0.8 cmH2O, median Fio2 78 and mean NIV use 4 ± 3.9 days. HACOR score ≥ 5 was found in 36 (76.6%) patients, and updated HACOR score ≥ 11 points in 42 (89.4%) patients. Updated HACOR scores ≤ 7, 7.5–10.5,11–14, and > 14 one hour after initialization of NIV were found in 2.1%, 8.5%, 42.6%, and 46.8% of cases respectively. Thirty-three patients (70.2%) experienced NIV failure, among them 29 (87.9%) patients have HACOR ≥ 5 and 97% of them have an updated HACOR score ≥ 11 with (p-value at 0.005 and 0.009 respectively). The area under the curve (AUC) for predicting NIV failure was 0.74 (95% CI,0.57–0.91) when it was tested with HACOR score and 0.76 (95% CI,0.61–0.91) when updated HACOR score was used. The AUC in the range of 0.7–0.9 was interpreted as moderate diagnostic accuracy.

**Conclusion:** The HACOR and the updated HACOR scores are clinically useful bedside tools for the prediction of NIV failure in COVID19 pneumonia with hypoxemic respiratory failure. They could be helpful in decision-making when NIV is used.

**Compliance with ethics regulations:** Yes in clinical research.

## FC-081 Effects of continuous anterior chest compression (CACC) and comparison with prone position in the ARDS « The StrapVent Study»

### Mohamed Ahmed Boujelben^1^, Samuel Tuffet^1^, Elsa Moncomble^1^, Anne Fleur Haudebourg^1^, Pascale Labedade^1^, Keyvan Razazi^1^, Nicolas De Prost^1^, Armand Mekontso Dessap^1^, Guillaume Carteaux^1^

#### ^1^CHU Henri Mondor, Créteil, France

##### **Correspondence:** Mohamed Ahmed Boujelben (m.ahmedboujelben@gmail.com)

*Annals of Intensive Care *2013, **13(Suppl 1)**:FC-081

**Rationale:** Previous studies have shown that continuous anterior chest compression (CACC) can induce a paradoxical decrease in plateau pressure (Pplat) in persistent ARDS with severe impairment of the respiratory mechanics by limiting overdistension. This study aimed to assess the physiological effects of CACC in the early phase of ARDS and to compare them to prone position.

**Patients and methods/materials and methods:** This was a prospective single-center physiologic study. The patients with moderate-to-severe ARDS who were placed in prone position by the attending physician were eligible. The regional respiratory mechanics and distribution of ventilation were explored by esophageal catheter and electrical impedance tomography (Enlight 800, Timpel) during four consecutive steps: prone position (PP), supine position (SP1), supine position with CACC applying a pressure opposite the sternum equal to that observed during prone position, and supine position again (SP2). The primary endpoint was the end-inspiratory transpulmonary pressure derived from elastance ratio (PLinsp). Secondary endpoints included regional lung compliances.

**Results:** We present the results of the first seven patients included in this study. The median age was 60 years [57–70] and the median BMI was 33 kg/m2 [26–39]. All patients had pneumonia-related ARDS. The median tidal volume was 5.9 mL/kg [5.9–6.1] and the median PEEP 12 cm H2O [10–15]. PaO2/FiO2 was 96 mmHg [77–113] and respiratory system compliance was 31.4 ml/cm H2O [31.2–39.3]. The pressure exerted by the body weight at the sternum during PP was 72 cmH2O [60–86]. During CACC using the same pressure, two physiological responses were observed: either an increase in Pplat (non-responders, n = 3), or a paradoxical decrease in Pplat (responders, n = 4). In all responders, PLinsp decreased during CACC and both anterior and posterior regional lung compliances increased, suggesting both a reduction in pre-existing anterior overdistension and an improvement in posterior recruitment. The opposite was observed in non-responders. The effects of CACC on Pplat, PLinsp and regional lung compliances appeared comparable to the effects of PP according to the patients’ profiles.

**Conclusion:** The effects of CACC on PLinsp and regional ventilation distribution were comparable to those observed during prone position and may depend on the pre-existing level of overdistension.

**Compliance with ethics regulations:** Yes in clinical research.

**Figure 1 (abstract FC-081):**
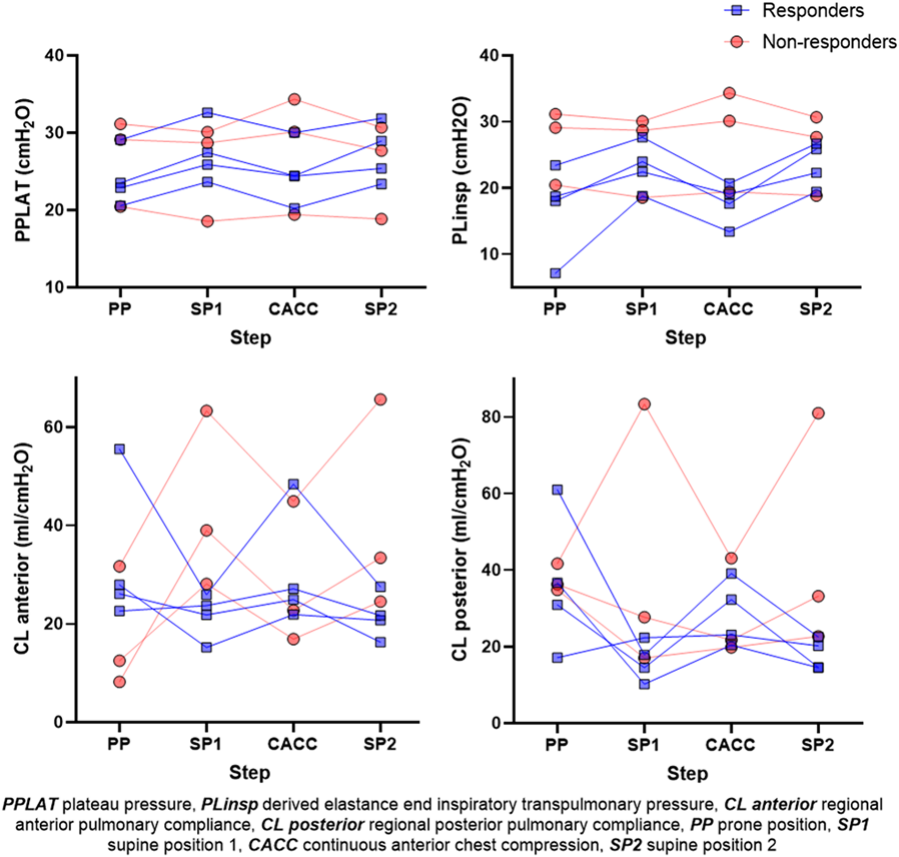
Evolution of PPLAT, PLinsp and regional lung compliances according to CACC response: responder patients are those in whom the PPLAT decreased during CACC

## FC-082 Moving beyond the lines: lung ultrasound pixel-wise computer-assisted analysis for critically ill patients

### Orphée Faucoz^2^, Denis Standarovski^2^, Amazigh Aguersif^1^, Sihem Bouharaoua^1^, Benjamine Sarton^1,3^, Stein Silva^1,3^

#### ^1^Critical Care Unit. University Teaching Hospital of Purpan, Toulouse, France; ^2^French National Center for Spatial Studies (CNES). Calculation and Data Engineering Department, Toulouse, France; ^3^Toulouse NeuroImaging Center, Toulouse University, UMR INSERM/UPS 1214, Toulouse, France

##### **Correspondence:** Amazigh Aguersif (aguersif.a@chu-toulouse.fr)

*Annals of Intensive Care *2013, **13(Suppl 1)**:FC-082

**Rationale:** Lung ultrasonography (LUS) has become an essential component of the evaluation and clinical management of patients admitted to the intensive care unit (ICU). The interpretation of LUS artifacts (A- and B-patterns), analysis of the pleura, and the visualization of real images (C pattern) have demonstrated usefulness for the differential diagnoses of acute respiratory failure (ARF). However, current methods are non-quantitative and have important drawbacks deriving from visually guided assessment of LUS data. Nevertheless, it is commonly acknowledged that these methods, which are based only on medical experts’ analysis, can be time-consuming, are user-dependent, and hold the risk of leading to oversimplified and potentially harmful diagnosis algorithms, particularly in the complex pathophysiological setting of critically ill patients. To accurately cope with these issues, computer vision approaches, built upon machine learning (ML) algorithms have been recently proposed. These methods have potential to provide new computer-aided diagnosis for LUS data that could transform the way in which ICU practitioners assess and manage critically ill patients. However, it is worth noting that the data available in this field have demonstrated variable classifier’s accuracy and was exclusively limited to the analysis of B-lines. We followed a knowledge transfer approach from satellite to medical imaging based on semantic segmentation and signal processing. In order to do this, we designed a proof-of-concept study built upon one of the largest LUS datasets from severe COVID-19 patients reported to date.

**Patients and methods/materials and methods:** We prospectively recruited adult COVID-19 patients who were in ARF (pulse oximetry < 90% while breathing room air or respiratory rate > or = 30 breaths/min) at hospital admission. Overall, 5000 LUS frames from 78 patients affected by COVID-19 with different degree of severity were gathered and labeled.

**Results:** We provided an automatic pixel-wise classifier, which was able to accurately identify for the first time, all the main LUS patterns that can be observed in this setting (overall Area Under the Curve, AUC = 0.97; pleural line AUC = 0.99; A-pattern AUC = 0.96; B pattern AUC = 0.97; C-pattern AUC = 0.95).

**Conclusion:** The eventual integration of this model into ultrasound hardware seems plausible as a method to ultimately improve outcomes through a combined use of LUS with other components of critical care ultrasonography to yield a more comprehensive and accurate evaluation of the critically ill patient at point of care.

**Compliance with ethics regulations:** Yes in clinical research.

**Figure 1 (abstract FC-082):**
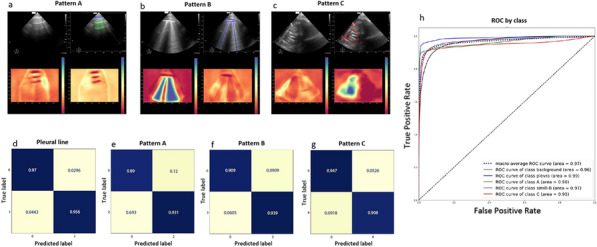
Automatic pixel-wise LUS image classification based on semantic segmentation

## FC-083 Impact of prone position on the oxygenation of COVID-19 patients with acute respiratory distress syndrome

### Iyed Maatouk^1^, Hamza Ben Hassine ^1^, Asma Zarrouk^1^, Saoussen Ben Abdallah^1^, Hedia Ben Ahmed^1^, Abir Chihaoui^1^, Oussema Saadaoui^1^, Manal Lahmar^1^, Wiem Nouira^1^, Zeineb Hammouda^1^, Fahmi Dachraoui^1^, Fekri Abroug^1^, Lamia Ouanes Besbes^1^

#### ^1^University Hospital Fattouma Bourguiba of Monastir, Monastir, Tunisie

##### **Correspondence:** Iyed Matouk (maatouk.yed@gmail.com)

*Annals of Intensive Care *2013, **13(Suppl 1)**:FC-083

**Rationale:** Coronavirus disease 2019 (COVID-19) is considered as a global health emergency. It can cause an acute respiratory distress syndrome (ARDS) requiring mechanical ventilation. The prone position is one of the methods used in the management of patients with this syndrome. Our study aimed to determine the impact of the prone position on the oxygenation of COVID-19 patients with ARDS.

**Patients and methods/materials and methods:** We conducted a retrospective study between March 2020 and May 2022 in the Intensive Care Unit of the University Hospital Fattouma Bourguiba of Monastir (Tunisia). COVID-19 patients who responded to the clinical criteria of the Berlin definition of ARDS and requiring mechanical ventilation were included. Data analysis was performed using the Statistical Package for Social Sciences (SPSS) version 21.0.

**Results:** In total, 182 mechanically ventilated patients diagnosed with COVID-19 ARDS were enrolled in the study. Most patients were male (67.6%). The mean age was 63.5 ± 11 years. Obesity was found in 44.9% of the patients. The median length of stay was 10.5 (Interquartile range (IQR): 5.7–16) days. The median SAPS II, APACHE II and SOFA score were 34 (IQR: 28- 4), 11 (IQR: 9–15), and 4 (IQR: 3–4), respectively. Patients were intubated after a median duration of 2 days (IQR: 1–5) after admission. Mean compliance was 25.5 ± 9.8 ml/cmH2O. The median number of prone position sessions was 3 (IQR: 1.5–6). PaO2/FiO2 ratio was significantly improved from 103.7 ± 44.9 mmHg in supine position to 126.61 ± 59.7 mmHg in prone position (p < 10^-3).

**Conclusion:** Our study shows that the change from supine to prone position improves oxygenation among COVID-19 ARDS patients. Further studies should be conducted on a larger number of patients in order to better guide intensivists in the treatment of ARDS due to COVID-19.

**Compliance with ethics regulations:** Yes in clinical research.

## FC-084 Acute respiratory failure in the elderly in the emergency department: Epidemiological, clinical and evolutionary particularities

### Ines Sdiri^1^, Hamdi Hamdene Doghri^1^, Oussema Ben Souissi^1^, Ines Sedghiani^1^, Wafa Homrani ^1^, Imen Zaghdoudi^1^, Nebiha Falfoul Borsali^1^

#### ^1^Hôpital habib thameur, Tunis, Tunisie

##### **Correspondence:** Ines Sdiri (sdiri.ynes@gmail.com)

*Annals of Intensive Care *2013, **13(Suppl 1)**:FC-084

**Rationale:** Acute respiratory failure is a frequent cause of hospitalization in the elderly. Symptoms are often atypical and responsible for a delay in diagnosis. The aim of our study was to describe the epidemiological, clinical and evolutionary particularities of acute respiratory failure in the elderly who consulted in the emergency room.

**Patients and methods/materials and methods:** It was a retrospective and descriptive study including elderly subjects admitted to the emergency and medical ICU between January 1, 2022 and August 1, 2022. We collected clinical, paraclinical, therapeutic and evolutionary data. We defined elderly age range over 65 years.

**Results:** We canalyzed 72 patients admitted for acute respiratory failure. The median age was 77.5 years [66–100]. The sex ratio was 1.2. We noted 40 (55.6%) patients with chronic hypertension. Nineteen (26.3%) patients had chronic heart failure, 10 (13.9%) had chronic obstructive pulmonary disease, 11 (15.3%)had chronic renal failure, 5 (6.9%) patients were on hemodialysis, and 9 (12.5%) of whom had a history of stroke. The reasons for consultation were dyspnea in 48 patients (66.7%), altered neurological status in 7 cases (9.7%) and asthenia in 17 cases (23.6%). The acute respiratory failure etiology was COVID-19 infection in 63 patients (87.5%) associated with a bacterial superinfection in 4 cases, a pulmonary embolism in 8 cases, a left heart failure in 17 cases and an acute coronary syndrome in 10 cases. Left heart failure was found in 5 patients (7%) and bacterial pneumonia in 4 patients (5.5%). Twelve patients (16.7%) required Continuous Positive Airway Pressure (CPAP), 18 (25%) received non-invasive ventilation in pressure support mode, 8 (11.7%) were put on high flow oxygen, and 7 (9.7%) were intubated and ventilated in controlled mode. The most common complications were acute renal failure in 12 patients (16.7%) and upper gastrointestinal bleeding in 4 cases (5.6%). The evolution was fatal in 23 cases (31.9%). Three patients (4.2%) were discharged on long-term oxygen at home.

**Conclusion:** Acute respiratory failure in the elderly remains a serious pathology with a high rate of mortality. Its main etiology was Covid-19 during the pandemic. A specific emergency department for geriatric patients must be evaluated to optimize the management of these patients who suffer from several chronic diseases.

**Compliance with ethics regulations:** Yes in clinical research.

## FC-085 Non-Rebreather Mask: No More Effective Than Nasal Cannula?

### Rida Cheikh Youssef^1^, Sharam Machayekhi^1^, Lauren Bergeret ^4^, Jean Roeseler^2^, Gregory Cuvelier ^3^, Frédéric Duprez^1^

#### ^1^Epicura Hospital, Hornu, Belgique; ^2^UCL—Cliniques universitaires Saint-Luc, Bruxelles, BELGIQUE; ^3^Laboratoire du mouvement Condorcet, Tournai, Belgique; ^4^Unité de Recherche et Innovation Condorcet Epicura, Hornu, Belgique

##### **Correspondence:** Rida Cheikh Youssef (rida.cheikhyoussef@gmail.com)

*Annals of Intensive Care *2013, **13(Suppl 1)**:FC-085

**Rationale:** Non-rebreather face masks (NRM) are used in patients with profound hypoxemia, they have a reputation for delivering very high FiO2. (1) However, for some authors, these masks provide the same FiO2 as nasal cannulas, i.e., an increase of 3% of FiO2 per litre of O2 administered. (2) The purpose of the study was to bench analyze the FiO2 delivered by the NRM at different oxygen flow rates, and to verify if the increase in FiO2 is equal to 3% per litre of O2 administered.

**Patients and methods/materials and methods:** On a bench study, the FiO2 delivered by a total NRM was analysed with an adult lung model (Michigan Instrument Inc., Grand Rapids, MI), driven by a mechanical ventilator (Servo I™ Maquet) set to volume control mode. Resistive and elastic characteristics of the test lung were set at 5 cm H2O/L/sec and 0.06 L/cm H2O, respectively. Data acquisition was performed via the Labscribe 3TM software (Iworx®, United States) after a stabilization period. The parameters were modified as follows: O2 flow: 10 and 15 L/min, minute ventilation (MV) from 6 to 21 L/min, Ti/Ttot: 0.33 and 0.25.

**Results:** The agreement between calculated and measured FiO2 (CMFiO2) was expressed as proposed by Bland and Altman. Thus, the bias and limits of agreement are reported (95% CI for the difference between measurements). Next, an analysis of calculated and measured FiO2 as a function of MV was performed. Results: When MV increases, both the FiO2 and the difference between CMFiO2 decreases. The agreement between CMFiO2 was: Bias 1.46/limits of agreement from -13.89 to 16.82. The median value of FiO2 increase by litre of oxygen administered is equal to 3.2% (IQR: 2.7 to 3.6). There is not a statistically significant difference between CMFiO2 (p = 0.331). When the O2 flow rate is equal to 10 L/min, the "3% rule” is reached for MV of 15 L/min, while for an O2 flow rate of 15 L/min, the "3% rule” is reached for MV of 10 L/min (Figure 1).

**Conclusion:** In this study, the total NRM never delivered a FiO2 close to 100%. The "3% rule” was statistically verified. However, the limits of agreement between the FiO2 obtained with the "3% rule" calculation and those obtained at the bench are very large. We conclude that the calculation of theoretical FiO2 with NRM can be highly variable depending on O2 flow and MV.

**Reference 1**

Wagstaff T, Soni N. Performance of six types of oxygen delivery devices at varying respiratory rates. Anaesthesia. May 2007;62(5):492–503.

**Reference 2**

Frat JP, Thille AW, Mercat A, Girault C, Ragot S, Perbet S, et al. High-flow oxygen through nasal cannula in acute hypoxemic respiratory failure. N Engl J Med. June 4, 2015;372(23):2185–96.

**Compliance with ethics regulations:** N/A.

**Figure 1 (abstract FC-085):**
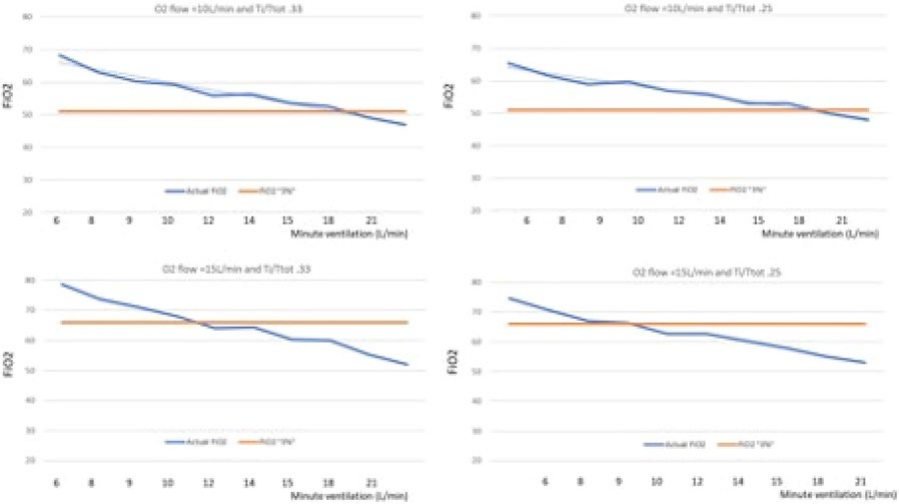
FiO2 comparison between actual FiO2 delivered by NRM and Vincent Formula prediction

## FC-086 Impact of an automated real time waveform guided algorithm (IntelliSync + ®) on patient-ventilator synchrony during non-invasive ventilation—A bench study

### Yann Renaud^1^, Jean-Daniel Chiche^1^, Lise Piquilloud^1^

#### ^1^Centre Hospitalier Universitaire Vaudois, Lausanne, Suisse

##### **Correspondence:** Yann Renaud (yann_r@bluewin.ch)

*Annals of Intensive Care *2013, **13(Suppl 1)**:FC-086

**Rationale:** Patient-ventilator synchrony refers to the matching between the ventilator pressurization and the patient’s neural inspiratory command. During non-invasive ventilation (NIV) synchrony is difficult to achieve because of inherent leaks, even with automatic leak compensation. Automated setting adaptation using algorithms designed to perform real-time analysis of airway pressure and flow-time curves to detect asynchrony is now available (IntelliSync + ®, Hamilton, Bonaduz, Switzerland). This bench study aim was to evaluate the benefits of IntelliSync + ® on patient-ventilator synchrony in case of obstructive respiratory mechanics.

**Patients and methods/materials and methods:** To simulate inspiratory effort, one chamber of a two-chamber Michigan test lung (Michigan Instruments, Grand Rapids, USA) was connected to a driver ventilator (P0.1 = 5,6 ± 0,2 cmH2O). The second chamber was connected to a Hamitlon-S1 ventilator (Hamilton medical, Bonaduz, Switzerland) equipped with IntelliSync + ® and set in pressure support (PS) with the NIV mode activated. Obstructive respiratory mechanics was obtained by placing a resistance in the ventilator circuit (R = 24,6 ± 0,7 cmH2O/L/sec). Continuous leak was created in the circuit to reveal triggering asynchronies, whereas cycling-off asynchronies were revealed by creating a leak limited to inspiration (leak flow of 20 L/min). PS was set at 14cmH2O. Synchrony was assessed at steady state (number of asynchronies and measurement of trigger delay and inspiratory time in excess). Pressurization capacity was assessed using the triggering pressure–time product (PTP) and the PTP300 and 500. Measurements were made without IntelliSync + ® (A), with IntelliSync + ® activated during inspiration (B), cycling off (C) and both (D). Results of 5 consecutive ventilatory cycles are reported as medians, 1st and 3rd quartile. Comparisons were performed with one-way ANOVA on ranks (Bonferroni correction for pairwise comparisons).

**Results:** The results are illustrated in the following table.

**Conclusion:** In this bench study, IntelliSync + ® shortened the trigger delay, improved expiratory synchrony and optimized pressurization capacity in a model of an obstructive patient in respiratory distress ventilated with NIV.

**Compliance with ethics regulations:** N/A.

**Table 1 (abstract FC-086):**
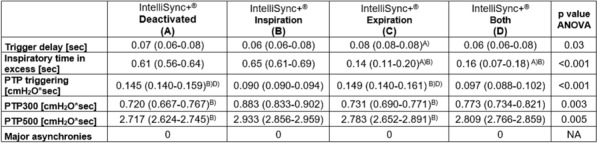
Results are expressed as medians, 1st and 3rd quatiles of 5 consecutive ventilatory cycles at steady state. A) p < 0.05 vs A. B) p < 0.05 vs B. D) p < 0.05 vs D

## FC-087 Technical performances of recent ventilators during noninvasive ventilation: a bench study

### Christian Caillard^1,2,3^, Emeline Fresnel^4^, Elise Artaud-Macari^2,3^, Antoine Cuvelier^2,3^, Fabienne Tamion^2^, Maxime Patout^5^, Christophe Girault^2,3^

#### ^1^CHI Elbeuf-Louviers-Val de Reuil, Saint Aubin Lès Elbeuf, France; ^2^CHU Rouen, Rouen, France; ^3^GRHVN UR 3830, Rouen, France; ^4^Kernel Biomedical, Bois-Guillaume, France; ^5^CHU Pitié Salpêtrière, Paris, France

##### **Correspondence:** Christian Caillard (cricaillard@orange.fr)

*Annals of Intensive Care *2013, **13(Suppl 1)**:FC-087

**Rationale:** A large choice of recent ventilators, dedicated or not, are available to perform noninvasive ventilation (NIV) in intensive care units (ICU). We conducted a bench study to compare the technical performances of different ventilators for NIV, and particularly their impact on patient-ventilator asynchrony (PVA) according to different respiratory conditions.

**Patients and methods/materials and methods:** Ventilators have been evaluated on a previously validated bench test for NIV consisting of a 3D mannequin head connected to an artificial lung model via. a dual-limb circuit with a non vented facial mask: Five ICU ventilators with the NIV algorithm activated, 2 dedicated NIV ventilators and 1 transport ventilator were tested according to 3 patient profiles simulated with the lung model (normal, obstructive, and restrictive lung) and different respiratory conditions: 3 levels of non-intentional leak (0, 15 and 30L/min), 2 levels of pressure support (8 and 14 cmH2O) and 2 respiratory rates (15 and 25 cycles/min).

**Results:** Median total leak was not different between the ventilators (p = 0,09). The asynchrony index (AI), defined as the [number of asynchrony events/(ventilator cycles + wasted efforts) × 100], was higher with ICU ventilators than with dedicated NIV ventilators (4% [0;76] vs 0% [0–15]; p < 0.05) (Figure 1). The AI was different between all ventilators (p < 0.001). The AI was higher with ICU ventilators for the normal and restrictive profiles as compared to dedicated NIV and transport ventilators (p < 0.01) but not different between ventilators for the obstructive profile. The overall median AI was correlated with the mean total leaks (p < 0.01), but several ventilators had a better adaptation to an increase of the level of leaks, the AI increasing less than for others (p < 0.01). Auto-triggering represented 43% of overall PVAs. AI was higher with a pressure support of 14cmH2O than 8cmH2O, there was no difference of AI with a respiratory rate of 25cycles/min than 15cycles/min. Triggering delay, cycling delay, pressure–time product, pressure rise time and pressure in the mask were different between all ventilators (p < 0.01). There was no difference between ventilators for minute ventilation (p = 0.21) and peak pressure (p = 0.24). Dedicated NIV ventilators induced a lower pressure–time product than ICU and transport ventilators (p < 0.01). The rising time was found higher with dedicated NIV ventilators than with others (p < 0.01).

**Conclusion:** Despite the implementation of NIV algorithm, the most recent ICU ventilators appear to be less efficient than dedicated NIV ventilators, particularly in terms of PVA and work of breathing. These performances could however change according to the underlying respiratory disease and/or the number of leaks.

**Compliance with ethics regulations:** N/A.

**Figure 1 (abstract FC-087):**
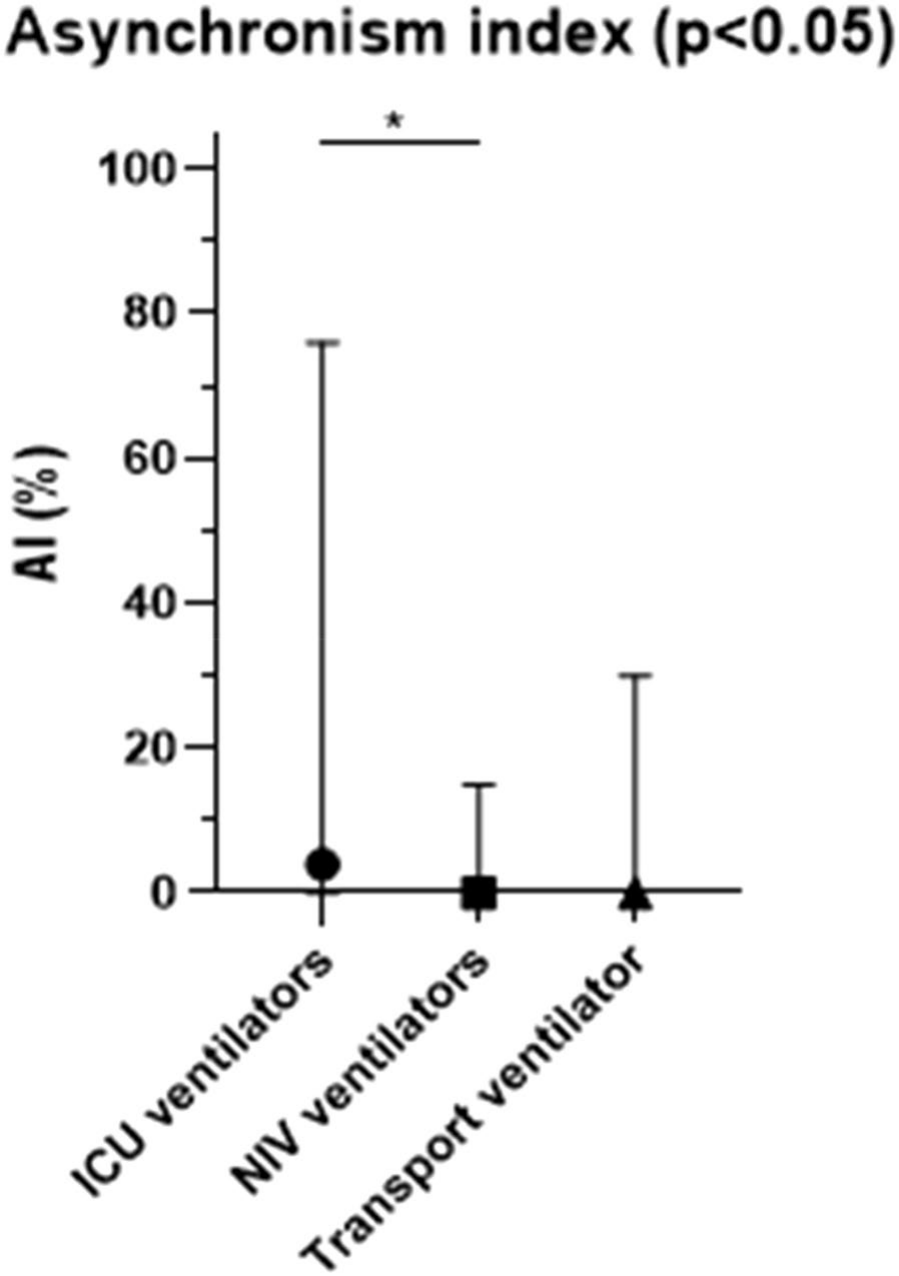
Comparison of Asynchronism Index (AI) between ventilator types, all ventilatory patterns combined, expressed as median (point, square, triangle) and 1st and 3rd interquartiles (bars). * = p < 0.05

## FC-088 Evaluation of expiratory humidity with different heated humidifiers circuits

### François Lellouche^1^, Pierre-Alexandre Bouchard^1^

#### ^1^Institut Universitaire de Cardiologie et de Pneumologie de Québec, Québec, Canada

##### **Correspondence:** François Lellouche (francois.lellouche@criucpq.ulaval.ca)

*Annals of Intensive Care *2013, **13(Suppl 1)**:FC-088

**Rationale:** The optimization of the inspiratory and expiratory circuits used with heated humidification is necessary to avoid condensation and related problems, such as auto-triggering, difficulties to trigger the ventilator, or increase in expiratory resistances (1). The objective of the study was to compare different expiratory circuits of different heated wire humidifiers.

**Patients and methods/materials and methods:** On a bench model simulating humidified expired gas (absolute humidity of 35 mgH2O/L), we have measured hygrometry of expiratory gases at proximal side (corresponding to the Y-piece), and distal side (ventilator inlet) of expiratory circuits. The circuits evaluated were Inspired ref. 51005683 (Vincent medical), FP950 (ref:950A81J, Fisher&Paykel), MR850 (RT210, F&P) and Evaqua2 (RT380, F&P), at different room temperatures (22–24 °C and 28–30 °C). The ventilator settings were Assist Control, tidal volume 400 ml; respiratory rate 25/min, PEEP 5 cmH2O, FiO2 0.21, Flow 60 lpm. Hygrometry was measured with the psychrometric method and 3 measurements were performed for each condition. Expiratory Tidal Volumes in each condition were all recorded from the ventilator screen.

**Results:** Main results are shown in the figure. The absolute humidity was reduced along the expiratory circuit with the FP950 and the Evaqua circuits (RT380). To avoid condensation in the expiratory limb, the VHB20 increases the temperature along the expiratory limb (leading to a reduction of relative humidity with stable water content). A similar functioning was observed with the MR850/RT210 circuit. No condensation was found along the expiratory limb for the different tested circuits in the bench study conditions. Results at different ambient temperatures were very closes. Despite high temperatures at the inlet ventilator, the tidal volumes measured by the ventilator were very close.

**Conclusion:** Different strategies are used to avoid the occurrence of condensation in the expiratory limb. Two circuits (FP950 and Evaqua2) use ‘’porous’’ materials allowing a reduction of the humidity along the expiratory line. Two circuits (VHB20 and RT210) use a strategy of increased temperature allowing a reduction of the relative humidity to limit the risk of condensation. However, this strategy may alter the tidal volume measurements (which has not been shown with the ventilator used in the study), and may increase the resistance if HME filters are used instead of filters (with low hygrophobic/hygroscopic properties) (1).

**Reference 1**

Tonnelier A, et al. Impact of humidification and nebulization during expiratory limb protection: an experimental bench study. Respiratory care 2013; 58(8): 1315–22.

**Compliance with ethics regulations:** N/A.

**Figure 1 (abstract FC-088):**
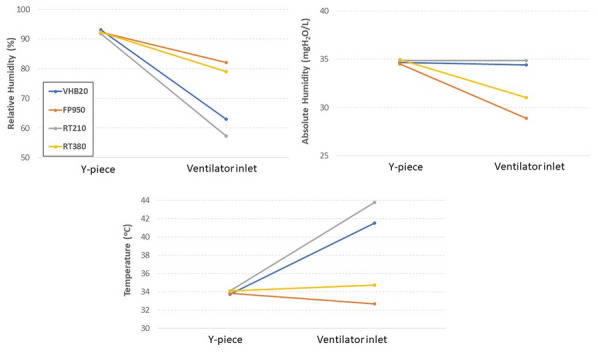
Mean Relative Humidity, Absolute Humidity and gas Temperature measured at the Y-piece and at ventilator inlet with different circuits used with VHB20, FP950 and MR850 (RT210 and RT380, Evaqua2) heated humidifiers

## FC-089 Performances and limits of Bag-Valve-Mask (BVM): a comparative bench and cadaver study

### Alexandre Broc^1^, François Morin^2^, Laura Polard^1^, Arnaud Lesimple^1^, Éloïse De Beaufort^1^, Adrien Drouet^3^, Hugo Schmit^4^, Alice Hutin^5^, Lionel Lahmaut^5^, Mathilde Taillantou-Candau^6^, Ugo Brisset^2^, Emmanuel Charbonney^7^, Stéphane Delisle^8^, Dominique Savary^2^, François Beloncle^6^, Jean-Christophe Richard^1,6^

#### ^1^Air Liquide Medical Systems, Med2Lab, Antony, France; ^2^University Hospital of Angers, Emergency Department, Angers, France; ^3^CHUV Centre hospitalier universitaire vaudois, Emergency Department, Lausanne, Suisse; ^4^Centre Hospitalier Annecy Genevois, Emergency Department, Annecy, France; ^5^SAMU de Paris and intensive care unit, Necker Hospital, Assistance Publique-Hôpitaux de Paris (APHP), Emergency Department, Paris, France; ^6^University Hospital of Angers, Medical ICU, Angers, France; ^7^Université de Montréal's Faculty of Medicine, Montréal, CANADA; ^8^Faculty of Medicine of the University Department of Family Medicine and Emergency Medicine, Montréal, Canada

##### **Correspondence:** Alexandre Broc (alexandre.broc@airliquide.com)

*Annals of Intensive Care *2013, **13(Suppl 1)**:FC-089

**Rationale:** Bag-Valve-Mask (BVM) is the most commonly used device for pre-oxygenation and ventilation during cardiopulmonary resuscitation (CPR). A minimal FiO2 above 85% is expected during pre-oxygenation [1] while insufflated volume (VTi) should not exceed 8 ml/kg during CPR [2]. The objective is to compare the performances of BVM in simulated pre-oxygenation and during CPR settings.

**Patients and methods/materials and methods:**

Nine BVM were evaluated as follows:

1. Pre-oxygenation: Moderate (PEEP 0) and severe (PEEP 0 and PEEP 5) spontaneous breathing profiles were simulated on an active lung model (ASL 5000). Actual FiO2 (inspired fraction of oxygen) was measured and CO2 rebreathing was evaluated.
2. CPR: BVM ventilation was performed by healthcare professionals belonging to three different emergency or ICU departments (n = 36) on a specific manikin. Ventilation was tested during mechanical Interrupted Chest Compressions strategy (30:2). The same procedure was repeated by healthcare professionals (n = 3) who provided manual ventilation on two cadavers with three BVM.

**Results:**

1. Pre-oxygenation: FiO2 was lower than 85% for three BVM in severe profile and for two BVM in moderate profile (PEEP 0). In eight out of nine BVM for which a PEEP valve was attached (PEEP 5), FiO2 was above 85% but at the expense of a slight increase in resistive load. One BVM induced CO2 rebreathing.
2. Ventilation during CPR: on the bench, mean VTi were above the predefined target range for lung protective ventilation (4–8 ml/kg of PBW) for three BVM. Similar results were observed on the cadaver model.

**Conclusion:** BVM tested demonstrated varying performances in pre-oxygenation and CPR settings. The minimal 85% FiO2 target expected during simulated pre-oxygenation was not guaranteed for three BVM. During simulated CPR, VTi actually delivered varied significantly and was excessive for three BVM. The BVM working principle explained at least in part most of these observations.

**Reference 1**

Frerk, C., Mitchell, V. S., McNarry, A. F., Mendonca, C., Bhagrath, R., Patel, A., O’Sullivan, E. P., Woodall, N. M., & Ahmad, I. (2015). Difficult Airway Society 2015 guidelines for management of unanticipated difficult intubation in adults. British Jour.

**Reference 2**

Acute Respiratory Distress Syndrome Network, Brower, R. G., Matthay, M. A., Morris, A., Schoenfeld, D., Thompson, B. T., & Wheeler, A. (2000). Ventilation with lower tidal volumes as compared with traditional tidal volumes for acute lung injury and the ac.

**Compliance with ethics regulations:** Yes in clinical research.

**Figure 1 (abstract FC-089):**
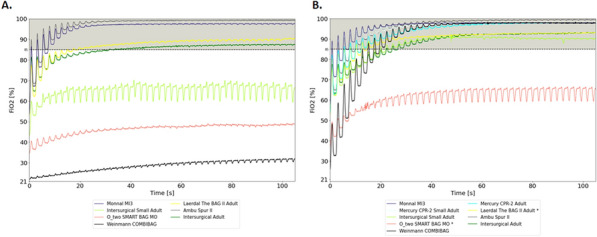
Actual FiO2 measured in percentage according to the time in dynamic conditions. a. Severe patient PEEP 0 cmH2O. b. Severe patient PEEP 5 cmH2O

## FC-090 Modeling lung ventilation in de novo acute respiratory failure: a bench study

### Elise Artaud-Macari^1,2^, Emeline Fresnel^2,3^, Adrien Kerfourn^2,3^, Christian Caillard^2,4^, Robin Thévenin^1^, Clemence Roussel^1^, David Debeaumont^5^, Marie-Anne Melone^1,2^, Gurvan Le Bouar^1^, Pierre-Alexandre Roger^1^, Luis-Carlos Molano^1^, Francis-Edouard Gravier^2,6^, Tristan Bonnevie^2,6^, Clement Medrinal^7,8^, Mathieu Salaun^1,9^, Antoine Cuvelier^1,2^, Christophe Girault^2,10^

#### ^1^Pulmonary, Thoracic Oncology and Respiratory Intensive Care Department, Rouen University Hospital, Rouen, France; ^2^Normandie Univ, UNIROUEN, UR3830-GRHVN, Institute for Research and Innovation in Biomedicine (IRIB), Rouen, France; ^3^Kernel Biomedical, Rouen, France; ^4^Elbeuf-Louviers-Val de Reuil Intercommunal Hospital, Medical and Surgical Intensive Care Unit, Saint Aubin Les Elbeufs, France; ^5^Department of Respiratory and Exercise Physiology and CIC-CRB 1404, Rouen University Hospital,, Rouen, France; ^6^ADIR Association, Rouen University Hospital, Rouen, France; ^7^Intensive Care Unit Department, Groupe Hospitalier du Havre, Le Havre, France; ^8^Université Paris-Saclay, UVSQ, Erphan, Versailles, France; ^9^Normandie Univ, UNIROUEN, UR4108-LITIS Lab QuantIF, Rouen, France; ^10^Medical Intensive Care Department, Rouen University Hospital, Rouen, France

##### **Correspondence:** Elise Artaud-Macari (eliseartaudmacari@yahoo.fr)

*Annals of Intensive Care *2013, **13(Suppl 1)**:FC-090

**Rationale:** In de novo acute respiratory failure (ARF), the worsening of underlying pulmonary injuries could be related to the risk of P-SILI (patientself-inflicted lung injury) during spontaneous breathing or VILI (ventilator- induced lung injury) with mechanical ventilation. To better assess the potential consequences of spontaneous or mechanical oxygenation techniques, we aimed to design a model of de novo ARF and assess the conditions that favor volo- and atelec-trauma.

**Patients and methods/materials and methods:** Based on our previously described model of ventilation in healthy subjects and patients with lung fibrosis (altered compliance (C)) on a 2-compartment (A1 and A2) artificial mechanical lung simulator, we have simulated conditions of breathing patterns and inspiratory efforts in de novo ARF based on physiological data from the literature and our cohort of healthy subjects at exercise. Inspiratory efforts were adjusted to maintain a minute ventilation of 28 L/min. Worsening of ARF was simulated by increasing the proportion of altered alveoli, and the model was therefore composed of a proportion (X) of alveoli with basal compliance (C1 = 200 ml/cmH_2_O) and a proportion (Y) of alveoli with altered compliance (C2 = 50 ml/cmH_2_O), where X + Y = 100%. Resistance was kept constant (5 cmH_2_O/L/s) in all simulations. The following parameters were measured: tidal volume (TV) distribution, end-expiratory lung volume (EELV), driving pressure (DeltaP), driving transpulmonary pressure (DeltaP_tp_), dynamic alveolar strain (Strain_alv_), mechanical power (MP), time lag between inspiratory flow in A1 and A2 (Deltat _(Q1-Q2)_).

**Results:** During ARF worsening,TV remained stable but was found higher in the compartment with highest compliance. EELV decreased dramatically (as low as 167 ml) especially in the compartment with the lowest compliance, suggesting a potential risk of alveolar collapse. From mild to very severe ARF, DeltaP increased (from 11.9 to 14.4 cmH_2_O), DeltaP_tp_ increased (5.9 to 14.2 cmH_2_O), Strainalv increased (0.8 to 1.2), suggesting a potential overdistension, and MP increased (27.8 to 45.1 J/min). Deltat _(Q1-Q2)_ was postitively correlated with the difference of compliance between A1 and A2 (r = 0.98, CI_95%_ (0.7783; 0.9990), p = 0.002) and increased when this difference increased, suggesting Pendelluft phenomenon.

**Conclusion:** This physiological bench study designed for the first time a mechanical ventilation model that can reproduce lung inhomogeneity and the mechanisms of volo- and atelec-trauma in de novo ARF. This model could be useful in the future to evaluate the impact of various oxygenation strategies in this clinical scenario.

**Compliance with ethics regulations:** Yes in clinical research.

**Figure 1 (abstract FC-090):**
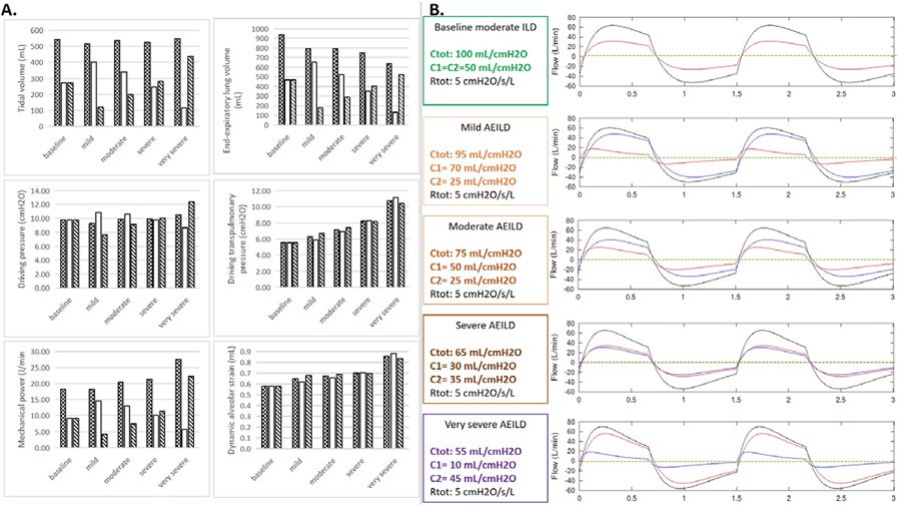
Method of de novo hypoxemic acute respiratory failure modelization on a mechanical artificial lung ventilation simulator. C1 was the compliance of healthy lung at baseline, i.e. 200 mL/cmH2O, C2 was the compliance of severe fibrosis model previously described

## FC-091 Impact of ICU ventilator bias-flow on inhaled nitric oxide administration: a comparative bench study

### Mathilde Lefranc^1^, Alice Vuillermoz^2,3^, Nathan Prouvez^1^, Alexandre Broc^1^, Alain Mercat^2,3^, François Beloncle^2,3^, Jean-Christophe Richard^1,2^

#### ^1^Air Liquide medical systems, Antony, France; ^2^CHU d'Angers, Angers, France; ^3^Université d'Angers, Angers, France

##### **Correspondence:** Alice Vuillermoz (alice.vuillermoz@chu-angers.fr)

*Annals of Intensive Care *2013, **13(Suppl 1)**:FC-091

**Rationale:** Several inhaled nitric oxide (iNO) devices are available with different modes of iNO administration: “basic continuous administration” (MiniKINOX, Cahouet), “guided” (OptiKINOX, ALMS) with an administration only during inspiratory-time and “synchronized-to-ventilator” (EZKINOX, EKU, SoKINOX, iNOsystems) which adapt continuously their administration to the total flow in the inspiratory limb. We hypothesized that the different bias-flows among ICU ventilators may affect iNO delivery, accuracy, NO_2_ formation and gas consumption depending on the device's capability to accurately detect and measure bias-flow.

**Patients and methods/materials and methods:** Four iNO devices were evaluated on a Michigan test lung (ARDS model), connected to four ventilators (V800, Dräger; R860, GE Healthcare; E500, Löwenstein andServo-i, Getinge) on volume-controlled ventilation. We tested four bias flows (2, 2.5, 10 and 30 L/min). NO was administered just before the heated humidifier, on the inspiratory limb. Four NO concentration settings were set: 5, 10, 14 and 20 ppm. Actual NO and NO_2_ concentrations were measured in the test lung by NO and NO_2_ electrochemical cells (SoKINOX). NO-N_2_ mixture flow was measured by TSI-flowmeter to assess gas consumption.

**Results:** With minimal bias flow, almost all the devices are able to achieve target iNO concentration except the OptiKINOX (16 [15; 17] ppm). Increasing the bias flow to 30 L/min resulted in a significant reduction of iNO concentration except for the two “synchronized-to-ventilator” devices that maintained iNO inside the target. As a result, NO/N_2_ consumption was higher with these devices (Figure 1). NO_2_ was below the toxic threshold in all experimental conditions.

**Discussion:** Bias flow setting is usually hidden or neglected by the caregiver. By increasing the quantity of fresh gas circulation in the inspiratory (and expiratory) circuit-limb, bias flow may dilute iNO concentration, resulting in iNO under administration. By directly detecting the inspiratory flow, new devices are able to adapt properly to iNO administration, thus maintaining iNO actual administration in the prest target.

**Conclusion:** Differences in ventilator bias flow can generate large variations on iNO administration accuracy. New devices detecting and compensating the total flow (inspiratory and bias flow) accurately deliver iNO concentration (EZKINOX and SoKINOX). On the contrary, devices that do not assess bias flow (MiniKINOX, OptiKINOX) significantly under deliver iNO.

**Compliance with ethics regulations:** N/A.

**Figure 1 (abstract FC-091):**
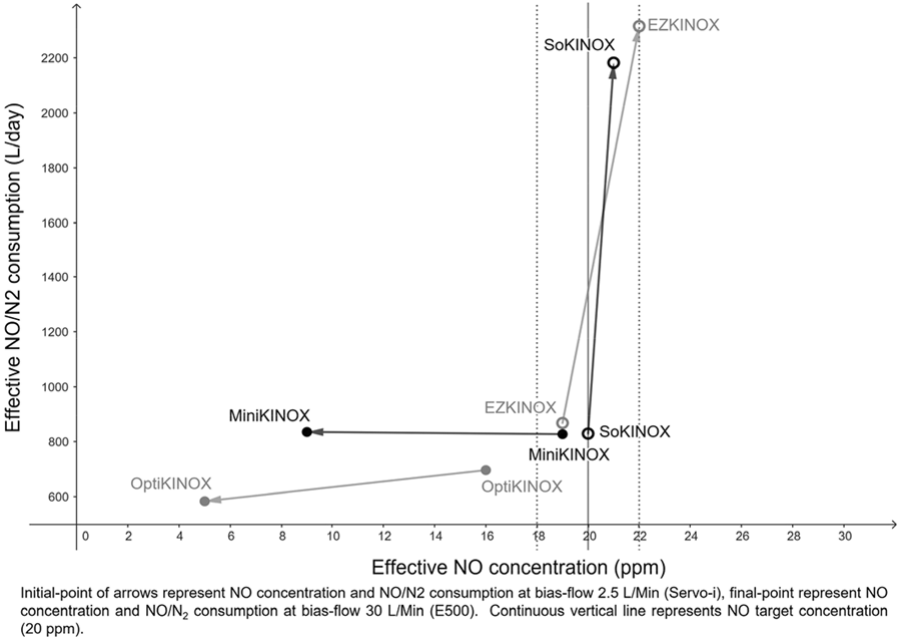
Effect of bias flow (2.5 L/min or 30 L/min) on iNO concentration and NO/N2 gas consumption with different devices (MiniKINOX, OptiKINOX, EZKINOX, SoKINOX)

## FC-092 Prognostic factors for severe acute asthma in children

### Samira Kalouch^1^

#### ^1^CHU Ibn Rochd, Casablanca, Maroc

##### **Correspondence:** Samira Kalouch (dr.kalouch@gmail.com)

*Annals of Intensive Care *2013, ** 13(Suppl 1)**:FC-092

**Rationale:** Severe acute asthma is an asthma attack that does not respond to well-conducted treatment, or whose intensity is unusual in its evolution or its symptomatology, putting the vital prognosis at risk. The aim of our study is to identify the prognostic factors of severe acute asthma in children hospitalized in the pediatric intensive care unit.

**Patients and methods/materials and methods:** This is a retrospective and analytical study, which involved 81 children, hospitalized for severe acute asthma in the pediatric intensive care unit, during a period from February 2013 to October 2022. The different variables collected at admission were analyzed and compared between two groups: deceased patients and survivors. Statical analysis (univariate and multivariate) by logistic regression was performed using SPSS version 23 software.

**Results:** The frequency was 2%. The average age was 5.5 years, with a predominance of females. There were 2 peaks of admission in April and October (12%). 4% of patients were mechanically ventilated. 46% of the patients reported having a trigger for the current attack, 27% of the patients had a viral infection, and 7% of patients suffer from passive smoking. 77% of the patients were under asthma treatment, however 36% had a bad compliance and 17% stopped the treatment. At admission, 64% of the patients had SpO2 between 80 and 90%. 42% of patients received magnesium sulfate, 74% of patients had antibiotic therapy. The mean duration of intensive care stay was 3.71 days. Overall, 15% of patents died. In univariate and multivariate analysis, the factors significantly influencing mortality in our study were: parental smoking, neurological faiure, hypercapnia and acidosis, mechanical ventilation, oxygenation by non-rebreather mask, magnesium sulfate and antibiotic therapy administration.

**Conclusion:** Comprehensive management of the asthmatic child is essential, as well as educating parents about the value of treatment and the severity of asthma disease, and the advantage of rapid management in acute severe asthma.

**Compliance with ethics regulations:** Yes in clinical research.

## FC-093 The effect of low oxygen concentrator flow rates on aerosol drug delivery to a simulated spontaneously breathing neonatal patient

### Marc Macgiollaeain^2^, Elena Fernandez Fernandez^1^, Mary Joyce^2^, Ronan Macloughlin^2^

#### ^1^Medical Affairs, Aerogen Ltd, Galway, Ireland; ^2^R&D, Science and Emerging Technologies, Aerogen Ltd, Galway, Ireland

##### **Correspondence:** Elena Fernandez Fernandez (efernandez@aerogen.com)

*Annals of Intensive Care* 2023, **13(Suppl 1):**FC-093

**Rationale:** According to the latest World Health Organisation Figures an estimated 2.4 million new-borns died from preventable and treatable diseases [1]. Amongst the leading causes were respiratory infections such as pneumonia. Supplemental oxygen in conjunction with aerosol therapy has become a mainstay in the treatment of respiratory illnesses in paediatric and neonatal patients in the critical care setting. The reliable supply of oxygen to prevent hypoxaemia is critical to improving patient outcomes [2]. Oxygen concentrators are one such means of facilitating reliable, medical grade supplemental oxygen generation and delivery, particularly in regions with lesser resources or where bottled oxygen is not readily available. The objective of this study was to investigate the effects of various low oxygen concentrator flow rates on aerosol drug delivery to a simulated spontaneously breathing neonatal patient.

**Patients and methods/materials and methods:** 500 µg of salbutamol was aerosolised using an Aerogen Solo vibrating mesh nebuliser and Pro-X controller (Aerogen, IRE). The aerosol was delivered using the Bitmos Oxy 6000 oxygen concentrator (Bitoms GmbH, DE), via a nasal cannula (Hudson RCI Teleflex Comfort flo, Flexicare Medical, UK) at flow rates of 0.2, 0.3 & 0.5 LPM to a 3D printed anatomically correct head model of a 4-month-old (RONAN model). A simulated neonatal breathing pattern (BR: 40 BPM, Vt: 25 mL, I:E: 1.0:3.0) was generated using a breathing simulator (BRS 2100, Copley, UK). The level quantity of aerosol available at the level of the trachea was measured using UV-spectrophotometry at 276 nm. A one-way ANOVA test determined statistical significance at p < 0.05.

**Results:** The results of this study are presented in Figure 1.

**Conclusion:** In this study, it was found that low oxygen concentrator gas flow rates had a statistically significant effect on the quantity of aerosol available at the level of the trachea in a simulated spontaneously breathing neonatal patient.

**Reference 1**

World Health Organisation (n.d.). Child mortality (under 5 years) https://www.who.int/news-room/fact-sheets/detail/levels-and-trends-in-child-under-5-mortality-in-2020

**Reference 2**

Enarson, P., LaVincente, S., Gie, R., Maganga, E. & Chokani, C. Implementation of an oxygen concentrator system in district hospital paediatric wards throughout Malawi. Bulletin of the World Health Organisation, 2008 (86); 344–348.

**Compliance with ethics regulations:** Yes in clinical research.

**Figure 1 (abstract FC-093):**
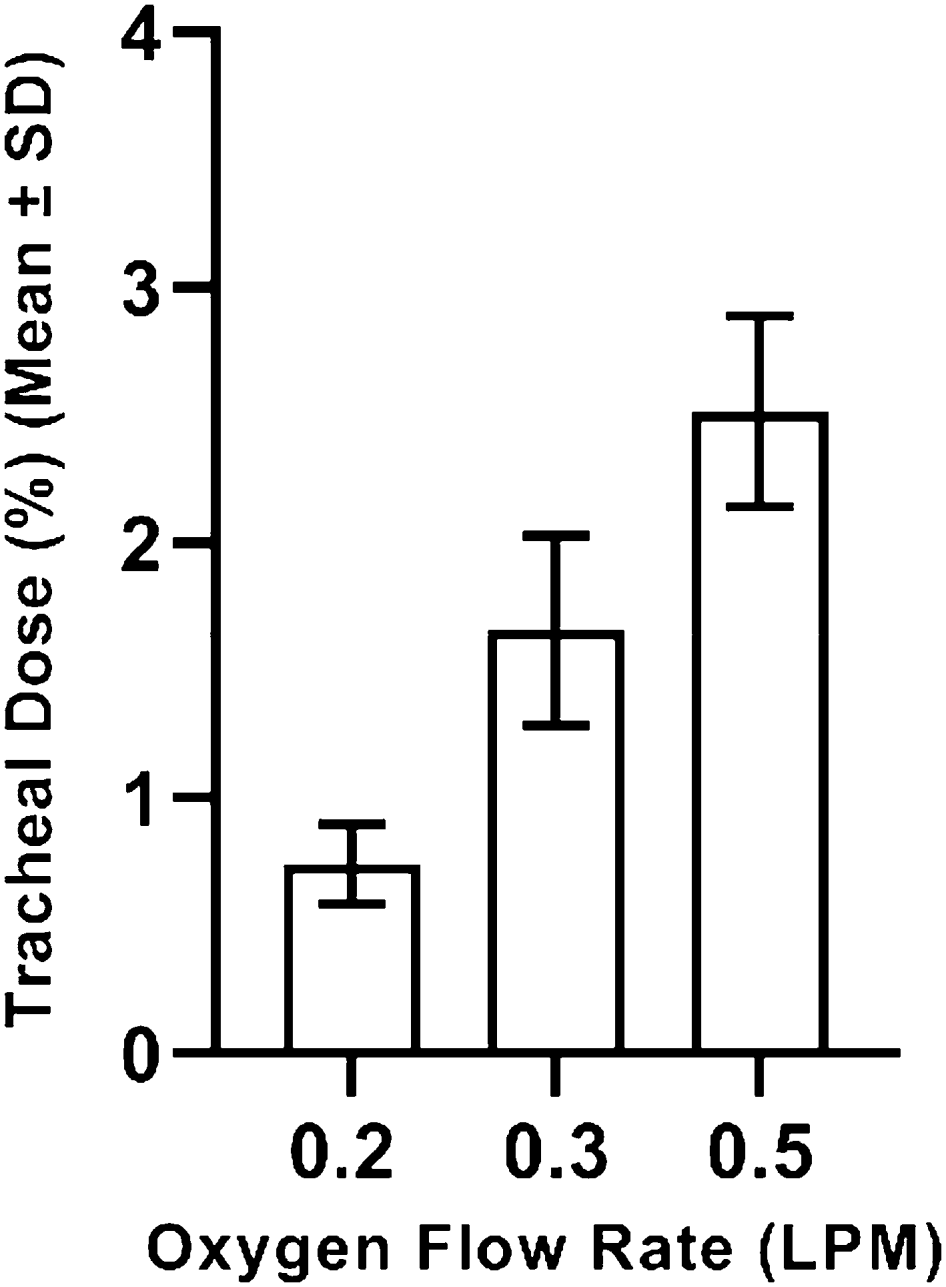
Effect of low oxygen concentrator flow rates (LPM) on aerosol drug delivery at the level of the trachea to a simulated spontaneously breathing neonate patient

## FC-094 Development of an annotated video database for child respiratory distress assessment

### Simon Mellul^1^, Buratti Cecilia^2^, Monisha Shcherbakova^2^, Victor Lestrade^2^, Rita Noumeir^3^, Philippe Jouvet^2^

#### ^1^Faculté de médecine de Tours, Tours, France; ^2^CHU Sainte Justine, Montréal, Canada; ^3^Laboratoire de traitement de l'information en santé, École de technologie supérieure, Montréal, Canada

##### **Correspondence:** Simon Mellul (simonmellul@orange.fr)

*Annals of Intensive Care* 2023, **13(Suppl 1):**FC-094

**Rationale:** In critically ill children, respiratory distress is clinically assessed using child inspection, auscultation and pulse oximetry. This assessment relies on caregivers’ expertise to analyze these signs including retraction signs. The development of supervised machine learning methods has the potential to help in the analysis of retraction signs in real time using video image. However, these methods need video annotation by caregivers to identify retraction signs. The aim of our study was to create an annotated video database of children with respiratory distress.

**Patients and methods/materials and methods:** In a video database (MEDEVAC database), we classified the videos of children according to the sign of retractions they presented. The database included 30 min RGB, 3D and thermography videos of critically ill children admitted in the pediatric intensive care of our institution. To annotate the MEDEVAC database, we collected demographics data (age, gender, disease) and patient’s sedation clinical scores at the time of video collection, from the electronic medical record. We classified seven categories of retractions signs on videos: sous costal, inter costal, sous sternal, supra sternal, supra clavicular, thoraco-abdominal asynchrony and generalized signs. A caregiver clinically scored retraction signs using a qualitative scale (+, ++, +++). The database is approved by the review ethical committee (number 2016-1242) as well as the respiratory distress analysis by machine learning methods (number 2020-2276).

**Results:** 176 videos were classified. The reason for admission was respiratory failure in 51.1% of cases (including 51 children with bronchiolitis), post cardiac surgery in 24.4% of cases and 24.5% other reasons. 36 children were mechanically ventilated with an endotracvheal tube (iMV), 59 children were on none-invasive mechanical ventilation (NIV), 22 children had high flow nasal canula (HFNC) and 9 were breathing spontaneously without any respiratory support (VS). In children < 6 years old (yo), the median (range) comfort B score was 12 (20–6) in iMV group and the median (range) FLACC scale was 0 (8–0) in the other groups. In children > 6 yo, we used a numerical scale of pain named EN which median (range) was 0 (2–0). The distribution of retractions signs was: 73 sous costal, 27 inter costal, 41 sous sternal, 14 supra-sternal, 5 supra clavicular retraction. Only 4 thoraco-abdominal asynchrony were seen as videos were registered when children were stabilized.

**Conclusion:** We annotated a large video database of critically ill children with respiratory failure. This video database will help for the development of clinical decision support system aimed at early recognition of respiratory distress.

**Compliance with ethics regulations:** Yes in clinical research.

## FC-095 Lung ultrasound performance in neonatal respiratory distress: an evaluative study in a pediatric intensive care unit

### Amal Miraoui^1^, Assaad Louati^1^, Ahmed Tamboura^1^, Ahmed Hajji^1^, Ahmed Ayari^1^, Chatila Ibn Hadj Hessine^1^, Asma Bouziri^1^, Aida Borgi^1^, Khaled Menif^1^

#### ^1^Hôpital d'enfants Bechir Hamza de Tunis, Tunis, Tunisie

##### **Correspondence:** Amal Miraoui (amal.miraoui92@gmail.com)

*Annals of Intensive Care* 2023, **13(Suppl 1):**FC-095

**Rationale:** Neonatal respiratory distress (NRD) is the most common cause of admission in neonatal intensive care units with high morbidity and mortality. Such an emergency justifies the need of a diagnostic tool that is accessible, fast and reproducible. The aim of this study was to evaluate lung ultrasound (LUS) performance in NRD and interoperator agreement level for LUS results.

**Patients and methods/materials and methods:** We conducted a prospective, observational and evaluative study in our pediatric intensive care unit between June the 1st, 2020 and June the 30th, 2021 including newborns admitted for NRD aged under 24 h of life.

**Results:** During the study period, we included 76 newborns. The median gestational age was 37 weeks [IQR: 34 + 1 − 38 + 3]. The median weight was 2950 g [IQR: 2412–3500]. The median age at transfer was 7 h [IQR: 5–12]. Clinical findings at admission were a median respiratory rate of 77 cycles/min [IQR: 69–85], a median Silverman score of 4.5 [IQR: 3–5] and a median FiO_2_ of 50% [IQR: 30–60]. Transient tachypnea of the newborn (TTN) was the most common etiology in our study. Agreement between LUS and reference diagnosis was moderate (kappa = 0.56; CI 95%: 0.43–0.69). LUS performance was studied for the 3 most common etiologies and concluded to a sensitivity and a specificity of 61.2% and 88.9% in TTN, 76.9% and 90.5% in respiratory distress syndrome (RDS), 100% and 98.5% in meconium aspiration syndrome (MAS). Interoperator agreement was almost perfect (kappa = 0.94; CI 95%: 0.88–1.00).

**Conclusion:** Lung ultrasound was performant in identifying the etiology of neonatal respiratory distress. A great interoperator agreement has been proved. This diagnostic tool should be introduced in all neonatal and pediatric intensive care units.

**Compliance with ethics regulations:** Yes in clinical research.

## FC-096 Foreign body inhalation in children

### Anas Erragh^1^, Karima Amanzoui^1^, Marouane El Harit^1^, Samira Kalouch^1^

#### ^1^CHU Ibn Rochd, Casablanca, Maroc

##### **Correspondence:** Karima Amanzoui (amenkarima@gmail.com)

*Annals of Intensive Care* 2023, **13(Suppl 1):**FC-096

**Rationale:** Inhalation of the foreign body is one of the main causes of death by domestic accidents in children. Asphyxia remains the main immediate risk, but very serious sequelae may appear if the extraction time is prolonged. With advances in instrumentation and anesthesia, the severity of this incident has been greatly reduced. The aim of this work was to study the epidemiological, clinical, para-clinical, therapeutic criteria as well as the anesthetic modalities of the extraction of inhaled foreign bodies at the ABDERRAHIM HAROUCHI Children’s University Hospital in Casablanca.

**Patients and methods/materials and methods:** Retrospective study carried out in the pediatric intensive care unit of the CHU IBN ROCHD during the years 2014–2015, which allowed the recruitment of 42 cases of foreign bodies extracted under general anesthesia.

**Results:** The inhalation of foreign bodies was frequent between 6 months and 3 years (74%), and the diagnosis was based on the search for the syndrome of penetration which was present in 88% of the cases. Chest X-ray was normal in 50% of cases and foreign bodies were radiopaque in 12% of cases. The first-line treatment was the extraction of foreign bodies by rigid bronchoscopy under general anesthesia. The nature of foreign bodies was dominated by plants including peanuts (28.57%) with a predominance of intra-bronchial location (90.48%). The evolution was favorable for most patients, one patient had a cardiorespiratory arrest following a severe bronchospasm, two patients presented a pneumothorax of great abundance and three patients presented a bronchial spasm.

**Conclusion:** Early extraction of foreign bodies is essential to avoid the risk of potentially serious medium and long-term sequelae. We must also strengthen the prevention and education of parents, as well as their training on the Heimlich maneuver, in order to ensure good vigilance, and a possible reduction in the incidence of this serious accident.

**Compliance with ethics regulations:** Yes in clinical research.

## FC-097 Automatic abstraction of data from electronic medical data monitoring systems (eMDMS) for large clinical trials: lessons learnt

### Avishay Sarfatti^2^, Michaël Sauthier^1^, Marisa Tucci^1^, Philippe Jouvet^1^, Stéphane Leteurtre^3^, Samiran Ray^4^, Simon Stanworth^5^, Pierre Demaret^6^, Thierry Ducruet^1^, Jacques Lacroix^1^, Geneviève Du Pont-Thibodeau^1^

#### ^1^CHU Sainte-Justine, Montréal, Canada; ^2^Oxford University Hospitals NHS Foundation Trust, Oxford, Royaume-Uni; ^3^Hôpital Jeanne de Flandre, Lille, France; ^4^Great Ormond Street Hospital, London, Royaume-Uni; ^5^NHS Blood & Transplant/Oxford Radcliffe Hospitals, Oxford, Royaume-Uni; ^6^CHC Liège, Liège, Belgique

##### **Correspondence:** Jacques Lacroix (jlacroix052@gmail.com)

*Annals of Intensive Care* 2023, **13(Suppl 1):**FC-097

**Rationale:** There is increasing interest in using electronic medical data monitoring system (eMDMS) to record outcome data for clinical trials. We provide an analysis of the P-OpTTICCA study, a pilot randomized controlled trial (RCT) of red cell transfusion thresholds in children admitted to the pediatric intensive care unit (PICU) of four international hospital sites. The aim was to describe the challenges and lessons learnt for using electronic systems to abstract data directly from an eMDMS and to transfer these data into the case report form (CRF) of the study.

**Patients and methods/materials and methods:** 120 patients were enrolled from the PICU of four university-affiliated hospitals In Canada, France and United Kingdom. The basic design of P-OpTTICCA is described in the registry “clinicalTrials.gov” (NCT03871244).

**Results:** All data were extracted manually in one site. Most data (88%) were extracted electronically in the three other sites, using the eMDMS IntelliSpace Critical Care and Anesthesia (ICCA, Philips Medical Systems, Royal Philips Electronics, Netherland). We identified a number of challenges at the three sites supporting use of electronic data. Specific limitations were identified for reports of co-morbidity. Electronic data flows needed to be tailorred to each site; for example, some data were not stored in the same fields in their eMDMS even though the three PICUs used ICCA. Some laboratory data were stored in different locations. Like hematocrit which can be found in hematology and blood gases fields. Different unique patient identifiers were used, including for the same patient. Local algorithms (e.g., to extract data and calculate PIM2 and PELOD-2 scores) were not reliable and needed to be validated manually.

**Discussion:** Despite the many advantages of electronic systems for collection of routine data for analysis in clinical trials, our study highlighted the importance of understanding the full IT processes at all sites. Multiple validations are necessary to check data collection in the data warehouse, and data transfer from the warehouse into the study database. Such validations must be done in each site because some data were not located in the same field in the eMDMS of different sites even though they used the same software.

**Conclusion:** These electronic set up and validation processes must be taken into account in the trial budget and execution.

**Compliance with ethics regulations:** Yes in clinical research.

## FC-098 Ten years practice of ecmo in Strasbourg PICU

### Maxence Fernandes^1^

#### ^1^CHU de Strasbourg, Strasbourg, France

##### **Correspondence:** Maxence Fernandes (maxence.fernandes@chru-strasbourg.fr)

*Annals of Intensive Care* 2023, **13(Suppl 1):**FC-098

**Rationale:** Despite the life-saving benefit of extracorporeal membrane oxygenation (ECMO), ECMO in Pediatrics iremains a risky procedure with a high mortality rate because of the pathology underneath but also given the complications. This work aims to analyze 10 years of ECMO’s practice in our University Hospital of Strasbourg, France.

**Patients and methods/materials and methods:** We analyzed in a retrospective way data from all patients supported by veno venous or veno-arterial or VA ECMO from 2010 to 2020.

**Results:** Data from 59 patients were analyzed. Our population had a median age of 5.5 months and a median weight of 5.1 kgt. 80% of ECMO were AV. For around 60% of patients, ECMO was implanted after cardiac surgery. Overall, 46% of patients died, and acute kidney injury was the only factor significantly associated with death in our population. The most frequent adverse outcomes were hemorragic (50%), thrombotic (30%), infectious (19%) and neurological (12%).

**Conclusion:** In the 10 years period, we used ECMO for 59 patients for various indication. Acute Kidney Injury was the only risk factor for death in our cohort. Hemorragic and thrombotic adverse outcome are by far the most prevalent.

**Compliance with ethics regulations:** Yes in clinical research.

**Table 1 (abstract FC-098):**
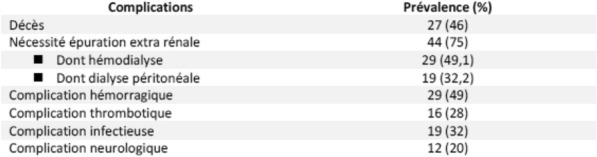
Complications during ECMO

## FC-099 Accuracy of FAST ultrasound in pediatric polytrauma patients

### Alaeddine Zouari^1^, Rim Karray^1^, Rezk Ghorbel^1^, Noureddine Rekik^1^

#### ^1^Hôpital Habib Bourguiba Sfax, Sfax, Tunisie

##### **Correspondence:** Alaeddine Zouari (zouari.aladin@gmail.com)

*Annals of Intensive Care* 2023, **13(Suppl 1):**FC-099

**Rationale:** The management of polytrauma patients at the emergency department has evolved significantly in recent years, in particular with the introduction of FAST ultrasound as a tool to detect possible intra-abdominal effusion. According to the literature, the sensitivity of FAST ultrasound in the pediatric population is lower than that in adults, thus posing a problem on the contribution of FAST ultrasound. In this study, we assessed the sensitivity and specificity of FAST ultrasound compared to the GOLD STANDARD which is the computed tomography, in pediatric polytrauma patients.

**Patients and methods/materials and methods:** This is a prospective study of patients who are admitted for polytrauma, over a period of 1 year, from first August, 2020 to 31st July, 2021 at the emergency department in the CHU Habib Bourguiba in Sfax. The inclusion criteria were polytrauma patients under the age of 16 years, admitted to our department during this period and in whom a FAST ultrasound and a CT scan were performed.

**Results:** The total number of multiple trauma patients admitted to our department during this period was 470 patients. The polytrauma pediatric population counted 37 patients, i.e. 7.8%. The average age was 9.11 years. They were 15 women and 22 men. On clinical examination, only one patient was in cardiorespiratory arrest. The average schok index was 0.9. The average Glasgow score was 13.6. The FAST ultrasound was abnormal in 8 cases by detecting 8 cases of peritoneal effusion, while CT scan detected 13 cases. In our study we found 25 true negatives, 8 true positives and 4 false negatives. The overall predictive value or precision was 89.1%. The positive predictive value, the negative predictive value, the sensitivity and the specificity of intraperitoneal fluid detection were 100%, 86.2%, 66.6% and 100% respectively.

**Conclusion:** Our results were consistent with those reported in the literature and concluded that FAST ultrasound can improve and guide the management of polytrauma patients.

**Compliance with ethics regulations:** Yes in clinical research.

## FC-100 Place of transthoracic ultrasound in the management of patients admitted in shock to the emergency department of hospital of Oran

### Djamel Alachaher^1,2^, Mourad Goulmane^1,2^, Nabil Tabet Aoul^1,2^

#### ^1^Centre Hospitalo-universitaire d'Oran, Oran, Algerie; ^2^Faculté de médecine d'Oran, Université Oran1 Ahmed Ben Bella, Oran, Algerie

##### **Correspondence:** Djamel Alachaher (jalachaher@yahoo.fr)

*Annals of Intensive Care* 2023, **13(Suppl 1):**FC-0100

**Rationale:** Shock is a frequent reason for hospitalization in the emergency department. The pathophysiological mechanisms are often intricated, making their determination by clinical examination alone difficult, hence the interest of certain additional explorations, in particular Transthoracic Ultrasound (TTE). The main objective of our study is to evaluate the contribution of echocardiography in our patients admitted in shock on the diagnostic, therapeutic and prognostic level.

**Patients and methods/materials and methods:** A clinical and epidemiological, descriptive, prospective and monocentric study realized in the reception unit of the medical emergency department of our hospital interesting any patient over the age of 15 admitted for a no traumatic shock. The group of patients benefiting from ETT evaluated over a period of 2 years from 01-01-2016 to 31-12-2017 and designated the “echo yes” group was compared with a second group of patients who did not benefit from the ETT collected from 01-01-2015 to 31-12-2015 and named the “echo no” group. A comparative study between the two groups on the epidemiological’s data, the determination of the etiological mechanism, and on the evolution.

**Results:** Seventy-seven (77) patients from the general population (n = 156) presenting with a shock collected in our study benefited from ETT, i.e. a frequency of 49.35% with an average age of 64.40 ± 17.70 years [17–106] and a sex ratio of 1.13. ETT by two-dimensional and Doppler exploration made it possible to determine the etiological mechanism in 94.8% of cases [38.96% (n = 30) hypovolemic shock, 36.36% (n = 28) cardiogenic shock, 16 0.88% (n = 13) septic shock, 2.59% (n = 2) anaphylactic shock]. The rate of cases with an undetermined mechanism fell very significantly (“echo yes”: 5.20% vs “echo no”: 36.70%), a drop of more than 31%, p < 0.001. The rectification of the initial diagnostic hypothesis was noted in 29/77 patients, i.e. a frequency of 37.66%. The ETT made it possible to orient the treatment, dobutamine was largely prescribed in the “echo yes” group (33.80%) vs (13.20%) the “echo no” group. It also improved the prognosis (in-hospital mortality rate of “echo yes” patients 33.80% vs. 48.10% “echo no”); a decrease of more than 14%

**Conclusion:** Our work has well illustrated and shown the contribution of ETT in improving the overall management of patients in shock admitted to the medical emergencies by modifying the initial diagnostic hypothesis with as corollary the therapeutic conduct,

**Compliance with ethics regulations:** Yes in clinical research.

## FC-101 Critical care ultrasound among Tunisian ICU residents: a cross-sectional survey

### Hassen Ben Ghezala^1^, Iyed Maatouk^1^, Amira Ben Jazia^1^, Nozha Brahmi^1^

#### ^1^Centre Mahmoud Yaacoub d'assistance médicale urgente de Tunis, Tunis, Tunisie

##### **Correspondence:** Hassen Ben Ghezala (hassen.ghezala@gmail.com)

*Annals of Intensive Care* 2023, **13(Suppl 1):**FC-0101

**Rationale:** Critical Care ultrasound (CCUS) is more and more used. The practice of this tool by residents practicing in Tunisian intensive care units (ICUs) is often limited. An objective assessment of this training has not been performed. The purpose of our survey was to assess the knowledge about CCUS among ICU residents.

**Patients and methods/materials and methods:** This is a cross-sectional study conducted among residents in ICU practicing during the period from January to June 2021 in several intensive care units in Tunisia. Residents were assessed based on their theoretical and practical knowledge about ultrasound. Data were collected using a French language questionnaire distributed on the day of the selection of the residents’ posts for the next training period (at the end of June 2021). Participation was completely voluntary.

**Results:** Out of a total of 75 residents, 37 accepted to answer to the survey (participation rate = 49%). The specialty of all participants was intensive care medicine. The majority were female (66.4%). The mean age was 29 ± 12 years. Only 5% of participants (n = 2) had previously received echocardiography training and only 8.1% of the participants have received training for lung ultrasound (LU). Of the participants, 80.1% of residents (n = 30) had never performed a transthoracic echocardiography (TTE). Competence in performing echocardiography was self-assessed as never done, quite good and bad by 51.4%, 5.4% and 43.2% of responders respectively. Most of the residents (86%) did not insert before ultrasound-guided central venous catheters. Only 3% have dedicated training for this type of procedure. Participants considered that TTE was a mandatory examination in order to assess hemodynamics and patients in respiratory failure in 91.9% and 73% respectively. Most participants (89%) approved the role of TTE in management plan. Different views that the participant could obtain in trans thoracic echocardiography (TTE) were parasternal long axis (16.2%), short axis parasternal section (10.8%), apical 4/5 cavities (16.2%), apical 2/3 cavities section (5.4%), sub-costal 4 cavities section (10.8%), and subcostal section (inferior vena cava incidence) (28.6%). 51.4% of ICU residents who answered the survey reported a good knowledge of CCUS criteria of fluid responsiveness such us small and hyperkinetic ventricular cavities. All participants (100%) thought that teaching ultrasound is a necessary part of the training of intensivists.

**Conclusion:** Our study highlighted the lack of training of Tunisian ICU residents regarding critical care ultrasound learning. There is a significant hope from residents to learn this golden tool as part of their training programs.

**Compliance with ethics regulations:** Yes in clinical research.

## FC-102 Correlation between portal blood flow and aortic velocity time integral among 106 critically ill patients

### Bertrand Litzler^1^, Gaël Piton^1^, Gilles Capellier^1^, Hadrien Winiszewski^1^, Thibault Vieille^1^

#### ^1^CHU Minjoz Besançon, Besançon, France

##### **Correspondence:** Bertrand Litzler (bert_lit@hotmail.fr)

*Annals of Intensive Care* 2023, **13(Suppl 1):**FC-0102

**Rationale:** Evaluation of cardiac output is required among critically ill patients. Trans-thoracic echocardiography allows non-invasive measurement of cardiac output but is not always feasible. Measuring the portal blood flow might help to estimate the cardiac output, because the level of hepatic portal flow could be proportional to that of the cardiac output, in absence of portal hypertension. We hypothesized that there would be a correlation between the mean portal velocity and the cardiac output, estimated with the aortic velocity time integral. We aimed to study such correlation using hepatic ultrasonography and trans-thoracic echocardiography.

**Patients and methods/materials and methods:** Adult patients requiring ICU admission, were prospectively included. Pregnant women were excluded. We measured mean portal velocity in the main trunk, aortic velocity integral, usual clinical and biological variables, as well as 28-day mortality. We studied the correlation between mean portal velocity and aortic velocity time integral.

**Results:** This prospective study was performed between January and June 2022 among 106 critically ill patients admitted to the ICU for medical or surgical cause. The SOFA score was 7 [4–10], 65% required mechanical ventilation and 36% required catecholamine use, 28-day mortality rate was 27%. Hepatic portal ultrasonography was feasible in 100% of the patients. One patient was excluded from the analysis because the portal blood flow was negative (cirrhosis with severe portal hypertension). There was a positive correlation between mean portal velocity and aortic velocity time integral (R = 0.49, p < 0.001; test de Spearman). A mean portal velocity ≥ 15 cm/s was associated with aortic velocity time integral ≥ 15 cm among 93% of the patients (81/87). A mean portal velocity < 15 cm/s was associated with aortic velocity time integral < 15 cm among 50% of patients (9/18). A mean portal velocity < 15 cm/s predicted an aortic velocity time integral < 15 cm with a 60% sensibility and 90% specificity. Mean portal velocity was significantly reduced among 28-day non-survivors.

**Conclusion:** The measurement of mean portal velocity is a supplementary tool for estimating the cardiac output. A mean portal velocity ≥ 15 cm/s is strongly suggestive of an aortic time velocity integral ≥ 15 cm. The mean portal velocity has a prognostic value among non-selected critically ill patients. Portal ultrasonography is feasible in almost all critically ill patients.

**Compliance with ethics regulations:** Yes in clinical research.

**Figure 1 (abstract FC-102):**
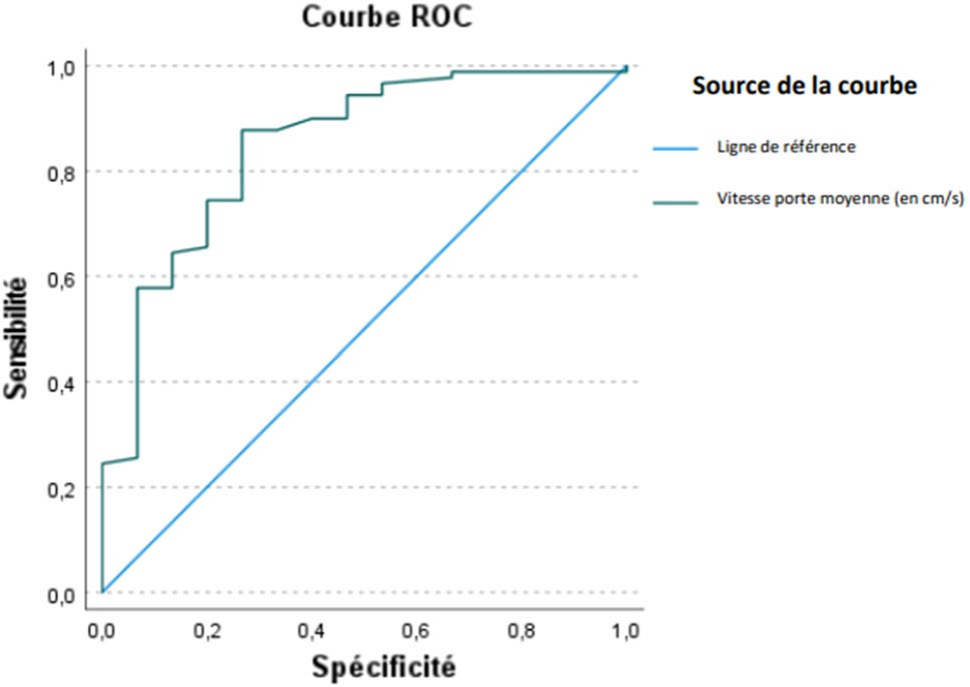
ROC curve

## FC-103 Evaluating left ventricular filling pressures by mitral Doppler does not predict the respiratory or hemodynamic consequences of volume expansion

### Bénédicte Zerr^1^, Nicolas Terzi^2^, Nicholas Sedillot^3^, Carole Schwebel^2^, Fabienne Prieur^1^, Vincent Peigne^1^

#### ^1^Centre hospitalier Métropole Savoie, Chambéry, France; ^2^CHU Grenoble Alpes, La Tronche, France; ^3^Centre Hospitalier de Bourg en Bresse Fleyriat, Viriat, France

##### **Correspondence:** Vincent Peigne (vincentpeigne@yahoo.fr)

*Annals of Intensive Care* 2023, **13(Suppl 1):**FC-0103

**Rationale:** Although static hemodynamic indices have been shown for over a decade to be poor predictors of response to volume expansion, many practitioners assess left ventricular filling pressures (LVFP) before prescribing fluid infusion, in the hope of avoiding the occurrence of a deterioration of haematosis. This work pragmatically assesses the merits of this practice.

**Patients and methods/materials and methods:** The inclusion criteria were: adult patient, mechanically ventilated, in whom vascular replacement was prescribed for hemodynamic instability. Exclusion criteria were pregnancy and breastfeeding. Clinical (including heat rate, arterial pressure, SpO_2_, FiO_2_) and echocardiographic (including mitral Doppler and aortic VTI) parameters were collected before and 30 min after volume expansion. Patients with an increase in LVFP (identified by one of the following mitral Doppler criteria: E/E′ > 10 or E/A ≥ 2 or TDE < 120 ms) were compared to those without an increase in LVFP). The primary endpoint was the frequency of occurrence of respiratory deterioration after volume expansion, defined as a decrease in the SpO_2_/FiO_2_ ratio of at least 15% after volume expansion. Secondary outcomes were response to volume expansion (defined as at least a 15% increase in cardiac output) and frequency of inability to use pulse pressure variation (PPV).

**Results:** 87 patients were included in 3 centers [median age 65 (55–73.5) median SAPS2 56 (43.5–72)]. Volume expansion had already been administered within the previous 12 h in 70% of patients [median volume 1500 mL (940–3062)] and 77% were receiving catecholamines. At least one criterion of increased LVFP was found in 30% (26/87) of the patients. Only 2 patients (2/87—2.3%), who had no increase in LVFP, showed respiratory deterioration. The response to volume expansion was identical in patients with or without increased LVFP (25% and 23%, p 0.8). The conditions for use of the PPV were not met in 54 (62%) patients: non-sinus rhythm (15), spontaneous ventilatory cycles (23) or tidal volume ≤ 6 mL/kg (27).

**Conclusion:** This pragmatic prospective study shows that the evaluation of LVFP by mitral Doppler is not a reliable index to predict the respiratory and hemodynamic consequences of filling. PPV, a more reliable marker according to the literature, was optimally applicable in only 40% of our patients.

**Compliance with ethics regulations:** Yes in clinical research.

## FC-104 Inferior vena cava collapsibility index to predict fluid responsiveness in spontaneously breathing patients: a prospective validation study

### Benoît Ter Schiphorst^1^, Claire Bourel^1^, Arthur Durand^1^, Alexandre Pierre^1,2^, Thierry Onimus^1^, Raphaël Favory^1,2^, Sébastien Preau^1,2^

#### ^1^CHU de Lille, Lambersart, France; ^2^INSERM UMR 1167, Lille, France

##### **Correspondence:** Benoit Ter Schiphorst (b.terschiphorst@gmail.com)

*Annals of Intensive Care* 2023, **13(Suppl 1):**FC-0104

**Rationale:** The ability of inferior vena cava (IVC)-derived parameters to predict fluid responsiveness in spontaneously breathing patients remains unclear in the intensive care unit (ICU). It has been previously proposed that a collapsibility index of IVC (cIVC) ≥ 33% or ≥ 44% is appropriate to detect fluid responsiveness during non-standardized (cIVC-ns) or standardized (cIVC-st) breathing, respectively. The aim of the study was to test the ability of these predetermined thresholds to predict fluid responsiveness.

**Patients and methods/materials and methods:** We have included spontaneously breathing adult patients admitted to the ICU of a university hospital. The cIVC-ns and cIVC-st were assessed at baseline in a semi-recumbent position, with the trunk elevated at 45°. Ultrasound recordings of the IVC were performed in a sub-xiphoid, long-axis view, using the two-dimensional mode. Measurements of cIVC-ns and cIVC-st were remotely performed, after anonymization, 4 cm from the cavo-atrial junction. The standardized inspiration maneuver consisted of a short (< 5 s) buccal depression beyond − 3 mmH_2_O. Fluid responsiveness was defined as an increase in the subaortic time-velocity integral during a passive leg raising test (ΔVTI) ≥ 10%.

**Results:** Among the 61 included patients, 38 (62%) were fluid-responsive. The median (interquartile range) simplified acute physiologic score II was 26 (23; 33). The cIVC were significantly higher in fluid responsive compared to fluid unresponsive patients: 56% (38; 70) versus 8% (4; 20) for cIVC-ns (p < 0.001), and 61% (52; 76) versus 18% (7; 26) for cIVC-st (p < 0.001). cIVC-ns and cIVC-st were positively correlated to ΔVTI: r = 0.56 and r = 0.58, respectively (p < 0.001). A cIVC-ns ≥ 33% predicted fluid responsiveness with a sensitivity and a specificity of 84% and 83%, respectively. A cIVC-st ≥ 44% predicted fluid responsiveness with a sensitivity and a specificity of 94% and 87%, respectively. A cIVC-ns ≥ 33% and a cIVC-st ≥ 44% classified correctly respectively 86% and 91% of the patients (p = 0.18).

**Conclusion:** cIVC is a reliable non-invasive tool to predict fluid responsiveness in spontaneously breathing patients admitted to ICU, provided that its measurement is performed 4 cm from the cavo-atrial junction in a sub-xiphoid, in a long-axis view, using the two-dimensional mode. In case of low values of cIVC during non-standardized breathing, we advise performing a standardized inspiration to increase the sensitivity of this parameter.

**Compliance with ethics regulations:** Yes in clinical research.

## FC-105 Pleuro-pulmonary ultrasound to assess pulmonary congestion: correlation with echocardiography and clinical evaluation: preliminary results

### Asma Mehdi^1^, Ahlem Trifi^1^, Eya Seghir^1^, Bedis Tlili^1^, Lynda Masseoud^1^, Asma Ouhibi^1^, Nousayer Azzouz^1^, Meriem Cherif^1^, Sami Abdellatif^1^

#### ^1^Hospital la Rabta, Tunis, Tunisie

##### **Correspondence:** Asma Mehdi (asmaelmahdi245@gmail.com)

*Annals of Intensive Care* 2023, **13(Suppl 1):**FC-0105

**Rationale:** Pulmonary oedema (PO) is associated with high morbi-mortality. Its diagnosis and patient’s monitoring are mainly based on clinical evaluation. Nevertheless, echocardiography with E/E′ ratio and inferior vena cava collapsibility index (CI IVC) are considered as validated tools to assess diastolic dysfunction and filling pressure. Currently, the B lines score (BLS) obtained with pulmonary ultrasound is considered to follow up patients under diuretics. We aimed to study the correlation between BLS and clinical score, E/E′, CI IVC to evaluate congestion and monitor patients under diuretics admitted in ICU for PO.

**Patients and methods/materials and methods:** A prospective evaluative study in ICU adult patients who presented PO. A clinical score of congestion (Brest score), E/E′ ratio, CI IVC and BLS were collected at 4 times: T1 (at inclusion), T2 (after 24 h), T3 (after 72 h) and T4 (at day 5). BLS was obtained by summing B lines in every lung area (12 areas of the BLUE protocol).

**Results:** In the included 16 patients, sex ratio was 1.6, and the mean age was 56.8 ± 13.9. Nine patients had hypertension and five had chronic cardiac disease. PO was diagnosed at admission in 11 cases. An acute coronary syndrome was incriminated in 25% of cases. Poor adherence to medication was noted for all patients with history of chronic cardiac disease. 14 patients received loop diuretics at inclusion with a median dose of 120 mg/d [IQR, 90–420]. Non invasive ventilation was required for 12patients. At inclusion, mean Brest score, E/E′, and BLS were respectively 9.94 ± 2; 12, 6 ± 3 and 40, 63 ± 12 with decrease over time, mainly at T2. Correlation studies: (1) According to the study times (all patients combined): A strong correlation was found between BLS and clinical score with Rho score = 1. No correlation was showed between BLS and E/E′ nor CI IVC. (2) According to the parameters values in each patient and in each time: significant correlations were showed between BLS and clinical scores at all the study times yet the stronger one was at T4 (Table 1). Moderate correlations were also found between BLS and E/E′, at inclusion and day 5. BLS had no correlation with CI-IVC at any time. (3) All patients and study times combined: the only significant correlation was objectified between BLS and clinical score (Rho = 0.729; p < 10^−3^).

**Conclusion:** During monitoring of PO under treatment, BLS score was strongly correlated with the clinical score and was moderately correlated with the E/E′ for certain follow-up times. This good correlation attributes a performance to this score to integrate it into our follow-up protocols.

**Compliance with ethics regulations:** Yes in clinical research.

**Table 1 (abstract FC-105):**
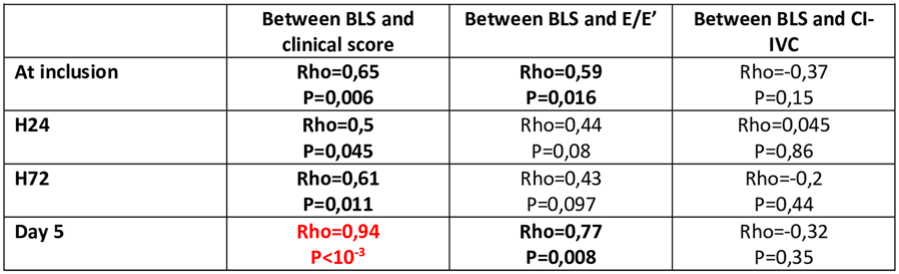
Correlation results between all assessed parameters

## FC-106 Short and long-term multimodal characterization of cardiac injury in critically ill patients with COVID-19: a longitudinal study

### Didac Aurenche Mateu^1^, Lucas Morand^1^, Gilles Bernardin^1^, Jean Dellamonica^1^, Mathieu Jozwiak^1^, Denis Doyen^1^

#### ^1^Réanimation Médicale, Nice, France

##### **Correspondence:** Didac Aurenche (daurenchem@gmail.com)

*Annals of Intensive Care* 2023, **13(Suppl 1):**FC-0106

**Rationale:** Numerous electrocardiographic (ECG) or echocardiographic abnormalities have been described during COVID-19. The significance and the long-term evolution of these abnormalities are poorly described in critically ill COVID-19 patients. Purpose of the study: To determine the incidence, characteristics and the long-term evolution of cardiac abnormalities during COVID-19.

**Patients and methods/materials and methods:** We conducted a prospective single-center study in a university hospital. Critically ill COVID-19 patients were consecutively included. A multimodal cardiac assessment combining ECG, transthoracic echocardiographic (TTE) and cardiac biomarker analysis was performed several times during the ICU stay. Acute cardiac injury (ACI) was defined by troponin elevation and newly diagnosed ECG or echocardiographic abnormalities, or both. The same multimodal analysis was performed within 3 to 6 months after discharge from hospital. According to a predefined decision algorithm, other advanced tests were performed: stress ECG, 24-h holter ECG, non-invasive functional imaging (single-photon emission computed tomography or stress echocardiography), coronary angiography or cardiac magnetic resonance imaging.

**Results:** Over a period of 1 year, 100 patients were included, of whom 37 (37%) presented with ACI. A total of 61% were on mechanical ventilation, 34% on high-flow oxygen therapy and 5% on noninvasive ventilation. The cardiovascular risk SCORE was higher in patients with than without ACI (10% vs. 3%, p < 0.01). Among ACI, the most frequently abnormalities were: alteration of the left ventricular global longitudinal strain (86%) and of the right ventricular free wall (78%), ECG/TTE signs of left ventricular abnormalities (i.e. suggestive of coronary heart disease, Takotsubo syndrome, myocarditis or septic cardiomyopathy) (73%), right ventricular dysfunction (51%), atrial fibrillation (30%), left ventricular diastolic dysfunction (24%), left ventricular systolic dysfunction (22%), cor pulmonale (14%) and pericardial effusion (14%). During long-term follow-up, all abnormalities improved but persisted significant alteration of the left and right ventricular strain (43%). Significant coronary lesions were found in 8 patients (10% of patients with follow-up). Only one diagnosis of myocarditis was confirmed (1%). The incidence of long-term atrial fibrillation was 6%. The median delay of follow-up between ICU discharge and consultation was 107 [95–162] days.

**Conclusion:** ACI is frequent in critically ill COVID-19 patients. All ECG/TTE abnormalities improved during follow-up. The incidence of coronary artery disease and atrial fibrillation were significant in the long term, but that of myocarditis remained rare. These results suggest that screening for coronary artery disease and long-term atrial fibrillation should be discussed in these patients given the significant morbidity and mortality associated with these pathologies.

**Compliance with ethics regulations:** Yes in clinical research.

**Figure 1 (abstract FC-106):**
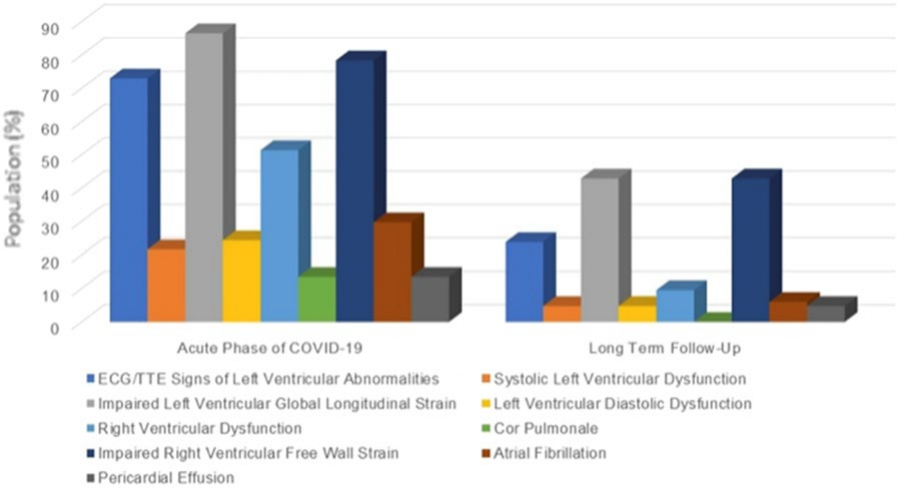
Evolution of each cardiac abnomality incidence between acute phase of COVID-19 and long-term follow-up

## FC-107 Does pulmonary mechanics correlate with right ventricular echographic parameters in very critically ill intubated covid-19 patients?

### Amir Bedhiafi^1^, Ahlem Trifi^2^, Asma Ouhibi^2^, Badis Tlili^2^, Salma Rabhi^1^, Noussair Azzouz^1^, Meriem Cherif^2^, Asma Mehdi^2^, Sami Abdellatif^2^

#### ^1^Medical ICU, la Rabta hospital, Tunis, Tunisie; ^2^Medical ICU, la Rabta hospital, Faculty of Medicine of Tunis, Tunis, Tunisie; ^3^Department of Anesthesia, University Hospital Center La Rabta, Tunis, Tunisie

##### **Correspondence:** Amir Bedhiafi (bedhiafi.emir@gmail.com)

*Annals of Intensive Care* 2023, **13(Suppl 1):**FC-0107

**Rationale:** The COVID-19-related severe acute respiratory syndrome (ARDS), is characterized by impaired pulmonary mechanics. In these patients, various studies, based on trans-thoracic echocardiography (TTE), have also shown several right ventricular (RV) abnormalities [1]. In this study, we aimed to investigate the correlation between altered respiratory mechanics and RV dysfunction in critically ill patients with COVID-19-related ARDS.

**Patients and methods/materials and methods:** This is a single-center cross-sectional study conducted in a medical ICU of a Tunisian university hospital from September 2020 to June 2021. We included consecutive confirmed COVID-19 patients older than 18 years old with moderate to severe ARDS, based on the BERLIN definition [2], and receiving invasive mechanical ventilation (IMV). Baseline demographics and clinical characteristics were collected. Trans-thoracic echocardiography was performed within the first 24 h of IMV by trained operators. Concomitant respiratory mechanics monitoring was recorded. A univariate analysis was performed.

**Results:** A total of 75 patients were enrolled. The median age was 64 [54–71] years with a male predominance, sex-ratio of 2.12. Major comorbidities were hypertension 38% and diabetes mellitus 38% with a median BMI of 26.2 [24.2–31] kg/m^2^. Acute cor pulmonale (ACP) was found in 18 (24%) patients. Pulmonary hypertension was noticed in 54 (72%) patients, with a median systolic pulmonary arterial pressure (SPAP) of 38 [32–45] mmHg. The median tricuspid annular plane systolic excursion (TAPSE) was 17 [15–19]. Concerning respiratory monitoring, the median PaO_2_/FiO_2_ ratio was 78 [69–102] under a PEEP of 8 [8–10], static lung compliance was 23 [19–28] mL/cmH_2_O, plateau pressure 27 [24–30] cmH2O, driving pressure 19 [16–20] cmH_2_O and mechanical power 27 [24.2–29.7] J/min. A linear regression was performed to assess the correlations. Patients with higher SPAP had lower static compliance, higher plateau pressure, driving pressure, and mechanical power but with no statically significant correlation (p = 0.653, 0.327, 0.516, and 0.471, respectively). On the other side, patients with lower TAPSE had also the same respiratory mechanics profile with no significant correlation (p = 0.833, 0.446, 0.626, and 0.143, respectively).

**Conclusion:** In the present study, no correlation was found between key noninvasive pulmonary mechanics. The study sample size and ETT timing, which may possibly underestimate RV dysfunction, could have impacted the results. Thus, further and larger studies might be needed to assess such correlations.

**Reference 1**

Paternoster G, Bertini P, Innelli P, et al. Right ventricular dysfunction in patients with COVID-19: a systematic review and meta-analysis. J Cardiothorac Vasc Anesth. 2021;35(11):3319–3324. https://doi.org/10.1053/j.jvca.2021.04.008

**Reference 2**

The ARDS Definition Task Force*. Acute respiratory distress syndrome: the berlin definition. JAMA. 2012;307(23):2526–2533. https://doi.org/10.1001/jama.2012.5669

**Compliance with ethics regulations:** Yes in clinical research.

## FC-108 Community acquired abdominal sepsis

### Khalid Khaleq^1^, Yassine El Khalfy^1^

#### ^1^CHU Ibn Rochd University Hassan2, Casablanca, Maroc

##### **Correspondence:** Khalid Khaleq (khaleq20@gmx.fr)

*Annals of Intensive Care* 2023, **13(Suppl 1):**FC-0108

**Rationale:** Community acquired abdominal sepsis a frequent medical-surgical emergency of the adult, acquired by the patient in a non-hospital environment. The multidisciplinary care is essential, involving surgeons, anesthetists, microbiologists and radiologists. The objective of our study is to describe the epidemiological, clinical, bacteriological, etiological, therapeutic and evolutionary data of community peritonitis, and to evaluate the prognostic factors.

**Patients and methods/materials and methods:** We carried out a descriptive and analytical retrospective study spread over 2 years and 9 months (between January 2020 and September 2022) involving 95 cases of Community acquired abdominal sepsis, hospitalized in the surgical emergency resuscitation department P33 of UHC Ibn Rochd Casablanca. We included adult patients with Community acquired abdominal sepsis who received medical and surgical management. The studied parameters are the demographic data, the clinical and paraclinical signs, the type of management and the evolution of the patients. The statistical analysis was performed using the SPSS software, the evaluation of prognostic factors was performed using a univariate and multivariate analysis.

**Results:** The study showed that the mean age was 53.88 ± 18.66 years, with a sex ratio of 1.71. The medical history was dominated by tobacco in 27.36%, the clinical signs were dominated by abdominal pain (96%), fever (66%), extra-abdominal signs (hemodynamic instability (64.21%), renal insufficiency (46.31%), hematological disorders (59.37%) and respiratory disorders (42.1%). Therapeutic management was based on perioperative resuscitation, treatment of organ failure, empirical antibiotherapy and surgery. The main etiologies of sepsis were: purulent effusion (36.84%), serous effusion and cholecystitis (15.78%), gallstone perforation (14.73%), intestinal necrosis (12.63%). The bacteriological samples taken during the surgery revealed the following bacteriological profile: predominance ofNGB (75.43%) dominated by *E. coli* (38.59%) followed by Klebsiella pneumoniae (17.54%), the mean hospital stay was 8.31 ± 9.54 days. The mortality rate was 62.1%. The main prognostic factors emerged in our study in univariate analysiswere: age, diabetes, cardiovascular disease, abdominal pain, hemodynamic failure, respiratory failure, renal failure, hematological failure, APACHE II and SAPSII scores, use of catecholamines and septic shock. The multivariate analysis showed a statistically significant association between renal failure, hematological failure, use of catecholamines and gallstone perforation.

**Conclusion:** Improving the prognosis of community sepsis of digestive origin can only be done by constant evaluation in terms of very early diagnosis and the establishment of appropriate resuscitation and antibiotic therapy.

**Reference 1**

Marshall JC, Innes M. Intensive care unit management of intra-abdominal infection. Crit Care Med. 2003;31(8):2228–2237. https://doi.org/10.1097/01.CCM.0000087326.59341.51

**Reference 2**

Blot S, Antonelli M, Arvaniti K, et al. Epidemiology of intra-abdominal infection and sepsis in critically ill patients: “AbSeS”, a multinational observational cohort study and ESICM Trials Group Project. Intensive Care Med. 2019;45(12):1703–1717.

**Compliance with ethics regulations:** Yes in clinical research.

## FC-109 Upper gastrointestinal bleeding in burns case control study

### Badis Tlili^1,2^, Hana Fredj^1,2^, Iheb Glenza^1,2^, Sarra Zarrouk^1,2^, Imen Jami^1,2^, Bahija Gasri^1,2^, Amel Mokline^1,2^, Amenne Allah Messadi^1,2^

#### ^1^Centre de traumatologie et des grands Brulés de Ben Arous, Ben Arous, Tunisie; ^2^Faculté de médecine de Tunis, Tunis, Tunisie

##### **Correspondence:** Badis Tlili (tlilibedis@gmail.com)

*Annals of Intensive Care* 2023, **13(Suppl 1):**FC-0109

**Rationale:** Upper gastrointestinal bleeding (UGIB) is a known relatively rare complication of the ICU stay. Few studies gave interest to this complication in severely burned patients requiring intensive care. To study the epidemiological, clinical manifestations, and evolutionary characteristics of the upper gastrointestinal bleeding and to identify its risk factors in severely burned patients’.

**Patients and methods/materials and methods:** Retrospective case–control study that took place in the severely burned patients’ intensive care unit of Tunisia over a period of 6 years (January 2016–July 2022), including all adult patients hospitalized with severe burns who presented at least one episode of upper gastrointestinal bleeding. The control group consisted of severely burned patients who did not present upper gastrointestinal bleeding during the ICU stay. The two groups were matched according to age, gender, and extent of burns.

**Results:** During the study period, 2258 patients were admitted, 52 patients presented an upper gastrointestinal bleeding, an incidence of 2.3%.

In the UGIB group, the mean age was 38.3 ± 21.9, sex ratio 2.25 (36/16), Total body burned surface area (TBSA) was 35.4% ± 14.3. The majority were consequences of domestic accidents 61.5% (32/52).

The average time of the gastrointestinal bleeding onset was 19 ± 17 days.

The clinical presentation was epigastric pain in 28 non-ventilated patients, hemoptysis in 12 patients (23.1%) and melena in 12 patients (23.1%). Esophagogastroduodenoscopy was performed in 45 patients (86.5%) showing: bulbar ulcer in 28 patients (65%), gastric ulcer in 8 patients (18%) and esophageal ulcerations in 3 patients (7%). Twenty-two patients developed hemorrhagic shock and 44 had acute anemia requiring blood transfusion in 67% of cases.

In the multivariate study, acute kidney injury OR 13.8, CI [2.9–67], p = 0.001, fluid intake < 2 ml/kg/%TBSA over the first 24 h OR = 10, IC [1.5–68.5], p = 0.019, and length of ICU stay > 10 days OR 48.2, IC [4.4–530], p = 0.002 were independent risk factors for the occurrence of UGIB. Mortality in the GIB group was significantly higher (55.8% vs 25%) (p = 0.001).

**Conclusion:** Upper gastrointestinal bleeding is a rare and serious complication of a burn patient’s stay in the ICU. Acute renal failure, fluid intake below 2 ml/kg/%TBSA in the first 24 h and a duration of ICU stay of more than 10 days were identified as independent risk factors for its occurrence.

**Compliance with ethics regulations:** Yes in clinical research.

## FC-110 Nutrition practices in the intensive care unit (observational study)

### Amine Schahrakane^1^, Khalid Khaleq^1^, Sara Madkhour^1^, Othman Maghrabi^1^, Driss Hamoudi^1^, Aziz Bouhouri^1^, Afak Nsiri^1^, Rachid Al Harrar^1^

#### ^1^Centre hospitalier universitaire IBN ROCHD, Casablanca, Maroc

##### **Correspondence:** Amine Schahrakane (amine.schahrakane@gmail.com)

*Annals of Intensive Care* 2023, **13(Suppl 1):**FC-0110

**Rationale:** Undernutrition results from an imbalance between energy and protein intake and requirements. It is associated with an increase in morbidity and mortality, which makes it essential to assess the nutritional status of any patient upon admission in order to determine early on the nutritional management best suited to him. The aim of this work is the screening of undernourished patients on admission, the nutritional management of patients hospitalized in intensive care units and the prognosis of these patients.

**Patients and methods/materials and methods:** This is a retrospective observational study of 180 cases admitted to the surgical intensive care unit of the University Hospital of CASABLANCA, during the period 2019–2020. We studied the clinical, biological and nutritional parameters, and compared these same parameters between two groups, undernourished and non-undernourished.

**Results:** 180 patients 42% were malnourished on admission; the mean age was 48 years with a male predominance 75%. The average length of stay was 14 days. 47% of whom were malnourished, compared with 53% of those who were not undernourished. Postoperative management of digestive surgery was the most frequent reason for hospitalization 34%, followed by polytrauma 28% and head trauma 13%. The most frequent risk factors for undernutrition were persistent symptoms related to digestive pathology 18%; age over 65 years 17%, diabetes 14%, cancer 13%, sepsis 7%. Biology: 80% had hypoalbuminemia, 95% had high CRP, 75% had high PCT 47% of patients received parenteral nutrition (CVC) against 82% for enteral nutrition (95% with gastric probe, 5% with Stomy) The most used type of nutrition was exclusive enteral nutrition 43%, mixed 40% and exclusive parenteral 13%. Albumin transfusion was used in 87% of the undernourished patients versus 13% of the non undernourished. 78% of the patients received antibiotics, 59% of whom were underlnourished, compared with 41% of those who were not. Complications related to nutrition were: digestive in 22%, nosocomial infections in 54%, 71% of whom were undernourished and 29% of whom were not, and hyperglycemia in 34% of cases, 49% of whom were undernourished and 51% were not. The mortality rate for all pathologies combined was 56%, with a rate of 76% for the undernourished and 24% for for those who were not.

**Conclusion:** Our results confirm the importance of an adapted nutritional strategy and its primary prognostic role. A good evaluation of the initial nutritional status and an early management allow to reduce considerably the occurrence of undernutrition as well as the associated complications.

**Compliance with ethics regulations:** Yes in clinical research.

## FC-111 Malnutrition in the medical intensive care unit: incidence, predictive and prognostic factors

### Rehab Haffar^1^, Fadwa Lahnine^1^, Mohammed Abidi ^1^, Firdaous Belkaid^1^, Kawtar Ziati^1^, Oussama Sounni^1^, Latifa Oualili^1^, Khalid Abidi^1^, Tarek Dendane^1^, Amine Ali Zeggwagh^1^

#### ^1^Hôpital universitaire Ibn Sina de Rabat, Rabat, Maroc

##### **Correspondence:** Rehab Haffar (rehabhaffar912@gmail.com)

*Annals of Intensive Care* 2023, **13(Suppl 1):**FC-0111

**Rationale:** Malnutrition in the intensive care unit is poorly studied, particularly in Moroccan hospitals. The goal of our study was to evaluate the nutritional status of patients and to identify the incidence, predictive factors and prognostic factors in patients admitted to the medical ICU of the Ibn Sina University Hospital in Rabat.

**Patients and methods/materials and methods:** This is a prospective descriptive and analytical monocentric study conducted at the Medical ICU of the Ibn Sina University Hospital in Rabat from March 8 to November 30, 2021. Patients aged 18 years or more who stayed in the department for a period of at least 72 h were included, so a total of 40 patients. Patients were diagnosed as malnourished if their BMI < 18.5 kg/m^2^, had significant weight loss and/or had a Transthyretin < 0.11 g/L. Predictive factors were determined by comparing the malnourished and non-malnourished populations. Prognostic factors were determined by comparing survivors and non survivors.

**Results:** At admission, 48% of patients were malnourished compared to 75% at discharge. In the malnourished group, the mean age was 60 years with a sex ratio of 1. The mean BMI was 25.3 kg/m^2^ at admission and 22.7 kg/m^2^ at discharge. The mean weight loss during hospitalization was 4.25 kg; 84% of patients experienced weight loss. 45% of patients had a transthyretin level of less than 0.11 g/L at admission and 55% at discharge. The only significant predictor of malnutrition was sepsis (OR = 6; 111 CI 95% [1.08–33.2]). The overall mortality rate was 42.5% with 50% mortality in the undernourished group. In the multivariate analysis, CRP and CPK are the predictive factors found.

**Conclusion:** Malnutrition is a common pathology in patients in ICU. It is responsible for nosocomial infections, of an extension of the stay and of excess mortality. In our study, the only predictor factor of malnutrition was sepsis.

**Compliance with ethics regulations:** Yes in clinical research.

## FC-112 Thromboembolic complications during digestive cancer before and after Covid 19, experience of a visceral surgery department about 6000 patients

### Rachid Jabi^1^, Brahim Housni^1^, Mohammed Bouziane^1^

#### ^1^Faculté de médecine et de pharmacie, Université Mohammed 1er, CHU Mohammed VI, Oujda, Maroc

##### **Correspondence:** Rachid Jabi (jabirachid@gmail.com)

*Annals of Intensive Care* 2023, **13(Suppl 1):**FC-0112

**Rationale:** The association between deep vein thrombosis (DVT) and cancer is a common clinical situation, known as ‘Trousseau’s syndrome’. This thrombosis may be indicative of cancer as well as aggravating the stages of multidisciplinary management.

**Aim:** To compare patients with a thromboembolic event in relation to digestive cancer before and after Covid 19 comparison between patients with venous thrombosis related to digestive cancer and other non-cancerous visceral etiologies to assess the impact of Covid 19 and vaccination on the epidemiology of thrombosis in the visceral setting.

**Patients and methods/materials and methods:** Retrospective analysis of 6000 patients hospitalized for a digestive reason associated with deep vein thrombosis between surgical and intensive care units over a 7-year period divided into two parts: G1 before and G2: during and after Covid 19.

**Results:** We collected 6000 patients, with an estimated incidence of thrombosis of 1.25%, i.e. 75 cases in the G1 group, 25 cases of thrombosis were reported out of 4000 patients, i.e. 0.6%, whereas in the G2 group, 50 cases were reported out of 2000 patients, i.e. 2.5% (p = 0.007) The mortality rate of our patients with venous thrombosis was 16% or 12 patients, 2 patients in the G1 group vs 10 patients in the G2 group (p = 0.043). Thromboembolic disease was associated with cancer in 80% of G1 patients (p = 0.04), whereas only 50% of our G2 patients had digestive cancer (p = 0.17). In Group G2, thrombosis was indicative of Covid in 20% of cases, cancer in 10% of cases, while massive pulmonary embolism complicated the initial management in 14% of cases. We noted the association of portal and cerebral thrombosis in 10% of G2 cases, and 3 cases (6%) with at least 3 thrombosed venous axes. We noted that 12% of our patients admitted for digestive cancer and thrombosis had received two doses of vaccine in the G2 group compared to 4% with a single dose.

**Conclusion:** Our study confirms the increased frequency of thromboembolic complications in digestive cancers. We also noted that the thrombosis spread with the Covid 19 infection and after vaccination. We propose to take Covid as a serious medical history and thromboembolic risk factor in the management of our patients.

**Compliance with ethics regulations:** N/A.

## FC-113 The future of the elderly operated for digestive pathology

### Khalid Khaleq^1^, Hicham Khalloud^1^, Khalid Hattabi^1^, Rachid Harrar^1^, Aziz Fadil^1^

#### ^1^CHU Ibn Rochd University Hassan2, Casablanca, Maroc

##### **Correspondence:** Khalid Khaleq (khaleq20@gmx.fr)

*Annals of Intensive Care* 2023, **13(Suppl 1):**FC-0113

**Rationale:** Demographic aging affects the entire planet; it is a more or less advanced global phenomenon depending on the country. Given this development, digestive surgery departments are increasingly required to take care of elderly patients. The objective of our study is to shed the light on the future of these patients after digestive surgery, to recognize the risk factors of mortality and to seek the predictive factors of death.

**Patients and methods/materials and methods:** We carried out a descriptive and analytical retrospective study based on the files of patients aged 65 or over, operated on for digestive abdominal pathology between January 1, 2020 and July 31, 2022 in the P35 operating room and having stayed in the P33 Surgical intensive care at the Ibn ROCHD University Hospital Center in Casablanca. Several data were the subject of univariate and multivariate statistical analysis by the SPSS software including the data relating to the patients, the preoperative, per and post operative evaluation as well as the severity scores.

**Results:** 100 patients aged 65 or over were included, 73 (73%) were men and 27 (27%) were women, with a sex ratio of 2.7. The average age of our patients is 72.1 years with a standard deviation of 6.54. 80% had comorbidities, dominated by smoking (39%), followed by hypertension (30%), and diabetes (29%). The most common surgical indications was the intestinal obstruction (21%), followed by acute peritonitis (17%), hernia/eventration (9%) and rectal tumors (9%). Overall mortality rate was 49% and the main cause of death was septic shock. Thanks to the multivariate statistical analysis, four prognostic factors significantly linked to mortality were deduced: ASA II: (OR: 3104.851; IC 95%: 2.677–3,600,597.205; P = 0.026). Enteral feeding: (OR: 23,812.129; CI 95%: 25.740–22,028,903.927; P = 0.004). Post-operative complications: (OR: 17.371; CI 95%: 1.378–218.964; P = 0.027) and APACHE II: (OR: 4.305; CI 95%: 1.566–11.834; P = 0.005).

**Conclusion:** The admission and treatment of elderly patients in intensive care raise new challenges for the intensive care physicians. Due to ethical and methodological difficulties, there are few data on the specific needs of the elderly person and on the responses to usual intensive care measures. Given the aging of the population and the future needs associated with surgery in elderly patients, there is a need for quality research in this area.

**Reference 1**

Hennessy D, Juzwishin K, Yergens D, Noseworthy T, Doig C. Outcomes of elderly survivors of intensive care: a review of the literature. Chest. Mai 2005;127(5):1764–74.

**Reference 2**

Ozkan E, Fersahoğlu MM, Dulundu E, Ozel Y, Yıldız MK, Topaloğlu U. Factors affecting mortality and morbidity in emergency abdominal surgery in geriatric patients. Ulus Travma Acil Cerrahi Derg. 2010;16(5):439–44.

**Compliance with ethics regulations:** Yes in clinical research.

## FC-114 Impact of the fungal component on the prognosis of pancreatic necrosis flow superinfections, about 400 cases

### Rachid Jabi^1^, Mohammed Bouziane^1^, Brahim Housni^1^

#### ^1^Faculté de médecine et de pharmacie, Université Mohammed 1er, CHU Mohammed VI, Oujda, Maroc

##### **Correspondence:** Rachid Jabi (jabirachid@gmail.com)

*Annals of Intensive Care* 2023, **13(Suppl 1):**FC-0114

**Rationale:** Acute pancreatitis is a very frequent reason for consultation. The morbidity of severe acute pancreatitis is often related to infectious complications. Although bacterial infections are the most common, fungal infections are increasingly recognized and are always associated with bacterial infections.

**Patients and methods/materials and methods:** A retrospective study of 400 cases of patients with necrotizing pancreatitis over a 7-year period. Purpose: To analyze the profile of patients admitted with necrotizing pancreatitis. To describe the particularity of fungal infection of necrotic flow.

**Results:** In a sample of 400 acute pancreatitis cases admitted to our clinic, 95% were of lithiasis origin, including 75 cases of necrotizing pancreatitis (18%). Casting infection was noted in 15 cases (20%) and fungal infection was present in 30% of cases. In all the patients with fungal infection, thrombocytopenia and at least two bacterial germs with resistance to antibiotics with pancreatic tropism were associated. Also, the fungal infection was always associated with organ failure, a long stay in intensive care, in our series 80% of the patients with fungal attack, that is to say 4 out of 5 died.

**Conclusion:** Fungal infection increases morbidity and mortality in necrotizing pancreatitis. The place of antifungal prophylaxis remains controversial. Limiting the use of broad-spectrum antibiotics, introducing enteral nutrition early, and changing vascular catheters promptly are important preventive strategies.

**Compliance with ethics regulations:** N/A.

## FC-115 Acute poisoning: predictive factors for hospitalization in an intensive care unit

### Medhioub Kaaniche Fatma^1^, Zouari Farah^1^, Ben Khlifa Atraa^1^, Smaoui Ayoub^1^, Allala Rania^1^

#### ^1^Mahres Regional Hospital, Faculty of Medicine, University of Sfax, Sfax, Tunisie

##### **Correspondence:** Medhioub Kaaniche Fatma (fatma_kaaniche@yahoo.fr)

*Annals of Intensive Care* 2023, **13(Suppl 1):**FC-0115

**Rationale:** Poisoning is the set of manifestations of the body following ingestion and/or superficial contact with a toxic substance. It can be accidental or voluntary. Acute poisoning is a frequent reason for consultation in the emergency department. The objective of our study is to identify the predictive factors of hospitalization in intensive care unit for acute poisoning.

**Patients and methods/materials and methods:** Poisoning is the set of manifestations of the body following ingestion and/or superficial contact with a toxic substance. It can be accidental or voluntary. Acute poisoning is a frequent reason for consultation in the emergency department. The objective of our study is to identify the predictive factors of hospitalization in intensive care unit for acute poisoning.

**Results:** During the study period, among the 44,763 patients admitted to the emergency room, we identified 80 cases (0.17%) of acute intoxication, 53 of which required hospitalization in intensive care (0.11%). The average age was 19 ± 9.5 years wit. A female predominance was observed with a sex ratio of 0.35. Forty-one patients (51%) presented drug intoxication. It was mono drug in 16 patients (20%) and multi-drug in 25 patients (31%). Pesticides were the cause of acute poisoning in 6 patients (7%). After analysis of the various epidemiological, clinical and biological parameters, the factors associated with hospitalization were multi-drug intoxication (OR = 3.3; 95% CI [2.6–6.7]; p = 0.031), reaching the toxic dose (OR = 1.7; 95% CI [1.1–3.5]; p = 0.04), paracetamol intoxication (OR = 1.4; 95% CI [1.2–2.7]; p = 0.02), the intoxication with antihypertensives (OR = 1.1; IC95% [1.1–2.8]; p = 0.01), intoxication with anxiolytics (OR = 1.9; IC95% [0.8–3.2]; p = 0.02), pesticide poisoning (OR = 1.8; 95% CI [1.2–4.2]; p = 0.03), miosis (OR = 1.2; 95% CI [1.2–4.2]; p = 0.043), convulsions (OR = 1.3 95% CI [0.8–2.1]; p = 0.001), tremor (OR = 1.3; 95% CI [1–2.8]; p = 0.003), sinus bradycardia (OR = 1.3; 95% CI [0.7–2.7]; p = 0.031) and conduction disorders (OR = 1.8; 95% CI [1.4–4.2]; p = 0.02).

**Conclusion:** All of these results underline the major number of hospitalizations. A specific poisoning guideline should be drawn up to standardize emergency procedures.

**Compliance with ethics regulations:** N/A.

## FC-116 Study of illicit drug intoxications in patients admitted in intensive care unit

### Salma Kammoun^1^, Hassen Ben Ghezala ^1^, Amira Ben Jazia^1^, Nozha Brahmi^1^

#### ^1^service de réanimation toxicologique du centre Mahmoud Yaacoub d'assistance médicale urgente (CMYAMU), Tunis, Tunisie

##### **Correspondence:** Salma Kammoun (utilecomptesk@gmail.com)

*Annals of Intensive Care* 2023, **13(Suppl 1):**FC-0116

**Rationale:** Illicit drugs are often consumed by young people during festivity for their psychostimulant effect. They are more and more commonly used worldwide. The objective of the present study was to describe the epidemiology, clinical and therapeutic features of poisoning with illicit drugs.

**Patients and methods/materials and methods:** It is a retrospective descriptive monocentric study conducted over a period of 8 years from August 2013 to December 2021 in a medical and toxicological intensive care unit.

**Results:** Twenty-three (23) patients were enrolled in the study with acute illicit drugs poisoning. The mean age was 27 ± 7 years with male predominance. Sixteen patients were drug addicts, only one had a psychiatric history. Fourteen patients were smokers and 13 were alcoholic. Two were known to have epilepsy, one had a history of attempted suicide and only one with a history of asthma. The intoxication was voluntary in 19 cases and accidental in 4 cases. The intoxications were divided according to the type of drug: ecstasy (n = 9), cocaine (n = 3), cannabis (n = 1), heroin (n = 1), buprenorphin (n = 1). We noticed one case of combination of cocaine and opiates (n = 1), three cases of combination of cannabis and cocaine (n = 3), one combination of cannabis and heroin (n = 1), one combination of cannabis and LSD (n = 1), one intoxication with heroin, ecstasy and cannabis (n = 1). In most cases, patients were admitted in the weekends (n = 13) and in the summer (n = 9), while no cases were observed on Monday. Neurological symptoms were predominant (n = 13). For ecstasy (n = 9), the clinical presentation was mainly adrenergic (n = 3) and serotonin syndrome (n = 3). Two patients developed hypokalemia with good outcome. Regarding cocaine overdose cases (n = 3), two patients had tachycardia. For the one case of cannabis intoxication and the other with buprenorphin poisoning, the main symptoms were neurological signs and they had a good outcome. The most common electrocardiographic abnormality (n = 8) was sinus tachycardia. The mean hospital length of stay was 33 ± 18 h. Twenty-two patients (22) were discharged from the hospital. We registered one case of death secondary to multiorgan failure after massive intoxication with cannabis, ecstasy and heroin.

**Conclusion:** Illicit drug intoxication is an emerging health problem in toxicology units in our country. Acute intoxication with illicit substances, as shown in this study, can be severe and fatal. Our results are similar to recent data in the literature.

**Compliance with ethics regulations:** Yes in clinical research.

## FC-117 Scorpion envenomation: predictive factors for intensive care admission

### Medhioub Kaaniche Fatma^1^, Zouari Farah^1^, Smaoui Ayoub^1^, Ben Khlifa Atraa^1^, Allala Rania^1^

#### ^1^Mahres Regional Hospital, Faculty of Medicine, University of Sfax, Sfax, Tunisie

##### **Correspondence:** Medhioub Kaaniche Fatma (fatma_kaaniche@yahoo.fr)

*Annals of Intensive Care* 2023, **13(Suppl 1):**FC-0117

**Rationale:** Scorpion envenomation is a fairly frequent reason for consultation in the emergency room. The Mahres delegation from the Sfax region is among the delegations where envenomation represents a real social scourge. The aim of this study is to analyse the population admitted in the emergency department for scorpion envenomation and to determine the predictive factors for intensive care admission.

**Patients and methods/materials and methods:** This is a prospective observational study, conducted in the emergency department during 24 months (01/01/2021–31/12/2022). We included all emergency consultants for scorpion envenomation.

**Results:** Among the 178 included patients, mean age was 34 ± 21 years, a sex ratio male/female of 0.89. The mean time to treatment was 1 h 20 min ± 27 min. Pain at the bite site was the most common sign (96.6%). Eight patients (4.4%) presented with hypertension and 2 patients were admitted in a state of shock (1.1%). Four patients presented an acute pulmonary edema (2.2%). Repolarization disorders on electrocardiogram were observed in 17 patients (45.9%). Priapism was noted in 20 patients (11.2%). Glycemia was in mean 7.2 ± 1.2 mmol/l. Hypernatremia was observed in 2 patients, hyperprotidemia in 5 patients and positive troponin Ic in 2 cases (33.3%). Most of the patients (83%) were considered as stage 1 in severity, twenty-five as stage 2 (14%) and 4 patients as stage 3 (2.2%). Home discharge was possible for 155 patients (87%). Twenty-three patients (12.9%) were hospitalized in intensive care. Age > 15 years (p = 0.04) and nocturnal consultation were predictive factors for hospitalization (p = 0.01). Sweating, vomiting, dyspnea, chest pain and agitation were significantly more frequent in hospitalized patients (p < 0.05). Shock, crackles, acute pulmonary edema and priapism were also predictors of hospitalization (p < 0.05). Hyperprotidemia and positive Troponin Ic assay were significantly more frequent in hospitalized patients (respectively p = 0.03 and p = 0.02).

**Conclusion:** Scorpion envenomation remains a summer danger, especially in rural areas. Efforts should be maximized by creating an envenomation management unit during the hot period.

**Compliance with ethics regulations:** N/A.

## FC-118 Ecstasy abuse: a 2-years experience in intensive care unit

### Nabil Sidi Aissa^1^, Mourad Goulmane^1^, Khadidja Reziga^1^, Nabil Tabet Aoul^1^

#### ^1^CHU Oran, Oran, Algerie

##### **Correspondence:** Nabil Sidi Aissa (sidiaissa_nabil@yahoo.fr)

*Annals of Intensive Care* 2023, **13(Suppl 1):**FC-0118

**Rationale:** The 3.4-methylenedioxymethamphetamine (MDMA), known as ecstasy, is a recreational drug becoming more and more popular among adolescents and young adults. Ecstasy use has increased significantly in ALGERIA. In this study, we aim to describe the clinical characteristics and outcomes of ecstasy abuse among patients admitted in the ICU.

**Patients and methods/materials and methods:** This is a retrospective observational study conducted in an Algerian ICU from 1st January 2021 to 31 December 2022. All consecutive patients admitted for acute MDMA poisoning were included. Baseline demographics, clinical presentation, and lab findings were recorded. A univariate descriptive analysis was performed.

**Results:** During the study period, 44 patients were included (median age 25 [21–29] years, mainly males (73%)]. About 55% of patients had a history of drug abuse: alcohol 60% and 4 (15%) patients had a history of psychological disorder and 7% had no comorbidities. MDMA was found in the urine in 22 (50%) patients. A co-ingestion of drugs was noted in 40% of cases: THC 10%, trihexyphenidyl (6%), opioids (30%), and cocaine and lorazepam each in one case. The intervals between ingestion and emergency department admission and then to the ICU were 4 [3–6] and 5 [4–9.5] hours, respectively. Five (11.36%) patients required invasive mechanical.

**Conclusion:** Although mortality rates are relatively low, MDMA abuse can be responsible for serious and life-threatening complications requiring ICU management.

**Compliance with ethics regulations:** Yes in clinical research.

## FC-119 Serious baclofen poisoning: a 10-year Tunisian ICU experience

### Amira Ben Jazia^1^, Salma Ghalloussi^1^, Hassen Ben Ghezala^1^, Mariem Cheikhrouhou^1^, Salma Essghaier^1^, Boudour Ben Dhia^1^, Ons Ellouze^1^, Nozha Brahmi^1^

#### ^1^CAMU, Tunis, Tunisie

##### **Correspondence:** Salma Ghalloussi (salmaghalloussi93@gmail.com)

*Annals of Intensive Care* 2023, **13(Suppl 1):**FC-0119

**Rationale:** Baclofen poisoning is rarely reported. It is increasing since the use of baclofen for alcohol withdrawal. The objective of our study is to describe the clinical features and prognosis of baclofen intoxications admitted in our intensive care unit.

**Patients and methods/materials and methods:** In this retrospective observational study, we collected data from patients who were hospitalized in the ICU for acute baclofen intoxication from September 2013 to November 2022. We collected epidemiological, clinical, paraclinical, therapeutic and prognostic data.

**Results:** During the study period, 53 patients (66% female) presented with baclofen intoxication with a mean age of 28 years ± 14. 52 patients were voluntarily intoxicated and only one patient was accidentally overdosed on chronic renal failure. A history of depression was noted in 11.3% of patients, while 49.1% had no known psychiatric history. 23 patients (43.4%) were mono-intoxicated. The median supposed quantity ingested was 300 mg [40, 1950]. Neurological signs were prominent with coma (GCS 7 ± 3) and agitation in 16 patients (32.1%), convulsions in 9 patients (17%) and delirium in 2 patients. Osteotendinous reflexes were absent in 16 patients (30.2%). Both myosis (30%) or mydriasis (24.5%) was observed. Bradycardia was noted in 54.7% of patients with BAV in two patients and prolonged QT in two patients. Other clinical signs included hypotension (n = 4), bradypnea (n = 12), vomiting (n = 9) and hypothermia (n = 17). thirteen patients had metabolic acidosis and rhabdomyolysis (24.5%). Mechanical ventilation was required in 43 patients with a median duration of 3 days [1, 16]. Weaning from the ventilator was associated with agitation in 16 patients and delirium in 6 patients. Complications observed were aspiration in 22 patients, ventilator-acquired pneumoniae in 5 patients and shock in 3 patients. Evolution was favorable in all cases. The median length of stay was 4 days [1, 14].

**Discussion:** The incidence of voluntry baclofen intoxication has increased in the last decade since its use in alcohol withdrawal. CNS depression is at the forefront, ranging from drowsiness to coma, as well as various cardiovascular disorders with frequent recourse to VMI. The treatment is essentially symptomatic with no existing antidote and the prognosis is favorable.

**Conclusion:** Acute baclofen intoxications in the ICU are characterized by neurological signs which include calm coma and agitation. Recognizing them facilitates the diagnosis. The intoxication is often poly-drug related. The concomitant management of other associated drugs must be systematically considered.

**Compliance with ethics regulations:** Yes in clinical research.

## FC-120 Acute insulin intoxication in the ICU: a retrospective study

### Maroua Jemii^1^, Hassen Ben Ghezala^1^, Amira Ben Jazia^1^, Nozha Brahmi^1^

#### ^1^Centre Mahmoud Yaacoub d'assistance médicale urgente de Tunis, Tunis, Tunisie

##### **Correspondence:** Hassen Ben Ghezala (hassen.ghezala@gmail.com)

*Annals of Intensive Care* 2023, **13(Suppl 1):**FC-0120

**Rationale:** Insulin is a protein hormone produced by the pancreas and is necessary in certain situations of diabetic patients since it is the only hypoglycemic hormone secreted in the human body. Insulin intoxication is underreported in recent literature. We report in this work the experience of the Tunisian national poison center for this intoxication.

**Patients and methods/materials and methods:** Single-center descriptive and observational retrospective study that included all patients hospitalized for insulin intoxication at the polyvalent and toxicological critical care unit over a 10-year period (2013–2022). Insulin intoxication was defined as flollows: injection of a supra-therapeutic quantity in a diabetic or injection of insulin in a non-diabetic patient, with a blood glucose level at the finger on admission < 0.7 g/L.

**Results:** During the study period, 61 patients were admitted for insulin intoxication: 20 patients (32.8%) presented with intermediate insulin intoxication, 14 patients with rapid insulin (23%), 8 patients with analogues (13%) and 19 patients (31%) had mixed intoxication with two types of insulin. The mean age was 32 ± 12 years with a sex ratio of 1.65. Diabete mellius was present in 26 patients (43%) and they were all treated with insulin. 39% of the patients were intoxicated with another associated treatment with a mean time of onset of hypoglycemia of 2.7 ± 2.5 h and a mean time of consultation to the emergency room of 6 ± 5.7 h. Intoxication was voluntary in 60 cases and one patient presented with insulin overdose. Psychiatric disorder was present in 30 patients (49%) with recurrence of suicide in 23 patients (38%). The mean dose of self-injected insulin was 295 ± 385 IU with a mean admission blood glucose level of 0.46 ± 0.25 g/L. The initial manifestations of intoxication included sweating in 39 patients (64%), tremors in 18 patients (29%), agitation with anxiety in 10 patients (16%). Confusion was present in 16 patients (26%), seizures in 7 patients and 10 patients (16%) had a coma, with 5 cases of coma who required intubation and mechanical ventilation. CT Brain showed only one case of cerebral edema. One patient had brain MRI which showed a laminar cortical necrosis. The average amount of glucose received to correct hypoglycemia was 363 ± 381.5 g and the average duration of hypoglycemia was 26 ± 18 h. Two patients presented severe neurological sequelae.

**Conclusion:** Insulin intoxication is rare but remains serious and life-threatening because of the risk of heavy neurological sequelae.

**Compliance with ethics regulations:** Yes in clinical research.

## FC-121 Coumarin poisoning in the toxicology intensive care unit: a descriptive case series study

### Mariem Cheikhrouhou^1^, Hassen Ben Ghezala^1^, Amira Ben Jazia^1^, Nozha Brahmi^1^

#### ^1^Centre Mahmoud Yaacoub d'assistance médicale urgente de Tunis, Tunis, Tunisie

##### **Correspondence:** Hassen Ben Ghezala (hassen.ghezala@gmail.com)

*Annals of Intensive Care* 2023, **13(Suppl 1):**FC-0121

**Rationale:** Coumarin poisoning is a rare and under reported intoxication in recent years. However, it can be a serious and life-threatening condition. The aim of our study was to determine the demographic, clinical and outcome of this intoxication.

**Patients and methods/materials and methods:** This is a retrospective descriptive single-center study conducted over an 8-year period from January 1, 2014, to December 31, 2022. All patients with coumarin poisoning were included. Demographic, clinical, biological and outcomes data of these patients were recorded from the national reference poison center critical care unit records.

**Results:** Sixty-five patients were included in our study. There was a decline in the incidence of coumarin poisoning from 2014 to 2022. The mean age was 29 ± 12 years and the sex ratio was 0.6 with a female predominance. Most of the patients had a history of psychiatric disorders (N = 17; 26.1%), diabete mellitus (N = 7; 10.7%) and hypertension (N = 6; 9.2%). In 18 cases, the patient was treated for valvular heart disease (N = 6; 9.2%), rhythmic heart disease (N = 5; 7.7%) and deep vein thrombosis (N = 6; 9.2%). Intentional intoxication was noted for all patients, 57 cases or 88% were acenocoumarin intoxications and 8 cases or 12% were coumarin rodenticide intoxications. It was a muti-drug intoxication for 56.9% of patients (N = 37). The median ingested dose was 50 mg [8, 240] for acenocoumarin and 15 g [5; 50] for rat poison. The average time of ER consultation was 16 + 12 h. Activated charcoal was administered in 3 patients and 2 patients had gastric lavage. Most of our patients were asymptomatic on admission, 15% of cases (N = 10) had abdominal pain and 12% of cases (N = 8) presented bleeding. The mean INR was 4.2 [1; 25.7] and a mean PT of 37.6% [7.3; 94]. Vitamin K was administered in 44 patients (67.7%). Active bleeding was observed in 5 patients (7.7%) without hemodynamic instability, 2 patients had PPSB and only 1 patient was transfused. The average length of stay was 2 days [1, 71]. Most of the patients recovered without any sequelae with a good outcome, but one patient died.

**Conclusion:** Coumarin intoxication is decreasing and this can be explained by the introduction of new direct oral anticoagulants. The outcome is usually good if it is managed in time. Critical care admission is indicated for symptomatic patients for whom acenocoumarin is their usual treatment.

**Compliance with ethics regulations:** Yes in clinical research.

**Figure 1 (abstract FC-121):**
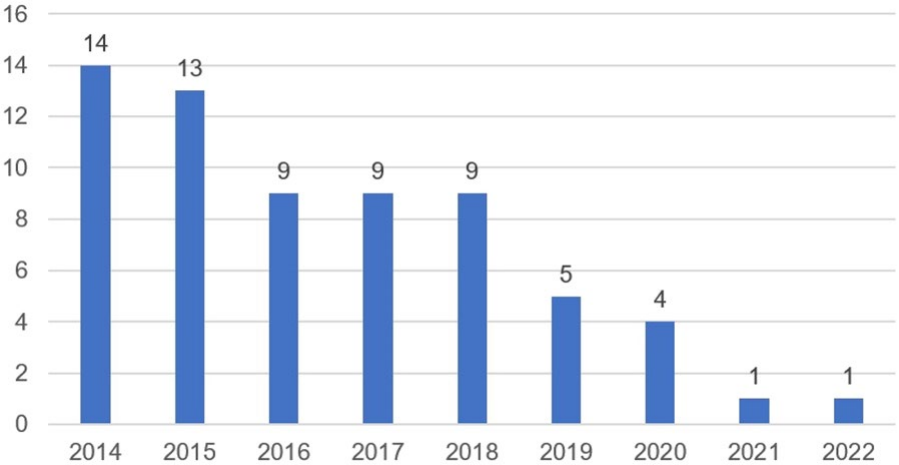
Trend of coumarin poisoning over years

## FC-122 Psychological impact of the COVID-19 outbreak among intensive care unit healthcare professionals

### Hend Zorgati^1^, Haifa Sfar^1^, Yosri Ben Ali^1^, Dhouha Ben Braiek^1^, Sourour Bel Haj Youssef ^1^, Nadine Boukadida^1^, Rahma Ben Jazia^2^, Amani Kacem^2^, Jihene Ayachi^1^

#### ^1^Medical Intensive Care Unit, Ibn El Jazzar University Hospital, Kairouan, Tunisie; ^2^Pulmonology Department, Ibn El Jazzar University Hospital, Kairouan, Tunisie

##### **Correspondence:** Jihene Ayachi (ayachijihen@gmail.com)

*Annals of Intensive Care* 2023, **13(Suppl 1):**FC-0122

**Rationale:** During the Covid-19 outbreak, health care systems have been overwhelmed and the health care professionals (HCP) especially in intensive care unit (ICU) are overworked and exposed to multiple risks impacting their physical and psychological wellbeing. Aim: To assess the psychological impact of COVID-19 outbreak among ICU HCP.

**Patients and methods/materials and methods:** A cross-sectional study was conducted in September 2022 in a medical ICU at a university hospital. HCP were voluntary invited to fulfill questionnaire evaluating the psychological impact of COVID-19 using the depression anxiety stress scale-21 (DASS-21), impact of event scale revised (IES-R) and Maslach Burn out Inventory (MBI), respectively. The Impact of Event Scale-Revised (IES-R was defined as more than 36/88 for post-traumatic stress disorder (PTSD).

**Results:** Twenty-seven HCP completed the questionnaire. Main characteristics of the participants were: median age 30 [27–33]; female 16 (59.3%); they had any comorbidity; 7 (25.9%) were smokers and reported an increase of tobacco use and 11 (40.7%) were married. Twenty-three participants (85.2%) were infected by COVID-19 disease and 24 (88.9%) had parents infected with COVID-19. Eighteen HCP found a high workload during the COVID-19 outbreak. Mild depression, anxiety and stress were noted in 10 (37%), 5 (17.2%) and 9 (33.3%) respectively. Extremely severe depression, anxiety and stress were reported in 6 (22.2%), 10 (34.5%) and 1 (3.7%) respectively. The mean scores in emotional exhaustion, depersonalization and lack of personal accomplishment were 11.5 ± 5.5, 7.5 ± 3.9 and 12.8 ± 5.8. Among the participants 11 (40.7%) had PTSD.

**Conclusion:** The current study showed that COVID-19 outbreak impacted the mental health ICU HCP. Early screening, communication and support program is needed to improve the long-term impact on ICU HCP’s mental health.

**Compliance with ethics regulations:** Yes in clinical research.

## FC-123 Post-traumatic stress disorder and burnout among ICU professionals during health crisis: cumulative effect

### Alicia Fournier^1^, Victoire Deltour^1,3^, Anne-Laure Poujol^3,4,5^, Florent Lheureux^6^, Fiona Ecarnot^13^, Christine Binquet^8,12^, Jean-Pierre Quenot^7,8,9,10,11^, Alexandra Laurent^1,2^

#### ^1^Université de Bourgogne, Psy-DREPI Laboratory, Dijon, France; ^2^Service de réanimation chirurgicale, CHU Dijon, Dijon, France; ^3^VCR EA 7403, Ecole de Psychologues Praticiens, ICP, Paris, France; ^4^APEMAC EA 4360, Université de Lorraine, Vandoeuvre Les Nancy, France; ^5^Service de réanimation chirurgicale polyvalente, Groupe Hospitalier Pitié Salpêtrière, AP-HP, Paris, France; ^6^Université de Franche-Comté, Laboratoire de psychologie, Besançon, France; ^7^Service de Médecine Intensive-Réanimation, CHU Dijon-Bourgogne, Dijon, France; ^8^Inserm CIC1432, module Epidémiologie Clinique (CIC-EC), Centre Hospitalier Universitaire, Dijon, France; ^9^Centre d’Investigation Clinique, Module Epidémiologie Clinique/Essais Cliniques, Dijon, France; ^10^Equipe Lipness, Centre de Recherche INSERM UMR1231 et LabEx LipSTIC, Université de Bourgogne, Dijon, France; ^11^INSERM, Espace de Réflexion Éthique Bourgogne Franche-Comté (EREBFC), Dijon, France; ^12^Inserm UMR 1231 "Lipides, Nutrition, Santé (LNC)", Université de Bourgogne, Dijon, France; ^13^Département de cardiologie, CHU de Besançon, et EA3920, Université de Franche Comté, Besançon, France

##### **Correspondence:** Alicia Fournier (alicia.fournier@u-bourgogne.fr)

*Annals of Intensive Care* 2023, **13(Suppl 1):**FC-0123

**Rationale:** The COVID-19 health crisis multiplied the stress factors in the ICU, confronting professionals with both acute and chronic stress related to the intensity and duration of the crisis. The objective of this study is to identify the impact of these acute and chronic stressors on the mental health of professionals 1 year after the first epidemic wave.

**Patients and methods/materials and methods:** An online questionnaire was administered, via the Lime survey platform, 1 year after the first wave of COVID-19. It included 3 scales to measure: the severity of post-traumatic stress states—PTSD-(IES-R), burnout (MBI-HSS) and perceived stressors (PS-ICU). A total of 1108 professionals (nurses' aides, nurses, nursing managers, residents, physicians) in 77 French hospitals participated in the PsyCOVID-ICU study.

**Results:** Of all the professionals, 318 (28.7%) had burnout, 34 (3.1%) had PTSD and 182 (16.4%) had both PTSD and burnout. All professionals with burnout and/or PTSD had higher perceived stress than those without. Professionals with PTSD and those with concurrent PTSD and burnout reported experiencing a difficult life event during the health crisis period.

**Conclusion:** This study, 1 year after the health crisis, shows a double vulnerability among ICU professionals at the level of trauma and burnout. The implementation of support should therefore be considered according to different psychological care modalities: one related to the development of traumatic care situations and the other related to a loss of meaning and professional accomplishment.

**Compliance with ethics regulations:** Yes in clinical research.

## FC-124 Burnout post-pandemic in ICU health-care workers in Tunisia

### Boudour Ben Dhia^1^, Najla Ben Slimene^1^, Fatma Essafi^1^, Khaoula Ben Ismail^1^, Moez Kaddour^1^, Imen Talik^1^, Takoua Merhabene^1^

#### ^1^Hôpital régional de Zaghouan, Tunis, Tunisie

##### **Correspondence:** Najla Ben Slimene (najlabenslimene@gmail.com)

*Annals of Intensive Care* 2023, **13(Suppl 1):**FC-0124

**Rationale:** The COVID-19 pandemic has certainly taken a toll on the health system all over the world during the last 2 years. In Tunisia, due to the limited ressources and ICU beds capacities for COVID-19 patients, health-care workers had to double their efforts facing a lot of personal and professional challenges. The aim of our study was to assess prevalence of burnout in health-care workers after the COVID-19 pandemic.

**Patients and methods/materials and methods:** We conducted a multi-centric cross-sectional survey including ICU health-care workers in Tunisia. Age, gender, marital status, having children, job category, experience as well as the level of burnout were measured. Maslach Burnout Inventory (MBI) was used to determine the risk of burnout by exploring 3 components: exhaustion, depersonalization and personal achievement.

**Results:** One hundred and seventeen ICU health-care workers responded to the survey. They were doctors in 52.1% with a female predominance (75.9%). They were mainly between 25 and 35 years old. Only 17.9% had a history of chronic disease and 23.5% had a history of depression. They were married in 47.4% of the cases, with children in 46.3% and living alone in 17.1%. Thirty-eight percent of health-care workers had work experience between 3 and 5 years. They had to change homes due to the pandemic in 25%. Almost 95.7% took night shifts and they had their first contact with a COVID-19 patient between March et June 2020. Most of them quarantined (83.5%) and confinement was mandatory in 63% of the cases. Sixty-seven percent contracted the virus while working, among them 37.3% were considered as occupational disease. Fifty-three percent had physical or psychological damage after the infection. Out of 117 ICU health-care workers, 34.5% met the criteria for the burnout definition by the MBI. The results showed that 40.2 percent had a high degree of emotional exhaustion, 16.8 percent had a high degree of depersonalization and 27.2 percent had a high degree of lack of personal accomplishment.

**Conclusion:** Burnout is a serious syndrome of exhaustion that is underdiagnosed in health-care workers. Prevention is key by strengthening personal and organizational support systems.

**Compliance with ethics regulations:** Yes in clinical research.

## FC-125 Assessment of psychotrauma and quality of life after an ICU stay during the SARS-CoV-2 pandemic

### Miren Hermant^1^, Benjamin Kowalski^2^, Anne Guaguere^2^, Camille Trouillet^1^, Juliette Perche^1^, Lucile Martin^1^, Remy Diesnis^1^, Martine Nyunga^1^

#### ^1^CH Roubaix, Roubaix, France; ^2^CH Douai, Douai, France

##### **Correspondence:** Martine Nyunga (martine.nyunga@ch-roubaix.fr)

*Annals of Intensive Care* 2023, **13(Suppl 1):**FC-0125

**Rationale:** A stay in an intensive care unit can generate psycho-trauma for patients and their families. Numerous factors favoring the development of post-traumatic stress disorder (PTSD) and anxiety-depression syndrome (ADS) have been identified in the ICU. The SARS-CoV-2 pandemic has led to many changes with a significant psychological impact on families and caregivers. However, few data on these consequences in patients are available. The objective of this study is to evaluate the occurrence of psycho-trauma in patients infected or not with SARS-CoV-2 during the pandemic period after their discharge from the ICU.

**Patients and methods/materials and methods:** This is a prospective, analytical, bi-centric cohort study, conducted from October 2020 to March 2021, involving 2 groups of patients hospitalized in the ICU, infected or not with SARS-CoV-2. The primary objective was to evaluate the occurrence of suspected or onset of PTSD and ADS between 3 and 6 months after discharge from the ICU. Secondary objectives were to assess the impact on quality of life and the identification of risk factors for developing PTSD and ADS.

**Results:** A total of 168 patients were included, 108 in the covid group with a median age of 66.5y [56.75, 73.00], 67.3% male, median length of stay of 7 days [4.00, 11.25] and 32.1% of patients intubated. Between 3 and 6 months after discharge, 41.1% of patients had an IES-R ≥ 12, 49.4% a HADS-A ≥ 8 and 30.4% a HADS-D ≥ 8. In the covid group, 43.5% of patients presented an IES-R ≥ 12, 48.1% a HADS-A ≥ 8 and 27.8% a HADS-D ≥ 12 without significant difference found. Of note, 15.5% of patients had proven symptoms of PTSD (IES-R ≥ 33) and 18.5% in the covid group. Quality of life was globally impaired (PCS 38.7 [30.47, 45.56], MCS 44.8 [33.60, 54.46]). Independent risk factors for psychotrauma were young age, media-related stress, immunosuppression, readmission to the ICU, and lack of family.

**Conclusion:** No significant difference between the covid and non-covid groups was found. However, the occurrence of psychotrauma after an ICU stay remains high independently of the SARS-CoV-2 pandemic context, raising the interest of an early detection by a systematic follow-up of patients. Its impact on the quality of life encourages the optimization of medical care, the environment and especially a strong family support system to the patient.

**Compliance with ethics regulations:** Yes in clinical research.

**Figure 1 (abstract FC-125):**
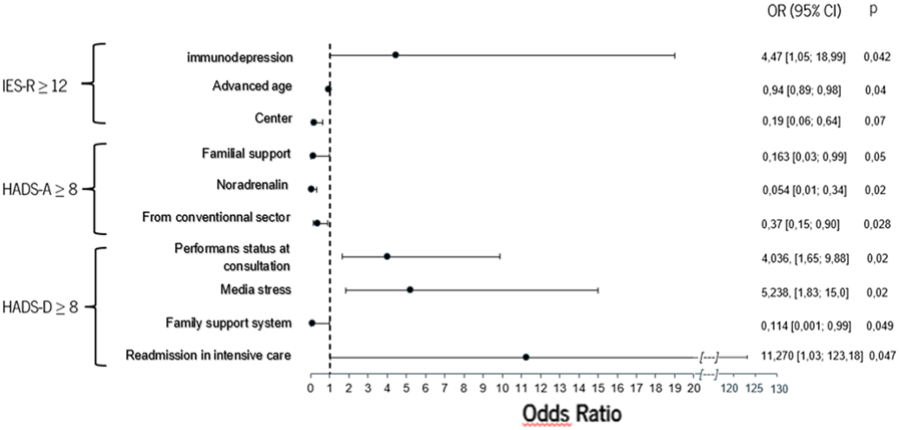
Independent risk factors for psychotrauma

## FC-126 Perceived inappropriate treatments in intermediate care units: association with burn-out, intention to leave, and with patients’ and units’ patterns

### Matthieu Evrard^1^, Mylien Nguyen Tan^3^, Hugues Georges^2^, Anne-Sophie Moreau^4^, Maxime Granier^5^, Erika Parmentier-Decrucq^4^, Caroline Varillon^6^, Martine Nyunga^7^, Olivier Nigeon^1^, Mélanie Verlay^1^, Hélène Behal^4^, Nicolas Van Grunderbeeck^1^

#### ^1^Centre Hospitalier de Lens, Lens, France; ^2^Centre Hospitalier de Tourcoing, Tourcoing, France; ^3^Centre Hospitalier de Valenciennes, Valenciennes, France; ^4^Centre Hospitalier Régional Universitaire de Lille, Lille, France; ^5^Centre Hospitalier d'Arras, Arras, France; ^6^Centre Hospitalier de Dunkerque, Dunkerque, France; ^7^Centre Hospitalier de Roubaix, Roubaix, France

##### **Correspondence:** Nicolas Van Grunderbeeck (nicovgdb9@orange.fr)

*Annals of Intensive Care* 2023, **13(Suppl 1):**FC-0126

**Rationale:** In Intensive Care Units, Perceived Inappropriate Treatments (PIT) have been associated with negative impact on caregivers, such as burn-out. Few data exist on Intermediate Care Units (IctdCU). Our study aimed to assess relationship between PIT of patients with life-supporting limitations, and burn-out or intention to leave of caregivers in this setting. Moreover, we studied the relationship between ItdCU structural characteristics and hospitalized patients data with burn-out or intent to leave of caregivers.

**Patients and methods/materials and methods:** An observational, multicentric retrospective study in 7 ItdCUs of public hospitals in the North of France from December 2017 to April 2018. An anonymous questionnaire of 28 items assessed working environment, burn-out, PIT, and intention to leave of caregivers. Furthermore, organizational characteristics of each ItdCUs were collected as well as hospitalized patient’s data including SOFA, Charlson, IGSII, frailty, admission pattern and type of therapeutic intervention.

**Results:** A hundred and ninety-seven questionnaires were analyzed (participation rate of 41.6%). In the univariate analysis, burn-out, PIT and intention to leave were greater in units where nurses´ teams solely worked in IdtCU. In multivariate analysis, perception of non-beneficial treatment of patients with life support withholding was associated with: bad collaboration with other units (OR 1.29; IC 95% [1.10–1.52]); burn-out state (OR 1.40; IC95% [1.22–1.60]). Favorable work relationship (between physicians and nurses 'teams) were associated with a lower perception of disproportionate treatments (OR 0.86; IC95% [0.75–0.98]). Having an adjoining ICU was associated with greater Intention to leave of caregivers. ItdCUs admissions for neurological or metabolic issues were associated with more burn-out and intent to leave. IGSII was significantly associated with more intentions to leave (RR de 1.14 IC95% [1.01–1.29]). Finally, a trend was shown between mortality and intent to leave, unless not statistically significant.

**Discussion:** This study presents several limits: lack of data on caregivers' responders to the questionnaire, low participation rate and a short study period. Nevertheless, PIT in IdtCU is linked to work ambience and burn-out. ItdCU organization and patients’ characteristics and severity are directly linked to caregivers’ moral distress.

**Conclusion:** Multidisciplinary teamwork and units’ interplay seem mandatory to improve practice and to achieve Goal-concordant cares for patients with life-supporting limitations. Better patients’ selection for ItdCU admissions seems necessary to prevent caregivers’ moral distress and to retain health care workers in the job.

**Reference 1**

Schwarzkopf D, Rüddel H, Thomas-Rüddel DO, Felfe J, Poidinger B, Matthäus-Krämer CT, Hartog CS, Bloos F. Perceived nonbeneficial treatment of patients, burnout, and intention to leave the job among ICU nurses and junior and senior physicians. Crit Care Me.

**Reference 2**

Van den Bulcke B, Metaxa V, Reyners AK, Rusinova K, Jensen HI, Malmgren J, Darmon M, Talmor D, Meert AP, Cancelliere L, Zubek L, Maia P, Michalsen A, Kompanje EJO, Vlerick P, Roels J, Vansteelandt S, Decruyenaere J, Azoulay E, Vanheule S, Piers R, Benoit.

**Compliance with ethics regulations:** Yes in clinical research.

## FC-127 ICU health care workers opinion on physician-assisted-suicide and euthanasia: a French survey

### Alexandre Boyer^1^, Mathieu Acquier^1^, Bertrand Guidet^2^, Alexandre Lautrette^3^, Jean Reignier^4^, Guillaume Thiery^5^, René Robert^6^

#### ^1^CHU Bordeaux, Bordeaux, France; ^2^APHP Hôpital Saint Antoine, Paris, France; ^3^Hôpital Montpied, Clermont Ferrand, France; ^4^CHU Nantes, Nantes, France; ^5^CHU Saint Etienne, Saint Etienne, France; ^6^CHU Poitiers, Poitiers, France

##### **Correspondence:** Alexandre Boyer (alexandre.boyer@chu-bordeaux.fr)

*Annals of Intensive Care* 2023, **13(Suppl 1):**FC-0127

**Rationale:** In France, physician-assisted suicide or euthanasia are not legal but are still debated. French intensive care unit (ICU) health care workers (HCWs) have an insider’s perspective on the global quality of the patient’s end-of-life, whether it occurs in ICU or not. However, their opinion about euthanasia/physician-assisted suicide remains unknown. The aim of this study is to investigate the opinion of French ICU HCWs about physician-assisted suicide/euthanasia.

**Patients and methods/materials and methods:** From February to May 2022, the head physicians of 290 French adult ICUs were contacted to distribute a self-administered anonymous questionnaire to all the HCWs of their unit. This questionnaire was developed by a group of 7 intensivists involved in the field of ethical issues. It was distributed by email with a google form link to answer the questionnaire. Briefly, after an introductive text eliciting the end-of-life historical perspective and definitions of euthanasia/physician-assisted suicide, the ICU HCWs were asked if they were satisfied with the end-of-life process framed in the Claeys-Leonetti law, if they would like or not a new law including euthanasia/physician-assisted suicide and if so, whether this law could improve the end-of-life process in the context of the ICU patients they care. Additionally, three vignettes of typical situations were developed and submitted to the same panel of questions: a patient with neurodegenerative disorder such as amyotrophic lateral sclerosis with swallowing disorders who refuse artificial feeding; a patient with prolonged coma related to severe brain injury with spontaneous ventilation fed by enteral nutrition; a patient with severe cognitive alteration. Finally, questions about modalities and potential limits of a law authorizing euthanasia/physician-assisted suicide were also tested.

**Results:** A total of 1149 ICU HCWs participated to a self-administered anonymous questionnaire: 411 (35.8%) physicians and 738 (64.2%) non-physicians. Among them, 76.5% indicated they were in favor of legalizing euthanasia/ physician-assisted suicide. Non-physicians HCWs were significantly more in favor of the legalization of euthanasia/physician assisted suicide than physicians (87% vs 57.8% p < 0.001). Euthanasia/physician-assisted suicide of an ICU patient raised the most important difference in positive judgment between physicians and non-physicians HCWs (80.3% vs 42.2%; p < 0.001 of non-physicians and physicians, respectively). The three case vignettes of concrete examples which participated to the increase in the rate of response in favor of euthanasia/physician-assisted suicide legalization (76.5% to 82.9%; p < 0.001).

**Conclusion:** Keeping in mind the unknown representation of our sample, ICU HCWs, particularly non physicians, would be in favor of a law legalizing euthanasia/physician-assisted suicide.

**Compliance with ethics regulations:** N/A.

## FC-128 Evaluation of Helge H10, a POC system for detection of hemolysis on whole blood samples

### Camille Chenevier-Gobeaux^1^, Morgane Ducastel^1^, Imane Dridi-Brahimi^1^, Julien Charpentier^1^, Didier Borderie^1^

#### ^1^Hôpital COCHIN, APHP, Centre-Université de Paris, Paris, France

##### **Correspondence:** Camille Chenevier-Gobeaux (camille.gobeaux@aphp.fr)

*Annals of Intensive Care* 2023, **13(Suppl 1):**FC-0128

**Rationale:** In-vitro hemolysis leads to plasmatic modification making plasma inappropriate for analysis such as potassium measurement (kalemia). Detection of hemolysis is of interest to evaluate sample pre-analytical quality. We aimed to evaluate the Helge H10, a point-of-care (POC) system for hemolysis detection, on whole blood samples from Emergency Department (ED) and critical care unit (CCU).

**Patients and methods/materials and methods:** Helge Hemolysis Index (HHI) was measured on 295 left-over whole heparinized blood samples (199 self-filling syringes, 96 tubes) obtained from ED and CCU. Delay between kalemia and HHI measurement (aging time) was collected. Kalemia obtained on whole blood syringes were measured on an ABL® analyzer and compared to those measured at the lab on a Cobas® analyzer on a matched non-hemolyzed plasma with < 12 h-delay in the collection time. Heparinized tubes were centrifuged for laboratory hemolysis index measurement on a Cobas® analyzer (LabHI). Hemolysis was declared if HHI or LabHI was > 90 (laboratory threshold, in mg/dL).

**Results:** Seventy-eight samples (26.4%) presented an HHI > 90. Hemolysis was higher in syringes (32.7%) than in tubes (13.5%, p < 0.001). When considering syringes, proportion of hemolyzed samples (1) was not different between arterial (29.6%) and venous collection (39.1%, p = 0.186), and (2) was higher on ED samples (46.0%) in comparison to CCU samples (21.0%, p = 0.004). HHI was correlated to LabHI (n = 81, r = 0.7560, p < 0.001); Passing-Bablok comparison is satisfying (y = 0.89x − 4.45). HHI was not correlated to the sample aging time (r = 0.033, p = 0.758). Kalemia measured on hemolyzed syringes presented a mean difference < 0.4 mmol/L in comparison to non-hemolyzed samples; in 12% of the observed cases, hemolysis led to a false normokaliemia or hyperkaliemia.

**Discussion:** We observed an elevated proportion of hemolyzed whole blood samples. This proportion was higher than observed in the literature [1]. Hemolysis could lead to a misinterpretation in 12% of cases. The Helge POC system is well correlated to the laboratory method of hemolysis detection.

**Conclusion:** The Helge system is a reliable tool for hemolysis detection on whole blood samples. Implemented in a care unit that uses routine measurement of kalemia on whole blood samples, this POC system might be useful to improve pre-analytical sample quality and results reliability. A supplementary study in CCU is needed to study the impact on patients’ care.

**Reference 1**

[1]. DiToro et al., Am J Clin Pathol XXXX 2022;XX:1–0, https://doi.org/10.1093/AJCP/AQAB217

**Compliance with ethics regulations:** N/A.

## FC-129 Implementation of a new teaching program including medical simulation for undergraduate medical students in intensive care unit (ICU): a case–control study

### Frédéric Martino^1^, Marie-France Petchy^1^, Pascale Piednoir^1^, Valérie Eugene^1^, Philippe Thomar^1^, Fanny Ardisson^1^, Marc Valette^1^

#### ^1^CHU de la Guadeloupe, Les Abymes, France

##### **Correspondence:** Frédéric Martino (frederic.martino@chu-guadeloupe.fr)

*Annals of Intensive Care* 2023, **13(Suppl 1):**FC-0129

**Rationale:** Learning medical semiology is basis for medical students during 2nd and 3rd years and one way to perform is by multiplying medical internships. Our faculty, the youngest of France and our islander situation reduce capacity of internship and moving for students, obliging to have 5-day internships. To optimize internship in ICU, we performed and evaluated a new complete teaching program including simulation for medical students.

**Patients and methods/materials and methods:** We included all students affected in our ICU from January to May 2022 (ICUstud). The teaching program included half welcome day, general teaching about medical files, and affectation to a unit of the ICU with daily objectives, accompanied by residents. High fidelity simulation training was performed with SimMan 3G™ the day before evaluation. Students were grouped by two or three and had to perform a medical examination, including patient history and objective clinical examination. Simulation included briefing, scenario and debriefing. During debriefing differences between normal and abnormal examination were highlighted (e.g. s listening to aortic insufficiency, pneumothorax) using the mannequin. Controls were students never affected to our ICU during 2nd or 3rd year (NoICUstud). They were asked to answer referring to the best internship they have ever had. All students received an anonymous questionary at the end of stage by email. It included satisfaction questions quoted from 1 (Not at all) to 5 (Totally satisfied) and Yes/No questions. A single reminder was performed for all of them.

**Results:** One hundred and three students were enrolled, including 51 ICUstud. In ICUmed, 41 (80%) were women. ICUstud were significantly more satisfied than NoICUstud concerning half welcome day and internship organization (respectively 5 (5–5) vs 4 (3–5), p < 0.0001 and 5 (4–5) vs 4 (3–4), p < 0.0001). All ICUstud (100%) appreciated the simulation session and 100% of NoICUstud would appreciate to benefit from this type of teaching. ICUstud were significantly more satisfied of the ICU internship (reception, organization, simulation, schedules) comparing to NoICUstud’s best internship [respectively 5 (5–5) vs 4 (3–4.8), p < 0.0001]. ICUstud also had a better felling of progression versus others [respectively 5 (4–5) vs 4(3–5) p = 0.0042]. Finally, 50 (98%) of ICUstud and 48 (92%) of NoICUstud would recommend this internship to other students of the faculty (p = 0.36).

**Conclusion:** A complete teaching program in ICU including bedside medical semiology and simulation is very useful, stimulating and appreciated by medical students. This new approach needs to be developed, particularly in isolated territories.

**Compliance with ethics regulations:** Yes in clinical research.

## FC-130 Impact of a computerized sign out tool on the quality of physician inter-shift handovers in the intensive care unit

### Matthieu Evrard^1^, Pauline Boddaert^3^, Saadala Nseir^2^, Anahita Rouze^2^

#### ^1^Centre Hospitalier de Lens, Lens, France; ^2^Univ. Lille, Inserm U1285, CHU Lille, Service de Médecine Intensive-Réanimation, CNRS, UMR 8576 – UGSF – Unité de Glycobiologie Structurale et Fonctionnelle, Lille, France; ^3^Centre Hospitalier de Dunkerque, Dunkerque, France

##### **Correspondence:** Matthieu Evrard (mevrard@ch-lens.fr)

*Annals of Intensive Care* 2023, **13(Suppl 1):**FC-0130

**Rationale:** Ineffective communication during handovers may lead to medical errors, harming critically ill patient quality of care and safety. The aim of our study was to determine the impact of a computerized sign-out tool on the quality of physician inter-shift handovers in the ICU, and on the rate of medical errors due to inadequate information handoff.

**Patients and methods/materials and methods:** Prospective single-center observational study, with a before-after design, performed in the ICU department of a French University Hospital, from July 2020 to March 2021. Handovers between day and night medical shift were monitored over 2 periods of 3 months, before and after an intervention including a computerized standardized sign-out tool and a dedicated training. A quality score, based on the presence of mistakes or omissions, was assigned to each written and oral handover. Participants’ experience and satisfaction as well as medical errors related to inadequate information handoff were collected. The handover quality score was compared between the 2 periods with a Wilcoxon test; and the medical error rate with a chi-square test or a Fischer’s exact test. Factors associated with oral handover quality score were determined by univariate and multivariate analysis.

**Results:** 395 and 327 transmissions were evaluated in the 1st and 2nd period, respectively. The quality score was significantly higher in period 2 (for oral handover, median score 27 [25; 28] vs 27 [26; 29], p = 0.011; for written handover, median score 26 [24; 28] vs 28 [26; 29], p < 0.001). There was no significant difference between the 2 periods in the rate of medical errors due to inadequate information handoff (10.4% in both periods, p = 0.99). Multivariate analysis only identified transmitter’s anxiety as negatively associated with good quality oral handover [OR (95% CI) 0.36 (0.16–0.80), p = 0.012]. The rate of medical errors was significantly lower when the oral handover quality score was above median [RR (95% CI) 1.12 (1.07–1.18), p < 0.001].

**Conclusion:** Physician’s training and implementation of a computerized standardized sign-out tool improved the quality of oral and written handovers but was not independently associated with the quality of oral handovers. The rate of medical errors due to inadequate information handoff was comparable between the 2 periods, but significantly associated with oral handover quality score.

**Compliance with ethics regulations:** Yes in clinical research.

## FC-131 Ability of Tunisian medical intensive care physicians to identify patient-ventilator asynchrony using waveform analysis

### Rym Chelbi^1,2^, Farah Thabet^3^, Emna Ennouri^1,2^, Nabil Bouguezzi^1,2^, Radhouene Toumi^1,2^, Rihab Rajah^1^, Azer Yacoub^1,2^, Khaoula Meddeb^1,2^, Mohamed Boussarsar^1,2^

#### ^1^University of Sousse, Faculty of Medicine of Sousse, Sousse, Tunisie; ^2^Farhat Hached University Hospital, Medical Intensive Care Unit, Research Laboratory “Heart Failure”, LR12SP09, 4000, Sousse, Tunisia, Sousse, Tunisie; ^3^Fattouma Bourguiba university hospital, pediatric department, Monastir, Tunisie

##### **Correspondence:** Rym Chelbi (rymchelbi.chelbi2@gmail.com)

*Annals of Intensive Care* 2023, **13(Suppl 1):**FC-0131

**Rationale:** Patient-ventilator asynchrony (PVA) is known to be associated with poor clinical outcomes. Therefore, improving the ability of intensivists to identify and manage PVA may decrease its incidence and thus improve patients’ outcomes. This study aimed to assess the ability of Tunisian intensivists to identify patient-ventilator asynchronies (PVA) using waveform analysis and to assess factors associated with this knowledge.

**Patients and methods/materials and methods:** This survey was conducted in 12 university-affiliated medical intensive care units (MICUs) in Tunisia. Intensivists practicing in those MICUs were asked to respond to a survey containing 4 clinical cases each one corresponding to a different PVA. The intensivists were described according to grade, years of experience, prior training in mechanical ventilation, the assessment of waveform ventilator in their daily clinical practice, characteristics of MICUs they were practicing in. Responders were categorized into two groups according to their ability to correctly identify PVA. Univariate and multivariate analyses were performed to identify factors associated to this knowledge.

**Results:** Seventy-two (52.9%) out of 136 intensivists responded to the present survey. Responders were rather, residents, 59 (81.9%) than senior physicians, 13 (18.1%); experienced, 9 (12.5%); less experienced, 63 (87.5%); trained, 50 (69.4%); non trained, 21 (29.2%). The rate of proper recognition of patient-ventilator asynchronies was 29.2%. Multivariate analysis identified senior physicians to have a better ability than residents to correctly identify PVAs (OR = 4.4, 95% CI [1.20–16.07], p = 0.025). Prior training in mechanical ventilation was not associated with a better ability to identify PVA.

**Conclusion:** The present survey demonstrated a low rate of recognition of PVA among university affiliated MICUs physicians. Senior physicians were more able than residents to correctly identify PVA.

**Compliance with ethics regulations:** N/A.

## FC-132 Simulation learning by videolarygoscopy in the emergency departement. Factors predicting intubation faillure among young doctors

### Badra Bahri^1^, Kharraz Taycir^1^, Tahar Kilani^1^, Aymen Zoubli^1^, Hela Manai^1^, Hanene Ghazali^1^, Nebiha Falfoul^1^

#### ^1^Hôpital Habib Thameur, Tunis, Tunisie

##### **Correspondence:** Badra Bahri (bahribadra@gmail.com)

*Annals of Intensive Care* 2023, **13(Suppl 1):**FC-0132

**Rationale:** Learning by procedural simulation takes an important place in teaching techniques in the emergency departement (ED). Orotracheal intubation by videolaryngoscopy (OTI) is becoming an essential skill to acquire and has demonstrated its usefulness during the covid-19 pandemic. The purpose of our study was to evaluate the predictive factors of intubation failure by videodeolaryngoscopy among young doctors practicing in the ED.

**Patients and methods/materials and methods:** This was a monocentric prospective study including residents practicing in the ED and novices in direct laryngoscopy. We scheduled a procedural simulation session whose theme was announced in advance. We carried out a theoretical and practical evaluation before and after the session. The primary endpoint was the rate of intubation failure after learning session defined by the presence of the following two criteria: a time elapsed between the insertion of the blade and the verification of the correct positioning of the intubation tube more than 120 s or esophageal intubation. Selective intubation was considered as a successful intubation in this study.

**Results:** Thirty-two residents were included. The mean age was 28 ± 2.5 years. Sex ratio at 0.18. The mean exercise seniority was 1.53 ± 0.7 years. 63% of learners were in their first year of training. The rate of intubation failure before and after the training was n (%): 28 (87.5) vs 9 (28%) after the training (p = 0.02) with an average time of OTI (seconds) respectively of 149.81 ± 108 s vs. 51 ± 96 s (p = 0.005). The number of esophageal OTI was: n (%) of 16 (50) vs 9 (28) (p = 0.49) and the mean number of attempts was 3.81 vs 1.84 (p = 0.08) before and after training. In multivariate analysis Intubation time is a predictive factor of intubation failure (p = 0.001); OR IC 95% = 1.44 [1.18–10.69]. Another factors analyzed but found not statistically significative (sex p = 0.52; specialty; p = 0.86; OTI failure before training p = 0.882; compliance with different steps; p = 0.96).

**Conclusion:** Learning by procedural simulation in the ED has led to a significant reduction in the time required for OTI. Intubation time is an independent factor predicting intubation failure among young doctors learning intubation by video laryngoscopy.

**Compliance with ethics regulations:** N/A.

## FC-133 Pharmaco-therapeutic problems: an often unknown iatrogenesis

### Hana Fredj^1^, Amal Aloui^1^, Sabrine Ben Hammouda^1^, Sarra Zarrouk^1^, Imen Jami^1^, Bahija Gasri^1^, Manel Ben Saad^1^, Meriem Gargouri^1^, Amal Mokline^1^, Amen Allah Messadi^1^

#### ^1^Centre de Traumatologie et des Grands Brûlés, Ben Arous, Tunisie

##### **Correspondence:** Hana Fredj (fredjhana@yahoo.fr)

*Annals of Intensive Care* 2023, **13(Suppl 1):**FC-0133

**Rationale:** In intensive care, the clinician is often led to make a multi-medication prescription, which can generate a pharmaco-therapeutic problem (PTP) that can compromise the effectiveness of the treatment. The aim of this study was to identify the different PTP that have occurred in the Burn Intensive Care Unit through the analysis of medical prescriptions by a clinical pharmacologist.

**Patients and methods/materials and methods:** This was a prospective study conducted in the burn intensive care unit at the Traumatology and Burns Center in Tunis, during a period of 4 months (October 2021–January 2022). All prescriptions were analyzed by a clinical pharmacologist. For each PTP identified, a pharmaceutical intervention (PI) was proposed to the prescriber. PTP are defined by the occurrence of one of these events: physical or chemical incompatibility, drug interaction, side effect, dosing problem, or administration route problem. This classification was developed by referring to the recommendations of the French Society of Clinical Pharmacology.

**Results:** During this period, 143 patients were admitted, with an average age of 35 ± 10 years, the sex ratio was 3, and the average burned skin area was 38%. The analysis of 572 prescriptions identified 32 PTP (5.5%). These PTP were related to a problem of physico-chemical incompatibility in the majority of cases (62%, n = 20). Physical incompatibility was observed in 72% of cases (n = 14), with acid–base reactions (50%, n = 7) predominating, followed by a solubility problem (28%, n = 4), leading to precipitation of products in the circuits in all cases. Chemical incompatibilities (n = 9) were observed in 28% of cases, mainly related to a change in the PH of the administered products (90%, n = 8). The second PPT encountered was drug interaction (16%, n = 5) (Figure 1). The drugs most frequently involved in PTP were antibiotics in 75% (n = 24) of cases, followed by sedatives in 18% of cases (n = 6). PI was accepted by physicians in 90% of cases.

**Conclusion:** In our study, the occurrence of PTP was frequent. The PTP was mainly related to physico-chemical incompatibility, especially for antibiotics. The collaboration between the physician and the pharmacist would thus optimize the medical prescription.

**Reference 1**

1-J.M. Bright, P.C. Tenni, The clinical services documentation (CSD) system for documenting clinical pharmacists’ services, Aust J Hosp Pharm, 30 (2000), pp. 10–15.

**Compliance with ethics regulations:** Yes in clinical research.

**Figure 1 (abstract FC-133):**
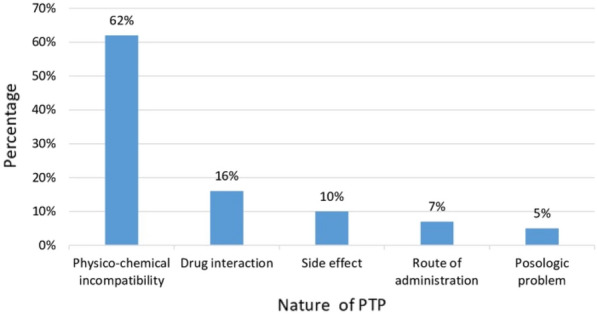
Nature and frequency of encountered pharmaco-therapeutic problems

## FC-134 Usefulness of a second course of systemic corticosteroids in patients mechanically ventilated for acute respiratory failure due to severe SARS-CoV2 pneumonia

### Laurent Truffaut^1^, Simon Van Migem^1^, Christine Colienne^1^, Virginie Montiel^1^, Xavier Wittebole^1^, Jean-Baptiste Mesland^1^, Philippe Hantson^1^, Ludovic Gerard^1^

#### ^1^Cliniques Universitaires Saint Luc, Bruxelles, Belgique

##### **Correspondence:** Laurent Truffaut (laurent.truffaut@hotmail.com)

*Annals of Intensive Care* 2023, **13(Suppl 1):**FC-0134

**Rationale:** Early use of corticosteroids has been associated with improved outcomes in patients with severe SARS COV 2 pneumonia who required respiratory support and has therefore become standard of care1. However, it is not known whether a second course of systemic corticosteroids in patients with refractory respiratory failure due to SARS-CoV2 pneumonia can provide any additional benefit.

**Patients and methods/materials and methods:** We conducted a retrospective single-center observational study in a tertiary center in Brussels, Belgium. Patients who underwent invasive mechanical ventilation for acute respiratory failure due to SARS-CoV2 pneumonia between June 15, 2020 and February 25, 2022 were included in the analysis. All patients fulfilled ARDS criteria and had been initially treated with dexamethasone. Among those, a subgroup of patients in whom a second course of corticosteroids was given for refractory respiratory failure was identified.

**Results:** During the study period, 151 patients who had been treated with dexamethasone underwent invasive mechanical ventilation for severe SARS-CoV2. A second line of steroids was initiated for refractory respiratory failure in 28 patients. Patients who were given two courses of corticosteroids were younger than patients with usual care (62.6 vs 56.3; p 0.013). However, no significant difference between the two groups was found regarding other demographic variables, severity, biological values at ICU admission, or adjunctive treatments. The second course of corticosteroids was initiated 15 (IQR 10–21) days after ICU admission and lasted a median of 12 (5–30) days. As shown in Figure 1, there was no statistically significant difference regarding 180 days mortality, (68.3 s 67.9; log-rank p = 0.22). Besides, no significant difference was observed regarding respiratory parameters, disease severity and ventilator settings before and after the initiation of the second course of corticosteroids.

**Conclusion:** In this retrospective analysis of patients who required invasive mechanical ventilation for acute respiratory failure due to severe SARS-CoV2 pneumonia and who had been initially treated with dexamethasone, the addition of a second course of corticosteroids for refractory respiratory failure was not associated with any relevant clinical benefit.

**Reference 1**

RECOVERY Collaborative Group. (2021). Dexamethasone in hospitalized patients with Covid-19. New England Journal of Medicine, 384(8), 693–704.

**Compliance with ethics regulations:** Yes in clinical research.

**Figure 1 (abstract FC-134):**
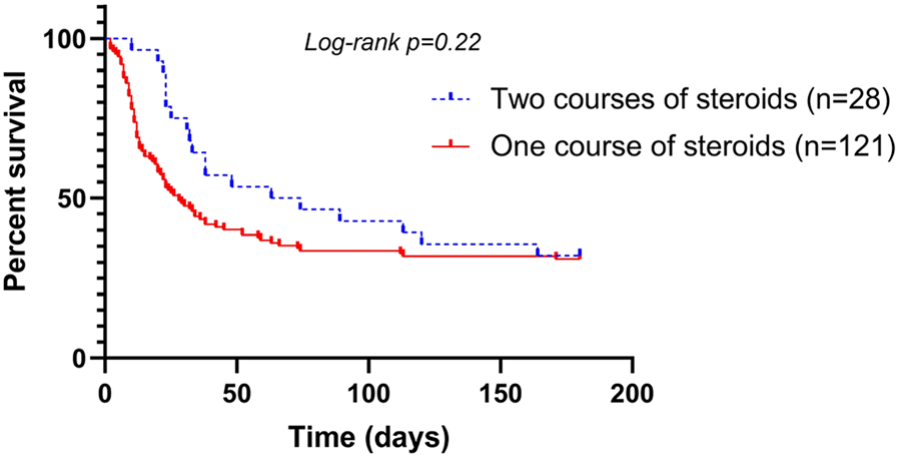
180 days mortality curves

## FC-135 Long haul air transfer preparation and impact on patient’s survival during Covid-19 pandemic

### Hossein Mehdaoui^1^, Bertrand Dubois^1^, Marie Sabia^1^, Fréderic Martino^2^, Hatem Kallel^3^, Shazima Vally^1^, Diane Paris^1^, Ruddy Valentino^1^, Remi Neviere^1^, Dabor Resiere^1^

#### ^1^Chu de Martinique, Fort-de-France, Martinique, France; ^2^CHU de Guadeloupe, Pointe à Pitre, France; ^3^CH de Guyane, Cayenne, Guyane Francaise

##### **Correspondence:** Dabor Resiere (dabor.resiere@chu-martinique.fr)

*Annals of Intensive Care* 2023, **13(Suppl 1):**FC-0135

**Rationale:** Objectives: this study aimed to describe the patient selection process for long-haul air transfer during the fourth wave of Covid-19, linked to the spread of the delta variant, and to analyze the impact on ICU mortality.

**Patients and methods/materials and methods:** Patients admitted between 11/08/2021 and 19/10/2021 in permanent and transient ICUs were analyzed. Individual criteria previously published by scientific societies adapted to long-haul transfers were used to select “fit-to-fly” patients. An empirical evaluation by the physician in charge was also performed, categorizing patients as red, yellow, or green. A propensity score was used to evaluate the impact of the selection and transfer on ICU mortality.

**Results:** Two thousand six hundred seventy-two evaluations were made on 367 patients during 49 dedicated rounds. 89 ± 4% had at least one disqualifying factor, most of them due to a respiratory cause. Seven fits-to-fly patients were detected seven times, and 29 times when patients subjectively classified in the yellow category were included. 67 patients were evacuated using 11 air transfers, with 6 having 5–12 patients at a time. Proclive ventilation (OR 1.5, p < 0.01) was associated with increased mortality, whereas air transfer was protective (OR 0.48, p < 0.02).

**Conclusion:** In cases where ICU facilities are overwhelmed, long-haul air transfers can help increase the capacity of care without an increase in mortality. They require dedicated logistical organization in the ICU to ensure a good selection and preparation of the patients.

**Compliance with ethics regulations:** N/A.

## FC-136 Corticosteroids induces an early but limited decrease of the systemic pro-inflammatory profile in critically-ill SARS-CoV-2 patients

### Tomas Urbina^1^, Paul Gabarre^1^, Vincent Bonny^1^, Jean-Remi Lavillegrand^1^, Marc Garnier^1^, Jeremie Joffre^1^, Nathalie Mario^1^, Guillaume Dumas^3^, Geoffroy Hariri^1^, Alexandre Elabaddi^2^, Lucie Darrivère^1^, Muriel Fartoukh^2^, Bertrand Guidet^1^, Eric Maury^1^, Yannick Chantran^1^, Pierre-Yves Boelle^1^, Guillaume Voiriot^2^, Hafid Ait-Oufella^1^

#### ^1^Hôpital Saint-Antoine, Assistance Publique - Hôpitaux de Paris, Paris, France; ^2^Hôpital Tenon, Assistance Publique - Hôpitaux de Paris, Paris, France; ^3^CHU Grenoble, Grenoble, France

##### **Correspondence:** Tomas Urbina (tomas.urbina@aphp.fr)

*Annals of Intensive Care* 2023, **13(Suppl 1):**FC-0136

**Rationale:** Dysregulated host immune response was associated with outcome in critically ill COVID-19 patients during the first epidemic wave in early 2020. Corticosteroids have since become the standard of care for such patients, but their effect on the systemic immune-inflammatory response has been poorly investigated.

**Patients and methods/materials and methods:** In this multicenter prospective cohort, we included 150 COVID-19 patients between March and November 2020, 47 treated with corticosteroids, 103 without. C-reactive protein (CRP), lymphocyte count and fibrinogen levels upon hospital admission, before initiation of steroid treatment, were collected. These biomarkers were further collected upon ICU admission, 3 days and 7 days later, along with Interleukin (IL)-6, IL-10 and Tumor Necrosis Factor-alpha (TNF-a) plasma levels.

**Results:** Median age was 62 [53–70] years old, and 96 (65%) patients were mechanically ventilated. At ICU admission, 3 [2–6] days after steroid initiation for treated patients, CRP (204 [90–273] vs 107 [69–182] mg/L, (p = 0.02)) and fibrinogen (7.6 [6.9–8.7] vs 6.7 [5.4–7.7] g/L (p < 0.01)) were lower in the steroid treated group, with more profound lymphopenia (810 [590–1250] vs 645 [478–893]/mm^3^ (p = 0.01)). IL-6 was lower (85 [33–267] mg/L vs 43 [14–96] pg/mL, p < 0.01), with surprisingly higher TNF-a (17[0–30] vs 30 [0–50] pg/mL, p = 0.02) and lower IL-10 (21 [13–36] vs 11 [0–22] pg/mL, p < 0.01). These differences remained significant over time during ICU stay for CRP, fibrinogen, lymphocyte count and TNF-a, with a non-significant trend for Il-6 and IL-10. Sensitivity analysis with propensity score matching including pre-treatment hospital admission CRP levels rendered similar results. Biomarker levels were thus different at ICU admission between groups, but kinetics were similar during ICU stay, suggesting that corticosteroid treatment induced an early and sustained but partial decrease of systemic inflammation. Among corticosteroid-treated patients, CRP (p = 0.012), fibrinogen (p = 0.041) and lymphocyte count (p = 0.004) over time were associated with outcome, conversely to plasma cytokine levels.

**Conclusion:** In this multicentre prospective cohort of critically-ill SARS-CoV-2 patients with propensity score matching analysis, steroid treatment was associated with an early, sustained but partial decrease of the systemic pro-inflammatory signature. CRP and lymphocyte count remained associated with outcome in corticosteroid treated patients, conversely to plasma cytokine levels. Further research on the use of these biomarker’s kinetics to individualize immunomodulatory treatments is warranted.

**Compliance with ethics regulations:** Yes in clinical research.

**Figure 1 (abstract FC-136):**
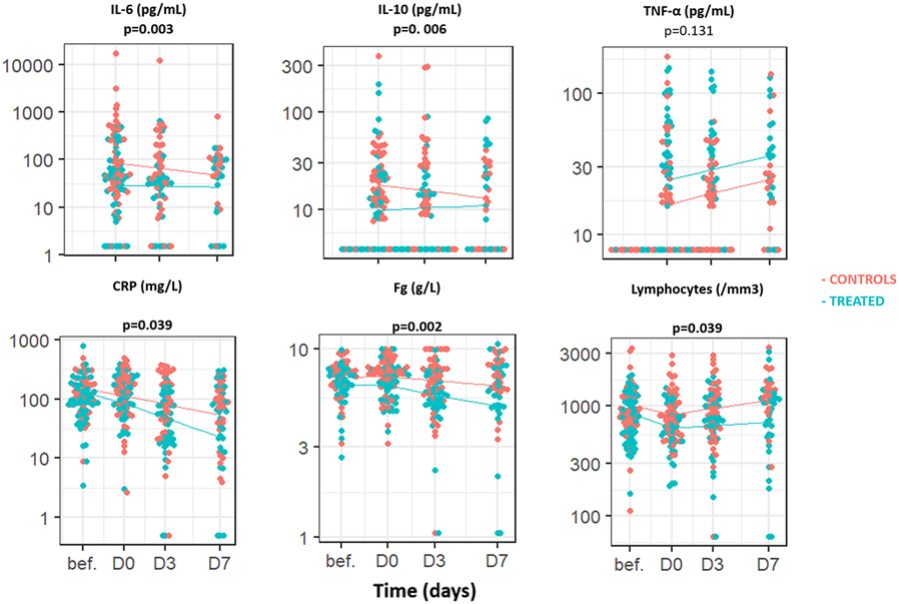
Time course of IL-6, CRP, fibrinogen, TNF-alpha, IL-10 plasma levels and blood lymphocyte count over time in the matched population (n = 92). (bef) pret-treatment values on hospital admission, Day 0 was ICU admission, p-value from a mixed model

## FC-137 Efficacy and safety of tocilizumab in COVID-19 related ARDS: the Tunisian intensive care society multi-centre study

### Amira Jamoussi^1^, Sirine Rhaiem^1^, Mohamed Khalil Ghozzi^1^, Emna Rachdi^1^, Fatma Jarraya^1^, Nacef Ben Mrad^1^, Imed Aissa^2^, Kais Ben Romdhane^3^, Youssef Blel^3^, Asma Mahdi^4^, Sami Abdellatif^4^, Oussama Jawed^5^, Souheil El Atrous^5^, Emna Ennouri^6^, Mohamed Boussarssar^6^, Hana Ben Ali^7^, Hatem Ghadhoun^7^, Houda Mateur^8^, Fatma Essefi^9^, Takoua Merhebene^9^, Amira Ben Jazia^10^, Nozha Brahmi^10^, Samia Ayed^1^, Jalila Ben Khelil^1^

#### ^1^Hôpital Abderrahmen mami, Tunis, Tunisie; ^2^Clinique Hannibal, Tunis, Tunisie; ^3^Clinique Carthagène, Tunis, Tunisie; ^4^Hôpital la Rabta, Tunis, Tunisie; ^5^Hôpital Tahar Sfar, Mahdia, Tunisie; ^6^Hôpital Farhat hached, Sousse, Tunisie; ^7^Hôpital Habib Bougatfa, Bizerte, Tunisie; ^8^Hôpital régional Hédi Jaballah, Tozeur, Tunisie; ^9^Hôpital régional de Zaghouan, Zaghouan, Tunisie; ^10^CAMU, Tunis, Tunisie

##### **Correspondence:** Amira Jamoussi (amira.jamoussi@fmt.utm.tn)

*Annals of Intensive Care* 2023, **13(Suppl 1):**FC-0137

**Rationale:** Interleukin-6 (IL-6), one of the main pivots in mediating inflammation in COVID-19, is associated with disease’s severity and mortality. Treatment with IL-6 receptor blockers has been recommended by the World Health Organization for patients with severe or critical COVID-19 infection. Objective: We aimed to explore factors associated with mortality among critically ill COVID-19 patients who received tocilizumab; and to detect safety issues and possible complications attributable to tocilizumab administration.

**Patients and methods/materials and methods:** It was a retrospective multi-centre study conducted in 10 Tunisian ICUs between November 2020 and March 2022. Critically ill patients diagnosed with COVID-19 ARDS who received tociluzimab were included. Anamnestic, clinical and outcome data were collected. Factors associated with survival were investigated and attributable tocilizumab complications were collected.

**Results:** We included 176 patients with mean age of 56.8 ± 14 years, gender ratio of 1.55. Most patients had comorbidities, including obesity (n = 84, 47.4%), hypertension (n = 60, 34.1%), and diabetes (n = 49, 27.8%). All of them met ARDS criteria divided into severe (n = 75; 42.6%), moderate (n = 83; 47.2%) and mild (n = 18; 10.2%). Dexamethasone was administrated in 169 patients (96%) and high dose of methylprednisolone in 28 (15.9%). Median tocilizumab dose was 800 mg IQR [600–800]. On average, tocilizumab was administrated at day 4 from ICU stay, at day 8 from respiratory distress and at day 14 from symptoms beginning. A second tocilizumab dose was administered in 33 patients (18.8%) and a third one in one patient. Median length of stay was 14 days IQR [9–20] and overall mortality was 57.4%. Independent predictive mortality factors are listed in Table 1. Complications related with tocilizumab administration were: mild transaminases elevation (n = 9, 5.1%), thrombocytopenia (n = 13, 7.4%) and nosocomial infections (n = 72, 40.9%).

**Conclusion:** In critically ill COVID-19 patients who received tocilizumab, mortality was associated with age > 54 years, white blood cells count > 8990/mm^3^, severe ARDS and invasive mechanical ventilation. Apart from multifactorial infection susceptibility, very few complications were noted.

**Compliance with ethics regulations:** Yes in clinical research.

**Table 1 (abstract FC-137):**
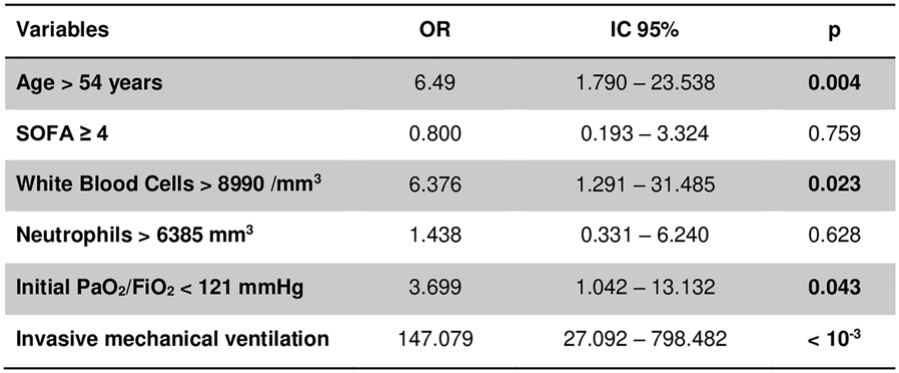
Mortality multivariate analysis among COVID-19 patients receiving tocilizumab

## FC-138 SARS-COV2 infection in patients hospitalized with diabetes and high blood pressure

### Abdallah Mechebbek^1^, Sabrina Benissad^1^, Meriem Boumaza^1^, Yamna Raho^1^, Badreddine Errouane^1^, Belarbi Khemliche^1^

#### ^1^Établissement hospitalier et universitaire (EHU), Oran, Algerie

##### **Correspondence:** Abdallah Mechebbek (mecbec4@yahoo.fr)

*Annals of Intensive Care* 2023, **13(Suppl 1):**FC-0138

**Rationale:** Diabetes and high blood pressure are antecedents found in patients with COVID-19 whose evolution was marked by worsening or even death [1]. Our objective is to estimate the incidence of diabetes associated with high blood pressure, to calculate the number of intensive care admissions and the number of deaths in these patients. Are diabetes and high blood pressure risk factors of severity in the event of SARS-COV2 infection?

**Patients and methods/materials and methods:** This was a retrospective and descriptive study, concerning all patients hospitalized for COVID-19 infection between November 2020 and October 2021. The number of patients included was 1439. We separated the patients into two groups (G1 and G2) according to the presence or absence of a history of diabetes and high blood pressure: G1 with diabetes and high blood pressure (n = 759) and G2 without diabetes and high blood pressure (n = 680).

**Results:** Fifty-two point 7% of patients were diabetic and treated for high blood pressure. The mean age was significantly higher in G1 than in G2 (68.09 ± 12.79 years vs. 59.07 ± 15.58 years; p < 0.0001). There was a male predominance in both groups (54.4% vs 62.1%). The number of patients admitted to intensive care unit was significantly higher in G1 than in G2 (33.7% vs 21.0%; p < 0.0001; odd ratio: 1.91 (1.50–2.42) 95% CI). The number of deaths was significantly higher in G1 than in G2 [46.1% vs (31.8%; p < 0.000w; odd ratio: 0.54 (0.43–0.67) 95% CI].

**Conclusion:** In our study, diabetes and high blood pressure were associated with an increased risk of intensive care admission and death in patients hospitalized for COVID-19. The patients with diabetes and high blood pressure are older than others.

**Reference 1**

Grasselli G, Zangrillo A, Zanella A, Antonelli M, Cabrini L, Castelli A, et al. Baseline characteristics and outcomes of 1591 patients infected with SARS-CoV-2 admitted to ICUs of the Lombardy Region, Italy. JAMA 2020;323 (16):1574.

**Compliance with ethics regulations:** Yes in clinical research.

## FC-139 Analysis of dexamethasone dosages used in patients hospitalized with COVID-19

### Abdallah Mechebbek^1^, Meriem Boumaza^1^, Yamna Raho^1^, Sabrina Benissad^1^, Badreddine Errouane^1^, Belarbi Khemliche^1^

#### ^1^Établissement hospitalier et universitaire (EHU), Oran, Algerie

##### **Correspondence:** Abdallah Mechebbek (mecbec4@yahoo.fr)

*Annals of Intensive Care* 2023, **13(Suppl 1):**FC-0139

**Rationale:** Corticosteroids have been used in the COVID-19 therapeutic protocol [1]. Our objective was to evaluate the dosages of dexamethasone used in our patients hospitalized with hypoxic COVID. Should we increase the doses of dexamethasone for a better effect? We analyzed the SpO_2_ at 10 days, the number of intensive care unit (ICU) admissions, the number of deaths and the duration of stay.

**Patients and methods/materials and methods:** This was a retrospective, descriptive study of hypoxemic patients who received dexamethasone for hypoxic COVID-19. The number of patients included was 829. Dexamethasone was used intravenously. We separated the patients into two groups with respect to the median, (G1): patients who received a dose ≤ 8 mg/day (n = 539) of dexamethasone and (G2): patients receiving a dose > 8 mg/day (n = 290).

**Results:** The mean age was comparable between the two groups (63.96 ± 14.48 years vs 64.17 ± 13.40 years; p = 0.83). There was a male predominance in both groups (58.8% vs. 59.3%; p = 0.88). The SpO_2_ at 10 days was 91.86 ± 7.81% in G1 vs 90.95 ± 7.59% in G2; p = 0.10. The number of patients admitted to ICU was significantly higher in G2 vs G1 (37.2% vs 30.2%, p = 0.04; odd ratio: 1.36 (1.01–1.84) 95% CI). The number of deaths was significantly higher in G2 vs G1 (40.3% vs 29.3%, p = 0.001; odd ratio: 0.61 (0.45–0.82) 95% CI). The mean duration of stay was significantly prolonged in G2 vs G1 (17.39 ± 14.65 days vs 13.14 ± 9.51 days, p < 0.0001).

**Conclusion:** dexamethasone dosages > 8 mg/day have been associated with a risk of ICU admission and death. There is no improvement in SpO_2_ after 10 days of treatment with dexamethasone > 8 mg/day. According to our study, it is probably not necessary to increase dexamethasone doses beyond 8 mg/day in patients hospitalized with COVID-19.

**Reference 1**

Granholm A, Munch MW, Myatra SN, (2022) Dexamethasone 12 mg versus 6 mg for patients with COVID-19 and severe hypoxaemia: a pre-planned, secondary Bayesian analysis of the COVID STEROID 2 trial. Intensive Care Med 48:45–55.

**Reference 2**

2. Ebrahimi Chaharom F, Pourafkari L, Ebrahimi Chaharom AA, Nader ND. Effects of corticosteroids on Covid-19 patients: a systematic review and meta-analysis on clinical outcomes. Pulmonary pharmacology & therapeutics. 2022;72:102107. Epub 2021/12/18.

**Compliance with ethics regulations:** Yes in clinical research.

## FC-140 Challenges of a pandemic: experience of a dedicated hospital

### Meriem Boumaza^1^, Abdallah Mechebbek^1^, Yamna Raho^1^, Sabrina Benissad^1^

#### ^1^EHU, Oran, Algerie

##### **Correspondence:** Meriem Boumaza (meriem.boumaza@hotmail.com)

*Annals of Intensive Care* 2023, **13(Suppl 1):**FC-0140

**Rationale:** The pandemic of COVID 19 infection has forced health care systems to make significant and ongoing adjustments, both logistically and medically. The challenges of managing patients with COVID 19 were coping with a poorly understood disease, a sudden influx of patients, and maintaining the quality and consistency of care for patients with other conditions. We share here the experience of our hospital, a structure attached to a university hospital and entirely dedicated to the management of COVID 19.

**Patients and methods/materials and methods:** The university hospital transferred the COVID 19 activity, which had been initiated within the structure's services, to the new hospital, whose construction was being completed. The teams assigned to this activity are responsible for providing triage and diagnostic consultation, admitting, and treating patients requiring emergency hospital care, and receiving transfers of COVID patients screened at the university hospital. Multiple statistics are recorded to measure the efficiency of the facility.

**Results:** Management and absorption of the different waves of COVID: from July 2020 to February 2022, 4753 patients were admitted, 27,213 consultations were provided. Evacuation of COVID patients from the EHU to our hospital, allowing an almost normal activity of the medical-surgical services throughout the pandemic. Organization and constitution of a work team, training of general practitioners in intensive care techniques. Acquisition of experience of medical and paramedical staff in the management of critical care patients during this pandemic.

**Conclusion:** Our experience suggests the efficiency of outsourcing the management of COVID patients to dedicate structures to curb the various disturbances inherent in the management of potentially contaminating patients, influxes in large numbers and often requiring advanced care. Consequently, other hospitals could continue to provide the usual care to non-COVID patients.

**Compliance with ethics regulations:** Yes in clinical research.

## FC-141 Impact of COVID-19 pandemic waves on health-care worker hand hygiene activity in department of medicine and ICU as measured by an automated monitoring system

### Amine Si Ali^1^, Frédérique Schortgen^1^, Valérie Garrait^1^, Stéphanie Poullain^1^, Camille Jung^1^

#### ^1^CH Intercommunal de Créteil, Créteil, France

##### **Correspondence:** Amine Si Ali (amine.siali@chicreteil.fr)

*Annals of Intensive Care* 2023, **13(Suppl 1):**FC-0141

**Rationale:** Hand hygiene (HH) compliance among health-care workers is important for preventing transmission of infectious diseases. Aim: To describe health-care worker hand hygiene activity in ICU and non-ICU patients’ rooms, using an automated monitoring system (AMS), before and after the onset of the COVID-19 pandemic.

**Patients and methods/materials and methods:** In December 2019, the Hospital installed an AMS to monitor ABHS consumption and measure the effectiveness of efforts to promote HH. The present study reports consumption between 1st February 2020 and 30th November 2020. This duration may be divided into four consecutive periods: P1, from 1st February to 10th March 2020, is the baseline period. P2, from 11th March to 24th May 2020, included the lockdown, and most patients then hospitalized were positive for SARS-CoV-2. During P2, nursing and medical staff received intensive HH training, and the media stressed the importance of HH on a daily basis. P2 may be considered an intervention period. P3, from 25th May to 7th August 2020, was the interepidemic period, characterized by few COVID-19 cases. Finally, P4, from 8th August to 30th November 2020, encompassed the second wave of the pandemic, and was marked by a rise in COVID-19 cases. In December 2019, AMS wireless units were installed inside ABHS dispensers placed in all ICU and DM rooms. There were 16 ICU and 54 DM devices in all: 2 per single room and 3 per double room. No AMS unit was installed outside of patient rooms.

**Results:** Over the 10 months of the study, in the DM, ABHS consumption was higher in COVID-19 patients’ rooms during P2 (16.1 ± 17, versus 9.3 ± 10.7 in other rooms; P = 0.01) and P4 (12.7 ± 11.3, versus 7.5 ± 2.4 in other rooms; P < 0.001). A similar trend was observed in the ICU during the first wave (46 ± 18.8 in COVID-19 rooms, versus 22.7 ± 24.9 in other rooms; P < 0.001), but ABHS consumption did not differ by COVID-19 status during the second wave (46.9 ± 16.3 in COVID-19 rooms, versus 47.7 ± 12.6 in other rooms; P = 0.59). Factors found to be associated with ABHS consumption were period, in the DM, and number of HCWs present, in the ICU.

**Conclusion:** The pandemic greatly increased ABHS consumption in COVID-19 patients’ rooms. AMS is a valuable tool for the real-time evaluation of ABHS consumption. Real-time monitoring system can be used to adapt hand hygiene promotion measures.

**Compliance with ethics regulations:** Yes in clinical research.

**Figure 1 (abstract FC-141):**
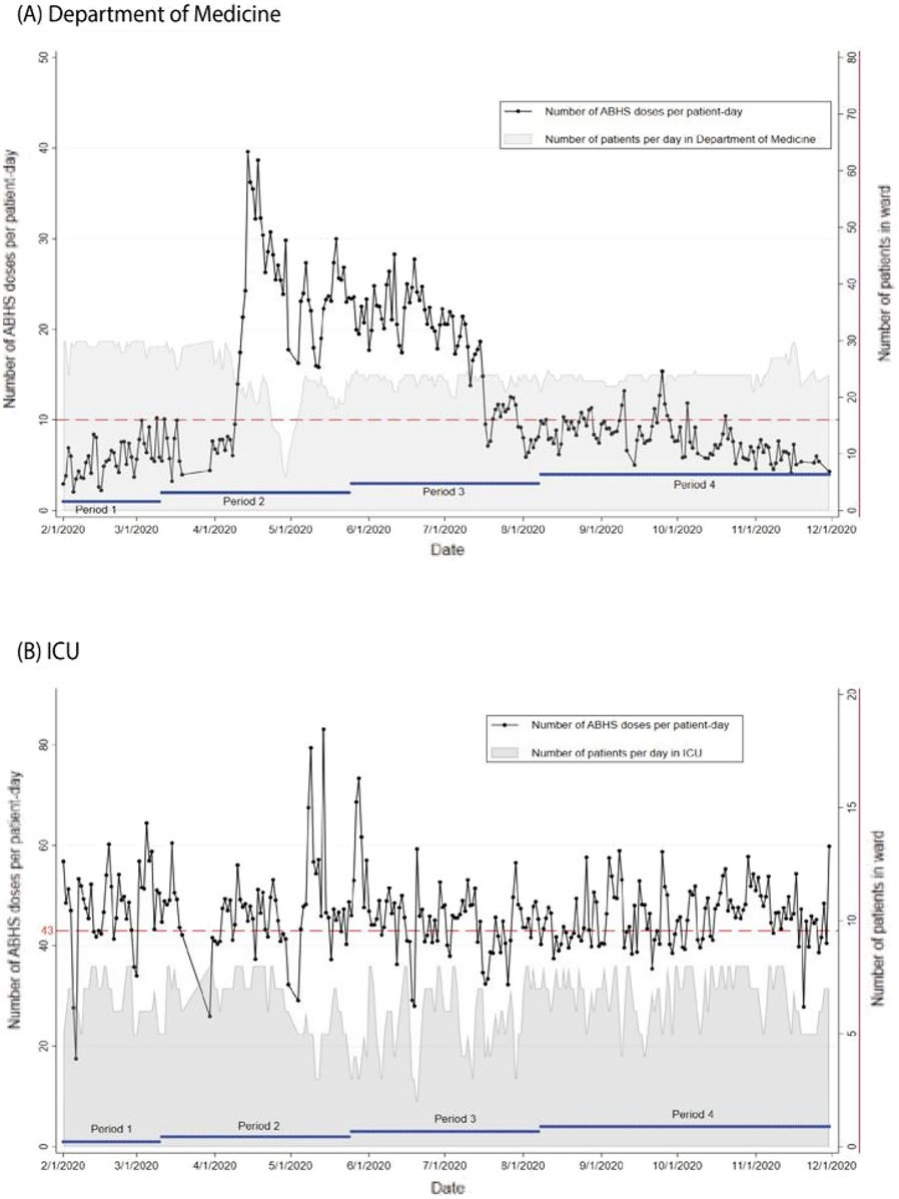
Number of 3-ml doses of ABHS per patient-day- and numbers of hospitalized patients in (A) DM and (B) ICU over course of study

## FC-142 Biomarkers of inflammation in critically ill COVID-19 patients: Prognostic value at ICU admission

### Amira Jamoussi^1^, Lynda Messaoud^1^, Emna Rachdi^1^, Fatma Jarraya^1^, Nacef Ben Mrad^1^, Sadok Yaalaoui^1^, Samia Ayed^1^, Jalila Ben Khelil^1^

#### ^1^Hôpital Abderrahmen Mami, Tunis, Tunisie

##### **Correspondence:** Amira Jamoussi (amira.jamoussi@fmt.utm.tn)

*Annals of Intensive Care* 2023, **13(Suppl 1):**FC-0142

**Rationale:** Coronavirus disease 2019 (COVID-19) causes an inflammatory cytokine storm whose degree is linked to COVID-19 severity. Elevated levels of interleukin-6 (IL-6), C-reactive protein (CRP), procalcitonin (PCT) and D-dimer have been frequently reported in severe forms. In critically ill COVID-19 patients, the ability of these biomarkers to predict poor outcome is still debated. We aimed to determine the ability of inflammatory biomarkers (Il-6, PCT, CRP, and leukocytes) to predict mortality in COVID-19 patients in the ICU.

**Patients and methods/materials and methods:** A prospective study was conducted in the respiratory medical intensive care unit of Abderrahmen Mami teaching hospital between August 2020 and February 2022. Critically ill patients diagnosed with COVID-19 and having benefited from a complete investigation of the biomarkers of inflammation at ICU admission were included. Demographic, clinical, biological, and therapeutic data were collected, and independent predictive factors for mortality were determined.

**Results:** During the study period, 133 patients were included, and the mortality rate was 51.1%. Biomarkers of inflammation significantly associated with mortality were: IL-6 level > 47.6 pg/mL (p < 10^–3^), CRP > 76 mg/L (p = 0.006), procalcitonin > 2.36 µg/L (p < 10^–3^), D-dimer ≥ 896 ng/mL (p < 10^–3^), fibrinogen ≥ 5.89 g/L (p = 0.001), and white blood cell count ≥ 12,500 cells/mm^3^ (p = 0.006). Other biological data associated with mortality included: PaO_2_/FiO_2_ ratio ≤ 104 mmHg (p < 10^–3^) and LDH level ≥ 439 IU/L (p = 0.013). Independent predictors of mortality are summarized in Table 1.

**Conclusion:** Among the biological markers of inflammation measured at ICU admission, IL-6 was the only independent predictor of mortality identified in COVID-19 patients.

**Compliance with ethics regulations:** Yes in clinical research.

**Table 1 (abstract FC-142):**
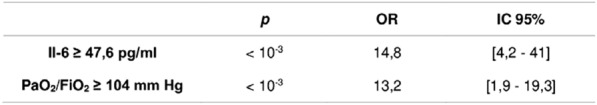
Independent predictive factors of ICU mortality

## FC-143 Severe COVID-19 infection: predictive factors for mortality observed in Algerian patients

### Sabrina Benissad^1^, Abdallah Mechebbeck^1^, Arslan Bettayeb^1^, Djahida Terfani^1^

#### ^1^Etablissement hospitalo universitaire d'Oran 1er Novembre, Oran, Algerie

##### **Correspondence:** Sabrina Benissad (sabrina.benissad@gmail.com)

*Annals of Intensive Care* 2023, **13(Suppl 1):**FC-0143

**Rationale:** Variations in the characteristics and risk factors of patients with covid 19 may occur from one region to another. There are limited data available on the Algerian population. In this study, we aim to examine the factors that determine survival among hospitalized COVID-19 patients across the different outbreaks of COVID-19 disease in the Oran region of Algeria.

**Patients and methods/materials and methods:** This is a monocentric, retrospective study concerning patients admitted to the 240-bed Hai Nedjma hospital. The number of patients included was 1644, from November 1, 2020 to March 31, 2022. Inclusion criteria were: (1) Adult patients admitted for severe COVID-19 infection, (2) Patients from whom data were collected. Exclusion criteria: Surgical patients.

**Results:** Of the 1644 patients, 603 (39.4%) have died. Survivors were younger (60.1 ± 1.1 vs 67.1 ± 1.4 years) and had fewer comorbidities (33% mortality in the patients without comorbidities VS 43% mortality in the patients with comorbidities. The most common pathologies found in our patients were high blood pressure (39.7%), diabetes (34.9%), heart disease (10.2%), respiratory disease (7.9%) and cancer (4.2%). In the group of patients over 65 years old, there was no significant difference in mortality whether the patients had a comorbidity or not (48.3 vs 48.2). Morbidity was significantly higher among the patients under the age of 65 years with chronic disease compared to those who had none (35.4% vs 24.5).

**Conclusion:** The results of our study indicate that patients with severe COVID-19 had a hospital mortality rate similar to that observed in some regions of the world. Although cardiovascular pathology is an important risk factor for mortality, advanced age appears to be the main risk factor for death in severe COVID-19 infection.

**Compliance with ethics regulations:** Yes in clinical research.

## FC-144 Leukocytosis as predictor of mortality in COVID-19 critically ill patients

### Dhouha Ben Braiek^1^, Hend Zorgati^1^, Yosri Ben Ali^1^, Sourour Bel Haj Youssef^1^, Nadine Boukadida^1^, Rihab Boubtane^1^, Rahma Ben Jazia^2^, Amani Kacem^2^, Jihene Ayachi^1^

#### ^1^Medical Intensive Care Unit, Ibn El Jazzar University Hospital, Kairouan, Tunisie; ^2^Pulmonology Department, Ibn El Jazzar University Hospital, Kairouan, Tunisie

##### **Correspondence:** Jihene Ayachi (ayachijihen@gmail.com)

*Annals of Intensive Care* 2023, **13(Suppl 1):**FC-0144

**Rationale:** Intense inflammatory response triggered by COVID-19 plays an essential role in the severity and prognosis of the disease. Thus, biomarkers that indicate the state of inflammation can be used to predict the severity of the disease. Our aim was to investigate the prognostic value of inflammatory markers such as C-reactive protein (CRP), leukocytes and procalcitonin (PCT) in critically ill COVID_19 patients.

**Patients and methods/materials and methods:** Retrospective study conducted in a 9-bed medical intensive care unit (ICU) of a university hospital between September 2020 and December 2021 in patients with confirmed SARS-CoV-2 infection. Demographic characteristics, admission severity, clinical course, management and outcomes were collected. Statistical analysis was performed to identify inflammatory markers predictive of mortality in critically ill patients with COVID-19. Receiver operating characteristic (ROC) curve was used to determine cut-off and area under the curve (AUC).

**Results:** During the study period, 172 patients were admitted for COVID-19 pneumonia. The mean age was 51.5 ± 14.4 years with male predominance 104 (60.5%). Obesity (n = 60, 34.9%), diabetes (n = 48, 28.5%) and hypertension (n = 30, 17.4%) were the most common underlying comorbidities. Initial symptoms were dyspnea (n = 138, 80.1%), asthenia (n = 136, 79.1%), fever (n = 118, 68.6%) and non-productive cough (n = 98, 57%). Median leucocytes and CRP levels were 9650 [6625–12260] cells/mm^3^ and 90 [66.5–155] mg/L, respectively. Mean PCT was 0.168 ± 0.08 ng/mL. Invasive mechanical ventilation (IMV) was performed in 69 (40.1%) cases. Empiric antibiotics were prescribed in 168 (98.3%) patients. During ICU stay, 41 (23.8%) patients developed septic shock. The most frequent adverse events were shock (n = 68, 39.5%), nosocomial infection (n = 58, 33.7%) and acute renal failure (n = 51, 29.7%). Median ICU length of stay was 9 [6–14] days and 70 patients died (40.7%). Univariate analysis showed that, among inflammatory biomarkers, leukocytosis was significantly associated with mortality (11,300 [8540–14600] vs 8500 [5800–11000] cells/mm^3^; p = 0.000), whereas CRP (p = 0.460) and PCT (p = 0.577) were similar between survivors and non-survivors. The optimal cut-off value for predicting ICU mortality by ROC analysis was 9650 cells/mm^3^ for leukocytes. The AUC for ICU mortality was 0.702 (95% CI, 0.62–0.78), which was above 0.7, indicating that it was a good predictor.

**Conclusion:** The present study demonstrated that among inflammatory biomarkers, leukocytosis at admission is a significant predictor of mortality in COVID-19 critically ill patients. Leukocyte count is a good predictive marker of mortality.

**Compliance with ethics regulations:** Yes in clinical research.

## FC-145 Hyperglycemia and COVID-19: impact on severe hospitalized patients

### Yamna Raho^1^, Abdallah Mechebbek^1^, Meriem Boumaza^1^, Sabrina Benissad^1^

#### ^1^Etablissement Hospitalier Universitaire d’Oran, Oran, Algerie

##### **Correspondence:** Yamna Raho (yamna.raho@gmail.com)

*Annals of Intensive Care* 2023, **13(Suppl 1):**FC-0145

**Rationale:** Hyperglycemia in patients hospitalized for COVID-19 has been found in non-diabetic patients [1]. Our goal was to estimate the incidence of hyperglycemia and to analyze the number of intensive care unit (ICU) admissions, the number of deaths and the length of stay.

**Patients and methods/materials and methods:** This is a retrospective, descriptive study of all non-diabetic patients admitted for COVID-19. A total of 512 patients were included. Patients were divided into two groups, normoglycemia (n = 295) vs hyperglycemia (n = 277).

**Results:** Hyperglycemia was found in 48.33% (277/512) of patients. The mean age was comparable in the two groups 62.14 ± 14.78 years vs 60.90 ± 16.71 years (p = 0.34). There were more patients admitted to ICU in the hyperglycemia vs normoglycemia group [115/277, 41.51%] vs [91/295, 30.84%]; the difference was statistically significant (OR: 1.59; p = 0.008). The number of deaths was higher in the hyperglycemia vs. normoglycemia group [107/277, 38.62%] vs. [89/29, 30.17%]; the difference was significant (OR: 0.68; p = 0.03). The mean length of stay was longer in the hyperglycemia vs. normoglycemia group (12.49 ± 9.18 days vs 11.50 ± 9.22 days); the difference was non-significant (p = 0.19).

**Conclusion:** Hyperglycemia has been associated with an increased risk of ICU admission and death in non-diabetic patients hospitalized for COVID-19.

**Reference 1**

1. Umpierrez GE, Isaacs SD, Bazargan N, You X, Thaler LM, Kitabchi AE. Hyperglycemia: an independent marker of in-hospital mortality in patients with undiagnosed diabetes. The Journal of Clinical Endocrinology & Metabolism. 2002; 87(3):978–82.

**Compliance with ethics regulations:** Yes in clinical research.

## FC-146 Annexin-V positive membrane-derived microparticles levels assessment in severe COVID-19

### Benjamin Swinyard^1^, Valentine Jacob^1^, Alexis Lambour^1^, Yoann Zebib^1^, Momar Diouf^1^, Simon Soudet^1^, Etienne Brochot^1^, Isabelle Six^1^, Marie-Antoinette Sevestre^1^, Mailys Le Guyader^1^, Sandrine Soriot-Thomas^1^, Julien Maizel^1^, Etienne Brochot^1^, Michel Slama^1^

#### ^1^CHU Amiens-Picardie, Amiens, France

##### **Correspondence:** Benjamin Swinyard (benjamin.swinyard@hotmail.fr)

*Annals of Intensive Care* 2023, **13(Suppl 1):**FC-0146

**Rationale:** To evaluate microparticle levels in a cohort of SARS-CoV-2 patients hospitalized in an intensive care unit with and without COVID-19 associated thromboembolic events.

**Patients and methods/materials and methods:** Annexin-V positive microparticle levels prospectively assessed by flow cytometry in connection with a SARS-CoV-2 infection. Patients were adult ICU admissions with a SARS-CoV-2 infection from February 2020 through October 2021 at the ICU of the Amiens University Hospital.

**Results:** We prospectively investigated 123 patients who met the inclusion criteria for analysis. Thirty-four patients (27.6%) had a thromboembolic event, fifty-three (43%) died. Total endothelial and platelets membrane-derived particles were drastically increased in SARS-CoV-2 patients with severe disease compared to moderate disease and healthy volunteers. Moreover, a slightly higher small/large ratio for platelet membrane-derived particles in patients was associated with thrombo-embolic events.

**Conclusion:** Endothelial and platelets membrane-derived particles levels were increased in severe COVID-19 and their sizes could be considered as biomarkers of COVID-19-associated thrombo-embolic events.

**Compliance with ethics regulations:** Yes in clinical research.

## FC-147 Mortality of COVID-19 patients in the intensive care unit: comparison of two prognostic scores

### Imen Talik^1,2^, Asma Azaza^1,2^, Khaoula Ben Ismail^1,2^, Fatma Essafi^1,2^, Hamdi Chaabouni^1,2^, Najla Ben Slimene^1,2^, Takoua Merhabene^1,2^

#### ^1^Service de réanimation médical, hôpital régional Zaghouan, Tunis, Tunis, Tunisie; ^2^Faculté de médecine Tunis, Université Tunis El Manar, Tunis, Tunisie

##### **Correspondence:** Asma Azaza (asmaazaza8@gmail.com)

*Annals of Intensive Care* 2023, **13(Suppl 1):**FC-0147

**Rationale:** Since its outbreak, COVID-19, has been life-threatening for many patients. Early identification of the most severe patients enhances the quality of care. As such, several scores have been proposed. The aim of this study was to evaluate the performance of the “Confusion, Urea nitrogen, Respiratory rate, Blood pressure and age of 65 years or older” (CURB-65) score and the “National Early Warning Score 2” (NEWS 2) to predict COVID-19 mortality in the intensive care setting.

**Patients and methods/materials and methods:** This was an observational retrospective study conducted in a medical intensive care unit (ICU), from September 5, 2020, to September 29, 2021. All consecutive patients admitted to the ICU for acute respiratory failure with confirmed SARS-CoV-2 infection were included. Demographic, clinical and para-clinical features, therapeutic modalities and outcomes were collected. Yield was evaluated by calculating the area under the curve (AUC). In high-risk groups sensitivity, specificity, positive predictive value (PPV) and negative predictive value (NPV) were calculated.

**Results:** Three hundred and twelve patients were included during the study period. The mean age was 55 ± 13 years with a male predominance (58.3%). The three most frequent comorbidities were obesity (41.3%), diabetes (36.8%) and hypertension (36.2%). The mortality rate in the ICU was 41.6% (n = 130). Deceased patients had higher mean scores for CURB-65 (1.5 ± 0.9 vs 1 ± 0.9, p < 10^−3^) and NEWS 2 (7.5 ± 2 vs 8.7 ± 2, p < 10^−3^). The AUC for CURB-65 and NEWS 2 were 0.633 and 0.641, respectively. With a cutoff of 3, the CURB-65 had a specificity of 98.3% and a PPV of 84.2% for predicting mortality in COVID-19 patients in intensive care. The NEWS ≥ 7 had a sensitivity of 90% and an NPV of 78%.

**Conclusion:** CURB-65 and NEWS 2 are not effective enough to predict COVID-19 mortality in the ICU. However, with a cutoff of 7, NEWS 2 can be used to screen patients with severe COVID-19 who are at a high risk of ICU.

**Compliance with ethics regulations:** Yes in clinical research.

## FC-148 C-reactive protein as a prognostic indicator in critically ill patients with COVID-19

### Ilef Alila^1^, Sana Kharrat^1^, Olfa Turki^1^, Kamilia Chtara^1^, Mabrouk Bahloul^1^, Mounir Bouaziz^1^

#### ^1^Hôpital habib bourguiba Sfax, Sfax, Tunisie

##### **Correspondence:** Ilef Alila (ilefalila1323@gmail.com)

*Annals of Intensive Care* 2023, **13(Suppl 1):**FC-0148

**Rationale:** The coronavirus disease 2019 (COVID-19) outbreak is an emerging global health threat. Nevertheless, predictors of clinical severity and complications in critically ill patients are still poorly described. Recent studies have reported that levels of inflammatory marker are elevated in patients with COVID-19 and may correlate with disease severity and progression. We aimed to investigate the association between C-Reactive Protein (CRP) and COVID-19 severity.

**Patients and methods/materials and methods:** We conducted a retrospective study of critically ill patients with confirmed SARS-CoV-2 infection in an intensive care unit (ICU) for 16 months. Outcomes were the need for ventilator support and in-hospital mortality. CRP levels were collected on admission.

**Results:** During the study period, 586 patients were included with a mean age of 59.5 ± 14.7 years and a sex ratio of 1.6. Mean SAPSII and SOFA scores were 29 ± 14.8 and 4 ± 2.6, respectively. Most patients had comorbidities, including hypertension (36%), obesity (32.1%), and diabetes mellitus (36.2%). A total of 419 (71.5%) patients had severe ARDS. Severe lung damage ranging between 50 and 75% of the lung parenchyma was estimated in 175 patients (30%). 117 (20%) had lung parenchymal damage ≥ 70%. Invasive mechanical ventilation was required in 264 patients (45.1%) for a median of 3 days. The median ICU length of stay was 6 [3–10] days and mortality was 49%. The mean CRP level was 163.8 ± 123 mg/L. CRP levels were significantly associated with mortality (p < 10^–3^) and a higher use of mechanical ventilation (p < 10^–3^).

**Conclusion:** C-reactive protein (CRP) levels have been identified as predictors of clinical severity and complications to provide a reference for clinical management. More sensitive indicators able to reflect lung lesion changes and disease severity need to be explored.

**Compliance with ethics regulations:** Yes in clinical research.

## FC-149 Etiologies of fever in severe traumatic brain injury

### Khaoula Ben Ismail^1,2^, Malek Kharrat^1,2^, Mariem Chaabani^1,2^, Fatma Essafi^1,2^, Najla Ben Slimene^1,2^, Imene Talik^1,2^, Moez Kaddour^1,2^, Takoua Merhabene^1,2^

#### ^1^Intensive Care Unit, Regional Hospital Zaghouan, Tunisia, Tunis, Tunisie; ^2^Faculty of Medicine of Tunis, University Tunis El Manar, Tunis, Tunisia, Tunis, Tunisie

##### **Correspondence:** Malek Kharrat (kharratmalek1@gmail.com)

*Annals of Intensive Care* 2023, **13(Suppl 1):**FC-0149

**Rationale:** Fever must be closely monitored in traumatic brain injury to prevent secondary brain damage due to systemic origin parameters. However, it is not always associated with a confirmed infection and could be a predictor of worse outcome. Aim: to describe origin and impact of fever in patients with traumatic brain injury admitted to intensive care unit (ICU).

**Patients and methods/materials and methods:** We conducted a retrospective study including traumatic brain injury patients admitted to the ICU, over a period of 4 years (from January 1st 2019 to December 31st 2022). Fever was daily monitored. Epidemiological, biological, radiological and evolving data were collected.

**Results:** Overall 57 patients were included, all of whom sustained a severe head injury following road accident. Gender ratio was 18. Mean age was 38.9 ± 16.7 years. Average APACHE II and SAPS II scores were 18 and 42. A thoracic trauma and/or abdominal trauma were associated respectively in 59.6% and 8% of cases. Need to orotracheal intubation and invasive mechanical ventilation were necessary in 68.4% of cases. Prophylactic antibiotics ware administrated on admission in 71.9% of patients. It was a beta-lactam antibiotics in all of cases. Confirmed bacteriological infections were noted in 61.4% of the patients. They were pneumonia in 60% of cases, urinary tract infections in 25% of cases and skin infection in 15% of cases. Non-fermenting pathogens were the most frequent microorganisms isolated in 25 cases among them P*seudomonas aerogenosa* was observed in 22 cases and A*cinetobacter baumanii* in 3 cases. Carbapenem resistant pathogens were isolated in 4 cases. The enterobacteriacae microorganisms were recorded in 6 cases with *Klebsiella pneumonia* isolated in 4 cases, P*roteus mirabilis* in 2 cases. These enterobacteriacae were bata-lactam resistant in 4 cases and carbapenem resistant in only one case. Fever higher than 38.2 °C was noted in 49.1% of the patients. It appeared meanly at the second day of ICU stay. Exhaustive survey didn’t find an infectious origin in 28.6% of cases. The mortality rate was 36.8%. Incidence of non-explained fever was significantly higher in the deceased group (50% versus 16% of the surviving group. P = 0.013). Moreover, 47.6% of the deceased patients received a probabilistic antibiotic versus 16.7% in survivors (p = 0.017).

**Conclusion:** Fever in patients with brain injury following traumatic brain injury is frequent but is not always associated with an infection leading to an over prescription of antibiotics. It seems however associated with poorer outcome in our study.

**Compliance with ethics regulations:** Yes in clinical research.

## FC-150 Prognostic significance of common neurobiological and neurophysiological markers at the acute phase of severe Covid-19

### Mathilde Piljan^1^, Adam Celier^1^, Geoffroy Vellieux^1^, Paul Henri Wicky^1^, Pierre Jaquet^1^, Hana Manceau^2^, Lila Bouadma^1^, Etienne De Montmollin^1^, Jean-François Timsit^1^, Katell Peoch^1^, Romain Sonneville^1^

#### ^1^Hôpital Bichat, Paris, France; ^2^Hôpital Beaujon, Clichy, France

##### **Correspondence:** Mathilde Piljan (mathilde.piljan@gmail.com)

*Annals of Intensive Care* 2023, **13(Suppl 1):**FC-0150

**Rationale:** SARS-Co-V2 presents a tropism for the central nervous system, as evidenced by a high prevalence of neurological symptoms in severe COVID-19 cases. In the absence of severe neurological symptoms, the prognostic significance of common neurobiological and neurophysiological markers measured at the acute phase of Covid-19 is unknown. We aimed to investigate serum concentrations of biomarkers of neuronal (Neuro-Specific Enolase, NSE) and glial (S100-beta Protein, S100B) injuries, EEG alterations observed at the acute phase of severe COVID-19, and their association with short term outcomes (i.e. invasive mechanical ventilation and mortality at 90 days).

**Patients and methods/materials and methods:** We conducted a prospective single-center cohort study at the Bichat Claude Bernard University Hospital Paris, France. Patients with COVID-19 infection (proven by nasopharyngeal reverse transcription–polymerase chain reaction) who required hospitalization for acute respiratory failure in the intensive care unit (ICU) between September 1, 2020 and March 31, 2021 were included. We excluded patients already intubated upon admission. NSE and S100B serum concentrations were measured on day 1, day 2, and day 3 after ICU admission. EEG was performed as early as possible after admission and analyzed prospectively according to a standardized grid by an experienced neurophysiologist blinded to patients’ outcomes. Main outcome measures included the need for invasive mechanical ventilation and mortality at 90 days.

**Results:** 134 patients (age: 63 [53–72] years) were included. At 90 days, 33 (24.6%) patients had been intubated, and 47 (35.1%) had died. In univariate analysis, serum concentrations of NSE on day 1, day 2, and day 3 were significantly associated with invasive mechanical ventilation and 90-day mortality. By contrast, S100B concentrations were not associated with intubation or mortality. Among EEG features, the absence of sleep Figures was associated with invasive mechanical ventilation. In contrast, the absence of sleep Figures and the presence of periodic slow complex were significantly associated with mortality at 90 days. In multivariable analysis, maximal NSE concentration [OR 1.15, 95% CI 1.06–1.26], presence of sleep Figures on EEG [OR 0.23, 95% CI 0.04–0.91] and SOFA score [OR 1.38, 95% CI 1.07–1.85] were independently associated with invasive mechanical ventilation. Maximal NSE concentration [OR 1.17, 95% CI 1.08–1.30], presence of sleep Figures on EEG [OR 0.07, 95% CI] and SAPS II [OR 1.12, 95% CI 1.05–1.24] were independently associated with mortality at 90 days.

**Conclusion:** NSE serum concentrations and simple EEG features measured at the acute phase of COVID-19 identify patients at high risk of short-term adverse outcomes, including invasive mechanical ventilation and mortality.

**Compliance with ethics regulations:** Yes in clinical research.

## FC-151 Neurological manifestations in patients with SARS-CoV-2 infection: a cases series about 1340 patients

### Salma Taouihar^1^, Amine Bouabdallaoui^1^

#### ^1^CHU Mohammed VI Oujda, Oujda, Maroc

##### **Correspondence:** Salma Taouihar (salma.taouihar@gmail.com)

*Annals of Intensive Care* 2023, **13(Suppl 1):**FC-0151

**Rationale:** The neurological manifestations associated with SARS CoV-2 are frequent and varied. The virus can induce, by different pathophysiological mechanisms, neurological manifestations and complications affecting the central nervous system (CNS) and peripheral nervous system (PNS). These occur at different stages of the infection.

**Patients and methods/materials and methods:** It is a Retrospective case series report including all patients, admitted with confirmed SARS-CoV-2 infection and neurologic manifestations at our university hospital. In this study, we describe the different neurological disorders, namely those of the central nervous system and the peripheral nervous system. 1340 hospitalized patients with laboratory confirmation of SARS-CoV-2 were included in the analysis.

**Results:** We included in this analysis 1340 patients with confirmed SARS-CoV-2 infection. A total of 835 patients were man (62.4%) while we had 37.6% of female patients, The mean age was 63.12 years. Our patient had as medical history, hypertension (32.3%) diabetes (32.5) %, heart disease (14.3%), and cerebrovascular disease (2.4%). The most frequent symptom was dry cough in 80.6% and dyspnea in 80.5% myalgia in 68.1%, Headache 49.8%, ageusia 32.2% and anosmia 9.3% Non-specific symptoms such as headache, dizziness, pain, myalgia, anosmia and ageusia are described in 9.3% to 68.1% of cases. More severe neurological damage affected 2.7% of SARS-CoV-2 hospitalized patients including various central or peripheral manifestations. the most frequent severe manifestations were cerebrovascular disease especially ischemic stroke reported in 19 patients (1.4%), followed by hemorrhagic stroke reported in 3 patients (0.2%), Guillain–Barré syndrome was described in 3 cases (0.2%) and facial palsy was described in 3 cases as a peripheral nervous system involvement (0.2%) and meningitis (0.1%).

**Discussion:** The CNS manifestations represent 27.7% of the patients while 28.5% present a PNS of SARS-CoV-2 manifestations. Several studies have explained how CoV-SRAS enters human cells via a cellular receptor that is the angiotensin converting enzyme 2 (ACE2) expressed on the surfaces of several human organs, including the nervous system. The pathophysiology explaining how the virus enters the central nervous system is still poorly understood.

**Conclusion:** Sars-CoV-2 can induce by different physiopathological mechanisms, neurological complications affecting the central nervous system (CNS) and peripheral nervous system (PNS). These occur at different stages of the infection.

**Compliance with ethics regulations:** Yes in clinical research.

## FC-152 Severe neurological SARS-COV2 patients in ICU: a monocenter retrospective Serie during the pandemic

### Laurent Camous^1^, Jean-David Pommier^1^, Floran Delamare^1^, Frederic Martino^1^

#### ^1^CHU de Guadeloupe, Les Abymes, France

##### **Correspondence:** Laurent Camous (laurent.camous@chu-guadeloupe.fr)

*Annals of Intensive Care* 2023, **13(Suppl 1):**FC-0152

**Rationale:** Since the original description of COVID 19, various clinical manifestations of SARS COv2 have been described. In large series of COVID 19 patients, neurological symptoms are reported in up to 10% of hospitalized patients. Early clinical reports on neurological abnormalities in ICU patients after discharge, showed that neurological lesions can be pleomorphic with both peripherical or central damages. Mechanisms of neurological damage during COVID 19 have been extensively discussed, but mostly on case reports.

**Patients and methods/materials and methods:** All patients admitted in ICU during the study period (2020–2022) for primary neurological failure (defined by glasgow score < 10) and SARS-COV2 infection were included. Clinical characteristics, ICU management and outcome were analysed.

**Results:** In our ICU, 950 consecutive COVID 19 patients had been hospitalized during the study period. Twenty patients (1.8%) were admitted for primary neurological failure (neurological COVID 19 group). Briefly, comparison between neurological COVID 19 group and overall cohort showed that neuro-COVID patients were older, had more comorbidities and were more intubated. 30% of the patients (n = 6) were admitted for severe stroke and 70% (n = 14) had encephalitis features. At ICU admission, complex status epilepticus was the most common clinical presentation (n = 10/20). In the neurological COVID 19 group, pulmonary SarsCOv2 involvement was lightly severe (< 25% pulmonary involvement). All patients had paraclinical exploration to precise mechanism of neurological failure: 10/14 patients had lumbar puncture (2/14 patients had lymphocytic meningitis and 10/14 had hyper-proteinorachia), 10/14 had cerebral MRI (5/10 without abnormalities and 5/10 with signs of encephalitis).

**Conclusion:** Neurological tropism and mechanisms of SARSCov2 cerebral damages have been discussed, but to date, many questions remain unanswered. In our center, status epilepticus was the main neurological manifestation in severe COVID 19 patients with primary neurological failure. Mechanisms and treatment remain unclear.

**Reference 1**

Picod A, Dinkelacker V, Savatovsky J, Trouiller P, Guéguen A, Engrand N. SARS-CoV-2-associated encephalitis: arguments for a post-infectious mechanism. Crit Care 2020;24(1):658.

**Reference 2**

Newcombe VFJ, Dangayach NS, Sonneville R. Neurological complications of COVID-19. Intensive Care Med 2021;47(9):1021–3.

**Compliance with ethics regulations:** Yes in clinical research.

## FC-153 Use of plasma apheresis in a Tunisian intensive care unit (ICU): clinical indications and applications

### Iyed Maatouk^1^, Wiem Nouira^1^, Maha Hamdi^1^, Manel Lahmar^1^, Hedia Ben Ahmed^1^, Zeineb Hammouda^1^, Abir Chihaoui^1^, Saoussen Ben Abdallah^1^, Fahmi Dachraoui^1^, Fekri Abroug^1^, Lamia Ouanes Besbes^1^

#### ^1^CHU Fattouma Bourguiba Monastir Tunisie, Monastir, Tunisie

##### **Correspondence:** Wiem Nouira (wiemnouira1@gmail.com)

*Annals of Intensive Care* 2023, **13(Suppl 1):**FC-0153

**Rationale:** Plasmapheresis is an extracorporeal technique for removing pathogenic macromolecules from blood. It is a treatment that has been used successfully for several decades in the management of a wide variety of diseases. The aim of our study was to describe clinical indications and practice of this technique in our ICU.

**Patients and methods/materials and methods:** We conducted a retrospective study from 2015 to 2022 in the ICU department of Fattouma Bourguiba of Monastir (Tunisia). All patients transferred from neurology and nephrology department outside COVID-19 pandemic to receive plasmapheresis were included.

**Results:** During the study period, 21 patients were admitted for plasmapheresis. The majority were male (66.3%). Their median age was 38 (interquartile range (IQR): 25–49) years. Median SAPSII was 13 (IQR: 6–13). Median length of stay was 10 (IQR: 10–23) days. The most frequent medical histories were myasthenia (19%) and respiratory diseases (19%), multiple sclerosis (9.5%) and neuromyelitis optica (9.5%). Five patients presented acute respiratory failure of whom 3 required mechanical ventilation. Only 1 patient presented an acute kidney injury. Indications for plasmapheresis were: myasthenia (42%), neuromyelitis optica (19%), polyradiculoneuritis (14.3%), multiple sclerosis (9.5%), vasculitis (ANCA) (4.8%), Retrobulbar Optic Neuropathy (4.8%) and Goodpasture syndrome (4.8%). The mean number of plasmapheresis was 3.8 ± 1 sessions. Patients’ condition after plasmapheresis has improved in 81%, worsened in 4.8% and was stable in 14.3%. Only 2 complications related to this technique were reported (thrombosis of the circuit). The evolution was favorable in the majority of cases (19 cases).

**Conclusion:** Plasmapheresis has shown to be efficacious in many diseases particularly in neurological conditions. Nevertheless, caution is required and their possible complications should be well monitored.

**Compliance with ethics regulations:** Yes in clinical research.

## FC-154 Neurological burden and outcomes of excessive beta-lactam serum concentration in critically ill patients: a prospective cohort study

### Clément Gaulin^1^, Yoann Zerbib^1^, Sandra Bodeau^1^, Benjamin Batteaux^1^, Anne-Sophie Lemaire-Hurtel^1^, Julien Maizel^1^, Loay Kontar^1^, Youssef Bennis^1^

#### ^1^CHU Amiens, Amiens, France

##### **Correspondence:** Clément Gaulin (clement.gaulin@hotmail.fr)

*Annals of Intensive Care* 2023, **13(Suppl 1):**FC-0154

**Rationale:** Neurotoxicity is a well-known concentration-dependent adverse effect of beta-lactams, but how excessive concentration of these antibiotics can burden the care of critically ill patients has not been thoroughly investigated.

**Patients and methods/materials and methods:** In a prospective monocentric cohort study, we examined whether excessive beta-lactam serum concentration contributes to the neurological status deterioration and complications of adult patients treated by beta-lactams in medical ICU without recent history of neurological disease. Excessive beta-lactam concentration was defined as a steady state serum concentration (at trough for drugs administered intermittently) that exceeded the upper limit of the therapeutic range recommended by the French Society of Pharmacology and Therapeutics (SFPT) and the French Society of Anaesthesia and Intensive Care Medicine (SFAR). Neurological status deterioration was defined as a change in the neurological Sequential Organ Failure Assessment score (nSOFA) of 1 or more between the day of treatment start at admission and the day of therapeutic drug monitoring performed 2 days after treatment initiation.

**Results:** We included 119 patients [median age: 65; males: 78 (65.5%)], admitted for acute respiratory distress [59 (49.6%)] or septic choc [25 (21%)]. In a multivariate regression analysis, excessive beta-lactam serum concentration was associated with neurological status deterioration [Odds ratio (OR) with 95% confidence interval (CI95): 9.58 (2.99–30.59), p = 0.0001]. Furthermore, excessive beta-lactam serum concentration was associated with time to discharge alive (β = 0.346, p = 0.0007) and, among mechanically ventilated patients discharged alive, with time to extubation following sedation withdrawal (β = 0.371, p = 0.0008).

**Conclusion:** These results confirm the clinical relevance of the upper concentration limit recommended for dose reduction and suggest that excessive accumulation of beta-lactams could complicate the management of septic patients in the ICU.

**Compliance with ethics regulations:** Yes in clinical research.

## FC-155 Non-traumatic disorders of consciousness in the intensive care unit: retro-prospective study

### Mohammed Abidi^1^, Ismail El Abbadi^1^, Rehab Haffar^1^, Firdaous Belkaid^1^, Kawtar Ziati^1^, Latifa Oualili^1^, Oussama Ssouni^1^, Tarik Dendane^1^, Khalid Abidi^1^, Amine Ali Zeggwagh^1^

#### ^1^Centre hospitalier universitaire Ibn Sina Rabat, Rabat, Maroc

##### **Correspondence:** Mohammed Abidi (dr.mohammed.abidi@gmail.com)

*Annals of Intensive Care* 2023, **13(Suppl 1):**FC-0155

**Rationale:** Disorders of consciousness (DoC) are common in critically ill patients and associated with high mortality. The aims of the study were to describe the epidemiological characteristics of the patients with non-traumatic DoC in medical intensive care unit, to identify its etiologies, and to determine the factors associated with prognosis.

**Patients and methods/materials and methods:** A retrospective-prospective study was conducted in the medical ICU of the University Hospital of Rabat, from January 2021 to December 2021. We include all patients presenting non-traumatic DoC with a Glasgow coma scale (GCS) under 15. To identify the factors associated with mortality, we compared 2 groups: survivors vs deceased patients.

**Results:** Out of 587 patients who were admitted in the medical ICU, 196 patients have been admitted for DoC (33.4%). 62 (31.6%) of patients were in a coma (GCS ≤ 8). The mortality rate was 41.8%. The Study shows that metabolic disorders were the most common cause of admissions and were observed in 96 (49.2%) patients. Structural lesions and diffuse deterioration of central nervous system were respectively observed in 64 (32 0.8%) and 35 (18%) patients. We found also that diabetic ketoacidosis was the most common cause of admissions (17%) among all admissions. Central nervous system infections represented the most common cause of admissions among patients with structural lesions and the second etiology of all admissions categories, respectively in 48.4% and 15.9%. Also, acute poisoning represented the third etiology of DoC and was observed in 21 patients (10.7%). In multivariate analysis, the independent factors associated with mortality were: the structural lesions (OR = 8.08; IC 95% = 1.57–41.55; p = 0.01), low prothrombin ratio (OR = 0.97; IC 95% = 0.95–42.55; p = 0.004). However, there is a tendency for higher mortality in patients with elevated C-Reactive Protein (OR = 1.005; IC 95% = 1.00–1.01; p = 0.052).

**Conclusion:** The incidence and mortality of non-traumatic DoC in medical intensive care unit remain high. Concerning the etiologies, nearly half of the patients had a metabolic disorder. Also, this study demonstrated that structural lesions and low prothrombin ratio were independently associated with high mortality.

**Compliance with ethics regulations:** Yes in clinical research.

## FC-156 Bispectral index monitoring in patients with acute respiratory distress syndrome requiring continuous neuromuscular blockade

### Laura Benichou^1^, Astrid Bertier^1,3,4^, Femke Mestre^1^, Maryline Couette^1,3^, Antoine Gaillet^1,3,4^, Muriel Paul^1^, Rachida Ouedraogo^1,3,4^, Aline Alves^1,3,4^, Ann Cécile Pallud^1,3,4^, Damien Carras^1,3,4^, Jérôme Cecchini^2^, Keyvan Razazi^1,3,4^, Clément Ourghanlian^1^, Armand Mekontso Dessap^1,3,4^

#### ^1^Hôpitaux universitaires Henri Mondor - APHP, Créteil, France; ^2^Centre hospitalier intercommunal de Créteil, Créteil, France; ^3^Univ Paris Est Créteil - CARMAS, Créteil, France; ^4^Univ Paris Est Créteil – INSERM - IMRB, Créteil, France

##### **Correspondence:** Astrid Bertier (astrid.bertier@aphp.fr)

*Annals of Intensive Care* 2023, **13(Suppl 1):**FC-0156

**Rationale:** This observational study aimed to evaluate the feasibility and efficacy of a sedation-analgesia monitoring protocol based on Bispectral Index (BIS) in patients with acute respiratory distress syndrome (ARDS) receiving continuous neuromuscular blocking agents (NMBA).

**Patients and methods/materials and methods:** 43 adult patients were included. The primary outcome was the percentage of patients achieving the BIS therapeutic target (40–60) at 24 h of NMBA initiation. Secondary outcomes were the frequency of BIS measurements in daily practice and the subsequent post-BIS changes in sedative dosage.

**Results:** Over the 48 h of continuous NMBA, 298 BIS measurements were recorded and 69.8% of patients had a BIS value within the therapeutic target at 24 h. The median number of BIS per patient was 7 [5–8], whereas the protocol expected 12 to 16 if monitored every 3–4 h as prescribed. No difference was found between groups with lower (≤ 25%, n = 22) or higher (> 25%, n = 21) percentage of BIS values outside the target, except for more females in the latter. The former group showed significantly higher median number of BIS per patient and shorter time to obtain a BIS within the therapeutic target.

**Conclusion:** We demonstrated that a treatment protocol that allows nurses to drive sedation according to a BIS monitor-based algorithm in critically ill patients requiring continuous NMBA for ARDS was effective; most patients reached BIS target at 24 h of NMBA initiation. A close monitoring of the sedation depth in those patients is essential given that nearly one third were outside the target at 24 h.

**Reference 1**

Bass, S. et al. Bispectral index for titrating sedation in ARDS patients during neuromuscular blockade. Am. J. Crit. Care Off. Publ. Am. Assoc. Crit.-Care Nurses 28, 377–384 (2019).

**Compliance with ethics regulations:** Yes in clinical research.

## FC-157 Effect of the course of mechanical power on 30-day mortality in COVID-19 patients with ARDS

### Martin Mombrun^1,2,4^, Olivier Lucidarme^2^, Michel Ramakers^1^, Bertrand Canoville^3^, Damien Du Cheyron^4^, Anaïs R. Briant^4^, Jean-Jacques Parienti^4^, Cédric Daubin^4^

#### ^1^Hôpital Mémorial Saint-Lô, Saint-Lô, France; ^2^Hôpital Robert Bisson, Lisieux, France; ^3^Hôpital Avranches Granville, Avranches, France; ^4^CHU Caen Côte de Nacre, Caen, France

##### **Correspondence:** Martin Mombrun (mombrun.martin@gmail.com)

*Annals of Intensive Care* 2023, **13(Suppl 1):**FC-0157

**Rationale:** Mechanical power is a recently introduced surrogate tool to assess the intensity of mechanical ventilation. Mechanical power is reported to be associated with ventilator-induced lung injury (VILI). Therefore, to target a low mechanical power could be useful to prevent the risk of death from VILI in ARDS. So, we investigated the association between the course of mechanical power during the first days of mechanical ventilation and the 30-day mortality in COVID-19 ARDS patients.

**Patients and methods/materials and methods:** A retrospective analysis of a bicentric cohort study in 2 French ICUs. We collected data from mechanical ventilation from day 1 to day 5 to compute daily mechanical power and its three fractions (static elastic, dynamic elastic and resistive), according to the surrogate formula as follows: Mechanical power = 0.098 × Vt × RR × [PEEP + ½ × ΔP + (Ppeak − Pplat)] J min^−1^. We tested the association of mechanical power on day 1 with 30-day mortality (primary outcome), and the association of the course of mechanical power from day 1 to day 5 with mortality. Then, ROC curves were performed to evaluate the performance of mechanical power and its fractions to predict death over time. Day 1 was set as the day where ARDS diagnosis criteria was met.

**Results:** Data from 109 patients were analyzed, with median age 62 years [Q1–Q3: 53–71], SAPS II score 38 [31–44], SOFA score 7 [5–8] and median lowest PaO_2_/FiO_2_ ratio on day 1 to 5 of 113 [93–138]. 30-day mortality was 22.0%. On day 1, median mechanical power was 35 [30–43] J min^−1^ and increased over the 5 days to reach 42 [34–51] J min^−1^ (p < 0.01). On day 1, mechanical power was not associated with 30-day mortality. From day 3, mechanical power was significantly higher in non-survivors (for example at day 3, 45 J min^−1^ [39–47] versus 40 J min^−1^ [32–44] (p = 0.03)). Interestingly, increase in mechanical power of more than 10.2 J min^−1^ from day 1 to day 3 was associated with lower survival probability (log-rank test, p < 0.001) (Figure 1). The dynamic elastic mechanical power (DEMP) at day 2 had a good predictor of death (AUC = 0.76 95%Confidence Interval [0.65–0.87], p < 0.001). The survival probability was lower in patients with a DEMPS > 7 J min^−1^ on day 2 (log-rank test, p < 0.001).

**Conclusion:** In COVID-19 ARDS patients, the course of mechanical power was associated with 30-day mortality. Other studies are needed to confirm this result.

**Reference 1**

COVID-ICU Group on behalf of the REVA Network and the COVID-ICU Investigators. Clinical characteristics and day-90 outcomes of 4244 critically ill adults with COVID-19: a prospective cohort study. Intensive Care Med. 2021;47(1):60–73.

**Reference 2**

Costa ELV, Slutsky AS, Brochard LJ, Brower R, Serpa-Neto A, Cavalcanti AB, et al. Ventilatory variables and mechanical power in patients with acute respiratory distress syndrome. Am J Respir Crit Care Med. 2021;204(3):303–11.

**Compliance with ethics regulations:** Yes in clinical research.

**Figure 1 (abstract FC-157):**
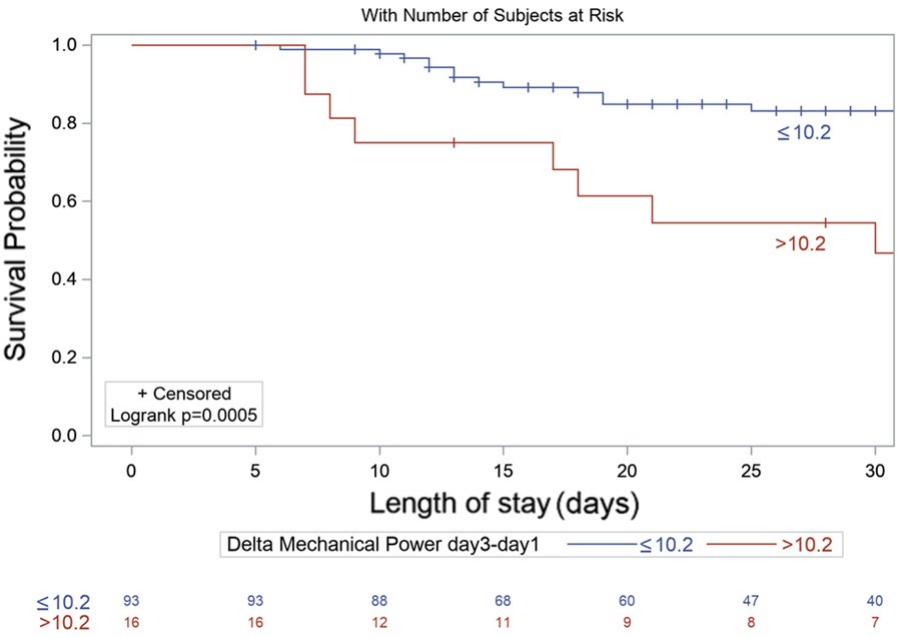
Kaplan–Meier surviving curves according to mechanical power delta from day 1 to day 3

## FC-158 Effect of the course of ventilatory ratio on 30-day mortality in COVID-19 patients with ARDS

### Martin Mombrun^1,2,4^, Nicolas Despierre^3^, Olivier Lucidarme^2^, Michel Ramakers^1^, Damien Du Cheyron^4^, Anaïs R Briant^4^, Jean-Jacques Parienti^4^, Cédric Daubin^4^

#### ^1^Hôpital Mémorial Saint-Lô, Saint-Lô, France; ^2^Hôpital Robert Bisson, Lisieux, France; ^3^Hôpital Avranches-Granville, Avranches, France; ^4^CHU Côte de Nacre, Caen, France

##### **Correspondence:** Martin Mombrun (mombrun.martin@gmail.com)

*Annals of Intensive Care* 2023, **13(Suppl 1):**FC-0158

**Rationale:** Increased pulmonary dead space fraction (VD/VT) is associated with a higher risk of death in ARDS. However, the measurement of VD/VT is complex in clinical practice. Ventilatory ratio is suggested as surrogate tool to monitor ventilator efficiency. This index is well correlated with VD/VT. Whether ventilatory ratio is associated with mortality in COVID-19 ARDS patients remains unclear. Therefore, we investigated the association between the course of ventilator ratio during the first days of mechanical ventilation and the 30-day mortality in these patients.

**Patients and methods/materials and methods:** A retrospective analysis of a cohort study in 4 French ICUs. We collected data from mechanical ventilation from day 1 to day 5 to compute daily ventilatory ratio according to the formula as follows: Ventilatory ratio = [minute ventilation (ml.min-1) × PaCO_2_ (mmHg)]/Predicted body weight (kg) × 100 × 37.5). We tested the association of ventilatory ratio on day 1 (primary outcome) with 30-day mortality, and the association of the course of ventilatory ratio from day 1 to day 5 with mortality. We also compared ventilatory ratio on day 1 with estimated VD/VT calculated according to the rearranged alveolar gas Eq. (1). Then, we constructed ROC curves to evaluate the performance of ventilatory ratio to predict death over time. Day 1 was set as the day where ARDS diagnosis criteria was met.

**Results:** Data from 242 patients were analyzed, with median age 68 years [58–73], SAPS II score 38 [31–46], SOFA score 7 [4–8] and median lowest PaO_2_/FiO_2_ ratio on day 1 to 5 of 118 [94–149]. 30-day mortality was 34%. On day 1, median ventilatory ratio was 1.97 [1.57–2.35] and increased over the 5 days to reach 2.17 [1.88–2.49] (p < 0.001). On day 1, the ventilatory ratio was not associated with 30-day mortality. Ventilatory ratio was well correlated with VD/VT with linear regression correlation coefficient r = 0.39, p < 0.001. Median VD/VT on day 1 was 62.4% [55.1–68.3] but was not associated with higher risk of death at day 30. Progression of ventilatory ratio from day 1 to day 5 was not associated with mortality (0.192 [− 0.01–0.458] (alive) versus 0.281 [− 0.05–0.712], p = 0.20). AUROC for progression of ventilatory ratio from day 1 to day 5 was 0.56 (p = 0.12).

**Conclusion:** Ventilatory ratio was not associated with 30-day mortality in this cohort of COVID-19 ARDS patients. Other studies are needed to confirm this result.

**Reference 1**

Fenn RHWO, Otis AB: A theoretical study of the composition of the alveolar air at altitude. Am J Physiol 1946, 146:637–653.

**Reference 2**

Morales-Quinteros L, Schultz MJ, Bringué J, Calfee CS, Camprubí M, Cremer OL, et al. Estimated dead space fraction and the ventilatory ratio are associated with mortality in early ARDS. Annals of Intensive Care. 2019 Nov 21;9(1):128.

**Compliance with ethics regulations:** Yes in clinical research.

**Figure 1 (abstract FC-158):**
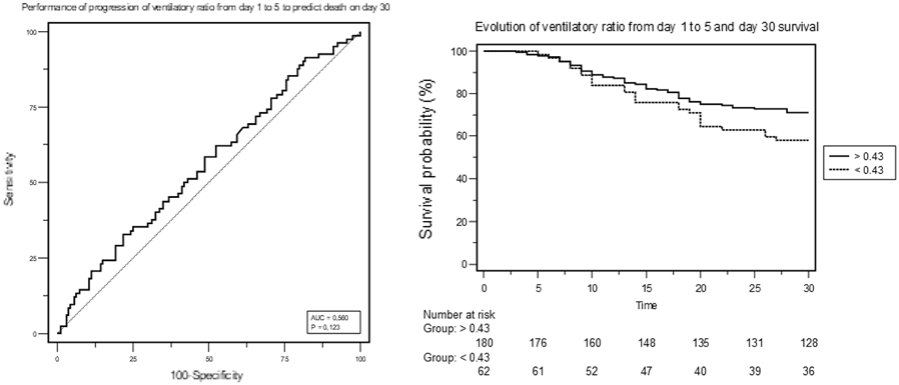
ROC curve and Kaplan–Meier survival curves according to progression of ventilatory ratio from day 1 to 5

## FC-159 Ventilatory ratio and pre-ICU management delay as early predictors of mortality in invasively ventilated COVID-19-related ARDS patients. A retrospective observational cohort study

### Radhouane Toumi^1,2^, Khaoula Meddeb^1,2^, Nabil Bouguezzi^1^, Azer Yacoub^1^, Rihab Rajah^1^, Emna Ennouri^1,2^, Imen Ben Saida^1,2^, Mohamed Boussarsar^1,2^

#### ^1^Medical Intensive Care Unit, Farhat Hached University Hospital, Sousse, Tunisie; ^2^Research Laboratory N° LR12SP09. Heart Failure. Farhat Hached University Hospital, Sousse, Tunisie

##### **Correspondence:** Radhouane Toumi (Radhouane.toumi@gmail.com)

*Annals of Intensive Care* 2023, **13(Suppl 1):**FC-0159

**Rationale:** Coronavirus disease 2019 (COVID-19) has been a burden on healthcare systems with numerous hospital and intensive care unit (ICU) admissions, the most severe of which requiring invasive mechanical ventilation (IMV). The ventilatory ratio (VR), a novel and simple tool that reflects ventilatory efficiency, is directly correlated to worse outcome in patients on IMV. The aim of this study is to determine whether VR is a predictive factor of mortality in COVID-19-related ARDS patients requiring IMV.

**Patients and methods/materials and methods:** This is a retrospective observational cohort study carried out in the medical ICU of a University hospital in a low-middle income country, between March 3rd 2020 and December 31st 2021. All consecutive patients with confirmed SARS-CoV-2 infection admitted to the ICU and requiring IMV were included. Demographic, clinical, physiological and ventilator data were collected. Univariate and multivariate cox regression models and Kaplan–Meier overall survival curves were used to identify risk factors of mortality.

**Results:** 732 patients were admitted to the ICU, 465 (63.5%) had a confirmed COVID-19 infection, 247 (53.1%) required IMV. Median [IQR] age was 67 [59–74] years; and pre-ICU management delay, 4 [3–7] days. 149 (60.3%) were on standard oxygenation; High-Flow Nasal Oxygen, 20 (8.1%); Non-Invasive Positive Pressure Ventilation, 56 (22.7%) and already on IMV, 15 (6.1%). SAPS II, 31 [27–37]; P/F ratio, 96 [76.3–139.8]. At day 1 of IMV, tidal volume, 6.5 [6–7] ml; PEEP, plateau and driving pressures, respectively, 10 [8–10], 27 [24–28] and 16 [14–19] cmH_2_O; P/F ratio, 126.3 [90.3–170.8]; ventilatory ratio, 2.36 ± 0.89 and mechanical power, 36.0 [29.3–45.2] J/min. Median length of stay, 12 [7–17] days and median IMV duration, 8 [5–12] days. Mortality rate, 84.6%. Early independent factors predicting mortality were age, pre-ICU management delay and ventilatory ratio at day1 of IMV.

**Conclusion:** In invasively ventilated COVID-19-related ARDS patients, older age, longer pre-ICU management delay and higher ventilatory ratio at day 1 of invasive mechanical ventilation, were independent risk factors of mortality.

**Compliance with ethics regulations:** Yes in clinical research.

## FC-160 sRAGE and severity of COVID-19 patients admitted in intensive care unit, a retrospective observational study

### Fatma Gamara^1^, Olivier Mascle^1^, Marina Brailova^1^, Julien Domitile^1^, Kévin Grapin^1^, François Thouy^1^, Laure Calvet^1^, Olivia Guido^1^, Bonnet Benjamin^1^, Bertrand Evrard^1^, Vincent Sapin^1^, Bertrand Souweine^1^, Claire Dupuis^1^

#### ^1^CHU Clermont Ferrand, Clermont-Ferrand, France

##### **Correspondence:** Claire Dupuis (cdupuis1@chu-clermontferrand.fr)

*Annals of Intensive Care* 2023, **13(Suppl 1):**FC-0160

**Rationale:** The soluble receptor of advanced glycation end products (sRAGE) is a biomarker shown to predict ARDS. Until nowadays, few studies focused on sRAGE levels in severe COVID-19 pneumonia. In that context, the aim of our study was to assess the performance of sRAGE and others inflammatory biomarkers to predict the severity of COVID-19 -Acute hypoxemic respiratory failure (AHRF) in terms of risk of intubation and mortality.

**Patients and methods/materials and methods:** It is a retrospective single center study. All consecutive adult patients aged ≥ 18 years admitted between March 2020 and February 2021 into the medical ICU of Clermont Ferrand, France, for SARS-CoV-2 pneumonia, with an ICU length of stay (LOS) > 48 h were enrolled. sRAGE, plasma coagulation biomarkers (D-Dimers and fibrinogen levels) and other serum inflammatory biomarkers (CRP, ferritin, IL-1, IL-6, IL-10, PCT, mHLA-DR) were collected on ICU admission. Their levels depending on PaO_2_/FiO_2_ were reported. The association between those biomarkers and (1) day-90 mortality and (2) risk of intubation and/or ICU death were assessed by ROC curves, Cox survival model (1) or subdistribution survival model (2).

**Results:** 150 patients were included, with a median age of 71 years [64; 76], and SAPS II score of 35.6 [29; 44]. Only fibrinogen levels and m-HLA-DR levels varied according to the severity of COVID-19. 36.6% patients died at day 90. sRAGE (AUC = 0.72 [0.63; 0.81]), Il10 (AUC = 0.71 [0.62; 0.8] and Il6/HLA DR ratio (AUC = 0.69 [0.61; 0.78) predicted the most death at day 90. A high level of sRAGE was associated the death at day 90 (HR = 3.86 [2.01; 7.41], p < 0.01). Similarly, the risk of intubation and/or ICU death were also the best predicted by Il10 (AUC = 0.71 [0.61; 0.8]) and sRAGE (0.78 [0.69; 0.86]). sRAGE was association with risk of Intubation and or ICU death (SubHR = 4.83[2.10; 11.11], p < 0.01).

**Conclusion:** sRAGE concentrations, but also Il10 and Il6/mHLA DR ratio seemed interesting to predict the risks of intubation and death in critically ill COVID-19 patients.

**Compliance with ethics regulations:** Yes in clinical research.

## FC-161 Type-H, and Type-L COVID-19: comparison of clinical features and outcomes in critically ill patients

### Yosri Ben Ali^1^, Amira Hmaidi^1^, Dhouha Ben Braiek^1^, Hend Zorgati^1^, Sourour Bel Haj Youssef^1^, Azer Yaacoub^1^, Rahma Ben Jazia^2^, Amani Kacem^2^, Abderrahmen Daadoucha^3^, Jihene Ayachi^1^

#### ^1^Medical Intensive Care Unit, Ibn El Jazzar University Hospital, Kairouan, Tunisie; ^2^Pulmonology Department, Ibn El Jazzar University Hospital, Kairouan, Tunisie; ^3^Radiology Department, Ibn El Jazzar University Hospital, Kairouan, Tunisie

##### **Correspondence:** Jihene Ayachi (ayachijihen@gmail.com)

*Annals of Intensive Care* 2023, **13(Suppl 1):**FC-0161

**Rationale:** Recent studies have shown that there are two types of COVID-19 responses: an H phenotype with high lung elastance, and an L phenotype with low measures. Identification of distinct clinical phenotypes may facilitate management and improve outcomes. Aim: To identify phenotypes across critically ill COVID-19 patients and to compare their characteristics and outcomes.

**Patients and methods/materials and methods:** A retrospective observational study conducted from September 19th, 2020 to December 31st, 2021 in a 9-bed medical ICU at teaching hospital including adult patients admitted for acute respiratory distress syndrome (ARDS) related COVID-19 pneumonia. Data were collected by reviewing the medical patients’ charts. Chest computed tomography (CT) scan findings were used to define and divided into two groups (type-L and type-H) according to their findings. Statistically significant variables found in univariate analysis were used to compare the two groups.

**Results:** During the study period, 172 patients were admitted; 57(33.1%) with H-type and 111 (64.5%) with L-type. Mean age and man predominance of Type-H and Type-L was respectively 53.5 ± 14.1 versus 50.1 ± 14.2 (p = 0.14) and 61.4% vs 51.9%. There was no significant difference between the two groups in terms of socio-demographic and clinical features according to lung structure. Non-invasive ventilation (NIV) was used in 35.1% of patient with H-type versus 21.6% with p = 0.06. Invasive mechanical ventilation was required in 14 (29.8%) of cases. In the two groups, the mean PEEP, plateau pressure, driving pressure and pulmonary compliance were respectively:10 cmH_2_O, 28 cmH_2_O, 17 cmH_2_O and 22 ml/cmH_2_O with no significant difference. The duration of symptom onset to ICU admission for ARDS was 8 days in the two groups. The mean ICU length of stay was longer in H-type (13.5 vs 9.8 days; p = 0.006). H phenotype was significantly associated with vasoactive drugs use (p < 0.001), invasive mechanical ventilation requirement (p < 0.001), and mortality rate (p < 0.001).

**Conclusion:** The current study demonstrated that H phenotype in COVD19 patients is more frequently associated with vasoactive drugs use, invasive mechanical ventilation and mortality. Future research is needed to determine the utility of these phenotypes in clinical practice.

**Compliance with ethics regulations:** Yes in clinical research.

## FC-162 Procalcitonin cut-off level and the high risk of mortality in 30 days, in critically ill patients infected by covid 19

### Amine Elmouhib^1^, Younes Oujidi^1^, Inass Arhoun El Haddad^1^, Ilyass Laaribi^1^, Houssam Bkiyar^1^, Brahim Housni^1^

#### ^1^CHU Mohammed VI Oujda, Oujda, Maroc

##### **Correspondence:** Inass Arhoun El Haddad (iarhounelhaddad@gmail.com)

*Annals of Intensive Care* 2023, **13(Suppl 1):**FC-0162

**Rationale:** Procalcitonin (PCT) is a biomarker that has been proposed as a helpful tool in inflammatory Clinique cases especially patients with pulmonary bacterial infection. Our objective is to assess the relationship between Procalcitonin and 30-day mortality in patients admitted to the resuscitation department of our hospital infected by the SARS COV 2 Virus.

**Patients and methods/materials and methods:** All Patients admitted to the ICU for acute respiratory failure related to SARS-CoV-2 pneumonia between June 1st 2020 and January 31st 2022 were retrospectively included. Files were retrospectively reviewed from Hospital Medical History system as well as laboratory findings, radiological results and outcome data. We analyzed the relationship between PCT values and mortality at 30 days. Continuous and categorical variables were presented as median (interquartile range, IQR) and n (%). We used univariate tests by chi-square, Fisher exact, Mann–Whitney tests or t tests to compare differences between groups according to the characteristics of each variable. Variables that reached a level of significance greater than 0.20 were introduced into a multivariable regression with a forced input of PCT in order to identify the variables independently associated with mortality.

**Results:** Over a period of 19 months since June 1, 2020, 1253 patients with the SARS CoV-2 virus were hospitalized in the ICU Department of the hospital, the mortality rate within 30 days of their admission was 30.9%. There was a male predominance with Sex Ratio M/F 2.09, average age is 50 years, all patients had a PCT measurementupon admission, the average PCT was 3.01 ng/ml in survivors and 6.64 ng/ml in non-survivors. Non-survivors had a significantly higher PCT level ≥ 0.5 ng/ml than survivors (p = 0.000053) (Table 1). The PCT prognostic value at a threshold of 0.5 ng/mL showed a sensitivity of 69.9%, a specificity of 55.55% and a positive likelihood ratio of 1.57 as well as an Odds Ratio of 2.9; a threshold of 1 ng/mL showed a sensitivity of 81.59% but a specificity of 40.55%, whereas a threshold of 5 increases the sensitivity to 92.7 with a loss of specificity to 15 0.55.

**Conclusion:** We conclude that a high level of Procalcitonin more than 0.5 ng/ml increased mortality at 30 days after admission in ICU for SARS CcV-2 patients, and it could be used as criteria for emergency triage of patients with other scores for ICU COVID patient admission.

**Compliance with ethics regulations:** Yes in clinical research.

**Figure 1 (abstract FC-162):**
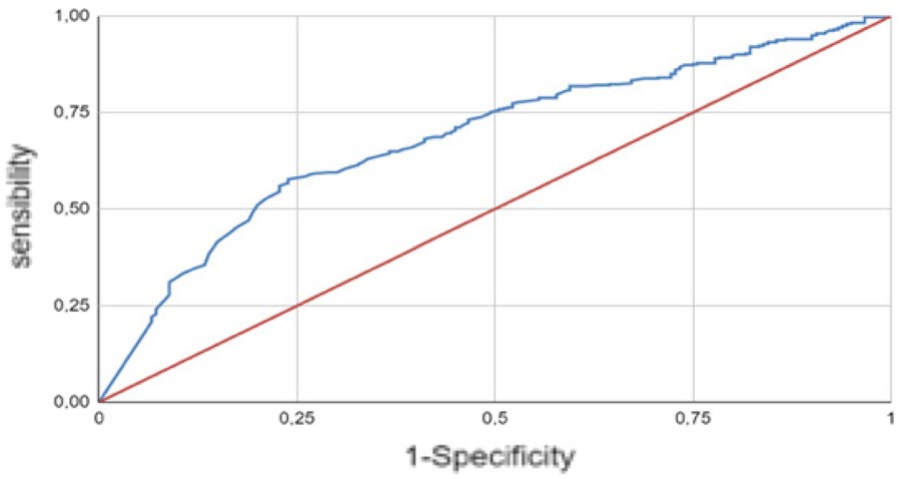
Receiver operating characteristic curve of procalcitonin

## FC-163 Cardiovascular phenotypes in COVID-19 related acute respiratory distress syndrome. An alternative approach for ECMO indication during ICU surge

### Matthieu Petit^2^, Annick Legras^3^, Edouard Jullien^2^, Bruno Evrard^4^, Cyril Charron^2^, Anousone Daulasim^2^, Misylias Bouaoud^3^, Maeva Gourraud^4^, Marine Goudelin^4^, Philippe Vignon^4^, Antoine Vieillard-Baron^2^

#### ^1^Service de Médecine Intensive Réanimation, Hôpital Ambroise Paré, Assistance Publique Hôpitaux de Paris, Boulogne-Billancourt, France; ^2^Service de Médecine Intensive Réanimation, Centre Hospitalier Universitaire de Tours, Tours, France; ^3^Service de Réanimation Polyvalente, Centre Hospitalier Universitaire de Limoges, Limoges, France; ^4^Service de Cardiologie, Centre Hospitalier Universitaire de Tours, Tours, France

##### **Correspondence:** Matthieu Petit (matthieu.petit@aphp.fr)

*Annals of Intensive Care* 2023, **13(Suppl 1):**FC-0163

**Rationale:** Coronavirus disease 2019 (COVID-19) related acute respiratory distress syndrome (C-ARDS) is associated with high mortality. Extra-Corporeal Membrane Oxygenation (ECMO) has been proposed in this setting but optimal criteria to select target patients remain unknown. Our hypothesis is that evaluation of right ventricular (RV) function by echocardiography could be helpful. Aims of our study were to report incidence of patients potentially eligible to ECMO according to Eolia criteria, to report their outcome and to identify a specific phenotype with a particular high mortality, which could the adequate target for ECMO.

**Patients and methods/materials and methods:** Retrospective observational study involving 3 intensive care units (ICU) of tertiary teaching hospital in France. Consecutive patients with confirmed SARS-CoV-2 infection between March 2020 and March 2021 admitted to the ICU for invasive mechanical ventilation with a diagnosis of ARDS and with available echocardiography were included. Patients eligible to ECMO were identified according to the EOLIA criteria. Hierarchical clustering on principal components based on baseline characteristics, clinical respiratory and echocardiographic data at the worst PaO_2_/FiO_2_ ratio during the first 7 days of mechanical ventilation was used to identify different phenotypes of patients. Kaplan–Meier survival curves were used to analyze patient outcome according to clusters as well as a Cox model on 90-days mortality.

**Results:** 915 patients were hospitalized for COVID-19, 418 of them with ARDS. A total of 283 patients with available echocardiography were enrolled in the study. One hundred and ninety-one patients (67.5%) were eligible to ECMO with a day-90 mortality of 44%, among them only 18 (9%) received ECMO with the same day-90 mortality. Three different clusters were identified, including one with a remarkable respiratory severity and RV overloading (cluster 3, n = 78 patients). After adjustment for confounding variables, this cluster had higher 90-days mortality (58%, HR 3.44 [2.84; 4.18], p < 0.001).

**Conclusion:** We identified a cluster of patients with severe RV overload and respiratory impairment with a particular poor outcome, which could help physicians in determining when and in whom initiate ECMO. This represented 29% of the cohort compared to 67% of patients who met the Eolia criteria. How this better characterization could change management and prognosis should be evaluated in the future.

**Compliance with ethics regulations:** Yes in clinical research.

## FC-164 Epidemiological characteristics and prognosis of severe acute respiratory infection in the aftermath of COVID-19 pandemic in a Tunisian MICU

### Nabil Bouguezzi^1,2^, Emna Ennouri^1,2^, Salma Gallas^1,2^, Radhouane Toumi^1,2^, Khaoula Meddeb^1,2^, Rym Chelbi^1,2^, Latifa Maazaoui^3^, Ilhem Boutiba^4,5^, Salma Abid^4,5^, Mohamed Boussarsar^1,2^

#### ^1^University of Sousse, Faculty of Medicine of Sousse, Sousse, Tunisia; ^2^Farhat Hached University Hospital, Medical Intensive Care Unit, Research Laboratory “Heart Failure”, LR12SP09, 4000, Sousse, Tunisia; ^3^National Influenza Centre-Tunis, Unit of Virology, Microbiology Laboratory, Charles Nicolle’s Hospital, Tunis, Tunisia; ^4^Primary Health Care Directorate, Tunis, Tunisia; ^5^Faculty of Medicine, University of Tunis El Manar, Tunis, Tunisia

##### **Correspondence:** Nabil Bouguezzi (dr_nabil@live.fr)

*Annals of Intensive Care* 2023, **13(Suppl 1):**FC-0164

**Rationale:** Recent experience with COVID-19 pandemic highlighted the importance of close surveillance for Severe Acute Respiratory Infection (SARI), to support seasonal influenza control. Routine pathogen monitoring, in the intensive care unit (ICU), can provide insight into the burden and severity of SARI infections. The aim of this study is to characterize the epidemiology of SARI, in a Tunisian medical ICU throughout the 2022/2023 influenza season.

**Patients and methods/materials and methods:** This is a prospective observational study. Active surveillance was conducted at one of the four nationwide sentinel-MICUs in Tunisia from October 2022 to January 2023, in a 16-bed MICU. All consecutive patients aged over 16 years old, with SARI, were screened, within 24 h of MICU admission, by an RT-PCR SARS-CoV-2/Flu Multiplex Assay for the presence of 16 respiratory viruses in nasopharyngeal swabs or endotracheal aspiration. Epidemiological and clinical information were prospectively collected on specific forms. Patients were divided into two groups: SARI with confirmed pathogens group and SARI without confirmed pathogens group. Univariate analysis was used to identify factors associated with ICU mortality in SARI patients.

**Results:** Over 177 admitted patients during the study period, 95 SARI patients were enrolled. Mean age, 60 ± 17 years; 56 (59%) male; 82 (86.3%) have at least one underlying chronic medical condition. The most frequent symptom was cough 87(91.6%). 42 patients (44.2%) tested positive for at least one pathogen and 10 (10.5%) for multiple pathogens. The pathogens identified most frequently were, Flu A/H3N2, 14 (33.3%); human rhinovirus (HRhV), 10 (23.8%); Flu A/pH1N1, 9 (21.4%) Flu B, 4 (9.5%); respiratory syncytial virus (RSV) type B, 4 (9.5%); SARS-CoV-2, 3 (7.1%); human coronavirus (HCoV) type OC43, 1 (2.4%); adenovirus (AdV) 1 (2.4%); parainfluenza virus (PIV) type 1, 1 (2.4%); PIV type 2, 1 (2.4%); PIV type 3, 1 (2.4%); PIV type 4, 1 (2.4%). 10 (10.5%) of SARI patients developed ARDS. 66 (69.5%) were managed by NIV and 36 (37.9%) underwent invasive mechanical ventilation. Median MICU length of stay was 6 [4–10] days. MICU mortality was higher in SARI with confirmed pathogens compared with those without, (12 (28.6%) vs 6 (11.5%), p = 0.037).

**Conclusion:** The present SARI surveillance study identified Flu A/H3N2 and HRhV as the main pathogens detected in SARI patients hospitalized in a MICU in Tunisia. Multi-pathogen surveillance among SARI patients can provide useful information on SARI etiologies and epidemiology to improve healthcare system preparedness and patients’ prognosis.

**Compliance with ethics regulations:** Yes in clinical research.

## FC-165 Patients with SARS-CoV2 admitted to the ICU: analysis according to the extent of lung parenchymal lesions on chest CT scan, and clusters of patients at admission

### Kévin Grapin^1^, Romain De Bauchene^1^, Benjamin Bonnet^1^, Lucie Cassagnes^1^, Laure Calvet^1^, François Thouy^1^, Radhia Bouzgarrou^1^, Bertrand Evrard^1^, Mireille Adda^1^, Bertrand Souweine^1^, Claire Dupuis^1^

#### ^1^CHU Clermont Ferrand, Clermont-Ferrand, France

##### **Correspondence:** Claire Dupuis (cdupuis1@chu-clermontferrand.fr)

*Annals of Intensive Care* 2023, **13(Suppl 1):**FC-0165

**Rationale:** Inconsistent results from COVID-19 studies raise the issue of patient heterogeneity. The objectives of this study were to measure the prognostic value of the extent of lung parenchymal lesions on computed tomography (CT), and to identify homogeneous subgroups of patients (clusters) and compare their outcomes.

**Patients and methods/materials and methods:** It was a retrospective single center study, achieved in the Medical ICU of the university hospital of Clermont-Ferrand, France. All consecutive adult patients aged ≥ 18 years, admitted between March 20th, 2020 and August 31th, 2021 for COVID-19 pneumonia were included. Their characteristics at baseline, during ICU stay and outcomes at day-60 were recorded. On the chest CT performed at admission the extent of lung parenchyma lesions was established by artificial intelligence software. Clusters were determined by hierarchical clustering on principal components using principal component analysis of admission characteristics including plasma interleukin-6, HLA-DR monocytic–expression rate (mHLA-DR) and the extent of lung parenchymal lesions. Factors associated with day-60 mortality were investigated by univariate survival analysis.

**Results:** 270 patients were included. The extent of lung parenchymal lesions was associated with a decrease in the PaO_2_/FiO_2_ ratio (p < 0.01), fewer ventilatory-free days (p = 0.03) and a higher death rate at day-60 (p = 0.01). Three clusters were identified. Cluster 1 (obese patients, with moderate hypoxemia, moderate extent of lung parenchymal lesions, no inflammation and no downregulation of mHLA-DR) had a better prognosis at day-60 (HR = 0.26 [0.15; 0.46], p < 0.01) while cluster 2 (older patients with comorbidities, moderate extent of lung parenchyma lesions but significant hypoxemia, inflammation and downregulation of mHLA-DR) and cluster 3 (patients with severe parenchymal disease, hypoxemia, inflammatory reaction and downregulation of mHLA-DR) presented an increased risk of mortality (HR = 2.03 [1.34; 3.07], p < 0.01 and HR = 1.55 [1.02; 2.36], p = 0.04, respectively).

**Conclusion:** The extent of lung parenchyma lesions in CT was associated with a poorer prognosis. Three clusters with distinct characteristics and outcomes were identified. Such clusters could facilitate the identification of targeted populations for future trials.

**Compliance with ethics regulations:** Yes in clinical research.

## FC-166 Impact of immunosuppressive regimen on ICU acquired pneumonia in critically ill COVID-19

### Antoine Villa^1^, Wulfran Bougouin^2^, Tomas Urbina^1^, Vincent Bonny^1^, Paul Gabarre^1^, Louai Missri^1^, Jean-Luc Baudel^1^, Jean-Claude Buzzi^1^, Bertrand Guidet^1^, Eric Maury^1^, Hafid Ait-Oufella^1^, Jeremie Joffre^1^

#### ^1^Hôpital Saint-Antoine APHP, Paris, France; ^2^Ramsay Générale de Santé, Hôpital Privé Jacques Cartier, Massy, France

##### **Correspondence:** Jeremie Joffre (jeremie.joffre@aphp.fr)

*Annals of Intensive Care* 2023, **13(Suppl 1):**FC-0166

**Rationale:** Immunosuppressors (IS) such as Dexamethasone (DXM), Tocilizumab, and high-dose methylprednisolone boli (HDMB), are used in COVID-19-related acute respiratory distress syndrome (ARDS). This study aimed to determine whether COVID-19 ARDS-related combined IS therapy was associated with an increased incidence of ICU-acquired pneumonia (IAP).

**Patients and methods/materials and methods:** We retrospectively analyzed COVID-19 ARDS admitted to ICU from March 2020 to April 2022. Patients’ and IAP characteristics were analyzed according to five IS regimens: No IS, DXM alone, DXM + HDMB, DXM + tocilizumab, and DXM + tocilizumab + HDMB. To investigate the role of IS on IAP incidence, we performed a multivariate logistic regression and built a propensity score. Ultimately, we used a conditional logistic regression after pairing on the propensity score.

**Results:** 496 COVID-19 ARDS were included. Regarding the IS therapy, 12.7% received no IS, 43% DXM alone, 21.6% DXM + HDMB, 15.5% DXM + tocilizumab and 5.4% DXM + tocilizumab + HDMB. 37% presented at least one IAP, and the IAP incidence was higher with DXM + HDMB (66.4%) compared to no IS (P < 0.0001), DXM (P < 0.0001) and DXM + tocilizumab (P < 0.0001). HDMB and probabilistic antibiotherapy at admission were independent IAP predictors after adjustment on the propensity score (respectively OR: 2.44, P < 0.0001 and OR: 2.85, P < 0.001).

**Conclusion:** In critically ill COVID-19, HDMB significantly increases the risk of IAP whereas DXM alone, nor in combination with tocilizumab, did not.

**Compliance with ethics regulations:** Yes in clinical research.

## FC-167 Management of pregnant women admitted to the ICU for severe pneumonia related to SARS-Cov-2: experience of the referral maternities in Paris area (COVADIS-PREG study)

### Frédérique Schortgen^1^, Cecilia Tabrah^1^, Suela Demiri^1^, Cléo Dzogang^1^, Camille Jung^1^, Edouard Lecarpentier^1^, Study Group Covadis-Preg^1^

#### ^1^CHI Créteil, Créteil, France

##### **Correspondence:** Frédérique Schortgen (frederique.schortgen@chicreteil.fr)

*Annals of Intensive Care* 2023, **13(Suppl 1):**FC-0167

**Rationale:** Current evidence for respiratory and obstetric management of pregnant women with acute respiratory failure are lacking. The impact of invasive respiratory support on the best timing for delivery remains unknown. We report the management and outcomes of pregnant women with Sars-Cov-2 pneumonia admitted in the ICU.

**Patients and methods/materials and methods:** Retrospective cohort (Feb 2020–June 2021) including pregnant women admitted in the ICU of 15 referral maternities in Paris area for proven Sars-Cov-2 pneumonia requiring at least 6 L/min of standard O_2_. Patients were described according to the timing of delivery: early or delayed (> 2d) after ICU admission while not intubated, early or delayed (> 2d) after intubation. Risk factors associated with the need for intubation were assessed.

**Results:** 107 women 34 (30–38) years-old with ongoing pregnancy at a gestational age of 27 (25–30) weeks were included, 62 (58%) were obese, 34 (32%) had comorbidities. Median PaO_2_:FiO_2_ was 165 (130–208) mmHg at ICU admission. 94 (88%) received corticosteroids and 6 (6%) tocilizumab. Co-infection was suspected or proven in 33 (31%) women. 87 patients had CT-scan, 27 showing an extent > 50% and none had pulmonary embolism. Unplanned intubation was required in 47 (44%) patients: 35 with ongoing pregnancy, 19 per- or immediate post-partum. Intubation rate was 14/19 (74%) patients managed with standard O_2_, 17/36 (47%) managed with non-invasive ventilation (NIV) ± high flow nasal oxygen (HFNO), 16/52 (31%) managed with HFNO alone. ECMO was required in 10 (9%), 1 before and 9 after delivery. Factors associated with intubation were pulmonary co-infection OR: 2.88 (95% CI 1.04–7.99), HFNO: 0.11 (0.02–0.51) and NIV ± HFNO: 0.21 (0.04–0.97). 46 women were delivered during ICU stay with a median term birth of 30 (28–36) weeks, 85% for maternal worsening, 91% by caesarean route. 19 women were delivered early after ICU admission while not intubated (3 extremely and 3 very preterm), 24 were delivered early after intubation (7 extremely and 17 very preterm). Pregnancy was prolonged more than 2d after intubation in 11 patients, 3 were delivered in the ICU (1 stillbirth, 1 < 28w, 1 < 32w) and 8 after ICU discharge (1 < 28, 1 < 32w). Neonatal ICU admission was required in 29/46 (63%). One maternal death and one still birth were recorded.

**Conclusion:** The risk of intubation was reduced in pregnant women managed in first line with HFNO or NIV and increased in the presence of pulmonary co-infection. Pregnancy was prolonged under invasive mechanical ventilation in one third of patients.

**Compliance with ethics regulations:** Yes in clinical research.

## FC-168 Management of spontaneous pneumothorax post COVID 19 in the thoracic surgery department of the hospital center of Oran, Algeria

### Amel Zerhouni^1^, Soulef Bousbia^1^, Kheira Daho^1^, Mohamed Amine Benhamed^1^

#### ^1^CHUO, Oran, Algérie

##### **Correspondence:** Amel Zerhouni (azerhouni2000@yahoo.fr)

*Annals of Intensive Care* 2023, **13(Suppl 1):**FC-0168

**Rationale:** Introduction: Spontaneous pneumothorax (SP) is characterized by the leakage of bronchoalveolar air into the pleural space without prior trauma, which requires rapid diagnosis and treatment. It is a complication observed after a pneumonia due to COVID-19. The objective of the study was to describe the cases of SP during pneumonia due to COVID-19 for a period of 3 months (1/12/2021 to 1/02/2022) in the thoracic surgery department.

**Patients and methods/materials and methods:** Retrospective observational study of patients with spontaneous pneumothorax after pneumonia due to COVID-19, and their management.

**Results:** Twenty patients were included. The mean age was 50 years-old with a sex ratio of 1.3, 42% of the patients had no previous history, the remaining patient had diabete mellitus and hypertension. Tthe diagnosis of COVID-19 was made by the positive PCR test and CT-scan with a percentage of involvement between 10 and 75%. Partial pneumothorax appeared after 7 to 15 days of hospitalization. All patients required high-flow oxygen therapy, 14% non-invasive ventilation in addition to antibiotic therapy and corticosteroid therapy a (8 mg of Dexamethasone/day). 10% of patients had good outcomes and 70% of their pneumothorax became total requiring emergency thoracic drainage, 20% of which required intubation and mechanical ventilation. The duration of drainage was 5 to 21 days. The duration of hospitalization was from 12 to 48 days.

**Conclusion:** We must always keep in mind the risk of occurrence of a SP in all patients with COVID-19 even after the acute phase, it is necessary to investigate the mechanism of formation and especially the risk factors of its occurrence.

**Compliance with ethics regulations:** Yes in clinical research.

## FC-169 Barotrauma in critically-ill COVID-19 patients with acute respiratory distress syndrome

### Hedia Ben Ahmed^1^, Manel Lahmar^1^, Abir Chihaoui^1^, Hamza Ben Hassine^1^, Oussama Saadaoui^1^, Iyed Maatouk^1^, Zeineb Hammouda^1^, Wiem Nouira^1^, Saousen Ben Abdallah^1^, Fahmi Dachraoui^1^, Fekri Abroug^1^, Lamia Ouanes Besbes^1^

#### ^1^Hôpital fattouma bourguiba, Monastir, Tunisie

##### **Correspondence:** Manel Lahmar (firassmal4@gmail.com)

*Annals of Intensive Care* 2023, **13(Suppl 1):**FC-0169

**Rationale:** Barotrauma is rare in patients with acute respiratory distress syndrome undergoing mechanical ventilation. Its incidence seems to have increased among critically-ill COVID-19 patients in both intubated and non-intubated patients. The aim of this study was to evaluate the incidence, risk factors, clinical characteristics and radiological aspects of barotrauma among COVID-19 patients with ARDS receiving invasive and non-invasive ventilation.

**Patients and methods/materials and methods:** This observational study was conducted between March 2020 and September 2022 including consecutive patients hospitalized to the ICU for C-ARDS, and who have developed spontaneous pulmonary barotrauma.

**Results:** During the study period, 352 COVID-19 patients with ARDS were admitted to our ICU. Forty-four patients (50% male) have developed spontaneous pulmonary barotrauma and the overall incidence was 12.5%. The median age was 63 years [IQR: 52–70], the median SAPS II score was 27 points [IQR: 20–36] and the median P/F ratio was 93 mmHg [IQR: 74, 5–149]. 16% of patients had suffered from pre-existing lung parenchyma pathology (6 cases of pulmonary emphysema and one of fibrosis). Various associated co-morbidities were hypertension (40.9%), diabete mellitus (36.4%), and obesity (31.8%). Sixteen patients (36.4%) had subcutaneous emphysema, 14 (31.8%) had pneumothorax, 8 pneumomediastinum (18.2%), and 6 patients (13.6%) had association of subcutaneous emphysema and pneumothorax or pneumomediastinum, pneumoperitoneum and pneumomediastinum. The diagnosis of barotrauma was made clinically in 7cases, via chest X-ray in 28 cases and via CT scan in 9 cases. Sixteen patients (36.4%) breathing spontaneously under HFNC or NIV developed barotrauma, 28 patients (63.6%) during invasive ventilation with median PEEP of 8 cmH_2_O [IQR: 6–10]. The median time to occurrence of barotrauma after admission was 7 days [IQR: 3–13]. In multivariate analysis, the only predictor factor of barotrauma occurrence was PEEP (OR = 2, 48; 95% IC [1.61–3, 83]. Mortality was 84% in patients who developed barotrauma under invasive ventilation and those who required it after barotrauma.

**Conclusion:** Barotrauma is a frequent complication in critically ill COVID-19 patients and is associated with a poor prognosis. Since lung protective ventilation was delivered, the ventilatory management might not be the sole factor in the development of barotrauma.

**Compliance with ethics regulations:** Yes in clinical research.

## FC-170 Barotrauma in COVID-19 patients undergoing invasive mechanical ventilation: incidence, risk factors and impact on mortality

### Hajer Nouira^1^, Rihab Rajah^1^, Soumaya Chtioui^1^, Oussama Jaoued^1^, Mohamed Fekih Hassen^1^, Habiba Ben Sik Ali^1^, Souheil Elatrous^1^

#### ^1^EPS Taher Sfar, Mahdia, Tunisie

##### **Correspondence:** Oussama Jaoued (oussamajaoued@gmail.com)

*Annals of Intensive Care* 2023, **13(Suppl 1):**FC-0170

**Rationale:** Covid-19 was responsible for an increase in the use of invasive mechanical ventilation (IMV) in intensive care units (ICU). The incidence of barotrauma (a serious complication of IMV) in patients with Covid-19 pneumonia varies according to studies. The objectives of the study were to determine the incidence of barotrauma in Covid-19 patients admitted to the ICU, the risk factors for this complication and their impact on morbidity and mortality.

**Patients and methods/materials and methods:** This was a prospective, observational study conducted in the ICU from September 2020 to October 2021. We included patients with Covid-19 pneumonia requiring an IMV for more than 24 h. Barotrauma is defined by the occurrence of pneumothorax, pneumomediastinum, subcutaneous emphysema or pneumopericardium.

**Results:** A total of 137 patients with a median age of 59 ± 13 years and a median APACHE II score of 12 ± 5 points were included. The incidence of barotrauma was 24%. Barotrauma occurred 8 days (IQR: 4–13) after IMV initiation. Median tidal volume (Vt) at initiation of IMV was higher in the barotrauma group (7 ml/kg IQR (6.2–7) versus 6 ml/kg IQR (6–6.5), p < 10^−3^). Peak pressure was higher in the barotrauma group during the 14 days following initiation of IMV. IMV duration was higher in the barotrauma group [16 days (IQR: 11–23) versus 9 days (IQR: 5–17) days, p < 10–3], as well as the length of stay in ICU (22 days, (IQR: 14–28) versus 13 (IQR: 9–21), p < 10^−3^). There was no difference in the mortality rate between the two groups. In the multivariate analysis, factors associated with the occurrence of barotrauma were Vt (OR = 10.32; 95% CI (2.56–41.52), p = 0.001), PaO_2_/FiO_2_ ratio (OR 0.96; 95% CI 0.94–0.98; p = 0.001) and peak pressure at day 7 of IMV (OR = 1.28; 95% CI 1.13–1.46; p < 10^−3^).

**Conclusion:** Barotrauma is a frequent complication in patients with COVID-19 pneumonia that requires IMV. Factors associated with the occurrence of this complication were Vt, PaO_2_/FiO_2_ ratio and peak pressure on day 7 of IMV. Mortality was similar in both groups. Patients with barotrauma had a longer duration of IMV and longer length of stay in the ICU.

**Compliance with ethics regulations:** Yes in clinical research.

## FC-171 High emergency lung transplant in anti-MDA5 dermatomyositis patients with rapidly progressive interstitial lung disease

### Mathilde Neuville^1^, Antoine Roux^1^, Benjamin Zuber^1^, Yves Allenbach^2^, Morgan Le Guen^1^, Jérôme Devaquet^1^, Matthieu Glorion^1^, Charles Cerf^1^

#### ^1^Hôpital Foch, Suresnes, France; ^2^Pitié-Salpêtrière, Paris, France

##### **Correspondence:** Mathilde Neuville (m.neuville@Hôpital-foch.com)

*Annals of Intensive Care* 2023, **13(Suppl 1):**FC-0171

**Rationale:** Rapidly progressive interstitial lung disease (RP-ILD) associated with anti-Melanoma Differentiation-Associated Gene 5 dermatomyositis (anti-MDA5 DM) is a rare but life-threatening condition despite aggressive immunosuppressive treatment. When patients are intubated and not eligible for LT, mortality in Intensive Care Unit (ICU) is close to 100% (1). The prognosis of this disease after lung transplant (LT) in a High Emergency process is not well known.

**Patients and methods/materials and methods:** We retrospectively analysed data from 7 adults who underwent LT after a High Priority registration on the National Waiting List in Hôpital Foch, Suresnes, France, between 2018 and 2022.

**Results:** Patients were 50 ± 7 years old. The delay between first symptoms and diagnosis was 72 [22–137] days, and 83 [20–123] days between first signs and ICU admission. All patients presented with RP-ILD, mostly with skin involvement, but none of them suffered from symptomatic muscular, cardiac, or swallowing impairment. SOFA score at ICU admission was 2, reflecting an isolated respiratory failure. All patients worsened under High Flow Nasal Oxygen and needed to be placed under mechanical ventilation 6.7 [1–19] days after ICU admission. Five of the 7 patients needed veno-venous extracorporeal membrane oxygenation (VV-ECMO) before LT, for a total duration of 16 [1–28] days. LT was performed 3 ± 3.7 days after registration on the National Waiting List with High Priority; SOFA score at LT was 7 ± 2.4. The Lung Allocation Score was 31. Even under ECMO, all patients over 49 years of age had a diagnostic coronarography and a PET-scan to check for the absence of cancer; only one of them showed a muscle involvement. After LT, one patient died at day 35 of respiratory failure due to refractory acute humoral rejection. For the six others, ventilation was weaned after 32 [2–126] days. ICU and hospital stay were respectively 47 [18–163] and 80.5 [70–218] days. Patients showed no sign of relapse of the anti-MDA5 DM after a median following time of 17 [1–43] months.

**Discussion:** Our data confirm the good results of LT in ILD associated with amyositic idiopathic inflammatory myositis even in a High Emergency process, when patients were previously unknown by LT centers (2).

**Conclusion:** LT can be a life-saving procedure in anti-MDA5 DM RPILD. High emergency allocation priority on transplant list may shorten the time between ICU admission and surgery. Since 2021, a French national Multidisciplinary Consortium meeting composed of pulmonologists, intensivists, specialists in internal medicine, rheumatologists and lung transplant experts can be set in emergency, when LT needs to discuss for a MDA5 patient.

**Reference 1**

Bay P, Pineton de Chambrun M, Roux A, et al. Extracorporeal life support allows lung transplant in anti-MDA5 + rapidly progressive interstitial lung disease. Eur Respir J 2022;59(5):2102968.

**Reference 2**

Rivière A, Picard C, Berastegui C, et al. Lung transplantation for interstitial lung disease in idiopathic inflammatory myositis: a cohort study. American Journal of Transplantation 2022;22(12):2990–3001.

**Compliance with ethics regulations:** Yes in clinical research.

## FC-172 Intensive care readmissions of lung transplant recipients: etiologies, prognosis, management

### Marc Domant^1^, Jonathan Messika^2^, Alexandre Vallée^2^, Etienne De Montmollin^1^, Enora Atchade^1^, Alexy Tran Dinh^1^, Antoine Roux^2^, Hervé Mal^1^, Edouard Sage^2^, Pierre Mordant^1^, Philippe Montravers^1^, Morgan Le Guen^2^, Charles Cerf^2^, Benjamin Zuber^2^

#### ^1^Hôpital Bichat, Paris, France; ^2^Hôpital Foch, Suresnes, France

##### **Correspondence:** Benjamin Zuber (b.zuber@Hôpital-foch.com)

*Annals of Intensive Care* 2023, **13(Suppl 1):**FC-0172

**Rationale:** The course of lung transplant recipients (LTR) might be complicated by intensive care unit (ICU) admission. We aimed at performing a reappraisal of etiologies and prognosis of acute respiratory failure (ARF) leading to ICU readmission in LTR after the 1st month of the initial ICU stay.

**Patients and methods/materials and methods:** Bicenter “real-life” retrospective study of the first ICU readmission (Jan 2012–Dec 2018) for an ARF of all LTR. The main endpoint was in-hospital mortality. Univariate analysis by center cluster and multivariate Cox model analysis were performed. Data are expressed in med [IQR 25–75] or n (%).

**Results:** One hundred and twenty-three LTR with an ICU readmission for ARF were included. Seventy were male (56.9%), aged 55 yo [44–61], with a bilateral LT (n = 82, 66.7%), 11.1 months [3.6–24.3] before the ARF episode. Eighty-seven patients were discharged alive from hospital (70.7%). No statistical difference was evidenced in demographic, anamnestic, ongoing treatments and previous complications according to hospital survival. Non-survivors had higher severity score at ICU admission (SAPSII 37.5 [26–52] vs 29 [24–37]; p < 0.001 and SOFA score 3 [2–7.2] vs 3 [2–4]; p < 0.001), more invasive mechanical ventilation (77.8 vs 36.8%; p < 0.001), need for vasopressors (68.6 vs 21.8%; p < 0.001) and renal replacement therapy (36.1 vs 6.9%; p < 0.001), compared with survivors. Pneumonia was the main cause of ARF in 73 LTR (59.4%) and associated to another cause in 23 cases (31.5%). Acute graft rejection caused ARF in 19 LTR (15.5%), either as a definite diagnosis (n = 7; 36.8%) or being “possible” or “probable” in other cases. Rejection was associated to another etiology in 11 cases (57.9%). In multivariate analysis, acute graft rejection and need for vasopressors predicted hospital mortality (resp. OR 9.41 [1.76–10.28] and 17.31 [4.53–26.16]), while an increase in forced vital capacity at last evaluation was associated with lower odds of death (OR, by unit 0.93 [0.90–0.97]).

**Conclusion:** In this bicenter retrospective cohort of LTR readmitted in the ICU for an ARF, a third did not survive hospital stay. The most frequent cause for ARF was infection. Acute rejection was seldomly documented, frequently associated with another cause, holded a poor prognosis, and predicted hospital death.

**Compliance with ethics regulations:** Yes in clinical research.

## FC-173 Impact of oxygenation strategies on outcome in acute exacerbation of interstitial lung disease: a multicenter retrospective study

### Robin Thévenin^1^, André Gillibert^5^, Nicolas Dognon^2^, Mélanie Drucbert^4^, Laurie Lagache^3^, Mathieu Salaun^1^, Antoine Cuvelier^1,6^, Christophe Girault^6,7^, Elise Artaud-Macari^1,6^

#### ^1^Pulmonary, Thoracic Oncology and Respiratory Intensive Care Unit, CHU Rouen, Rouen, France; ^2^Department of Intensive Care Medicine, Critical Care Centre, CHU Lille, Lille, France; ^3^Department of Intensive Care Medicine, Le Havre Hospital, Montvilliers, France; ^4^Department of Respiratory Diseases, Amiens University Hospital, Amiens, France; ^5^Department of Biostatistics, CHU Rouen, Rouen, France; ^6^Normandy University, UNIROUEN, UR 3830, Rouen University Hospital, Rouen, France; ^7^Medical Intensive Care Unit, CHU Rouen, Rouen, France

##### **Correspondence:** Elise Artaud-Macari (eliseartaudmacari@yahoo.fr)

*Annals of Intensive Care* 2023, **13(Suppl 1):**FC-0173

**Rationale:** The benefits of non-invasive ventilation (NIV) in hypoxemic ARF are controversial and have never been studied in acute exacerbation of interstitial lung disease (AE-ILD). The potential protective effects of high-flow nasal cannula oxygen therapy (HFNC) deserve to be evaluated in this condition. This study aimed to compare outcomes of adult patients with AE-ILD admitted in respiratory intensive care unit (ICU) according to oxygenation strategies that were used within 48 h of admission: NIV ± HFNC or HFNC alone.

**Patients and methods/materials and methods:** We conducted a multicenter retrospective study in four French tertiary hospitals between 01/01/2010 and 12/31/2021 with in-hospital mortality as primary outcome. A propensity score was used to adjust on age, Activities of Daily Living (ADL), long-term oxygen therapy, body mass index (BMI), corticosteroids/immunosuppressors in the first 48 h.

**Results:** Among 3962 screened patients, 163 were included (mean ± SD age: 70.2 ± 10.3, 51 [31.3%] women): 118 with HFNC and 45 with NIV ± HFNC. The propensity score adjusted in-hospital mortality rate was 55.2% (95% CI 36.0 to 74.4%) in the NIV ± HFNC group vs 45.9% (95% CI 36.5 to 55.4%) in the HFNC group with a difference estimated at − 9.3% (95% CI − 30.5 to + 12.0%, p = 0.39). The adjusted intubation rates were 36.9% and 25.9% in the HFNC and NIV ± HFNC groups, respectively, with a difference at − 11.3% (95% CI − 30.7 to + 8.1%, p = 0.25).

**Conclusion:** Management of AE-ILD has progressively included HFNC use during the past 10 years, despite the lack of evidence in the literature. Our study did not find any superiority of NIV ± HFNC vs HFNC alone in AE-ILD outcome but deserves further research with more patients.

**Compliance with ethics regulations:** Yes in clinical research.

## FC-174 Pulmonary embolism severity in ICU: correlation of computed tomography with echocardiography and clinical presentation: preliminary results

### Asma Mehdi^1^, Ahlem Trifi^1^, Hounaida Galaii^1^, Bedis Tlili^1^, Eya Seghir^1^, Linda Masseoud^1^, Salma Rabhi^1^, Sami Abdellatif^1^

#### ^1^Hospital la Rabta, Tunis, Tunisie

##### **Correspondence:** Asma Mehdi (asmaelmahdi245@gmail.com)

*Annals of Intensive Care* 2023, **13(Suppl 1):**FC-0174

**Rationale:** Pulmonary embolism (PE) is a frequent cause of hospitalization in ICU, with a possible requirement of mechanical ventilation and vaso-active agents. Positive diagnosis is based on chest computed tomography (CT). Risk stratification is based according to ESC guidelines on hemodynamic tolerance, PESI score, biomarkers disorders and right ventricular (RV) dysfunction. Four groups are individualized: high, intermediate (high/low) and low risk of mortality. The aim of our study was to assess wheter there is a parallelism between scannographic severity of PE and hemodynamic status, biological disorders and RV dysfunction.

**Patients and methods/materials and methods:** A prospective observational study in ICU adult patients who presented a PE on chest CT scan. Delay between chest CT scan diagnosis and clinical, biological and echocardiographic evaluation was limited to 72 h. RV dysfunction was defined as a dilatation of right cavities, SPAP > 40 mmHg, TAPSE < 15 mm or paradoxal septum.

**Results:** Thirteen patients were enrolled. Mean age was 60 ± 18.5. Most patients had no medical history: 3/13 had hypertension, 2/13 had diabetes mellitus, 2/13 had chronic respiratory disease and 1 patient had chronic cardiac disease. PE was diagnosed in 8/13 cases at admission. When diagnosed during hospital stay, mean delay was 8 days ± 8.1. PE was considered as severe according to chest CT scan in 7 cases (54%). All these patients had not present obstructive shock during hospital stay.RV dysfunction on echocardiography was found in only two cases without biomarkers disorders: mean troponin US level was 22.2 ± 13.5. No patient was classified at high risk of mortality according to ESC classification. Thrombolysis was not required with favorable outcome.

**Conclusion:** Chest CT scan is the reference exam to establish the diagnosis of PE. When this latter was considered as severe on CT scan, it was not after clinical and echocardiographic evaluation. We suggest reconsidering the prognosis value of this exam and the need of ICU admission for all severe PE according to scannographic criteria.

**Compliance with ethics regulations:** Yes in clinical research.

## FC-175 Non-ventilated ICU-acquired pneumonia and acute exacerbations of COPD in French ICUs

### Louis-Marie Galerneau^1^, Sébastien Bailly^1^, Nicolas Terzi^2^, Stéphane Ruckly^3^, Maité Garrouste-Orgeas^4^, Yves Cohen^5^, Vivien Hong Tuan Ha^6^, Marc Gainnier^7^, Shidasp Siami^8^, Claire Dupuis^9^, Michael Darmon^10^, Jean-Marie Forel^11^, Anaïs Dartevel^1^, Christophe Adrie^12^, Dany Goldgran-Toledano^13^, Virginie Laurent^14^, Etienne De Montmollin^15^, Laurent Argaud^16^, Jean Reignier^17^, Jean-Louis Pepin^1^, Jean-François Timsit^15^

#### ^1^University Hospital Grenoble Alpes, Grenoble, France; ^2^Rennes University Hospital, Rennes, France; ^3^Outcomerea, Paris, France; ^4^French and British Institute, Levallois-Perret, France; ^5^Avicenne Hospital, Paris, France; ^6^Meaux Hospital, Meaux, France; ^7^La Timone Hospital, Marseille, France; ^8^Etampes-Dourdan Hospital, Etampes, France; ^9^Gabriel Montpied University Hospital, Clermont-Ferrand, France; ^10^Saint-Louis Hospital, Paris, France; ^11^Nord University Hospital, Marseille, France; ^12^Delafontaine Hospital, Saint-Denis, France; ^13^Le Raincy-Montfermeil Hospital, Montfermeil, France; ^14^André Mignot Hospital, Le Chesnay, France; ^15^Bichat Hospital, Paris, France; ^16^Edouard Herriot Hospital, Lyon, France; ^17^Nantes University Hospital, Nantes, France

##### **Correspondence:** Louis-Marie Galerneau (lmgalerneau@chu-grenoble.fr)

*Annals of Intensive Care* 2023, **13(Suppl 1):**FC-0175

**Rationale:** The non-ventilated Intensive Care Unit acquired pneumonia (NV-ICU-AP), nosocomial pneumonia not associated with invasive ventilation, has been much less studied than ventilator-associated pneumonia, and never in the context of patients in ICU for a severe acute exacerbation of chronic obstructive pulmonary diseases (AECOPD), a common cause of ICU admission. We assessed the impact of NV-ICU-AP on the outcomes of these patients.

**Patients and methods/materials and methods:** Data were prospectively collected in the French ICU database OutcomeReaTM. By using survival analyses with competing risk management, we assessed the impact of NV-ICU-AP on mortality, intubation and length of stay in ICU.

**Results:** On 844 COPD exacerbations managed in ICU without IMV, the NV-ICU-AP occurred in 42 patients, corresponding to an incidence density of 10.8 per 1000 days of exposition. NV-ICU-AP occurred after a median delay after admission of 6 [4.0; 11.0] days and 32 patients with NV-ICU-AP (76.2%) were intubated within 48 h after the NV-ICU-AP diagnosis. After adjustment for confounders, NV-ICU-APs were associated with increased 28-day mortality (HR = 3.08 [1.39; 6.82]).

**Conclusion:** We found that NV-ICU-APs were associated with an increased risk of 28-day mortality, intubation and longer stay for patients referred in ICU for AECOPDs. Progress is needed on the diagnosis and prevention of NV-ICU-PA, particularly for fragile patients with chronic respiratory disease such as COPD.

**Compliance with ethics regulations:** Yes in clinical research.

## FC-176 Respiratory tract infections triggering acute severe COPD exacerbation in critically ill patients: an observational cohort study

### Georges Abi Abdallah^1^, Sylvain Diop^2^, Matthieu Jamme^3^, Stéphane Legriel^1^, Alexis Ferré^1^

#### ^1^Centre Hospitalier André Mignot, Le Chesnay, France; ^2^Hôpital Marie Lannelongue, Le Plessis Robinson, France; ^3^Hôpital Privé de l'Ouest Parisien, Trappes, France

##### **Correspondence:** Georges Abi Abdallah (georges.abiabdallah@aphp.fr)

*Annals of Intensive Care* 2023, **13(Suppl 1):**FC-0176

**Rationale:** Respiratory infections are responsible for two-thirds of acute exacerbation in patients with chronic obstructive pulmonary disease (COPD). However, data are scarce on their predictive factors and prognosis in critically ill patients. We aimed to investigate respiratory infection patterns at intensive care unit (ICU) admission during severe COPD exacerbation and to identify variables associated with the type of infection, the need for invasive mechanical ventilation (MV) and in-hospital mortality.

**Patients and methods/materials and methods:** Patients admitted for a severe acute exacerbation of COPD in a French ICU between January 2015 and December 2021 were retrospectively included. Logistic multivariate regression analysis was performed to predict factors associated with the infection and to assess the association between infection type and outcomes.

**Results:** We included 473 patients. Among them, 288 (60.9%) had respiratory infection as triggering factor. Eighty-nine infections (30.9%), 81 (28.1%) and 34 (11.8%) were respectively viral, bacterial and mixed infections as opposed to not documented infection in 84 cases (29.2%). Among the global cohort, 139 (29.4%) patients required invasive MV. Forty-seven (9.9%) patients died in ICU and 67 (14.2%) died in hospital. Factors independently associated with respiratory infection were temperature and neutrophil polynuclear blood count (OR(+1 °C) = 1.43, 95% CI [1.16–1.78], P = 0.008; and OR = 1.07, 95% CI [1.02–1.11], P = 0.002; respectively). Male sex and neutrophil polynuclear blood count were associated with bacterial infection (OR = 2.21, 95% CI [1.14–4.41], P = 0.02; and OR = 1.06, 95% CI [1.01–1.13], P = 0.04; respectively). In multivariate analysis, pneumonia, initial PaCO_2_ and lactate were associated with the need for invasive MV (OR = 1.75, 95% CI [1.18–2.60], P = 0.005; OR = 1.08, 95% CI [1.01–1.17], P = 0.04; OR = 1.14, 95% CI [1.03–1.28], P = 0.02; respectively). In multivariate analysis, age, immunosuppression and altered performance status were associated with in-hospital mortality (OR = 1.03, 95% CI [1.01–1.06], P = 0.03; OR = 1.96, 95% CI [1.08–3.55], P = 0.02; OR = 1.78, 95% CI [1.23–2.57], P = 0.002; respectively) whereas respiratory infection as triggering factor, pneumonia or bacterial infection were not.

**Conclusion:** Respiratory tract infections are the main cause of severe acute exacerbation of COPD in ICU and bacteria account for 43% of cases. Early bacterial identification is crucial to adapt antibiotic therapy. Only body temperature and neutrophil polynuclear blood count are predictive factors of bacterial infection. Pneumonia is associated with the need for invasive MV but not with hospital mortality as opposed to age, immunosuppression and altered performance status. Prospective larger studies are needed to explore this further.

**Compliance with ethics regulations:** Yes in clinical research.

## FC-177 Impact of beta-blockers on medium term prognosis of severe acute exacerbation of chronic obstructive pulmonary disease (AECOPD) patients admitted to ICU

### Iyed Maatouk^1^, Abir Chihaoui^1^, Asma Zarrouk^1^, Rania Lahouimel^1^, Zeineb Hammouda^1^, Oussema Saadaoui^1^, Wiem Nouira^1^, Manel Lahmar^1^, Saoussen Ben Abdallah^1^, Fahmi Dachraoui^1^, Fekri Abroug^1^, Lamia Ouanes Besbes^1^

#### ^1^University Hospital Fattouma Bourguiba of Monastir, Monastir, Tunisie

##### **Correspondence:** Iyed Matouk (maatouk.yed@gmail.com)

*Annals of Intensive Care* 2023, **13(Suppl 1):**FC-0177

**Rationale:** Patients with chronic obstructive pulmonary disease (COPD) are more likely to present cardiovascular diseases which are commonly treated with Beta-blockers (BB). Patients with chronic obstructive pulmonary disease (COPD) are more likely to present cardiovascular diseases which are commonly treated with Beta-blockers (BB). The objective of our study was to evaluate the impact of BB on the prognosis of AECOPD outcomes.

**Patients and methods/materials and methods:** We conducted a single-centre retrospective study in a 16-bed ICU of a Tunisian University Hospital from January 2016 to June 2019. All patients admitted for an AECOPD and aged ≥ 18 years were included. Follow-up data included mortality at 6 months of ICU discharge and life quality assessment using the Clinical COPD Questionnaire (CCQ).

**Results:** A total of 220 patients were included. The median age was 68 [61–75]. The majority were male (88.2%). Median SAPS II was 45 [40–53]. NIV was the first line ventilatory treatment in 88.2% with a failure rate of 26.8%. Median Length of stay was 16 [10–27] days with a survival rate of 80.5%. In our study, 62 patients received BB (28.2%) (BB+ group). Bisoprolol was the most frequently used molecule (96.8%) with a median posology of 2.5 [2.5–5] mg. Almost one quarter of patients previously received BB (24.2%). Indications for BB upon admission were hypertension (62.5%), tachycardia (9.7%) and left heart failure (3.2%). The two groups presented no major differences at baseline concerning severity assessment, ventilatory management or NIV failure. Six month-mortality was significantly higher in BB- group (34.2 versus 17.7%; p = 0.016) (Figure1) and risk of recurrence of AECOPD at 1 year was lower in BB+ group (43.5% versus 58.2%; p = 0.04). No significant difference in CCQ assessment was found (p = 0.2).

**Conclusion:** Patients on beta-blockers have better prognosis after AECOPD with lower mortality and risk of recurrence of AECOPD. Further studies should be conducted to evaluate the impact of other drugs on COPD patients' outcomes to improve quality of life.

**Compliance with ethics regulations:** Yes in clinical research.

**Figure 1 (abstract FC-177):**
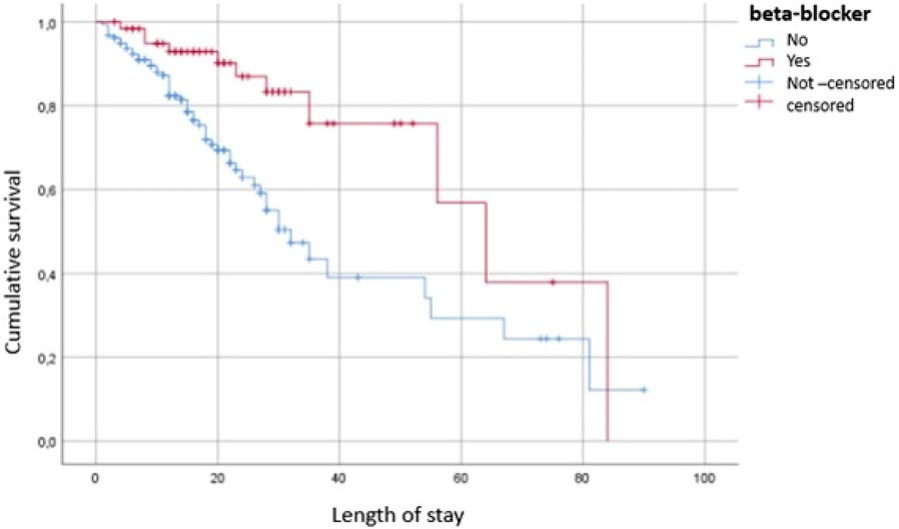
Kaplan–Meier curve of 6-month survival of patients according to beta-blocker use

## FC-178 Predictors of invasive ventilation requirement in acute hypercapnic respiratory failure: a retrospective clinical study

### Sourour Bel Haj Youssef^1^, Rihab Boubtane^1^, Dhouha Ben Braiek^1^, Azer Yaacoub^1^, Nadine Boukadida^1^, Yosri Ben Ali^1^, Hend Zorgati^1^, Arij Hammedi^1^, Rahma Ben Jazia^2^, Amani Kacem^2^, Jihene Ayachi^1^

#### ^1^Medical Intensive Care Unit, Ibn El Jazzar University Hospital, Kairouan, Tunisie; ^2^Pulmonology Department, Ibn El Jazzar University Hospital, Kairouan, Tunisie

##### **Correspondence:** Jihene Ayachi (ayachijihen@gmail.com)

*Annals of Intensive Care* 2023, **13(Suppl 1):**FC-0178

**Rationale:** Noninvasive ventilation (NIV) has been reported to be the most important treatment for patients with acute hypercapnic respiratory failure (AHRF) in intensive care units (ICU). However, the NIV failure rate remains high, which will delay intubation and increase hospital mortality. Therefore, it is important to identify patients at high risk of intubation. This study aimed to describe the epidemiologic and clinical characteristics of patients admitted for AHRF, the treatment and the evolution in ICU in order to deduce the factors predicting invasive ventilation (IV).

**Patients and methods/materials and methods:** A retrospective analytical study, carried out in a 9-bed medical ICU from January 2021 to January 2023, which included patients admitted consecutively for AHRF, with pH < 7.35, and PaCO_2_ > 45 mmHg and requiring the use of NIV on admission. The primary endpoint was IV requirement.

**Results:** Eighty-eight patients were admitted for AHRF. Among them, 75 (85.2%) received NIV as the first ventilatory support. Mean age was 64 ± 11 and male female ratio was 4.7. Sixty-two of patients (82.7%) were COPD, 5 (6.7%) were SOH and bronchial dilation was found in 10.7% of cases. The most common comorbidities were high blood pressure (34.2%) and diabetes (23.7%). Sixty (80%) of patients were smokers. History of ICU hospitalization was found in 12% of all cases. Ten (13%) patients were ventilated at home. Mean SAPS II, median of APACHE II and Charlson index were 26 ± 9.3, 10 [7–15] and 3 [3–4], respectively. Mean GCS was 14 ± 2. Mean pH and PaCO_2_ measured at 1–2 h (h) of NIV were 7.32 ± 0.08 and 62.3 ± 17, respectively. The most common diagnosis was tracheobronchitis (76%). Pneumonia was founding 13.3% of cases. Sixteen (21.3%) patients experienced NIV failure, all of them were intubated. The mean duration of NIV use was 5 ± 3.5 days. APACHE II (p = 0.032), SAPS II (p = 0.004), heart rate (p = 0.002), GCS (p = 0.008), pH at 1–2 h (p = 0.031) and 24 h (p = 0.023), PaCO_2_ at 1–2 h (p = 0.015) and 24 h (p = 0.018), pneumonia on chest CT (p = 0.04) were all predictive of IV requirement in univariate analysis, but only APACHE II (OR 1.33; 95% CI, [1.002–1.789]; p = 0.048) and pneumonia (OR 60.34; 95% CI, [1.659–2194.7]; p = 0.025) were significant in multivariate analysis.

**Conclusion:** APACHE II and pneumonia are associated with IV requirement in patients with AHRF. A predictive model based on the risk factors could help to identify patients at high risk of intubation.

**Compliance with ethics regulations:** Yes in clinical research.

## FC-179 Association between oxygenation parameters and acute kidney injury in critically ill COVID-19 patients

### Amir Bedhiafi^1^, Ahlem Trifi^2^, Wafa Ammous^3^, Asma Mehdi^2^, Eya Seghir^2^, Lynda Messaoud^2^, Badis Tlili^2^, Adel Ammous^3^, Sami Abdellatif^2^

#### ^1^Medical ICU, la Rabta hospital, Tunis, Tunisie; ^2^Medical ICU, la Rabta hospital, Faculty of Medicine of Tunis, Tunis, Tunisie; ^3^Department of Anesthesia, University Hospital Center La Rabta, Tunis, Tunisie

##### **Correspondence:** Amir Bedhiafi (bedhiafi.emir@gmail.com)

*Annals of Intensive Care* 2023, **13(Suppl 1):**FC-0179

**Rationale:** Acute kidney injury (AKI) is a common complication among critically ill COVID-19 patients. It is also correlated to higher rates of morbidity and mortality. Pathophysiological processes have been largely discussed, among them the severity of hypoxemia and ARDS. In this study, we aimed to assess the association between oxygenation parameters and AKI occurrence.

**Patients and methods/materials and methods:** This is a retrospective observational cohort study conducted in two ICUs of a Tunisian university hospital over 16 months, from September 2020 to December 2021. All consecutive patients with confirmed COVID-19 pneumonia admitted to the ICU were included. Demographics, baseline clinical, characteristics and oxygenation parameters and trends were recorded. Worsening PaO_2_/FiO_2_ was measured as the difference between its value at admission and its worst value throughout the evolution. Patient’s evolution, outcome, and complications of ICU course were collected. A univariate analysis was performed.

**Results:** During the study period, 309 patients were included. They were mainly males with a sex ratio of 1.7 and median aged 63 [55–70] years. Patients had a median BMI of 27.7 [24.2–33] kg/m^2^ and major comorbidities were hypertension 45% (n = 139) and diabetes 41.4% (n = 128). On admission, SAPS II at 24 [13–37], APACHE II [16-9-27], and SOFA score 4 [3–7]. On respiratory assessment, 87.7% fulfilled the ARDS criteria, predominantly moderate 31.7% to severe 43.3% ARDS with a median PaO_2_/FiO_2_ ratio of 102 [76–160]. On biology, median creatinine and uremia levels were respectively 8 [6.6–11] mg/L and 0.47 [0.3–0.75] g/L. Acute kidney injury occurred in 34.7% of patients, KDIGO grade-I 22.7%, grade-II 22.6%, and grade-III 54.7%. Regarding the relation with oxygenation parameters, a lower PaO_2_/FiO_2_ ratio on admission and a greater worsening of its value throughout the ICU course were associated with the occurrence of AKI (p = 0.022 and p = 0.013, respectively). However, no correlation was found between the severity of hypoxemia and creatinine levels.

**Conclusion:** Our findings suggest that compromised oxygenation parameters are associated to the onset of AKI. Thus, early intervention to improve oxygenation might improve renal outcomes.

**Compliance with ethics regulations:** Yes in clinical research.

**Figure 1 (abstract FC-179):**
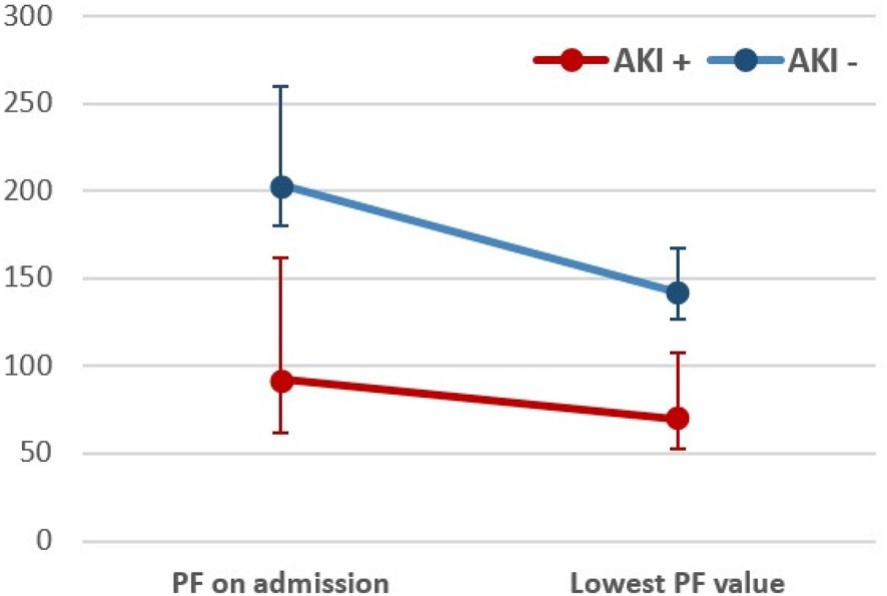
Compared PaO_2_/FiO_2_ ratio between the two groups

## FC-180 Acute kidney injury induced by COVID-19 in critical patients: incidence, risk factors and outcome

### Ahlem Trifi^1^, Wafa Ammous^1^, Asma Mehdi^1^, Yosri Masseoudi^2^, Sami Abdellatif^1^, Adel Ammous^2^

#### ^1^Medical ICU, the teaching hospital of la Rabta, Tunis, Tunisie; ^2^Department of anesthesiology, the teaching hospital of la Rabta, Tunis, Tunisie

##### **Correspondence:** Ahlem Trifi (trifiahlem2@gmail.com)

*Annals of Intensive Care* 2023, **13(Suppl 1):**FC-0180

**Rationale:** During SARS-CoV-2 infection, the kidney is considered as a “target” organ and acute kidney injury (AKI) represents the most common organ dysfunction after respiratory failure. The aim of our work was to determine the frequency, risk factors and impact on the evolution of AKI in critical patients affected with COVID-19.

**Patients and methods/materials and methods:** Retrospective analytical study recruiting all patients with COVID-19 and hospitalized in intensive care. The definition and classification of AKI severity was based on the KDIGO classification. The biological data were collected over several times. Studies of frequencies, correlations, multivariate analysis and survival were carried out.

**Results:** 309 patients were included, among them 107 (34.6%) presented AKI within a median time = 5 [1–7] days. AKI was classified as KDIGO 1 (23.5%), KDIGO 2 (22.5%) and KDIGO 3 (54%) and hemodialysis was required in 11.2% of cases. Negative correlations were found between urea/creatinine and pH, bicarbonates and PT and positive correlations between urea and D dimers in addition to lactate and between creatinine and white blood cells and lymphocytes. COVID/AKI+ patients (n = 107) were older (67 vs 61 years, p < 10^−3^), composed more of men (72% vs 60%, p = 0.04), having more co- morbidities and had, on admission, higher values of urea (0.71 vs 0.4 g/L, p = 0.0001), creatinine (12 vs 7.17 mg/L, p < 10^−3^) and D dimers (1776 vs 1138 IU, p = 0.016) and lower values of P/F (92.5 vs 110, p = 0.028) and platelets (210,000 vs 257,000 elts/mm^3^, p = 0.024). Vasopressors (OR = 9.9 [2.53–38.7]), septic shock (OR = 2.57 [1.57–4.2]), P/F < 92 (OR = 1, 57 [1.08–2.53]) and a baseline creatinine > 10 mg/L (OR = 1.33 [1.16–152]) were associated with AKI. Survival analysis showed a decline in survival by 9 days in patients with AKI (p = 0.018).

**Conclusion:** AKI was a frequent complication during critical COVID. Hemodynamic and oxymetric precariousness and high baseline creatinine contributed to its occurrence. It reduced survival by 9 days. Prevention and the appropriate use of renal replacement techniques are the mainstays of the AKI management in this kind of patients.

**Compliance with ethics regulations:** Yes in clinical research.

## FC-181 Predictors of mortality in patients with COVID-19 infection- associated acute kidney injury

### Nabil Bouguezzi^1,2^, Imen Ben Saida^1,2^, Radhouane Toumi^1,2^, Rym Chelbi^1,2^, Rihab Rajah^1^, Azer Yaacoub^1,2^, Imen Belhouchet^1,2^, Zakaria Meftah^1^, Khaoula Meddeb^1,2^, Mohamed Boussarsar^1,2^

#### ^1^University of Sousse, Faculty of Medicine of Sousse, Sousse, Tunisie; ^2^Farhat Hached University Hospital, Medical Intensive Care Unit, Research Laboratory “Heart Failure”, LR12SP09, Sousse, Tunisie

##### **Correspondence:** Nabil Bouguezzi (dr_nabil@live.fr)

*Annals of Intensive Care* 2023, **13(Suppl 1):**FC-0181

**Rationale:** Coronavirus disease 2019 (COVID-19) is a respiratory illness caused by an emerged virus “severe acute respiratory syndrome coronavirus2 (SARS-CoV-2)”. An alarming number of COVID-19 patients especially severe cases developed Acute Kidney Injury (AKI). This serious complication may result in increased mortality, longer ICU stay and high medical cost. Data on predictors of mortality in critically ill COVID-19 patients with AKI are scarce. The aim of the present study is to determine the predictors for ICU (intensive care unit) mortality among patients with COVID-19 associated AKI in the ICU.

**Patients and methods/materials and methods:** This is an analytic retrospective study carried out in a medical intensive care unit from March 2020 to December 2021. All patients who developed AKI were included. Clinical characteristics and laboratory data were extracted from electronic hospitalization and laboratory databases. AKI was defined and staged according to the 2012 Kidney Disease: Improving Global Outcomes criteria. The association of AKI with ICU mortality was assessed. The patients were divided into two groups: survivors and non survivors. Univariate and multivariate analysis were used to identify factors associated with ICU mortality in AKI COVID-19 patients.

**Results:** AKI was diagnosed in 229 patients. Median age, 65 [55–71] years, male 137 (59.8%); median SAPSII, 33 [27–42]; IMV, 144 (62.9%). Forty-seven (20.5%) patients received renal replacement therapy, with a total of 117 sessions. A total of 135 (58.9%) patients with AKI died in hospital. In univariate analysis, age, comorbidities (hypertension, diabetes, obesity and chronic renal failure), CCI, APACHE II and SAPS II, stage 3 AKI, invasive mechanical ventilation, post intubation shock and septic shock were found to be associated (p < 0.05) with ICU mortality among those patients. Multivariate regression model identified the following factors as independently associated with mortality: age (OR, 1.05; 95% CI, [1.02–1.09]; p = 0.001), septic shock (OR, 3.65; 95% CI, [1.32–10.10]; p = 0.012), IMV (OR, 48.23; 95% CI, [18.05–128.89]; p < 0.001).

**Conclusion:** In critically ill patients with COVID-19 pneumonia, who developed AKI, age, septic shock, and IMV use were determined as predictors of mortality.

**Compliance with ethics regulations:** Yes in clinical research.

## FC-182 Factors associated with acute kidney injury among crtically ill COVID-19 patients

### Iyed Maatouk^1^, Rania Lahouimel^1^, Maha Hamdi^1^, Saoussen Benabdallah^1^, Hamza Ben Hssine^1^, Dorra Berkhaies^1^, Hanene Lahmar^1^, Zeineb Hammouda^1^, Lahmar Manel^1^, Wiem Nouira^1^, Fahmi Dachraoui^1^, Fekri Abroug^1^, Lamia Ouanes Besbes^1^

#### ^1^University Hospital Fattouma Bourguiba of Monastir, Monastir, Tunisie

##### **Correspondence:** Iyed Matouk (maatouk.yed@gmail.com)

*Annals of Intensive Care* 2023, **13(Suppl 1):**FC-0182

**Rationale:** Coronavirus infection disease 2019 (COVID-19) is considered as a global health emergency. Acute Kidney Injury (AKI) is frequent in COVID-19. The primary goal was to describe the characteristics of moderate-severe AKI of COVID-19 patients in an ICU context. As a secondary goal, we aimed to find independent predictors of AKI progression, Renal Replacement therapy (RTT) requirement and mortality among critically ill COVID-19 patients.

**Patients and methods/materials and methods:** We conducted an observational study of COVID-19 patients between 2020 and 2022 in the Intensive Care Unit (ICU) of a Tunisian University Hospital. We included COVID-19 patients who developed AKI aged more than 18 years admitted to ICU for severe ARDS due to SARS-COV2. KDIGO clinical practice Guideline was used to identify AKI.

**Results:** During the study period, 433 patients were admitted to our ICU for COVID-19 ARDS. The majority were male (66.7%). Mean age was 59.6 ± 13 years. Obesity was found among 38.3% of patients. Median SAPSII was 29 (Interquartile range (IQR): 23.7–35). Median APACHE was 6 (IQR: 9–12). Median length of stay was 10 (IQR: 6–14) days. Almost a third of the patients (30%) presented AKI of whom 7% required RTT. Mortality was more frequent in patients with AKI than those without AKI (89.5% vs 5.6%; p < 0.01). AKI was more frequent in patients with Hypertension (42.2% vs 22%; p < 0.01), patients with diabetes (40.1% vs 24%; p = 0.001), patients with dyslipidemia, (54.8% vs 33.6%; p = 0.028), patients with SOH (83.3% vs 35.4%; p = 0.027), septic shock (63.6% vs 5.8%; p < 0.01), patients with IMV (59.2% vs 3.9%; p < 0.01). A significant association was found between AKI and BMI (p = 0.03), SAPS (p < 0.01), APACHE (p < 0.01) and SOFA (p < 0.01), In multivariate analysis, factors independently associated with AKI were Hypertension (OR: 1.83; 95% CI [1.005–3.335]; p = 0.048), BMI (OR: 1.06; 95% CI [1.006–1.126]; p = 0.031), SAPS (OR: 1.041; 95% CI [1.008–1.075]; p = 0.015), and APACHE II (OR: 1.147; 95% CI [1.058–1.244]; p = 0.001).

**Conclusion:** Hypertension, BMI, SAPS, APACHE II were independently associated with AKI. Mortality was high among critically ill COVID-19 patients with AKI. Other studies should be conducted in order to identify factors associated with mortality among critically ill COVID-19 patients.

**Compliance with ethics regulations:** Yes in clinical research.

## FC-183 Frequency and prognosis of acute kidney injury in critically ill COVID-19 patients

### Nabil Bouguezzi^1,2^, Imen Ben Saida^1,2^, Radhouane Toumi^1,2^, Emna Ennouri^1,2^, Rym Chelbi^1,2^, Azer Yaacoub^1,2^, Rihab Rajah^1^, Imen Belhouchet^1,2^, Khaoula Meddeb^1,2^, Mohamed Boussarsar^1,2^

#### ^1^University of Sousse, Faculty of Medicine of Sousse, Sousse, Tunisie; ^2^Farhat Hached University Hospital, Medical Intensive Care Unit, Research Laboratory “Heart Failure”, LR12SP09, Sousse, Tunisie

##### **Correspondence:** Nabil Bouguezzi (dr_nabil@live.fr)

*Annals of Intensive Care* 2023, **13(Suppl 1):**FC-0183

**Rationale:** Acute Kidney Injury (AKI) has been reported to be quite prevalent even earlier within the evolution of COVID-19. We aimed to evaluate the frequency, and prognosis of AKI in critically ill COVID-19 patients.

**Patients and methods/materials and methods:** We performed a retrospective cohort study of adult patients with COVID-19 diagnosis admitted to the intensive care unit between March 2020 and December 2021. Clinical characteristics and laboratory data were extracted from electronic files and laboratory databases. AKI was defined and staged according to the 2012 Kidney Disease: Improving Global Outcomes criteria. The association of AKI with 28-day mortality was assessed. Kaplan Meier survival curves for 28-day mortality by KDIGO stages were used.

**Results:** A total of 465 COVID-19 patients were admitted during the study period. Patients’ characteristics were: mean age, 62 ± 13.7 years; male, 290 (62.4%); mean SAPSII, 32.32 ± 11.3; IMV, 244 (52.5%). AKI was diagnosed in 229 (49.2%) patients. It occurred within a mean of 4.59 ± 3.67 days. Thirty (13.1%) patients were stratified as stage 1; 27 (11.8%), stage 2; and 172 (75.1%), stage 3 AKI. The overall 28-day mortality was 228(49%). A stepwise increase in the stage of AKI conferred an incremental risk of 28-day mortality as shown in Figure 1. A total of 135 (58.9%) patients with AKI died in hospital, and the mortality in AKI stage 1, stage 2 and stage 3 was 43%, 44%, and 63.9%, respectively. Forty-seven (20.5%) patients received renal replacement therapy, with a total of 117 sessions. Length of stay was at 8 [5–14], 7 [4–11] and 9 [5–15] days in AKI stage 1, stage 2 and stage 3, respectively.

**Conclusion:** AKI is common and carries high in 28-day mortality in critically ill COVID-19 patients.

**Compliance with ethics regulations:** Yes in clinical research.

**Figure 1 (abstract FC-183):**
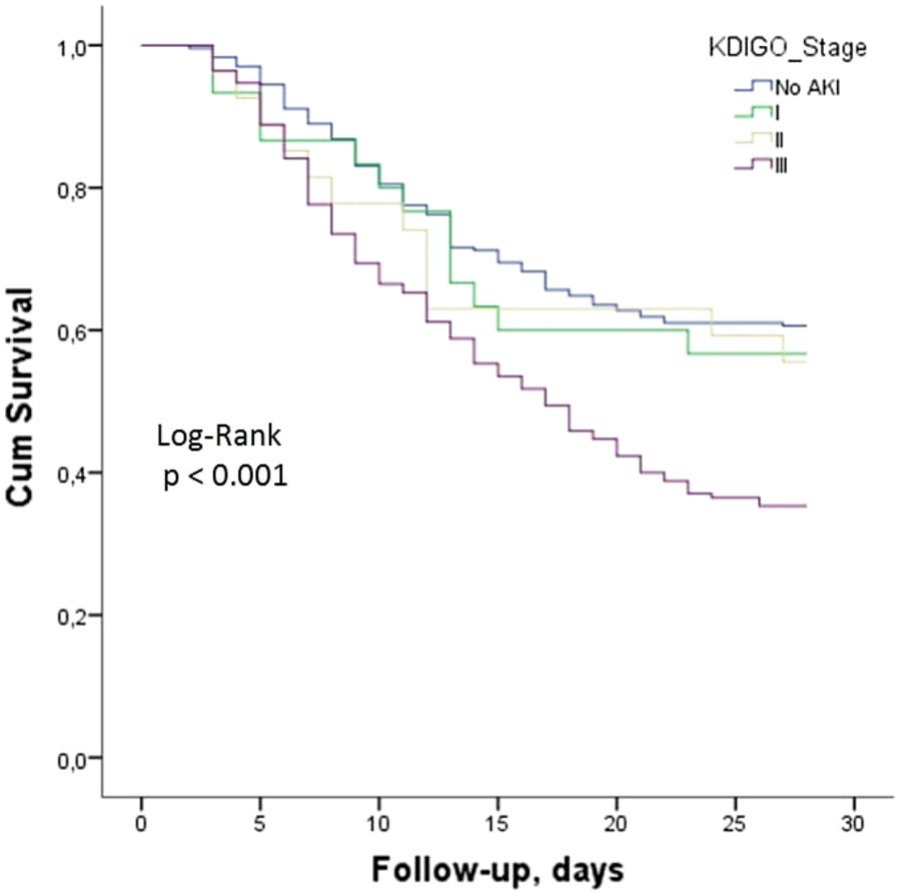
Kaplan–Meier curves for ICU mortality according to the stages of AKI

## FC-184 Characteristics and prognosis of acute renal injury associated with SARS-CoV-2 ARDS

### Maxime Mouasseh^1^, Kais Regaieg^1^, Giulia Moratelli^1^, Dany Goldgran-Toledano^1^

#### ^1^GHI Montfermeil, Montfermeil, France

##### **Correspondence:** Kais Regaieg (kais.regaieg@gmail.com)

*Annals of Intensive Care* 2023, **13(Suppl 1):**FC-0184

**Rationale:** Rationale: to describe the epidemiological, clinical and paraclinical characteristics of patients with acute renal injury (AKI) associated with SARS-CoV-2 ARDS and to identify predictive factors for dialysis and mortality.

**Patients and methods/materials and methods:** A retrospective, single-center study of all patients with AKI associated with severe SARS-CoV2 ARDS, hospitalized between March 2020 and March 2022. The primary endpoint was mortality in ICU.

**Results:** During the study period, 88 patients were included. The mortality rate was 52%. The mean SAPS II score was 40.4. The average age was 64.2 years. The sex ratio was 2.82 (M/F). The majority of patients had comorbidities. The reason for admission in ICU was acute respiratory failure. During hospitalization, 72% of patients had mechanical ventilation, 74% received vasoactive amines and 34% benefited from dialysis. The average duration of mechanical ventilation was 13.7 days. The average time to use dialysis was 3.03 days and the average duration of dialysis in surviving patients was 20.42 days. In multivariate analysis, the predictive factors for dialysis were diabetes, high uremia, high creatinine, high serum potassium, low pH. The predictive factors for mortality were: high uremia, high creatinine, high serum potassium, low pH, use of dialysis, use of vasoactive amines and mechanical ventilation.

**Conclusion:** Acute renal injury in patients with severe SarS CoV2 ARDS is frequent and associated with higher mortality. The mechanism of this AKI remains poorly understood and seems to be of multifactorial origin. Predictive factors for dialysis appear similar to other AKI. Further studies on a larger scale are needed to conclude.

**Compliance with ethics regulations:** Yes in clinical research.

## FC-185 Prognostic value of acute kidney injury (AKI) duration in patients with COVID-19 in intensive care unit

### Yosri Ben Ali^1^, Dhouha Hamdi^1^, Hend Zorgati^1^, Dhouha Ben Braiek^1^, Sourour Bel Haj Youssef^1^, Khalil Attia^1^, Rahma Ben Jazia^2^, Amani Kacem^2^, Jihene Ayachi^1^

#### ^1^Medical Intensive Care Unit, Ibn El Jazzar University Hospital, Kairouan, Tunisie; ^2^Pulmonology Department, Ibn El Jazzar University Hospital, Kairouan, Tunisie

##### **Correspondence:** Jihene Ayachi (ayachijihen@gmail.com)

*Annals of Intensive Care* 2023, **13(Suppl 1):**FC-0185

**Rationale:** An alarming number of patients with COVID 19 infection, especially severe cases develop acute kidney injury (AKI). Those patients have extremely high rates of mortality. Aim: To evaluate the prognostic impact of AKI duration on in-hospital mortality in critically ill COVID 19 patients.

**Patients and methods/materials and methods:** A retrospective analytic study performed in a 9-bed medical ICU of a university hospital from September 2020 to December 2021 including patients with confirmed ARDS related COVID 19 pneumonia. Demographic and clinical characteristics are recorded from medical patients’ charts. AKI is defined according to the KDIGO clinical practice Guideline. AKI patients were divided into two groups: G1 transient AKI and G2 persistent AKI based on whether serum creatinine level returned to baseline within 48 h post-AKI. Statistically significant variables found in univariate analysis were used to compare the two groups.

**Results:** During the study period 172 patients were included. Mean age was 51.5 ± 14.4 years, and 104 (60.5%) patients were men. AKI occurred in 54 (31.4%) patients during their ICU stay, among them, 16 (29.6%) had transient AKI, and 38 (70.4%) had persistent AKI. The most common comorbidities found in persistent AKI group compared to transient AKI group were: diabetes, arterial hypertension and obesity in respectively 39.5% vs 37.5%, 29.1% vs 31.2% and 47.4% vs 31.25% without significant difference between the two groups. The mean Creatinine and urea blood level were higher in the persistent AKI group with respectively 118 [154–646] vs 98 [45–151] µmol/L and 11.8 vs 8.6 mg/l without significant difference. Vasoactive drug use was 63% in persistent AKI group versus 43.7% in the other group with significant difference p = 0.042. In-hospital mortality was 29 (24.6%) for patients without AKI, 7 (43.8%) for patients with transient AKI, and 34 (89.5%) for patients with persistent AKI with significant differences between the groups (P < 0.001). Kaplan–Meier curve analysis revealed that patients with both transient AKI and persistent AKI had significantly an increased mortality than those without AKI (log-rank P = 0.016).

**Conclusion:** Among patients with AKI, an increased mortality has been observed in those with persistent acute renal injury. The AKI duration should be rapidly recognized to identify the appropriate treatment for early intervention.

**Compliance with ethics regulations:** Yes in clinical research.

## FC-186 Early versus late acute kidney injury among critically ill COVID-19 patients

### Nabil Bouguezzi^1,2^, Imen Ben Saida^1,2^, Emna Ennouri^1,2^, Radhouane Toumi^1,2^, Rihab Rajah^1^, Rym Chelbi^1,2^, Azer Yaacoub^1,2^, Khaoula Meddeb^1,2^, Mohamed Boussarsar^1,2^

#### ^1^University of Sousse, Faculty of Medicine of Sousse, Sousse, Tunisie; ^2^Farhat Hached University Hospital, Medical Intensive Care Unit, Research Laboratory “Heart Failure”, LR12SP09, Sousse, Tunisie

##### **Correspondence:** Nabil Bouguezzi (dr_nabil@live.fr)

*Annals of Intensive Care* 2023, **13(Suppl 1):**FC-0186

**Rationale:** Acute kidney injury (AKI) is an important complication of coronavirus disease 2019 (COVID-19), which could be caused by both systematic responses from multi-organ dysfunction and direct virus infection. While advanced evidence is needed regarding its clinical features and mechanisms. We aimed to describe two phenotypes of AKI as well as their risk factors and their association with mortality.

**Patients and methods/materials and methods:** This is an analytic retrospective study carried out in a medical intensive care unit from March 2020 to December 2021. All patients who developed AKI were included. Clinical characteristics and laboratory data were extracted from electronic hospitalization and laboratory databases. AKI was defined and staged according to the 2012 Kidney Disease: Improving Global Outcomes criteria. Patients with AKI were classified as early AKI and late AKI; Early AKI was defined as diagnosed at ≤ 72 h after intensive care unit (ICU) admission, with any diagnosis > 72 h denoted as late AKI.

**Results:** A total of 465 cases with laboratory-confirmed COVID-19 were included and 229 (49%) of them were identified as AKI. It occurred within a mean of 4.59 ± 3.67 days. 86 (37.5%) patients were defined as early AKI and 143(62.5%) as late AKI. Compared with patients with late AKI, patients with early AKI had significantly higher SAPS II score; 37.6 ± 14.3 versus 32.6 ± 10.1, p = 0.005 and higher levels of systemic inflammatory markers (CRP); 203 ± 106 versus 151 ± 75, p = 0.05. Only hypertension and coronary disease were associated with the risk of early AKI while shock and right heart failure were associated with the risk of late AKI. Patients with late AKI had a higher mortality 42 (65%) than early AKI; 93 (48.8%) p = 0.016 and a significantly prolonged length of stay 12.3 ± 4.6 versus 7.5 ± 3.8 days, p < 0.001.

**Conclusion:** AKI among patients with COVID-19 has two clinical phenotypes, which could be due to different mechanisms. Considering the increased risk for mortality for both phenotypes, monitoring for AKI should be emphasized during COVID-19.

**Compliance with ethics regulations:** Yes in clinical research.

**Figure 1 (abstract FC-186):**
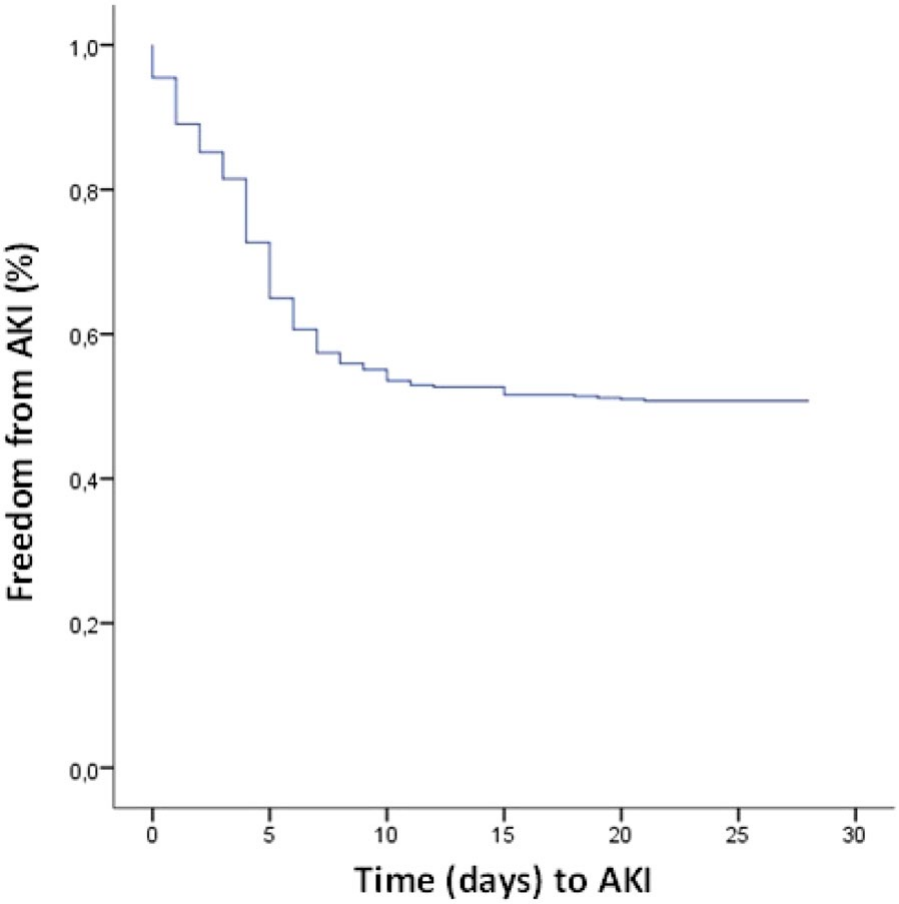
Kaplan–Meier estimates of freedom from acute kidney injury in critically ill COVID-19 patients

## FC-187 Risk of preeclampsia in women living in coastal areas affected by sargassum stranding on the French island of Martinique

### Dabor Resiere^1^, Hossein Mehdaoui^1^, Jonathan Florentin^1^, Hatem Kallel^2^, Moustapha Drame^1^, Remi Neviere^1^

#### ^1^Chu de Martinique, Fort-de-France, Martinique, France; ^2^CH de Cayenne, Cayenne, Guyane Francaise

##### **Correspondence:** Dabor Resiere (dabor.resiere@chu-martinique.fr)

*Annals of Intensive Care* 2023, **13(Suppl 1):**FC-0187

**Rationale:** Since the beginning of 2011, an increasing proliferation of sargassum algae has been observed on the coasts of the Caribbean islands, including Martinique and Guadeloupe. After 48 h of stranding, due to the decomposition of organic matter, the algae produce large quantities of toxic gases, including hydrogen sulfide (H2S) and ammonia (NH3). The toxicity to humans of exposure to high concentrations of H2S is well described. The severity of the symptoms increases proportionally with exposure, leading to potentially fatal respiratory, neurological, and cardiovascular disorders, especially in comorbidities. Conversely, the health consequences of chronic or repeated exposure to the gases emitted by decomposing sargassum are much less known. This physiological gasotransmitter, fulfilling a large number of biological functions, is currently under evaluation as a therapeutic avenue in many medical fields, especially in ischemic situations. Pre-eclampsia is a global vascular pathology of the 3rd trimester of pregnancy, whose deficiency in the endogenous H2S synthesis circuits has been demonstrated in some animal studies. Our objective was to study the risk of preeclampsia in pregnant women living in coastal areas regularly affected by massive sargassum strandings.

**Patients and methods/materials and methods:** Three thousand twenty records of pregnant women who gave birth in the Department of Gyneco-obstetrics of the University Hospital of Martinique between 2016 and 2020 were retrospectively analyzed. An exposure index was assigned based on the average H2S concentration at the closest recording site to the home over the gestation period, taking into account the distance to the shoreline of the house. Distance to shoreline Sargassum stranding sites was characterized as follows: < 500 m, 500 m–2 km, > 2 km.

**Results:** These results showed that time to preeclampsia was significantly shorter in women living within ≤ 2 km (mean survival time of 32 weeks of amenorrhea) compared with those living beyond 2 km (mean survival time of 35 weeks of amenorrhea, p = 0.037).

**Conclusion:** In addition to traditional risk factors, environmental exposure to Sargassum strandings could potentially trigger the early onset of preeclampsia.

**Reference 1**

1- de Lanlay DB, Monthieux A, Banydeen R, Jean-Laurent M, Resiere D, Drame M, Neviere R. Risk of preeclampsia among women living in coastal areas impacted by sargassum strandings on the French Caribbean island of Martinique. Environ Toxicol Pharmacol. 20.

**Compliance with ethics regulations:** N/A.

## FC-188 Epidemiological aspects and maternal prognosis of eclampsia in the intensive care unit of the CHU Gabriel Touré, Bamako, Mali

### Diop Thierno Madane^1^, Moustapha Issa Mangane^1^, Abdoul Hamidou Almeimoune^1^, Ngompe Junior Olsen ^1^, Alfousseini Soumare^1^, Andre Kassogue^1^, Daouda Diallo^2^, Mahamadoun Coulibaly^3^, Drissa Kaloga Bagayoko^3^, Kassoum Ouattara^5^, Aminata Dabo^3^, Aladji Seidou Dembele^4^, Mahamane Djibo Diango^1^

#### ^1^CHU Gabriel Toure, Bamako, Mali; ^2^CHU Kati, Bamako, Mali; ^3^CHU Luxembourg, Bamako, Mali; ^4^CHU IOTA, Bamako, Mali; ^5^Hôpital du District Commune II, Bamako, Mali

##### **Correspondence:** Diop Thierno Madane (madane.diop@gmail.com)

*Annals of Intensive Care* 2023, **13(Suppl 1):**FC-0188

**Rationale:** In sub-Saharan Africa, and particularly in Mali, pre-eclampsia and its complications are frequent and have significant morbidity and mortality.

**Patients and methods/materials and methods:** This was a 12-month prospective and descriptive study from January 1, 2021 to December 31, 2021 including all patients admitted for eclampsia. The diagnosis was defined by convulsive seizures associated with positive proteinuria in the peripartum period.

**Results:** We collected data from 268 patients among whom 39 had eclampsia (14.5%). The mean age was 21.03 ± 4.83 years. Housewives represented 53.8% and were primiparous in 61.5% with no medical history in 94.7%. Among the patients, 17.9% did not have a prenatal consultation (CPN) and more than half of them 64.1% had more than 3 seizures. At admission 63.8% had proteinuria ≥ 3 crosses and 1.,4% had a Glasgow score ≤ 8. The systolic blood pressure was > 140 mmHg in 46% of the patients and above 160 mmHg in 27.7%. The therapeutic protocol based on magnesium sulphate (MgSO_4_) was initiated in 92.3% associated with antihypertensives including nicardipine and labetalol, respectively in 30.8% and in 28.2% of patients. Caesarean section was the most frequent mode of delivery with 66.7%. Associated complications were Hellp syndrome (30.8%), acute kidney injury (23.1%) and cardiac failure (2.6%). Maternal lethality was 15.4%.

**Conclusion:** Eclampsia is common in our country in young patients. Morbimortality is high. Better awareness could improve the prognosis.

**Compliance with ethics regulations:** Yes in clinical research.

## FC-189 Peripartum neurovascular emergencies

### Rabeb Hammami^1^, Mahmoud Marzouk^1^, Wassim Bourhil^1^, Asma Hosni^1^, Sabeur Thamlaoui^1^, Nader Baffoun^1^, Chokri Kaddour^1^

#### ^1^Institut National De Neurologie, Tunis, Tunisie

##### **Correspondence:** Rabeb Hammami (hammamirabeb2@gmail.com)

*Annals of Intensive Care* 2023, **13(Suppl 1):**FC-0189

**Rationale:** Peripartum neurovascular emergencies are rare but potentially life-threatening. The aim of this study was to identify prepartum and postpartum neurovascular emergencies and to study their epidemiological, clinical, radiological, and evolutionary aspects.

**Patients and methods/materials and methods:** This was a retrospective descriptive study conducted in our intensive care unit over a 12-year period from 2010 to 2021. Pregnant and postpartum patients with cerebrovascular pathology such as arterial ischemic stroke, venous thrombosis, eclampsia, and ruptured vascular malformations were included. The data collected were: age, geographical origin, medical, gynaeco-obstetrical and family history, pregnancy follow-up, the reasons for admission, clinical presentation, radiological and biological investigations, obstetrical and medical management, evolution, and mortality in intensive care.

**Results:** 45 patients were selected for the study. The average age was 29 years ± 5. Eleven percent of patients were from the greater Tunis area, 15.5% from Bizerte and 11.1% from the northwest. The cases studied were dominated by eclampsia in 77.77% of the cases followed by cerebral venous thrombosis (CVT) in 11.1% of cases, ischemic stroke (iCVT) in 8.9% in cases, and one case of a combination of iCVT and CVT. 77.77% occurred in prepartum and 22.2% in postpartum. 64.4% of patients were primiparous. Epilepsy was found in 4.4% of cases, hypertension in 2.2%, and a history of gravid hypertension in 6.6%. The revealing signs were dominated by convulsions in 40%, signs of pre-eclampsia in 28.8%, agitation in 13.33%, and neurological deficit in 2.2%. The main reason for admission to the intensive care unit was epilepsy in 44.4% of cases, coma in 37.7%, agitation in 8.9%, and neurological deficit in 6.6% of cases. The average time between the first symptom and admission to the intensive care unit was 1.8 days ± 3.5. The patients were investigated by brain CT in 71.1% of cases and MRI in 31.1%. The abnormalities noted were PRES syndrome in 31.1% of cases, intracerebral hematoma in 15.6%, CVT in 8.8%, cerebral edema in 8.8%, mass effect, and meningeal hemorrhage in 6.6%. Mortality was 6.6%. The average time to the hospitalization in the intensive care unit in the event of mortality was 3.6 days ± 4.61, without this being significant (p = 0.376).

**Conclusion:** Management must be multidisciplinary, involving obstetricians, resuscitators, and radiologists. Imaging plays an important role in the positive and etiological diagnosis, thus improving the management and the maternal prognosis.

**Compliance with ethics regulations:** Yes in clinical research.

## FC-190 First evaluation of BioFire® pneumonia plus panel on bronchial blind samples in pediatric intensive care unit and its impact on early adaptation of antimicrobial therapy

### Guillaume Geslain^1^, Aurélie Cointe^1^, Jérôme Naudin^1^, Stéphane Dauger^1^, Nora Poey^1^, Justine Pages^1^, Enora Le Roux^1^, Stéphane Bonacori^1^

#### ^1^CHU Robert Debré, Paris, France

##### **Correspondence:** Guillaume Geslain (guillaume.geslain@aphp.fr)

*Annals of Intensive Care* 2023, **13(Suppl 1):**FC-0190

**Rationale:** Community-acquired and nosocomial lower respiratory infections in Pediatric Intensive Care Unit (PICU) are a major public health problem. We aimed to assess the validity of the new rapid-multiplex PCR (RM-PCR), BioFire® Pneumonia plus Panel test, a, on respiratory blind bronchial samples (BBS) compared to the reference method and to determine its potential impact on therapeutic management.

**Patients and methods/materials and methods:** We conducted a monocentric, prospective study in a PICU. We collected data on patients hospitalized under 18 years old with suspicion of community acquired pneumonia, hospital-acquired pneumonia or ventilator-associated pneumonia between April 2021 and January 2022 and analyzed their samples with the new RM-PCR and the reference techniques (culture and antibiogram).

**Results:** Thirty-six patients (median age of 1.4 years) were included comprising 41.7% of community-acquired pneumonia, 27.8% hospital-acquired pneumonia and 30.6% ventilator-associated pneumonia. The kappa coefficient between both techniques was 0.74 showing a good strength of agreement. The new RM-PCR test had a sensitivity and specificity of 92% (IC 95%: 77–98) and 95% (IC 95%: 92–97) respectively. The median time between the sample collection and the result of antibiogram was 3.9 h [2.5; 15] with the tested panel versus 60.5 h [47.6; 72.2] with the reference technique. A therapy de-escalation could have been applied in 76% of the patients based on the RM-PCR results.

**Conclusion:** The BioFire® Pneumonia plus Panel had good diagnostic performance on BBS in pediatric critically ill patients. It may guide efficiently the antibiotic therapy adjustment by changing its spectrum and may help the decision of stopping antibiotic treatment.

**Compliance with ethics regulations:** Yes in clinical research.

## FC-191 Description of milrinone use in fluid-refractory septic shock in children: doses, frequency of administration, complications and mortality

### Gauthier Bonjour^1^, Emeline Caillau^3^, Etienne Javouhey^2^, Jérémie Rousseaux^1^, Marie-Emilie Lampin^1^, Morgan Recher^1^, Stéphane Leteurtre^1^

#### ^1^Réanimation Pédiatrique Hôpital Jeanne de Flandre, Lille, France; ^2^Réanimation Pédiatrique Hôpital Femme-Mère-Enfant, Lyon, France; ^3^Biostatistics Department CHU de Lille, Lille, France

##### **Correspondence:** Gauthier Bonjour (gauthierbonjour@gmail.com)

*Annals of Intensive Care* 2023, **13(Suppl 1):**FC-0191

**Rationale:** Septic shock is a frequent, life-threatening condition in children. Although management is well-described concerning fluid-therapy and vasopressor, data lacks about septic cardiomyopathy treatment and inotropes use. Milrinone, a non-adrenergic inodilatator, used in cardiac surgery, could be useful in septic shock. The first objective was to describe the use, complications of milrinone in a population of fluid-refractory septic shock. The second objective was to compare outcomes between patients with milrinone and patients without milrinone.

**Patients and methods/materials and methods:** We conducted a retrospective single-centre review of children, hospitalized in PICU, who presented an episode of septic shock (CIM-10 code R572), fluid-refractory and receiving vasopressors (norepinephrine, dopamine) or inotropes (epinephrine, dobutamine, milrinone), between 2011 and 2021. Variables analyzed was doses of milrinone, frequency of use and complications. Outcomes was the PICU-mortality and the PICU-length of stay, compared in two groups: patients with and without milrinone.

**Results:** We included 130 patients with septic shock. The median volume resuscitation before vasoactive agent was 40 ml/kg (IQR 26.7–60). 39 (30%) received milrinone. Milrinone was used in 18 patients (14%) as the first-line inotropic drug whereas dobutamine was the most employed drug [(52 patients, 40%) and 44 (34%) as first-line]. The incidence of milrinone use over years was inconstant with a tendency to grow in the last 2 years. Median initial dose of milrinone was 0.5 mcg/kg/min (IQR 0.4–0.7) and median maximum dose was 0.6 mcg/kg/min (IQR 0.5–0.8). Compared to the groups without milrinone, patients with milrinone had more frequent cardiac arrythmia (33% vs 5.5%, p < 0.001), a mean PELOD-2 score higher at H24 and H48 of the beginning of septic shock (H24: 10.5 ± 4.5 vs 7.8 ± 4.2, p < 0.002/H48: 8.1 ± 4.4 vs 6.1 ± 3.2, p 0.029), a median vaso-inotropic score higher (244.5, IQR 80.3–412, versus 40 IQR 20–105, p < 0.001), and more cardiac arrest (51.3% versus 10%, p < 0.001). Adjusted on antibiotic administration delay, vaso-inotropic score, use of mechanical ventilation and of renal replacement therapy, mortality was not higher in milrinone group vs without milrinone (HR = 1.482, IC95% 0.552–3.976, p 0.434) and length of stay was not different (HR 0.731, IC 0.427–1.252, p 0.254).

**Conclusion:** Our study showed that milrinone is frequently used as inotropic drug in septic cardiomyopathy. Despite the patients seemed to have a more serious condition and more cardiac arrythmias, use of milrinone was not associated with higher PICU mortality or length of stay.

**Reference 1**

Weiss SL, Chair CV, Peters MJ, Chair CV, Alhazzani W, Flori HR, et al. Surviving sepsis campaign international guidelines for the management of septic shock and sepsis-associated organ dysfunction in children. Pediatr Crit Care Med. 2020;21(2):55.

**Reference 2**

Barton P, Garcia J, Kouatli A, Kitchen L, Zorka A, Lindsay C, et al. Hemodynamic effects of IV milrinone lactate in pediatric patients with septic shock. Chest. Mai 1996;109(5):1302–12.

**Compliance with ethics regulations:** Yes in clinical research.

## FC-192 Is eosinopenia a prognostic factor in children with bacteremia?

### Kaoutar El Fakhr^1^, Wissal Aissaoui^1^, Samira Kalouch^1^, Abdelaziz Chlilek^1^

#### ^1^CHU Ibn Rochd, Casablanca, Maroc

##### **Correspondence:** Kaoutar El Fakhr (kaoutarelfakhr2013@gmail.com)

*Annals of Intensive Care* 2023, **13(Suppl 1):**FC-0192

**Rationale:** Bacteremia is a common cause of increased mortality in children. It is now acknowledged that eosinopenia is a marker of infection and/or severity of the systemic inflammatory response.

**Patients and methods/materials and methods:** We retrospectively included 30 pediatric patients (≤ 18 years old) admitted for documented bacteremia between June 2022 and December 2022. The primary outcome was a positive blood culture. The white blood cell was systematically gathered at the admission to evaluate the eosinophil count. Overall survival and progression-free survival in relation to the number of eosinophils after 48 h of antibiotic therapy were estimated by the Kaplan–Meier method.

**Results:** A total of 30 patients were included in this study. The median age was 2.5 ± 3.6 years. 63% of patients were female, 96.7% had eosinopenia before antibiotic therapy, 83.3% had persistant eosinopenia after antibiotic therapy with a statistically significant association with mortality p < 0.001.

**Conclusion:** The persistence of eosinophilia 48 h after antibiotic therapy during bacteremia appears predictive of short- and long-term mortality.

**Compliance with ethics regulations:** Yes in clinical research.

**Figure 1 (abstract FC-192):**
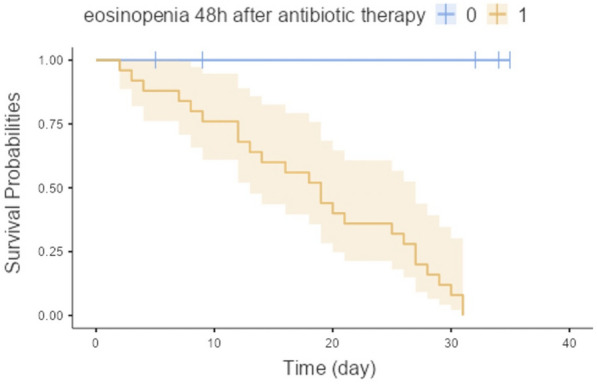
Survival curve

## FC-193 Piperacillin population pharmacokinetics in children under extracorporeal membrane oxygenation assistance

### Adrien Arsene^1^, Mehdi Oualha^1,3^, Sihem Benaboud^3^, Saik Urien^3^, Florence Moulin^1^, Fabrice Lesage^1^, Sylvain Renolleau^1^, Jérome Rambaud^2^, Pierre-Louis Leger^2^, Deborah Hirt^3^, Jean-Marc Treluyer^1,3^, Agathe Béranger^1,3^

#### ^1^Hôpital Necker, Paris, France; ^2^Hôpital Trousseau, Paris, France; ^3^CIC Paris Centre, Paris, France

##### **Correspondence:** Adrien Arsene (ad.arsene@gmail.com)

*Annals of Intensive Care* 2023, **13(Suppl 1):**FC-0193

**Rationale:** Extracorporeal membrane oxygenation (ECMO) is a rising technology for the treatment of extremely severe respiratory or circulatory failure. It has been reported that this device can interact with drugs pharmacokinetic [1]. This can be challenging in children whose pharmacokinetic is already complex due to growth and maturation. Piperacillin is a common broad-spectrum antibiotic used in intensive care. We aimed to study if ECMO influenced the pharmacokinetic of Piperacillin.

**Patients and methods/materials and methods:** Thirty-three ECMO and forty-four non ECMO patients were enrolled. Pharmacokinetic parameters were estimated by a non-linear mixed effect model using Monolix© along with the stochastic approximation of expectation maximization algorithm (SAEM)[2]. Tested covariates were: weight, renal function, ECMO presence and ECMO related covariates. Dosing regimen was optimized using simulation.

**Results:** Final model was a mono-compartmental model with linear elimination and associated covariates were weight in a standard allometric fashion, estimated glomerular filtration rate and indexed ECMO output on clearance, and ECMO circuit volume to weight ratio on volume. Simulations showed that no doses adaptation was needed and continuous infusion was the best choice.

**Discussion:** It is the first study which aims to evaluate the impact of ECMO on piperacillin pharmacokinetics in critically ill children. However, the number of samples was barely sufficient and the ECMO and non ECMO groups’ severity were different. Future studies should take at least two blood samples per patient and the sampling moment should be wisely decided with a population plan optimization based on the population Fisher information matrix (PFIM).

**Conclusion:** ECMO significantly impacted piperacillin pharmacokinetics, but these modifications were not important enough to adapt the dosing regimen.

**Reference 1**

Shekar K, Fraser JF, Smith MT, Roberts JA. Pharmacokinetic changes in patients receiving extracorporeal membrane oxygenation. J Crit Care. 2012;27(6):741.e9–18.

**Reference 2**

Kuhn E, Lavielle M. Coupling a stochastic approximation version of EM with an MCMC procedure. ESAIM Probab Stat. 2004;8:115–31.

**Compliance with ethics regulations:** Yes in clinical research.

## FC-194 Impact of the pharmacist on the management of antibiotics in pediatric intensive care unit

### Omar Hanafia^1^, Marie Tabon^1^, Pierre Bertault-Peres^1^, Stéphane Honore^2^, Fabrice Michel^1^

#### ^1^Hôpitaux Universitaires de Marseille/AP-HM, Marseille, France; ^2^Aix Marseille Université/Faculté de Pharmacie, Marseille, France

##### **Correspondence:** Omar Hanafia (omarhanafia@yahoo.fr)

*Annals of Intensive Care* 2023, **13(Suppl 1):**FC-0194

**Rationale:** The objective of this work was to study the impact of the pharmacist involvement on the antibiotic therapeutic follow-up in a pediatric intensive care unit.

**Patients and methods:** It was a retrospective, monocentric study over a 2-months period. For each patient we collecteded the antibiotic prescription, the reasons for dosage and whether it was suggested by a pharmacist or directly initiated by a physician.

**Results:** Seventeen patients were included, with a mean length of stay of 28 days. Assays were 35% in patients with no change in pharmacokinetics and 65% in those with a change. 36.5% of patients had circulatory support. 85 measurements were performed: 38 were on target, 42 revealed an under or overdosage. On 16 normal assays, 5 were pharmaceutical-initiated (31%) and 11 were medical-initiated (69%). On 15 off-target assays 8 were from a pharmacist request (53.3%) and 7 from a physician request (46.7%). Following, the pharmacist intervened again for therapeutic optimization: 54% for a new monitoring and 46% for a dosage adjustment.

**Conclusion:** Our study showed that half of the dosages performed were outside the target range. We also showed that the pharmacist was able to better identify at-risk patients. The pharmaceutical team therefore had an important impact on the monitoring and adaptation of antibiotic therapy.

**Compliance with ethics regulations:** N/A.

## FC-195 The initial fluid volume required for resuscitation of septic shock patients depends on the source of infection

### Rui Shi^1^, Simone Cappio Borlino^1^, Francesca Moretto^1^, Eduardo Rocca^1^, Marta Fasan^1^, Wasineenart Mongkolpun^2^, Simone Carelli^1^, Christopher Lai^1^, Jean-Louis Teboul^1^, Xavier Monnet^1^

#### ^1^Université Paris Saclay, AP-HP, Service de médecine intensive-réanimation, Hôpital Kremlin Bicêtre, Le Kremlin-Bicêtre, France; ^2^Université Libre de Bruxelles, Intensive care department, Erasme Hospital, Bruxelles, Belgique

##### **Correspondence:** Simone Cappio Borlino (simone.cappio@unimi.it)

*Annals of Intensive Care* 2023, **13(Suppl 1):**FC-0195

**Rationale:** The Surviving Sepsis Campaign recommends an initial volume expansion of 30 mL/kg in all septic shock patients. However, the fluid volume required initially may differ with the sepsis source, which induces various degrees of absolute and relative hypovolemia. In this prospective, observational study in septic shock patients, we quantified the fluid volume received before the occurrence of preload unresponsiveness, i.e. a state in which fluid infusion does not increase cardiac output significantly.

**Patients and methods/materials and methods:** In patients at the initial phase of septic shock admitted in our intensive care unit, we repeatedly assessed preload responsiveness from inclusion, either through the response of cardiac output to a fluid bolus (134/196 measurements), or through a passive leg raising test (43/196 measurements), an end-expiratory occlusion test (12/196 measurements) or a tidal volume challenge (7/196 measurements). We quantified the volume of fluid received before the first occurrence of preload unresponsiveness.

**Results:** Among the 62 patients included (mean age: 63 ± 12 years), median left ventricular ejection fraction was 60 (48–65) %, the source of infection was lung for 27 (44%) patients, abdomen for 12 (19%) patients, urinary tract for 13 (21%) patients, skin and soft tissues for 6 (10%) patients and miscellaneous for 4 (6%) patients. The time from shock diagnosis to inclusion was 440 (182–1005) minutes. Forty (65%) patients had been previously admitted to the emergency department. At inclusion, 43 (69%) patients had already received 19 (12–36) mL/kg of crystalloids, 18 (29%) were already supported by norepinephrine [0.27 (0–0.80) µg/kg/min]. Only 43 (69%) patients were still preload-responsive at inclusion, and they evolved into a non-responsive state after 26 (20–42) mL/kg of fluid infused from shock diagnosis. This volume was 25 (19–38) mL/kg for pulmonary origin, 39 (25–61) mL/kg for abdominal origin, 34 (23–61) mL/kg for urinary tract origin, 21 (19–39) mL/kg for cutaneous and soft tissues origin (Figure). Among the preload-responsive patients, 24 (56%) became preload unresponsive before receiving 30 mL/kg of fluid: 13 (59%) patients in the lung group, 3 (50%) patients in the abdominal, 4 (50%) patients in the urinary, and 2 (67%) patients in the cutaneous.

**Conclusion:** The volume of resuscitation fluid required by septic shock patients at the initial phase seems to differ depending on the source of infection, abdominal sepsis requiring more fluid than others. A relevant number of patients with shock from pulmonary origin required fluid resuscitation < 30 mL/kg. The study is ongoing, but these primary results suggest a personalization of initial fluid therapy.

**Compliance with ethics regulations:** Yes in clinical research.

**Figure 1 (abstract FC-195):**
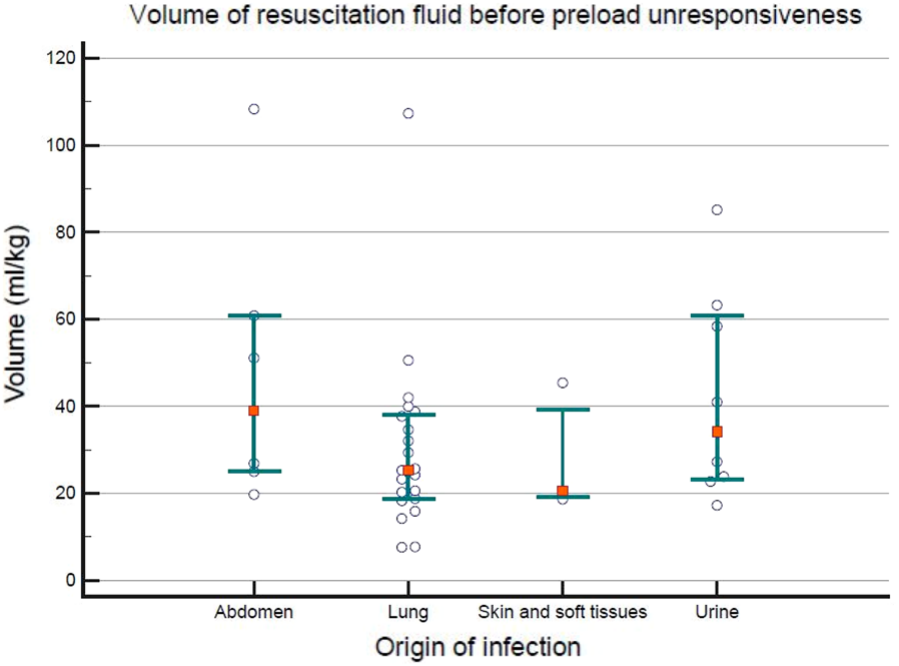
Volume of resuscitation fluid administrated before preload unresponsiveness

## FC-196 Assessing the effects of the tidal volume challenge for detecting preload responsiveness through the perfusion index of the plethysmographic signal in critically ill patients

### Chiara Bruscagnin^1,2^, Rui Shi^1^, Daniela Rosalba^1^, Gaelle Fouque^1^, Julien Hagry^1^, Katia Donadello^2^, Jean-Louis Teboul^1^, Xavier Monnet^1^

#### ^1^Hôpital de Bicêtre, Hôpitaux universitaires Paris-Saclay, AP-HP, Paris, France; ^2^Università di Verona, Verona, Italie

##### **Correspondence:** Chiara Bruscagnin (chiaragnin@gmail.com)

*Annals of Intensive Care* 2023, **13(Suppl 1):**FC-0196

**Rationale:** For detecting preload responsiveness in patients ventilated with a tidal volume (Vt) < 8 mL/kg, the Vt challenge consists in a 1-min increase in Vt from 6 to 8 mL/kg, whose effects are assessed through increases in pulse pressure variation (PPV). However, this requires an arterial catheter. It has been suggested that the perfusion index (PI), which reflects the amplitude of the plethysmographic signal, reflects stroke volume and that its respiratory variation (pleth variability index, PVI) is a surrogate of PPV. We assessed whether changes in PVI or in PI during a Vt challenge test could be as reliable as changes in PPV for detecting preload responsiveness.

**Patients and methods/materials and methods:** In critically ill patients mechanically ventilated with Vt < 6 mL/kg, monitored with a PiCCO_2_ system and a Masimo SET technique (plethysmography sensor placed on the finger), in whom a Vt challenge and a passive leg raising (PLR) test were performed, hemodynamic data were recorded during these interventions. Preload responsiveness was diagnosed by an increase in cardiac index (CI) ≥ 10% during PLR.

**Results:** Among the 63 screened patients, 14 were excluded because of poor PI signal, including 7 patients with atrial fibrillation. Among the included patients, 38.1% were preload responders. During the Vt challenge, PPV expressed in absolute value increased by 4.4 [1.9] in preload responders vs. 1.0 [1.0] in preload non-responders (p < 0.01). Simultaneously, PVI expressed in absolute value augmented by 1.9 [2.6] in preload responders vs. 0.3 [1.1] in preload non-responders (p = 0.01). PI decreased by 14 [11]% in preload responders vs 1 [8]% in preload non-responders (p < 0.01). The area under the ROC curve for detecting preload responsiveness was 0.95 (0.04) for the Vt challenge-induced changes in PPV in absolute value (p < 0.001 vs. 0.50), 0.74 (0.08) for the Vt challenge-induced changes in PVI in absolute value (p < 0.001 vs. 0.50, p = 0.03 vs. changes in PPV) and 0.86 (0.05) for the Vt challenge-induced changes in PI in percentage (p < 0.001 vs. 0.50, p = 0.48 vs. changes in PPV).

**Conclusion:** Changes in PI during a tidal volume challenge reliably detect preload responsiveness. Changes in PVI do the same, but with lower accuracy. This may offer a possibility to use this test in patients with no arterial catheter.

**Reference 1**

Cannesson M et al.: Pleth variability index to monitor the respiratory variations in the pulse oximeter plethysmographic waveform amplitude and predict fluid responsiveness in the operating theatre. Br J Anaesth 2008, 101(2):200–206.

**Reference 2**

Taccheri T, Gavelli F, Teboul JL, Shi R, Monnet X: Do changes in PPV and inferior vena cava distensibility during PLR and Vt challenge detect preload responsiveness in case of low tidal volume ventilation? Crit Care 2021, 25(1):110.

**Compliance with ethics regulations:** Yes in clinical research.

## FC-197 Effect of volume expansion and modification of norepinephrine infusion rate on capillary refill time in patients with septic shock

### Nicolas Fage^1,2,3^, Francesca Moretto^1^, Daniela Rosalba^1^, Rui Shi^1^, Christopher Lai^1^, Jean Louis Teboul^1^, Xavier Monnet^1^

#### ^1^Université Paris-Saclay, AP-HP, Service de médecine intensive-réanimation, Hôpital de Bicêtre, DMU CORREVE, Inserm UMR S_999, FHU SEPSIS, Groupe de recherche clinique CARMAS, Le Kremlin-Bicêtre, France; ^2^Département de Médecine Intensive Réanimation – Médecine Hyperbare, Centre Hospitalo-Universitaire d’Angers, Angers, France; ^3^Laboratoire MITOVASC UMR INSERM 1083 – CNRS 6015, Université d’Angers, Angers, France

##### **Correspondence:** Nicolas Fage (fage.nicolas@gmail.com)

*Annals of Intensive Care* 2023, **13(Suppl 1):**FC-0197

**Rationale:** The capillary refill time (CRT) is used for clinical monitoring during septic shock. However, whether CRT is influenced by macrohaemodynamic variables, such as cardiac output and vasomotor tone, is unclear, and the effects on CRT of volume expansion and norepinephrine remain uncertain. In septic shock patients, we investigated the determinants of the CRT and its changes during volume expansion or modification of norepinephrine infusion rate.

**Patients and methods/materials and methods:** In patients with septic shock, cardiac index (CI, transpulmonary thermodilution), arterial pressure and five CRT measurements were recorded before and after volume expansion (500 mL saline) and before and after modification of norepinephrine infusion rate. CRT was measured in a standardized way (standardized pressure applied on the fingertip under standardized light, computer-assisted measurement of skin color changes). Determinants of the initial value and changes of CRT (in percent change from baseline) were explored.

**Results:** We enrolled 69 patients with septic shock (lactate 2.7 [2.0–4.5] mmol/L, norepinephrine dose 0.43 [0.14–0.75] µg/kg/min), 33 receiving volume expansion and 36 in whom the norepinephrine dose was changed. The least significant change of CRT was 23%. In the 17 patients (51%) with fluid responsiveness (increase in CI ≥ 15% with volume expansion), CI increased from 2.34 [1.69–3.06] to 3.17 [2.28–4.03] L/min/m^2^ (p < 0.001). Among them, CRT decreased ≥ 23% in 9 (53%) patients and changed by less than 23% in the other ones (Figure 1A). In the 16 patients (49%) in whom CI changed < 15% with volume expansion, CRT decreased ≥ 23% in two (13%) patients and remained unchanged in the other ones (Figure 1B). In the 28 (78%) patients in whom mean arterial pressure (MAP) increased ≥ 15% during norepinephrine changes, MAP increased from 64 (60–70) to 86 (80–103) mmHg and CRT decreased > 23% in 11 patients and remained unchanged in the other ones. At multivariate analysis, the absolute value of CRT at baseline was associated only with arterial lactate at baseline. Still at multivariate analysis, the fluid- and norepinephrine-induced changes in CRT were associated only with the initial value of CRT, but not with the change of the investigated macrohemodynamic variables (CI, MAP, diastolic arterial pressure, heart rate, central venous pressure).

**Conclusion:** In patients with septic shock, the absolute value of CRT is related to arterial lactate but not to macrohaemodynamic variables. Fluid- and norepinephrine-induced changes of CRT are not straightforwardly associated with changes of macrohaemodynamic variables. The precision of CRT measured in a standardized way is poor.

**Compliance with ethics regulations:** Yes in clinical research.

**Figure 1 (abstract FC-197):**
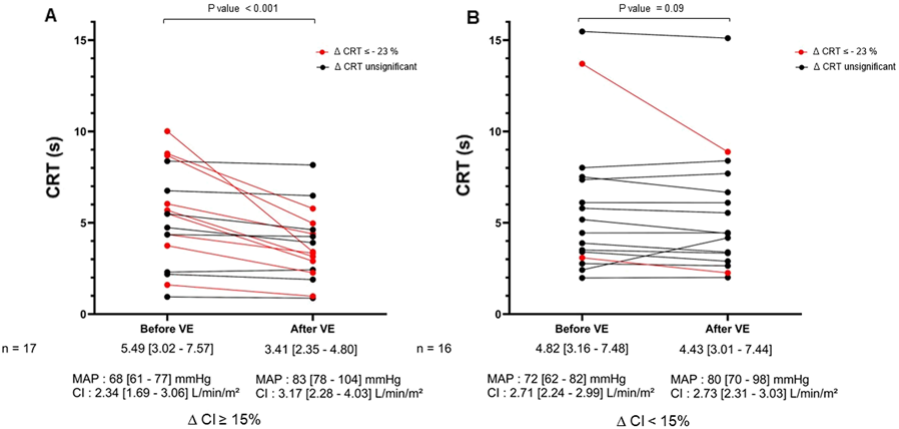
Evolution of capillary refill time in septic shock patients with fluid responsiveness (A) and patients without fluid responsiveness (B)

## FC-198 Association of post-cardiac arrest shock with neurological outcome

### Juliette Didier^1^, Marie Salvetti^1^, François-Xavier Laborne^3^, Stéphane Legriel^1,2,4^, Marine Paul^1,2^

#### ^1^Centre Hospitalier de Versailles - Hôpital André Mignot, Le Chesnay-Rocquencourt, France; ^2^AfterROSC Study Group, Paris, France; ^3^Centre Hospitalier Sud Francilien, Corbeil-Essonnes, France; ^4^University Paris-Saclay, UVSQ, INSERM, CESP, Team “PsyDev”, Villejuif, France

##### **Correspondence:** Marie Salvetti (marie.salvetti@gmail.com)

*Annals of Intensive Care* 2023, **13(Suppl 1):**FC-0198

**Rationale:** Mortality after cardiac arrest (CA) is very high and chiefly due to refractory post-resuscitation shock (PRS) and hypoxic-ischaemic brain injury (HIBI) (1). The severity of PRS could be in line with global anoxia and thus the initial sign of anoxic cerebral damage, on the other hand the severity of the shock could induce secondary cerebral damage. The aim of this study was to explore an association between the intensity of PRS and the progression to severe HIBI.

**Patients and methods/materials and methods:** We performed a retrospective monocentric study in a tertiary CA center. Among all consecutive patients admitted between 2015 and 2020 with a sustained return of spontaneous circulation (ROSC), we excluded patients died of other causes than HIBI and patients with a Cerebral Performance Category (CPC) 3 before CA. Intensity of PRS was assessed by the Vasoactive Inotropic Score (VIS), calculated for the first 3 days (2). An analysis of the association between VIS and CPC score at 3 months (dichotomized into favorable outcome 1–2 versus 3–4–5) was performed by multivariable logistic regression.

**Results:** Among 407 CA patients, 194 were included in the study, with 64.9% of men and a median age of 66 years. The majority of CA were out-of-hospital (66%) and due to shockable rhythm (54.6%). Two-thirds of patients experienced PRS, with a maximum mean VIS value in the first 3 days of 8.1. Outcome at 3 months was considered unfavorable (CPC 3, 4 or 5) in 94 patients, including 75 deaths. VIS was not significantly associated with unfavorable outcome at 3 months in multivariable analysis. Four variables were independently associated with a poor prognosis at 3 months: a cumulative duration of No-flow and Low-flow ≥ 16 min, a non-shockable initial rhythm, the administration of epinephrine before ROSC, and the presence of dysglycemia during the first 3 days. The results of the multivariable analysis are presented in the table below.

**Conclusion:** Intensity of the PRS evaluated with the VIS was not significantly associated with unfavorable outcome at 3 months, but no-flow and low-flow, non-shockable CA, initial epinephrine and dysglycemia were associated with brain damage. Our population consisted mainly of patients with mild hemodynamic failure, which may help to explain the lack of association found between the intensity of post-CA shock and the neurological prognosis at 3 months. Pending a larger prospective study, it seems appropriate not to limit catecholamine doses after CA.

**Reference 1**

Witten L, Gardner R, Holmberg MJ, Wiberg S, Moskowitz A, Mehta S, et al. Reasons for death in patients successfully resuscitated from out-of-hospital and in-hospital cardiac arrest. Resuscitation. 2019;136:93–99.

**Reference 2**

Belletti A, Lerose CC, Zangrillo A, Landoni G. Vasoactive-inotropic score: evolution, clinical utility, and pitfalls. J Cardiothorac Vasc Anesth. 2021;35(10):3067–3077.

**Compliance with ethics regulations:** Yes in clinical research.

**Table 1 (abstract FC-198):**
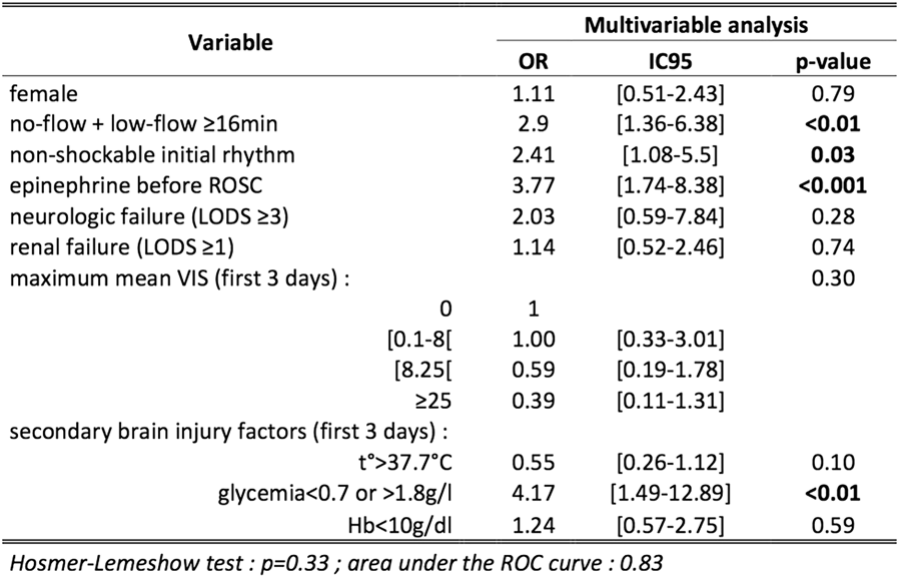
Multivariable analysis of factors associated with poor outcome

## FC-199 Determinants of vascular leakage after cardiac arrest

### Emmanuelle Guérin^1,2^, Alexandre Rutault^2^, Iris Marangon^2^, Cyrielle Desnos^3^, Thomas Frapard^2^, Alain Combes^5^, Pierre-Louis Tharaux^4^, Stéphane Germain^2^, Nicolas Bréchot^1,2^

#### ^1^Hôpital Européen Georges Pompidou, APHP, Paris, France; ^2^INSERM U1050- Collège de France, Paris, France; ^3^Hôpital Tenon, APHP, Paris, France; ^4^INSERM U970-PARCC, Paris, France; ^5^Hôpital Pitié-Salpêtrière, Paris, France

##### **Correspondence:** Nicolas Bréchot (nicolas.brechot@aphp.fr)

*Annals of Intensive Care* 2023, **13(Suppl 1):**FC-0199

**Rationale:** Although vascular leakage is a major feature of systemic inflammatory response syndrome associated with shocks, its molecular mechanisms remain poorly characterized in humans. RNAseq analysis of circulating monocytes, performed in 11 patients after resuscitated cardiac arrest, revealed 860 genes that were differentially expressed between patients with and without massive vascular leakage. Ingenuity® pathway analysis identified clusters of genes related to (i) inflammation, (ii) Hypoxia-Inducible Factor-induced angiogenesis, and (iii) cell cycle regulation. Thirty-eight genes were retained after correction for false discovery rate. Among them, a gene coding for a protein P (protein with an ongoing patent protection), ligand of a receptor involved in vascular biology, had a 56 times higher expression in patients with massive leakage. In accordance, plasma levels of P-PROTEIN were found strongly associated with the level of vascular leak in an independent validation cohort of 52 post-cardiac arrest patients. The objective of the study was to test the hypothesis that P-PROTEIN may play a role during cardiac arrest induced vascular leakage.

**Patients and methods/materials and methods:** We first set up a model of resuscitated cardiac arrest in mice, with a no-flow and a low-flow time of 8 min each. Survival and vascular leakage were then compared after cardiac arrest between P-protein knock-out (KO), wild-type (WT) mice, and WT mice injected with recombinant mouse P-PROTEIN (rmP-PROTEIN) at the time of resuscitation (after the no-flow). Vascular leakage was compared between conditions using i.v. injected fluorescent dextrans.

**Results:** We confirmed an important vascular leak in all organs after the return of spontaneous circulation (ROSC) in WT mice, and an induction of circulating P-PROTEIN levels at one-hour post-ROSC. Intra-venous injection of rmP-PROTEIN significantly reduced the vascular leakage quantified by extravasation of fluorescent dextrans. Survival was also significantly affected by modulating P-PROTEIN activity. We demonstrated a beneficial effect of rmP-PROTEIN injection, with 88% of ROSC in WT mice injected with rmP-PROTEIN vs. 67% in controls and an improved survival in rmP-PROTEIN injected mice vs controls (Figure 1). The proportion of mice achieving a ROSC was on the contrary strongly reduced in P-protein KO mice compared to their WT littermates (10%, p < 0.0001 vs. WT).

**Conclusion:** These results demonstrate that the P-PROTEIN, isolated from observations in humans, is a key regulator of post-cardiac arrest vascular leakage, with beneficial effects of P-PROTEIN gain of function in mice. Further experiments are ongoing to characterize its mechanisms of action on vascular leakage, as well as its optimal dosing regimen in a pre-clinical setting.

**Compliance with ethics regulations:** Yes in clinical research.

**Figure 1 (abstract FC-199):**
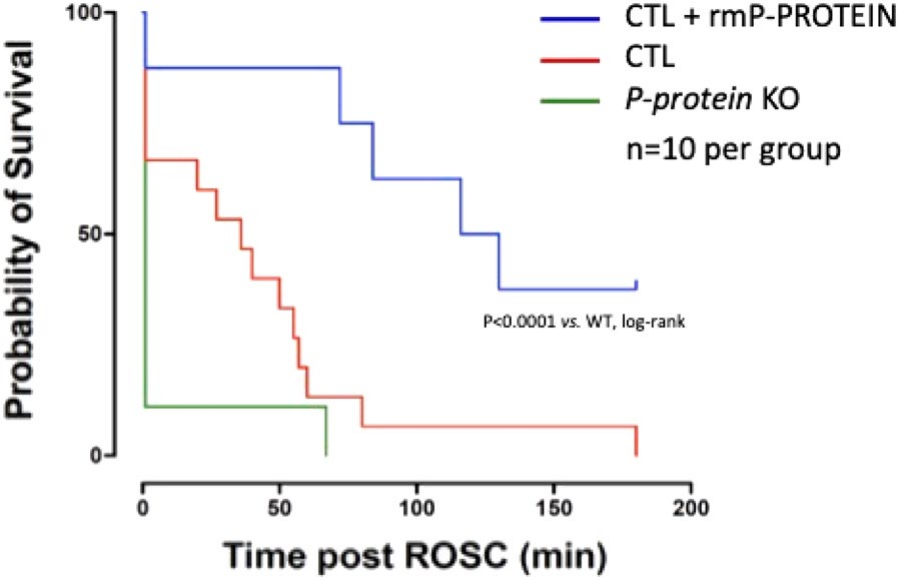
Survival after cardiac arrest of wild-type mice, p-protein KO mice, and wild type mice injected with recombinant mouse P-PROTEIN at the time of resuscitation

## FC-200 Neuron-specific enolase prognostic value according to EEG patterns following cardiac arrest

### Juliette Pelle^1^, Estelle Pruvost-Robieux^2^, Florence Dumas^3^, Julien Charpentier^1^, Alain Cariou^1^, Sarah Benghanem^1^

#### ^1^Médecine Intensive Réanimation, Université Paris Cité. APHP. Paris Centre site Cochin, Paris, France; ^2^Laboratoire de Neurophysiologie, GHU Neurosciences Sainte-Anne, Paris, France; ^3^Service d'accueil des Urgences, Université Paris Cité. APHP. Paris Centre site Cochin, Paris, France

##### **Correspondence:** Juliette Pelle (juliette.pelle@aphp.fr)

*Annals of Intensive Care* 2023, **13(Suppl 1):**FC-0200

**Rationale:** Approximatively 50% of patients remain comatose 72 h after cardiac arrest (CA). The prognostication algorithm proposed by the ESICM highlighted 6 robust criteria for poor outcome prediction, including “highly malignant” electroencephalogram (EEG) and Neuron-Specific Enolase (NSE) level > 60 µg/l at 48-72 h after CA (1). The prognostic value of “malignant” EEG and particularly of electrographic seizure remains controversial. Moreover, serum NSE is known to be sensible to epileptic activity. We hypothesized that the NSE level could be modified by EEG-detected epileptic features after CA, and that NSE cut-off for poor outcome prediction could be adapted according to EEG pattern.

**Patients and methods/materials and methods:** A retrospective study based on a prospective registry was conducted between 2017 and 2021 in our Cardiac Arrest center. Data were collected from adult patients admitted alive after CA, remaining comatose at 72 h. CA from neurological and traumatic causes were the exclusion criteria. EEG and serum NSE level at 72 h were required. An expert neurophysiologist retrospectively assessed EEG pattern according to Westhall et al. (2) as “highly malignant”, “malignant” and “benign” EEG. The primary outcome was the CPC score at 3 months, CPC1-2 being considered as good neurological outcome.

**Results:** One hundred and sixty patients were included. They were 63.5 years old [IQR = 9.5], and 73% were male. 80% were out-of-hospital CA and 47% were initial shockable rhythm. Among patients with good neurologic outcome, 87% had a benign pattern, 13% a malignant pattern and none presented highly malignant EEG. Regarding the poor outcome group, 17% had a benign pattern, 41% a malignant and 41% a highly malignant EEG (p < 0.001). We observed a low rate of seizure or status epilepticus (5% and 8% in good and poor outcome, respectively). NSE levels were significantly lower in the good outcome (median 20 µg/l [15.8; 28]) compared to poor outcome group (median 109 µg/l [50.3; 307.3], p < 0.001). Benign EEG patterns were associated with lower levels of NSE compared to malignant and highly malignant patterns (p < 0.001). The presence of electrographic seizures or status epilepticus was not significantly associated with an increase in NSE level (p = 0.52).

**Conclusion:** In comatose patients after CA, NSE was congruent to EEG patterns, emphasizing the correlation between biological and neurophysiological markers of brain injury. We did not find a significant increase of NSE levels in case of EEG epileptic features. This suggests that seizure could be a limited factor of secondary brain injury.

**Reference 1**

Nolan JP, Sandroni C, Böttiger BW, et al.: European Resuscitation Council and European Society of Intensive Care Medicine guidelines 2021: post-resuscitation care. Intensive Care Med 2021; 47:369–421.

**Reference 2**

Westhall E, Rossetti AO, van Rootselaar A-F, et al.: Standardized EEG interpretation accurately predicts prognosis after cardiac arrest. Neurology 2016; 86:1482–1490.

**Compliance with ethics regulations:** Yes in clinical research.

## FC-201 Benefits of cardiopulmonary resuscitation in cancer patients

### Marie Geelhand De Merxem^1^, Lieveke Ameye ^1^, Anne-Pascale Meert^1^

#### ^1^Institut Jules Bordet, Université Libre de Bruxelles, Bruxelles, Belgique

##### **Correspondence:** Marie Geelhand De Merxem (marie.geelhand.de.merxem@ulb.be)

*Annals of Intensive Care* 2023, **13(Suppl 1):**FC-0201

**Rationale:** According to meta-analytic data, the prognosis of a cancer patient post- cardiopulmonary resuscitation (CPR) is relatively similar to the general population. However, preselection of patients, the details of CPR, patient-specific characteristics, and post-CPR care are poorly described. The aim of this study is to identify prognostic factors in order to recognize cancer patient profiles more likely to benefit from CPR.

**Patients and methods/materials and methods:** This is a retrospective study on a series of patients with solid or hematological malignancies who received CPR between January 2010 and December 2020 in a cancer institute.

**Results:** Sixty-eight patients were included. The ratio of solid to hematological malignancy was 44/24, of which 32 were metastatic solid tumors. Median age was 61 years. Hypoxemia (29%) was the primary factor for cardiac arrest, followed by septic shock (21%). ICU mortality and hospital mortality were 87% and 88% respectively. Younger age, the presence of hematological malignancy or a metastatic solid tumor were predictors of poor outcome for in-hospital mortality. Similarly, cardiac arrest in the ICU, as the final consequence of a pathological process, and a resuscitation time of more than 10 min have a negative influence on prognosis.

**Conclusion:** This study shows that CPR is a useful intervention in cancer patients, even in the elderly patient, especially in non-metastatic solid tumors when cardiac arrest is the cause of an acute event and not a terminal process.

**Compliance with ethics regulations:** Yes in clinical research.

## FC-202 Return to work after out of hospital cardiac arrest, insights from a prospective multicentric French cohort

### Nolwen Flajoliet^1^, Jérémy Bourenne^2^, Nathalie Marin^3^, Jonathan Chelly^4^, Jean-Baptiste Lascarrou ^5^, Cédric Daubin^6^, Wulfran Bougouin^7^, Alain Cariou^3^, Guillaume Geri^8^

#### ^1^APHP, Paris, France; ^2^CHU La Timone 2, Marseille, France; ^3^CHU Cochin, Paris, France; ^4^CHITS, Toulon, France; ^5^CHU Nantes, Nantes, France; ^6^CHU Caen, Caen, France; ^7^Hôpital Privé Jacques Cartier, Massy-Palaiseau, France; ^8^Clinique Ambroise Paré, Neuilly-Sur-Seine, France

##### **Correspondence:** Nolwen Flajoliet (nolwen.flajoliet@hotmail.fr)

*Annals of Intensive Care* 2023, **13(Suppl 1):**FC-0202

**Rationale:** The evaluation of long-term prognosis of cardiac arrest (CA) survivors is challenging and includes multiple aspects from daily-life autonomy to health-related quality of life as well as social interactions. Return to work assessment has recently been situated at the crossroads of quality of life, social life as well as neurological recovery. About 60 to 70% of CA survivors who worked before CA return to work within 1 year. The objective of this study is to assess components of return to work in details in CA survivors.

**Patients and methods/materials and methods:** We included patients included between April 1st 2021 and March 31st 2022 in the French national multicentric cohort AfterRosc, discharged alive from the hospital and less than 65 years old. Patients retired or on sick leave were not included in the analysis. A phone-call interview was conducted 1 year after CA to assess return to work, level of education, former level of occupation (both evaluated using the World Health Organization ISCED and ISCO questionnaires) as well as neurological recovery using the MPAI-4 (Mayo-Portland Ability Inventory) form. We also collected geographic and socio-economic data. Comparisons were performed between patients who returned to work and those who did not.

**Results:** Among 86 patients from 7 centers, 54 patients were contacted, and 31 patients were finally included in the analysis. Seventeen survivors returned to work after a median delay of 112 days (Q1Q3 92–157). Out of these 17, 9 (53%) needed work adjustments such as part-time work or remote working. Out of the 47% (n = 8) that didn’t have official work adjustments, 50% (n = 4) worked less hours, and one had to switch to a therapeutical part time job after a few weeks. Higher educational level, type of job, better socio-economical level, as well as higher scores on all three components of the MPAI-4 score (abilities, adjustment and participation) were significantly associated with return to work. Median monthly income for all participants notably decreased after CA, from 2400 euros (Q1Q3 1712–3152) to 1775 euros (Q1Q3 1050–2077) (p < 0.05).

**Conclusion:** Return to work in French CA survivors was associated with better socio-economical status, as well as better scores on all MPAI-4 components. Further research is needed to better characterize CA survivors who would benefit from focused interventions to make return to social and professional life easier.

**Compliance with ethics regulations:** Yes in clinical research.

## FC-203 Use of anti-hypertensive drugs in intensive care units: a prospective observational study. The CUICUI-AHD study

### Charles Verney^2^, Abirami Thiagarajah^5^, Gaëtan Plantefeve^7^, Nicolas Bonnet^3^, Laura Federici^2^, Yonatan Perez^1^, Matthieu Lemeur^6^, Constance Vuillard^2^, Arnaud Le Flécher^4^, Lucie Le Fevre^4^, Pierre-Antoine Billet^4^, Stephan Ehrmann^1^, Romain Sonneville^4^, Charles Bokobza^4^, Clément Massonnaud^4^, Damien Roux^2^, Jean-Damien Ricard^2^, Stéphane Gaudry^3^

#### ^1^CHRU de Tours, Tours, France; ^2^Hôpital Louis Mourier - APHP, Colombes, France; ^3^Hôpital Avicenne - APHP, Bobigny, France; ^4^Hôpital Bichat - APHP, Paris, France; ^5^Hôpital René Dubos, Pontoise, France; ^6^Groupe hospitalier Nord Essonne, site Longjumeau, Longjumeau, France; ^7^Hôpital Victor Dupouy, Argenteuil, France

##### **Correspondence:** Charles Verney (charlesverney@gmail.com)

*Annals of Intensive Care* 2023, **13(Suppl 1):**FC-0203

**Rationale:** Except in the context of hypertensive crisis, data are lacking on the use of antihypertensive drugs (AHD) in intensive care units (ICU). Non urgent hypertension is however a frequent situation in ICU. We aimed at assessing the current therapeutical management of such hypertension in ICU patients having experienced hemodynamic shock.

**Patients and methods/materials and methods:** We conducted a prospective study in seven French ICUs including patients aged of 18 years old or more who received at least one AHD following a circulatory shock that required epinephrine or norepinephrine infusion. The main objective of the study was to describe the strategies in AHD prescription and the reasons of their choice. Second objectives were to compare the efficacy of the AHD and their tolerance.

**Results:** From July 2019 to December 2021, 179 patients were enrolled. Median age was 65 years and IGSII at admission was 46 (Interquartile range [IQR] 39–58). At the time of first AHD prescription, the median systolic blood pressure was 170 mmHg (IQR 158–181) and the median diastolic blood pressure was 81 mmHg (IQR 70–91). The most frequently prescribed AHD were calcium channel blockers (38%) following by alpha-blockers (19%), renin angiotensin system inhibitors (16%), central acting antihypertensive agents (11%) and beta-blockers (6%). We found a large heterogeneity in AHD prescriptions across the units (p < 0.0001) (Figure 1). The blood pressure target was defined as inferior to 140/90 mmHg for 81% of the patients and 73% of AHD were administered with continuous intravenous perfusion. The first line of AHD led to blood pressure control in 76% of the cases and led to adverse side effect in 11% of the cases in the first 72 h following their initiation. We did not find any differences in efficacy or safety in the different prescription strategies, regarding the drugs, their class, administration modalities or combination therapies. The main motivations for the choice of prescription strategies were unit habits (79%) and personal believes (66%), whereas price was considered in only 20% of the prescriptions.

**Conclusion:** This study shows a large heterogeneity in AHD prescription for non-urgent hypertension following circulatory shock in ICU. The most prescribed AHD were calcium channel blockers, followed by alpha-blockers and renin angiotensin system inhibitors. The choice of AHD was essentially based on habits of the intensivists and units. This confirms the need to conduct interventional studies to better define the place of each AHD in ICU.

**Compliance with ethics regulations:** Yes in clinical research.

**Figure 1 (abstract FC-203):**
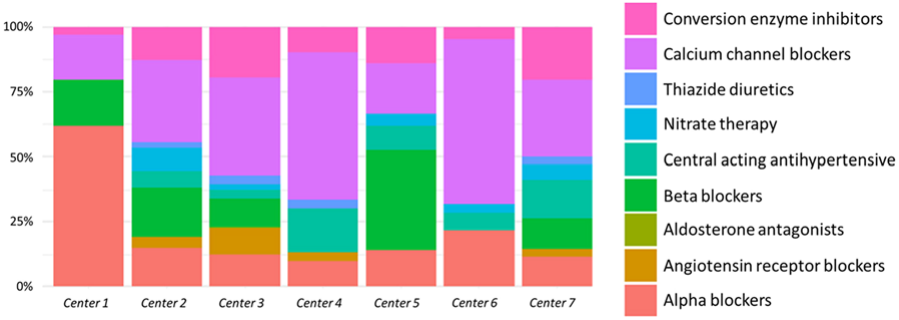
Proportion of therapeutic class prescribed across the participating centers

## FC-204 Acute renal failure in septic shock: interest of the renal resistance index

### Rabeb Hammami^1^, Mahmoud Marzouk^1^, Sabeur Thamlaoui^1^, Maryem Ben Amor^1^, Nader Baffoun^1^, Chokri Kaddour^1^

#### ^1^Institut National De Neurologie, Tunis, Tunisie

##### **Correspondence:** Rabeb Hammami (hammamirabeb2@gmail.com)

*Annals of Intensive Care* 2023, **13(Suppl 1):**FC-0204

**Rationale:** The diagnosis of acute renal failure in septic shock is based on the measurement of creatinine and urine output. Ultrasound measurement of the renal resistance index could be a predictor of its occurrence. The aim of this study was to investigate the correlation between the renal resistance index (RRI) and renal function and to describe the variability of the RRI as well as the variability of the different hemodynamic and biological parameters.

**Patients and methods/materials and methods:** This was a prospective observational study conducted over a period of 6 months. Patients aged superior to 18 years of age with septic shock were included. Patients with chronic kidney disease, cardiac arrhythmias, chronic liver disease, urinary tract obstruction, or surgical abdomen were excluded. Patients were included from the first 24 h of septic shock. During the first 5 days, measurements of renal resistance index, urine flow renal resistance index, urine output, renal function, hemodynamic parameters, and norepinephrine output.

**Results:** Twenty patients were selected for the study. The sex ratio was 2.33. The average age was 53 ± 11.91 years. The history was dominated by hypertension in 35% of patients. The main reason for admission was stroke in 55% of patients. The mean SOFA score was 6.26 ± 4.05. The mean time to onset of septic shock was 12.39 ± 11.57 days. The causative organisms were mainly BGN in 80% of cases. The starting point was the lung in 65% of cases. The peak of the IRR was noted on day 1 with a mean of 0.75 ± 0.07. The peaks of the other parameters collected were noted on day 1 for the following parameters: urea (8.68 mmol/l ± 8.13) and norepinephrine dose (0.51 gamma/kg/min ± 0.35). Peak creatinine was noted on the second day (133.68 μmol/l ± 166.26). The lowest values were noted on day 1 for pH (7.35 ± 0.1), urine output (1.22 cc/kg/h ± 0.64), HCO3- (22.63 meq/l ± 4.66), ScVO_2_ (66.66% ± 14.12) and MAP (81.26 mmHg ± 11.72). Seven patients had an acute renal failure (ARF) or 35% according to KDIGO diagnostic criteria. The RRI measured at D1 and D2 correlated significantly with the occurrence of acute renal failure (Table 1). The mean duration of mechanical ventilation was 30.47 ± 20.18 days. The average length of stay in intensive care was 33.47 days ± 18.29. The mortality rate was 55%.

**Conclusion:** RRI measured at D1 and D2 significantly predicts the occurrence of AKI. Large-scale studies are needed to validate this ultrasound tool. This will help to strengthen early renal protection measures.

**Compliance with ethics regulations:** Yes in clinical research.

## FC-205 Attempted suicide among prisoners in intensive care unit: comparison of patients with or without mental disorders

### Hassen Ben Ghezala^1^, Boudour Ben Dhia^1^, Amira Ben Jazia^1^, Nozha Brahmi^1^

#### ^1^Centre Mahmoud Yaacoub d'assistance médicale urgente de Tunis, Tunis, Tunisie

##### **Correspondence:** Hassen Ben Ghezala (hassen.ghezala@gmail.com)

*Annals of Intensive Care* 2023, **13(Suppl 1):**FC-0205

**Rationale:** Suicide is a severe global cause of death and a psychiatric emergency. Among prisoners, it is the leading cause of death compared to the general population. But suicide in prison is a neglected public health issue. Does the psychiatric history (PH) have a correlation with this phenomenon?

**Patients and methods/materials and methods:** A 25 months (from January 2020 to January 2023) retrospective analytic observational single centre study included all detainees admitted to ICU for management of intoxication. The cohort was divided in 2 groups (group A: patients with PH and group B: patients without PH).

**Results:** Of the sixty patients enrolled, seventeen patients (28%) had a PH: 24% depression, 6% bipolar disorder and in 70% PH was unspecified. The mean age of the population was 35 ± 9 years with male predominance (gender ratio = 0.063). There was no significant difference between the two groups in gender, socioeconomic conditions, marital status, and type of intoxication. Having a history of psychiatric disorder is correlated with a history of suicide attempts (P = 0.036). No one of the body packers had a psychiatric disorder. The table below showed the clinical characteristics. Having PH was a risk factor for recurrence (P = 0.007). Severity of the initial clinical presentation (GCS, respiratory failure and hemodynamic failure) were comparable in the two groups. Multidrug intoxication was present in 56%: for group A this intoxication was associated with more than two drugs P = 0.004 Supposed ingested doses were higher for the most used drugs for group A: metformin and sulfonamide with P respectively 0.032; 0.041. The use of mechanical ventilation was comparable (P = 0.599) between the two groups, but group A had longer duration of mechanical ventilation P = 0.012 and withdrawal syndrome P = 0.046. The need for hemodialysis was comparable between the two groups P = 0.701. The duration of hospitalization was longer for group A 6 days versus 3 days, P = 0.023. Multivariate analysis showed that psychiatric disorders were an independent risk factor for recurrence of suicide attempt P = 0.025 RR 3.991 IC95% [1.190; 13.38]. In our study one death was reported in the first group in the context chemical ingestion.

**Conclusion:** More research is required to improve the prediction of suicide among inmates with PH, as well as the more effective implementation of preventive measures.

**Compliance with ethics regulations:** Yes in clinical research.

**Table 1 (abstract FC-205):**
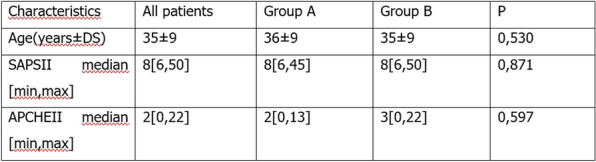
Clinical characteristics of prisoners

## FC-206 No evidence of a July effect in a low-middle income country medical intensive care unit. A retrospective cohort analysis

### Radhouane Toumi^1,2^, Khaoula Meddeb^1,2^, Nabil Bouguezzi^1^, Amir Bedhiafi^1^, Rym Chelbi^1^, Imen Belhouchet^1^, Imen Ben Saida^1,2^, Mohamed Boussarsar^1,2^

#### ^1^Medical Intensive Care Unit, Farhat Hached University Hospital, Sousse, Tunisie; ^2^Research Laboratory N° LR12SP09. Heart Failure. Farhat Hached University Hospital, Sousse, Tunisie

##### **Correspondence:** Radhouane Toumi (Radhouane.toumi@gmail.com)

*Annals of Intensive Care* 2023, **13(Suppl 1):**FC-0206

**Rationale:** Each year, teaching hospitals receive freshly-graduated doctors commencing their professional life and quasi-immediately given patient-related responsibilities. Previous studies have stated that patients in teaching hospitals are at higher risk of morbidity and mortality at the start of the academic year when newly-graduated young doctors arrive. This phenomenon is known as “The July Effect”. Critically-ill patients represent a particular group of patients already at high risk of mortality. The aim of the current study is to determine whether critically-ill patients are at higher risk of mortality during the first month of the academic year compared to the last month in a medical intensive care unit (ICU) of a teaching hospital in a low-middle income North-African country.

**Patients and methods/materials and methods:** This is a monocentric retrospective cohort analysis conducted in the medical ICU of a University Hospital in a low-middle income country over a 20-year period, from 2001 to 2020, including all consecutive patients admitted during January and December which are, consecutively, the first and last months of the academic year. Collected data included age, severity assessed by the simplified acute physiology score II (SAPS II), use and duration of invasive mechanical ventilation (IMV), ICU-length of stay (ICU-LOS) and ICU-mortality.

**Results:** A total of 990 patients were included in the final analysis. There were no significant differences between patient severity or outcomes between patients admitted in January and those admitted in December with SAPS II, (median IQR) [30 (21–42) vs 30 (20–42), p = 0.415]; IMV, [402 (75.4%) vs 320 (70%), p = 0.057]; duration of IMV, [4 (2–9) vs 4 (2–10), p = 0.273]; IMV mortality, [191 (47.5%) vs 149 (46.6%), p = 0.799], ICU-LOS [6 (3–12) vs 6 (3–11), p = 0.5] and ICU-mortality [221 (41.5%) vs 178 (38.9%), p = 0.421].

**Conclusion:** There was no equivalent of a July Effect in the MICU of a teaching hospital in a low-middle income country. Further studies are necessary for the sake of generalizability and for more insight on the impact of newly-arrived physicians-in-training.

**Compliance with ethics regulations:** Yes in clinical research.

## FC-207 Epidemiology of intensive care unit-acquired infections at the Centre Hospitalier Universitaire de Libreville

### Laurence Essola^1^, Arsène Ifoudji Makao^1^, Luc Bitégué Methe^1^, Fernande Manga^1^, Aurélie Baderwha^1^, Adrien Sima Zue^1^

#### ^1^Centre hospitalier universitaire de Libreville, Libreville, Gabon

##### **Correspondence:** Laurence Essola (laurenceessola@yahoo.fr)

*Annals of Intensive Care* 2023, **13(Suppl 1):**FC-0207

**Rationale:** Nosocomial infections (NI) or healthcare-related infections constitute a major public health problem. The aim of this work was to carry out an inventory of INs in intensive care at the CHUL in order to determine the causative microorganisms.

**Patients and methods/materials and methods:** This was a prospective, observational study from January to August 2021. All patients who stayed more than 48 h in intensive care and who developed an IN were included. The parameters studied on admission were socio-demographic, clinical and paraclinical data, the pathologies selected, initial antibiotic therapy and invasive procedures. From the onset of an IN, the parameters studied were temperature, biological (blood count and reactive protein C), microbiological and radiological data.

**Results:** One hundred and seventy patients had a length of stay greater than 48 h in intensive care. 26 (15.3%) of them contracted at least one NI. The main reasons for admission were medical conditions (88.5%). The mean time to onset of fever was 7.1 ± 5.4 days. Forty-three samples were taken, including seventeen cytobacteriological examinations of the urine (ECBU). Culture allowed forty-three microorganisms to be isolated: forty bacteria (93%) and three yeasts (7%). Of the bacteria, twenty-six (65%) were gram negative bacilli (BGN) and fourteen (35%) gram positive cocci (CGP). The main bacteria isolated were Staphylococcus aureus (27.9%) and Klebsiella pneunoniæ (23.3%). The yeasts were of the genus Candida. Thirty-one INs were found including twelve urinary tract infections (38.7%) and ten bacteremia (32.3%).

**Conclusion:** The most frequently observed INs in intensive care are urinary tract infections. The main germs isolated are BGNs at the head of which Klebsiella pneunoniæ. As the transmission of germs is mainly carried out by hand, the organization of training for healthcare staff on hand hygiene is essential.

**Compliance with ethics regulations:** Yes in clinical research.

## FC-208 Diabetic in intensive care: experience of the resuscitation unit from the medical emergency department of the HUC of Oran, Algeria

### Nabil Sidi Aissa^1^, Mourad Goulmane^1^, Khadidja Reziga^1^, Nabil Tabet Aoul^1^

#### ^1^CHU Oran, Oran, Algerie

##### **Correspondence:** Nabil Sidi Aissa (sidiaissa_nabil@yahoo.fr)

*Annals of Intensive Care* 2023, **13(Suppl 1):**FC-0208

**Rationale:** Acute metabolic complications of diabetes are a frequent reason for admission to intensive care. Diabetes mellitus, a silent disease, is becoming more and more frequent in the Maghreb countries with sometimes discovery fortuitous during an acute complication such as an ketoacidotic coma.

**Patients and methods/materials and methods:** AThis was an analytical study of the acute metabolic complications of diabetes mellitus in the intensive care unit of the Oran University Hospital Center. Retrospective study over a period of 1 year (January 1, 2022 to December 31, 2022). Population studied corresponds to patients admitted to our department for complicated diabetes mellitus.

**Results:** Eighty patients were admitted with an admission frequency of 8.3% with an average age of 54 ± 15 years and a male predominance (sex-ratio = 1.06). The main reasons for admission were severe coma (72.1%), respiratory distress (67.8%) and dehydration (34.1%). Ketoacidosis constituted 61.1% followed by hypoglycemia (27.3%) and hyperosmolar hyperglycemia syndrome (HHS) (11.6%). Mean hyperglycaemia was 28.41 mmol/L and average hypoglycaemia of 1.4 ± 0.8 mmol/L. Overall mortality (47.15%).

**Conclusion:** Acute complications of diabetes are frequent reasons for admission to intensive care with a rate considerable mortality, the latter could be avoided by early and appropriate management of diabetes.

**Compliance with ethics regulations:** Yes in clinical research.

## FC-209 0.9% Sodium chloride versus Ringer’s lactate in the treatment of severe diabetic ketoacidosis: a randomized trial

### Ahlem Trifi^1^, Salma Ghalloussi^1^, Hounaida Galai^1^, Asma Mehdi^1^, Lynda Masseoud^1^, Eya Seghir^1^, Badis Tlili^1^, Emir Bedhiafi^1^, Asma Ouhibi^1^, Sami Abdellatif^1^, Salah Ben Lakhal^1^

#### ^1^Medical ICU, Hôpital la Rabta, Tunis, Tunisie

##### **Correspondence:** Ahlem Trifi (trifiahlem2@gmail.com)

*Annals of Intensive Care* 2023, **13(Suppl 1):**FC-0209

**Rationale:** Severe diabetic ketoacidosis (DKA) is a potentially serious complication of diabetes mellitus. The treatment regimen is based on insulin and rehydration. The choice of rehydration solution is a question that remains open. We sought to compare the effect of sodium chloride 0.9% (SC) versus ringer lactate (RL) in the resolution of severe DKA as well as on the variation of electrolytes.

**Patients and methods/materials and methods:** The study was an open randomized trial in adult patients admitted to an intensive care unit (ICU) for severe DKA. The insulin therapy protocol was identical and the randomization concerned the rehydration solution either by SC or RL. The primary endpoint was resolution of DKA at H48 defined by a composite endpoint (glycemia < 11 mmol/l, bicarbonates > 15 mmol/l or pH > 7.30 and anion gap < 16). The secondary endpoints were resolution of DKA at H24, change in base excess to superior or equal to − 3 meq/L at 48 h and H24 and change in electrolytes, insulin requirements, length of stay and mortality. Blood gases, ionogram with chloride and lactate were performed at baseline, H6, H12, H24 and H48. Ethics committee approval and participant consent was obtained.

**Results:** 41 patients were included: SC arm (n = 20) and RL arm (n = 21). Baseline clinical characteristics were similar between groups with an overall female predominance. Similarly, the basic biological parameters (glycaemia, pH, bicarbonates, BE, electrolytes, lactates, anion gap) were comparable. No difference was highlighted for the primary and secondary endpoints apart from a more prolonged ICU-LOS in the RL group (attached table). Concerning the monitoring of the parameters over the four times of the study, it was observed that at H12, the level of bicarbonates (17 vs 13, p = 0.08) and BE (− 9.6 vs − 13, p = 0.09) tended to be higher in the RL group. Also, at H48 the natremia and chloremia were higher in the SC group but without reaching statistical significance.

**Conclusion:** The use of RL did not lead to a faster resolution of diabetic ketoacidosis but seems to better improve the base excess rate with a lower risk of hypernatremia and hyperchloremia.

**Compliance with ethics regulations:** Yes in clinical research.

**Table 1 (abstract FC-209):**
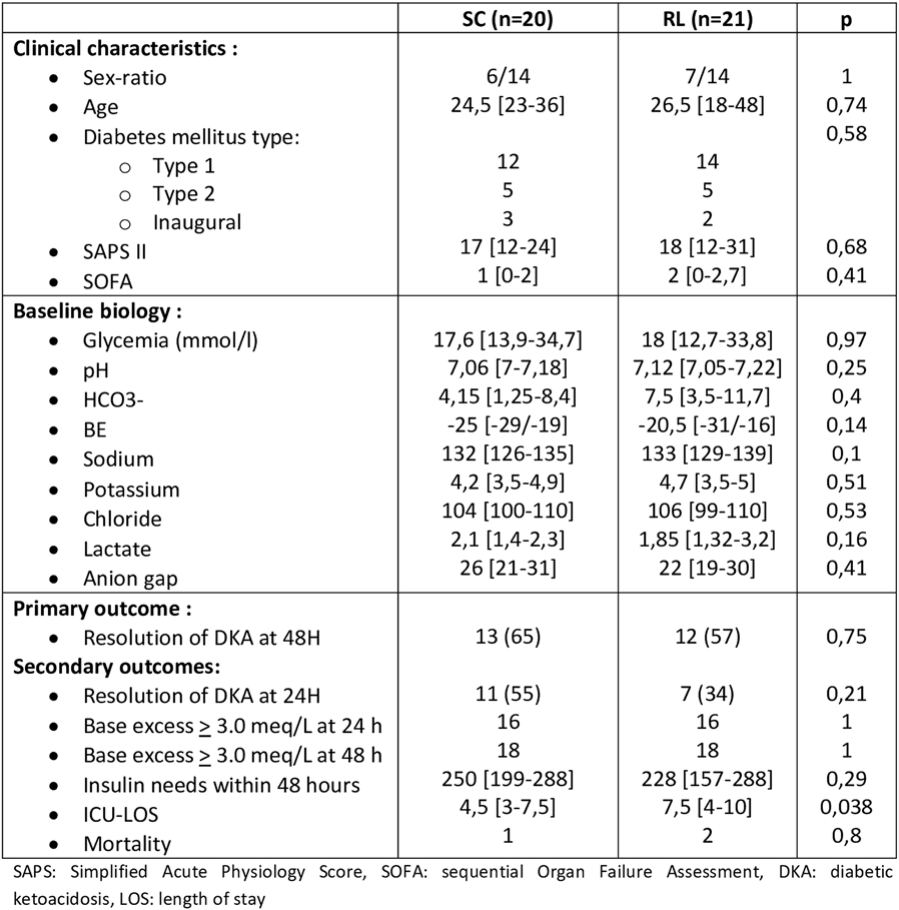
Comparison of clinical characteristics and outcome parameters between the study groups

## FC-210 Anaphylaxis in intensive care: a 15 years case series retrospective study

### Mariem Cheikhrouhou^1^, Hassen Ben Ghezala^1^, Amira Ben Jazia^1^, Nozha Brahmi^1^

#### ^1^Centre Mahmoud Yaacoub d'assistance médicale urgente de Tunis, Tunis, Tunisie

##### **Correspondence:** Hassen Ben Ghezala (hassen.ghezala@gmail.com)

*Annals of Intensive Care* 2023, **13(Suppl 1):**FC-0210

**Rationale:** Anaphylaxis is often a serious health problem. Several allergens are incriminated, often unrecognized, these immediate hypersensitivity reactions can be life threatening. The objective of our study is to determine the epidemiological, clinical and outcomes of anaphylaxis.

**Patients and methods/materials and methods:** Retrospective descriptive single-center study conducted over a period of 15 years (2007–2022) that included all adult patients admitted to the toxicology critical care unit for anaphylaxis.

**Results:** During this period, 148 patients were enrolled in the study. The mean age was 42 ± 16 years. The sex ratio was 1.3 with a female predominance. Drug intoxication was involved in 53.4% (N = 79): penicillin was incriminated in thirty-one cases (20.9%) followed by NSAIDs in ten cases (6.7%). The other allergens involved were hymenopter stings in thirty-six cases (24.3%) and food in twenty-seven cases (18%): tuna 6.7% (N = 10) and fruit 4% (N = 6). Thirty-three patients had a history of allergy (22%) Allergic reactions were anaphylaxis in ninety-eight patients (66.2%) and anaphylactic shock in fifty patients (33.8%), of which forty-four were grade 2 (29.7%), forty-two were grade 3 (28.4%) and two were grade 4 (1.4%). The average consultation time was 8 h [1, 120]: 50.7% (N = 75) of patients presented with urticaria, 17.5% (N = 26) had dyspnea, 16.2% (N = 24) had facial edema and only one patient presented with cardiorespiratory arrest. Generalized urticaria was present in 65.5% of cases (N = 97), respiratory signs were noted in fifty-nine patients (39.4%), the majority of which were dyspnea, and only twenty-seven patients (18.2%) had laryngeal edema. Fifty patients (33.7%) presented a state of septic shock, three patients (2%) were intubated and ventilated for coma, two patients (1.3%) presented a status epilepticus. The management plan included: volume expansion in sixty cases (40.5%), use of adrenaline in fifty-six cases (36.8%), administration of steroids in 134 cases (90.4%) and antihistamines in 126 cases (85.1%). The average length of stay was 27.6 h [6, 240]. The outcome was favorable in the majority of patients, only one case of death was registered.

**Conclusion:** Anaphylactic reactions are frequent, of drug origin in most cases. It is a serious pathology, but if treated in time, the prognosis can be transformed.

**Compliance with ethics regulations:** Yes in clinical research.

## FC-211 COVID-19 among undocumented migrants admitted to French intensive care units during the 2020–2021 period: a retrospective nationwide study

### Sami Hraiech^1,3^, Vanessa Pauly^3^, Laurent Papazian^4^, Elie Azoulay^2^, Laurent Boyer^3^

#### ^1^APHM CHU Nord, Marseille, France; ^2^APHP, Paris, France; ^3^Aix-Marseille Université, Health Service Research and Quality of Life Cente (CEReSS), Marseille, France; ^4^CH Bastia, Bastia, France

##### **Correspondence:** Sami Hraiech (sami.hraiech@ap-hm.fr)

*Annals of Intensive Care* 2023, **13(Suppl 1):**FC-0211

**Rationale:** Before the Coronavirus-Disease 2019 (COVID-19) pandemic, in France, undocumented migrants had a higher risk than general population for being admitted to the ICU because of acute respiratory failure or severe infection. We aimed to analyze the impact of COVID-19 during the first months of pandemic focusing on critically ill undocumented migrants.

**Patients and methods/materials and methods:** All the adult patients admitted to French hospitals during the study period (March 2020 to May 2021) were identified using the French nationwide hospital information system (Programme de Médicalisation des Systèmes d’Information). We retrospectively included all undocumented adult migrants admitted in French hospitals and ICUs in 2020–2021. We then specifically focused on admissions related to COVID-19. Undocumented migrants were compared to the general ICU population, first in crude analysis, then after matching on age, severity and main comorbidities. Our primary objective was to compare the rate of ICU admission for COVID-19 among undocumented migrants and general population. Secondary outcomes were the severity of the respiratory disease, as reflected by the rate of acute respiratory distress syndrome (ARDS), need for invasive mechanical ventilation and mortality rate.

**Results:** During the study period, the rate of ICU admission among patients hospitalized for COVID-19 was higher for undocumented migrants than for general population (463/1627 (28.5%) vs. 81 813/344 001 (23.8%); p < 0.001). Migrants were more frequently admitted directly to the ICU and exhibited a shorter delay from hospital to ICU admission. In the matched analysis, the odds ratio for ARDS during the ICU stay among undocumented migrants was 1248 (1055–1478; p = 0.01). The need for invasive MV was also higher (odds ratio 1196 (1006–1421); p = 0.04) for migrants whereas general population had a more frequent recourse to NIV (29.6% vs. 22.6%; p < 0.001). After matching, there was no difference between migrants and general population concerning outcomes, in particular, ICU and hospital mortality rates. Palliative care was less frequent among migrants.

**Conclusion:** During the first waves of COVID-19 in France, undocumented migrants were more frequently admitted to the ICU and were at higher risk for needing invasive mechanical ventilation and developing ARDS. The mortality rate was similar to that of the general population. These results highlight the need for reinforcing prevention and improving primary healthcare access for people in irregular situation.

**Compliance with ethics regulations:** Yes in clinical research.

## FC-212 Limitation of life-sustaining therapies in critically ill patients with COVID-19: a descriptive epidemiological investigation from the COVID-ICU study

### Mikhael Giabicani^1^, Christophe Le Terrier^2^, Antoine Poncet^2^, Bertrand Guidet^3^, Jean-Philippe Riagud^4^, Jean-Pierre Quenot^5^, Marie-France Mamzer^6^, Jérôme Pugin^2^, Emmanuel Weiss^1^, Simon Bourcier^2^

#### ^1^Hôpital Beaujon AP-HP, Clichy, France; ^2^Geneva University Hospitals, Genève, Suisse; ^3^Hôpital Saint-Antoine AP-HP, Paris, France; ^4^Centre Hospitalier de Dieppe, Dieppe, France; ^5^François Mitterrand University Hospital, Dijon, France; ^6^Hôpital Necker-Enfants malades AP-HP, Paris, France

##### **Correspondence:** Mikhael Giabicani (mikhael.giabicani@aphp.fr)

*Annals of Intensive Care* 2023, **13(Suppl 1):**FC-0212

**Rationale:** Limitations of life-sustaining therapies (LST) practices are frequent and vary among intensive care units (ICUs). However, few data were available during the COVID-19 pandemic when ICUs were under intense pressure. We aimed to investigate the prevalence, cumulative incidence, timing, modalities, and factors associated with LST decisions in critically ill COVID-19 patients.

**Patients and methods/materials and methods:** We did an ancillary analysis of the European multicenter COVID-ICU study, which collected data from 163 ICUs in France, Belgium and Switzerland. ICU load, a parameter reflecting stress on ICU capacities, was calculated at the patient level using daily ICU bed occupancy data from official country epidemiological reports. Mixed effects logistic regression was used to assess the association of variables with LST limitation decisions.

**Results:** Among 4671 severe COVID-19 patients admitted from February 25 to May 4, 2020, the prevalence of in-ICU LST limitations was 14.5%, with a nearly sixfold variability between centers. Overall 28-day cumulative incidence of LST limitations was 12.4%, which occurred at a median of 8 days (3–21). Median ICU load at the patient level was 126%. Age, clinical frailty scale score, and respiratory severity were associated with LST limitations, while ICU load was not. In-ICU death occurred in 74% and 95% of patients, respectively, after LST withholding and withdrawal, while median survival time was 3 days (1–11) after LST limitations.

**Conclusion:** In this study, LST limitations frequently preceded death, with a major impact on time of death. In contrast to ICU load, older age, frailty, and the severity of respiratory failure during the first 24 h were the main factors associated with decisions of LST limitations.

**Reference 1**

Clinical characteristics and day-90 outcomes of 4244 critically ill adults with COVID-19: a prospective cohort study. Intensive Care Med. 2020;1–14.

**Compliance with ethics regulations:** Yes in clinical research.

**Figure 1 (abstract FC-212):**
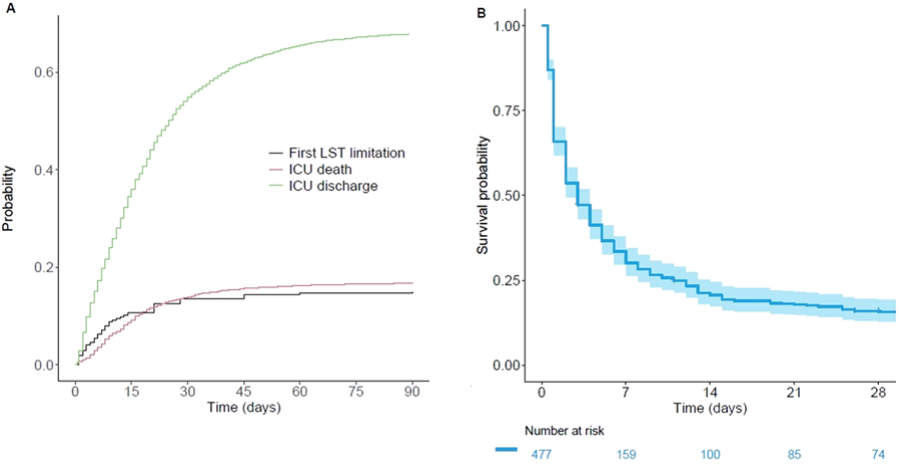
(A) Cumulative incidence plot of time from ICU admission to first LST limitation decision, and (B) survival probability after LST withholding or withdrawing decisions within 14 days after ICU admission

## FC-213 Post-traumatic stress disorder in survivors of severe covid-19 infections

### Abir Chihaoui^1^, Oussama Saadaoui^1^, Amina Haddad^1^, Zeineb Hammouda^1^, Iyed Maatouk^1^, Fadwa Houri^1^, Wiem Nouira^1^, Manel Lahmar^1^, Saoussen Ben Abdallah^1^, Fahmi Dachraoui^1^, Fekri Abroug^1^, Lamia Besbes Ouanes^1^

#### ^1^EPS Fattouma Bourguiba Monastir, Monastir, Tunisie

##### **Correspondence:** Zeineb Hammouda (zanoubia83@hotmail.com)

*Annals of Intensive Care* 2023, **13(Suppl 1):**FC-0213

**Rationale:** Post-traumatic stress disorder (PTSD) is quite common in hospitalized ICU patients. The aim of this study is to determine the prevalence of PTSD in the context of the COVID-19 pandemic in patients surviving severe covid 19 infections.

**Patients and methods/materials and methods:** This study was conducted on surviving COVID-19 patients who have been hospitalized in the intensive care unit of Monastir in 2020 and 2021 using a validated instrument: Post-Traumatic Stress Disorder Checklist-5 (PCL-5) (1) based on the Diagnostic and Statistical Manual of Mental Disorders (DSM-5)—for screening PTSD. Data were collected via a telephonic questionnaire.

**Results:** During the study period, 256 patients were hospitalized and discharged alive. We identified 105 patients' telephone numbers (41%). The telephonic questionnaire was conducted in 64 patients and 41 patients were not available for contact. The characteristics of the patients participating in the study were as follows: the median age of the patients was 55 years [47–61]. We noted a masculine predominance with a sex ratio of 1.6. All patients were ventilated with a non-invasive support and had prone sessions. The median SAPS II score was 25.5. The median PCL-5 score was 13 points (IQR = [7–26]). Among the population, seven patients (10.9%) had a PCL-5 score above the cut-off point of 31, which allowed the provisional diagnosis of posttraumatic stress disorder. We noticed that 35 patients (54.7%) had concentration and memory disorders. Sleeping disorders were present in 24 patients (37.5%) and 25 patients reported symptoms of irritability (39.1%). Four patients (6.3%) consulted a psychiatrist and only one patient was treated with benzodiazepines.

**Conclusion:** About ten percent of COVID-19 survivors had symptoms of PTSD. Active involvement of mental health professionals for psychosocial support and providing timely counseling is necessary to avoid the deleterious effects of the pandemic on mental health.

**Compliance with ethics regulations:** Yes in clinical research.

## FC-214 Psychological impact for family members of inter-regionally transfers of COVID-19 ICU patients

### Margaux Isnard^1^, Virginie Longueville^1^, Fabienne Prieur^1^, Alexandre Lautrette^2^, Vincent Peigne^1^

#### ^1^Centre hospitalier Métropole Savoie, Chambéry, France; ^2^Centre Jean Perrin, Clermont-Ferrand, France

##### **Correspondence:** Vincent Peigne (vincentpeigne@yahoo.fr)

*Annals of Intensive Care* 2023, **13(Suppl 1):**FC-0214

**Rationale:** In order to avoid ICU beds saturation during the first COVID-19 pandemic surges, inter-regional transfers of COVID-19 ICU patients were organized. The aim of this research is to determine a possible link between these transfers and the manifestation of psychological issues such as post-traumatic stress (PTSD), depression and anxiety among family members of the patients.

**Patients and methods/materials and methods:** Case/control observational prospective monocentric study with score measures IES-R (pathological threshold = 33/88) and HADS (pathological threshold at 7/14 for each scale) on one hand, and a qualitative analysis by two psychologists of semi-structured interviews conducted with family members of patients 6 months after their admission in ICU on the other hand. The case participants were close family members of the 23 patients transferred from our ICU in November 2020. The control participants were close family members of ICU patients admitted for a severe case of COVID-19 and who were not transferred. Cases and controls were paired according to age, gender and 3-month survival of the patients.

**Results:** Fifteen family members of transferred patients accepted to participate and were paired with 15 family members of non-transferred patients. The mean distance of inter-regional transfers was of 770 kms from the family member’s residence. Participants of the case group and control group had respectively symptoms of PTSD in 40% (n = 6) vs. 27% (n = 4) p = 0.7, symptoms of anxiety in 40% (n = 6) vs. 20% (n = 3) and symptoms of depression in 27% (n = 4) vs. 20% (n = 3).The qualitative analysis demonstrated that transfers were associated with increased anxiety due to the temporality of the decision making and the practical questions surrounding the medical transfer, intra-familial tensions and a sense of guilt that appeared sometime after the transfer. Four participants (27%) from the case group said that they would not give their consent for another transfer should the situation arise again.

**Discussion:** Although the small number of participants in this study (with both groups having a high prevalence of anxiety, depression and stress) did not produce robust quantitative data, the qualitative analysis helped identify a psychological burden linked to the transfers.

**Conclusion:** The consent to an inter-regional transfer of a family member seems associated to negative feelings (anxiety, sense of guilt). This data should be taken into account to improve the support provided to families of transferred patients should a new health crisis arise.

**Compliance with ethics regulations:** Yes in clinical research.

## FC-215 Health-related quality of life after ICU discharge among COVID-19 patients

### Haifa Sfar^1^, Safa Ben Mansour^1^, Dhouha Ben Braiek^1^, Yosri Ben Ali^1^, Hend Zorgati^1^, Sourour Bel Haj Youssef ^1^, Imen Mighri^1^, Rahma Ben Jazia^2^, Amani Kacem^2^, Jihene Ayachi^1^

#### ^1^Medical Intensive Care Unit, Ibn El Jazzar University Hospital, Kairouan, Tunisie; ^2^Pulmonology Department, Ibn El Jazzar University Hospital, Kairouan, Tunisie

##### **Correspondence:** Jihene Ayachi (ayachijihen@gmail.com)

*Annals of Intensive Care* 2023, **13(Suppl 1):**FC-0215

**Rationale:** ICU stay is known to negatively impact physical and mental well-being and consequently the health-related quality of life (HRQoL) after discharge. Little is known about the factors associated with a poor quality of life. The aim of the study was to evaluate the quality of life among COVID-19 ICU survivors and identify the risk factors of a poor HRQoL.

**Patients and methods/materials and methods:** A prospective analytical study conducted in a 9-bed medical ICU from May 2021 to September 2021.Survivors from acute respiratory distress syndrome (ARDS) related to COVID-9 fulfilled the SF-36 score 6 months after ICU discharge. SF-36 has 36 questions to assess general health-related quality of life. The 36 questions are divided into eight dimensions which are physical function, social functioning, role limitations due to physical problems, role limitations due to emotional problems, mental health, bodily pain, vitality (energy and fatigue), and general health perception. The overall score on each SF-36 subscale ranges from 0 to 100, and a higher score indicates a better QoL. Scores for the different subscales were converted and a reference level of 50 points was adopted for physical and mental health.

**Results:** During the study period, 70 patients with ARDS related to COVID-19 were discharged alive, among them 48(68.6%) patients completed the SF-36 questionnaire. Mean age was 48.2 ± 11.38 years with male predominance (n = 28, 58.3%). Diabetes (n = 8, 16.7%) was the most common comorbidities. Mean Body Mass Index was 28.5 ± 3.65 kg/m^2^ and median SAPS-II score was 22 [13–24]. Patients were discharged under oxygen therapy in 25 (52.1%) and 15 (31.3%). For the physical capacity, univariate analysis identified female gender (p = 0.018), home oxygen therapy (p = 0.009), dyspnea (p < 0.001), age (p = 0.005), anosmia (p = 0.028) and palpitations (p = 0.001) as risk factors for impaired physical capacity. Multivariate analysis identified home oxygen therapy (OR, 19.8; 95% CI, [2–194.7]; p = 0.01 and palpitations (OR, 26.5; 95% CI, [2.9–241.3]; p = 0.004) as independent risk factors for decreasing physical capacity. For perception of general health, univariate analysis identified age (p = 0.022), Charlson Comorbidity Index (CCI) (p = 0.047) and unemployment (p = 0.031) as risk factors for impaired general health. In multivariate analysis, only CCI was the independent risk factor of a misperception of general health (OR, 2.18; 95% CI, [1.06–4.51]; p = 0.035).

**Conclusion:** ICU COVID-19 patients’ HRQoL slightly deteriorate 6 months after discharge especially for female, older patients and those with home oxygen therapy. Nevertheless, medications received during ICU stay had no effect on physical or mental HRQoL.

**Compliance with ethics regulations:** Yes in clinical research.

## FC-216 Long-term “post-covid” in survivors of COVID-19 ARDS

### Wiem Nouira^1^, Dorra Berkhaies^1^, Hanene Lahmar^1^, Zeineb Hammouda^1^, Iyed Maatouk^1^, Manel Lahmar^1^, Oussama Saaadaoui^1^, Hedia Ben Ahmed ^1^, Saoussen Ben Abdallah^1^, Fahmi Dachraoui^1^, Fekri Abroug ^1^, Lamia Ouanes Besbes^1^, Abir Chihaoui^1^

#### ^1^CHU Fattouma Bourguiba Monastir Tunisie, Monastir, Tunisie

##### **Correspondence:** Wiem Nouira (wiemnouira1@gmail.com)

*Annals of Intensive Care* 2023, **13(Suppl 1):**FC-0216

**Rationale:** A growing body of evidence support that a substantial minority of patients surviving the acute phase of the human coronavirus 2019 disease (COVID-19) and the associated acute respiratory distress syndrome (ARDS) present with long-term sequelae lasting for up to 6 months following acute infection. The aim of this study was to identify persistent long-term symptoms in survivors of COVID-19 ARDS for up to 2 years.

**Patients and methods/materials and methods:** This is a descriptive study including the surviving COVID-19 patients with ARDS hospitalized in the ICU of the University Hospital Fattouma Bourguiba of Monastir between April 2020 and December 2021 (n = 240). A telephone survey was conducted in February 2023, asking the survivors COVID-19 patients with ARDS about persistent COVID-19 symptoms in the 2 years following their ICU discharge.

**Results:** Of the 95 patients contacted, 69 (72.6%) could be reached. 58% of the patients were male. The median age was 57 years (interquartile range (IQR): 45–64 years old), the median duration of stay in the ICU was 11 days (IQR = 8–17 days). During their ICU stay, high flow nasal canula was used in 83% of patients. 91% of them complained of persistent symptoms and only one patient died 2 months after her discharge. The main symptoms described were dyspnea (68.8%), memory impairment (57%), asthenia (55.6%) and chest pain (31.7%).

**Conclusion:** Long-term persistent COVID-19 symptoms are common, dominated by respiratory symptoms, cognitive disorders and asthenia. These conditions may require careful clinical assessment, treatment and close follow-up to avoid short-term and long-term complications.

**Compliance with ethics regulations:** Yes in clinical research.

## FC-217 Multiple-site decontamination to prevent acquired infection in patients with veno-venous ECMO support

### Nicolas Massart^1^, Christophe Camus^2^, Nicolas Nesseler^2^, Pierre Fillatre^1^, Erwan Flecher^2^, Alexandre Mansour^2^, Jean-Phillipe Verhoye^2^, Lucie Lefevre^3^, Charles-Edouard Luyt^3^

#### ^1^Centre Hospitalier Yves Le Foll, Saint-Brieuc, France; ^2^CHU Rennes - Pontchaillou, Rennes, France; ^3^APHP, Paris, France

##### **Correspondence:** Nicolas Massart (nicolasmassart@hotmail.fr)

*Annals of Intensive Care* 2023, **13(Suppl 1):**FC-0217

**Rationale:** Acute distress respiratory syndrome (ARDS) patients with veno-venous extra corporeal membrane oxygenation (ECMO) support are particularly exposed to ECMO-associated infection (ECMO-AI) (1). Unfortunately, data regarding AI prophylaxis in this setting is lacking. Selective decontamination regimens decrease AI incidence, including ventilator-associated pneumonia (VAP) and bloodstream infection (BSI) in critically ill patients (2). We hypothesized that a multiple-site decontamination (MSD) regimen is associated with a reduction in the incidence of AI among VV-ECMO patients.

**Patients and methods:** We conducted a retrospective observational study in three French ECMO referral centers from January 1st 2010 to December 31th 2021. All adult patients (> 18 year old) who received VV-ECMO support for ARDS were eligible. In addition to standard-care (SC), 2 ICUs used MSD, which consists of the administration of (i) an aminoglycoside, colistin sulfate and amphotericin B, four times daily in the oropharynx and the gastric tube, (ii) once daily chlorhexidine body wash and (iii) a 5-day nasal mupirocin course. AIs were compared between the 2 ICUs using MSD (MSD group) and the last ICU using SC.

**Results:** There were 241 patients available for the study. Sixty-nine were admitted in an ICU that applied MSD while the 172 others received standard care and constituted the SC group. There were 19 ECMO-AIs (12 VAP, 7 BSI) in the MSD group (1162 ECMO-days) compared to 143 AIs (104 VAP, 39 BSI) in the SC group (2376 ECMO-days), (p < 0.05 for all infection site). In a Poisson regression model, MSD was independently associated with a lower incidence of ECMO-AI (IRR = 0.42, 95% CI [0.23–0.60] p < 0.001). There were 30 multidrug resistant microorganisms (MDRO) acquisition in the SC group as compared with two in the MSD group (IRR = 0.13, 95% CI [0.03–0.56] p = 0.001). Patients in the DMS group had fewer days with antimicrobial treatment for AI as compared with SC group (0 days [0–5] vs 7 days [0–14] p < 0.001), but also more days alive without antimicrobial treatment for AI (50 days [16–60] vs 41 days [3–60] p = 0.012). Mortality in ICU was similar in both groups (43% in the SC group vs 45% in the MSD group p = 0.90). Results were similar after propensity-score matching (Figure).

**Conclusion:** In this retrospective observational study, VV-ECMO patients receiving MSD had lower ECMO-AI and MDRO acquisition incidences. Since residual confounders may persist, these promising results deserve confirmation by randomized controlled trials.

**Reference 1**

1- Grasselli G, Scaravilli V, Di Bella S, Biffi S, Bombino M, Patroniti N, Bisi L, Peri AM, Pesenti A, Gori A, Alagna L. Nosocomial infections during extracorporeal membrane oxygenation: incidence, etiology, and impact on patients’ outcome. Crit Care Med.

**Reference 2**

2- Massart N, Reizine F, Fillatre P, Seguin P, La Combe B, Frerou A, Egreteau PY, Hourmant B, Kergoat P, Lorber J, Souchard J, Canet E, Rieul G, Fedun Y, Delbove A, Camus C. Multiple-site decontamination regimen decreases acquired infection incidence in m.

**Compliance with ethics regulations:** Yes in clinical research.

**Figure 1 (abstract FC-217):**
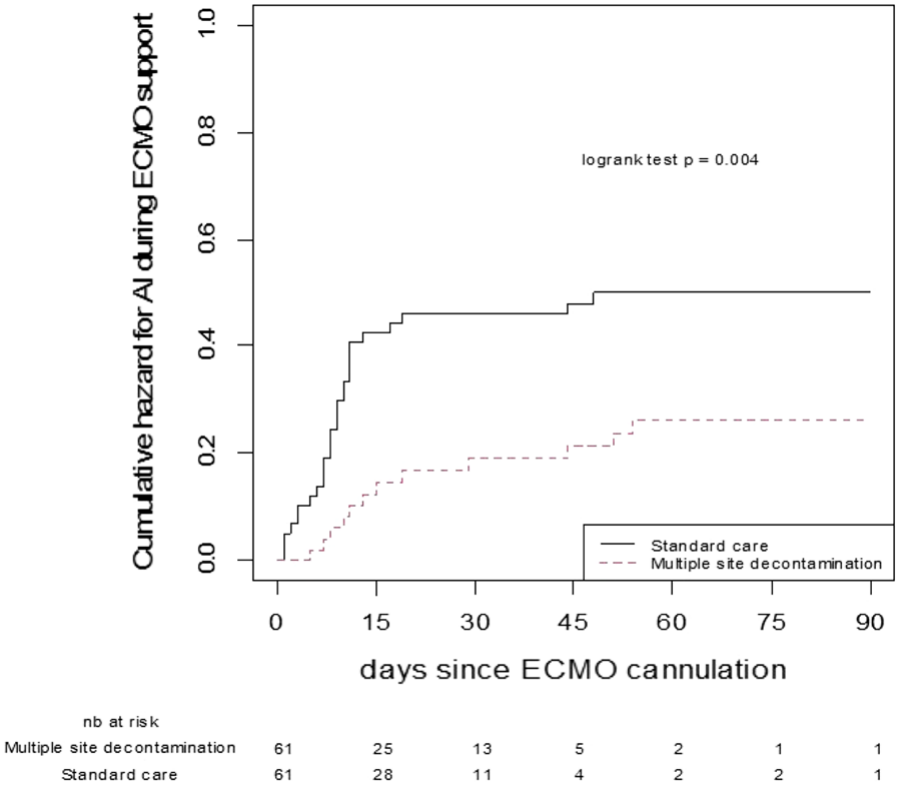
ECMO-AI cumulative incidences

## FC-218 Ultrasound guidance practices used for the placement of central venous catheters in intensive care units an observational multicentre study

### Nathalie Van Der Mee-Marquet^1^, Anne-Sophie Valentin^1^, Isabelle Duflot^1^, Mathilde Farizon^1^, Agnès Petiteau^1^

#### ^1^CHRU, Tours, France

##### **Correspondence:** Nathalie Van Der Mee-Marquet (n.vandermee@chu-tours.fr)

*Annals of Intensive Care* 2023, **13(Suppl 1):**FC-0218

**Rationale:** Central catheters expose the patients to infectious complications responsible for morbidity and mortality. Ultrasound guidance (USG) is recommended to determine the correct place to insert these catheters. The impact of this technique on the risk of catheter-related infection is evoked in recent cohort studies. Our goal is to evaluate the breaches in aseptic conditions during catheter insertion in routine practice in French ICUs.

**Patients and methods/materials and methods:** We carried out an inventory of the use of USG during central catheter insertion based on both a questionnaire addressed to intensivists and an observational study of their practices.

**Results:** We analyzed 111 questionnaires and 36 direct observations from 26 ICUs. The questionnaires revealed most HCWs (88%) using USG for central catheter insertion. Among those using USG, 56% are trained in USG, 17% benefit from a specific procedure, 76% carry out insertion site marking before skin disinfection, and 96% use single-dose sterile gel and 100% a sterile sheath during catheter insertion. Direct observation established that the use of the sterile devices starts often too early, that is to say as soon as the marking phase [i.e., gel (24%) and sheath (29%)] or during skin disinfection (i.e., gloves (100%). In these cases, the sterile devices are contaminated before the beginning of the insertion phase, and maximum aseptic conditions are not ensured during catheter insertion. Direct contacts between the sheath and the catheter were observed during the catheter insertion phase (19%), and one out of the 36 HCWs (3%) continued catheter insertion after handling US system with his gloved hands.

**Conclusion:** There is need for interventions to improve compliance with prevention measures necessary for safe insertion of central catheters and prevention of the breaches in aseptic conditions during catheter insertion..

**Compliance with ethics regulations:** N/A.

## FC-219 Ultrasound guidance for placement of jugular venous catheters

### Alaeddine Zouari^1^, Safia Othmani^1^, Hana Hedhli^1^, Asma Mellouli^1^, Rezk Ghorbel^2^, Aymen Zoubli^1^, Sarra Jouini^1^

#### ^1^Hôpital Charles nicolle, Tunis, Tunisie; ^2^Hôpital Habib Bourguiba, Sfax, Tunisie

##### **Correspondence:** Alaeddine Zouari (zouari.aladin@gmail.com)

*Annals of Intensive Care* 2023, **13(Suppl 1):**FC-0219

**Rationale:** The placement of central venous catheters can be followed by failures or complications with high morbidity. This can be reduced by the use of ultrasound guidance.

**Patients and methods/materials and methods:** We conducted a prospective study at the emergency department during a period of 3 months from February to April 2022. This study included the patients benefiting from ultrasound-guided jugular catheter placement. All patients were sedated and ventilated at the time of catheter placement. No patient was drained at the time of placement. Catheter placement was performed by emergency medicine residents. The insertion time was calculated from the puncture of the skin by the needle till it’s the ablation. Patients with a major hemostasis disorder or a contraindication to the placement of a jugular catheter were excluded.

**Results:** This study included 23 patients including 17 men and 6 women. The average age of our patients was 52 years with extremes ranging from 14 to 76 years. On clinical examination, four patients (17%) were with hemodynamic instability. Six patients (26%) had respiratory failure. Two patients (8%) showed signs of right heart failure. The lactate assay showed an average lactatemia of 5.4 mmol/L. The placement of the catheter was performed by a first year-resident in twelve cases (52%) and by a third year-resident in eleven cases (48%). The average set-up time was 110 s with extremes ranging from 20 s to 10 min. Thirteen patients (61%) underwent a single puncture, six patients (28%) two punctures and two patients more than three punctures respectively, i.e. 82% of patients had one or two successful punctures. The failure of catheter placement was encountered in two patients (8.7%). Immediate complications were encountered in five patients (21.7%), all of whom had an arterial puncture. No case of pneumothorax or subsequent complications were reported.

**Conclusion:** Ultrasound-guided placement of jugular venous catheters reduces complications and failure according to our study and the literature.

**Compliance with ethics regulations:** Yes in clinical research.

## FC-220 Needle guide for in plane ultrasound guided venous catheterization

### Antoine Villa^1^, Vladimir Hermand^2^, Vincent Bonny^1,3^, Gabriel Preda^1^, Tomas Urbina^1^, Maxime Gasperment^1^, Paul Gabarre^1,3^, Louai Missri^1^, Jean-Luc Baudel^1^, Daniel Zafimahazo^1^, Jérémie Joffre^1,3,4^, Hafid Ait-Oufella^1,3,5^, Eric Maury^1,3,6^

#### ^1^Medical Intensive Care Unit, Hôpital Saint Antoine, Assistance Publique-Hôpitaux de Paris, Paris, France; ^2^Learning Planet Institute, Paris, France; ^3^Sorbonne University, Faculty of Medicine, Paris, France; ^4^Centre de Recherche Saint-Antoine (CRSA), INSERM UMR_S938, Paris, France; ^5^Paris University, Paris Cardiovascular Research Center, INSERM U970, Paris, France; ^6^Sorbonne University, Pierre Louis Institute of Epidemiology and Public Health, INSERM U1136, Paris, France

##### **Correspondence:** Antoine Villa (antoine.villa.bober@gmail.com)

*Annals of Intensive Care* 2023, **13(Suppl 1):**FC-0220

**Rationale:** Ultrasound-guided insertion of central venous catheters (CVCs) is most of the time performed using the out of plane (OOP) approach. However, the in plane (IP) approach, owing to visualization of the entire needle path, could improve safety and efficacy of catheterization. Nevertheless, the IP approach is less easy to master due to difficulty to maintain the needle in the ultrasound beam during puncture. Needle guiding systems actually available are either single use or probe specific and often expensive. This study aimed to assess, in a simulation survey, a homemade needle guide device built with a 3D printer, to improve CVC insertion IP approach.

**Patients and methods/materials and methods:** A prospective, randomized crossover study conducted on an inanimate manikin, among physicians with inhomogeneous proficiency, was conducted to assess the impact of a homemade needle guide on central venous puncture. Each participant performed ultrasound guided puncture of internal jugular, subclavian and femoral vein, with three different approaches, assigned in random order: out of plane free hand (OOP-FH), in plane free hand (IP-FH), and in plane needle guided (IP-NG). Success rate at first pass, time elapsed (i) from skin contact to first skin puncture, (ii) from first skin puncture to successful venous puncture and (iii) from skin contact to successful venous return and number of needle redirections as well as success rate were measured.

**Results:** Six iterations were printed and tested before obtaining the perfect fit for the probe. Design and conception took 36 h. Thirty participants accepted to take part of the study. NG-IP approach translated in high success rate at first pass (jugular: 80%, subclavian: 95% and femoral 100%) and was significantly higher than the one observed with OOP-FH and IP-FH approaches whatever site (p < 0.001) (Figure 1). Compared to IP-FH approach, the IP–NG approach decreased the total procedure duration (23 s vs 16 s, p = 0.04 jugular vein; 30 s vs 20 s, p = 0.006 subclavian vein; 20 s vs 18 s, p = 0.05, femoral vein). Moreover, compared to OOP-FH and IP-FH, the IP-NG approach significantly decreased the number of needle punctures (skin breaches) or redirections at all sites (p = 0.009). Finally uncomplicated puncture defined as successful puncture performed in less than 120 s without arterial puncture occurred more frequently with NG-IP compared to IP-FH and OOP-FH (95%, 80%, 70%, respectively, p = 0.01).

**Conclusion:** For ultrasound guided central vein puncture, in-plane approach using a homemade needle guide decreased both entire procedure duration and puncture/redirections while it increased success rate compared to free hand approaches.

**Compliance with ethics regulations:** N/A.

**Figure 1 (abstract FC-220):**
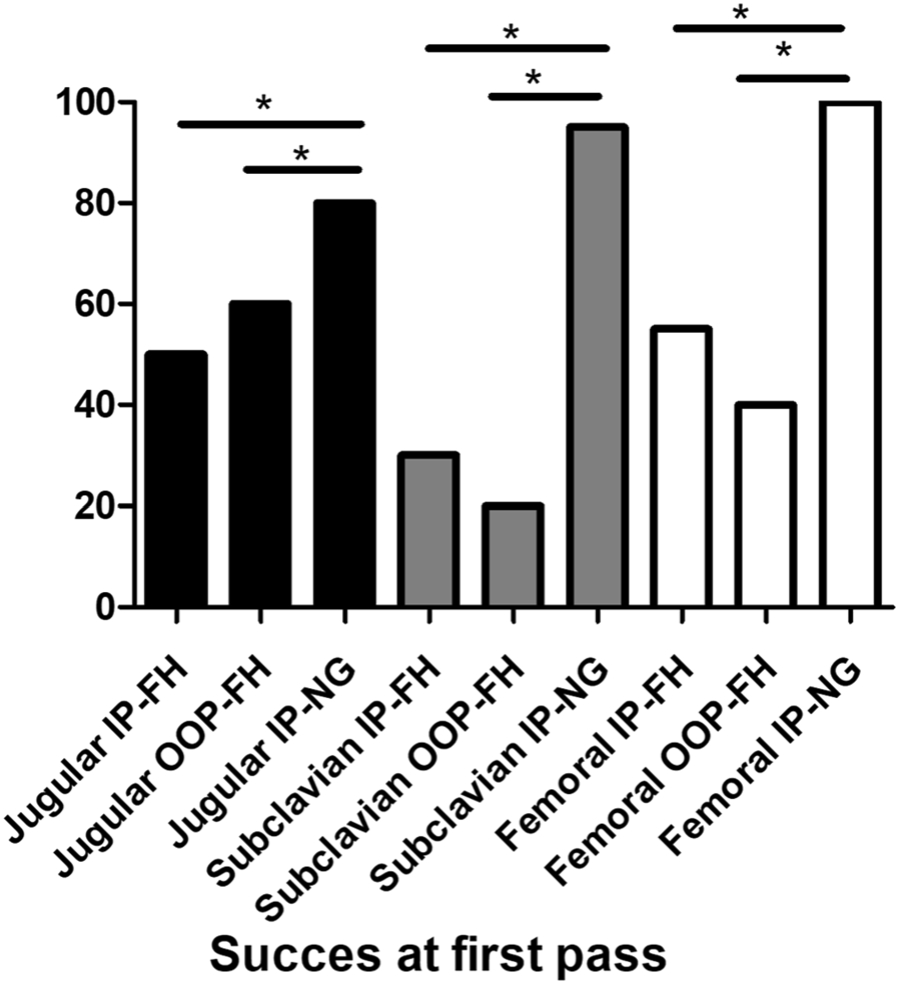
Success at first pass

## FC-221 Adverse events related to intravascular catheters in ICU: incidence and impact on outcome

### Najla Ben Slimene^1^, Imen Talik^1^, Boudour Ben Dhia^1^, Fatma Essafi^1^, Khaoula Ben Ismail^1^, Moez Kaddour^1^, Takoua Merhabene^1^

#### ^1^Hôpital régional de Zaghouan, Tunis, Tunisie

##### **Correspondence:** Najla Ben Slimene (najlabenslimene@gmail.com)

*Annals of Intensive Care* 2023, **13(Suppl 1):**FC-0221

**Rationale:** Patient safety is the absence of unnecessary or potential harm associated with health care. One of its indicators is the occurrence of adverse events (AE), especially in critical care where patients need heavy therapeutics. The aim of our study was to assess the incidence of adverse events related to intravascular catheters and determine their impact on patient’s outcome.

**Patients and methods/materials and methods:** We conducted a prospective descriptive analytic study in the ICU of Zaghouan’s hospital between January 2018 and December 2020, including all patients over 18 years old admitted and having had intravascular catheters: Peripheral venous catheter (PVC), central venous catheters (CVC) and arterial catheters (AC). Were calculated the incidence of adverse events related to intravascular catheters. The risk factors for occurrence of AE were determined by a multivariate analysis and the impact on outcomes was reported.

**Results:** We included 556 patients during the study period. The mean age was 52 ± 19 years with a male predominance. Patients had 1423 intravascular catheters during their ICU stay (834 PVC, 462 CVC and 127 AC) for 3503 days of catheterization. Adverse events related to intravascular catheters were reported in 40 patients with 74 episodes during 2 years. Estimated incidence was 5%. AEs occurred in a median delay of 4 [2–11] days. The following table shows the details of AE related to intravascular catheters (Table I). The univariate analysis showed that the occurrence of AE was a risk factor for mortality (*p* = 0.008) and a longer ICU length of stay (*p* = 0.001).

**Conclusion:** Although adverse events related to intravascular catheters are usually rare, they can have serious consequences on the patient’s outcome.

**Compliance with ethics regulations:** Yes in clinical research.

**Table 1 (abstract FC-221):**
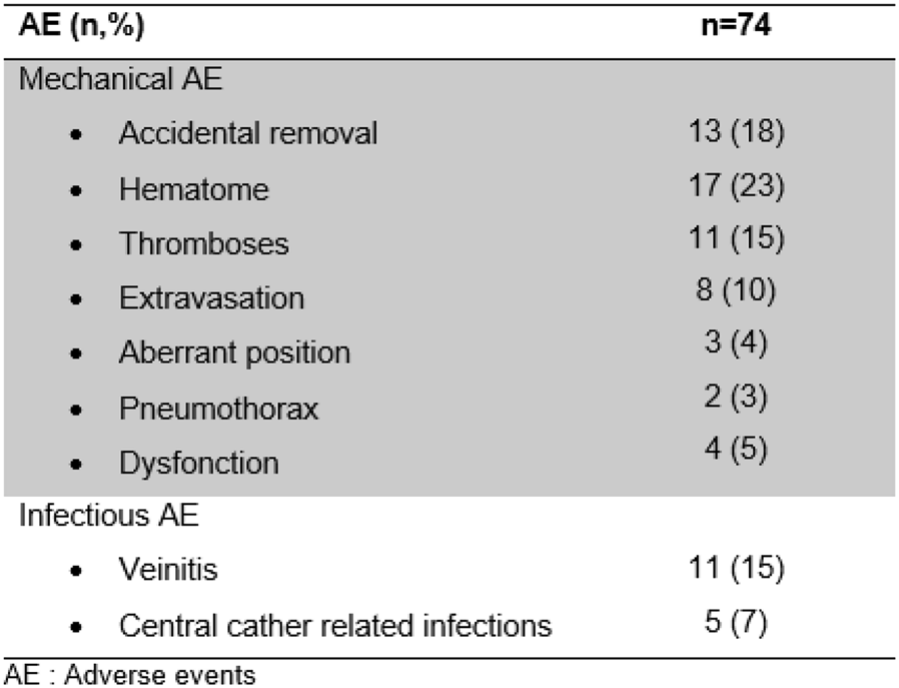
Adverse events related to intravascular catheters

## FC-222 Assessment of mechanical complications of central venous catheterization and arterial lines in intensive care unit

### Khaoula Ben Ismail^1^, Iheb Glenza^1^, Mohsna Bhiri^1^, Fatma Essafi^1^, Najla Ben Slimene^1^, Talik Imen^1^, Moez Kaddour^1^, Takoua Merhabene^1^

#### ^1^Hôpital régional de Zaghouan, Zaghouan, Tunisie

##### **Correspondence:** Iheb Glenza (iheb.glenza@gmail.com)

*Annals of Intensive Care* 2023, **13(Suppl 1):**FC-0222

**Rationale:** The placement of central venous catheters and arterial lines is a common procedure used by intensivists in the monitoring of critically ill patients. However, this invasive procedure has the potential of minor and major mechanical complications. The aim is to describe the expected incidence of mechanical complications associated with central venous and arterial cannulation in critically ill patients.

**Patients and methods/materials and methods:** We monitored prospectively occurrence of minor and major mechanical complications after central venous catheters and arterial lines implemented by intensivists from March 17 to September 9, 2022, in all departments of the regional hospital of Zaghouan, Tunisia. We proceeded by daily clinical examination of the insertion sites of catheters and collected epidemiological, clinical and paraclinical data.

**Results:** During the study period, a total of 70 procedures were performed:53 central venous catheters, 10 peripheral arterial lines, 7 central arterial lines (85% in intensive care unit and 10% of procedures were inserted at night). Median age of the included patients was 53 years [25–79]. A clear male predominance was noted (gender ratio 1.8). Chronic heart disease, diabetes mellitus, hypertension and chronic obstructive pulmonary disease were the most common comorbidities (25%, 22%, 21% and 14% respectively). Acute respiratory failure and coma were the main causes of hospital admission (24% and 21% respectively). Average APACHE II and SAPS II scores were 12 and 30. In 15 operators, 87% were right-handed and 4% had limited experience. Insertion sites were: Femoral 68% (48), Subclavian 14% (10), Radial 14% (10) and Jugular 2% (2). Main indications were: Catecholamines administration (65%), administration of Veno toxic drugs (15%) and metabolic disorders managing (13%). Median number of active health workers present through the procedure was 3 [1–5]. In 95% of cases, the procedures were successful on the 1st attempt. Only 5% (4) of the inserted catheter were subject to an immediate mechanical complication: Two Subcutaneous Hematoma with a peripheral arterial line, One Pneumothorax with a Subclavian central venous catheter and one aberrant trajectory with a jugular line. No fatal complication was noted. The multivariable logistic regression analysis showed that subclavian vein catheterization (OR 5.91 [95% CI [2.13–17.26]; P = 0.001), limited operator experience (3.29 [1.19–9.61]; P = 0.024) and night procedure (OR 2.1 [95% CI [1.13–6.26]; P = 0.04) were independently factors associated with mechanical Complications.

**Conclusion:** Subclavian site, Night procedure timing and limited operator experience represent the risk factors for developing mechanical complications. Standardization of the procedures through guided recommendations may develop better practice.

**Compliance with ethics regulations:** Yes in clinical research.

## FC-223 Tracheostomy in critically ill patients: practices and outcomes in a Tunisian medical ICU

### Ines Sebri^1^, Sourour Bel Haj Youssef ^1^, Dhouha Ben Braiek^1^, Khalil Attia^1^, Hend Zorgati^1^, Yosri Ben Ali^1^, Rahma Ben Jazia^2^, Amani Kacem^2^, Jihene Ayachi^1^

#### ^1^Medical Intensive Care Unit, Ibn El Jazzar University Hospital, Kairouan, Tunisie; ^2^Pulmonology Department, Ibn El Jazzar University Hospital, Kairouan, Tunisie

##### **Correspondence:** Jihene Ayachi (ayachijihen@gmail.com)

*Annals of Intensive Care* 2023, **13(Suppl 1):**FC-0223

**Rationale:** Tracheostomy is a frequently used procedure in critically ill patients. It has many advantages when prolonged ventilation is required. Indeed, it allows reduced sedation requirement, gradual weaning of ventilator and enhanced nursing care. Aim: To describe state of tracheostomy practices and outcomes in a Tunisian medical ICU.

**Patients and methods/materials and methods:** A prospective descriptive study was carried out in a 9-beds medical ICU at university hospital, from January 2021 to January 2023. Were included all patients who required tracheostomy for prolonged ventilator support due to chronic coma or difficult weaning from the ventilator. All patients had planned surgical tracheostomy. They were all performed at the bedside by ICU team or by a dedicated ear, nose and throat (ENT) surgeon in the operating room. Were collected: clinical features at admission, reason for admission, severity of illness, indication, timing, and technique of tracheostomy, duration of mechanical ventilation, length of stay and the various complications. Outcomes were assessed by in-hospital mortality rate.

**Results:** A total of 22 patients were included within the study period: 12 male (54.5%) and 10 female (45.5%), with median age of 54.5 [35.5–64] years. Mean SAPSII and APACHE II were respectively 41.09 ± 13.99 and 18.86 ± 6.75 and median Charlson index at 1 [0–2.25]. Twelve patients (54.5%) were admitted for acute respiratory failure and 5 (22.7%) had COPD with GOLD stage of D. Fifteen patients (68.2%) received invasive mechanical ventilation as a first line ventilator support and 3 (13.6%) required vasopressors. Bedside surgical technique was used in 63.6%. The indication for tracheostomy was weaning difficulty in 14 patients (63.6%) and chronic coma in 8 patients (36.4%). The mean time from tracheal intubation to tracheostomy was 12.68 ± 5.02 days with mean ventilation duration with tracheostomy of 13 ± 9.42 days. There was no death related to tracheostomy. Post-operative complications, especially tracheal stenosis, occurred in only one patient. Mean ICU stay was at 27.8 ± 12.5 days with ICU mortality rate at 31.8%. Fifteen patients (68.2%) were discharged alive, among them: only 5 patients (33.3%) required home ventilation for difficult weaning and 3 patients (20%) discharged after successful tracheostomy tube removal. Three patients (20%) had full autonomy. Nine patients (60%) were fed with gastric tube and one with gastrostomy.

**Conclusion:** Planned tracheostomy whether performed surgically at the bedside or by an ENT surgeon was a safe procedure as the rate of complications is very low with an improvement in ventilation management and weaning and reduction in mortality.

**Compliance with ethics regulations:** Yes in clinical research.

## FC-224 Exclusion of mechanical complications related to placement of central venous catheters: interest of ultrasonography

### Rabeb Hammami^1^, Mahmoud Marzouk^1^, Asma Hosni^1^, Intissar Abassi^1^, Maryem Ben Amor^1^, Sbeur Thamlaoui^1^, Nader Baffoun^1^, Chokri Kaddour^1^

#### ^1^Institut National De Neurologie, Tunis, Tunisie

##### **Correspondence:** Rabeb Hammami (hammamirabeb2@gmail.com)

*Annals of Intensive Care* 2023, **13(Suppl 1):**FC-0224

**Rationale:** The placement of central venous catheters (CVCs) is a frequent procedure in intensive care. It is not without complications, the diagnosis of which was for a long time based on chest X-rays. Currently, ultrasound, a non-irradiating tool, appears to be an interesting alternative. The aim of our study was to compare the radiological and ultrasound times required to exclude immediate mechanical complications (pneumothorax and catheter misplacement) after CVC placement and to study the incidence of complications according to the diagnostic tool.

**Patients and methods/materials and methods:** This was a prospective observational study conducted in an intensive care unit. Patients older than 18 years of age who had the decision to have a CVC placed were included. After placement, a chest X-ray was requested and an ultrasound was performed to look for signs of misplacement in an aberrant vein and for signs of pneumothorax (absence of slippage, absence of B-lines, and search for lung point or pleural pulse). The interpretation of the two examinations was done by two different doctors. The T1 ultrasound or radiological time was the time required to exclude complications.

**Results:** 30 patients were included in our study. The average age was 48.1 ± 17.77 years. The sex ratio was 1.3. The most frequent reasons for admission were: Stroke in 23.33% of cases, neuropathy in 23.33%, and polytrauma in 12.9%. The history was dominated by hypertension in 23.33% of patients and diabetes in 13.33%. The mean APACHE III score was 56.97 ± 31.94. CVCs were placed on the right side in 66.7% of cases and on the left side in 33.3%. The distribution in the 4 veins is represented as follows: Right SVC (33.3%), right IJV (26.7%), left SVC (23.3%), and left IJV (16.7%). The average number of punctures was 2.27 ± 1.99 and the average insertion time was 19.24 ± 14.50 min. There was a significant difference between the mean T1 ultrasound and radiological time (Table 1). There were no complications of misplacement. Only one case of pneumothorax was observed (3.3%) after the left VSC puncture. It was first detected by ultrasound. This was confirmed by standard radiography and chest CT scan showing an upper and anterior left pneumothorax.

**Conclusion:** Mechanical complications after CVC placement are rare. Ultrasound is a promising diagnostic tool for the exclusion of these complications. It guarantees a rapid and non-irradiating examination for intensive care patients compared to radiography.

**Compliance with ethics regulations:** Yes in clinical research.

## FC-225 Epidemiology of children with sickle cell disease admitted to pediatric intensive care unit after haematopoietic stem cell transplantation

### Charlotte Pourdieu^1^, Adrien Arsene^1^, Julie Sommet^1^, Jérôme Naudin^1^, Aurélie Hayotte^1^, Arielle Maroni^1^, Stéphane Dauger^1^, Michael Levy^1^

#### ^1^Hôpital Universitaire Robert-Debré, Paris, France

##### **Correspondence:** Michael Levy (michael.levy@aphp.fr)

*Annals of Intensive Care* 2023, **13(Suppl 1):**FC-0225

**Rationale:** Over the last decades, in high-income countries, the implementation of newborn screening together with prophylactic penicillin, parental education, anti-pneumococcal vaccination, regular follow-up with prompt appropriate therapeutic interventions, chronic transfusions and haematopoietic stem cell transplantation (HSCT), have led to a decrease in mortality in children with sickle cell disease (SCD). However, few data exist on the evolution of these patients after HSCT and especially regarding the need to be admitted in Pediatric Intensive Care Units (PICU). The aim of this study was to describe the epidemiology of patients with SCD admitted to PICU after HSCT.

**Patients and methods/materials and methods:** We conducted a single center retrospective study in a tertiary center PICU. Consecutive children with SCD admitted to PICU between January 1st, 2009 and December 31, 2019 after HSCT were included. Patients were identified by searching the hospital databases for codes D57 and Z9480 in the International Classification of Diseases-10th revision then selecting those who required PICU admission. All admissions during the study period were included and one patient could have several admissions.

**Results:** 17 patients were admitted during the study period and accounted for 24 PICU stays. All patients were of SS genotype before HSCT and the median age was 9.3 [7.1–11.8]. Patients were admitted after a median of 42.5 [20.2–69] days after HSCT. Most patients were admitted from the hematology unit and only 2 patients from the emergency department. The two main reasons for admissions were acute respiratory failure (n = 8) and shock (n = 8). The main diagnosis were septic shock (n = 4), sepsis (n = 3), pericarditis (n = 4) and acute respiratory distress syndrome (n = 2). 11 (46%) patients required non-invasive-ventilation and 9 (8%) invasive ventilation. 6 (25%) required fluid resuscitation and 4 (17%) vasoactive drugs. The median length of stay was 3.5 ([2–6.5] days and one (4%) patient died of septic shock.

**Conclusion:** Patient with SCD admitted to PICU after HSCT were mainly admitted for respiratory and cardiovascular failures. The median length of stay was short and the mortality low.

**Compliance with ethics regulations:** Yes in clinical research.

## FC-226 A pilot randomized controlled trial to organize OpTTICCA, which will be a large international trial on the efficacy and safety of a low hemoglobin threshold to guide red blood cell transfusions in almost all anemic critically ill children

### Marisa Tucci^1^, Geneviève Du Pont-Thibodeau^1^, Stéphane Leteurtre^2^, Avishay Sarfatti^3^, Samiran Ray^4^, Simon Stanworth^5^, Patricia Fontela^6^, Pierre Demaret^7^, Ducruet Ducruet^1^, Jacques Lacroix^1^

#### ^1^CHU Sainte-Justine, Montréal, Canada; ^2^Hôpital Jeanne de Flandre, CHRU Lille, Lille, France; ^3^Oxford University Hospitals NHS Foundation Trust, Oxford, Royaume-Uni; ^4^Great Ormond Street Hospital, London, Royaume-Uni; ^5^NHS Blood & Transplant/Oxford Radcliffe Hospitals, Oxford, Royaume-Uni; ^6^The Montreal Children's Hospital, Montréal, Canada; ^7^CHC Liège, Liège, Belgique

##### **Correspondence:** Jacques Lacroix (jlacroix052@gmail.com)

*Annals of Intensive Care* 2023, **13(Suppl 1):**FC-0226

**Rationale:** The main recommendation of the Transfusion Requirement In Pediatric Intensive Care Units (TRIPICU) trial1 was to consider a red blood cell (RBC) transfusion to hemodynamically stable critically ill children only if their hemoglobin (Hb) concentration is < 70 g/L. TRIPICU was published in 2007; in 2021, there was still a clinically significant gap in the application of this recommendation2. We undertook a pilot randomized controlled trial (RCT) to assess the feasibility of Optimizing Transfusion Threshold in Critically-ill Children with Anaemia (OpTTICCA) which will be a large international non-inferiority RCT addressing the efficacy and safety of Hb thresholds of 70 g/L not only in hemodynamically stable children, but in almost all PICU patients with a Hb concentration ≤ 95 g/L.

**Patients and methods/materials and methods:** The pilot-OpTTICCA study (P-OpTTICCA) was a small multicenter international non-inferiority RCT; its basic design is described in the registry “clinicalTrials.gov” (NCT03871244). Its main objective was to assess the feasibility of OpTTICCA. All patients with a Hb ≤ 95 g/L were eligible to participate to this pilot RCT.

**Results:** 120 patients were randomized to a restrictive (transfusion only if Hb < 70 g/L) or to a liberal RBC strategy (standard care) in four university affiliated PICU in Canada, France and the United Kingdom. There was almost no difference at baseline in the 63 children allocated to the restrictive arm and the 57 children allocated to the control arm (Table 1). The outcomes were almost similar in the restrictive and the liberal arm of P-OpTTICCA. The incidence rate of the primary outcome measure (new and progressive multiple organ dysfunction syndrome) was similar in the restrictive and the liberal arm of the trial (5 and 6 cases, respectively). The highest daily PELOD score post-randomization was 4.9 ± 3.4 and 4.9 ± 4.7. The number of ventilator-free days was 12.4 ± 43.7 and 12.6 ± 35.2 days, and the number of PICU-free days was 18.6 ± 8.1 and 18.6 ± 8.8 days, respectively. We observed no withdrawal, no protocol violation and two protocol deviations.

**Discussion:** We observed almost no difference in the baseline data and in the most important outcomes monitored in P-OpTTICCA.

**Conclusion:** Given that the incidence rate of the primary and the most important outcomes are almost similar among participants to P-OpTTICCA, the basic design of OpTTICCA should be a non-inferiority RCT.

**Reference 1**

Lacroix J, Hébert PC, Hutchison JH, et al. Transfusion strategies for patients in pediatric intensive care units. N Engl J Med 2007;356:1609–19.

**Reference 2**

Galland A, Tucci M, Leteurtre S, et al. Trend over three decades of the stated practice pattern on the hemoglobin (Hb) threshold that would guide red blood cell (RBC) transfusion practice in pediatric intensive care units, on behalf of the CCCTG, PCCS, JI.

**Compliance with ethics regulations:** Yes in clinical research.

**Table 1 (abstract FC-226):**
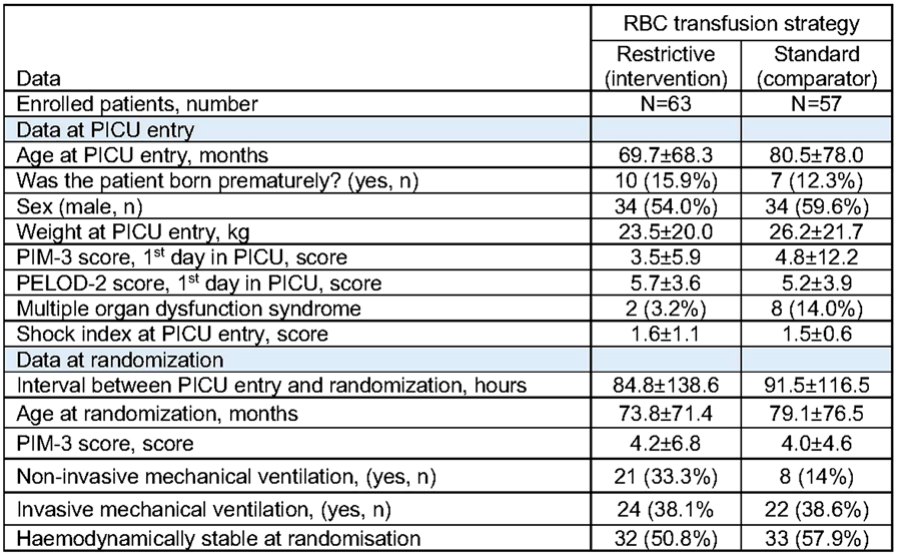
Baseline data at PICU entry and at randomization. PELOD: pediatric logistic organ dysfunction; PICU: pediatric intensive care unit; PIM: pediatric index of mortality; RBC: red blood cell

## FC-227 A comparison of critically ill children hemoglobin measures from the hematology laboratory vs. arterial blood gases

### Ishak Tamani^1^, Jacques Lacroix^1^, Michael Sauthier^1^

#### ^1^Division of Pediatric Critical Care Medicine, Department of Pediatrics, CHU Sainte-Justine, Université de Montréal, Montréal, Canada

##### **Correspondence:** Ishak Tamani (ishak.tamani@umontreal.ca)

*Annals of Intensive Care* 2023, **13(Suppl 1):**FC-0227

**Rationale:** The decision to prescribe a red blood cell (RBC) transfusion is frequently based on the hemoglobin concentration (Hb). Therefore, getting a reliable measurement of Hb is very important in transfusion medicine. The Hb can be measured in the hematology laboratory or through blood gases. It is frequently said that the difference between the results obtained by these two methods can be clinically significant, but hard data on this statement are missing. This study aimed to determine if there is a clinically and/or statistically significant difference in the results of Hb measurement made in the hematology laboratory and blood gases.

**Patients and methods/materials and methods:** Using the data prospectively collected in the electronic medical data monitoring system (eMDMS) of the multidisciplinary hospital’s PICU, we compared the results of Hb concentration measured in the hematology laboratory and in blood gazes that were collected simultaneously or within a 15-min interval. Only one comparison per patient was done using the first sampling through an arterial catheter that happened after the PICU entry. Patients with a life-threatening hemorrhage were excluded. Bland and Altman method was used for statistical analysis.

**Results:** 1702 critically ill children with a Hb measure in the PICU who were consecutively hospitalized in the PICU from January 1, 2013, to June 30, 2022, were included. The mean difference between the Hb measured with arterial blood gases and the Hb value measured in the hematology laboratory was 1.4 g/L. The limits of agreements were from − 9.2 g/L and 12.0 g/L (Figure 1).

**Conclusion:** The Hb measured using arterial blood gases is a clinically unbiased measure. The limits of agreement are probably precise enough to make the Hb measured by blood gas a good estimator to safely guide RBC transfusion therapy.

**Reference 1**

Bland JM, Altman DG. Statistical methods for assessing agreement between two methods of clinical measurement. Lancet 1886;1:307–10.

**Compliance with ethics regulations:** Yes in clinical research.

**Figure 1 (abstract FC-227):**
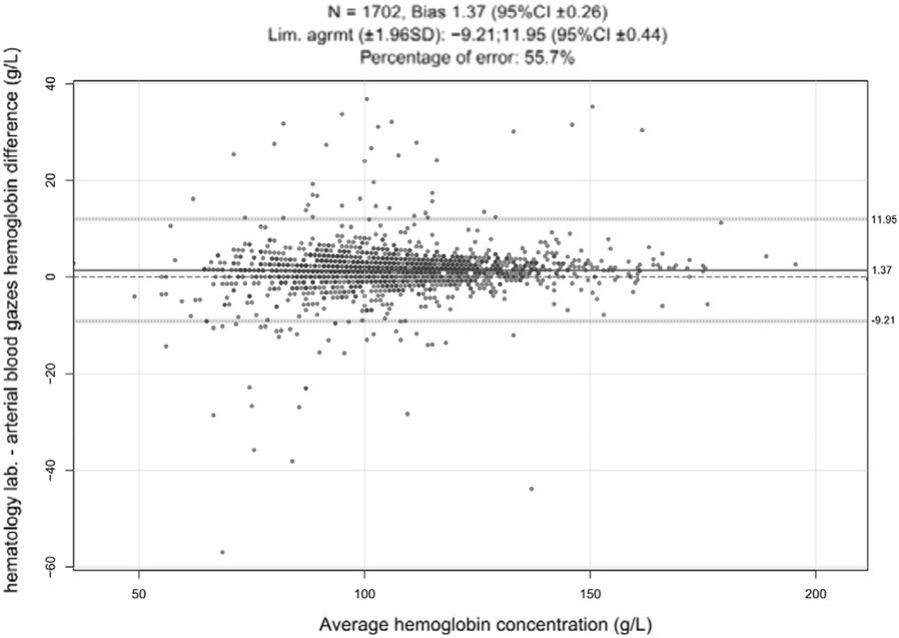
Bland–Altman plot between the Hb concentration acquired by the hematology laboratory and the arterial blood gazes among 1702 critically ill children; upper and lower lines represent the 95% limits of agreements

## FC-228 Iatrogenic anemia in severely burned patients

### Rania Hidri^1^, Iheb Glenza^1^, Safa Ben Mansour^1^, Hana Fredj^1^, Sarra Ben Zarrouk^1^, Imen Jami^1^, Bahija Gasri^1^, Manel Ben Saad^1^, Amel Mokline^1^, Messadi Amen^1^

#### ^1^centre de traumatologie et des grands brulés de Ben Arous, Tunis, Tunisie

##### **Correspondence:** Rania Hidri (rannouch.hidri6@gmail.com)

*Annals of Intensive Care* 2023, **13(Suppl 1):**FC-0228

**Rationale:** Background: Iatrogenic anemia due to phlebotomy-related blood loss, is common in intensive care units and continues harming far too many critically ill patients. In severely burned patients, it is multifactorial, mainly related to hemorrhage during surgical procedures and repeated blood loss (diagnostic blood sampling, invasive procedures, etc.). We aimed to study the characteristics of iatrogenic anemia and its impact on morbidity and mortality in severely burned patients.

**Patients and methods/materials and methods:** Methods: This retrospective study was conducted in the intensive burn care department in Tunisia during 6 months (From January 1st to June 2020). Were included adult burn patients who developed anemia during hospitalization (hemoglobin (Hb) < 13 g/dl for men and Hb < 12 g/dl for women), apart from active bleeding related to surgical interventions and/or digestive hemorrhage. Anemia was considered severe if (Hb) < 6 g/dl. For each patient, were recorded demographics, clinical, biological, therapeutic (including blood transfusion), and evolutionary characteristics. Invasive procedures performed on these patients and the volumes of blood spoliation were analyzed.

**Results:** Results: During the study period, 60 patients were included. The average age was 40 years ± 16 with a sex ratio of 1.16. No medical history was reported in 51% of cases. The total burn skin area (TBSA) was 35% ± 16. Invasive procedures performed in our patients were: arterial catheterization (n = 18) with an average duration of 4 days; hemodynamic monitoring by Volume View (n = 4) and hemodiafiltration requirement (n = 5). The total blood volume collected was 246 mL for patients with TBSA above 20% versus 138 mLfor those with TBSA below this threshold. This blood spoliation was more significant in burns with arterial catheterization (266 mL ± 98 ml vs. 189 mL ± 72; p = 0.04). In univariate analysis, our study showed that acute renal failure was associated with a significant risk of developing severe anemia (p < 10^−3^). The higher the amount of blood collected, the greater the risk of developing severe anemia (p = 0.026) with a cutoff of 98 ml of blood volume (sensitivity = 94.6%/specificity = 73.9%). The total amount of blood collected from our patients was 13,727 mL, with a quantity of an entire blood transfused at 10,100 mL.

**Conclusion:** In severely burned patients, phlebotomy-associated iatrogenic blood loss anemia was serious especially in the presence of arterial catheterization. So, efforts to optimize both the frequency and the volume of tests will prevent wasting huge amounts of blood, reduce blood transfusion and improve clinical outcomes of patients in intensive care units all over the world.

**Compliance with ethics regulations:** N/A.

## FC-229 Anemia and iron deficiency in children that survive a critical illness

### Li Shu Yin Han^1^, Geneviève Du Pont-Thibodeau^1^, Laurence Ducharme-Crevier^1^, Camille Jutras^1^, Kostas Pantopoulos^2^, Catherine Farrell^1^, Nadia Roumeliotis^1^, Karen Harrington^1^, Céline Thibault^1^, Noémi Ba Roy^3^, Akshay Shah^3^, Jacques Lacroix^1^, Simon Stanworth^3^

#### ^1^Department of Pediatrics, CHU Sainte-Justine, Université de Montréal, Montreal, Canada; ^2^Lady Davis Institute for Medical Research, Jewish General Hospital, McGill University, Montreal, Canada; ^3^Department of Hematology, John Radcliffe Hospital, Oxford University, Oxford, Royaume-Uni

##### **Correspondence:** Li Shu Yin Han (li.shu.yin.han@umontreal.ca)

*Annals of Intensive Care* 2023, **13(Suppl 1):**FC-0229

**Rationale:** Many children are discharged from pediatric intensive care units (PICUs) with anemia. The causes of anemia have been poorly described and investigated, and anemia-specific treatment plans are rarely offered in clinical practice. The purpose of this study was to evaluate the contributions of iron deficiency/iron-responsive anemia (ID) vs. anemia of inflammation (AI) to post-PICU anemia in the pediatric population. The specific objectives of this study are (1) to describe the changes in a range of markers of iron metabolism at PICU discharge, 2 months, and 6 months post-PICU; (2) to apply diagnostic algorithms for ID and AI; (3) to assess the different causes of anemia and (4) to determine a cut-off value for hepcidin (iron-marker) for identification of ID in patients with inflammation.

**Patients and methods/materials and methods:** Prospective cohort study at single site PICU. We included children requiring invasive ventilation for ≥ 48 h or non-invasive ventilation for ≥ 96 h. Children with pre-existing hematologic disorders, renal failure and/or post-operative congenital cardiac surgery were excluded. Complete blood count, inflammatory markers, hepcidin and iron profiles were determined at PICU discharge, and at 2 and 6 months post-PICU if patients were anemic at the previous appointment.

**Results:** Fifty-six children participated; 59% were aged less than 2 years. Median PELOD-2 score was 11 (IQR:5). Sixty-six percent were anemic at discharge, 7% at 2 months and 3.5% at 6 months. The proportion of children with anemia of inflammation decreased from discharge to 2 months (14.8% to 4.1%). The proportion of children with ID increased from 6.3% at discharge to 58.2% and 71.3% at 2 and 6 months, respectively. Using ROC curves, a cut-off value for hepcidin of 31.9 pg/mL was identified for the diagnosis of ID during inflammation. This increased the prevalence of ID at discharge from 6 to 25%.

**Conclusion:** Although anemia resolved in most PICU children within 2 months following PICU discharge, 58% developed ID. Hepcidin may improve the diagnostic yield of ID in patients with underlying inflammation and help develop better-targeted strategies for anemia therapy.

**Reference 1**

Lasocki S, Asfar P, Jaber S, Ferrandiere M, Kerforne T, Asehnoune K, et al. Impact of treating iron deficiency, diagnosed according to hepcidin quantification, on outcomes after a prolonged ICU stay compared to standard care: a multicenter, randomized, si.

**Reference 2**

Shah A, Wray K, James T, Shine B, Morovat R, Stanworth S, et al. Serum hepcidin potentially identifies iron deficiency in survivors of critical illness at the time of hospital discharge. Br J Haematol. 2019;184(2):279–81.

**Compliance with ethics regulations:** Yes in clinical research.

**Figure 1 (abstract FC-229):**
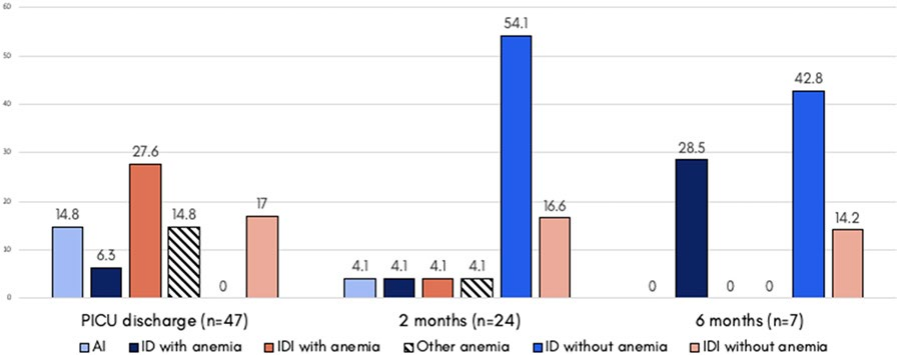
Types of anemia and iron profiles at PICU discharge, and 2 and 6 months post-PICU

## FC-230 Extra-cerebral anticoagulant related bleeding accidents admitted to intensive care units: a French multicenter retrospective study 2007–2018

### Thomas Botrel^1^, Julie Helms^2^, Sibylle Cunat^2^, Jeremie Lemarie^3^, Sébastien Preau^4^, Raphael Favory^4^, Florence Boissier^5^, Jeremie Joffre^1^, Hafid Ait Oufella^1^

#### ^1^Hôpital Saint Antoine, Paris, France; ^2^Hôpital Universitaire de Strasbourg, Strasbourg, France; ^3^Hôpital Universitaire de Nantes, Nantes, France; ^4^Hôpital Régional Universitaire de Lille, Lille, France; ^5^Hôpital Universitaire de Poitiers, Poitiers, France

##### **Correspondence:** Thomas Botrel (botrel.thomas@gmail.com)

*Annals of Intensive Care* 2023, **13(Suppl 1):**FC-0230

**Rationale:** Anticoagulants are widely used but responsible for iatrogenic events such as bleeding. Very few data are available regarding the characteristics and management of anticoagulant-treated patients admitted to intensive care units (ICU) for severe bleeding.

**Patients and methods/materials and methods:** A retrospective observational study was conducted in 5 french ICUs. From January 2007 to December 2018, all patients over the age of 18, admitted to intensive care units for extracerebral bleeding and receiving curative anticoagulation, were included.

**Results:** A total of 486 patients were included in 5 French intensive care units. The average age was 73 ± 13 years, 39% were women and most of the patients had comorbidities (arterial hypertension 68%, heart disease 49%, diabetes 33%). The anticoagulants responsible for the bleeding were vitamin K antagonists (VKA: 54%), heparins (25%) and direct oral anticoagulants (DOAC: 7%). The incidence of bleeding increased during the 2007–2018 period, from 3.2/1000 admissions in 2007 to 5.8/1000 admissions in 2018, with stability in VKA group but an increase in patients receiving DOACs. Mortality in intensive care was 27%. In multivariate analysis, factors associated with mortality were: Glasgow Coma Scale, hyperlactatemia, ventilatory support and a modified a PTT at admission. The medical management of bleeding and in particular antagonization was not optimal, the use of vitamin K and prothrombin complex concentrates was effective in only 60% of patients treated with VKA, and use of prothrombin complex concentrates was found in only 29% of patients treated with DOACs.

**Discussion:** The incidence of anticoagulation related bleeding is increasing as the prescription of anticoagulants rises. Bleeding occurred in comorbid patients, characteristics being different according to the type of anticoagulants. Mortality is strongly associated with organ failures (mainly circulatory, respiratory, neurological and hematological). Despite its high mortality, the management of anticoagulant related bleeding is not optimal. Knowledge about anticoagulant-associated bleeding and adherence to clinical therapeutic guidelines must be improved.

**Conclusion:** Anticoagulant related extra-cerebral bleeding admitted to ICU are serious complications responsible for organ failure and significant mortality. Their incidence is increasing. Therapeutic management of these accidents must be improved.

**Reference 1**

Hauguel M, Boelle P, Pichereau C, Bourcier S, Bigé N, Baudel J, et al. Severe extra-cerebral anticoagulant-related bleeding in intensive care unit: a retrospective study from 2000 to 2013. Medicine (Baltimore). 2015;94(47):e2161.

**Reference 2**

Tremey B, Tazarourte K, Ract C, Gabteni M, Lavagna L, Dépret-Vassal J, et al. Teaching improves adherence to clinical guidelines in the treatment of oral anticoagulation-related severe bleeding in the emergency department. Intensive Care Med. 2009;35.

**Compliance with ethics regulations:** Yes in clinical research.

**Figure 1 (abstract FC-230):**
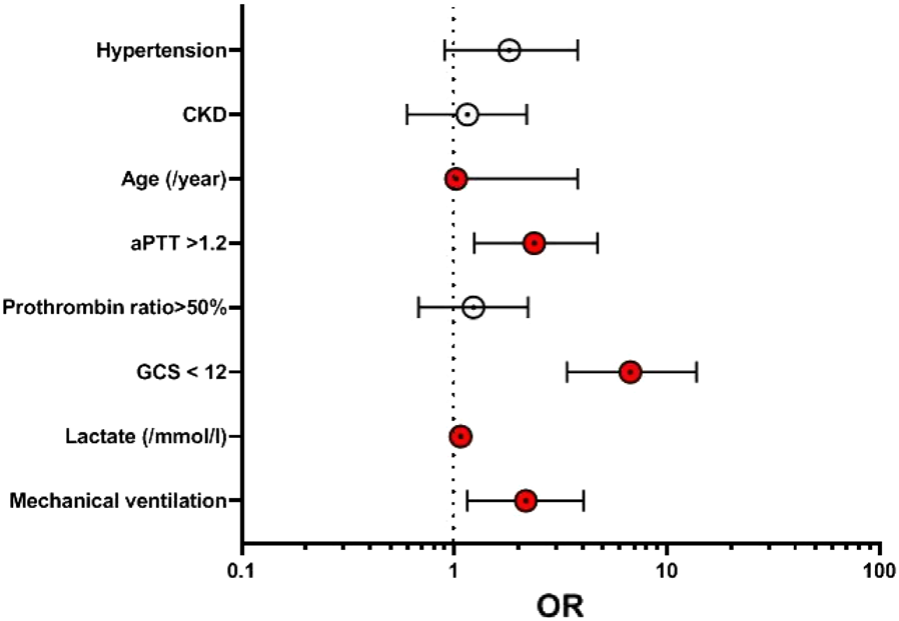
OR for mortality of bleeding events (multivariable analysis)

## FC-231 Heparin monitoring during extracorporeal membrane oxygenation: the effect of dextran sulfate on anti-Xa assay

### Emna Hammami^1^, Laure Stiel^1^, Clément Palpacuer^1^, Ines Harzallah^1^

#### ^1^Hôpital Emile Muller, Groupe Hospitalier de Mulhouse Sud Alsace, Mulhouse, France

##### **Correspondence:** Laure Stiel (lau57@hotmail.fr)

*Annals of Intensive Care* 2023, **13(Suppl 1):**FC-0231

**Rationale:** Management of Extracorporeal membrane oxygenation (ECMO) requires the use of systemic anticoagulation. Although there is no uniform ECMO anticoagulation practice, unfractionated heparin (UFH) monitored by Anti-Xa assay is widely used. Nevertheless, anti-Xa reagents differ between ECMO centers. Most of the reagents contain dextran sulfate (DXS), added to prevent heparin from binding to protein other than antithrombin like platelet factor 4 (PF4). PF4 release is increased in some pathological situations, notably under ECMO. This study aimed to compare agreement of anti-Xa activity between two reagents, with (D-Anti Xa) and without DXS (ND-Anti Xa) during ECMO treatment.

**Patients and methods/materials and methods:** This monocentric retrospective study was conducted between May 2020 and November 2021 in a French Intensive Care Unit. Patients under ECMO anticoagulated with UFH and monitored with both D-Anti Xa and ND-Anti Xa were included. The study was approved by the local ethical committee.

**Results:** Thirteen patients were enrolled and 177 pairs of anti-Xa assay were performed. Nine patients experienced bleeding and five thrombosis. Median anti-Xa value measured with D-Anti Xa (0.41 IU/mL) was higher than with ND-Anti-Xa assay (0.32 IU/mL) with a “mean bias” of the Bland and Altman plot of + 0.062 IU/mL (Figure 1). Lin’s concordance correlation coefficient was 0.89. The therapeutic range for Anti-Xa tests was fixed at 0.2 to 0.5 IU/mL according to recommendations. Forty-four discrepant pairs of anti-Xa levels (24.9%) were observed.

**Discussion:** We herein report discrepant anti-Xa assay results using two reagents, with and without DXS, illustrating the ongoing challenge of monitoring anticoagulation during ECMO. Although DXS is added to anti-Xa reagents to promote stability, it could induce an overestimation of heparin level that does not reflect the in vivo anticoagulant effect of heparin. Indeed, anti-Xa assay does not reflect the clot formation, and there is no data demonstrating that specific anti-Xa targets improve outcomes in patients under ECMO. The design of this small sample size’ study, and the diversity in underlying diseases, do not allow to demonstrate clinical impact of the biological differences.

**Conclusion:** These results suggest an overestimation of heparin concentration with D-Anti-Xa assay that could lead to inadequate estimation of bleeding and thrombosis risk. The results of anti-Xa assay should be interpretated with caution by clinicians in situations with suspected high circulating PF4 levels like during ECMO, and other clinical and biological parameters should be considered to evaluate the in vivo coagulation state.

**Compliance with ethics regulations:** Yes in clinical research.

**Figure 1 (abstract FC-231):**
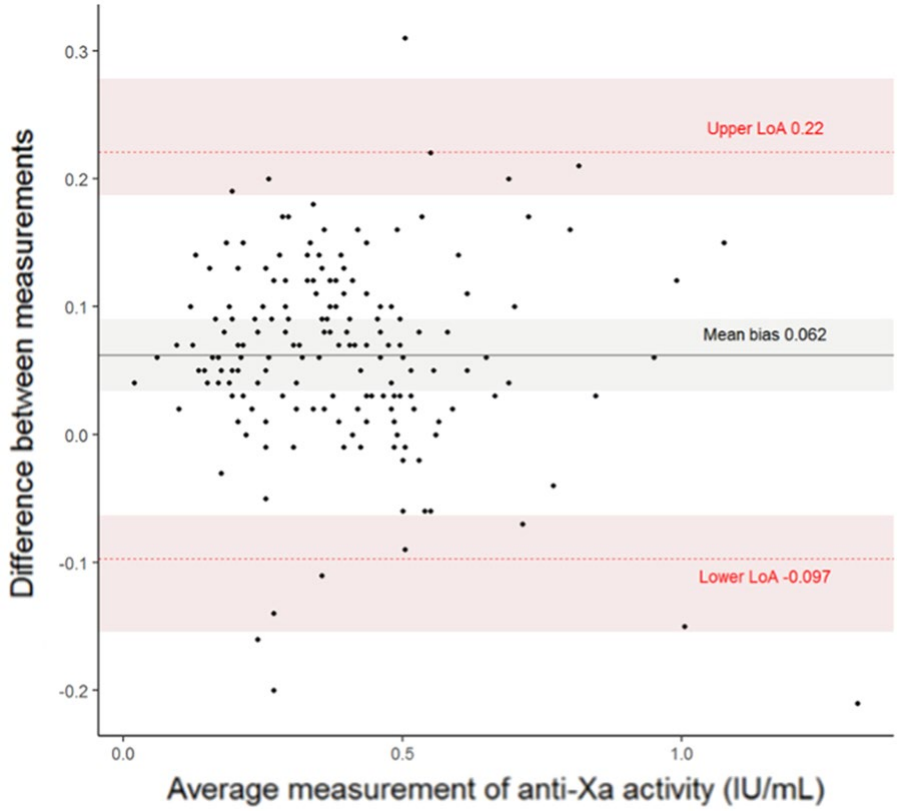
Altman Bland graphic. The difference between measurements was calculated as: anti-Xa assay with dextran—anti-Xa assay without dextran

## FC-232 Pulmonary embolism in COVID-19 patients: about to 12 cases in intensive care unit at the Centre Hospitalier Universitaire de Libreville

### Laurence Essola^1^, Arsène Ifoudji Makao^1^, Luc Bitegue Methe^1^, Fernande Manga^1^, Adrien Sima Zue^1^

#### ^1^Centre hospitalier universitaire de Libreville, Libreville, Gabon

##### **Correspondence:** Laurence Essola (laurenceessola@yahoo.fr)

*Annals of Intensive Care* 2023, **13(Suppl 1):**FC-0232

**Rationale:** The complications observed during COVID-19 are mostly related to venous thromboembolic disease and the most common of these is pulmonary embolism (PE). The aim of this work was to identify the main risk factors for the occurrence of PE in patients admitted for severe COVID-19 pneumonia in intensive care at the University Hospital of Libreville (CHUL).

**Patients and methods/materials and methods:** This was a retrospective, descriptive and analytical study from April 2020 to March 2021 in patients admitted for severe COVID-19 pneumonia and PE during hospitalization. The parameters studied on admission were socio-demographic, clinical and paraclinical data. Two groups were compared: PE (+) and PE (−) to determine the main risk factors for PE.

**Results:** During the study period, 12 out of 141 patients had PE, a frequency of 8.5%. The mean age was 47.9 ± 15.5 years and the sex ratio was 1. Hypertension was found alone in 7 patients (58.3%). The mean BMI of PE patients (+) was 31.3 ± 7.8 kg/m^2^ versus 29.4 ± 6.3 kg/m^2^ for PE (−) patients. The mean D-dimer level was 20.5 ± 48.9 ng/l for PE (+) versus 5.1 ± 5.1 ng/l for PE (−). On electrocardiogram, subepicardial ischemia was found in 6 patients (50%). On thoracic CT angiography, PE was subsegmental in 7 patients (58.3%) and massive in 3 (25%) of them. The mean value of the Wells score was 9.4 ± 1.4 with extremes of 7.5 and 11.5 for PE (+) versus 7.0 ± 2.9 with extremes of 0 and 11.5 for PE (−). The mean value of the sPESI score was 116.7 ± 34.8 with extremes of 71 and 202. The q SOFA score averaged 1.7 ± 0.8 for PE (+) patients and 1.1 ± 0.7 for PE (−) patients. The mean Brescia score was 2.7 ± 0.6 for PE patients (+) and 2.8 ± 0.8 for PE (−). Therapeutically, mechanical ventilation was required in 2 PE patients (+). Only one patient (7.7%) had thrombolysis. The mean length of hospital stay was 9.0 ± 6.7 days for PE patients (+) versus 8.2 ± 6.2 days for PE (−). Death was recorded in 3 PE patients (+). In bivariate analysis, tobacco use was a risk factor for PE (p < 0.001). By multivariate analysis, respiratory signs (p = 0.019 OR = 12.1 (95% CI = [1.5–97.5]) and impaired consciousness at admission (p = 0.007, OR = 27.6 (95% CI = [2.5–311.6]) were associated with PE.

**Conclusion:** This study has shown that PE is a complication that affects young adults. The main risk factors for PE are tobacco use, respiratory parameters and impaired consciousness at admission.

**Compliance with ethics regulations:** Yes in clinical research.

## FC-233 Healthcare-associated infections prevalence in medical ICUs in Tunisia: results of the multi-centre NOSOREA2 study

### Amira Jamoussi^1^, Nacef Ben Mrad^1^, Najeh Baccouche^2^, Mounir Bouaziz^2^, Ines Sedghiani^3^, Nabiha Borsali Falfoul^3^, Hana Fredj^4^, Amenallah Messaadi^4^, Asma Hamdi^5^, Sami Abdellatif^5^, Emna Ennouri^6^, Mohamed Boussarssar^6^, Ines Fathallah^7^, Nadia Kouraichi^7^, Khaoula Ben Ismail^8^, Takoua Merhebene^8^, Amira Ben Jazia^9^, Nozha Brahmi^9^, Youssef Blel^10^, Kais Ben Romdhane^10^, Hajer Nouira^11^, Souheil El Atrous^11^, Jihen Ayachi^12^, Hend Allouche^13^, Hatem Ghadhoun^13^, Rafla Ben Dabebiss^14^, Houssem Hamouda^14^, Fatma Kaaniche^15^, Houda Mateur^16^, Nasreddine Foudhaili^17^, Fatma Jarraya^1^, Emna Rachdi^1^, Samia Ayed^1^, Jalila Ben Khelil^1^

#### ^1^Hôpital Abderrahmen mami, Tunis, Tunisie; ^2^Hôpital Habib Bourguiba, Sfax, Tunisie; ^3^Hôpital Habib Thameur, Tunis, Tunisie; ^4^Centre de traumatologie et des grands brûlés, Ben Arous, Tunis, Tunisie; ^5^Hôpital la Rabta, Tunis, Tunisie; ^6^Hôpital Farhat Hached, Sousse, Tunisie; ^7^Hôpital régional Yasminet Ben Arous, Tunis, Tunisie; ^8^Hôpital régional Zaghouan, Zaghouan, Tunisie; ^9^Centre d'assistance médicale urgente, Tunis, Tunisie; ^10^Clinique Carthagène, Tunis, Tunisie; ^11^Hôpital Tahar Sfar, Mahdia, Tunisie; ^12^Hôpital régional Ibn El Jazzar, Kairouan, Tunisie; ^13^Hôpital Habib Bougatfa, Bizerte, Tunisie; ^14^Hôpital Sahloul, Sousse, Tunisie; ^15^Hôpital régional de Mahrès, Sfax, Tunisie; ^16^Hôpital Régional Hedi Jaballah, Tozeur, Tunisie; ^17^Hôpital régional du Kef, Le Kef, Tunisie

##### **Correspondence:** Amira Jamoussi (amira.jamoussi@fmt.utm.tn)

*Annals of Intensive Care* 2023, **13(Suppl 1):**FC-0233

**Rationale:** Healthcare-associated infections (HAI) in ICU are common and often associated with higher costs, work burden and morbi-mortality. Controlling this fatal scourge should begin with greater awareness of ICU ecology and HAI frequencies. In 2017, we conducted NOSOREA 1 study^(1)^: 15 participating ICUs, 103 patients, HAI prevalence = 25.2% CI 95% [15–35]. Five years later, after COVID-19 pandemic, we propose NOSOREA 2 to compare these previous findings. OBJECTIVE: We aimed to determine the prevalence of HAI in Tunisian ICUs, identify the predominant infecting organisms, and evaluate risk factors of HAI.

**Patients and methods/materials and methods:** Design: a 1-day point-prevalence study (September 27th, 2022). Setting: Medical intensive care units in Tunisia, excluding cardiologic and paediatric care units. Patients: all patients hospitalized in ICU were enrolled. Main outcome measures: prevalence, types of HAI and resistance patterns of microbiological isolates. This study was conducted under the aegis of the ‘Association Tunisienne de réanimation (ATR)’ NOSOREA2 is registered with ClinicalTrials.gov NCT05547646.

**Results:** On the day of the study, 17 centres participated, 143 patients were enrolled with a median age of 53.6 years and a gender-ratio of 1.8. Main comorbidities were hypertension (n = 40, 28%), diabetes (n = 34, 23.8%) and COPD (n = 21, 14.7%). Reasons for ICU admission were: neurological (n = 56, 39%), respiratory (n = 43, 14%) and haemodynamic (n = 20, 14%). COVID-19 vaccination was noticed among 78 (54.5%) patients. We recorded 46 documented HAI, making a 1-day point-prevalence of 32.1 [25.1–39.1]. HAI sites were: VAP (n = 19), VAT (n = 2), NV-HAP (n = 2), BSI (n = 7), central line BSI (n = 6), urinary (n = 9) and cutaneous (n = 1). Main identified microorganisms were: K*lebsiella pneumonia* (n = 11), *Acinetobacter baumanii* (n = 6) and P*seudomonas aeruginosa* (n = 6). Characteristics comparison between HAI and non-HAI patients is shown in Table I.

**Conclusion:** In 2022, the 1-day point-prevalence of HAI in Tunisian ICUs was of 32.1 [25.1–39.1]. It was higher than 2017. Most prevalent microorganisms were *Klebsiella pneumonia* and non-fermenting pathogens. The only identified risk factor associated to HAI occurence was invasive mechanical ventilation.

**Reference 1**

Jamoussi A, Ayed S, Ismail KB. The prevalence of healthcare-associated infection in medical intensive care units in Tunisia. Results of the multi-centre nosorea1 study. Tunis Med. 2018;96(10e11):731e6.

**Compliance with ethics regulations:** Yes in clinical research.

**Table 1 (abstract FC-233):**
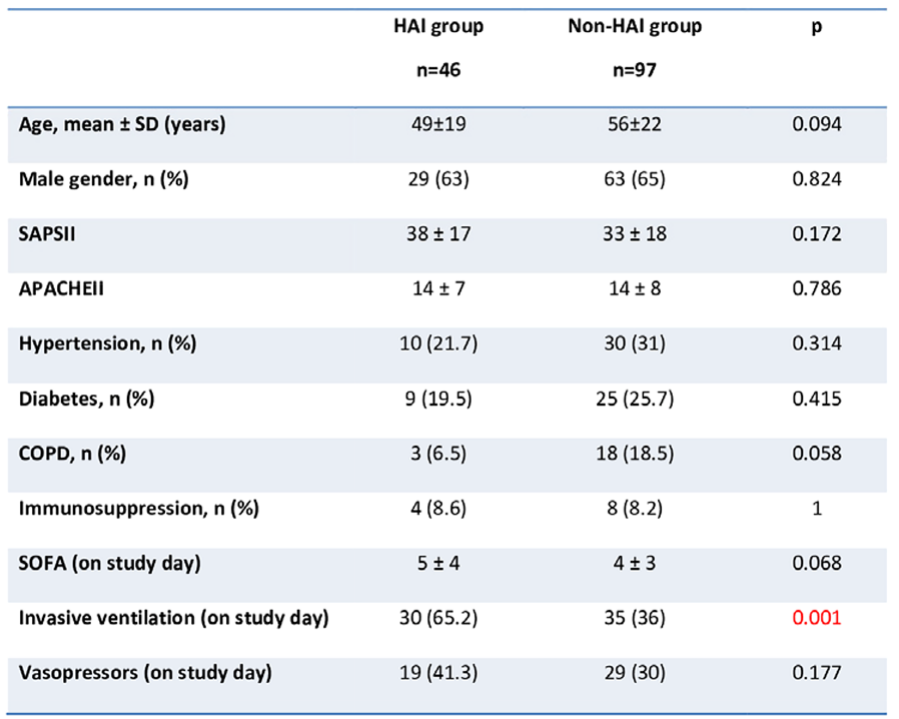
Comparison between HAI and non-HAI patients

## FC-234 Prevalence and risk factors of ventilator-associated pneumonia (VAP) in an intensive care unit in Algeria

### Abdelkader Azza^1^, Soumia Benbernou^1^, Amina Khiali^1^, Houria Mokhtari Djebli^1^

#### ^1^Faculté de médecine, Oran, Algerie

##### **Correspondence:** Abdelkader Azza (azza.abdelkader@univ-oran1.dz)

*Annals of Intensive Care* 2023, **13(Suppl 1):**FC-0234

**Rationale:** Pneumonia acquired in intensive care (ICU) are the first nosocomial infections in terms of frequency, and mortality. They are associated with an increase in morbidity and mortality, as well as an extension of the length of stay and therefore an additional cost. This is a serious infection complicating the prognosis of the patient, mainly associated with ventilation (ventilatory associated pneumonia, VAP).

**Patients and methods/materials and methods:** Prospective study between 2017 and 2018, performed in the intensive care unit of the university hospital in Oran. Our aim was to analyze risk factors of VAP. Data analysis was made using SPSS software, version 23.

**Results:** During the study period, 384 patients were hospitalized and ventilated in the department, among them 81 patients developed VAP (21.1%), among which 1/3 were early VAPs. Chronic obstructive pulmonary disease multiplies the risk of VAP by 7 (p < 0.001) Tobacco multiplies it by 3.5 (p = 0.007). Reintubation multiplies the risk by 11.8 (p < 0.001). Inhalation multiplies the risk by 6.7 (p < 0.001).

**Conclusion:** VAPs are a major complication of ICU hospitalization. IDentifying the main risk factors could help to control the modifiable risk factors, using appropriate bundles.

**Compliance with ethics regulations:** Yes in clinical research.

## FC-235 Prediction of invasive pulmonary aspergillosis in patients with ventilator associated pneumonia with an easy to use score (PIPA-VAP score)

### Nicolas Massart^1^, Florian Reizine^2^, Emma Plainfosse^3^, Clarisse Dupin^1^, François Legay^1^, Anne Cady^2^, Guillaume Rieul^2^, Nicolas Barbarot^1^, Eric Magalhaes^1^, Pierre Fillatre^1^

#### ^1^Centre Hospitalier Yves Le Foll, Saint-Brieuc, France; ^2^CH de Vannes, Vannes, France; ^3^CHU Rennes- Pontchaillou, Rennes, France

##### **Correspondence:** Nicolas Massart (nicolasmassart@hotmail.fr)

*Annals of Intensive Care* 2023, **13(Suppl 1):**FC-0235

**Rationale:** Invasive pulmonary aspergillosis (IPA) is an opportunistic condition, thought to occur mostly in immunocompromised patients, whose prevalence has lately been reported as high among patients with suspected ventilator-associated pneumonia (VAP) (1, 2). Because risk factors for IPA in critically-ill patients are not well established, it is a commonly missed diagnosis and anti fungal therapy is often delayed, worsening the prognosis. We aim to develop a clinical score for prediction of ICU acquired IPA among patients suspected of VAP.

**Patients and methods/materials and methods:** A retrospective observational study was conducted in 2 ICUs in Bretagne, western France. Patients who developed VAP from January 1, 2020 until 31, December 2021 were eligible. The study protocol received approval from the Rennes Hospital ethics committee (Comité d’Éthique du CHU de Rennes, avis 19–52). All respiratory samples sent for bacteriological analysis were also cultured in a dedicated fungal media for *Aspergillosis* screening. Patients were classified as having possible, putative, probable or proven aspergillosis according to the AspICU (2), IAPA and CAPA criteria when indicated. IPA was considered as ICU-acquired only in patients without microbiological or suggestive radiological findings in the first 48 h of stay. The score was constructed with the variables associated with IPA in multivariable logistic regression model with a p-value < 0.2.

**Results:** There were 121 patients with VAP during study period, of whom 20 had IPA (6 possible IPA and 14 probable IPA). Patients with IPA were more often male (100% vs 76.2%, p = 0.033), have received more frequently immunomodulatory treatments (30.0% vs 5.9%, p = 0.004), low dose steroids (25.0% vs 6.9%, p = 0.028), had a lower lymphocyte count at admission (0.50 [0.29–0.72] vs 0.84 G/L [0.56–1.28], p = 0.005) and a lower PaO_2_/FiO_2_ ratio (115 mmHg [89–132] vs 133 [100–205], p = 0.021). Variables composing the score were immunocompromised patients (2 points), lymphocytes count < 0.8 G/L (2 points), PaO_2_/FiO_2_ ratio < 130 mmHg (1 point) and VAP onset > 10 days (1 point). Area under the receiving operator curve was 0.817. Incidence of IPA was low (3.2%) among patients with low risk (score 0–2), intermediate (23.9%) in patients with moderate risk (risk 3–4) and as high as 50% in patients with high risk (5–6) (Figure).

**Conclusion:** We have developed a clinical score, with good predictive value, which may help to predict IPA in patients with VAP. An external validation cohort is needed to confirm our results.

**Reference 1**

Loughlin L, Hellyer TP, White PL, McAuley DF, Conway Morris A, Posso RB, et al. Pulmonary aspergillosis in patients with suspected ventilator-associated pneumonia in UK ICUs. Am J Respir Crit Care Med. 2020;202(8):1125–1132. https://doi.org/10.1164/rccm.20200

**Reference 2**

Blot SI, Taccone FS, Van den Abeele AM, Bulpa P, Meersseman W, Brusselaers N, et al. A clinical algorithm to diagnose invasive pulmonary aspergillosis in critically ill patients. Am J Respir Crit Care Med. 2012;186(1):56–64. https://doi.org/10.1164/rccm.201111

**Compliance with ethics regulations:** Yes in clinical research.

**Figure 1 (abstract FC-235):**
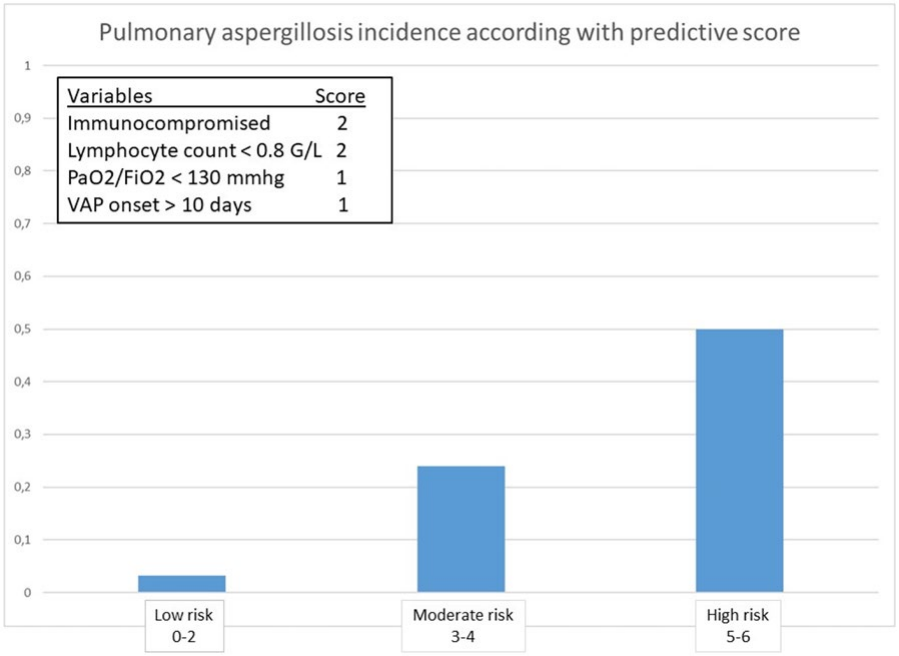
Altman Bland graphic. The difference between measurements was calculated as: anti-Xa assay with dextran—anti-Xa assay without dextran

## FC-236 Impact of closed versus open tracheal suctioning systems on the incidence of ventilator-acquired pneumonia and outcome in ventilated patients

### Ahlem Trifi^1^, Eya Seghir^1^, Asma Mehdi^1^, Lynda Masseoud^1^, Salma Rabhi^1^, Sami Abdellatif^1^

#### ^1^Medical ICU, Hôpital la Rabta, Tunis, Tunisie

##### **Correspondence:** Ahlem Trifi (trifiahlem2@gmail.com)

*Annals of Intensive Care* 2023, **13(Suppl 1):**FC-0236

**Rationale:** In ventilated intensive care patients, the endo-tracheal suctioning (ETS) maneuver is essential to eliminate secretions and clear the airways to facilitate ventilation. However, it has major risks, first and foremost contamination of the respiratory tract and ventilator-associated pneumonia (VAP). One of the tools that would be associated with the reduction of VAP is the use of the closed suctioning system (CSS). We aimed to compare the occurrence of VAP in ventilated patients in addition to other parameters of outcome according to the used suctioning system: CSS versus open suctioning system (OSS).

**Patients and methods/materials and methods:** Open prospective randomized study over 5 months (August–December 2022). Adult patients admitted to intensive care who required invasive ventilation (IV) for more than 48 h were included. The exclusion criterion was the occurrence of death within 72 h of IV. A sample was taken by bronchial aspiration: on D3 of IV, on D5 and on D7. The primary endpoint was the incidence of VAP and the secondary endpoints were the outcome parameters (length of stay and death).

**Results:** We included 40 patients (CSS, n = 20 vs OSS, n = 20) and the two groups were similar for the clinical characteristics, severity scores and indication of intubation. All suggestive signs of VAP (fever, time of fever, purulent sputum, radiological new images) as well as the 3 times monitoring of P/F ratio, WBC and CRP didn’t show any difference except a lower WBC count at day 5 in OSS group (attached table). Overall, the proportion of positive cultures of endotracheal aspirates was considerable (56/120 = 47%) distributed in a similar way between the groups and between the 3 times. VAP was diagnosed in 22 cases with 13 (65%) in the CSS group and 9 (45%) in the OSS group with no statistically significant difference (p = 0.34). The other outcome parameters were also similar. All the studied parameters according to the used suctioning system are displayed in the attached table.

**Conclusion:** The use of CSS was not associated with the reduction of VAP. However, it is possible that the use of closed systems in combination with other care-related pneumonia prophylactic strategies provides a benefit.

**Compliance with ethics regulations:** Yes in clinical research.

**Table 1 (abstract FC-236):**
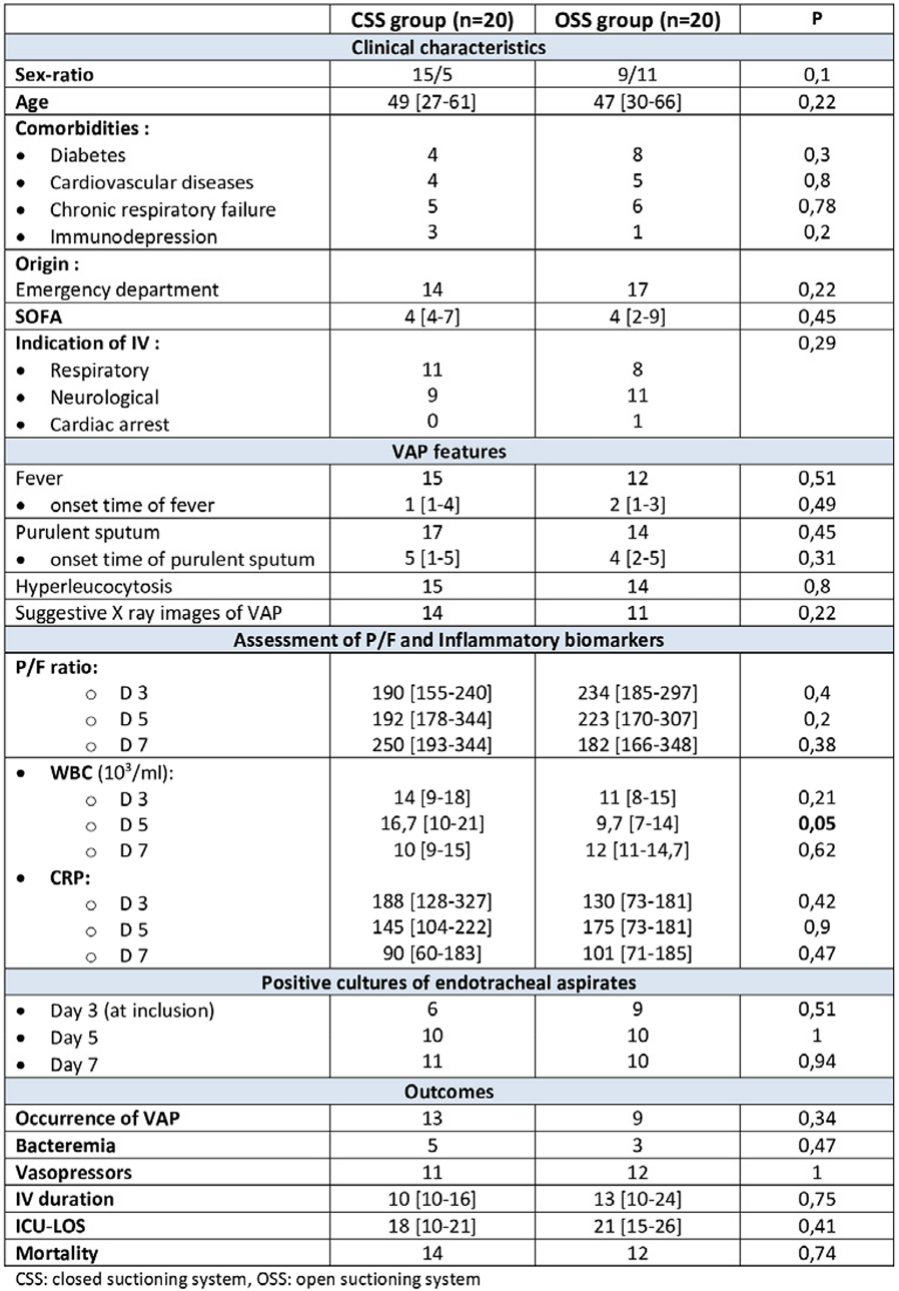
All studied parameters according to the used suctioning system

## FC-237 Predicting the transmission of multidrug-resistant bacteria and hospital acquired infection in ICU patients

### Clément Lejealle^1^, Pierre Moenne-Locoz^1^, Stéphane Ruckly^2^, Nesrine Boujelbene^1^, Jean-François Timsit^2^, Guillaume Gele-Decaudin^1^, Jean-Ralph Zahar^1^, Yves Cohen^1^

#### ^1^Hôpital Avicenne, Paris, France; ^2^Hôpital Bichat, Paris, France

##### **Correspondence:** Clément Lejealle (clejealle@gmail.com)

*Annals of Intensive Care* 2023, **13(Suppl 1):**FC-0237

**Rationale:** The spread and acquisition of multidrug resistant bacteria (MDRB), notably has major consequences in intensive care unit (ICU) patients. The transmission and acquisition of MDRB depends on individual factors (antibiotic selective pressure, exposure to invasive procedures) but also on collective factors such as colonization pressure and workload. The aims of this study was to assess the impact of health worker workload on extended-spectrum b-lactamase (ESBL)-producing Enterobacteriaceae (ESBL-PE) acquisition and ICU acquired infection in ICU patients.

**Patients and methods/materials and methods:** We performed a 6 months monocentric prospective study in a 24 beds medico-surgical ICU. All consecutive patients aged 18 years or older admitted in our ICU and staying at least 24 h were enrolled within 48 h of ICU admission. A rectal sample was collected within 48 h after admission, weekly thereafter and before the discharge from ICU. Health worker workload was assessed using the Nine Equivalents of Nursing Manpower Use Score (NEMS), an accepted tool for the measurement of nursing workload in the ICU.

**Results:** Four hundred and four stays were analyzed. Patients were mostly male (67%), median [IQR] age was 63 [53–73] years, and median SAPS2 score was 40 [28–54]. Eighty-one (20%) were ESBL-PE cases, including 55 (14%) imported cases and 28 (7%) acquisitions. Two patients had an ESBL-PE colonization at admission and acquired another ESBL-PE during their ICU stay. Two ventilation-associated pneumonia related to ESBL-PE were diagnosed. The health worker workload, assessed by NEMS, was not different as well as daily occupancy and the ratio of the healthcare workers to the number of patients. Only the use of central venous catheter and ICU length of stay was significantly more frequent in patients with ESBL-PE acquisition.

**Conclusion:** The workload of health workers was not associated with the acquisition of ESBL-PE in ICU in our study. The low incidence of acquired colonization and the absence of understaffing, avoiding an overload of work, limit the relevance of our results. Further larger scale studies are needed to accurately assess the role of ICU workload on MDRB acquisition.

**Compliance with ethics regulations:** Yes in clinical research.

## FC-238 Monitoring of immature granulocytes as a marker of ventilator-associated pneumonia—preliminary results

### Thomas Daix^1,2,3^, Robin Jeannet^1,4^, Julien Vaidie^3^, Anne-Laure Fedou^3^, Arnaud Desachy^3^, Ana C Hernandez^1,3^, Jean Feuillard^4,5^, Philippe Vignon^1,2,3^, Bruno François^1,2,3^

#### ^1^Inserm CIC 1435, CHU Dupuytren, Limoges, France; ^2^Inserm UMR1092, Faculté de Médecine, CHU Dupuytren, Limoges, France; ^3^Réanimation polyvalente, CHU Dupuytren, Limoges, France; ^4^CNRS UMR 7276, Faculté de Médecine, Université de Limoges, Limoges, France; ^5^Laboratoire d'hématologie, CHU Dupuytren, Limoges, France

##### **Correspondence:** Thomas Daix (thomas.daix@chu-limoges.fr)

*Annals of Intensive Care* 2023, **13(Suppl 1):**FC-0238

**Rationale:** During bacterial stress, neutrophils express activation markers such as CD64 (1). It has been demonstrated in mechanically ventilated patients with ARDS related to SARS-CoV-2 that the presence of circulating CD16low immature granulocytes (IGs) expressing the CD64 marker was related to bacterial co-infection (2). In this study, we aimed to determine in patients without pulmonary disease whether the emergence of this sub-population over time was associated with the development of ventilator-associated pneumonia (VAP).

**Patients and methods/materials and methods:** Immunocompetent patients without underlying pulmonary disease who were ventilated for at least 24 h for a traumatic brain injury (TBI) or a stroke were included in this prospective single-centre study. During the first week of hospital stay, expression of several markers on granulocytes (CD10, CD64, CD15, CD16, Lox-1, and PD-L1) and plasma concentration of 10 cytokines (CCL-2, G-CSF, GM-CSF, CXCL-8, IL-1ra, IL-6, IL-10, IP-10, TNF-α, and IFN-γ) were daily assessed. Two independent physicians blinded from the flow cytometry results and the cytokine concentrations adjudicated all patients and determined the date of onset of VAP or ventilator-associated tracheobronchitis (VAT), when applicable, according to clinical, radiological, biological data and predefined definitions.

**Results:** To date, 35 patients have been included (15 TBI, 20 strokes; median age: 58 ± 16 years; median Glasgow: 8 ± 4). After adjudication, 4 VAP and 7 VAT were confirmed in 11 patients (31%). In patients without pulmonary infection between day 0 and 7, the median of CD16low/CD64+IGs level was 0.37% [25–75 percentiles: 0.15–1.09], and no significant variation was observed during the follow-up (Figure). Similar results applied for patients who developed a VAT (median 0.28 [0.12–1.43]) (Figure). Regarding the 4 patients who developed a VAP, a low level of CD16low/CD64+IGs was also observed until day 7 (median 1.48 [0.24–5.85]). However, a peak of CD16low/CD64+IGs was observed within 1 day of VAP clinical diagnosis with values above the 75 percentile (36.71%, 36.23%, 38.23% and 7.36%) (Figure). None of the other granulocyte markers was associated with VAP diagnosis. The assessment of cytokine concentrations showed an increase of IL-6 > 1000 pg/mL and G-CSF > 160 pg/mL, at the time of CD16low/CD64 + IGs peak.

**Conclusion:** These preliminary results seem to associate the development of VAP with increased circulating CD16low/CD64+IGs. The recruitment of this sub-population is presumably related to the increased level of G-CSF which stimulates neutrophil production and CD64 expression.

**Reference 1**

Dimoula A, Pradier O, Kassengera Z, Dalcomune D, Turkan H, Vincent JL. Serial determinations of neutrophil CD64 expression for the diagnosis and monitoring of sepsis in critically ill patients. Clin Infect Dis. 2014;58(6):820–829.

**Reference 2**

Daix T, Jeannet R, Hernandez Padilla AC, Vignon P, Feuillard J, François B. Immature granulocytes can help the diagnosis of pulmonary bacterial infections in patients with severe COVID-19 pneumonia. J Intensive Care. 2021;9(1):58.

**Compliance with ethics regulations:** Yes in clinical research.

**Figure 1 (abstract FC-238):**
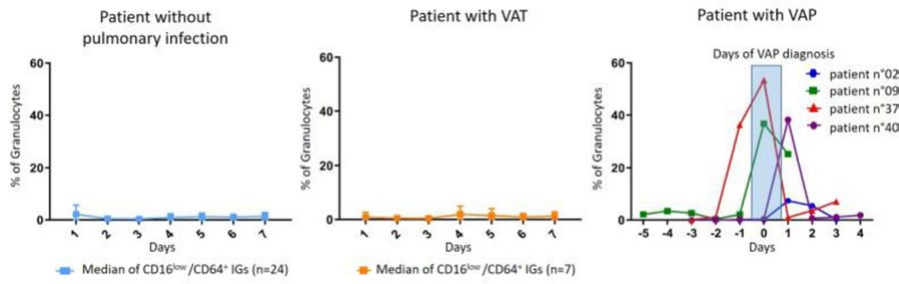
Median or individual daily follow-up of CD16low/CD64+ immature granulocytes (IGs) in peripheral blood during the first week of hospital stay under mechanical ventilation

## FC-239 Ventilator-associated pneumonia: the effect of using the closed suctioning system

### Rabeb Hammami^1^, Maryem Ben Amor^1^, Mahmoud Marzouk^1^, Sabeur Thamlaoui^1^, Nader Baffoun^1^, Kamel Baccar^1^, Chokri Kaddour^1^

#### ^1^Institut National De Neurologie, Tunis, Tunisie

##### **Correspondence:** Rabeb Hammami (hammamirabeb2@gmail.com)

*Annals of Intensive Care* 2023, **13(Suppl 1):**FC-0239

**Rationale:** Ventilator-associated pneumonia (VAP) is seen in 5 to 40% of ventilated patients with a huge variability between countries. A prevention strategy is mandatory to decrease them. Using the closed suctioning system could be one of them. The aim of this study was to study the impact of a closed endotracheal suction system on the development of VAP compared to the open system.

**Patients and methods/materials and methods:** We led a retrospective, monocentric, repeated cross-sectional, and comparative study for 2 years 2018 and 2020, in the department of anesthesia and intensive care at the institute of neurology. We included patients who required more than 48 h of mechanical ventilation. They were divided into two groups: the open system group named OS (2018) and the closed system group named CS (2020). The primary outcome was to compare the incidence of VAP between the two groups. Data were analyzed using SPSS version 25.0 and P < 0.05 was considered significant.

**Results:** We included 169 patients (CS group: 85; OS group: 84). Demographic characteristics, and severity scores were comparable between the two groups. For the CS group, 31 patients (36.4%) presented a VAP, versus 42 (50%) for the OS group (p = 0.076). The period between the onset of VAP and intubation was shorter in the OS group (9 days versus 15; p = 0.016). *Pseudomonas aeroginosa* was more frequent in the OS group (p = 0.047). There was no difference between the two groups in terms of drug resistance (p = 0.08), multidrug-resistant (p = 0.96), extensively drug-resistant (p = 0.12), and pan-drug-resistant (p = 0.86) bacteria. The length of hospitalization and duration of mechanical ventilation were higher in the CS group (p = 0.001). As for the complications, no difference was found. If a VAP occurred, the CS group had better survival (p = 0.006). No difference in cost-efficiency was found between the two groups.

**Conclusion:** A closed suctioning system does not significantly reduce the development of VAP. Its place in prevention strategies has still to be proven.

**Compliance with ethics regulations:** Yes in clinical research.

## FC-240 Ventilator-associated pneumonia (VASP) prospective study

### Abdelkader Azza^1^, Jamel Alachaher^1^, Hajer Boudadi^1^, Khalida Bouyacoub^1^, Sofiane Ouali Dada^1^, Nabil Tabet Aoul^1^, Houria Mokhtari Djebli^1^

#### ^1^Faculté de médecine, Oran, Algerie

##### **Correspondence:** Abdelkader Azza (azza.abdelkader@univ-oran1.dz)

*Annals of Intensive Care* 2023, **13(Suppl 1):**FC-0240

**Rationale:** Ventilatory associated pneumonia (VAP) is defined as nosocomial infectious pneumona developing within ≥ 48–72 h after intubation and mechanical ventilation. The aim of our work was to study the epidemiological, bacteriological and evolutionary profile of VAP.

**Patients and methods/materials and methods:** Prospective study of 2 years (2017 to 2018) in the multipurpose intensive care unit of the CHU Oran, including all patients who developed VAP.

**Results:** During the study period, 384 patients were hospitalized and ventilated in the department. Among them, 81 patients developed VAP (21.1%). The average age of our patients was 44.11 ± 20.49 years (range 2–86 years). A male predominance was observed (2/1). The mean time to onset of VAP was 5 ± 3.87 days (range 2–17 days). Regarding microbiological documentation, a single bacteria was isolated in 53 cases (81.5%), and two bacteria in 11 cases (16.9%). Gam negative bacteria were the most frequent: *Pseudomonas* (34) and *Acinetobacter baumanii* (34) followed by *E. coli* (6). In 3 cases (3.7%) the diagnosis of VAP was retained without bacteriological documentation. The average length of stay was 16.40 ± 12.39 days (range 2–78 days) and mortality was 61.7%.

**Conclusion:** The incidence of pneumonia acquired under mechanical ventilation in intensive care is very variable (from 16 to 70%) depending on the studies and the patients. Under our skies, the often-multi-resistant gram-negative bacilli are the most incriminated and mortality due to VAP remains high.

**Compliance with ethics regulations:** Yes in clinical research.

## FC-241 Betalactams for obese patients with sepsis or septic shock (BLOBSSS): a prospective multicenter study of exposition assessment

### Pauline Royet^1^, Perrine Alexandrzak^1^, Clara Lu^1^, Olivier Pouly^4^, Fabien Lambiotte^3^, Malcolm Lemyze^6^, Justine Lemtiri^3^, Christophe Vinsonneau^5^, Thomas Caulier^2^, Mélanie Verlay^1^, Benjamin Hennart^4^, Nicolas Van Grunderbeeck^1^

#### ^1^Centre Hospitalier de Lens, Lens, France; ^2^Centre Hospitalier de Tourcoing, Tourcoing, France; ^3^Centre Hospitalier de Valenciennes, Valenciennes, France; ^4^Centre Hospitalier Régional Universitaire de Lille, Lille, France; ^5^Centre Hospitalier de Béthune, Béthune, France; ^6^Centre Hospitalier d'Arras, Arras, France

##### **Correspondence:** Nicolas Van Grunderbeeck (nicovgdb9@orange.fr)

*Annals of Intensive Care* 2023, **13(Suppl 1):**FC-0241

**Rationale:** Obesity is pandemic and affects antibiotic pharmacokinetics (PK). Betalactams efficacy in sepsis could therefore be altered. We assessed the exposure of septic obese patients to four betalactams targeting Gram-negative bacteria (piperacillin-tazobactam TZP, cefepime, ceftazidime, meropenem).

**Patients and methods/materials and methods:** Prospective multicenter study, 6 centers. Patients with sepsis (2016 criteria) treated with cited betalactams, were sampled after 24 h of antibiotic treatment. Dosing was performed by liquid chromatography/tandem mass spectrometry. Antibiotic posology and infusion patterns were at the discretion of the clinician, and the PK targets (100% of time with dosing superior to 4 × Minimal Inhibitory Concentrations*,* and inferior to 8 × MIC for *Pseudomonas aeruginosa*) were defined following guidelines (1). Patient data included age, height, weight, body mass index (BMI), SOFA score, infection focus, renal clearance, and fluid balance. Antibiotics infusion data collected included 24-h posology, loading dose, infusion modes, and posology according to standard dose, total or adjusted weight. Patterns associated with PK targets were analyzed using Fisher and Mann–Whitney tests.

**Results:** Thirty-nine patients were included (TZP 15, cefepime 9, ceftazidime 4, meropenem 11). Median age was 61 [56–72] years, total weight was 112 [99–131] kg, and BMI was 37.8 [34.3–44.2] kg/m^2^. Infection foci were mainly pulmonary (43%), abdominal (23), and urinary (13%). Median SOFA on admission was 9 [5–11]. A loading dose of antibiotic was used in 27 patients (69%), and continuous infusion in 23 (59%). Intermittent infusion and standard posology were used in 12 and 19 patients, respectively. Results varied by molecules but some were significant for the whole population: - PK target of efficacy (100% of time > 4×MIC_*Pseudomonas*_) was obtained in 28 patients (72%), and 51% presented with dosing superior to 8×MIC, with toxicity risk. - Efficacy PK target was associated with loading dose (p = 0.06) and continuous infusion prescribing (p = 0.02). - No associations were found with posology or severity.

**Discussion:** Our study has limitations: limited recruitment, heterogeneous prescribing habits and infection foci. We couldn't assess the impact of fluid balance and renal clearance because of concerns regarding precision of data recovery. The study was not designed to assess the impact on outcome. Though, these data reflect current antibiotic use in this population, with room for improvement. They support guideline suggestions for loading doses and continuous infusion of betalactams at the early phase of sepsis, in morbidly obese septic patients. They also underline the variability of antibiotic exposure in sepsis, with therapeutic monitoring as a possible way to improve it.

**Conclusion:** Our results support guidelines suggesting the use of betalactams with a loading dose and continous infusion in obese patients with sepsis (1, 2).

**Reference 1**

Guilhaumou, R., Benaboud, S., Bennis, Y. et al. Optimization of the treatment with beta-lactam antibiotics in critically ill patients—guidelines from the French Society of Pharmacology and Therapeutics (Société Française de Pharmacologie et Thérapeutique

**Reference 2**

Evans, L., Rhodes, A., Alhazzani, W. et al. Surviving sepsis campaign: international guidelines for management of sepsis and septic shock 2021. Intensive Care Med 47, 1181–1247 (2021). https://doi.org/10.1007/s00134-021-06506-y

**Compliance with ethics regulations:** Yes in clinical research.

## FC-242 Cefotaxime dosing should be adapted to renal function

### Théo Dillies^1^, Guillaume Thiery^1^, Jérôme Morel^1^, Rémi Balluet^1^, Manon Launay^1^, Sophie Perinel-Ragey^1^

#### ^1^CHU de Saint Etienne, Saint Etienne, France

##### **Correspondence:** Manon Launay (manon.launay@chu-st-etienne.fr)

*Annals of Intensive Care* 2023, **13(Suppl 1):**FC-0242

**Rationale:** Intensive Care Unit patients present many pharmacokinetic and pharmacodynamic specificities leading to the risk of underdosing. Therapeutic monitoring of beta-lactam antibiotics has therefore been recommended in France since 2018. According to the French Summary of Product Characteristics, cefotaxime should be administered up to 12 g/day, depending on the severity of the infection, in all adults with creatinine clearance (CrCl) greater than 5 mL/min. But French nephrologists (“GPR”) recommend weight-normalized dose reduction of 25%, 50%, and 75% in mild, moderate, and severe impaired renal function, respectively. Moreover, obesity has been associated with lower exposure to other beta-lactams, such as ceftazidime. This study aims to investigate the impact of obesity and renal function on cefotaxime plasma concentrations.

**Patients and methods/materials and methods:** This retrospective cohort study was conducted between December 2020 and March 2022. The study was approved by the Institutional Review Board. All consecutive critically ill patients with cefotaxime monitoring at steady state (target range: 25–60 mg/L) were included in this study.

**Results:** A total of 70 patients (49 males), aged 59 ± 14 years, were included and 137 blood samples were collected. Cefotaxime underexposure and overexposure were observed in 34% and 12% of the patients, respectively. Mean cefotaxime concentration at first blood collection was not significantly different according to obesity (37.3 mg/L in obese versus 40.1 mg/L in non-obese patients, respectively p = 0.6452) for similar amount and CrCl. Cefotaxime concentrations were significantly different according to CrCl (ANOVA test on 5 stages, p < 0.0001), with 68 ± 32 mg/L for CrCl between 5 and 30 ml/min, to 27 ± 16 mg/L for CrCl above 130 ml/min.

**Conclusion:** Obesity had no significant impact on cefotaxime exposure, but CrCl was a significant covariate. As similar exposures were observed in obese and non-obese patients, a non-normalized dose reduction according to CrCl may be more appropriate. Further studies are necessary to confirm these preliminary findings.

**Compliance with ethics regulations:** Yes in clinical research.

**Figure 1 (abstract FC-242):**
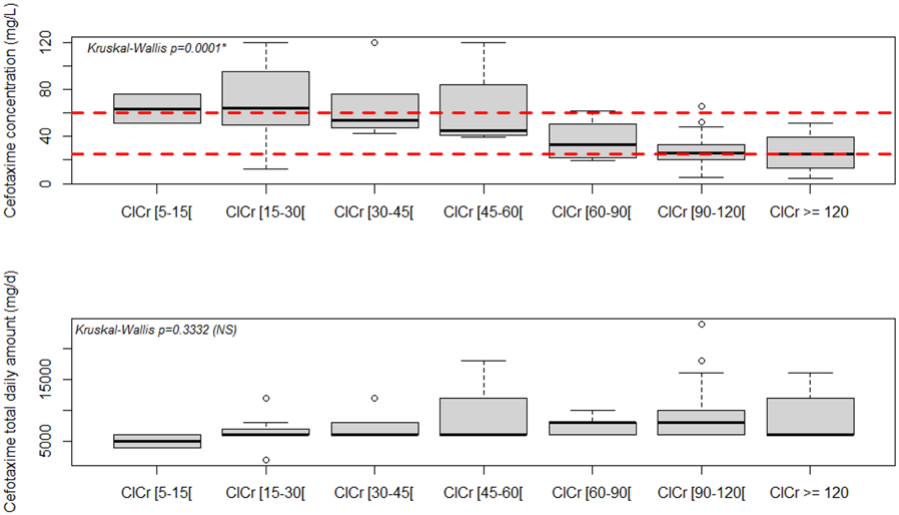
Box plot representing the difference of cefotaxime concentrations and dosing according to CKD-EPI classes. Target concentration was drawn using red dashed lines

## FC-243 Suboptimal concentrations of isavuconazole in critically ill patients treated for mold infections: a population pharmacokinetic analysis

### Charlotte Rabault^1^, Sébastien Bailly^2^, Etienne De Montmollin^1^, Paul-Henri Wicky^1^, Lila Bouadma^1^, Romain Sonneville^1^, Christine Bonnal^1^, Jean-François Timsit^1^, Minh Le^1^

#### ^1^Centre Hospitalo-Universitaire Bichat - Claude-Bernard, Paris, France; ^2^Université Grenoble Alpes, Grenoble, France

##### **Correspondence:** Charlotte Rabault (rabaultcharlotte@yahoo.fr)

*Annals of Intensive Care* 2023, **13(Suppl 1):**FC-0243

**Rationale:** Isavuconazole is approved as a first-line therapy for aspergillosis, including COVID-19 associated pulmonary aspergillosis (CAPA), and as an alternative treatment for mucormycosis. The objectives of this study were to determine the pharmacokinetic parameters of intravenous isavuconazole in critically ill patients using a population modeling approach and to identify covariates affecting PK in this specific setting.

**Patients and methods/materials and methods:** Critically ill patients treated for mold infections with rich pharmacokinetic blood sampling were retrospectively enrolled. The isavuconazole regimen consisted of an intravenous loading dose of 200 mg q8h for 2 days, followed by an intravenous maintenance dose of 200 mg q24h. Isavuconazole plasma concentrations were obtained as part of routine therapeutic drug monitoring using LC-MSMS (Waters Xevo). Population pharmacokinetic modeling was performed using Monolix 2021R2. AUC 0-24 h (Area Under the Curve), Cmin (concentration before the next infusion) and Cmax (concentration 1 h after infusion) were predicted by the model at the end of the loading dose (day 2).

**Results:** A total of 13 patients with 84 plasma isavuconazole concentrations were included. Our population was predominantely male with a median age of 55 years old and a median SOFA score of 8.5 and 12 at admission and on the day of dosing, respectively. 8 patients had a solid organ transplantation and 1 was diabetic. The diagnosis was aspergillosis in 11 cases (5 CAPA) and mucormycosis in 2 cases. No clinically relevant drug interaction was reported. The pharmacokinetic parameters of isavuconazole were best described by a two-compartment model. No significant covariate was identified at this stage. On day 2, the median predicted AUC 0–24 h was 30.48 mg h/L and predicted Cmin were 0.83 mg/L, with a coefficient of variability of 57.7% and 79.2%, respectively. At this stage, Cmin values tended to correlate with the AUC 0–24 h, both in observed and predicted data (Spearman correlation, R^2^ = 0.298, p = 0.088).

**Conclusion:** The median AUC 0–24 h of isavuconazole at the end of the loading dose in ICU patients is variable and less than one third of that previously described in the literature (Desai and al), with a risk of not achieving an adequate pharmacodynamic target in the first week of treatment. These results may highlight the need for further analysis of different loading dose regimens to achieve an adequate isavuconazole plasma exposure.

**Reference 1**

Desai AV, Kovanda LL, Hope WW, et al. Exposure–Response Relationships for Isavuconazole in Patients with Invasive Aspergillosis and Other Filamentous Fungi. Antimicrob Agents Chemother. 2017;61(12):e01034-17. https://doi.org/10.1128/AAC.01034-17

**Compliance with ethics regulations:** Yes in clinical research.

**Figure 1 (abstract FC-243):**
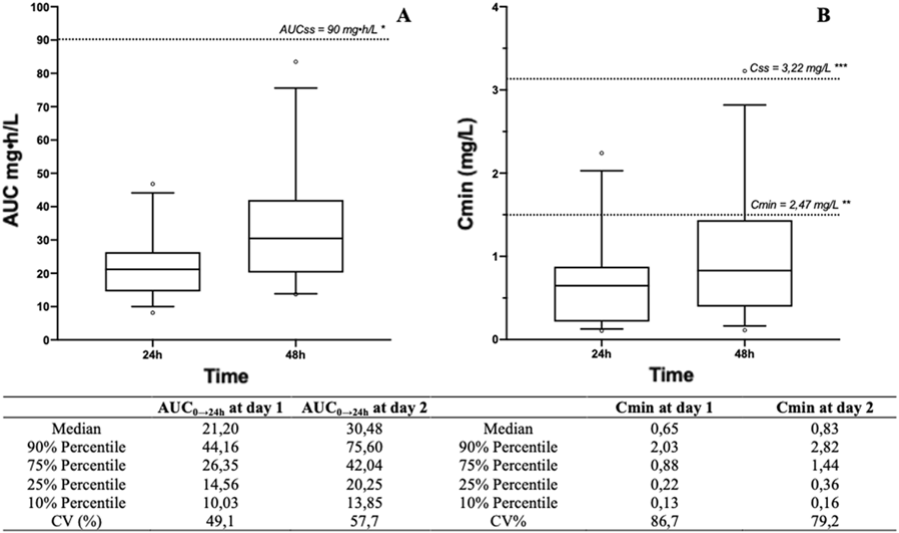
Predicted ISA serum concentration at day 1 and day 2 A. AUC_0–24 h_ at day 1 and day 2 B. C_min_ at day 1 and day 2 C. C_max_ at day 1 and day 2 AUC, Area Under Curve; C_min_, Serum concentration at 24 h post infusion; C_max_, serum concentration at 1 h post in

## FC-244 Equivalent concentration of isavuconazole administered via enteral feeding tube compared to IV or oral formulation in critically ill patients treated for invasive pulmonary aspergillosis

### Antoine Piantoni^1^, Benjamin Hennart^2^, Julien Poissy^1^, Emelin Cailliau^3^, Benjamin Valentin^4^, Marie Boulo^1^, Fanny Vuotto^5^

#### ^1^CHU Lille, Critical Care center, Lille, France; ^2^Toxicology Unit, Biology and Pathology Centre, CHU Lille, Lille, France; ^3^Biostatistics Department, CHU Lille, Lille, France; ^4^Institut de Pharmacie, CHU de Lille, Lille, France; ^5^Infectious Diseases Department, CHU Lille, Lille, France

##### **Correspondence:** Antoine Piantoni (antoine.piantoni@hotmail.fr)

*Annals of Intensive Care* 2023, **13(Suppl 1):**FC-0244

**Rationale:** Isavuconazole is a broad-spectrum triazole used as a first-line antifungal agent for invasive pulmonary aspergillosis (IPA) either orally or by intravenous (IV) infusion. Both forms are considered to be bioequivalent. During the SARS-CoV-2 pandemic, due to a lack of IV formulation, oral capsules of isavuconazonium were opened and administered via enteral feeding tube (EFT) to some patients hospitalized in our intensive care unit (ICU), based on a few studies in non-ICU patients suggesting bioequivalence. Considering the limited data available on this modality, a systematic drug concentration monitoring was performed. In this study, we compared concentrations obtained after either oral or IV administration in critically ill patients treated for IPA with isavuconazole.

**Patients and methods/materials and methods:** This is a retrospective monocentric study including patients hospitalized in the ICU between October 2017 and April 2022 and treated for IPA with isavuconazole administered either by conventional method (IV infusion or administration of isavuconazonium sulfate by oral capsules) or by EFT with therapeutic drug monitoring (TDM). Serum concentrations were compared between EFT and oral or IV administration in ICU patients on day 3 (after loading dose) and day 10 (at equilibrium). We also collected data on parameters potentially influencing the pharmacokinetic or pharmacodynamic properties of isavuconazole.

**Results:** 30 patients were included, 9 were treated via EFT, and 21 via conventional methods (13 via IV infusion and 8 via oral capsules). Most patients treated via EFT were included during SARS-CoV-2 pandemic and therefore treated for COVID-associated pulmonary aspergillosis (89% vs 29%). More of them were shocked (55 vs 31%). Other pharmacokinetic parameters were mostly similar (IMC 32 ± 4 vs 29 ± 6 kg/m^2^), acute kidney injury (33 vs 29%), acute liver failure (0 vs 14%). There was no difference between TDM of patients treated via EFT vs IV or PO in the ICU at equilibrium (respectively 1.8 ± 0.7 vs 1.9 ± 1.3, p = 0.86). All TDM at day 3 were in the therapeutics range, however there were too few data to perform a statistical comparison.

**Conclusion:** In this retrospective study, we demonstrated an equivalent concentration of isavuconazole administered via EFT compared to conventional methods in critically ill patients treated for IPA at equilibrium. Recommended therapeutic ranges were obtained with all routes of administration either after the loading dose (day 3) or at equilibrium.

**Compliance with ethics regulations:** Yes in clinical research.

## FC-245 Rezafungin treatment of candidemia and invasive candidiasis: outcomes stratified by baseline renal function—analysis of the phase 2 + phase 3 trials

### Hervé Dupont^1^, Taylor Sandison^2^, Jose A. Vazquez^3^, Patrick M. Honore^4^, Alex Soriano^5^, Karine Gouhier^7^, Monica Slavin^8^

#### ^1^CHU Amiens, Amiens, France; ^2^Cidara Therapeutics, San Diego, Etats-Unis; ^3^Augusta University, Augusta, Etats-Unis; ^4^Brugman Univ Hospital, Brussels, Brussels, Belgique; ^5^Hospital Clínic, CIBERINFEC, Univ. of Barcelona, Barcelona, Espagne; ^6^Hospital Del Mar-IMIM, CIBERINFEC, Barcelona, Espagne; ^7^Mundipharma, Paris, France; ^8^Peter MacCallum Cancer Centre and Royal Melbourne Hospital, Melbourne, Australie

##### **Correspondence:** Karine Gouhier (karine.gouhier@mundipharma.fr)

*Annals of Intensive Care* 2023, **13(Suppl 1):**FC-0245

**Rationale:** Rezafungin (RZF) once-weekly (QWk) is a next-generation echinocandin in development for the treatment of candidemia and invasive candidiasis (IC) and the prevention of invasive fungal disease caused by Candida, Aspergillus, and Pneumocystis spp. in BMT. RZF QWk was compared to caspofungin (CAS) QD in two double-blind, randomized, controlled trials for the treatment of candidemia and/or IC: STRIVE (Phase 2, NCT02734862) and ReSTORE (NCT03667690). Trial data (Phase 2 + Phase 3) were analyzed to evaluate outcomes stratified by renal function at baseline: CrCl ≥ 60 mL/min (normal/mild impairment [Norm/Mild]) and < 60 mL/min (moderate/severe impairment [Mod/Sev]).

**Patients and methods/materials and methods:** Outcomes were evaluated for differences between CrCl categories and between treatment groups: RZF QWk 400 mg on Wk 1 then 200 mg vs CAS QD 70 mg on Day (D)1 then 50 mg, for ≥ 14 days (≤ 4 Wks) w/optional oral fluconazole stepdown for CAS.

- **Results: D30 all-cause mortality (ACM)**
  Mod/Sev: RZF, 13% (7/54); CAS, 30.5% (18/59) Norm/Mild: RZF, 22.7% (17/75); CAS, 10.8% (9/83).
- **Mycological eradication (ME) at D5**
  Mod/Sev: RZF, 75.9% (41/54); CAS, 61.0% (36/59) Norm/Mild: RZF, 74.7% (56/75); CAS, 66.3% (55/83).
- **ME at D14**
  Mod/Sev: RZF, 75.9% (41/54); CAS, 57.6% (34/59) Norm/Mild: RZF, 69.3% (52/75); CAS, 74.7% (62/83).
- **≥ 1 treatment-emergent AE**
  Mod/Sev: RZF, 93.2% (55/59); CAS, 88.9% (56/63) Norm/Mild: RZF, 88.9% (72/81); CAS, 76.7% (69/90).

**Conclusion:** RZF efficacy was comparable across CrCl categories, with higher ME and lower D30 ACM in the Mod/Sev group. Further analyses are needed to evaluate the observed differences between treatment groups.

**Compliance with ethics regulations:** N/A.

## FC-246 Ventilator-associated pneumonia in sever burn patients

### Mohsna Bhiri^1,2^, Hana Fredj^1,2^, Rahma Messaoudi^1,2^, Maroua Jemii^1,2^, Bahija Gasri^1^, Imen Jami^1^, Amel Mokline^1,2^, Amen Allah Messaadi^1,2^

#### ^1^Service de réanimation des grands brûlés, Centre de traumatologie et des grands brûlés (CTGB), Tunis, Tunisie; ^2^Faculté de médecine de Tunis, Université El Manar, Tunis, Tunisie

##### **Correspondence:** Mohsna Bhiri (mohsna.bhiri@gmail.com)

*Annals of Intensive Care* 2023, **13(Suppl 1):**FC-0246

**Rationale:** Ventilator-associated pneumonia (VAP) is the most frequent hospital-acquired infection in intensive care units. This infection is associated with a high mortality rate. Critical burn patients are particularly susceptible to this nosocomial infection. We aim to study the epidemiologic, bacteriologic profile and evolutive pattern of VAP among severe burn, ICU hospitalized patients.

**Patients and methods/materials and methods:** We conducted a retrospective descriptive study, including all ICU severe burn patients presenting a VAP, from January 2020 to August 2022. The sociodemographic data of the patients were collected. The infectious event details (clinical and biological symptoms), responsible pathogens, conducted treatments as well as the outcomes were analyzed.

**Results:** During the study period, 1174 patients were admitted to the ICU, of which 224 patients underwent mechanical ventilation (MV) (with a duration more than 3 days) (21%). Fifty-five patients presented a VAP (22.5%). The mean age of the patients was 33 ± 19 years with a total burned surface area of 40 ± 19%. Intubation was an urgent procedure in 49 patients (89%), mostly (60%) for facial burns. The study event occurred within a delay of 6 ± 4 days [3–18]. Early-onset VAP was diagnosed in 42% of the population. The clinical findings leading to the VAP diagnosis were mainly: purulent tracheal secretions (34%), fever (65%) or hypothermia (30.9%), hypoxemia with a mean PF ratio of 157 ± 79 and progressive radiographic infiltrate (70%). Respiratory samples’ cultures were monomicrobial in 51% of the cases, and negative in 18%. The most frequent isolated pathogens leading to this infection were: *Acinetobacter baumanii* (n = 22), *Pseudomonas* (n = 9) and *Klebsiella pneumoniae* (n = 8). Ninety percent of the population was treated with an empiric antibiotic regimen. Early onset VAPs were mainly treated with: colistin (21%), piperacillin-tazobactam (14%) and teicoplanin (14%). However, the most commonly used antibiotics for late onset events were: colistin (40%), ertapenem (19.5%) and imipenem (11%). The mean duration of antibiotic therapy was 6.5 ± 2.6 days. The mean duration of mechanical ventilation was 12 ± 8 days, and the length of hospital stay was 17 ± 12.7 days. The global mortality rate was 74.5%.

**Conclusion:** Ventilator-associated pneumonia remains a common, yet preventable infectious complication (22.5%) among severely burnt patients. It is associated with a high mortality rate.

**Compliance with ethics regulations:** Yes in clinical research.

## FC-247 High dose of acid ascorbic improves hemodynamic state in septic shock burn patients?

### Rayane Nachi^1^, Amel Mokline^1^, Hana Fraj^1^, Sarra Ben Zarrouk^1^, Imen Jemi^1^, Bahija Gasri^1^, Manel Ben Saad^1^, Amen Allah Messadi^1^

#### ^1^Centre de traumatologie et des grands brulés, Tunis, Tunisie

##### **Correspondence:** Amel Mokline (dr.amelmokline@gmail.com)

*Annals of Intensive Care* 2023, **13(Suppl 1):**FC-0247

**Rationale:** Sepsis remains a serious and life-threatening condition with high morbidity and mortality. It is marked by an imbalance between increased production of free radicals and insufficient neutralization by endogenous antioxidants, including vitamin C. This causes a spread of oxidation reactions and contributes to organ failure (1). Although this imbalance has been proven to be more marked in burn patients, few studies were interested on the effects of vitamin C in this population. So, the aim of our study was to assess the hemodynamic impact of high-dose vitamin C (100 mg/kg/d) in septic burn patients.

**Patients and methods/materials and methods:** Case–control study conducted in an intensive burn care department in Tunisia for 26 months (July 2019–October 2021). Were included adult burn patients presenting with sepsis or septic shock (as defined in Sepsis-3). Were excluded pregnant woman, patients with a history of lithiasis or vesico-renal calculus, of hemochromatosis or G6PD deficiency, and those taking long-term vitamin C or glucocorticoid treatement. Only one septic episode was considered per patient during his hospitalization. After inclusion, ascorbic acid was prescribed at a dose of 100 mg/kg/day in 3 doses over 4 days. The vitamin C group was compared with a retrospective group (non-vitamin C) from the same center matchedfor age, sex and extent of burns (TBSA). Therapeutic management of sepsis was similar for the 2 groups in terms of fluid resuscitation with the same hemodynamic objectives (hourly output of 0.5 mL to 1 mL/kg and MAP > 65 mmHg). The primary endpoint was the impact of Vit C on the hemodynamic state as evaluated by fluid resuscitation, dose and duration of catecholamines.

**Results:** During the study period, 100 patients were included, divided into 2 groups: G1 (Vit C+; n = 50) and G2 (Vit C−: n = 50). Patients in the two groups were comparable in terms of sex, age and severity of burns (Table 1). Administration of vitamin C reduced the fluid balance on day 3 (2 ml/kg/day for G1 vs 13 ml/kg/day for G2; p = 0.008), reduced significantly doses of noradrenaline on day 3 (1.8 mg/h vs 3.5 mg/h; p = 0.01), and shortened the duration of noradrenaline dependance (4 days for G1 vs 4.84 days for G2; p = 0.28) in septic shock burn patients. No ascorbic acid-related adverse events were identified in the treatment group during the study.

**Conclusion:** High-dose vitamin C therapy was associated with reduced fluid balance, doses of noradrenaline and duration of dependance in septic shock burn patients within the first 3 days of sepsis.

**Reference 1**

Mikio Nakajima and al., Morita Kojiro, Shotaro Aso. Effect of high-dose vitamin C therapy on severe burn patients: a nationwide cohort study. Critical Care volume 23, Article number: 407 (2019).

**Compliance with ethics regulations:** Yes in clinical research.

**Table 1 (abstract FC-247):**
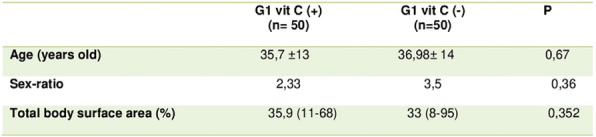
Characteristics of 2 groups

## FC-248 Plasma NET markers kinetics in COVID-19 patients receiving immunomodulatory treatments: a cohort study

### Geoffrey Garcia^1^, Antoine Dewitte^1^, Sylvie Labrouche-Colomer^1^, Christine Mouton^1^, Céline Delassasseigne^1^, Fanny Ménard^2^, Camille Vinclair^2^, Erwan Begot^2^, Laurent Petit^1^, Cédric Carrié^1^, Didier Brönnimann^1^, Denis Malvy^1^, Alexandre Duvignaud^1^, Chloé James^1^, Renaud Prével^1^

#### ^1^CHU Bordeaux, Bordeaux, France; ^2^CH Côte Basque, Bayonne, France

##### **Correspondence:** Renaud Prével (renaud.prevel@hotmail.fr)

*Annals of Intensive Care* 2023, **13(Suppl 1):**FC-0248

**Rationale:** In COVID-19, dexamethasone (DXM) and tocilizumab are used to alleviate inflammation in patients requiring oxygen support. Several evidence support a role of increased immunothrombosis and NETosis in particular, in the pathogenesis of disease severity. Whether the use of immunomodulatory drugs has any impact on NETosis is currently unknown. The potential beneficial effect of immunomodulatory drugs as early treatment in mild patients is being investigated in the COVERAGE trial (NCT04356495). The aim of this study is to characterize the kinetics of plasma NET markers in COVID-19 patients (early mild outpatients and critically ill ones) receiving immunomodulatory treatments.

**Patients and methods/materials and methods:** Early (≤ 7 days from the onset of symptoms) COVID-19 outpatients were randomly allocated to control group or experimental arms (inhaled ciclesonide or inhaled IFN-β-1b). Blood samples were collected at inclusion and Day 7. COVID-19 worsening was assessed at Day 7. Critically ill COVID-19 patients received or not immunomodulatory treatments (dexamethasone ± tocilizumab) depending on the date of admission. Blood samples were collected at admission to ICU, Day 3 and Day 7. Plasma NET markers were assessed by quantification of MPO-DNA complexes and citrullinated histone H3.

**Results:** One hundred and sixty-six outpatients were included: 79 (48%) received placebo, 48 (29%) inhaled ciclesonide and 39 (23%) received inhaled IFN-β-1b. Ten (6%) required further hospitalization for respiratory support. Inhaled IFN-β-1b, but not inhaled ciclesonide nor placebo, was linked to a decrease in plasma H3Cit levels (p = 0.02). Fifty-nine critically ill patients were included: 8 received placebo (14%), 42 (71%) dexamethasone and nine (15%) dexamethasone + tocilizumab. Five (9%) patients deceased, for in the dexamethasone group and 1 in the dexamethasone + tocilizumab group. Dexamethasone alone and in association with tocilizumab was not linked to a decrease in plasma NET markers levels.

**Conclusion:** Use of immunomodulatory therapies is not linked to a decrease in plasma NET markers in COVID-19 patients, except for inhaled IFN-β-1b in early mild outpatients. Adjunction of treatment targeting NETosis might be an interesting therapeutic approach in COVID-19 patients.

**Compliance with ethics regulations:** Yes in clinical research.

## FC-249 Continuous renal replacement therapy with oxiris filter in critically ill covid-19 patients

### Rania Bounab^1^, Nicholas Heming^1^, Virginie Maxime^1^, Emmanuelle Kuperminc^1^, Miguel Carlos^1^, Pierre Moine^1^, Sylvie Chevret^2^, Djillali Annane^1^

#### ^1^APHP Raymond Poincaré, Garches, France; ^2^Saint-Louis hospital and Clinical Epidemiology, Paris, Paris, France

##### **Correspondence:** Rania Bounab (rania.bounab@aphp.fr)

*Annals of Intensive Care* 2023, **13(Suppl 1):**FC-0249

**Rationale:** COVID-19 causes a major inflammatory response, which may progress to shock and multiple organ failure. We explored whether continuous renal replacement therapy (CRRT) using adsorption membrane (oXiris®) could reduce the inflammatory response in critically ill COVID-19 patients with acute renal failure (ARF) [1–2].

**Patients and methods/materials and methods:** Case–control study including 24 critically ill COVID-2019 patients requiring RRT using an oXiris filter. We measured cytokines before and during treatment as well as relevant clinical endpoints. The control group was selected among COVID-19 patients included into our ongoing RECORDS trial (NCT04280497) who received RRT without adsorbing filters.

**Results:** 24 severe COVID-19 patients, admitted to the intensive care unit (ICU) and treated with CRRT using the Oxiris filter between March and April 2020 (20 males and 4 females); median age 67. The average time from COVID-19 symptoms to initiation of oXiris treatment was 18 ± 7 days, and from ICU admission to initiation of oXiris treatment 9.5 ± 7.8 days and from ARF to oXiris treatment was 3 ± 5 days. The average length of treatment was 152.8 ± 92.3 h. Treatment was associated with cytokine decreases for IL-1beta (p = 0.00022), MCP-1 (p = 0.03), and MIP-1 alpha (p = 0.03). The SOFA scores also showed a reduction over 48 h of therapy without reaching statistical significance. Our study found no significant effect of hemodynamic status. The average ICU stay length was 14 ± 5 days and the mortality rate was 79% in the Oxiris group. We compared the mortality across the two matched groups, there was no evidence of any difference in mortality (Figure 1).

**Conclusion:** In our study, CRRT using the oXiris filter seemed to effectively remove IL-1 beta, MCP-1, and MIP-1 alpha in COVID-19 patients. These exploratory results should be confirmed in a randomized controlled study.

**Reference 1**

Broman, M.E., et al., PLoS One, 2019. 14(8): p. e0220444.

**Reference 2**

Stockmann, H., et al., Crit Care Med, 2022. 50(6): p. 964–976.

**Compliance with ethics regulations:** Yes in clinical research.

## FC-250 Clinical and biological risk factor of thromboembolic in the COVID-19 disease in intensive care unit

### Benjamin Swinyard^1^, Alexis Lambour^1^, Yoann Zebib^1^, Simon Soudet^1^, Valery Salle^1^, Virginie Hoguet^1^, Momar Diouf^1^, Julien Maizel^1^, Nicolas Guillaume^1^, Michel Slama^1^

#### ^1^CHU Amiens-Picardie, Amiens, France

##### **Correspondence:** Benjamin Swinyard (benjamin.swinyard@hotmail.fr)

*Annals of Intensive Care* 2023, **13(Suppl 1):**FC-0250

**Rationale:** Sars-cov2 pulmonary disease did a lot of victims and intensive care unit hospitalization since February 2020. Deep vein thrombosis and pulmonary embolism are two of the most currents complications. This study tries to Figure out clinical and biological risk factor of thrombosis in intensive care unit.

**Patients and methods/materials and methods:** Patients hospitalized between February 27, 2020, and October 3, 2021, were included. Biological test occurred every week since inclusion and for 4 weeks. A Doppler echography was performed every week.

**Results:** 123 patients were included, 34 (27.6%) had a thrombosis event. Patients with thrombosis event stayed longer in intensive care unit (median 21 vs 11.5 days, p = 0.03), had more extra-corporal oxygenation (20.6 vs 6.4%, p = 0.03). No medical history was significantly different. In the initial biology test, patient with a thrombosis had less hemoglobin (median = 9.85 vs 11.5, p = 0.015), longer aPPT ratio (median = 1.29 vs 1.04, p = 0.036), higher IL-6 level (median = 34.75 vs 22.75, p = 0.045). Won Willebrand factor were non significantly higher (median = 415 vs 387, p = 0.2) but were significantly higher with the deceased patients, especially at D14 (p = 0.035). In the multivariate analysis, only hemoglobin is significantly lower in the thrombosis group. Non-other biological test was significantly different in the two groups in the follow-up biology tests.

**Conclusion:** This study confirms the link between thrombosis and inflammation in COVID-19 disease and shows interest in anti-inflammatory treatment. It needs other studies to understand the meaning of the lesser hemoglobin level in patient with thromboembolic event.

**Compliance with ethics regulations:** Yes in clinical research.

## FC-251 Omicron variant induced distinct immune respiratory transcriptomics signatures compared to pre-existing variants in COVID-19 patients requiring ICU admission

### Pierre Bay^1^, Christophe Rodriguez^1^, Stefano Caruso^1^, Vanessa Demontant^1^, Laure Boizeau^1^, Alexandre Soulier^1^, Paul-Louis Woerther^1^, Armand Dessap^1^, Jean-Michel Pawlotsky^1^, Nicolas De Prost^1^, Slim Fourati^1^

#### ^1^CHU Henri Mondor, Créteil, France

##### **Correspondence:** Pierre Bay (pierre.bay@aphp.fr)

*Annals of Intensive Care* 2023, **13(Suppl 1):**FC-0251

**Rationale:** Severe COVID-19 is generally related to dysregulated immune responses. We aimed to explore the differential effect of several SARS-CoV-2 variants of concern (VOC) on the immune and inflammatory responses in patients requiring admission to the intensive care unit (ICU).

**Patients and methods/materials and methods:** This prospective monocentric study investigated the nasopharyngeal transcriptomic characteristics of patients with severe COVID-19 admitted to the ICU during several COVID-19 waves when several VOC circulated in France (ancestral, Alpha, Delta and Omicron variants), between March 2020 and March 2022. Patients were classified according to VOC, based on matching on prespecified clinical criteria. Profiling of gene expression markers of innate and adaptive immune responses were investigated by respiratory transcriptomics at ICU admission. Gene expression markers were explored by both unsupervised and supervised analyses. Immune cell lineages abundance was determined by use of MCP-counter. Gene-set enrichment analysis was performed by use of Metascape.

**Results:** Overall, among 277 patients admitted in the ICU for severe COVID-19, 88 were included in the study after matching (ancestral (n = 24), Alpha (n = 24), Delta (n = 22) and Omicron (n = 18) variants). Fifty-three patients (60.2%) required invasive mechanical ventilation support during ICU stay. Transcriptomic analysis, performed on nasopharyngeal swab obtained within 72 h of ICU admission, revealed distinct innate and adaptive immune profiling between variants. There was a reduced expression of neutrophil degranulation, T cell activation, cytokines signalling pathways in patients infected with Alpha and Delta variants compared to patients infected with the ancestral variant (Figure 1). In contrast, there was a significantly higher expression of interferon-alpha, neutrophil degranulation, T and B cells activation, and inflammatory interleukins signalling pathways in patients infected with Omicron variant compared to previously circulating variants.

**Conclusion:** Omicron induced distinct immune respiratory transcriptomics signatures compared to pre-existing variants in patients with severe COVID-19 pneumonia, explained in part by reduced innate immune suppression, pointing to an evolving pathophysiology of severe COVID-19 pneumonia in the Omicron era. Further studies are needed to investigate the specific pathways involved in these responses, to better define immunotherapy strategies in severe COVID-19 patients in the context of continuous emergence of Omicron sublineages.

**Compliance with ethics regulations:** Yes in clinical research.

**Figure 1 (abstract FC-251):**
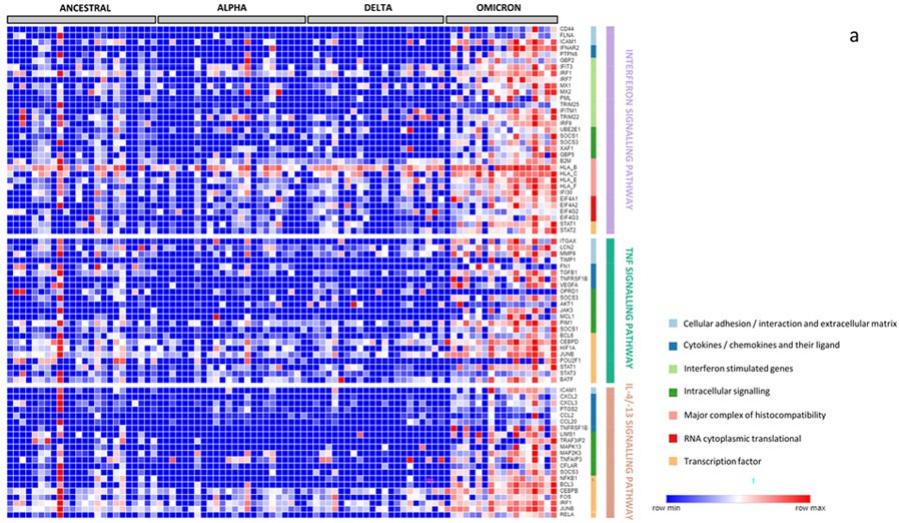
Heatmap analysis the transcripts with significantly different expression (i.e., normalized count expression in patients infected with Alpha, Delta or Omicron in comparison with ancestral variant, using Kruskal–Wallis test adjusted

## FC-252 Lung abscesses complicating ventilator-associated pneumonia in patients admitted to the ICU for COVID-19: characteristics and prognosis in a retrospective multicenter series

### Kais Ladjal^1^, Sami Hraiech^1^

#### ^1^Hôpital Nord, Réanimation médicale, Marseille, France

##### **Correspondence:** Kais Ladjal (kais.ladjal@hotmail.fr)

*Annals of Intensive Care* 2023, **13(Suppl 1):**FC-0252

**Rationale:** Patients undergoing mechanical ventilation (MV) for SARS-COV-2 pneumonia have an increased risk of bacterial ventilator-associated pneumonia (VAP). The occurrence of lung abscesses following VAP in these patients has been little studied. We aimed to describe here the incidence, microbiology, patients’ characteristics and prognosis and to identify risk factors for lung abscesses complicating VAP after SARS-CoV-2 pneumonia.

**Patients and methods /materials and methods:** We conducted an observational, retrospective study in French three intensive care units. Patients admitted for acute respiratory failure secondary to SARS-CoV-2 pneumonia and requiring MV for more than 48 h were included. Patients were compared according to the occurrence of 1 VAP or more and the development lung abscess.

**Results:** Three hundred and twenty-six patients (64%) had a confirmed diagnosis of bacterial VAP among the 507 requiring MV for more than 48 h. Of these, 7% (23/326) developed a lung abscess during their ICU stay. In terms of microbiological identification of these abscesses, enterobacteria were the majority bacteria, followed by non-fermenting gram-negative bacilli and then gram-positive cocci. In univariate analysis, the occurrence of lung abscesses was associated with the presence of a bacterial co-infection at ICU admission, probabilistic antibiotic therapy and the use of immunosuppressive treatments during the ICU stay. In multivariate analysis, the occurrence of lung abscess was associated with polymicrobial first VAP documentation [Odds Ratio (OR) 95% CI 3.79 (1.2–12)], treatment with hydrocortisone [OR 6.2 (1.9–19.5)] and tocilizumab [OR 5 (1.4–18.1)]. Lung abscess was associated with a longer duration of MV (10 days in patients without VAP, 25 days in patients with VAP but no abscess, and 49 days in patients with VAP and lung abscess, p < 0.001), prolonged ICU stay (14 days in patients without VAP, 28 days in patients with VAP but no abscess, and 52 days in patients with VAP and lung abscess, p < 0.001) and a higher ICU mortality (20% in patients without VAP, 35% in patients with VAP but no abscess, and 52% in patients with VAP and lung abscess, p = 0.001).

**Conclusion:** In our cohort, lung abscess affected 7% of patients with VAP following SARS-CoV-2 pneumonia. It was associated with significant mortality and morbidity. The use of immunosuppressive or immunomodulatory therapies such as hydrocortisone and tocilizumab as well as a polymicrobial first VAP documentation seemed to be the main risk factors.

**Compliance with ethics regulations:** Yes in clinical research.

## FC-253 SARS-CoV-2-related and attributable deaths in children: results from the French pediatric national registry

### Marguerite Lockhart-Bouron^1^, Noémie Vanel^6^, Etienne Javouhey^5^, Marion Caseris^4^, Julia Dina^2^, Sophie Breinig^3^, Michael Levy^4^, Stéphane Leteurtre^1^, Morgan Recher^1^, David Brossier^2^

#### ^1^CHU de Lille - Hôpital Jeanne de Flandre, Lille, France; ^2^CHU de Caen, Caen, France; ^3^CHU de Toulouse - Hôpital Mère et enfants, Toulouse, France; ^4^Hôpital Robert Debré - APHP, Paris, France; ^5^Hôpital Femme Mère Enfant - HPL, Lyon, France; ^6^Hôpital La Timone Enfants - APHM, Marseille, France

##### **Correspondence:** Marguerite Lockhart-Bouron (marguerite.bouron@chu-lille.fr)

*Annals of Intensive Care* 2023, **13(Suppl 1):**FC-0253

**Rationale:** SARS-COV-2 is responsible for an important mortality rate worldwide. Deaths related to SARS-COV-2 represent 6.6 million people, mainly in adult population. In pediatrics, mortality data related to SARS-COV-2 vary between countries, with an overall mortality of 0.08%. Mortality occurred in children with comorbidities. There are few descriptions of pediatric clinical presentations related to SARS-COV-2 that have resulted in death. Causality of SARS-COV-2 remains to be demonstrated. The main objective of this study was to evaluate the mortality rate of SARS-CoV-2 infected patients in the PICU. The secondary was the evaluation of SARS-CoV-2 imputability on death.

**Patients and methods/materials and methods:** A multicentric prospective cohort study with post-hoc analysis was conducted. All deceased pediatric patients infected with SARS-COV-2, hospitalized in pediatric intensive care unit between September 1, 2021, and August 31, 2022, and registered in the French national registry PICURE were included. PICURE is a public health database including 33 pediatric intensive care and emergency units in France, Belgium and Canada. The data were demographic, clinical, microbiological, radiological, ventilatory, therapeutic, complications, specific per-death management, and treatment limitations. Included patients were described and compared to a control group of SARS-COV-2 living patients whose data were registered in the PICURE database during the study period. All included cases were evaluated by 4 experts in the field of both pediatric infectiology and pediatric intensive care (1 Infectivologist, 2 Pediatric Intensivist, and 1 Biologist-Virologist). Experts were asked to classify the SARS-CoV-2 imputability on death in four categories: formal, probable, possible, or not retained. Their decision was based on temporality, semiology, conditions of death, terrain, differential diagnosis and autopsy.

**Results:** 891 children were included in PICURE database during the study period with 39 deaths (4.3%). Ages ranged from 10 days to 15 years. Only 1 death (2.6%) of the 39 was formally attributable to SARS-COV-2, 10 deaths were probably attributable (25.6%), 11 deaths were possibly attributable (28.2%), and 17 deaths were not attributable to SARS-COV-2 (43.6%).

**Conclusion:** Pediatric deaths directly related to SARS-COV-2 are rare and often associated with comorbidities.

**Compliance with ethics regulations:** Yes in clinical research.

## FC-254 Risk of admission to the pediatric intensive care unit for SARS-CoV-2 delta and omicron infections

### Morgan Recher^1^, Stéphane Leteurtre^1^, Etienne Javouhey^2^, Luc Morin^3^, Florent Baudin^2^, Jérôme Rambaud^6^, Guillaume Mortamet^5^, Hervé Hubert^1^, François Angoulvant^7^, Michael Levy^4^

#### ^1^Centre hospitalier universitaire de Lille, Lille, France; ^2^Centre hospitalier universitaire de Lyon, Lyon, France; ^3^Centre hospitalier universitaire du Kremlin-Bicêtre, Paris, France; ^4^Centre hospitalier universitaire de Robert-Debré, Paris, France; ^5^Centre hospitalier universitaire de Grenoble, Grenoble, France; ^6^Centre hospitalier universitaire de Trousseau, Paris, France; ^7^Centre hospitalier universitaire de Lausanne, Lausanne, Suisse

##### **Correspondence:** Morgan Recher (morgan.recher@chu-lille.fr)

*Annals of Intensive Care* 2023, **13(Suppl 1):**FC-0254

**Rationale:** The severity of SARS-CoV-2-related diseases in children remains unclear. This study aimed to describe the incidence of French pediatric intensive care units (PICUs) admissions with acute COVID-19, incidental positive SARS-CoV-2 test result, and multisystem inflammatory syndrome in children (MIS-C) during the delta and omicron variant periods.

**Patients and methods/materials and methods:** This study used the French PICU registry to obtain data on all patients admitted to 41 French PICUs diagnosed with acute COVID-19, incidental positive SARS-CoV-2 test result, or MIS-C between August 30, 2021, and April 20, 2022. Data regarding the total number of positive SARS-CoV-2 polymerase chain reaction results according to the type of variants were obtained from the French National Public Health Agency.

**Results:** Of 745 children, 244 (32·8%) were admitted for acute COVID-19, 246 (33·0%) for incidental positive SARS-CoV-2 test results, and 255 (34·2%) for MIS-C. The incidence of each group was higher with delta than with omicron. The incidence rate ratios with the delta variant were 7·47 (95% CI 4·22–13·26) for acute COVID-19, 4·78 (95% CI 2·30–9·94) for incidental positive SARS-CoV-2 test results, and 10·46 (95% CI 5·98–18·31) for MIS-C compared to the omicron variant (Figure). The median age was 66 (7.7–126.8) months; 314 (42%) patients had comorbidities. Patients with acute COVID-19 and incidental positive SARS-CoV-2 test results had similar proportions of comorbidities. No patient with MIS-C died, whereas the mortality rates in the acute COVID-19 and incidental positive SARS-CoV-2 test results groups were 6·8% and 3·8%, respectively.

**Conclusion:** The incidence of acute COVID-19, incidental positive SARS-CoV-2 test results, and MIS-C admitted to the PICU were significantly higher with the delta variant than with the omicron variant.

**Compliance with ethics regulations:** Yes in clinical research.

**Figure 1 (abstract FC-254):**
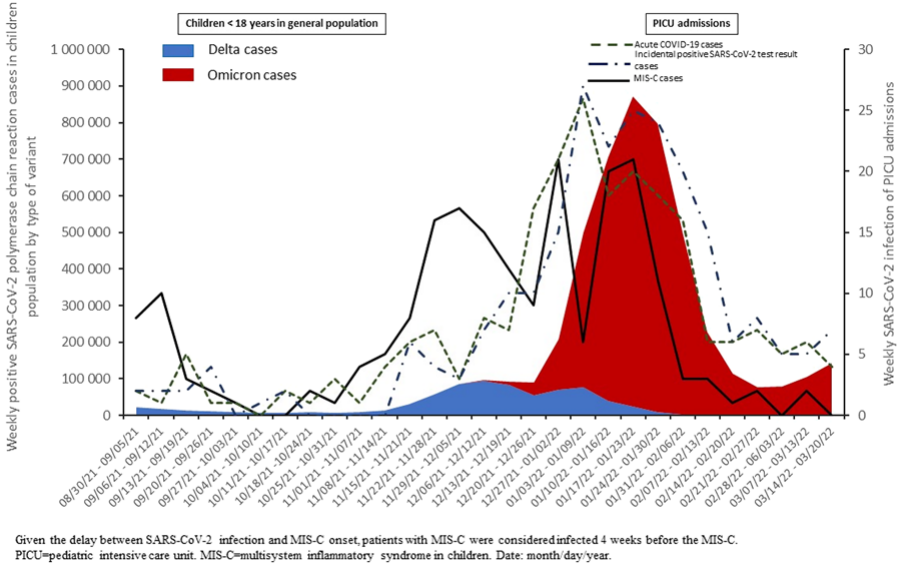
Weekly evolution of positive SARS-CoV-2 polymerase chain reaction in general population and PICU admission with acute COVID-19, incidental positive SARS-CoV-2 test results, and MIS-C

## FC-255 Toll-like receptor 2 (TLR2) modulates the bronchial epithelial response following remote polymicrobial sepsis

### Alice Friol^2^, Jolan Malherbe^2^, Christophe Rousseau^2^, Charlène Dauriat^2^, Edwige Peju^2,3^, Jean-François Llitjos^2^, Léa Boulant^2^, Clémence Martin^2,4^, Benoit Chassaing^2^, Pierre-Régis Burgel^2,4^, Maha-Zohra Ladjemi^2^, Frédéric Pène^2,3^

#### ^1^Hôpital Lariboisière, Paris, France; ^2^Institut Cochin, Equipe “Réponses immunitaires pulmonaires et systémiques au cours des infections aiguës et chroniques”, Paris, France; ^3^Service de médecine intensive réanimation de l'Hôpital Cochin, Paris, France; ^4^Service de pneumologie de l'Hôpital Cochin, Paris, France

##### **Correspondence:** Alice Friol (alice.friol@aphp.fr)

*Annals of Intensive Care* 2023, **13(Suppl 1):**FC-0255

**Rationale:** Abdominal polymicrobial sepsis induces dramatic susceptibility to secondary bacterial pneumonia (i.e.) dependent on TLR2 signalling as suggested by the particular resistance of *Tlr2*^*−/−*^ post-septic mice to secondary *Pseudomonas aeruginosa* pneumonia. Sepsis-impaired lung immunity has been ascribed to several defects in hematopoietic immune cells, but the role of airway epithelium is poorly understood despite its key-role of first-line lung defense through various functions of physical barrier, tissue repair, pathogen sensing and crosstalk with immune cells. In addition, microbiota is emerging as an essential factor of regulation of mucosal immunity. We hypothesized that polymicrobial sepsis may modulate the pulmonary dysbiosis and the main functions of airway epithelial cells. To this aim, we addressed the pulmonary dysbiosis and the morphological and functional alterations of airway epithelium following polymicrobial peritonitis in WT and *Tlr2*^*−/−*^ mice.

**Patients and methods/materials and methods:** Wild Type (WT) and Tlr2-deficient (*Tlr2*^*−/−*^) C57BL/6J mice were used for the experiments. Mice were subjected to polymicrobial peritonitis induced by caecal ligation and puncture (CLP), or control sham surgery. Morphology and functions of airway epithelial cells were studied by immunohistochemistry and flow cytometry on day 7 after surgery, prior to any secondary bacterial challenge. Gut (faeces) and lung microbiota diversities were studied by 16s ribonucleic acid (RNA) sequencing.

**Results:** Polymicrobial peritonitis induced pulmonary dysbiosis in WT mice, characterized by an increase in proportion of *Enterobacteriaceae*. In addition, polymicrobial sepsis resulted in morphological and functional changes in WT mice, characterized by increased epithelial thickness (Figure) and alteration in tight junctions with decreased expression of *Zonula occludens 1* (ZO-1). In addition, polymicrobial sepsis promoted an immunosuppressive pattern of bronchial epithelial cells through increased expression of the checkpoint molecules programmed cell death ligand 1 (PD-L1) and Herpes Virus Entry Mediator (HVEM), and increased expression of TLR5. Such sepsis-induced changes in epithelial cells were prevented in *Tlr2*^*−/−*^ mice subjected to polymicrobial sepsis.

**Conclusion:** TLR2 is involved in the sepsis-induced alterations in airway epithelial cells, possibly in relation with pulmonary dysbiosis. How airway epithelium is regulated following remote sepsis and whether it actually contributes to lung defense in this setting deserves further investigations.

**Compliance with ethics regulations:** Yes in clinical research.

**Figure 1 (abstract FC-255):**
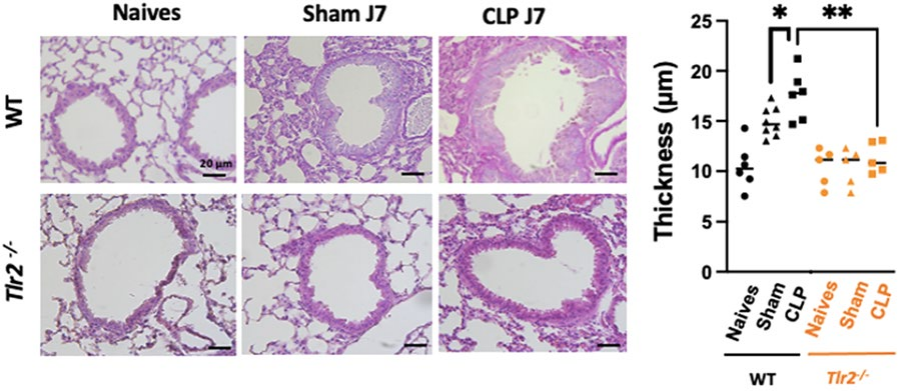
Bronchial epithelial thickness. (A)Representative images of haematoxylin–eosin stained lung sections from naïve WT(n = 6), sham(n = 8), CLP(n = 6) mice, and naïve Tlr2-/-(n = 5), sham(n = 5), CLP(n = 5) mice. (B)Thickness was mesured and analysed *p < 0.05 **p < 0.01

## FC-256 Platelet activation in leptospirosis and other bacterial sepsis: a preliminary study

### Frédéric Martino^1^, Livia Claude^1^, Laurent Camous^1^, Jean- David Pommier^1^, Marc Valette^1^, Véronique Baccini^1^

#### ^1^Service de Médecine Intensive Réanimation, CHU de la Guadeloupe, Les Abymes, France

##### **Correspondence:** Frédéric Martino (frederic.martino@chu-guadeloupe.fr)

*Annals of Intensive Care* 2023, **13(Suppl 1):**FC-0256

**Rationale:** Sepsis is one of the main causes of admission in Intensive Care Unit (ICU). Leptospirosis is among the most common anthropozoonoses in the world and specially in our tropical area. This disease, associated with thrombopenia, acute kidney injury and jaundice often leads to multiple organ failure and is associated with ICU hospitalization. Platelets are known to be involved in immune response to bacterial or viral stimuli. To date all mechanisms involved are not resolved. We aim to better understand platelets activation and pathways involved in this disease.

**Patients and methods/materials and methods:** We included consecutive leptospirosis admitted in ICU from October 2021. We also included patients with other bacterial sepsis and healthy donors not hospitalized as controls. We collected demographic data (age, gender), Severe Acute Physiological Score II (SAPS II), Sequential Organ Failure Assessment (SOFA) score and regular blood test. Platelets activation was assessed by flow cytometry using labelled anti-body: anti-CD41-ECD, anti-CD62P-APC, a fluorescent green probe labeled inhibitor of caspase-1 (FLICA) that irreversibly binds to activated caspase-1. Except Body Mass Index (BMI), data are presented as median and interquartile range 25–75.

**Results:** Thirty-two patients were included, among them 9 leptospirosis (Lepto), 11 bacterial sepsis (Bact) and 12 healthy controls (HC). Causes of bacterial diseases were: 4 pneumonia, 2 bacteremia, 2 urinary tract infections, 2 peritonitis, 1 Fournier's gangrene. Fifty six percent of them were male patients, median-aged of 58 (40–65) years old. The mean BMI was 26.6 ± 4.2 (kg/m^2^), with no difference between Lepto and Bact. The median SOFA score at ICU admission was 13 (8–16) in Lepto vs 7 (6–10) in Bact, p = 0.067. Median SAPS II was 49 (42–57). Median caspase-1 in HC was 4.65% (2.35–7.03); compared to Lepto 9.53% (5.78–20.87) p = 0.069 and Bact 9.54% (5.63–49.19) p = 0.06, no significant difference was identified. Membrane expression of P-selectin was 13.99% (8.17–20.3) in HC and was significantly higher in Bact 18.93% (14.90–38.59) vs Lepto 8.68% (5.26–12.53) p = 0.0084. Platelets were lower in Lepto 72 (42.5–105.5) vs HC 283 (254–323) G/L p = 0.0005. Bilirubin was higher in Lepto 203 (105–359) vs Bact 18 (6–53) µmol/L p = 0.0019. C-reactive protein was lower in Lepto 137 (74.9–239) vs Bact 280.4 (186–344) mg/L p = 0.0165.

**Conclusion:** Platelet activation revealed by P-selectin expression is higher in Bact than Lepto whereas thrombopenia is more severe in Lepto than Bact which could suggest platelets might be more activated and then destroyed in Lepto. All pathways are not highlighted but further works are in progress including increase of patients.

**Compliance with ethics regulations:** Yes in clinical research.

## FC-257 B-glucan enhances LPS-induced acute lung injury via alveolar macrophages reprogramming

### Renaud Prével^1^, Erwan Pernet^1^, Abderrahmane Sadek^2^, Shradha Wali^1^, Kim Tran^1^, Mina Sadeghi^1^, Renata Sindeaux^1^, Leonardo Jurado^1^, Jérémie Poschmann^2^, Maziar Divangahi^1^

#### ^1^Meakins-Christie Laboratories, McGill University, Montreal, Canada; ^2^Centre de Recherche en Transplantation et Immunologie, UMR1064, ITUN, Nantes, France

##### **Correspondence:** Renaud Prével (renaud.prevel@hotmail.fr)

*Annals of Intensive Care* 2023, **13(Suppl 1):**FC-0257

**Rationale:** Mortality due to acute respiratory distress syndrome is still as high as 40% leading to about 400,000 deaths per year worldwide. Despite more than four decades of research, no major improvements have been made except the development of “protective ventilation”. In particular, no immunomodulatory treatment has clearly been proven to be effective. Trained immunity (TI) is the ability for the innate immune system to be reprogrammed by a first insult, without persistent inflammation, then providing an increased response to a second insult. The aim of this study is to confirm that TI could provide protection against acute lung injury (ALI).

**Patients and methods/materials and methods:** Twelve weeks old male C57BL/6J mice were intra-nasally instilled with 50 μg and 100 μg of LPS and with 50 μg of poly(I:C), 7 or 30 days after intra-peritoneal injection of a training agent (1 mg of β-1,3-(d)-glucan). Alveolar macrophages (AM) depletion was obtained by intra-nasal clodronate instillation. AM adoptive transfer was performed in Day 2 Csf2rb−/− pups. ALI was assessed by lung imaging, histology, alveolar-capillary permeability, pro-inflammatory cytokines production and inflammatory cells recruitment. AM reprogramming was assessed ex vivo by pro-inflammatory cytokines production, metabolism exploration (Seahorse) and transcriptomics.

**Results:** Lung injury is significantly increased in β-glucan trained mice compared to controls in both LPS and poly(I:C) models with long-term persistence. Depletion of AM alleviates this increase in ALI which was restored after adoptive transfer of trained AM, demonstrating the direct role of AM in increased inflammatory responses. Ex vivo experiments confirmed AM reprogramming with increased pro-inflammatory cytokines production after LPS stimulation with switch to glycolytic metabolism.

**Conclusion:** β-Glucan reprograms alveolar macrophages promoting acute lung injury after LPS challenge.

**Compliance with ethics regulations:** Yes in animal testing.

## FC-258 Skin biopsy in adult patients with meningococcal purpura fulminans: a French multicenter retrospective cohort study

### Damien Contou^1^, Gaëtan Béduneau^5^, Charlotte Rabault^3^, Antoine Marchalot^4^, Martin Cour^6^, Sébastien Préau^7^, Rémi Coudroy^8^, Julien Massol^10^, Gwenhaël Colin^11^, Guillaume Schnell^12^, Stephan Ehrmann^13^, Romain Sonneville^3^, Nicolas De Prost^2^

#### ^1^CH Victor Dupouy Argenteuil, Argenteuil, France; ^2^Hôpital Henri Mondor, Créteil, France; ^3^Hôpital Bichat, Paris, France; ^4^Hôpital de Dieppe, Dieppe, France; ^5^CHU Rouen, Rouen, France; ^6^CHU Lyon, Lyon, France; ^7^CHU Lille, Lille, France; ^8^CHU Poitiers, Poitiers, France; ^9^CHU Louis Mourier, Colombes, France; ^10^CHU Cochin, Paris, France; ^11^Hôpital de La Roche sur Yon, La Roche Sur Yon, France; ^12^CH le Havre, Le Havre, France; ^13^CHU Tours, Tours, France; ^14^CH Montreuil, Montreuil, France

##### **Correspondence:** Damien Contou (damien.contou@ch-argenteuil.fr)

*Annals of Intensive Care* 2023, **13(Suppl 1):**FC-0258

**Rationale:** *Purpura fulminans* (PF) is a rare bacterial infection carrying a high mortality and morbidity. *Neisseria meningitidis* (Nm) is the leading responsible bacterium accounting for two thirds of PF. Obtaining a microbiological documentation of PF is crucial for confirming the diagnosis, as well as for adjusting the antibiotic therapy. It is also of paramount importance for public health interventions and postexposure chemoprophylaxis. Given the high susceptibility of *Nm* to β-lactam antibiotics, together with the high proportion of patients empirically treated before ICU admission, blood cultures may be sterile in half of the patients with meningococcal PF. Moreover, lumbar puncture has been shown to be of limited diagnostic value in this context. Our aim was to evaluate the diagnostic yield of SB in adult patients with meningococcal PF.

**Patients and methods/materials and methods:** We conducted a 17-year multicenter retrospective cohort study including adult patients admitted to the ICU for a meningococcal PF. SB was performed at the discretion of the intensivist on a purpuric lesion by using a punch-biopsy device after local anesthesia. SB was considered as contributive when culture grew *Nm* and/or when PCR was positive for *Nm*.

**Results:** Among the 306 patients admitted for PF, 195 had a meningococcal PF (64%) with SB being performed in 68 (35%) of them (Figure). SB was performed 1 [IQR 0–1] day after initiation of antibiotic therapy. Standard culture of SB was performed in 61/68 (90%) patients and grew *Nm* in 28 (46%) of them. *Nm* PCR on SB was performed in 51/68 (75%) patients and was positive in 50 (98%) of them. Among these 50 positive meningococcal-PCR, 5 were performed 3 days or more after initiation of antibiotic therapy. Finally, SB was considered as contributing in 60/68 (88%) patients, knowing that meningococcal-PCR was not performed in the 8 patients with a non-contributive skin biopsy. Identification of the meningococcal serogroup was obtained with SB in 48/68 (71%) patients.

**Discussion:** SB seems to be contributive in most of the patients with meningococcal PF, especially when a meningococcal PCR is performed (even 3 days after initiation of antibiotic therapy). We acknowledge that a standardized protocol with a systematic realization of SB combining conventional culture and meningococcal-PCR might have increased the proportion of patients with a contributive SB.

**Conclusion:** SB with conventional culture and meningococcal-PCR has a global sensitivity of 88% and should be systematically performed in case of suspected meningococcal PF in order to improve both the patient’s management and the public health interventions.

**Reference 1**

Contou D, Sonneville R, Canoui-Poitrine F, et al. (2018) Clinical spectrum and short-term outcome of adult patients with purpura fulminans: a French multicenter retrospective cohort study. Intensive Care Med 44:1502–1511. https://doi.org/10.1007/s00134-018

**Reference 2**

Staquet P, Lemee L, Verdier E, et al. (2007) Detection of Neisseria meningitidis DNA from skin lesion biopsy using real-time PCR: usefulness in the aetiological diagnosis of purpura fulminans. Intensive Care Med 33:1168–1172. https://doi.org/10.1007/s00134

**Compliance with ethics regulations:** Yes in clinical research.

**Figure 1 (abstract FC-258):**
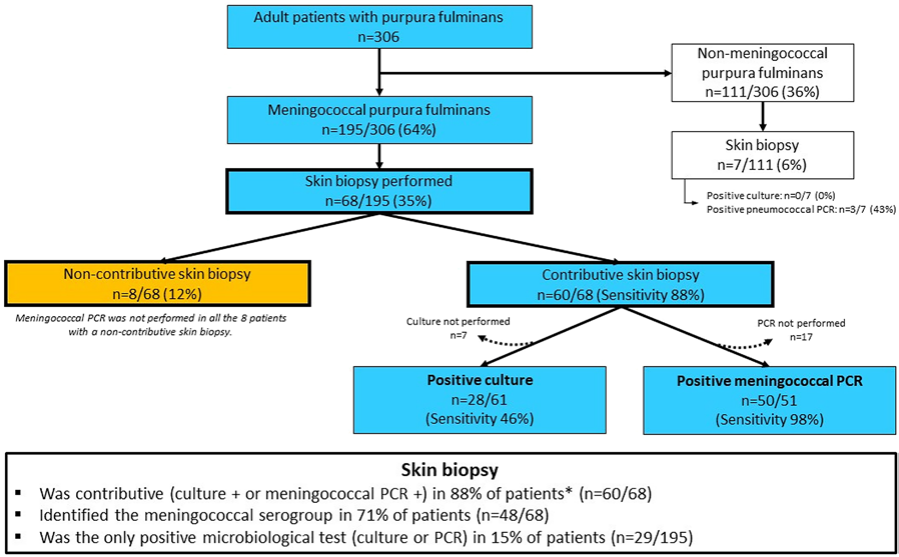
Flow chart of patients with meningococcal purpura fulminans. A skin biopsy was performed in 35% of patients (n = 68/195). The diagnostic yields of standard bacterial culture and meningococcal polymerase chain reaction (PCR) are displayed

## FC-259 Performance of BioFire® FilmArray® Blood Culture Identification Panel for the detection of bloodstream infections in burned patients

### Emna Hammas^1^, Hana Fredj^1^, Badis Tliti^1^, Sinda Bettaieb^1^, Sarra Zarrouk^1^, Amel Mokline^1^, Sarra Dhraief^1^, Lamia Thabet^1^, Amen Allah Mesaadi^1^

#### ^1^Centre de traumatologie et des grands brûlés Ben Arous, Tunisie, Tunis, Tunisie

##### **Correspondence:** Emna Hammas (emnahammas48@gmail.com)

*Annals of Intensive Care* 2023, **13(Suppl 1):**FC-0259

**Rationale:** Rapid initiation of targeted antimicrobial treatment is crucial in reducing mortality in severe septic states. BioFire® FilmArray® Blood Culture Identification Panel uses a multiplex PCR technique which allows the identification of pathogens within an hour, resulting in a rapid initiation of adequate antibiotic therapy. The aim of our study was to evaluate the performance of BioFire® FilmArray® Panel for the detection of bloodstream infections in burned patients with sepsis.

**Patients and methods/materials and methods:** We conducted a prospective, monocentric study, in the burn center of Ben Arous Hospital, Tunisia, from 11 January 2022 to 08 Mars 2022. Patients with at least one infection during which a blood culture was performed were included. Septic patients who could not benefit from a blood culture due to the unavailability of the blood culture bottle or the PCR test were excluded. For each positive blood culture, a molecular test using the BioFire Blood Culture Identification Panel (BCID) technique was performed in parallel with a conventional culture and an individual susceptibility test using conventional methods.

**Results:** In the study period 63 samples of 42 patients were examined. 92% of the patients were admitted for extended burns. The median age of the patients was 35 ± 20 years, sex ratio was 2.5 and the mean burned skin surface was 40%. Blood cultures were collected because of fever, hypothermia, or hypotension in 75%, 13% and 43% of cases respectively. The delay in receiving the results of the molecular test was one hour and 9 min compared to 86 h et 12 min for the classic blood culture, resulting in a gain of 85 h. The results obtained by the molecular test were concordant with those obtained by standard techniques in 60% of cases (66% in the case of monomicrobial cultures and 27% in the case of polymicrobial cultures). The isolated germs were staphylococcus in 33% of cases, enterobacteria in 32% of cases, Acinetobacter Baumannii in 27% of cases and Pseudomonas Aeruginosa in 11% of cases. Considering the result of the molecular test, the initial antibiotic therapy was maintained in 70% of the cases and was changed in the remaining 30%. We observed a 100% positive outcome for the septic episodes investigated by the molecular test.

**Conclusion:** The Biofire molecular technique produced results in accordance with standard techniques in the majority of cases, with a shorter delay which allows a rapid adaptation of the antibiotic treatment and therefore a favorable evolution of the septic episode.

**Compliance with ethics regulations:** Yes in clinical research.

## FC-260 Real-world use of cefiderocol in the EU and US for *Pseudomonas aeruginosa*: interim datafrom the PROVE study

### Romaric Larcher^2^, Jeffrey Pearson^3^, Stefano Verardi^1^, Andreas Karas^1^, Anne Santerre Henriksen^1^, Raymond Pecini^4^, Stephen Marcella^4^

#### ^1^Shionogi BV, London, Royaume-Uni; ^2^CHU Nimes, Nimes, France; ^3^Brigham and Women’s Hospital, Boston, Etats-Unis; ^4^Shionogi Inc, Florham Park, Etats-Unis

##### **Correspondence:** Anne Santerre Henriksen (anne.henriksen@shionogi.eu)

*Annals of Intensive Care* 2023, **13(Suppl 1):**FC-0260

**Rationale:** Gram-negative bacterial resistance, including that of *Pseudomonas aeruginosa* (PA) is an urgent global health problem. Cefiderocol (CFDC) is a novel siderophore antibiotic, active against multi-drug resistant (MDR) PA. PROVE is an ongoing international, retrospective study assessing the use of CFDC for Gram-negative infections (GNI). This describes interim results for PA.

**Patients and methods/materials and methods:** PROVE uses real world data extracted from patient charts with documented GNI. This report summarizes data as of November 2022. Cases were eligible if they received ≥ 72 h of CFDC. Patient characteristics, pathogens, hospital course, and treatment patterns are described. Outcomes include clinical cure and 30-day all-cause mortality (ACM) stratified by key patient characteristics.

**Results:** 194 patients with PA treated with CFDC (123 from the US, 71 from the EU) were included. The characteristics of the study population were similar in both regions. The median age was 56 years, interquartile range (44–65) with 66.5% males. Comorbidities were common. The most frequent sources of GNI were respiratory tract infections and primary or secondary bacteraemia; 32% of GNI were polymicrobial. Nearly half of patients received mechanical ventilation and more than one-third vasopressor support (Table 1). CFDC use was mainly targeted (n = 173, 91%) with known culture results of which 14% (n = 26) was salvage therapy. Empirical use accounted for 8%. Susceptibility testing was available for 72% of PA cultures of which 90% were susceptible by local criteria. Collectively, the clinical cure rate was 65% and 30-day ACM rate was 19% (Table 2). CFDC monotherapy was used in 57% with half the mortality of combination therapy. Outcomes were similar between bacteremia and respiratory infections: clinical cure was achieved66% and 64% respectively, and 30-day ACM was 22% for both.

**Conclusion:** This study highlights that most patients treated with CFDC for PA were complex with multiple comorbidities, and severe illness. Most were treated with monotherapy for respiratory infections and bacteremia. Two thirds achieved clinical resolution and 81% were alive 30 days after CFDC initiation.

**Compliance with ethics regulations:** Yes in clinical research.

**Table 1 (abstract FC-260):**
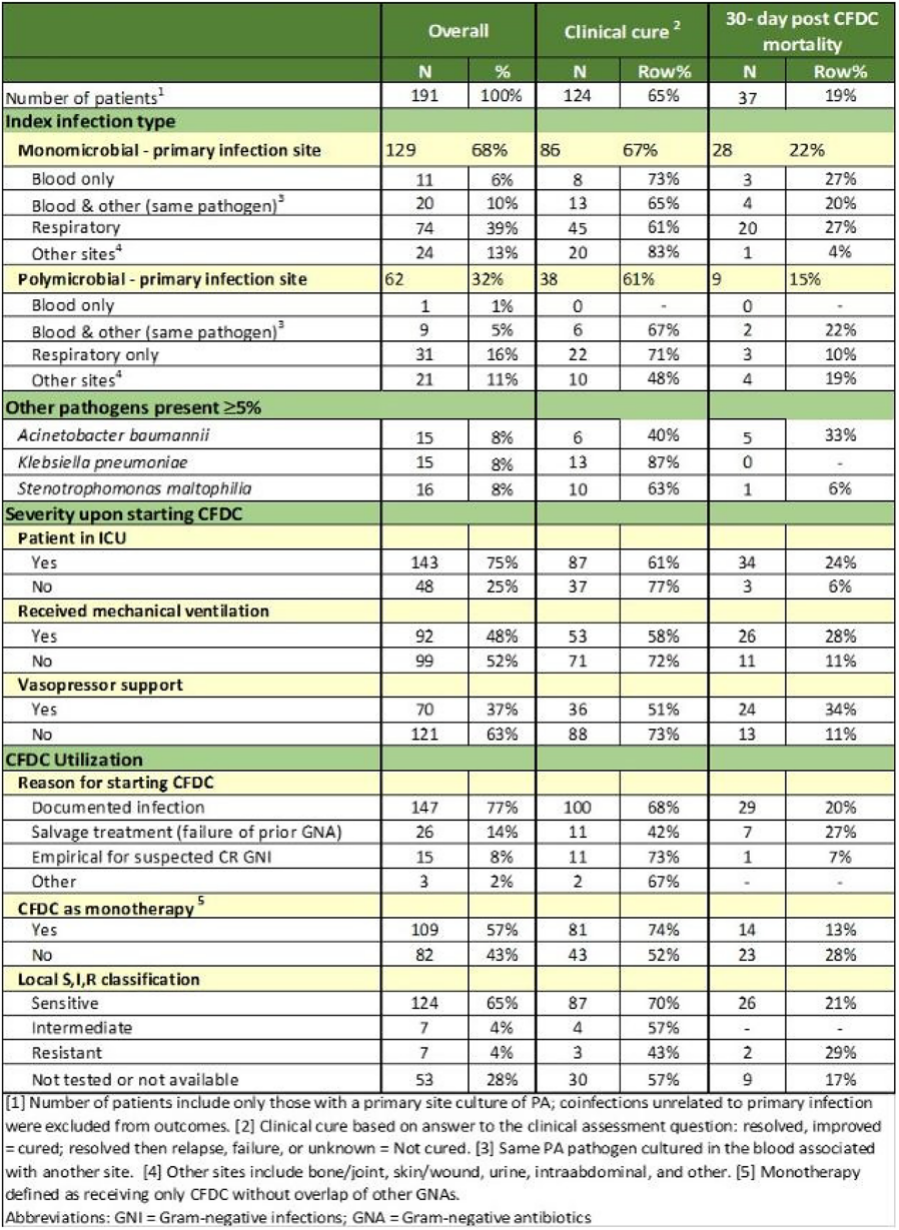
Outcomes by key characteristics in patients with *Pseudomonas aeruginosa*

## FC-261 Real-world use of cefiderocol in the EU and US for *Acinetobacter baumannii*: interim data from the PROVE study

### Jean Francois Timsit^2^, James Sanders^3^, Stefano Verardi^1^, Andreas Karas^1^, Anne Santerre Henriksen^1^, Bin Cai^4^, Hannah Russo^4^, Stephen Marcella^4^

#### ^1^Shionogi BV, London, Royaume-Uni; ^2^Hôpital Bichat, Paris, France; ^3^University of Texas, Southwestern Medical Center, Dallas, Etats-Unis; ^4^Shionogi Inc, Florham Park, Etats-Unis

##### **Correspondence:** Anne Santerre Henriksen (anne.henriksen@shionogi.eu)

*Annals of Intensive Care* 2023, **13(Suppl 1):**FC-0261

**Rationale:** Acinetobacter baumannii (AB) infections are difficult to treat with limited treatment options. Cefiderocol (CFDC), a novel siderophore antibiotic, has demonstrated potent in vitro activity against carbapenem-resistant AB (CRAB). PROVE is an ongoing international, retrospective study of CFDC use for Gram-negative infections (GNI). This report describes interim results for 98 patients with AB infections across EU and US sites.

**Patients and methods/materials and methods:** Data was abstracted from patient charts with documented GNI. Cases were eligible if they received ≥ 72 h of CFDC in a real-world setting. Charts were abstracted from commercial availability to November 2022. Patient characteristics, pathogens, hospital course, and treatment patterns were described. Outcomes of clinical cure and 30-day all-cause mortality (ACM) were stratified by key characteristics.

**Results:** 98 patients with AB were treated with CFDC (71 from US, 27 from the EU). Respiratory infection and bacteremia were the most frequent infections. Collectively, nearly a third of patients received vasopressor support associated with nearly three times the risk of mortality (Table 1). CFDC use was targeted as first treatment or as salvage therapy in 93%. Monotherapy was used in 41%, and combination therapy in 59%: aminoglycosides, n = 2, carbapenems, n = 4, polymyxins, n = 17, tetracyclines, n = 26, and others, n = 29. Susceptibility was available for 49%; 81% were susceptible by local criteria. The median duration of CFDC was 11.5 days. CFDC was stopped for clinical cure, clinical failure, or adverse events in 50, 4, and 2 cases respectively. Clinical cure rate was 60% and 30-day ACM rate was 24% (Table 2). Polymicrobial infections (36%) had similar clinical cure as monomicrobial (63% vs. 59%) and mortality (26% vs. 23%). Respiratory infections without secondary bacteremia had greater clinical cure (62% vs.42%) and lower ACM (19% vs. 42%) than bacteremia, and together accounted for 63% of CFDC treatment.

**Conclusion:** In this study, approximately half of patients treated with CFDC for AB required organ support. CFDC was used mostly to treat respiratory infections and bacteremia. Greater cure rates were achieved for respiratory infections.

**Compliance with ethics regulations:** Yes in clinical research.

**Table 2 (abstract FC-261):**
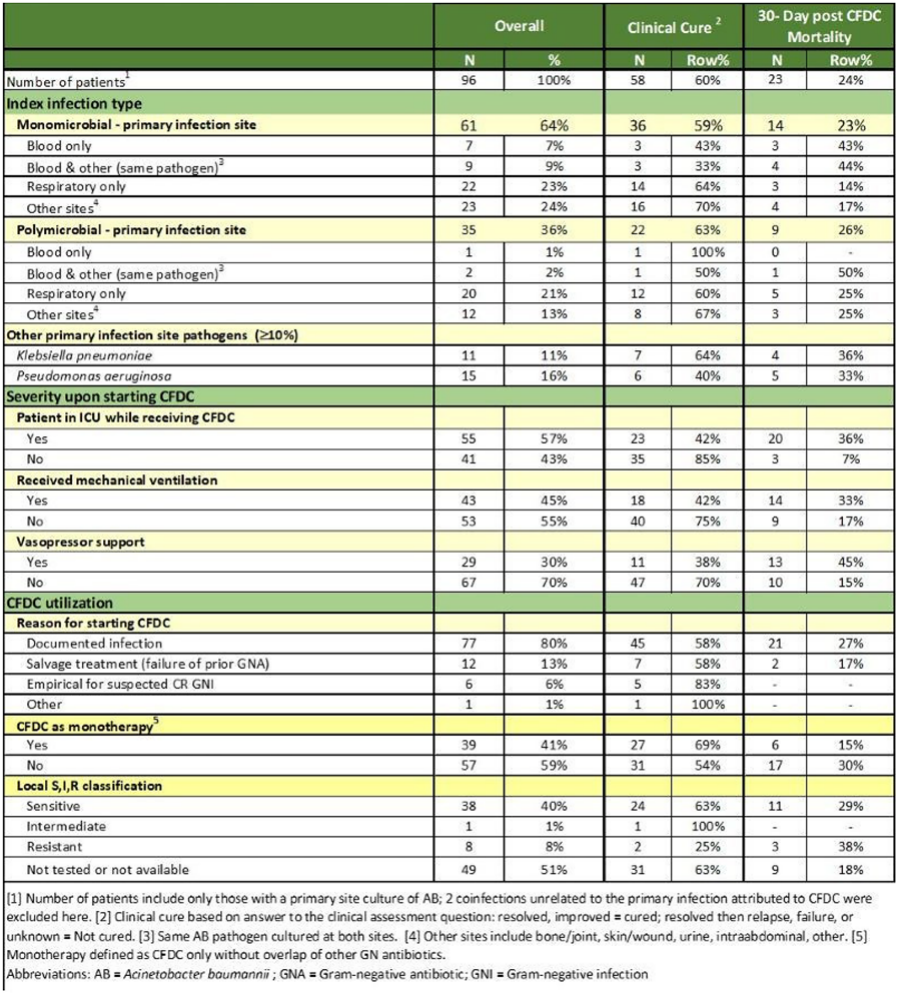
Outcomes by key characteristics in patients with *Acinetobacter baumannii*

## FC-262 Toxic epidermal necrolysis: epidemiological evolutionary and prognostic aspects

### Amal Aloui^1^, Hana Fredj^1^, Sarra Zarrouk^1^, Bahija Gasri^1^, Manel Ben Saad^1^, Imen Jami^1^, Bahija Gasri^1^, Amel Mokline^1^, Amen Allah Messadi^1^

#### ^1^Centre de Traumatologie et des Grands brûlés, Ben Arous, Tunisie

##### **Correspondence:** Hana Fredj (fredjhana@yahoo.fr)

*Annals of Intensive Care* 2023, **13(Suppl 1):**FC-0262

**Rationale:** Toxic epidermal necrolysis (TEN) is a life threatening, rare and severe mucocutaneous disease. Almost all cases are drug-induced. This study aimed to describe the epidemiological, etiological, clinical, therapeutic and evolutionary data of TEN and to determine the predictive factors of mortality.

**Patients and methods/materials and methods:** It was a retrospective and descriptive study conducted within the intensive burn care unit over a period of 10 years, including all hospitalized cases of TEN.

**Results:** Fifty cases of NET were included, with an incidence of 1.6. The sex ratio (H/F) was 0.56. The mean age was 41 ± 15 years. None of the patients had a history of drug allergy. Almost 88% of patients had a history of disease. Self-medication was recensed in 24% of cases. 92% of patients were admitted in our department within 8.9 days. The most often implicated drugs are anticonvulsants (34%), urate-lowering drugs (20%) and antibiotics (18%). The mean time to onset after drug administration was 11 days. The average skin area detached was 38 ± 18, 4%. Mucous membrane was affected in all cases. Systemic signs occurred in 74% of cases, especially renal, respiratory and hematological disorders. A pharmacovigilance survey was carried out in 88% of patients. Skin biopsy is performed in 76% of patients. The main principles of symptomatic therapy include general measures and local mucocutaneous treatment. The evolution was favorable among 25 patients (50%) with rapid cutaneous healing (less than 3 weeks) in 32% of cases. Infection is the main complication especially of skin (74%). The mortality rate was 50%, most probably due to septic shock. The prognosis of (SL) remains poor, depending on several adverse factors including advanced age > 43 years, skin detachment > 39%, ICU admission time > 5.5 days, need for mechanical ventilation, and infectious complications were factors of poor prognosis.

**Conclusion:** NET is a rare with important morbidity and mortality. Early management in intensive care and compliance with asepsis rules could improve the prognosis.

**Compliance with ethics regulations:** Yes in clinical research.

## FC-263 Cerebral venous thrombosis: clinical presentations and factors associated with mortality

### Rabeb Hammami^1^, Mahmoud Marzouk^1^, Maryem Ben Amor^1^, Rym Karaborni^1^, Sabeur Thamlaoui^1^, Nader Baffoun^1^, Chokri Kaddour^1^

#### ^1^Institut National De Neurologie, Tunis, Tunisie

##### **Correspondence:** Rabeb Hammami (hammamirabeb2@gmail.com)

*Annals of Intensive Care* 2023, **13(Suppl 1):**FC-0263

**Rationale:** Cerebral venous thrombosis (CVT) is a serious and life-threatening condition because of intracranial hypertension and the ischemic and hemorrhagic softening that it causes. The diagnosis is evoked in front of clinical and anamnestic arguments. Confirmation is performed by radiology. The aim of our study was to clarify the different clinical and topographical presentations of cerebral venous thrombosis and to study the factors associated with mortality.

**Patients and methods/materials and methods:** This was a retrospective descriptive study conducted in our intensive care unit over a 20-year period from 2002 to 2021. Patients with imaging-confirmed CVT were included. The following data were collected: age, gender, medical history, clinical presentation, time from onset of symptoms to diagnostic confirmation, intracranial topography of the stroke, length of stay in the ICU, and mortality. Factors associated with mortality were investigated.

**Results:** 93 patients were selected for the study. The mean age was 32.3 ± 11.92 years. The sex ratio was 0.2: 16 men (17.20%) and 77 women (82.79%). Diabetes was found in 9.67% of patients. Hypercoagulability conditions were noted, namely pregnancy in 14%, systemic diseases in 7.5%, postpartum in 17.2%, and the use of oral contraceptives in 26.2% of patients. Other risk situations were identified such as thrombophilia, central nervous system infections, and postoperative, traumatic, and neoplastic contexts. Symptoms are dominated by headache in 75.3%, convulsions in 74.2%, neurological deficit in 69.9%, coma in 47.3%, and visual disturbances in 15.1% of cases. in 15.1% of cases. The average time between the onset of symptoms and diagnosis was 7.56 days ± 6.49. The diagnosis was confirmed by MRI in 54.8% of cases, injected CT in 37.6%, non-injected CT in 5.4% and angiography in 2.4% of cases. The preferred topography was the superior longitudinal sinus in 67.7% of cases and the lateral sinus in 57% of cases. The average duration of mechanical ventilation was 5.17 days ± 6.34. The average length of stay was 10.38 days ± 11. The mortality rate was 36.6%. The factors associated with mortality in univariate analysis are shown in Table 1.

**Conclusion:** CVT is a serious condition. The clinician must suspect the diagnosis when faced with a range of anamnestic and clinical arguments. This will allow the diagnosis to be made early, imaging to be indicated, curative treatment to be started and mortality to be reduced.

**Compliance with ethics regulations:** Yes in clinical research.

## FC-264 ICH score and vital prognosis of hemorrhagic strokes

### Nabil Sidi Aissa^1^, Mourad Goulmane^1^, Khadidja Rezigua^1^, Nabil Tabet Aoul^1^

#### ^1^CHU Oran, Oran, Algerie

##### **Correspondence:** Nabil Sidi Aissa (sidiaissa_nabil@yahoo.fr)

*Annals of Intensive Care* 2023, **13(Suppl 1):**FC-0264

**Rationale:** Cerebral hematoma is a serious pathology with a mortality rate of 35 to 52% at 30 days. The ICH score establishes the vital prognosis of these hematomas at 1 month. Our objective was to measure the applicability of the ICH score in patients admitted to the medical emergencies of the Oran University Hospital for hemorrhagic stroke and to identify the factors associated with the mortality of cerebral hematomas in our context.

**Patients and methods/materials and methods:** The study was descriptive and analytical retrospective covering all patients hospitalized in the emergency hospital of the Oran University Hospital in 2022 for a spontaneous cerebral hematoma.

**Results:** We included 85 patients with an average age of 64 years (30 to 92 years). Mortality at D30 was 30%, with more than 40% of deaths occurring after the first week. The ICH score ranged from 0 to 4. A Glasgow score < 12, systolic blood pressure > 200 mmHg, presence of decubitus complications and hyperglycaemia on admission > 10 mmol/l were significantly associated with the risk of death.

**Conclusion:** The ICH score did not accurately predict the prognosis of hematomas hospitalized in our department.

**Compliance with ethics regulations:** Yes in clinical research.

## FC-265 Aneurysmal subarachnoid hemorrhage: clinical features, risk factors, management and outcomes

### Rabeb Hammami^1^, Mahmoud Marzouk^1^, Sabeur Thamlaoui^1^, Nader Baffoun^1^, Chokri Kaddour^1^

#### ^1^Institut National De Neurologie, Tunis, Tunisie

##### **Correspondence:** Rabeb Hammami (hammamirabeb2@gmail.com)

*Annals of Intensive Care* 2023, **13(Suppl 1):**FC-0265

**Rationale:** With high rates of death and lifelong impairment, aneurysmal subarachnoid hemorrhage is a major global health concern. It’s described as sudden bleeding into the subarachnoid space when a brain aneurysm bursts and leaks. The aim of our study was to describe clinical characteristics, itemize the management of ruptured aneurysms, and look into prognostic and risk factors.

**Patients and methods/materials and methods:** We carried out a descriptive retrospective study over a period of 12 months, from January 2021 to December 2021, including patients with aneurysm subarachnoid hemorrhage. We gathered information on sociodemographic data, medical history, actual treatment, physical examination, paraclinical data, management of ruptured aneurysms, and postoperative outcomes.

**Results:** Our study included 98 patients. The mean age was 54.8 ± 11.8 years. 49% were male (sex ratio = 0.98). 45.1% of participants were smokers and 6.1% were receiving anti-coagulant or anti-aggregation therapy. Hypertension was found as the main risk factor in the development of cerebral aneurysms (38.6%) while just 6.86% of patients had physical or emotional triggers. The most common onset symptom, headache, was reported in 82.9% of cases. Regarding clinical manifestations, 10% of patients experienced seizures, 40.8% showed evidence of intracranial pressure syndrome, and 14.3% had focal neurological abnormalities, primarily hemiparesis. GCS was ≤ 14 in 39.8% of cases whereas 33.7% had a WFNS score ≥ 2. In terms of diagnosis, cerebral CT was performed systematically in all cases, and only 23.5% of patients underwent cerebral angiography. The Fisher scale was greater than 3 in 80.6% of cases and a giant aneurysm was found in 4% of cases. The most frequent locations were successively the anterior communicating artery (36.7%), the middle cerebral artery (20.4%), the internal carotid artery (14.3%), and the posterior cerebral artery (11.2%). 71.4% of patients had received endovascular treatment (endovascular coiling or stenting) whereas 10.2% had surgical clipping. Mechanical ventilation was used in 22 patients mostly for neurological reasons. The average stay was 8 days and the main complication was vasospasm in 23.2% of cases. The mortality rate was 34.7%. Both the WFNS scale (p = 0.005, r = 0.96) and the anterior communicating artery aneurysm (p = 0.009, r = 0.92) had a strong correlation to mortality among the predictive markers.

**Conclusion:** Numerous risk factors including age, tobacco use, anticoagulant and aggregate medication, and hypertension, had been studied. Both the WFNS scale and the anterior communicating artery aneurysm were associated with a high risk of mortality.

**Compliance with ethics regulations:** Yes in clinical research.

## FC-266 Chronic subdural hematoma: clinical features, risk factors and prognosis

### Rabeb Hammami^1^, Mahmoud Marzouk^1^, Maryem Ben Amor^1^, Sabeur Thamlaoui^1^, Nader Baffoun^1^, Chokri Kaddour^1^

#### ^1^Institut National De Neurologie, Tunis, Tunisie

##### **Correspondence:** Rabeb Hammami (hammamirabeb2@gmail.com)

*Annals of Intensive Care* 2023, **13(Suppl 1):**FC-0266

**Rationale:** One of the most prevalent diseases in neurosurgery and among the elderly, chronic subdural hematoma has been on the rise over time. A greater understanding of the numerous risk factors and the ability to forecast patients' prognoses are crucial. Our study objectives included describing clinical characteristics and looking into prognostic and risk factors.

**Patients and methods/materials and methods:** We carried out a descriptive retrospective study over a period of 3 months, from May 2021 to July 2021, in the neurosurgery department of the national institute of neurology. We gathered information on sociodemographic data, medical history, actual treatment, physical examination, paraclinical data, and postoperative outcomes.

**Results:** 100 patients were studied. 75% of participants were over 60, with a mean age of 69. 80% were male (sex ratio = 4). Most of the patients were from northwestern Tunisia (54%). 58% of participants had a history of head trauma, 44% smoked, and 35% were receiving anti-coagulant or aggregate therapy. 35% of people had hypertension precursors, while 24% had diabetes precursors. Regarding clinical manifestations, 21% of patients had intracranial pressure syndrome, 21% had linguistic dysfunction, and 61% of patients had headaches. The most prevalent physical symptom was hemiparesis (82% of cases). 81% had a GCS of 15 while 73% had a Markwalder grading scale of 1. In all patients, the urea/creatinine ratio was determined. As shown in other studies, the urea/creatinine ratio is connected to the degree of hydration. 32% had a ratio higher than 80. The hematoma was on the left side in 55% of cases. 20% were bilateral and 25% were right-sided. 95% of individuals underwent surgery, 18% underwent it in less than 24 h and 82% underwent it after 24 h (the longest delay was 7 days). The most typical therapy was trepanation (93%). 83% of patients had a full recovery, 12% experienced intellectual or motor sequelae, and 4% passed afterward. 2% had a site infection, and 4% experienced recurrence. GCS was the sole predictive indicator identified in our study that was associated with a poor outcome (p = 0.001; r = − 0.6).

**Conclusion:** Numerous risk factors including age, tobacco use, anticoagulant and aggregate medication, and hypertension, had been studied. GCS was the sole factor in predicting mortality.

**Compliance with ethics regulations:** Yes in clinical research.

## FC-267 Management of traumatic brain injury admitted to the polyvalent intensive care unit of the University Hospital of Brazzaville (Congo)

### Marie Elombila^1,2^, Hugues Brieux Ekouele Mbaki^1,2^, Mayick Chris Mpoy Emy Monkessa^1^, Peggy Dahlia Leyono Mawandza^1,2^, Gilles Niengo Outsouta^1^, Marina Aurole Nde Ngala^1^, Gilbert Fabrice Otiobanda^1,2^

#### ^1^CHU de Brazzaville, Brazzaville, Republique Du Congo; ^2^Université Marien Ngouabi, Brazzaville, Republique Du Congo

##### **Correspondence:** Marie Elombila (elombila@gmail.com)

*Annals of Intensive Care* 2023, **13(Suppl 1):**FC-0267

**Rationale:** Traumatic Brain Injury (TBI) is a major public health problem causing significant morbidity and mortality. The objective of the study was to describe the management of TBI’s admitted to the polyvalent intensive care unit of University Hospital of Brazzaville.

**Patients and methods/materials and methods:** It was a descriptive, retrospective study over a 32-month period, from December 2015 to July 2018. The variables studied were: socio-demographic, clinical and therapeutic.

**Results:** A total of 60 patients were admitted for TBI, representing a hospital incidence of 3.9%. The mean age was 37.05 ± 17.02 years with a sex ratio of 4.45. In 75% of the cases, the mechanism of the TBI was a road traffic accident. Severe TBI accounted for 61.7%. Cerebral contusions dominated the head CT-scan in 40% of the cases. Trauma associated with TBI was dominated by limb trauma (21.7%) followed by chest trauma (20%). Median MAP was 86 mmHg [Q1: 73; Q3: 106], SpO_2_ was below 92% in 33.3% of cases. Mechanical ventilation was performed in 48.3% of the patients with a median duration of ventilation of 4 days [Q1: 3; Q3: 5.5]. Sedation was achieved by midazolam and fentanyl in 32.8% and 47.5% of cases respectively. Osmotherapy was initiated in 15% of patients and noradrenaline in 40% of patients. Surgical intervention was performed in 13.3% of the patients. The mortality rate was 58.3%.

**Conclusion:** TBI affects young male subjects. TBI admitted to the ICU are generally severe. The mortality of TBI is high. Early and adequate management would reduce this mortality.

**Compliance with ethics regulations:** Yes in clinical research.

## FC-268 Clinical predictors of poor outcome in traumatic brain injury

### Khaoula Ben Ismail^1,2^, Malek Kharrat^1,2^, Mariem Chaabane^1,2^, Fatma Essafi^1,2^, Najla Ben Slimene^1,2^, Imene Talik^1,2^, Moez Kaddour^1,2^, Takoua Merhabene^1,2^

#### ^1^Intensive care unit, Regional Hospital Zaghouan.Tunisia, Zaghouan, Tunisie; ^2^Faculty of Medicine of Tunis, University Tunis El Manar, Tunis, Tunisia, Tunis, Tunisie

##### **Correspondence:** Malek Kharrat (kharratmalek1@gmail.com)

*Annals of Intensive Care* 2023, **13(Suppl 1):**FC-0268

**Rationale:** Traumatic brain injury patients are characterized with severe clinical presentation requiring critical care in intensive care unit (ICU). They are usually associated with poor outcome and high mortality. Aim: to identify prognostic factors associated with poor outcome in patients admitted to the ICU for severe Traumatic brain injury.

**Patients and methods/materials and methods:** A retrospective study including severe Traumatic brain injury patients admitted to our ICU over the period of 4 years (from January 1st 2019 to December 31st 2022). Epidemiological, biological and radiological and evolving data were collected.

**Results:** Overall 57 patients with severe head injury following public road accidents were included. A male predominance was noted (Gender ratio 1.9). Mean age was 38.9 ± 16.7 years. Average APACHE II and SAPS II scores were 18 and 42. The most common tomographic abnormalities found in the initial cerebral imaging were cerebral contusion and subarachnoid hemorrhage in 36.8% of cases, cranial fractures in 33.3%, subdural hematoma in 28% and extradural hematoma in 8.7%. A thoracic trauma was associated in 59.6% of cases and abdominal trauma in 8%. Only two patients needed an emergent neurosurgery. A state of shock was diagnosed in 38.6% (n = 22) of patients. It was secondary to a massive hemorrhage in 13 patients, sepsis in 7 patients and one patient sustained a cardiogenic choc. Intubation and invasive mechanical ventilation were necessary in 39 (68.4%) patients with mean duration of 10.44 days. Nine patients were extubated and 7 needed tracheostomy. The most common complication was infection noted in 61.4% of patients, followed by fever (49.1%) and acute kidney injury (19.6%). Mean duration of ICU stay was 6 days with extremes ranging from one to 96 days. Mortality rate was 36.8%. The surviving patients were significantly younger (34.3 years versus 46.7 years; p = 0.006) and had lower C-reactive protein levels since the first day of hospitalization (35.9 mg/l versus 88.3 mg/l; p = 0.009) and in the fifth day (63 mg/l versus 219.7 mg/l; p = 0.02). In univariate analysis, prognostic factors associated with poor outcome were: Invasive mechanical ventilation (p < 10–3), acute kidney injury (p = 0.001), non-explained fever (p = 0.010.) and the prescription of probabilistic antibiotics (p = 0.014). No independent risk factor was identified in the multi variant analysis.

**Conclusion:** Mechanical ventilation, acute kidney injury, fever non-related to an infection and the prescription of antibiotics without bacteriological confirmation were associated with poor outcome in severe traumatic brain injury patients.

**Compliance with ethics regulations:** Yes in clinical research.

## FC-269 Delirium in critically ill COVID-19 patients: risk factors and impact on outcome

### Dhouha Hamdi^1,2^, Imen Ben Saida^1,2^, Nabil Bouguezzi^1,2^, Rym Chelbi^1,2^, Rihab Rajah^1^, Azer Yaacoub^1,2^, Khaoula Meddeb^1,2^, Mohamed Boussarsar^1,2^

#### ^1^University of Sousse, Faculty of Medicine of Sousse, Sousse, Tunisie; ^2^Farhat Hached University Hospital, Medical Intensive Care Unit, Research Laboratory “Heart Failure”, LR12SP09, 4000, Sousse, Tunisia, Sousse, Tunisie

##### **Correspondence:** Nabil Bouguezzi (dr_nabil@live.fr)

*Annals of Intensive Care* 2023, **13(Suppl 1):**FC-0269

**Rationale:** Delirium is a serious problem which often accompanyiescritical illness and can be detrimental to the safety of the patient. Data regarding delirium in critically ill patients with COVID-19 is scarce. We aimed to investigate the frequency, risk factors for delirium in Critically Ill COVID-19 patients and its effects on clinical outcomes.

**Patients and methods/materials and methods:** It is a retrospective observational study conducted from January 2021 to September 2021, in a Medical ICU. Delirium was defined according to the confusion assessment method of ICU (CAM-ICU). Patients with a not to resuscitate decision, or had a history of dementia or psychosis, or who were kept sedated during all their ICU stay were excluded because of the difficulty to assess delirium in this group. Information regarding demographic and clinical characteristics of Critically Ill COVID-19 patients were obtained from medical records. Clinical data were compared between patients with and without delirium. Multivariate analysis was performed to evaluate risk factors for delirium and clinical outcomes.

**Results:** During the study period, 225 patients were assessed and 193 (85.7%) met the inclusion criteria. Patients’ characteristics were: mean age, 64 ± 14.9 years; age > 65 years, 104 (53.9%); female gender, 81 (42%); median Charlson index, 3 [2–4]; median SAPSII, 27 [21; 33]; invasive mechanical ventilation (IMV), 92 (47.7%) and vasopressors use, 99 (51%). Using the confusion assessment method of ICU (CAM-ICU), 62 (32.1%) had delirium. The most common subtype of delirium seen in the present study was hyperactive type, 43 (69%), followed by hypoactive subtype, 16 (26%) and few patients had mixed subtype of delirium, 3 (4.2%). There was no significant difference in duration of IMV between the two groups; (7 [4–13] vs 9 [5–13] p = 0.393), patients who were diagnosed with delirium had significantly longer length of ICU stay (14 [9; 18] vs 7 [5; 11] days, p < 0.001) and higher mortality rate (61.3% vs 42%, p = 0.000). Univariate analysis revealed the following factors to be associated to delirium respectively for delirium and controls: age ≥ 65 years, (56.5% vs 41.2%, p = 0.047); SAPSII, (30 [24; 38] vs 24 [19; 38], p < 0.001); IMV (74.2% vs 35.9%, p < 0.001); vasopressors use (75.8% vs 39.7%, p < 0.001), use of physical restraint, (82.3% vs 30.5%, p < 0.001). Multivariate regression model identified the following factors as independently associated to delirium: SAPS II, (OR, 1.039; 95% CI, [1.006–1.074]; p = 0.022) and midazolam use (OR, 4.820; 95% CI, [2.35–9.87]; p < 0.001).

**Conclusion:** Delirium seems to be common in critically covid-19 patients. Higher SAPS II score and midazolam use were determined as predictors of delirium.

**Compliance with ethics regulations:** Yes in clinical research.

## FC-270 Bench study of a spontaneous breathing trial with its different modalities

### Guillaume Rigault^1,5^, Claude Guerin^2,4,6^, Florian Sigaud^1^, Laurent Argaud^2,4^, Louis Marie Galerneau^1,5^, Nicolas Terzi^3^

#### ^1^Médecine intensive-Réanimation CHU Grenoble, La Tronche, France; ^2^Médecine intensive-Réanimation CHU Lyon, Lyon, France; ^3^Médecine intensive-Réanimation CHU Rennes, Rennes, France; ^4^Université Lyon, Lyon, France; ^5^Université de Grenoble Alpes, Grenoble, France; ^6^Institut Mondor de Recherche Biomédicale, inserm 955 CNRS ERL 7000, Créteil, France

##### **Correspondence:** Guillaume Rigault (grigault@chu-grenoble.fr)

*Annals of Intensive Care* 2023, **13(Suppl 1):**FC-0270

**Rationale:** Spontaneous breathing trial (SBT) is the final step of weaning from invasive mechanical ventilation (MV). SBT is aimed at predicting work of breathing (WOB) after extubation and, most importantly, a patient’s eligibility for extubation. The optimal SBT modality remains debated. High-flow oxygen (HFO) therapy has been tested during SBT in clinical study only, which is why no definite conclusion can be drawn on its physiological effects on the endotracheal tube. Our objective was to assess inspiratory tidal volume (VTi), total PEEP, and WOB across three different SBT modalities: T-piece, 40 L/min, and 60 L/min HFO, on a bench.

**Patients and methods/materials and methods:** The ASL5000 lung model was set with three conditions of resistance and linear compliance, three inspiratory efforts (Low, Normal and High), each at two respiratory rates (Low and High for 20 and 30 breaths/min respectively). Three SBT modalities were evaluated: a T-piece and HFO with 40 L/min and HFO with 60 L/min. Pairwise comparisons and Quasi Poisson generalized linear model comparing SBT modalities were performed.

**Results:** VTi, total PEEP, and WOB differed from one SBT modality to another. VTi remained higher in T-piece than HFO independently of mechanical condition, effort intensity and respiratory rate (P-value < 0.01 in each comparison). WOB adjusted by the VTi was significantly lower during SBT performed with HFO than when performed with T-piece (P-value).

**Conclusion:** With the same effort intensity and respiratory rate, VTi was higher in T-piece than in the other modalities. Compared with T-piece, WOB was significantly lower in HFO condition, and higher flow was a benefit. Based on the results, HFO as a SBT modality would seem to require clinical testing.

**Compliance with ethics regulations:** Yes in animal testing.

## FC-271 A nurse-driven protocol for early weaning from mechanical ventilation in critically ill patients with acute respiratory failure. A feasibility and safety study (the NURSES-WEAN study)

### Jean-Adoumngar Moussanang^1^, Ophélie Marcq^1^, Guillaume Thery^1^, Sarah Sellam^1^, Olfa Hamzaoui^1,2^, Damien Jolly^3^, Bruno Mourvillier^1^, Antoine Goury^1^

#### ^1^Service de Médecine Intensive – Réanimation Polyvalente, Hôpital Robert Debré, Centre hospitalo-universitaire de Reims, Reims, France; ^2^Unité HERVI "Hémostase et Remodelage Vasculaire Post-Ischémie" - EA 3801, Université de Reims Champagne-Ardenne, Reims, France; ^3^Pôle Recherche et Santé Publique, Hôpital Robert Debré, Centre hospitalo-universitaire de Reims, Reims, France

##### **Correspondence:** Jean-Adoumngar Moussanang (j.adoumngar@live.fr)

*Annals of Intensive Care* 2023, **13(Suppl 1):**FC-0271

**Rationale:** Prolonged mechanical ventilation (MV) exposes patients to increased risk of infection and mortality. A weaning protocol (WP) reduces the duration of MV but may be limited by the availability of the medical and nurse team, especially during periods of health crisis such as COVID-19 pandemic. This leads to impaired weaning process and prolonged MV for patients. We assessed the feasibility and safety of an early nurse-driven WP from the start of spontaneous ventilation (SV) until extubation in patients admitted for acute respiratory failure (ARF).

**Patients and methods/materials and methods:** This monocentric, prospective pilot study included patients admitted for ARF and under MV for more than 48 h. Sedation was left at the clinician’s discretion. Weaning protocol began when the following criteria were present: PEEP ≤ 8 cmH_2_O, PaO_2_/FiO_2_ ratio ≥ 150 or SaO_2_ ≥ 90% with FiO_2_ ≤ 0.5 and a successful completion of a 30-min pressure support (PS) test. Every 3 h, the nurse decreased PS by 2 cmH_2_O, fraction inspired in oxygen (FiO_2_) of 5% and positive end-expiratory pressure (PEEP), as long as the patient was in the predefined “breathing comfort zone” (for ventilatory and hemodynamics parameters). When the minimum level of PS (8 cmH_2_O) and PEEP (5 cmH_2_O) was reached, a PS was performed using a T-piece for 30 min and the patient was extubated if successful.

**Results:** Among the 39 patients included, the median age was 66 [60–73] years and median SAPSII 42 [34–55]. 31 patients had COVID-19 pneumonia, 4 bacterial pneumonia and 4 extra-pulmonary ARF. The median duration of MV before the beginning of WP was 9 [4–16] days and the WP was continued for 13 [4–20] days. Regarding the nurses’ compliance with the protocol, the percentage of time spent in agreement with the protocol was 69% [50–76]. Median satisfaction with the protocol was 8/10 [7.75–9] for nurses and 8.5/10 [8–9] for physicians on a visual analogic scale. Concerning adverse events, we encountered 4/39 (10%) auto-extubation and 7/39 (18%) extubation failure. The length of ICU stay was 32 days [14–42]. Mortality at day 90 was 13/39 (33%).

**Conclusion:** Our WP seems safe with good acceptance and compliance in the range of other published studies (40–95%). The nurse’s compliance could be improved but the WP had been conducted in patients with very long duration of MV and implemented in time of health crisis. We plan a second analysis with a preliminary cohort to compare WP efficacy and adverse events.

**Compliance with ethics regulations:** Yes in clinical research.

## FC-272 Risk factors for prolonged weaning from mechanical ventilation in patients with Covid-19

### Hajer Nouira^1^, Soumaya Chtioui^1^, Rihab Rajah^1^, Oussama Jaoued^1^, Mohamed Fekih Hassen^1^, Habiba Ben Sik Ali^1^, Souheil Elatrous^1^

#### ^1^Hôpital Taher Sfar Mahdia, Mahdia, Tunisie

##### **Correspondence:** Hajer Nouira (nouirahajer@gmail.com)

*Annals of Intensive Care* 2023, **13(Suppl 1):**FC-0272

**Rationale:** Many patients with COVID-19 pneumonia required invasive mechanical ventilation (IMV) in intensive care unit (ICU). They may later experience difficulties in weaning from IMV. Minimizing the duration of the ventilatory support is a crucial way to reduce complications. This study aimed to investigate the weaning rate and the factors associated with prolonged weaning from IMV among Critically ill patients with COVID-19 pneumonia.

**Patients and methods/materials and methods:** This retrospective study was conducted between September 2020 and December 2021. Patients admitted to the ICU with covid-19 pneumonia and required mechanical ventilation for more than 12 h were included. The diagnosis of Covid-19 was performed with reverse-transcription polymerase chain reaction (RT-PCR). Prolonged weaning was defined if the weaning process in the patient required more than 7 days, after the first spontaneous breathing trial. Patients who met the inclusion criteria were divided into two groups: Group 1: Prolonged weaning (> 7 days) Group 2: Non-prolonged weaning (< 7 days). A logistic regression analysis was used to identify factors associated with prolonged weaning.

**Results:** A total of 449 patients were admitted to the ICU with covid-19 pneumonia. Among them 203 patients required mechanical ventilation. Forty-one patients were extubated (the weaning rate was 20%). Out of these, 11 patients had non-prolonged weaning and 30 patients experienced prolonged weaning. The most common co-morbidities were hypertension in 39% and diabetes mellitus in 38%. The median of SOFA score and APACHEII score were respectively 4 (3–4) and 10 (6–14). The characteristics of the two groups were comparable (age, sex and comorbidities). In the univariate analysis, patients with prolonged weaning from IMV had a less partial arterial oxygen pressure (PaO_2_) to inspired fraction of oxygen (FiO_2_) ratio at day1 of ventilation (124 (84–165) vs 212 (140–400) p = 0.006) In addition, they received Neuromuscular blocker for a longer time [8 days (4–12) vs 2.5 days (2–4) p = 0.002] and they were more likely to present ventilator associated pneumonia: 63.3% vs. 18.2%, p = 0.015. In the regression multivariate analysis, the duration of treatment with Neuromuscular blocker was significantly associated with prolonged weaning (OR = 2.5; 95% IC [1.02–6.3], p = 0.04).

**Conclusion:** In this study, the weaning rate from IMV among Critically ill patients with COVID-19 pneumonia was 20%. A longer duration of neuromuscular blocker infusion was a risk factor for prolonged weaning from IMV.

**Compliance with ethics regulations:** Yes in clinical research.

## FC-273 Sleep continuity correlates with patients-reported sleep quality in conscious critically ill patients

### Eloïse Van Camp^1,2^, Christophe Rault^1^, Quentin Heraud^1^, Jean-Pierre Frat^1^, Arnaud W Thille^1^, Delphine Chatellier^1^, Anne Veinstein^1^, Remi Coudroy^1^, Pierre Olivier Fernagut^2^, Xavier Drouot^1,2^

#### ^1^CHU de Poitiers, Poitiers, France; ^2^INSERM 1084 Equipe 3, Poitiers, France

##### **Correspondence:** Xavier Drouot (xdrouot@yahoo.fr)

*Annals of Intensive Care* 2023, **13(Suppl 1):**FC-0273

**Rationale:** It is now well established that the sleep quality of critically ill patients is poor. Although sleep quantity might be normal, sleep is highly fragmented by arousals and brief awakenings. These sleep disruptions have been associated with prolonged weaning duration. Quantifying sleep in ICU patients is challenging. Polysomnography (PSG) offers the advantages to measure sleep continuity, a recent parameter which has been associated with outcome in patients treated with non-invasive ventilation (1). However, performing PSG in the critically ill is very challenging, requiring dedicated sleep technicians and sleep experts familiar with specific sleep scoring rules. Then, we elaborated an automated sleep scoring algorithm measuring sleep continuity by processing one single EEG channel. Richards-Campbell sleep questionnaire (RCSQ) is a validated questionnaire to assess sleep quality in ICU patients. However, RCSQ is difficult to administrate in patients who are not fully awake, and a simpler numeric visual analog scale has been proposed as an alternative. We investigated the relationship between sleep continuity measured with our automated scoring algorithm and patient-reported sleep quality.

**Patients and methods/materials and methods:** We analyzed retrospectively 52 polysomnography previously recorded in non-sedated and conscious ICU patients. Patients sleep was recorded the night after extubation. For each patient, one EEG channel (C3-A1) was processed, providing automated sleep hypnograms. Sleep continuity was measured using this automated scoring as previously described. Patient-reported sleep quality was assessed using Richards Campbell Sleep Questionnaire (score 0 to 100) and by using a visual analog scale, graduated from 0 “very poor sleep quality” to 100 “excellent sleep quality”. We searched for a correlation between patient-reported sleep quality and sleep continuity.

**Results:** Sleep continuity could be calculated on 29 PSGs, (age: 68y [58–77], median [25th–75th]). Richards-Campbell sleep questionnaire (390 [303–440]) and visual analog scale (5.5 [4.0–7.0]) have been obtained in 18 patients and 29 patients, respectively. Among the 29 patients, 5 (17%) had a positive delirium scale. Our results show a significant correlation between automated sleep continuity and visual analog scale (p < 0.008, Rho = 0.46; n = 29; Spearman correlation test). In contrast, no correlation existed between sleep continuity and RCSQ score (p = 0.66, n = 18; Spearman correlation test).

**Conclusion:** Self-reported sleep quality using a simple visual analog scale is easily administered in ICU patients. Sleep continuity measured by an automated algorithm may capture a large part of sleep perception and might be a mean to quantify sleep quality.

**Reference 1**

Drouot et coll. Sleep continuity: a new metric to quantify disrupted hypnograms in non-sedated intensive care unit patients, Crit Care 2014 18(6):628.

**Compliance with ethics regulations:** Yes in clinical research.

## FC-274 A real-time automated sleep scoring algorithm to detect refreshing sleep in conscious ventilated critically ill patients

### Christophe Rault^1^, Quentin Heraud^1^, Jean-Pierre Frat^1^, Arnaud Thille^1^, Remi Coudroy^1^, Xavier Drouot^1^

#### ^1^CHU de Poitiers, Poitiers, France

##### **Correspondence:** Xavier Drouot (xdrouot@yahoo.fr)

*Annals of Intensive Care* 2023, **13(Suppl 1):**FC-0274

**Rationale:** Due to the noisy environment, disease severity, treatments and anxiety, a vast number of patients admitted to intensive care units (ICUs) suffer from severe sleep disruptions. These sleep alterations have been associated with a prolonged need for assisted ventilation, a long ICU stay or even death. Currently, no treatments are available to improve sleep in ICU. Sleep studies and sleep scoring in the critically ill are very challenging and require sleep experts familiar with specific sleep scoring rules, limiting such studies to a few experienced teams. In this context, an automated scoring system would be of interest for researchers and clinicians. In addition, an algorithm operating in real-time, providing an instantaneous sleep scoring could be used by nurses to preserve patients’ sleep. We elaborated a sleep scoring algorithm operating in real time. Knowing that refreshing sleep episodes last more than 10 min, we devised an algorithm with a high sensitivity for such sleep episodes. To validate the algorithm, we compared the automated scoring against visual human scoring.

**Patients and methods/materials and methods:** We analyzed retrospectively 45 polysomnographies (PSG) previously recorded in non-sedated and conscious ICU patients. PSG were performed the night after the first failed spontaneous breathing trial. For each patient, one EEG channel (C3-A1) was processed, providing automated sleep hypnograms. We compared total sleep time obtained with visual scoring and with automated scoring. The proportion of sleep episodes lasting more than 10 min and correctly identified by the algorithm was calculated for each patient. Sensitivity and specificity of the algorithm to detect sleep episode lasting more than 10 min were determined.

**Results:** Thirty-six PSGs were then processed. Fifteen patients (41%) displayed atypical sleep on visual scoring. Automated total sleep time and visual total sleep time were significantly correlated (Rho = 0.48, p = 0.005, Spearman correlation test); the automatic system overestimated total sleep time. The median [25th–75th] percentage of sleep episodes lasting more than 10 min correctly detected by the algorithm was 100% [73.2–100.0]. Median sensitivity was 97.9% [92.5–99.9] and median specificity was 49.7% [26.1–73.5].

**Conclusion:** An automated sleep scoring system can identify nearly all long sleep episodes. Since these episodes are restorative, this real-time automated system opens the way for EEG guided—sleep protection strategies. Nurses could cluster their non-urgent care procedures and reduce ambient noise to minimize patients’ sleep disruptions. This innovative real-time sleep scoring algorithm could be a very useful tool to help implement sleep bundles and to counteract sleep alterations in ICUs.

**Compliance with ethics regulations:** Yes in clinical research.

## FC-275 Assessment of diaphragmatic function 18 months after discharge from intensive care in COVID-19 patients

### Salma Ben Othman^1^, Fatma Essafi^1^, Khaoula Ben Ismail^1^, Imen Talik^1^, Najla Ben Slimene^1^, Moez Kaddour^1^, Takoua Merhaben^1^

#### ^1^Hôpital régional de Zaghouan, Zaghouan, Tunisie

##### **Correspondence:** Salma Ben Othman (salma.sbo97@gmail.com)

*Annals of Intensive Care* 2023, **13(Suppl 1):**FC-0275

**Rationale:** Many survivors from severe coronavirus disease 2019 (COVID-19) suffer from persistent dyspnea. While these symptoms may stem from direct damage of the lung parenchyma itself, the possibility of underlying neuromuscular respiratory weakness should be considered. Recently, an assessment of diaphragmatic function has been described after recovery from COVID-19 in critically ill patients. Nevertheless, frequency and severity of diaphragm dysfunction remains unknown. Aim:to assess diaphragm function in COVID-19 survivors to detect diaphragm impairment.

**Patients and methods/materials and methods:** A prospective study was carried out on consenting COVID-19 survivors after 18 months discharge from ICU Zaghouan’s regional hospital, TUNISIA. The ultrasonographic examinations were performed by the same operator using a commercially available ultrasound machine (Vivid T8). The contractility of the right hemi-diaphragm was assessed by measuring the diaphragmatic excursion (DE) in quiet and deep breathing, inspiratory time (IT) in quite breathing and thickening fraction (TF) in quiet breathing. The measurements were made in a sitting position and were averaged from at least three different respiratory cycles. Subsequently the measurements were analyzed in comparison with recently published data in a healthy population according to gender [1, 2].

**Results:** Overall 42 patients were included, the median age was 50 years [28–81]. The gender ratio was 1.6. Twenty patients had comorbidities. Their mean body mass index was 30 kg/m^2^. All patients required mechanical ventilation; it was invasive in 4 patients. Initially, Lung extension abnormalities in chest CT were 60 ± 20.5%. The length of ICU stay was 9 ± 6 days. At 3 months follow-up new breathlessness was the most common reported symptom (21/42). We reported an oxygen desaturation during the 6-min walk test in five patients. CT control revealed abnormalities in 27 cases. At 18 months after ICU discharge, persistent respiratory difficulties were recorded in 20 patients. Median DE during quiet breathing was 2 [1.06–4.19] and during deep breathing was 5.9 [2.9–8.3]. Median IT was 0.98 [0.5–1.7]. Median TF was 28% [12.6–42.5]. The ultrasound examination detected 6 cases of diaphragm dysfunction corresponding to 2 patients with abnormal DE in deep breathing and 4 patients with abnormal TF. There was no significant difference by comparing those who experience persistent dyspnea, and others in diaphragmatic excursions (p = 0.33; p = 0.41) and inspiratory time (p = 0.74) on the other side there was a significant difference in the thickening fraction (p = 0.02).

**Conclusion:** Diaphragm dysfunction can be present 18 months after severe COVID-19 and may be related to persistent dyspnea. Larger studies are needed to evaluate itsclinical impact.

**Reference 1**

[1] Alain Boussuges. Diaphragmatic motion recorded by M-mode ultrasonography: limits of normality. ERJ. 2021.

**Reference 2**

[2] Alain Boussuges. Ultrasound assessment of diaphragm thickness and thickening: reference values and limits of normality when in a seated position. Frontiers in Medicine.2021.

**Compliance with ethics regulations:** Yes in clinical research.

## FC-276 Accuracy of SpO_2_ in patients with ARDS

### Imen Klai^1^, Hamdi Doghri ^1^, Ines Sdiri^1^, Ines Sedghiani^1^, Nebiha Borsali-Falfoul^1^

#### ^1^Hôpital Habib Thameur, Tunis, Tunisie

##### **Correspondence:** Imen Klai (Klaiimen@outlook.fr)

*Annals of Intensive Care* 2023, **13(Suppl 1):**FC-0276

**Rationale:** Critically ill patients hospitalized for ARDS requires close monitoring of blood oxygen saturation. Although the measurement of oxygen saturation in the arterial blood (SaO_2_) is the gold standard, this invasive technique requires blood sampling by arterial puncture. Pulse oximetry is a monitoring tool used to clinically assess peripheral oxygen saturation (SpO_2_) and to guide therapeutic interventions. It is a non-invasive, simple and relatively available measurement. This technique is widely used, particularly in intensive care units. The aim is to evaluate the accuracy of pulse oximetry (SpO_2_) in patients with ARDS.

**Patients and methods/materials and methods:** This is a monocentric, evaluative, prospective and comparative study including patients admitted in an ICU department from December 12, 2022 to February 09, 2023 with ARDS at the time of the study. Demographic, clinical, therapeutic and biological data were collected. Pulse oxygen saturation measurement was performed in all patients at the level of the middle finger of the right hand. The measurement of pulse oxygen saturation was made by a pulse oximeter of the monitor. The measurement was followed immediately by the realization of an arterial blood gas sample (by an arterial puncture or by a blood sample from the arterial catheter). DeltaSpO_2_ (SpO_2_–SaO_2_) was calculated.

**Results:** A total of 102 arterial blood gases (ABG’s) were obtained from 20 patients with ARDS. Sex ratio was 1.37. The median APACHE II, SAPSII and SOFA score were respectively 16 [9–22], 33 [23–55], and 4 [2–6]. 16 ABG’s (15.7%) were collected by arterial catheter and 85 (83.3%) by arterial punction. An arrythmia was present during 27 measurements (26.5%). The median temperature, respiratory rate and heart rate were respectively 37.4 [36.7–38.3], 22 [19–28] and 103 [90–115] The median of vaso-active drug dose and oxygen inspiratory fraction were respectively 3 mg/h [1–4] and 0.4 [0.35–0.6]. The analysis of ABG’s showed that the median pH, PaO_2_, PaCO_2_, bicarbonate, SaO_2_ and PaO_2_/FiO_2_ ratio were respectively 7.39 [7.27–7.46], 75 [62–92], 45 [38–62], 28 [23–33], 94% [91–97], 96 [93–98] and 157 [120–195]. Delta SpO_2_ (SpO_2_–SaO_2_) had a median of 1% [− 1–4]. Thus, delta SpO_2_ > 4% was found in 18 assays. In multivariate analysis, PaO_2_ < 68 mmHg was an independent risk factor for overestimating SpO_2_ (delta SpO_2_ > 4) with p = 0.014 (OR = 40 CI 95% [2.11–780]).

**Conclusion:** SpO_2_ may be overestimated in patients with ARDS. In our study, a cut off vaule of PaO_2_ < 68 mmHg was associated with an overestimation upper than 4%.

**Compliance with ethics regulations:** Yes in clinical research.

## FC-277 Accuracy of pulse oxygen saturation (SpO_2_) in patients with shock

### Imen Klai^1^, Hamdi Doghri^1^, Ines Sdiri^1^, Ines Sedghiani^1^, Nebiha Falfoul-Borsali^1^

#### ^1^Hôpital Habib Thameur, Tunis, Tunisie

##### **Correspondence:** Imen Klai (Klaiimen@outlook.fr)

*Annals of Intensive Care* 2023, **13(Suppl 1):**FC-0277

**Rationale:** Pulse oximetry (SpO_2_) is a monitoring tool, routinely used for critically ill patients to assess peripheral oxygen saturation and to guide therapeutic interventions. Shock is associated with microcirculatory abnormalities that lead to a decrease in oxygen delivery and tissue hypoxia which could affect pulse oximeter accuracy. The aim is to evaluate the accuracy of pulse oxygen saturation (SpO_2_) in patients with septic shock.

**Patients and methods/materials and methods:** Descriptive, prospective and monocentric study including patients admitted in an ICU department between 12/12/2022 and 07/02/2023 with septic shock. Demographic, clinical, therapeutic and biological data were recorded. We calculated the difference between (SaO_2_) and (SpO_2_), DeltaSpo_2_ (SpO_2_–SaO_2_).

**Results:** 40 ABGs were realized in eight patients with septic shock. APACHE II, IGS II and SOFA score had a median respective value 16 [9–22], 33 [23–55] and 4 [2–6]. An arrythmia was present at the time of sampling in 24 ABGs (60%) The median temperature and respiratory rate were respectively 37 [36–38.4] and 20 [15–24]. All patients were under vasoactive drugs with a median dose of 3 mg/h [1–4], The median heart rate, systolic arterial pressure and diastolic arterial pressure were respectively 60 bpm [54–68], 106 mmHg [92–127] and 60 mmHg [54–68]. The median pH, PaO_2_, PaCO_2_, Bicarbonate, SaO_2_, and SpO_2_ were respectively 26 [7.18–7.32], 108 [78–161], 47 [37–58], 20 [17–25], 98% [93–99] and 96 [94–97]. Median PaO_2_/FiO_2_ ratio and FiO_2_ were respectively 210 [135–300] and 0.4 [0.35–0.6]. Delta SpO_2_ median was − 2% [− 4–2]. PaO_2_ < 68 mmHg was an independent risk factor predictive of a DeltaSpO_2_ > − 2% P = 0.003, OR = 37.5, IC = 95%, [3.44–408].

**Conclusion:** Pulse oximetry underestimates ABG determined SaO_2_ in patients with septic shock. Arterial blood gas analysis is advised when a high level of precision in SaO_2_ determination is required especially for hypoxemic patients.

**Compliance with ethics regulations:** Yes in clinical research.

## FC-278 Comparison of two oxygenation SpO_2_ targets with two different oximeters—impact on oxygen flow rates and on oxygenation parameters

### François Lellouche^1^, Pierre-Alexandre Bouchard^1^

#### ^1^Institut Universitaire de Cardiologie et de Pneumologie de Québec, Québec, Canada

##### **Correspondence:** François Lellouche (francois.lellouche@criucpq.ulaval.ca)

*Annals of Intensive Care* 2023, **13(Suppl 1):**FC-0278

**Rationale:** SpO_2_ target influences oxygen utilization (1). It was recently shown that oximeter brand influenced the SpO_2_ measurements (2). The objective of the study is to evaluate the impact of the combination of SpO_2_ target and oximeter brand on oxygen utilization, occult hypoxemia and occult hyperoxemia.

**Patients and methods/materials and methods:** We currently conduct a randomized cross-over study in stable ICU patients requiring oxygen therapy delivered through nasal canula after cardiac surgery. Patients without adequate SpO_2_ signal are excluded. Four randomized periods of 10 min are conducted in all patients with different SpO_2_ targets (90 and 94%) and different oximeters (Nonin and Philips). For each period we collect the oxygen flow and oxygen blood gases at the end of the period. We compare the oxygen flow, the rate of occult hypoxemia (SaO_2_ < 90% with SpO_2_ ≥ 90%) and occult hyperoxemia (SaO_2_ > 96% with SpO_2_ ≤ 96%), oxygen partial weaning (< 0.5 L/min) or complete weaning and the rate of high O_2_ flow requirements (> 5 L/min).

**Results:** We present preliminary data based on first 12 patients (mean age 68 ± 9 years, 100% were men with light skin pigmentation, none had shock). At baseline, SpO_2_ was 93.3 ± 1.5% and oxygen flow was 2.5 ± 1.6 L/min. Main results for the oxygen flow and oxygenation parameters in the different study periods are displayed in the Figure. Differences in mean oxygen flow during Nonin 90 (2.0 ± 2.2 L/min) and Philips 94 (2.5 ± 2.3 L/min) are not statistically different (P = 0.11). However, all other comparisons of the flow are statistically different. The rate of complete weaning was 50% in the Philips 90 period and 0% in other periods. Oxygenation parameters (SaO_2_, PaO_2_) were similar during Nonin 90 (94 ± 1%, 72 ± 5 mmHg) and Philips 94% (94 ± 1%, 72 ± 8 mmHg). Conversely, there were statistically different oxygenation levels with Nonin 94 (97 ± 1%, 91 ± 7 mmHg) and Philips 90 (91 ± 1%, 62 ± 5 mmHg). The rate of occult hypoxemia and hyperoxemia were 4/12 and 0/12 in the Philips 90 period and 0/12, 10/12 in the Nonin 94 period.

**Conclusion:** In patients requiring conventional oxygen therapy, the SpO_2_ target, the oximeter brand and even more the combination of both had major impact on oxygen utilization, oxygen weaning and both occult hypoxemia and hyperoxemia. Patients managed with Nonin 90 and Philips 94 had similar oxygen flow and similar arterial oxygenation parameters. These data underline the necessity to use corrected SpO_2_ targets rather than universal SpO_2_ targets to manage oxygen therapy.

**Reference 1**

Bourassa S, et al. Oxygen conservation methods with automated titration. Respiratory care 2020; 65(10):1433–42.

**Reference 2**

Blanchet MA, et al. Accuracy of multiple pulse oximeter brands in stable critically ill patients—Oxygap study. Respiratory care 2023.

**Compliance with ethics regulations:** Yes in clinical research.

**Figure 1 (abstract FC-278):**
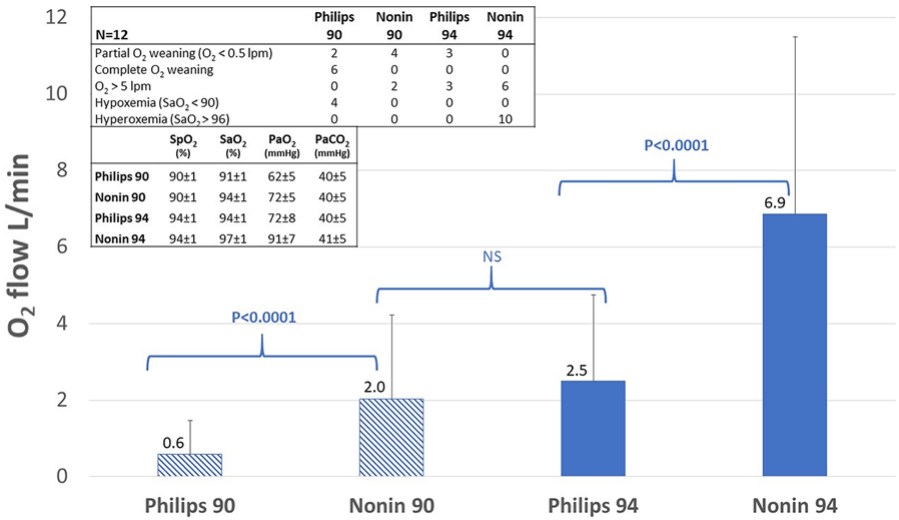
Comparison of oxygen flow utilization in different study conditions: Philips 90, Nonin 90, Philips 94, and Nonin 94) and ratio of oxygen utilization for the different tested conditions

## FC-279 Do SpO_2_ discrepancies related to oximeter’s brand and skin pigmentation cause ARDS misclassification with SpO_2_/FiO_2_?

### Antoine Delobel^1^, Marie-Anne Blanchet^1^, Pierre-Alexandre Bouchard^1^, François Lellouche^1^

#### ^1^Institut Universitaire de Cardiologie et de Pneumologie de Québec, Québec, Canada

##### **Correspondence:** François Lellouche (francois.lellouche@criucpq.ulaval.ca)

*Annals of Intensive Care* 2023, **13(Suppl 1):**FC-0279

**Rationale:** SpO_2_/FiO_2_ may be used for ARDS classification (1). It is known that skin pigmentation may induce bias in SpO_2_–SaO_2_ bias, and it was recently shown that oximeter brand influences the SpO_2_ measurements (2). The objectives of this study are to evaluate the impact of the SpO_2_ bias on ARDS misclassification when using SpO_2_/FiO_2_.

**Patients and methods/materials and methods:** We performed a retrospective analysis of the OXYGAP study (2) using 211 pairs of SpO_2_ values with Nonin-OEM and Philips-FAST pulse oximeters and related PaO_2_. We compared the classification provided by PaO_2_/FiO_2_ and SpO_2_/FiO_2_ with Nonin and Philips. The usual P/F thresholds were used (300–200–100) and thresholds based on Rice's equation were used for S/F (315–235–150). A theoretical set of SpO_2_/FiO_2_ values were obtained with SaO_2_ values going from 88 to 96% and FiO_2_ going from 25 to 80% with steps of 5%, for Nonin and Philips oximeters (SpO_2_ bias of − 3 and + 1%), and for light and dark skinned (SpO_2_ bias 0 and + 3%). ARDS classifications were compared with the different situations.

**Results:** FiO_2_ were available in patients on invasive mechanical ventilation (n = 22), high flow nasal canula (n = 15), in patients without respiratory support (n = 84, FiO_2_ = 21%), or in patients under conventional oxygen therapy (estimated FiO_2_ based on oxygen flow, n = 83). SpO_2_ values were missing for one of the evaluated oximeters in 7 patients. Classification of ARDS based on the different pulse oximeters did not differ much (differences below 3% between groups, Figure), however there were differences in comparison with PaO_2_/estimated FiO_2_ and SpO_2_/FiO_2_ classifications. In the theoretical set of values, 108 SpO_2_/FiO_2_ values were obtained for several conditions (Nonin and Philips oximeters, light and dark skin). ARDS misclassifications were below 5% for Nonin vs. Philips and light vs. dark skin pigmentation. With Nonin and Philips oximeters the classification as No, mild, moderate and severe ARDS were 8, 17, 37, 38% and 11, 17, 38, 34% respectively. With light and dark skin pigmentation, the classification as No, mild, moderate and severe ARDS were 10, 17, 37, 36% and 13, 17, 39, 31% respectively.

**Conclusion:** Utilization of SpO_2_/FiO_2_ to classify the severity of ARDS is feasible with low number of misclassifications when comparing SpO_2_/FiO_2_ with different pulse oximeters or with discrepancies related to skin pigmentation. In the cohort of patients, classifications differed when comparing PaO_2_/estimated FiO_2_ and SpO_2_/estimated FiO_2_ for No ARDS and mild ARDS categories.

**Reference 1**

Wick KD, Matthay MA, Ware LB. Pulse oximetry for the diagnosis and management of acute respiratory distress syndrome. Lancet Respir Med 2022; 10(11): 1086–98.

**Reference 2**

Blanchet MA, et al. Accuracy of multiple pulse oximeter brands in stable critically ill patients—Oxygap study. Respiratory care 2023.

**Compliance with ethics regulations:** Yes in clinical research.

**Figure 1 (abstract FC-279):**
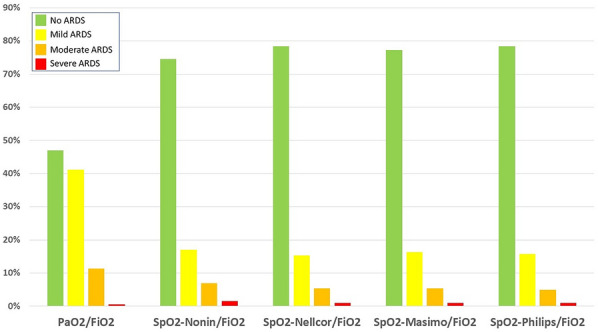
ARDS classification based on PaO_2_/estimated FiO_2_ or SpO_2_/estimated FiO_2_ with different pulse oximeters (Nonin, Nellcor, Masimo, Philips) in the secondary analysis of the Oxygap study and 211 pairs of SpO_2_ with four pulse oximeters and PaO_2_

## FC-280 Utilization of corrected SpO_2_ or corrected SpO_2_ target to homogenize the practices regarding management of oxygen therapy

### François Lellouche^1^, Antoine Delobel^1^, Marie-Anne Blanchet^1^, Pierre-Alexandre Bouchard^1^

#### ^1^Institut Universitaire de Cardiologie et de Pneumologie de Québec, Québec, Canada

##### **Correspondence:** François Lellouche (francois.lellouche@criucpq.ulaval.ca)

*Annals of Intensive Care* 2023, **13(Suppl 1):**FC-0280

**Rationale:** SpO_2_ target influences oxygen utilization with increased by 3 folds the oxygen flow with 4% difference in SpO_2_ target1. It was recently shown that oximeter brand influenced the SpO_2_ measurements2. Combination of the errors on the SpO_2_ measurement related to the oximeter brand and choice of different target may have major impact on the clinical management of patients in the clinical practice and during research. One solution may be to use a corrected SpO_2_ target rather than an universal SpO_2_ target to homogenize the practices. The other solution is to correct the SpO_2_ values based on the known SpO_2_–SaO_2_ biases. The objective of the study was to evaluate if the utilization of a corrected SpO_2_ target or of corrected SpO_2_ values could homogenize the O_2_ management.

**Patients and methods/materials and methods:** We performed a secondary analysis of the Oxygap study to evaluate the oxygen utilization (Increase, Decrease or No change of O_2_ support) in the 211 included patients. The oxygen flow was modified based on individual SpO_2_ values to maintain (i) a non-corrected SpO_2_ target of 92–96%, using the Nonin, Nellcor, Masimo, Philips and (ii) a corrected SpO_2_ target with same pulse oximeters. The correcting factors were based on the SpO_2_–SaO_2_ bias for each oximeters found in the Oxygap study2, respectively − 3.1, − 0.3, − 0.2 and + 0.9%. We also evaluated the impact of the utilization of corrected SpO_2_ values, based on the known SpO_2_–SaO_2_ bias.

**Results:** Based on the values obtained in the Oxygap study with 211 patients, the oxygen management showed significant discrepancies when SpO_2_ were not corrected or when SpO_2_ target was not corrected (Figure, panel A). When SpO_2_ values were corrected (Figure, panel B) or when SpO_2_ target were corrected (Figure, panel C), the management of oxygen support was homogenized with reduction of discrepancies.

**Conclusion:** The utilization of corrected SpO_2_ values or corrected SpO_2_ target may allow to reduce the large discrepancies in O_2_ utilization based on confounding factors related to pulse oximetry measurements. Additional corrections based on skin pigmentation will be required when bias is adequately evaluated.

**Reference 1**

Bourassa S, et al. Oxygen Conservation Methods With Automated Titration. Respiratory care 2020; 65(10): 1433–42.

**Reference 2**

Blanchet MA, et al. Accuracy of multiple pulse oximeter brands in stable critically ill patients—Oxygap study. Respiratory care 2023.

**Compliance with ethics regulations:** Yes in clinical research.

**Figure 1 (abstract FC-280):**
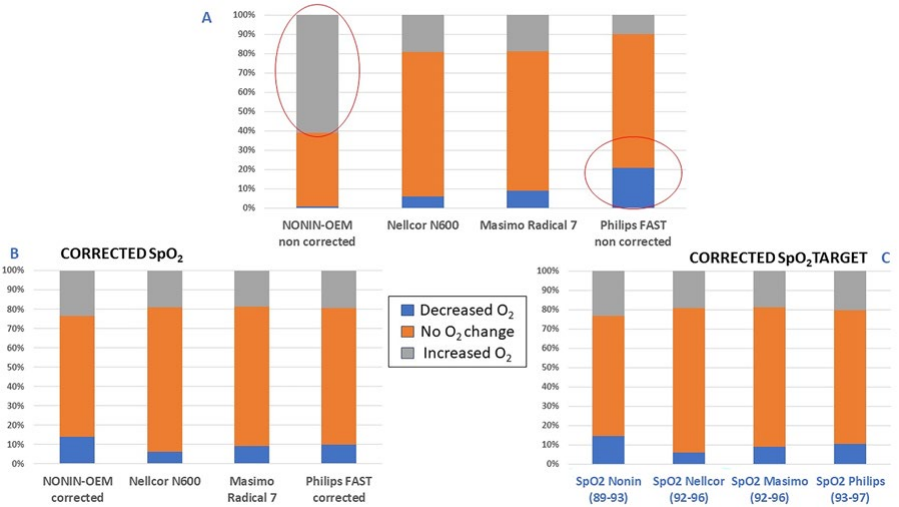
Management of oxygen support in the cohort of 211 patients of the OXYGAP study to maintain SpO_2_ within 92–96% with uncorrected SpO_2_ (Panel A), with corrected SpO_2_ (Panel B) or with corrected SpO_2_ target (Panel C)

## FC-281 SpO_2_/FiO_2_ ratio as an assessment tool for hypoxemia evaluation in intensive care department

### Ines Sdiri^1^, Hamdi Hamdene Doghri^1^, Imen Klai^1^, Ines Sedghiani^1^, Nebiha Falfoul Borsali^1^

#### ^1^Hôpital habib thameur, Tunis, Tunisie

##### **Correspondence:** Ines Sdiri (sdiri.ynes@gmail.com)

*Annals of Intensive Care* 2023, **13(Suppl 1):**FC-0281

**Rationale:** Criteria for acute respiratory distress syndrome (ARDS) is based on PaO_2_/FiO_2_ ratio measured by arterial blood gas analysis. This definition is used to assess the degree of hypoxemia and its severity. However, the pulse oximetric saturation offers a continuous, non-invasive hypoxemia evaluation. We hypothesized that the SpO_2_/FiO_2_ ratio can be substituted for the PaO_2_/FiO_2_ ratio in assessing the oxygenation criteria. We aim in this study to compare the reliability of SpO_2_/FiO_2_ ratio and PaO_2_/FiO_2_ ratio to assess and classify hypoxemia.

**Patients and methods/materials and methods:** A prospective cohort study of adults with ARDS confirmed based on Berlin classification admitted to the intensive care unit (ICU) in the Teaching hospital of Habib Thameur from 1st October 2022 to 1st February 2023 were performed. Demographic, clinical, therapeutic and biological data were collected. We analysis the correlation between the two ratios. Linear regression modeling was used to quantify the relationship between indices. Receiver operating characteristic (ROC) curves were used to determine the sensitivity and specificity of the threshold values.

**Results:** 206 assays for 57 patients were included. The sex ratio was 1.19. The age, APACHE II and SAPS II score had a median value respectively 68, 13 and 9. Among these assays, 103 (49.5%) and 40 (19.4%) were respectively realized in patients with ARDS and with shock. The PaO_2_, SpO_2_, PaO_2_/FiO_2_ and SPO_2_/FiO_2_ were respectively in median: 96 [93–98], 96 [94–98], 196 [140–280] and 220 [166–274]. Our analysis concluded that SpO_2_/FiO_2_ was correlated with PaO_2_/FiO_2_ (spearman’s rho = 0.583 and p < 10^−3^) (Figure n1). This correlation was stronger for moderate and severe ARDS (ROC curves no. 1, 2 and 3).

**Conclusion:** SpO_2_/FiO_2_ ratio was correlated with PaO_2_/FiO_2_ ratio. SpO_2_/FiO_2_ ratio may be a useful tool for an early real-time rapid assessment of hypoxemia and identification of ARDS, while decreasing the cost, blood loss, pain, skin breaks, and vascular punctures associated with serial arterial blood gas measurements.

**Compliance with ethics regulations:** Yes in clinical research.

## FC-282 Mortality and prognostic factors related to hyperoxia in ICU

### Badreddine Aouji^1^, Ibtihal Sebbata^1^, Mohammed Abidi^1^, Rehab Haffar^1^, Firdaous Belkaid^1^, Kawtar Ziati^1^, Oussama Ssouni^1^, Latifa Oualili^1^, Khalid Abidi^1^, Tarek Dendane^1^, Amine Ali Zeggwagh^1^

#### ^1^CHU Ibn Sina, Rabat, Maroc

##### **Correspondence:** Badreddine Aouji (aoujiaouji@gmail.com)

*Annals of Intensive Care* 2023, **13(Suppl 1):**FC-0282

**Rationale:** This is a retro-prospective analytical cohort study conducted in the Medical ICU Department between March 2021 and November 2022.

**Patients and methods/materials and methods:** All patients aged 18 years and older (n = 226) and with at least one arterial blood gas sampling during their hospitalization were included. Multiple variables were gathered and analyzed using the software called Jamovi 2.3.21. The determination of predictive and prognostic factors of hyperoxia (PaO_2_ ≥ 120 mmHg) was obtained by comparing the group of hyperoxic patients (PaO_2_ ≥ 120, n = 100), to the group of normoxic patients (70 ≤ PaO_2_ < 120, n = 101) and to hypoxic patients (70 < PaO_2_, n = 25).

**Results:** The hyperoxic patient group had an average age of 48.9 ± 18.8 years, with a sex ratio of 1.04. Diabetic patients were 28.3% and those with chronic lung disease (asthma, DBD, COPD) were 20%. The mean PaO_2_ at admission was 176 ± 106 mmHg in the hyperoxic group, compared with 88.7 ± 28.1 mmHg in the normoxic group and 67.7 ± 10.3 in the hypoxic group. Upon admission, hyperoxic patients had an average SOFA score of 4.65. Mechanical ventilation was required in 59% of hyperoxic patients whereas it was required in 38% of the normoxic patients, and in 25% of the hypoxic patients. Nosocomial infection (OR = 1.982; 95% CI [p = 0.036]) and length of stay (β = 2.79; [p = 0.038]) were risk factors for hyperoxia. As for the predictive factors, hyperoxia was significantly related to only one parameter, which was asthma (OR = 4.22; 95% CI [p = 0.036]). The mortality rate was 53% in the hyperoxic group, compared with 47% in the normoxic group and 52% in the hypoxic group. The factors influencing mortality were: mechanical ventilation (OR = 8.90; IC95% [p < 0.001]), nosocomial infection (OR = 4.60; IC95% [p < 0.001]). Hyperoxia had no effect on mortality.

**Conclusion:** In conclusion, no correlation was found between hyperoxia and mortality. However, nosocomial infection and length of stay were prognostic factors for hyperoxia. While a wider sample population could have led to more convincing results, the outcome of this case study begs the question: what ideal PaO_2_ range objectively defines hyperoxia and normoxia?

**Compliance with ethics regulations:** Yes in clinical research.

## FC-283 Utilization of the smartphone application VentilO to reduce post-intubation respiratory acidosis

### Marie Dinh^1^, Pierre-Alexandre Bouchard^1^, François Lellouche^1^

#### ^1^Institut Universitaire de Cardiologie et de Pneumologie de Québec, Québec, Canada

##### **Correspondence:** François Lellouche (francois.lellouche@criucpq.ulaval.ca)

*Annals of Intensive Care* 2023, **13(Suppl 1):**FC-0283

**Rationale:** Protective mechanical ventilation is recommended for patients requiring mechanical ventilation. However, tidal volumes (TV) are frequently reduced with protocols, but respiratory rates (RR) are not increased proportionally, leading to frequent respiratory acidosis (1). While induced permissive hypercapnia has been promoted over the years, it is now recommended to avoid severe respiratory acidosis associated with pulmonary hypertension, right heart failure and mortality (2). In the literature, the frequency of respiratory acidosis (pH < 7.25 and PaCO_2_ > 45 mmHg) in mechanically ventilated patients is 25 to 55%. A smartphone application, VentilO, has been developed to optimize the implementation of protective mechanical ventilation. The objective of this study is to evaluate if this app may reduce respiratory acidosis after intubation.

**Patients and methods/materials and methods:** We will retrospectively review charts of 100 adult patients intubated at the emergency department or intensive care units. Main demographic data, reason for intubation will be recorded. Arterial blood gases after intubation will be recorded. TV and RR set by the clinicians will be compared to those recommended by the application. Local REB accepted this retrospective chart review. This application determines TV and RR, based on the patient’s gender and height, the predictive and actual body weight, the body temperature, the instrumental dead space and the type of patient (planned surgery, critically ill patient or intermediate). Our hypothesis is that the recommendations of VentilO will increase the minute ventilation (which would allow a reduction of respiratory acidosis) in at least 1/3 of the cases.

**Results:** Preliminary results are based on 14 first analyzed patient’s chart. Eight women and six men were included, mean age was 71 ± 14 years. The TV and RR after intubation were 444 ± 77 ml (7.7 ± 1.2 ml/kg PBW) and 16 ± 2 breaths/min. First arterial blood gases after intubation showed mean ± SD pH, PaCO_2_ and bicarbonates of 7.28 ± 0.10, 51 ± 11 and 23 ± 3 respectively. Acidosis (pH < 7.35) was present in 8 patients (57%), and PaCO_2_ > 45 mmHg in 10 patients (71%). In patients with hypercapnia, VentilO recommended to increase the RR in 60% and to keep it constant in 20% of them.

**Conclusion:** In these preliminary results, we found that acidosis and hypercapnia occurred in more than half of the patients after intubation in the ICU or in the ED. A smartphone application (VentilO) developed to facilitate the implementation of protective ventilation, may reduce the frequency of respiratory acidosis. Prospective studies should be conducted to improve the evaluation of such tools.

**Reference 1**

Fuller BM, Ferguson IT, Mohr NM, et al. A Quasi-experimental, before-after trial examining the impact of an emergency department mechanical ventilator protocol on clinical outcomes and lung-protective ventilation in acute respiratory distress syndrome. Cr.

**Reference 2**

Nin N, Muriel A, Penuelas O, et al. Severe hypercapnia and outcome of mechanically ventilated patients with moderate or severe acute respiratory distress syndrome. Intensive Care Med 2017; 43(2): 200–8.

**Compliance with ethics regulations:** Yes in clinical research.

## FC-284 Long terme outcome of adult patients with acute kidney injury in the intensive care unit at the Centre Hospitalier Universitaire de Libreville

### Laurence Essola^1^, Luc Bitegue Methe^1^, Arsène Ifoudji Makao^1^, Fernande Manga ^1^, Parfait Divassa Divassa^1^, Adrien Sima Zue^1^

#### ^1^Centre hospitalier universitaire de Libreville, Libreville, Gabon

##### **Correspondence:** Laurence Essola (laurenceessola@yahoo.fr)

*Annals of Intensive Care* 2023, **13(Suppl 1):**FC-0284

**Rationale:** Acute kidney injury (AKI) is a common complication that can deteriorate into chronicity in survivors. The main objective of this research was to evaluate the long-term outcome of patients who presented with AKI in the intensive care unit of the CHUL.

**Patients and methods/materials and methods:** This was a two-phase study: the first phase was retrospective consisted in collecting the medical records of patients suffering from AKI between January 2018 and June 2021 to identify the frequency of AKI, the main pathologies associated with it, and the patients' outcomes. The second was prospective: the frequency of CKD in patients surviving after AKI and the risk factors for the occurrence of CKD were studied.

**Results:** The frequency of AKI was 12.8%. 164 patients (85.9%) had AKI on admission (Group A) and 27 patients (14.1%) during hospitalization (Group B). The mean age was 50.4 ± 17.7 years old and the sex ratio was 1.2. In group A, septic shock and pneumopathy were the main pathologies associated with AKI. In the second phase, 60 of 103 surviving patients agreed to the study. Of these, 6 patients (10%): 3 were followed in nephrology and the other 3 were referred to a nephrologist. The predictive factors for the development of CKD were anuria and pneumopathy.

**Conclusion:** The discovery of CKD was fortuitous for some patients. This confirms the importance of a regular follow-up of patients with AKI in the ICU.

**Compliance with ethics regulations:** Yes in clinical research.

## FC-285 Duration of coma in two delayed renal replacement therapy initiation strategies in critically ill patients with severe acute kidney injury: a secondary analysis of the AKIKI 2 multicenter randomized controlled trial

### Thomas Rambaud^1,34^, David Hajage^1^, Romain Sonneville^2^, Said Lebbah^1^, Laurent Martin-Lefevre^4^, Guillaume Louis^3^, Sebastien Moschietto^5^, Dimitri Titeca-Beauport^6^, Beatrice La Combe^7^, Bertrand Pons^8^, Nicolas De Prost^9^, Sebastien Besset^10^, Alain Combes^1^, Adrien Robine^11^, Marion Beuzelin^12^, Julio Badie^13^, Guillaume Chevrel^14^, Julien Bohe^15^, Elisabeth Coupez^16^, Nicolas Chudeau^17^, Saber Barbar^18^, Christophe Vinsonneau^19^, Jean Marie Forel^20^, Didier Thevenin^21^, Eric Boulet^22^, Karim Lakhal^23^, Nadia Aissaoui^24^, Steven Grange^25^, Marc Leone^20^, Guillaume Lacave^26^, Saad Nseir^27^, Florent Poirson^34^, Julien Mayaux^1^, Karim Asehnoune^28^, Guillaume Geri^29^, Kada Klouche^30^, Guillaume Thiery^31^, Laurent Argaud^32^, Bertrand Rozec^28^, Cyril Cadoz^3^, Pascal Andreu^23^, Jean Reignier^28^, Jean-Damien Ricard^10^, Jean-Pierre Quenot^33^, Didier Dreyfuss^10^, Stephane Gaudry^34^

#### ^1^Pitié Salpêtrière - APHP, Paris, France; ^2^Hôpital Bichat AP-HP, Paris, France; ^3^CHR Metz-Thionville, Metz, France; ^4^CHR La Roche Sur Yon, La Roche Sur Yon, France; ^5^CHG Henri Duffaut Avignon, Avignon, France; ^6^CHU Amiens Picardie, Amiens, France; ^7^CH Bretagne Sud, Lorient, France; ^8^CHU Pointe à Pitre Abymes, Pointe à Pitre, France; ^9^Henri Mondor, Créteil, France; ^10^Louis Mourier, Colombes, France; ^11^CH Bourg En Bresse, Bourg En Bresse, France; ^12^CH Dieppe, Dieppe, France; ^13^CH Belfort, Belfort, France; ^14^CHSF, Corbeil-Essonnes, France; ^15^CH Lyon Sud, Lyon, France; ^16^Hôpital Montpied, Clermont-Ferrand, France; ^17^CH du Mans, Le Mans, France; ^18^Hôpital Caremeau, Nîmes, France; ^19^CH Béthune, Béthune, France; ^20^Hôpital Nord, Marseille, France; ^21^CH Dr Shaffner, Lens, France; ^22^CH Portes de L'oise, Beaumont Sur Oise, France; ^23^Hôpital Nord Laennec, Nantes, France; ^24^Hôpital Européen Georges Pompidou, Paris, France; ^25^CHU Rouen, Rouen, France; ^26^Hôpital André Mignot, Versailles, France; ^27^Roger Salengro, Lille, France; ^28^Hotel Dieu, Nantes, France; ^29^Ambroise Paré, Boulogne Billancourt, France; ^30^Lapeyronie, Montpellier, France; ^31^CHU Saint Etienne, Saint Priest En Jarez, France; ^32^Edouard Henriot, Lyon, France; ^33^Hôpital François Mitterrand, Dijon, France; ^34^Hôpital Avicenne, AP-HP, Bobigny, France

##### **Correspondence:** Thomas Rambaud (rambaud.t@gmail.com)

*Annals of Intensive Care* 2023, **13(Suppl 1):**FC-0285

**Rationale:** Acute encephalopathy is associated with worse outcomes in critically ill patients. Observational studies identified an independent relationship between acute kidney injury (AKI) and coma. The extent to which renal replacement therapy (RRT) can be postponed in severe AKI patients with acute encephalopathy remains unknown. We assessed the transition from coma to sustained awakening state according to two delayed RRT initiation strategies in comatose critically ill patients with severe AKI.

**Patients and methods/materials and methods:** The AKIKI2 (Artificial Kidney Initiation for Kidney Injury 2) study was a randomized controlled trial that compared two delayed RRT initiation strategies in 39 ICUs. In that study, patients with severe AKI (KDIGO3) and oliguria > 72 h or plasma urea concentration > 40 mmol/L (RRT initiation criteria in the delayed strategy of AKIKI1) were randomly assigned to either immediate RRT initiation (standard-delayed strategy) or to a more-delayed strategy. With the more-delayed strategy, RRT initiation was postponed until life-threatening complications or until plasma urea concentration ≥ 50 mmol/L. We conducted a secondary analysis on patients comatose (RASS < − 3) at the time of randomization to assess the safety of the more-delayed strategy. A multi-state model describing patient’s neurological status during 28 days was built, defining 5 mutually exclusive states: death, coma (RASS < − 3), incomplete awakening (RASS [− 3; − 2]), sustained awakening (RASS [− 1; + 1] two consecutive days) and agitation (RASS > + 1). Primary outcome was the association between randomization group and transition intensity from coma to sustained awakening state.

**Results:** Among 278 patients randomized in AKIKI2, 168 were comatose at the time of randomization. The SAPS III score was 74 [69–84] and 78 [66–85] and serum urea concentration 33 mmol/L and 37 mmol/L in the standard-delayed and more-delayed groups respectively. Sedative exposure did not differ between groups (number of days alive without sedative, 9 [5–13] vs 8 [5–15], p = 0.72). The transition intensity from coma to sustained awakening was lesser in the more-delayed RRT group (HR = 0.36 ([0.17–0.78], p = 0.010) (Figure). Similar results were observed in non-sedated patients (HR = 0.35 [0.14–0.87], p = 0.023). Time spent sustainedly awaken was 10.11 [8.11–12.15] and 7.63 [5.57–9.64] (p = 0.093) days in the standard-delayed and the more-delayed groups respectively. Time to weaning from mechanical ventilation did not differ between groups (14 vs 13.5 days, p = 0.65). The magnitude of increase of plasma urea level a given day was correlated with a lower probability of being sustainedly awaken the following day (OR = 0.983 for 1 mmol/L of increase [0.968–0.998], p = 0.018).

**Conclusion:** In comatose critically ill patients with severe AKI, a more-delayed RRT initiation strategy resulted in worse neurological outcomes. Neurological status should be considered among criteria for RRT initiation.

**Compliance with ethics regulations:** Yes in clinical research.

**Figure 1 (abstract FC-285):**
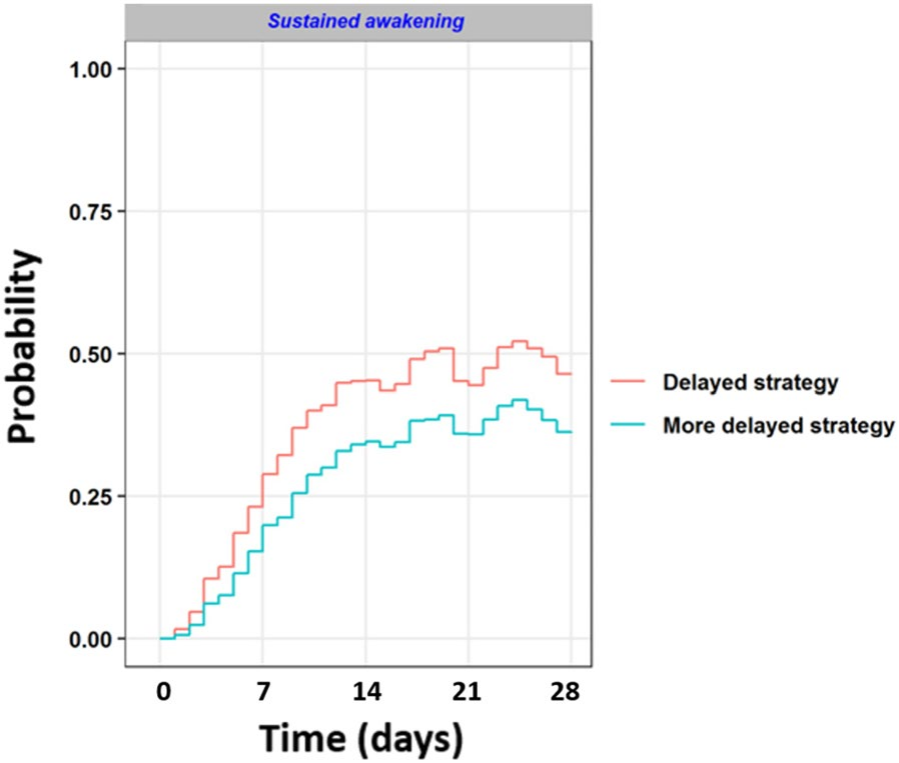
Probability of sustained awakening (RASS between − 1 and + 1 for 2 consecutive days) according to the RRT strategy

## FC-286 Urinary markers of dialysis initiation in intensive care unit at the diagnosis of acute kidney injury: a prospective study

### Faustine Vanackere^1^, Arthur Orieux^1^, Renaud Prevel^1^, Olivier Guisset^1^, Charline Sazio^1^, Didier Gruson^1^, Benjamin Clouzeau^1^, Fabrice Camou^1^, Sébastien Rubin^1^, Alexandre Boyer^1^

#### ^1^CHU de Bordeaux, Bordeaux, France

##### **Correspondence:** Arthur Orieux (arthur.orieux@chu-bordeaux.fr)

*Annals of Intensive Care* 2023, **13(Suppl 1):**FC-0286.

**Rationale:** Renal replacement therapy (RRT) is necessary for more than 10% of patients with acute kidney injury (AKI) in intensive care unit (ICU). A delayed initiation strategy for RRT is now recommended. Now, while respecting the strategy of delayed RRT initiation, the challenge may be to not delay the initiation of RRT in patients who will ultimately need it. Therefore, it would be interesting to detect earlier the future need for RRT. Simple urinary markers (urinary urea, natriuresis, fractional excretion of sodium or urea) may be relevant to assess the transient nature of the AKI. These markers have never been evaluated in an ICU population to predict the use of RRT. The main objective of this study is to assess the accuracy of these urinary markers to predict the use of RRT in ICU.

**Patients and methods/materials and methods:** We performed a prospective study including all patients admitted to ICU at the X University Hospital (two ICUs with 37 and 14 beds, respectively) who developed AKI between March and August 2022. We excluded patients with emergency RRT criteria, post-renal AKI, and chronic dialysis. RRT initiation criteria have been standardized according to the delayed strategy of the AKIKI study (Gaudry et al., 2016). The urinary assessment was carried out on D1, defined as the first day of AKI according to the KDIGO classification (serum creatinine and diuresis). A multivariate analysis was performed by logistic regression (RRT requirement).

**Results:** Six hundred sixty-three patients were admitted to ICUs. The incidence of AKI in ICU was 21% (142/663 patients), of whom 122 patients were included. The mean age was 65 ± 12 years, and most patients were men (68%). Median SAPSII was 58 [14–100]. AKI were severe (46% of KDIGO 3), and 22 out of 122 patients (18%) required RRT during their ICU stay. Mean urinary urea at D1 was 183 ± 12 mmol/L in the group without RRT compared to 107 ± 62 mmol/L in the RRT group (p = 0.01). In multivariate analysis, urinary urea on D1 (OR = 0.99 [0.98–0.99]) was significantly associated with the use of RRT.

**Conclusion:** A low urinary urea concentration at the AKI diagnosis was independently associated with the need for RRT in a strategy of delayed initiation. This marker should be further investigated to help identifying earlier in the follow-up the need for RRT of AKI patients in a delayed initiation strategy.

**Compliance with ethics regulations:** Yes in clinical research.

## FC-287 Biomarkers, EO-AKI and associated outcomes in COVID-19 patients, a prospective observational study

### Alice Ruault^1^, Carole Philipponnet^1^, Vincent Sapin^1^, Bertrand Evrard^1^, Bouzgarrou Radhia^1^, Laure Calvet^1^, François Thouy^1^, Kévin Grapin^1^, Benjamin Bonnet^1^, Mireille Adda^1^, Bertrand Souweine^1^, Claire Dupuis^1^

#### ^1^CHU Clermont Ferrand, Clermont-Ferrand, France

##### **Correspondence:** Claire Dupuis (cdupuis1@chu-clermontferrand.fr)

*Annals of Intensive Care* 2023, **13(Suppl 1):**FC-0287

**Rationale:** COVID-19 is characterized by inflammation and a pro coagulant state. Acute kidney injury (AKI) is the second organ failure in severe COVID-19. The aim of our study was to assess the association between inflammatory and nephrology biomarkers and Early-Onset-AKI (EO-AKI), no transient recovery of EO-AKI and day-90 mortality.

**Patients and methods/materials and methods:** It is a retrospective single center study. All consecutive adult patients aged ≥ 18 years admitted between March 2020 and February 2021 into the medical ICU of Clermont Ferrand, France, for SARS-CoV-2 pneumonia were enrolled. Patients with chronic kidney disease, referred from another ICU, and with an ICU length of stay (LOS) ≤ 72 h were excluded. EO-AKI was defined according to creatinine KDIGO, developing ≤ 7 days. EO-AKI was transient if recovery occurred within 48 h. Data collection: Biological testing on admission included plasma coagulation biomarkers (D-Dimers and fibrinogen levels), serum inflammatory biomarkers (CRP, ferritin, IL-1, IL-6, IL-10, PCT, mHLA-DR and Soluble receptor for advanced glycation end products (sRAGE). Nephrology biomarkers included the urinary cell cycle arrest biomarkers urinary tissue inhibitor of metalloproteinases-2 (TIMP-2)-insulin-like growth factor-binding protein 7 (IGFBP7) measured by NephroCheck Risk®, and Liver Fatty Acid-Binding Protein (LFABP). The association between those biomarkers and outcomes were assessed by ROC curve and binomial logistic regression or Cox survival model.

**Results:** 150 patients were included, with a median age of 71 years [64; 76], and SAPS II score of 35.6 [29; 44]. EO-AKI occurred in 30.7%. None of the inflammatory biomarkers, Nephrocheck, nor LFABP were predictive of EO-AKI. EO-AKI was classified as transient 43.5%. Nor LFABP, nor sRAGE, nor Nephrocheck were associated with no transient EO-AKI recovery. EO-AKI patients had lower survival at day-90 than patients without EO-AKI with 90-death rate of 63% vs 25% (p < 0.01). Il10, Il8 and sRAGE predicted the most death at day 90 with AUC of 0.71, 0.69 and 0.72 respectively. Sub AKI defined by no EO-AKI but positive Nephrocheck or LFABP were not associated with an increased risk of death. However, among patients without EO-AKI, patients with higher sRAGE had an increased risk of death (HR = 2.34 [1.0.5; 5.20], p = 0.03) and patients with EO-AKI and higher sRAGE were the most at risk of death (HR = 8.82 [4.03; 19.33], p < 0.01).

**Conclusion:** We only found that inflammatory biomarkers were predicted of day-90 mortality and patients with EO-AKI and higher sRAGE were the most at risk of death. Nephrocheck and LFABP had poor prognostic performances.

**Compliance with ethics regulations:** Yes in clinical research.

## FC-288 Inadvertent chloride loading during continuous veno-venous hemodialysis in critically ill patients: the CLODICUS study

### Matthieu Chivot^1^, Ian Baldwin^2^, Guillaume David^1^, Glenn Eastwood^2^, Jean-Christophe Richard^1^, Rinaldo Bellomo^2^, Laurent Bitker^1^

#### ^1^Service de Médecine Intensive – Réanimation, hôpital de la Croix Rousse, Hospices Civils de Lyon, Lyon, France; ^2^Department of Intensive Care, Austin Hospital, Melbourne, Australie

##### **Correspondence:** Matthieu Chivot (matthieu.chivot@gmail.com)

*Annals of Intensive Care* 2023, **13(Suppl 1):**FC-0288

**Rationale:** Inadvertent sodium flux during continuous renal replacement therapy (CRRT) in the intensive care unit (ICU) is a documented phenomenon [1]. We hypothesized that inadvertent chloride loading also occurs during CRRT, which may differ based on modality. We aimed to determine chloride mass transfer during CRRT performed with two different modalities.

**Patients and methods/materials and methods:** We performed a two-center, prospective, observational study in France and Australia in ICU patients with acute kidney injury treated with CRRT initiated within the 24 preceding hours. Patients received continuous veno-venous haemofiltration (CVVH), or continuous veno-venous haemodialysis (CVVHD) with citrate and CaCl_2_ regional anticoagulation. Patients were studied over a 24 h period, during which chloride concentrations were measured every 4 h in the plasma and from the waste effluent. The primary outcome was hourly chloride mass transfer, calculated as the difference between clean dialysate or replacement fluid chloride mass, and waste effluent chloride mass, measured over the 24 h period, and expressed in mmol h^−1^ L^−1^ of consumed dialysate or ultrafiltrate. Secondary outcomes included CRRT modality impact on chloride clearance and arterial pH. Results are presented with median [interquartile range]. Measurements comparison between CVVHD and CVVH accounted for the repetition of observation periods in a given patient, using mixed effects regression models.

**Results:** Between February 2021 and August 2022, we enrolled 37 patients (age 64 [56–71] years, 67% male), totalizing 20 CVVHD sessions and 20 CVVH sessions. There was no significant between-group difference in effluent and net ultrafiltration flow rates, and plasma chloride concentrations at enrolment. Hourly chloride mass transfer over 24 h was positive during CVVHD, and negative in CVVH (0.4 [0.2–0.4] vs. − 0.1 [− 0.3–0.1] mmol h^−1^ L^−1^, respectively, p < 0.01). During CVVHD, total chloride mass transfer over 24 h was 217 [85–349] mmol, with 100% [92–100%] of the chloride mass subsequent to CaCl_2_ reinjection contributing to total chloride mass transfer. Also, chloride clearance was negative during CVVHD, and positive in CVVH (− 7 [− 9 to − 5] vs. 2 [1–5] ml min^−1^, respectively, p < 0.01). Plasma chloride concentration increase over 24 h was significantly higher during CVVHD, compared to CVVH (2 [0–2] vs. 1 [− 1–3] mmol L^−1^, p = 0.02), with no significant impact on arterial pH (absolute change: 0.00 [− 0.07–0.03] vs. 0.01 [− 0.06–0.05] mmol L^−1^, p = 0.72).

**Conclusion:** In this prospective observational study, CVVHD induced significant plasma chloride loading, while CVVH resulted in chloride removal, with no significant impact on acid–base balance. CaCl_2_-citrate regional anticoagulation was the main source of chloride loading in CVVHD.

**Reference 1**

[1] Bihari S, Taylor S, Bersten AD. Inadvertent sodium loading with renal replacement therapy in critically ill patients. J Nephrol. (2014) 27:439–44. https://doi.org/10.1007/s40620-014-0041-8.

**Compliance with ethics regulations:** Yes in clinical research.

## FC-289 Renal replacement therapy in burns patients

### Nour Zeineb Jaafar^1^, Hana Fredj^1^, Sarra Zarrouk^1^, Shedha Ben Amor^1^, Bahija Gasri^1^, Imen Jami^1^, Amel Mokline^1^, Amen Allah Messadi^1^

#### ^1^Centre de Traumatologie et des Grands brûlés, Ben Arous, Tunisie

##### **Correspondence:** Hana Fredj (fredjhana@yahoo.fr)

*Annals of Intensive Care* 2023, **13(Suppl 1):**FC-0289

**Rationale:** Renal replacement therapy (RRT) is frequently required to manage critically ill patients with acute kidney injury (AKI). Continuous renal replacement therapy especially continuos veino-veinous hemodiafiltration (CVVHDF) is indicated for hemodynamically unstable patients. The aim of our study was to describe the epidemiological, clinical characteristics and outcome of burned patients who had recourse to RRT and to describe the parameters used.

**Patients and methods/materials and methods:** It was a retrospective, descriptive, monocentric study conducted between January 2019 and august 2022 in the Burn Intensive care department at the trauma and burn center in Tunisia. Were included all patients who required RRT.

**Results:** Forty-two patients had at least one session of RRT from 1439 patients hospitalized in this period (3%). The average age was 51 years. Sex-ratio was 6. The average total body surface area was 49% and the average UBS score was 92. The burns were caused by a domestic accident in 50% of cases, a suicide attempt in 33% of cases. The burns were thermal in 83% of cases and electrical in 12% of cases. At the time of the initiation of the RRT, the indication of RRT was acute kidney injury (90%), metabolic acidosis (88%), hyperkaliemia (50%), anuria with refractory fluid overload (40%) and pulmonary edema (15%). CVVHDF was used in 38 patients and intermittent hemodialysis was used in 4 cases. The site of insertion of the vascular cannula was femoral in majority of cases (80%). The average number of sessions per patient was 2 sessions [1; 4]. The average duration of the sessions was 9 h. The average dose of CVVHDF was 27 ml/kg/h. Unfractionated heparin was chosen as the anticoagulant in all sessions of RRT. CVVHDF sessions had to be stopped in front of hemodynamic instability (40%), severe thrombocytopenia (30%), clotting in the dialysis circuit (24%), technical problem (19%) and hypothermia (20%). RRT has been effective in 45% of cases. Mortality was 92%.

**Conclusion:** The use of RRT is frequent, the technique of choice was CVVHDF, the most frequent indication remains AKI. Unfortunately, this therapy did not improve the prognosis with a high mortality since the majority of patients are in severe septic shock.

**Compliance with ethics regulations:** Yes in clinical research.

## FC-290 Identification and validation of clinical phenotypes in patients admitted for diabetic ketoacidosis in medical intensive care unit: a retro-prospective study

### Firdaous Belkaid^1^, Loubna Bouattane^1^, Kawtar Ziati^1^, Rehab Haffar^1^, Mohammed Abidi^1^, Latifa Oualili^1^, Oussama Ssouni^1^, Tarek Dendane^1^, Amine Ali Zeggwagh^1^, Khalid Abidi^1^

#### ^1^CHU Ibn Sina Rabat, Rabat, Maroc

##### **Correspondence:** Firdaous Belkaid (belk.firdaous@gmail.com)

*Annals of Intensive Care* 2023, **13(Suppl 1):**FC-0290

**Rationale:** The studies on diabetic ketoacidosis (DKA) are rarely reported in critically illness adults. The objectives of our study were to identify the clinical phenotypes of patients with DKA and their correlation to prognosis. To our knowledge, this is the first study in the world investigating the existence and characterization of clinical phenotypes for DKA patients at ICU admission.

**Patients and methods/materials and methods:** This is a retro-prospective study conducted in a Medical Intensive Care Unit from June 1, 2020 to December 31, 2021. Patients admitted for DKA, aged 16 years or more and having stayed in the department for a minimum period of 48 h were included. For identification of clinical phenotypes, we did two-step cluster analysis using both continuous and categorical variables, which provided the optimal number of clusters. We analyzed the bivariate association of each variable of the phenotypes. Then, we did a multinomial logistic regression analysis. For prognostic assessment of the phenotypes, we compared the mortality of patients in the different phenotypes.

**Results:** Out of 903 patients who were admitted in the medical ICU, 123 were admitted for DKA (13.6%). The ICU mortality rate was 22.8%. Two clinical phenotypes were identified: phenotype A (n = 93; 75.6%) and phenotype B (n = 30; 24.4%). Patients with phenotype B were older, had lower respiratory rate, lower mean blood pressure, higher kaliemia, higher serum blood urea and creatinine, lower HbA1C, more anemia, higher serum procalcitonin and less frequently hypophosphoremia than phenotype A. In binomial logistic regression, patients with phenotype B had significantly higher serum creatinine (OR = 1.07; IC95% = 1–1.14; p = 0.04), higher serum procalcitonin (OR = 1.02; IC95% = 1–1.04; p < 0.01) and lower serum HbA1C (OR = 0.66; IC95% = 0.5–0.87; p = 0.003). In this study, the mortality was higher in the phenotype B (46.7%) compared to phenotype A (15.1%) (HR = 3.32 [1.55–7.10], p = 0.002).

**Conclusion:** Patients admitted in ICU with DKA can be classified into two clinical phenotypes that correlate with mortality. Further research is needed to determine the utility of these phenotypes in improving management of DKA patients by providing targeted and personalized treatment.

**Compliance with ethics regulations:** Yes in clinical research.

## FC-291 Association between fluctuations in serum chloride levels and mortality in critically ill patients: a prospective study

### Kawtar Ziati^1^, Maha Berriane^1^, Firdaous Belkaid^1^, Rehab Haffar^1^, Mohammed Abidi^1^, Latifa Oualili^1^, Oussama Ssouni^1^, Tarek Dendane^1^, Khalid Abidi^1^, Amine Ali Zaggwagh^1^

#### ^1^CHU Ibn Sina, Rabat, Maroc

##### **Correspondence:** Kawtar Ziati (kawtar.ziati@yahoo.fr)

*Annals of Intensive Care* 2023, **13(Suppl 1):**FC-0291

**Rationale:** Dyschloremia is frequently observed in ICU patients, but its influence on clinical outcomes still needs to be thoroughly examined. The purpose of this study was to determine the incidence of dyschloremia and to evaluate the association between abnormal serum chloride levels and mortality in medical ICU.

**Patients and methods/materials and methods:** A prospective study was conducted in the Medical ICU of CHUIS-Rabat between November 2020 and October 2021. Adult patients (≥ 16 years old) who were hospitalized for ≥ 72 h and had serum chloride measured on ICU admission were included. First, we proceeded to compare two groups: survivors vs non-survivors. Then we examined the association between dyschloremia at admission and at 72 h, and mortality, and between fluctuations in serum chloride within 72 h delta Cl (Cl72–Cl0) and mortality.

**Results:** 200 patients were included in this study. At admission, 50.5% of patients had dyschloremia, 27.5% with hyperchloremia and 23% with hypochloremia. At 72 h, 74% of patients had dyschloremia, 41.5% with hyperchloremia and 32.5% with hypochloremia. The mortality was 39.5%. In multivariate logistic analysis, a positive fluctuation of chloride within 72 h (delta Cl > 5 mmol/L) was independently associated with mortality (Adjusted odds ratio for delta Cl 5 moml/L = 1.33; IC95% = 1.06–1.66; p = 0.01). However, no association was found between dyschloremia and mortality at admission or 72 h.

**Conclusion:** Dyschloremia, at admission or acquired, is frequently found within MICU patients. The positive fluctuation of serum chloride within 72 h of admission was an independent predictor for mortality. However, serum chloride alterations on admission did not have an impact on mortality.

**Compliance with ethics regulations:** Yes in clinical research.

